# Modular and Stereoselective Synthesis of Cyclobutane *β*‐Amino Alcohols

**DOI:** 10.1002/anie.1530338

**Published:** 2026-08-28

**Authors:** Xinyu Yang, Tomoya Ozaki, Bo Li, Shih‐Yuan Liu

**Affiliations:** ^1^ Department of Chemistry Merkert Chemistry Center Boston College Chestnut Hill Massachusetts USA

**Keywords:** aminoalcohols, azaborines, boron, heterocycles, photoisomerization

## Abstract

Cyclobutane *β*‐amino alcohols are valuable structural motifs, yet their development has lagged behind that of five‐ and six‐membered analogs due to limited synthetic accessibility. In this work, we report a general and modular strategy for the synthesis of diversely substituted cyclobutane *β*‐amino alcohols. The electronic structure of 1,2‐azaborines is leveraged to achieve regioselective mono‐ and difunctionalization at all four carbon positions. The installed functionalities are subsequently translated into cyclobutane *β*‐amino alcohol frameworks through a tandem photoisomerization–hydrogenation–oxidation sequence. This strategy provides access to eight distinct substitution patterns with high levels of modularity and diastereoselectivity.

## Graphical abstract

Late‐stage functionalization and photoisomerization of 1,2‐azaborine leads to a general strategy for the synthesis of diversely substituted cyclobutane *β*‐amino alcohols. The relative stereochemistry of the resulting products is unambiguously defined by the stereospecific and stereoselective nature of the photoisomerization‐hydrogenation‐oxidation sequence, selectively producing a single diastereomer.

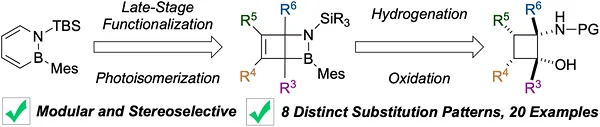

## 1 Introduction

Cyclic *β*‐amino alcohols are important building blocks for the synthesis of biologically active molecules [1, 2, 3, 4, 5, 6] and chiral catalysts [7, 8, 9, 10, 11, 12], as showcased by those well‐documented five‐membered and six‐membered homologues (Scheme 1A). Comparatively, four‐membered *β*‐amino alcohols have been far less explored [13, 14, 15, 16, 17], despite increasing interest toward cyclobutane rings in drug discovery (Scheme 1B) [18]. Arguably, this disparity in attention is unlikely to arise solely from an inherent superiority of five‐ and six‐membered β‐amino alcohols over their four‐membered counterparts but rather reflects a synthetic gap between these moieties. Indeed, the development of cyclobutane *β*‐amino alcohols has been hampered by the lack of general and versatile preparative routes.

**SCHEME 1:**
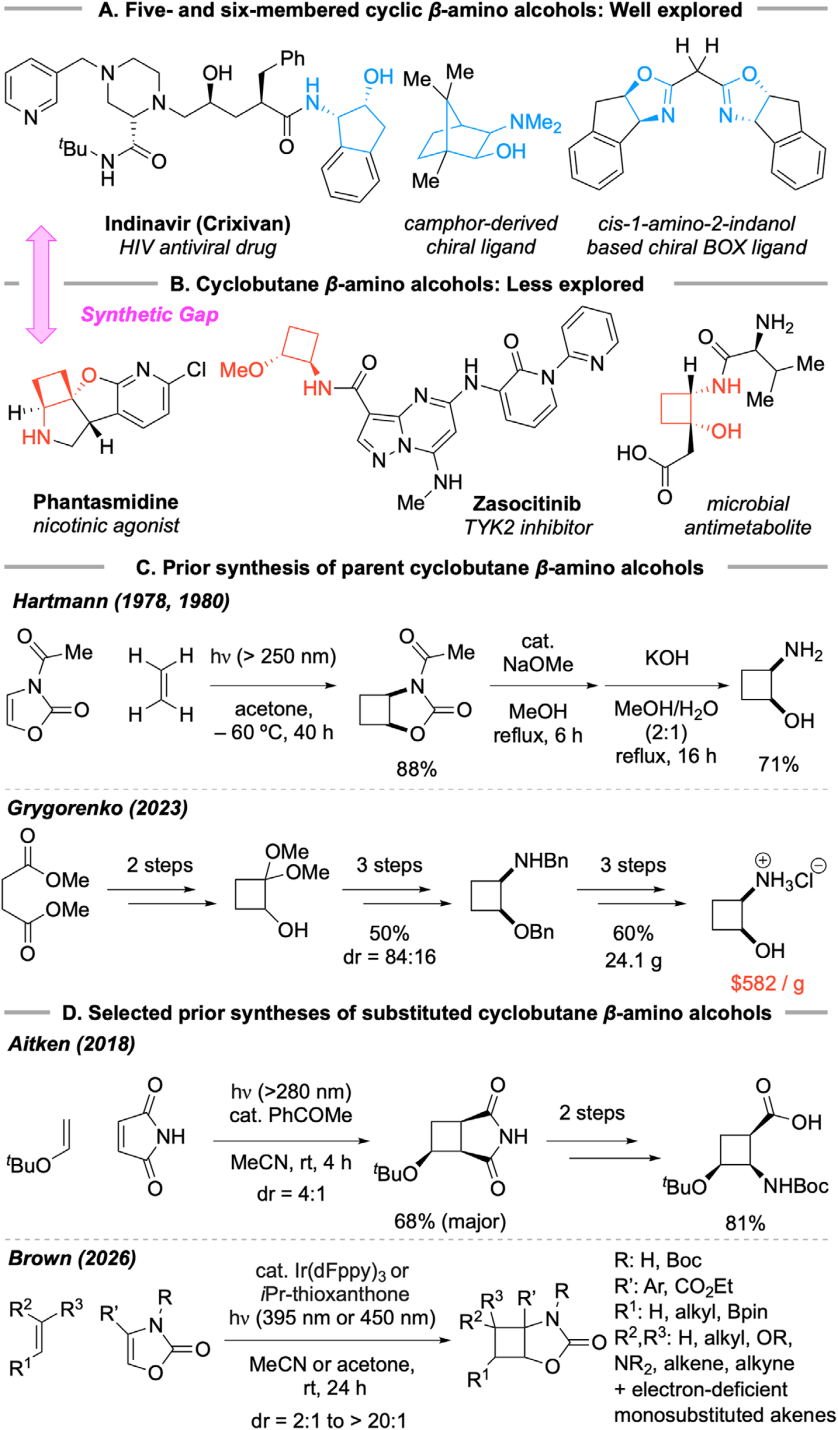
Background summary of cyclobutane *β*‐amino alcohols.

For example, hydroxyamination is among the most powerful strategies for accessing 1,2‐amino alcohols from alkenes and has been successfully applied to cyclopentene and cyclohexene substrates [19, 20, 21, 22, 23]. However, to the best of our knowledge, this transformation has never been successfully extended to cyclobutene substrates.

To date, several methods have been reported for the synthesis of the parent cyclobutane *β*‐amino alcohol, including [2 + 2] photocycloaddition [24, 25, 26, 27, 28, 29], intramolecular cyclization [30, 31, 32, 33], and C–H hydroxylation [34] of cyclobutylamines. In 1978, Hartmann reported the pioneering synthesis of the parent cyclobutane *β*‐amino alcohol via the [2 + 2] photocycloaddition of an oxazolone with ethylene [24], and related intermolecular photocycloadditions have since remained the mainstream synthetic strategy (Scheme 1C, top) [25, 26, 27, 28, 29]. However, the requirement for high‐energy UV irradiation under cryogenic conditions has significantly limited the practical utility and scalability of this approach. More recently, Grygorenko disclosed a highly scalable synthesis, reaching at least a decagram scale, starting from the acyloin condensation of inexpensive and readily available dimethyl succinate (Scheme 1C, bottom) [31]. Despite this significant advance, commercial availability of cyclobutane *β*‐amino alcohols remains limited.

Moreover, as implied by these parent precedents, access to more highly substituted analogs remains even more challenging, especially with respect to achieving high levels of regio‐ and diastereoselectivity for the installation of additional substituents on the cyclobutane skeleton. In certain cases, specific substitution patterns can be accessed using tailored approaches, as elegantly exemplified by Aitken's synthesis of a trisubstituted analog reported in 2018 [27, 28] and Brown's photocycloaddition of oxazolones and alkenes [29] (Scheme 1D). Such strategies rely on substrate control and therefore may lack generality across different substitution patterns. Accordingly, a generally applicable strategy for the synthesis of diversely substituted cyclobutane *β*‐amino alcohols remains a challenging objective and highly desirable.

Our group, along with others, has had a long‐standing interest in the development and application of 1,2‐azaborine, a boron(B)–nitrogen(N) isostere of benzene [35, 36, 37, 38, 39, 40, 41]. As part of a synthesis‐oriented research program, we have exploited these aromatic heterocycles as formal “4C + 1N + 1B” synthons through dearomative functionalization strategies [42, 43, 44]. Within this framework, photoisomerization of 1,2‐azaborine to BN‐Dewar benzene has been explored [45, 46, 47, 48, 49, 50, 51, 52, 53, 54]. In 2018, we demonstrated that aminoborylated cyclobutanes could be accessed through functionalization of BN‐Dewar benzene, valence isomer of 1,2‐azaborine generated upon photoisomerization (Scheme 2A, top) [47]. We subsequently functionalized the boron to access a series of 1,2‐substituted cyclobutane derivatives, including *cis*‐cyclobutane *β*‐amino alcohols following an oxidation of the C‐B bond (Scheme 2A, bottom).

**SCHEME 2:**
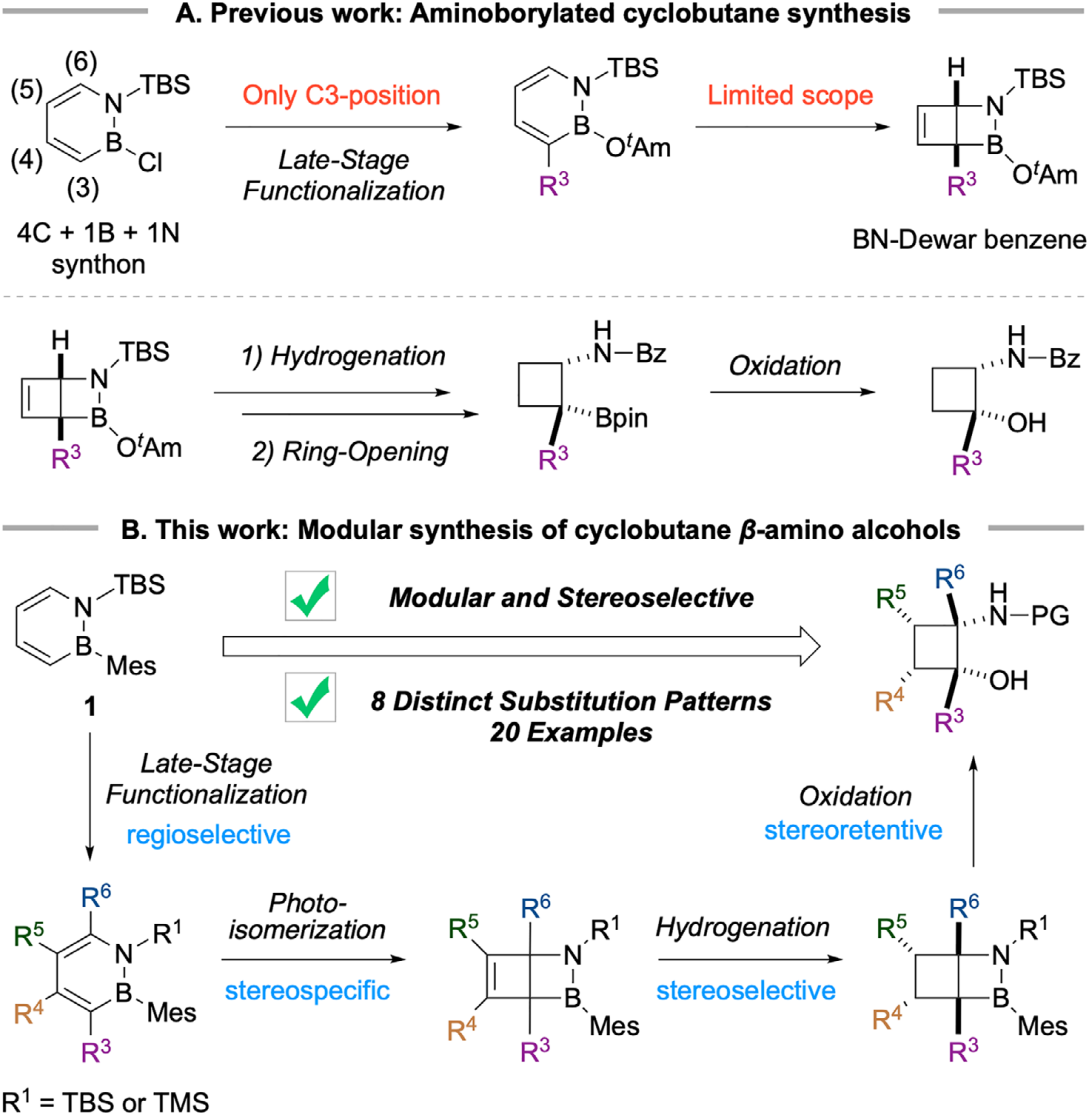
Cyclobutane *β*‐amino alcohols.

Although this prior work serves as a proof‐of‐concept for the present study, key imitations precluded its direct application, i.e., late‐stage functionalization was restricted to the C3 position and was largely limited to simple alkyl substituents. We attribute these limitations to the presence of a labile alkoxy substituent on boron and therefore reasoned that strategic choice of the substituent on boron would be critical to overcoming these challenges. To this end, we selected the mesityl group as a more robust substituent on the boron moiety.

Herein, we provide our full account of the development of a modular, regio‐ and diastereoselective synthetic strategy for cyclobutane *β*‐amino alcohols, enabling access to eight distinct substitution patterns (Scheme 2B). Starting from a readily available *N*‐TBS‐*B*‐Mes‐1,2‐azaborine **1**, a variety of functional groups can be introduced across the C3–C6 positions regioselectively. These functionalities are subsequently translated onto the cyclobutane framework through a tandem photoisomerization–hydrogenation–oxidation sequence, furnishing a diverse array of polysubstituted *cis*‐cyclobutane *β*‐amino alcohols as a single diastereomer.

## 2 Results and Discussion

### 2.1 Development of the Oxidation Conditions for B‐Mes Substrate

Although replacing the boron alkoxy substituent with a more robust mesityl group is expected to ease both late‐stage functionalization and the photoisomerization–hydrogenation sequence by increasing kinetic stability, this modification simultaneously introduces a new challenge in the final oxidation step. Therefore, we initiated our investigations to address this challenge.

For example, we previously demonstrated that hydrogenated intermediate **2**, bearing an alkoxyl substituent on boron, is competent for stereospecific C–B bond oxidation, affording the *cis*‐cyclobutane *β*‐amino alcohol **3** in a one‐pot, two‐step sequence (Scheme 3, Equation 1). However, application of these oxidation conditions to the *B*‐mesityl analog **4** failed to deliver the desired oxidation product **5** (Scheme 3, Equation 2).

**SCHEME 3:**
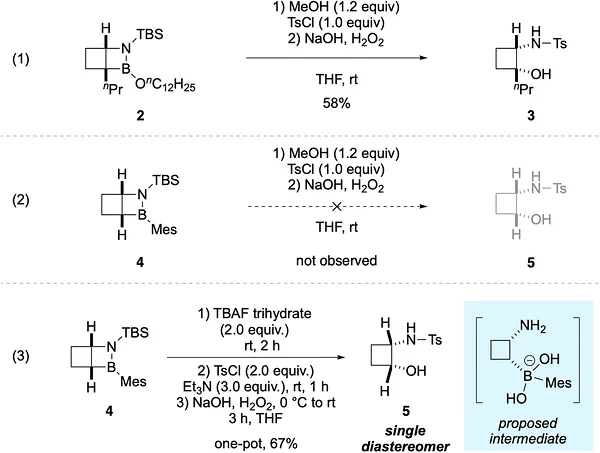
Optimized conditions for the boron oxidation step.

We reasoned that the increased steric congestion at boron in **4** may hamper nucleophilic addition and thereby inhibit B–N bond cleavage and subsequent boron oxidation. To address this issue, we sought to increase the accessibility of the boron center by removing the TBS group on nitrogen. Thus, treatment of **4** with TBAF trihydrate, followed by in situ reprotection of the liberated amine with tosyl chloride and triethylamine and subsequent oxidation in the same pot, proceeded smoothly to furnish the desired amino alcohol **5** in a stereoretentive manner (Scheme 3, Equation 3). We propose that the addition of TBAF trihydrate generates the depicted boron ate complex via TBS deprotection, followed by B–N bond cleavage by H_2_O.

### 2.2 General Route for the Substrate Preparation

Given that *N*‐TBS,*B*‐Mes‐1,2‐azaborine **1** serves as the universal starting point for all regioselective functionalization of 1,2‐azaborines in this study, the overall synthetic utility of the project hinges critically on the availability and scalability of its preparation. To address this issue, we redesigned the synthetic route to this compound from aminoborane **6** via a ring‐closing metathesis–mesityl addition–oxidation sequence, and we achieved significant improvements in both overall yield and operational simplicity relative to our previous methods (Scheme 4) [55]. The revised synthesis of *N*‐TBS‐*B*‐Mes‐1,2‐azaborine **1** is efficient, readily scalable to the decagram scale, can be completed within several hours, and requires only a single column chromatography purification. In contrast, the previous procedure required substantially longer reaction times and involved two distillations and one column chromatography purification (see Scheme S1 for details).

**SCHEME 4:**
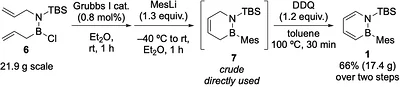
New synthetic route for *N*‐TBS‐*B*‐Mes‐1,2‐azaborine.

Starting with *N*‐TBS‐*B*‐Mes‐1,2‐azaborine **1**, functionalization handles such as Bpin or Br can be readily installed at each of all four carbon positions within two steps, accessing all the monosubstituted 1,2‐azaborine building blocks in a modular and regioselective fashion (Scheme 5) [56]. Electrophilic aromatic substitution of **1** with *N*‐bromosuccinimide (NBS) proceeded selectively at the C3 position to furnish **8** (Scheme 5, Equation 1) [57]. Although C–H borylation of **1** produced a mixture of C4‐ and C5‐borylated regioisomers, iridium‐catalyzed proto‐deborylation is applied to kinetically resolve two regioisomers, enabling the isolation of C4‐Bpin building block **9** (Scheme 5, Equation 2) [58]. Treatment of **1** with a larger borenium cation electrophile afforded the C5‐substituted product, presumably due to the steric shielding of the mesityl group at the C3 position (Scheme 5, Equation 3) [59, 60]. The in situ‐generated C5‐BBr_2_ intermediate was subsequently converted to C5‐Bpin building block **10** by quenching the reaction mixture with pinacol. Finally, removal of the TBS group on nitrogen rendered the C–H borylation selective for the C6 position to produce **11** (Scheme 5, Equation 4) [61]. The aforementioned building blocks with bromide or Bpin functionality could then engage in Negishi or Suzuki cross‐coupling reactions [62]. Notably, with the robust mesityl substituent on the boron, organozinc reagents as well as aqueous basic conditions were well tolerated, allowing the introduction of a wide range of substituents with precise positional control of the BN heterocyclic core.

**SCHEME 5:**
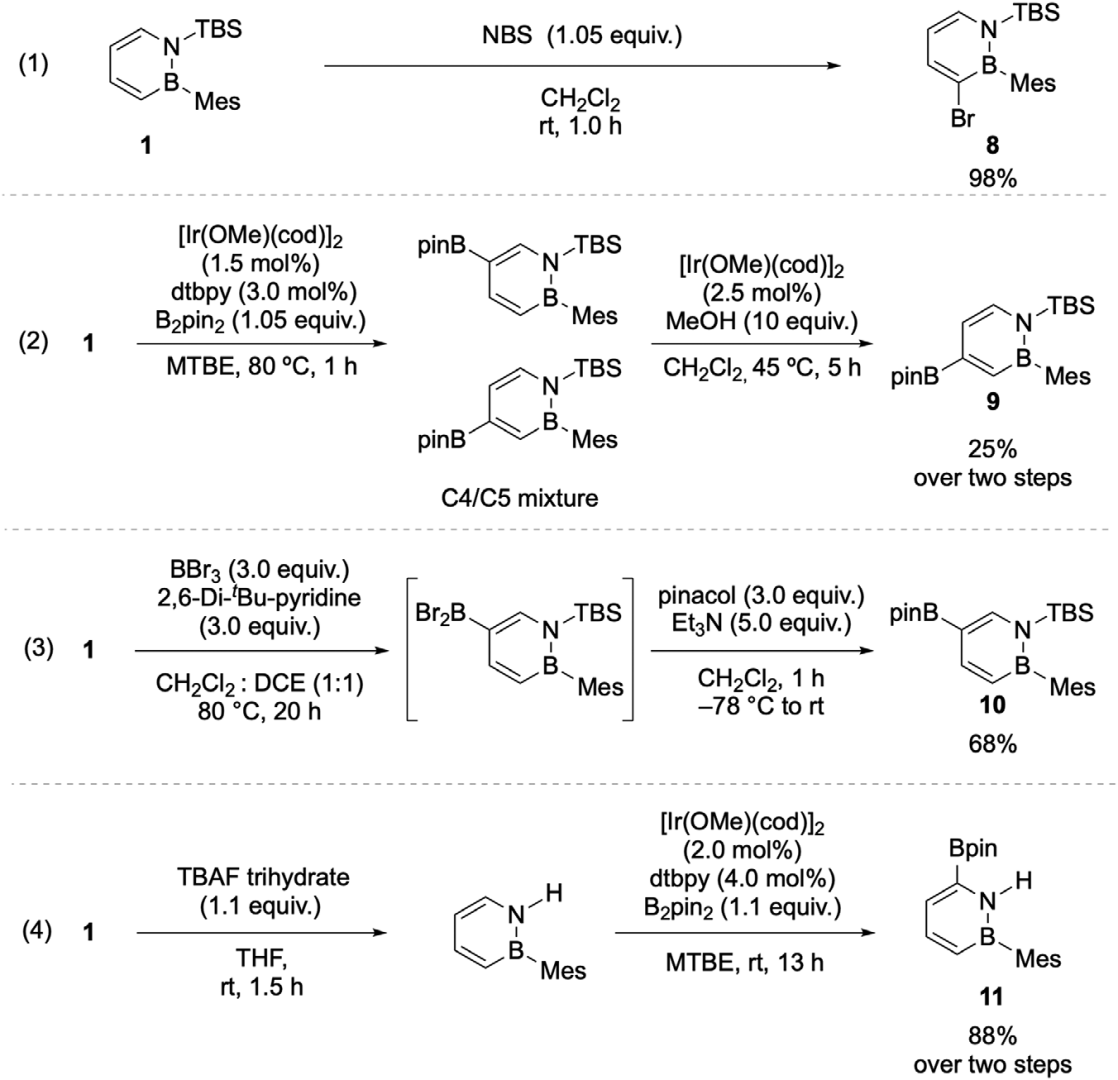
Regioselective preparation of 1,2‐azaborine building blocks **8–11**.

In addition to monofunctionalizations of 1,2‐azaborines, we were also interested in developing difunctionalization strategies, given that the resulting highly substituted cyclobutane *β*‐amino alcohols are particularly challenging to access using existing methods. Notably, C3‐Br‐*N*‐TBS‐*B*‐Mes‐1,2‐azaborine **8** and C6‐Bpin‐*N*‐H‐*B*‐Mes‐1,2‐azaborine **11** could be leveraged as versatile synthons for this purpose (Scheme 6).

**SCHEME 6:**
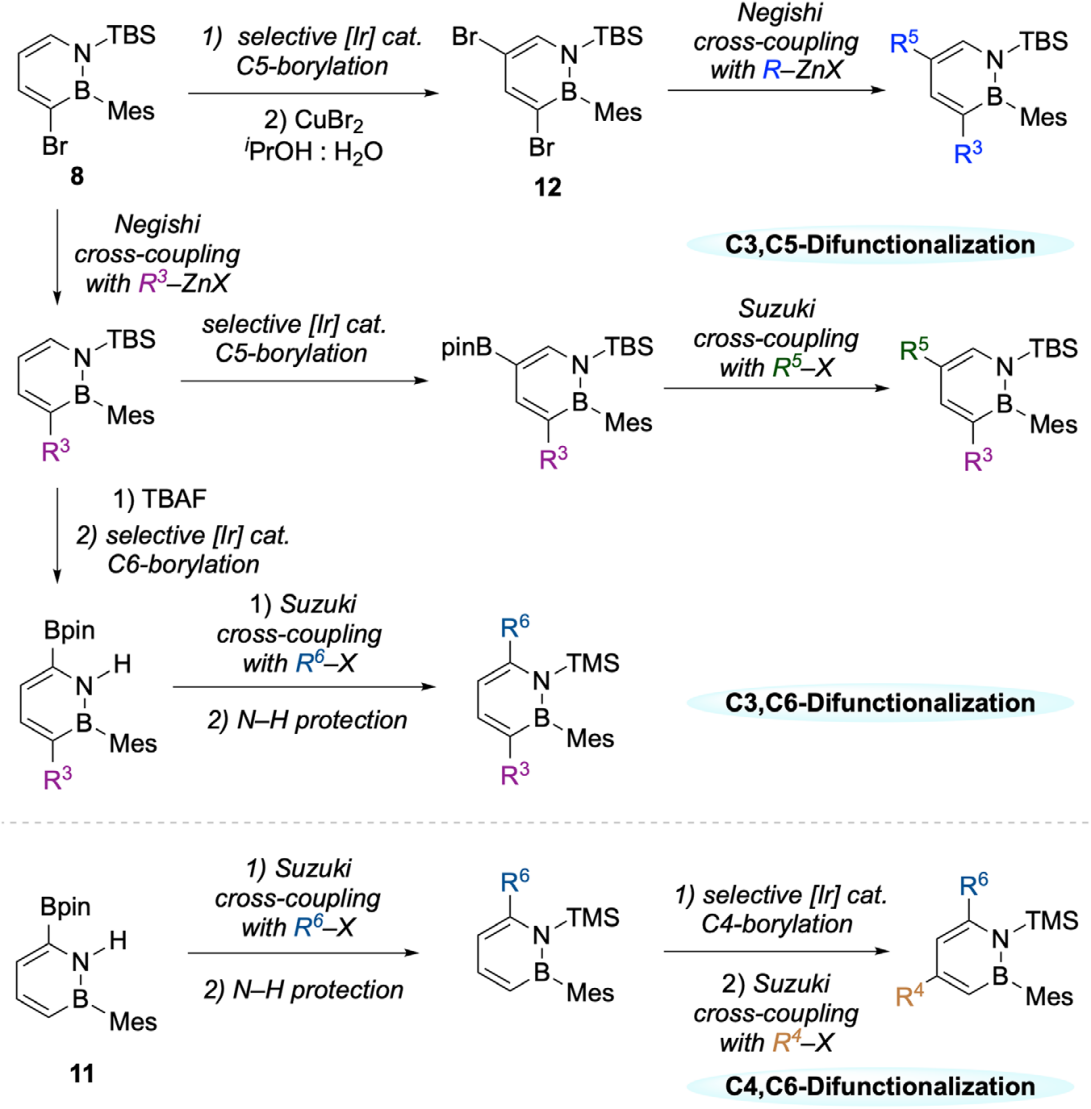
Modular preparation of difunctionalized 1,2‐azaborines.

For instance, starting from **8**, a sequential C5‐borylation–deboronative‐bromination sequence furnished the C3,C5‐dibromo‐1,2‐azaborine **12** as a building block for accessing difunctionalized 1,2‐azaborines bearing identical substituent at the C3 and C5 positions. Alternatively, installation of an initial substituent at the C3 position followed by selective C5‐borylation established a handle for introducing a second, distinct substituent at the C5 position. In contrast, prior removal of the bulky TBS group on nitrogen rendered the C6 position the most activated and accessible site for borylation, affording C3,C6‐difunctionalized 1,2‐azaborines.

Similarly, leveraging the C6 monoborylated building block **11**, we were also able to introduce different substituents at the C4 and C6 positions through a sequence consisting of (1) an initial Suzuki coupling, (2) TMS protection of the nitrogen, (3) C4–H borylation, and (4) a second Suzuki coupling [63]. Based on these modular protocols, a diverse array of difunctionalized 1,2‐azaborines featuring distinct substitution patterns was prepared.

### 2.3 Substrate Scope for the Dewar Isomer Formation

With modular access to a diverse array of mono‐ and difunctionalized 1,2‐azaborines in hand, we next turned our attention to evaluating the scope and versatility of the photoisomerization using the continuous‐flow apparatus adopted in our previous studies (Table 1) [64]. Gratifyingly, a wide range of functional groups and substitution patterns were well tolerated under the photo‐flow conditions. In general, 1,2‐azaborines bearing sp^3^‐hybridized carbon substituents (e.g., alkyl and benzyl groups) at all carbon positions underwent efficient photoisomerization, delivering the corresponding Dewar isomers exclusively. Disubstituted 1,2‐azaborines featuring distinct substitution patterns were also competent substrates (**26a–31a**). Notably, a variety of functional groups were compatible across different positions, including bromide (**16a**), methoxy (**17a**), boronic ester (**19a**), ester (**22a**), and fluoride groups (**23a** and **27a**).

**TABLE 1:** Investigations of photoisomerization of 1,2‐azaborines.

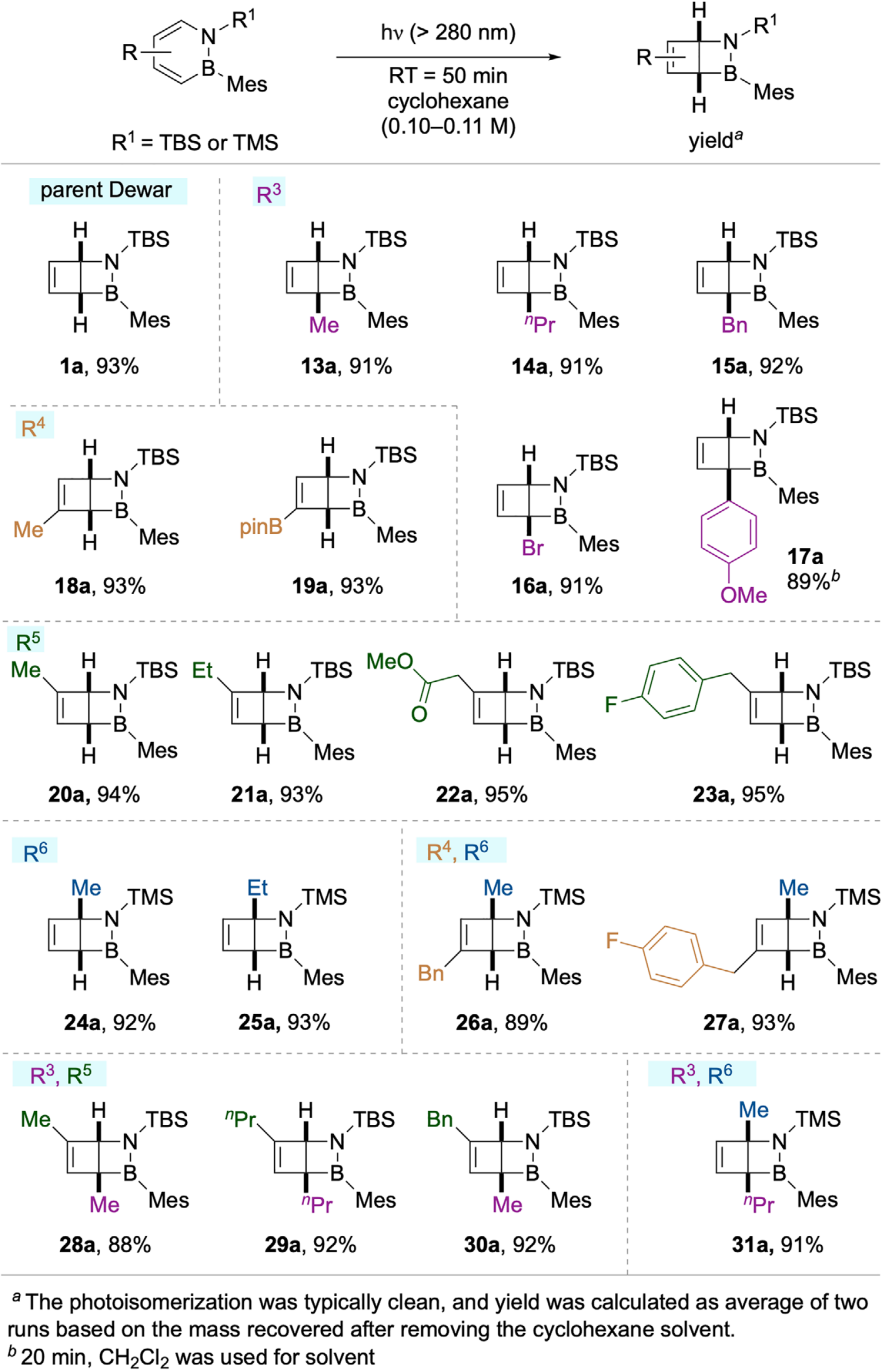

The result of photoisomerization was highly dependent on the position of aryl substitution on the 1,2‐azaborine core. As we previously reported, C5‐aryl‐substituted substrate **32** produced its benzvalene isomer **32a'** as a major product (Scheme 7, Equation 1) [65, 66, 67, 68, 69]. In addition, whereas the C3‐aryl‐substituted substrate underwent efficient photoisomerization to its Dewar isomer **17a** with complete consumption of the starting material (Table 1), the corresponding C4‐ and C6‐aryl‐substituted analogs (**33** and **34**) exhibited poor reactivity, reaching less than 25% conversion to its Dewar isomer (Scheme 7, Equations 2 and 3). Attempts to improve conversion by lowering the substrate concentration or extending the retention time proved ineffective. Moreover, re‐irradiation of the collected reaction mixture did not promote further isomerization. These observations suggest that, for the C4‐ and C6‐aryl‐substituted 1,2‐azaborines, a dynamic photochemical equilibrium between the 1,2‐azaborine and BN‐Dewar benzene isomer may be established.

**SCHEME 7:**
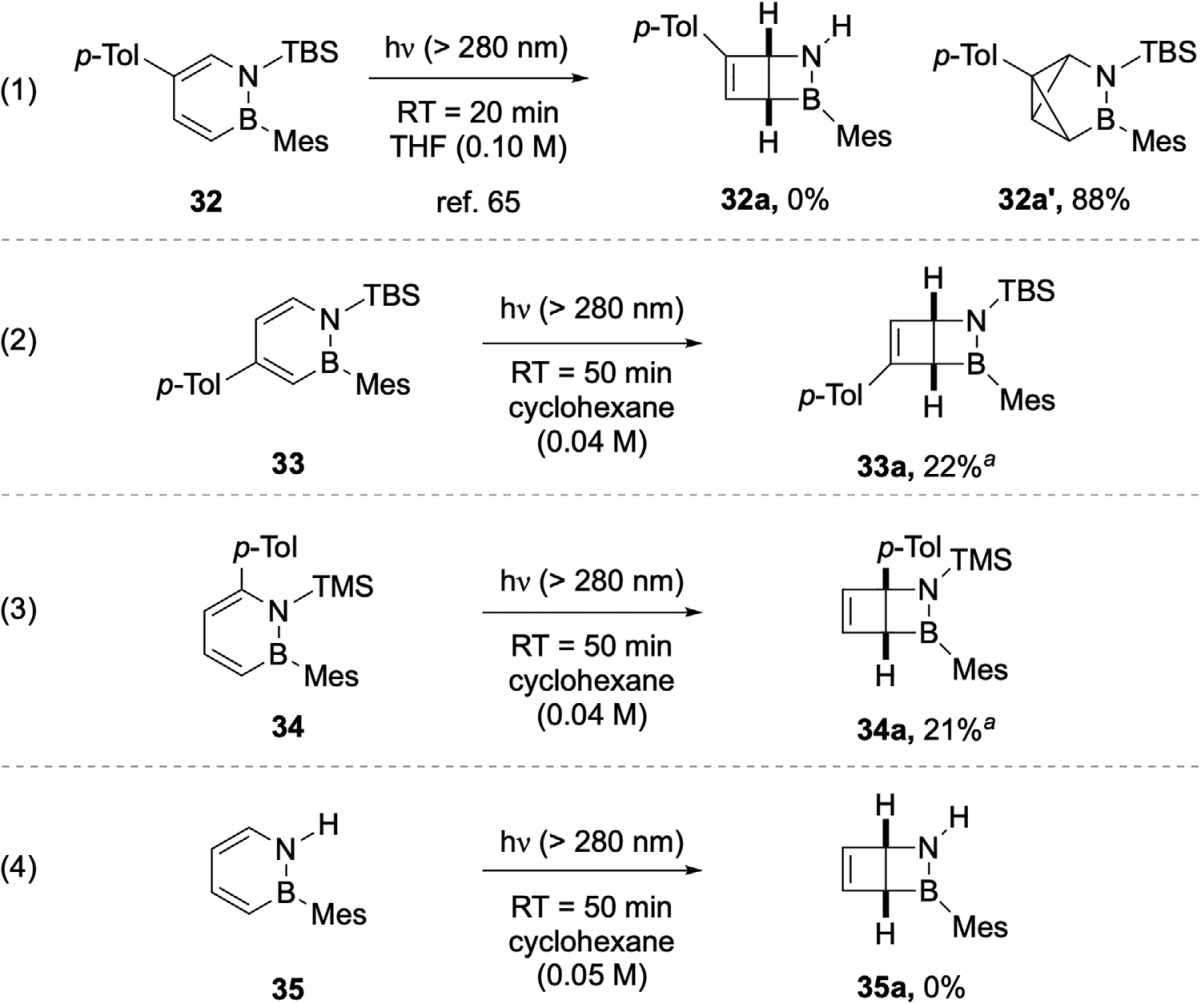
Unsuccessful substrates for Dewar isomer formation. (a) The yield was calculated based on the crude NMR (the ratio between product and the remaining starting material).

Notably, the presence of a silyl protecting group on nitrogen proved essential for the formation of the Dewar isomer at room temperature, as photoisomerization of the corresponding *N*‐H‐*B*‐Mes‐1,2‐azaborine **35** resulted in no detectable Dewar product, with only starting material recovered (Scheme 7, Equation 4). To account for this observation, we hypothesized that rapid thermal cycloreversion of the Dewar intermediate **35a** at room temperature leads to regeneration of the parent 1,2‐azaborine **35**. To corroborate this possibility, we subjected *N*‐TBS‐*B*‐Mes‐BN Dewar **13a** to standard TBS deprotection conditions. In this experiment, only the *N*‐H‐*B*‐Mes‐1,2‐azaborine **35** was obtained, with no Dewar species **35a** detected, supporting the proposed rapid cycloreversion (Scheme 8).

**SCHEME 8:**
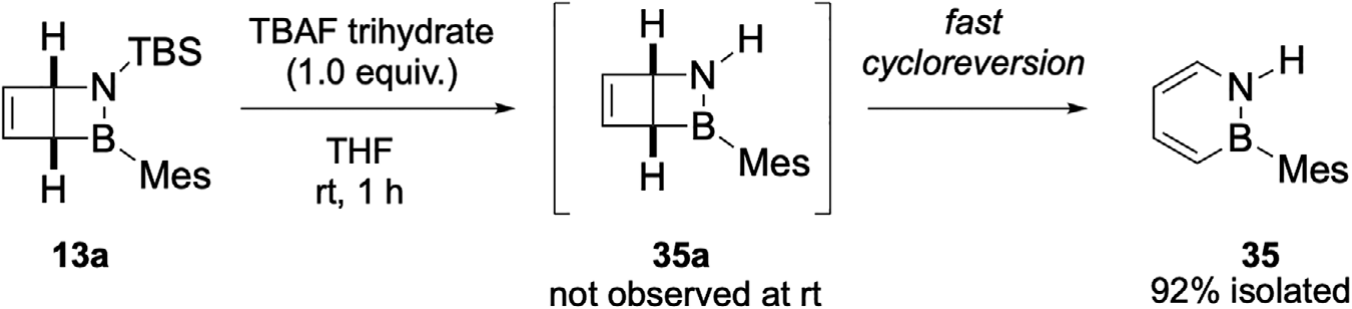
Unsuccessful substrates for Dewar isomer formation.

### 2.4 Development of the Hydrogenation Conditions

Encouraged by the broad scope of the photoisomerization, we next sought an efficient and stereoselective hydrogenation protocol to reduce the olefin of the Dewar isomer. Heterogeneous palladium‐on‐carbon (Pd/C) was identified as an effective catalyst, and the reaction proceeded smoothly in cyclohexane (Scheme 9, Equation 1). Since both the photoisomerization and hydrogenation steps were performed in cyclohexane, and Pd/C can be readily removed by simple filtration, the photoisomerization–hydrogenation sequence can be fully telescoped without solvent exchange. This operational simplicity further enabled direct telescoping into the subsequent oxidation step without intermediate purification. The practicality of this streamlined protocol was demonstrated by the scalable synthesis of the unsubstituted cyclobutane *β*‐amino alcohol (Scheme 9, Equation 2).

**SCHEME 9:**
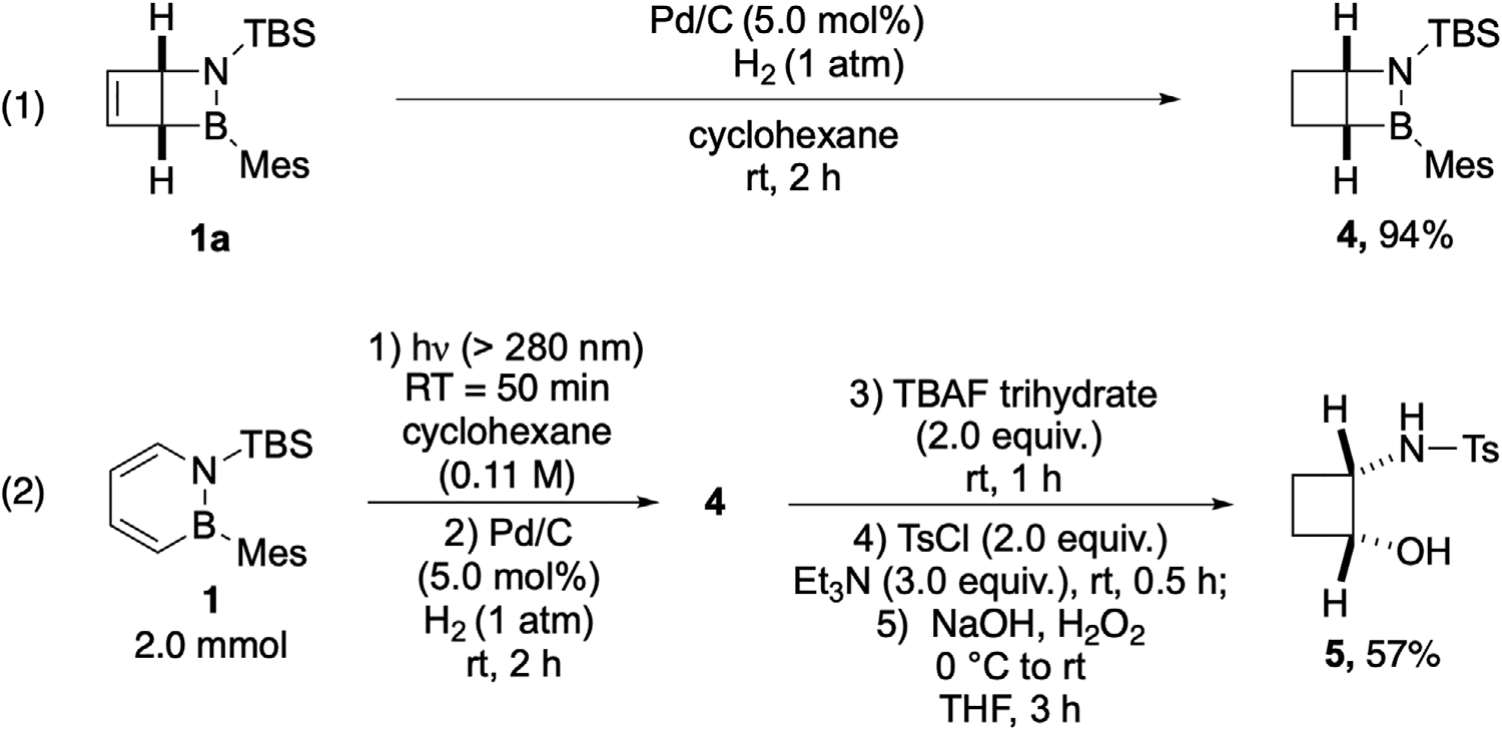
Telescoped hydrogenation and oxidation conditions.

As anticipated, C4‐ and C5‐substituted Dewar isomers bearing sterically hindered alkenes proved more challenging to hydrogenate under standard hydrogen pressure (1 atm). To establish a more general protocol for these hindered Dewar substrates, hydrogenation was therefore conducted under elevated hydrogen pressure (250 psi). Nevertheless, we found that certain substrates remained too sterically congested to undergo hydrogenation even under these high‐pressure conditions (see Figure S1 for details).

### 2.5 Substrate Scope for Cyclobutane *β*‐Amino Alcohols

Taking advantage of the clean hydrogenation, we next telescoped the newly developed oxidation protocol with the hydrogenated intermediates, enabling the stereospecific synthesis of a diverse array of polysubstituted cyclobutane *β*‐amino alcohols (Table 2). A broad range of functional groups originating from different positions of the 1,2‐azaborine precursors were successfully translated onto the cyclobutane *β*‐amino alcohol framework, including hydroxyl groups (derived from boronic esters, **19b**), esters (**22b**), and fluorinated benzyl groups (**23b** and **27b**). Although the origin of this behavior remains unclear, the C3‐*p*‐OMe‐Ph‐substituted substrate **17a** afforded a complex mixture under the standard conditions, and no desired product **17b** was detected. Notably, methyl substituents could be systematically “walked” to all four positions of the cyclobutane ring (**13b**, **18b**, **20b**, **24b**, **28b**) [70, 71], highlighting the high modularity of this strategy. Moreover, the relative stereochemistry of the resulting polysubstituted cyclobutane *β*‐amino alcohols was unambiguously defined by the stereospecific and stereoselective nature of the sequence, producing one single diastereomer for all examined substrates. Specifically, the stereochemistry at C3 and C6 was determined by stereospecific photoisomerization, affording *ci*s‐amino alcohols. The stereochemistry at C4 and C5 was established in the highly stereoselective hydrogenation step, and the subsequent boron oxidation proceeded stereospecifically. Notably, no epimerization was observed during either the hydrogenation or oxidation steps.

**TABLE 2:** Substrate scope for cyclobutane *β*‐amino alcohols.

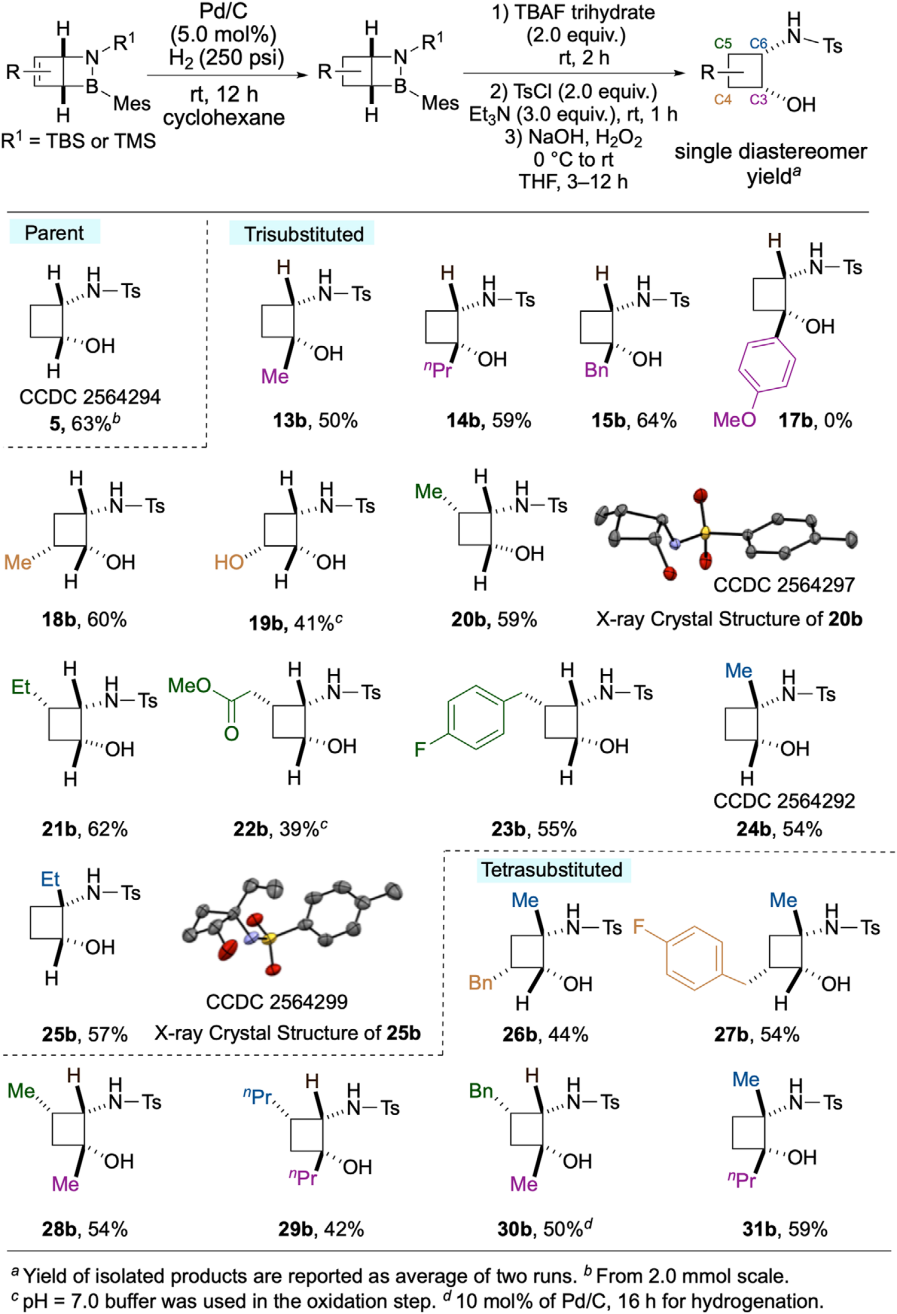

During our investigation of the oxidation step of the C3‐brominated substrate **36** (derived from hydrogenation of **16a**), we anticipated the formation of cyclobutanone **37** via *α*‐elimination of the bromide group from **16b**. Instead, we observed the unexpected formation of product **38**, arising from the migration of the mesityl group from boron to the C3 position (Scheme 10A). The stereochemistry of **38** was unambiguously assigned by x‐ray crystallographic analysis. Based on the observed trans relationship between the amine and mesityl substituents, we propose the following reaction mechanism (Scheme 10B, Mechanism 2). From intermediate **Int A**, base‐mediated formation of the aziridine **Int B** occurs initially. Subsequent migration of the mesityl group from boron to the C3 carbon results in aziridine ring opening [72, 73, 74]. Then, final oxidation of boron furnishes product **38**. Although direct mesityl migration from **Int A** (Scheme 10B, Mechanism 1) is also a conceivable pathway, this mechanism is ruled out because it would lead to diastereomer **39**, which is inconsistent with the experimentally observed stereochemistry of the product **38**. In addition to oxidation of the proposed boronic acid intermediate, this species can also be trapped by simple pinacol addition to afford the aminoborylated cyclobutane **40**.

**SCHEME 10:**
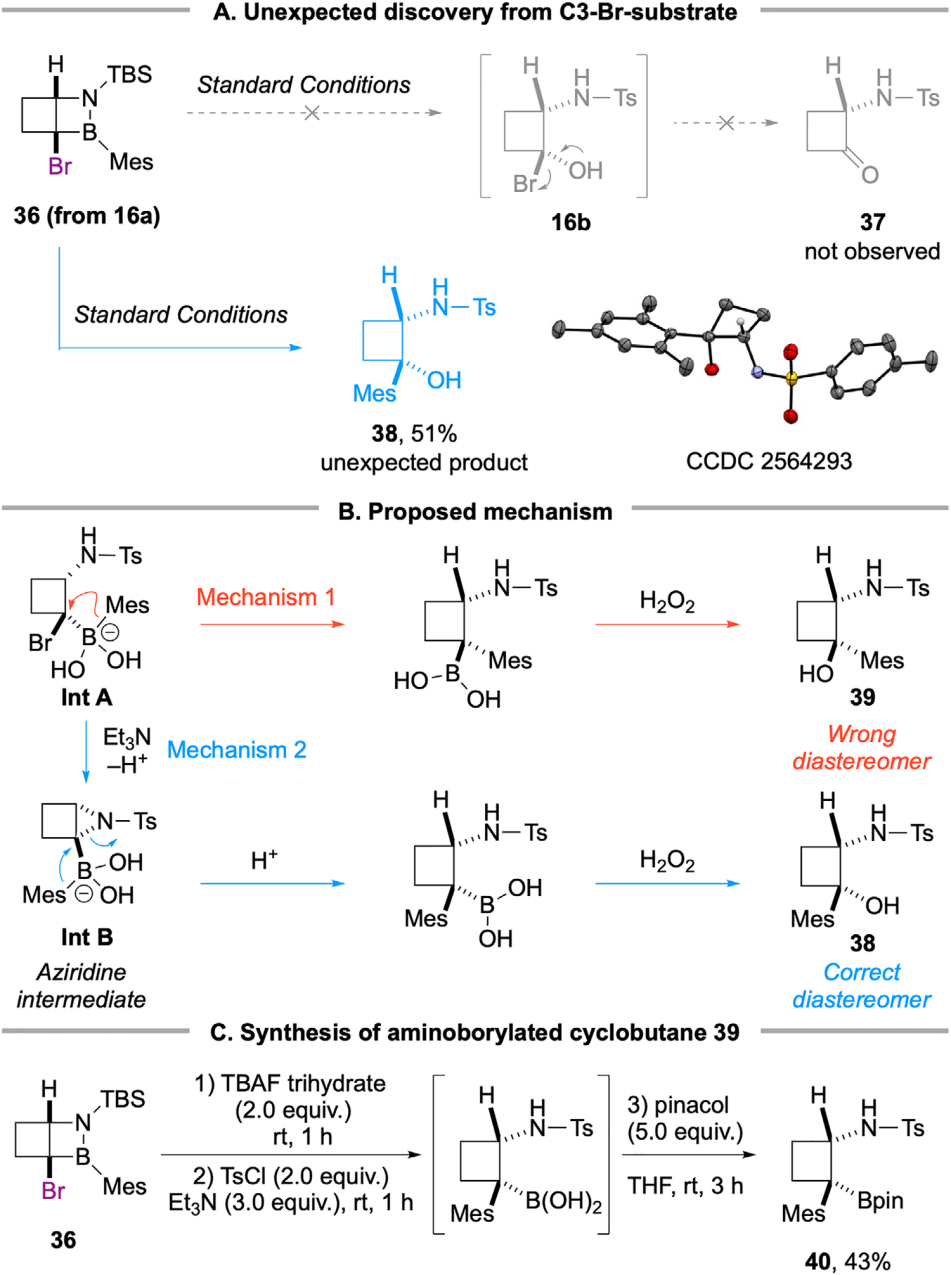
Unexpected discovery of the mesityl group migration.

### 2.6 Derivatizations of Amine and Alcohol Functionality

Cyclobutane *β*‐amino alcohols are not only valuable target molecules but also versatile building blocks for accessing a wide range of 1,2‐difunctionalized cyclobutanes [31]. To demonstrate the synthetic versatility of our method, we first explored the installation of different protecting groups on the amino functionality. Under the standard oxidation conditions developed in this work, a range of electrophiles, such as benzoyl chloride (BzCl) or Di‐tert‐butyl dicarbonate (Boc_2_O), could be used in place of tosyl chloride (TsCl) to furnish the corresponding protected products **42** and **43** (Scheme 11). Additionally, deprotection of the Boc group enabled access to the unprotected amino alcohol **44**.

**SCHEME 11:**
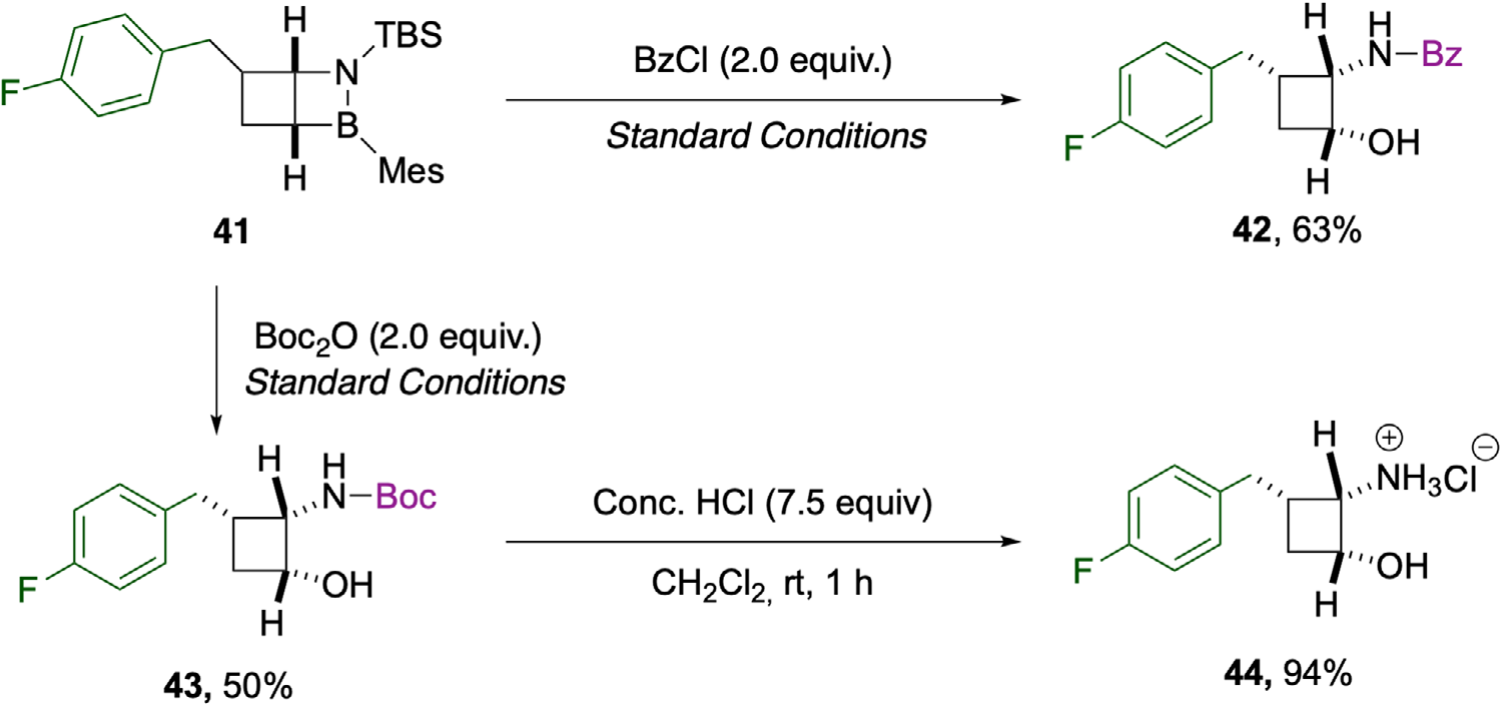
Further derivatizations of the amino group.

In addition, derivatization of the alcohol functionality was demonstrated using **24b** as a model substrate (Scheme 12). Following installation of a tosyl group on the alcohol to produce **45**, addition of *p*‐tolylmagnesium bromide furnished cyclopropane **46**. For the formation of product **46**, we propose a mechanism similar to the quasi‐Favorskii rearrangement: [75, 76] deprotonation and carbon migration furnish the cyclopropyl imine, which is then followed by the addition of another equivalent of Grignard reagent.

Although unprotected amino alcohols are generally challenging substrates for oxidation to ketones [77], we were pleased to find that IBX efficiently oxidized the alcohol of **24b** to deliver the corresponding α‐amino cyclobutanone **47** in a smooth manner [78]. Subsequent reduction of **47** with NaBH_4_ proceeded diastereoselectively to afford the trans‐cyclobutane *β*‐amino alcohol **48**. This two‐step oxidation–reduction sequence therefore enables inversion of the stereogenic center at the alcohol position.

**SCHEME 12:**
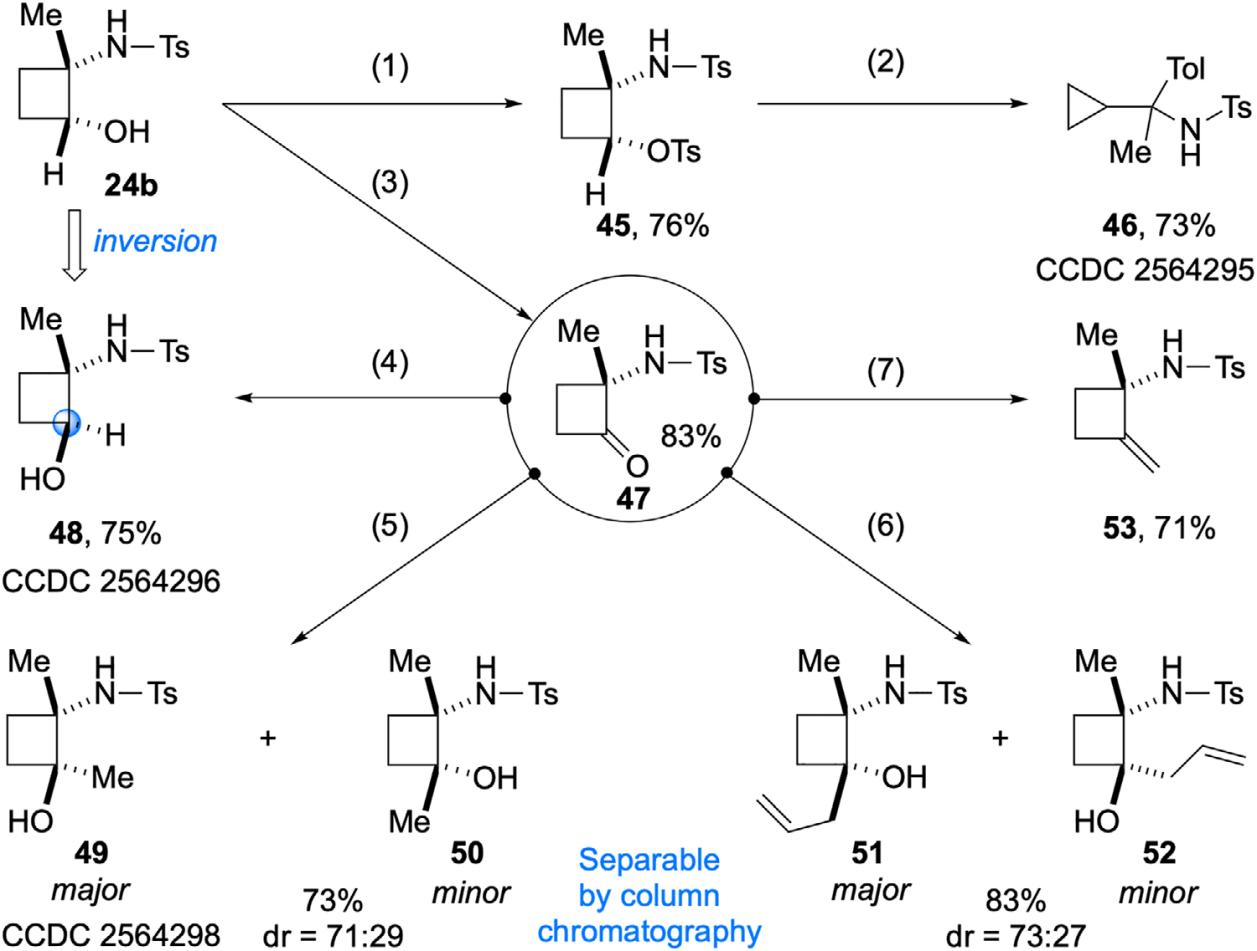
Further derivatizations of the alcohol group. (1) TsCl (2.0 equiv.), Et_3_N (4.0 equiv.), rt, CH_2_Cl_2_, 48 h. (2): Tol‐MgBr (5.0 equiv.), rt, THF, 2 h. (3) IBX (1.2 equiv.), EtOAc, 80°C, 2 h. (4) NaBH_4_, rt, THF, 2 h. (5) MeLi (5.0 equiv.), Et_2_O, rt, 2 h. (6) Allyl‐MgCl (5.0 equiv.), THF, rt, 2 h. (7) Tebbe reagent (2.0 equiv.), THF, rt, 1 h.

In addition, cyclobutanone **47** readily reacted with both Grignard and organolithium reagents to generate both *cis*‐ and *trans*‐cyclobutane *β*‐amino alcohols (**49–52**). This strategy provides access to adjacent quaternary stereogenic centers bearing nitrogen and oxygen substituents on each carbon. Notably, the resulting diastereomers were readily separable by silica gel column chromatography (see Figure S2 for details). Finally, treatment of **47** with Tebbe's reagent enabled conversion of the ketone into the corresponding terminal alkene **53** [79, 80].

## 3 Conclusion

In summary, we have developed a predictable and modular synthetic toolbox for substituted cyclobutane *β*‐amino alcohols. By capitalizing on regioselective mono and difunctionalizations of 1,2‐azaborines, we established a tandem photoisomerization‐hydrogenation‐oxidation protocol that converts 1,2‐azaborines into cyclobutane *β*‐amino alcohol motifs. Through translation of diverse functionalities from the 1,2‐azaborine core to the cyclobutane framework, this strategy provides access to eight distinct substitution patterns with high levels of modularity and stereoselectivity. We believe that this approach will facilitate access to challenging, highly substituted cyclobutane targets.

## Author Contributions

All authors have given approval to the final version of the manuscript.

## Conflicts of Interest

The authors declare no conflicts of interest.

## Supporting information




**Supporting File**: anie74508‐sup‐0001‐SuppMat.pdf.

## Data Availability

The data that support the findings of this study are available on request from the corresponding author. The data are not publicly available due to privacy or ethical restrictions.
