# Supplementary material for: Modular and Stereoselective Synthesis of Cyclobutane β‐Amino Alcohols
Source: Angew Chem Int Ed Engl. 2026 Aug 28;65(43):e1530338. doi: 10.1002/anie.1530338 (PMC13676385; doi:10.1002/anie.1530338)
Supplement: Supplementary file 1 [file ANIE-65-e1530338-s001.pdf]

Supporting Information for

Modular and Stereoselective Synthesis of Cyclobutane *β*-Amino Alcohols

Xinyu Yang,‡ Tomoya Ozaki,‡ Bo Li, and Shih-Yuan Liu*

Email : shihyuan.liu@bc.edu

[1] *Department of Chemistry, Boston College, Chestnut Hill, Massachusetts 02467-3860, United States*

Index **Page**

General Considerations S-2
Synthesis of 1,2-Azaborine Substrates S-3
General Procedure for Photoisomerization S-28
General Procedure for the Sequential Hydrogenation and Oxidation S-38
Unsuccessful Substrates S-52
Mesityl Group Migration S-52
Product Derivatization S-55
X-ray Crystallographic Data S-67
References S-75
NMR Spectra S-76
2D NMR Spectra for Stereochemistry Determination S-187

General Considerations

All oxygen- and moisture-sensitive manipulations were carried out under an inert atmosphere using either standard Schlenk technique or a nitrogen-filled glovebox.

Bulk volumes of diethyl ether, dichloromethane, pentane, and tetrahydrofuran were passed through an alumina column and dispensed from a solvent purification system under argon. Unless otherwise noted, all reagents were obtained from commercial sources (TCI, Sigma-Aldrich, Strem, Acros, Oakwood, Fisher) and used as received. The following compounds were purified prior to use: $CDCl_3$ was purchased from Cambridge Isotope Laboratories and was passed through K_2CO_3 plug and distilled over P_2O_5 under N_2 and stored in a Schlenk flask containing 4Å molecular sieves prior to use. Pinacol was purchased from Acros and recrystallized from cold diethyl ether. Pd(dppf)Cl_2 was purchased from Sigma-Aldrich. Pd(*t*-Bu_3P)$_2$ catalyst was purchased from Strem. Tebbe's reagent was prepared according to reported procedures.[1]

All work-up and purification procedures were performed with reagent grade solvents (purchased from Fisher Scientific) in air unless otherwise noted. ^{1}H, ^{11}B, ^{13}C, and ^{19}F NMR spectra were recorded on Varian 500 or 600 MHz spectrometers or a Bruker AVANCE NEO 500 MHz spectrometer at the Boston College nuclear magnetic resonance facility. Data are reported as follows: chemical shift, integration, multiplicity (s = singlet, d = doublet, t = triplet, q = quartet, br = broad, m = multiplet), and coupling constant (Hz). ^{1}H and ^{13}C spectra were internally referenced to residual solvent peaks (^{1}H $CDCl_3$: δ = 7.26 ppm; acetone-d_6: δ = 2.05 ppm; DMSO-d_6: δ = 2.50 ppm, ^{13}C $CDCl_3$: δ = 77.16 ppm; DMSO-d_6: δ = 39.52 ppm); ^{11}B spectra were externally referenced to a standard of BF_3•Et_2O (δ = 0.0 ppm). In ^{13}C NMR analysis, peaks for carbon atoms adjacent to a boron center were generally not observed owing to quadrupolar broadening.

The flow photoreactor was assembled according to previously reported set-up.[2] The hydrogenation was carried out in series 4760 mini non-stirred pressure vessel from the Parr Instrument Company. All IR spectra were measured on a Bruker Alpha-P FT-IR equipped with a single crystal diamond ATR module, and values are reported in cm^{-1}. High-resolution mass spectrometry (HRMS) data was generated in Boston College facilities using Direct Analysis in Real Time (DART) technique on a JEOL AccuTOF DART spectrometer. Single-crystal X-ray diffraction data were obtained at the X-ray Crystallography Center at Chemistry Department of Boston College with a Bruker Kappa Apex Duo fully automated diffractometer.

Synthesis of 1,2-Azaborine Substrates

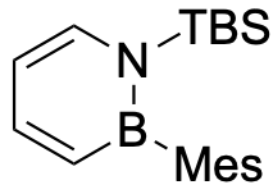
1

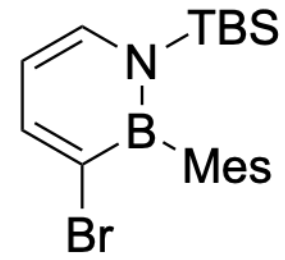
8

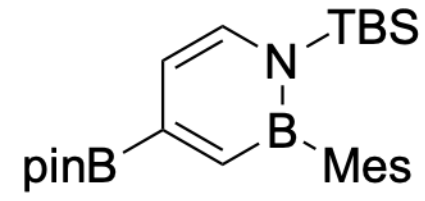
9

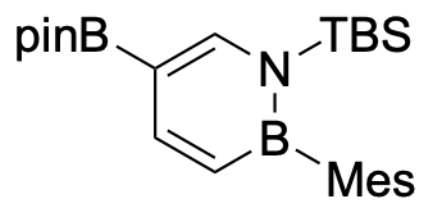
10

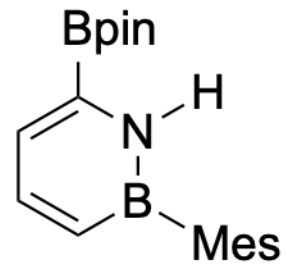
11

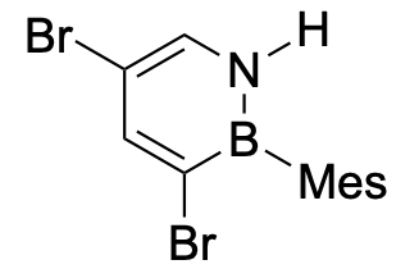
12

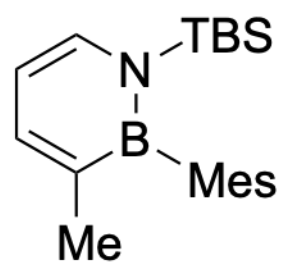
13[a]

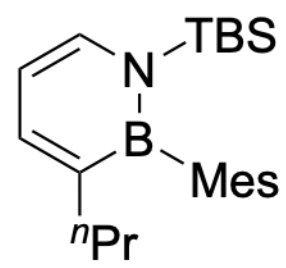
14

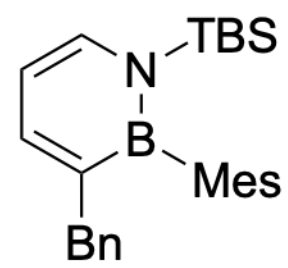
15

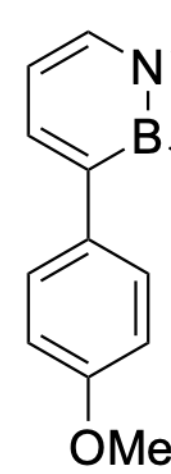
17

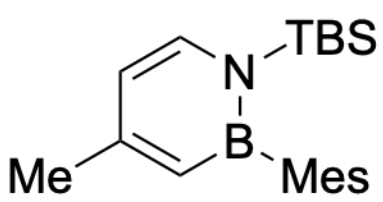
18

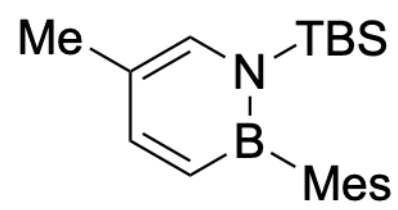
20

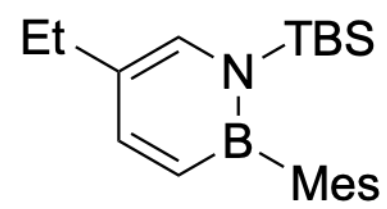
21

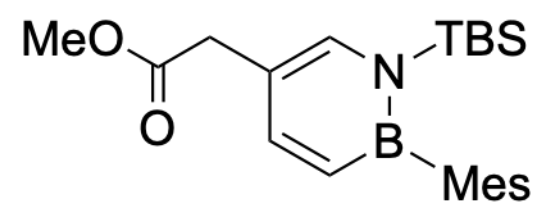
22

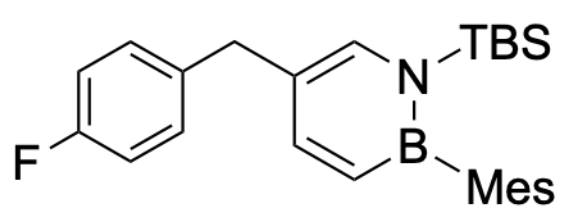
23

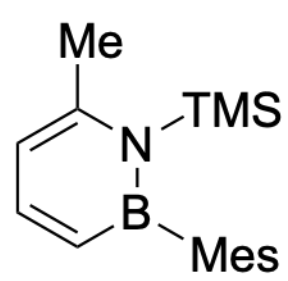
24

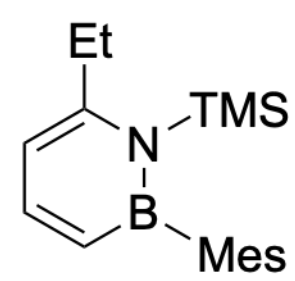
25

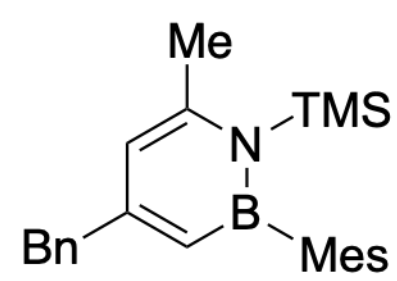
26

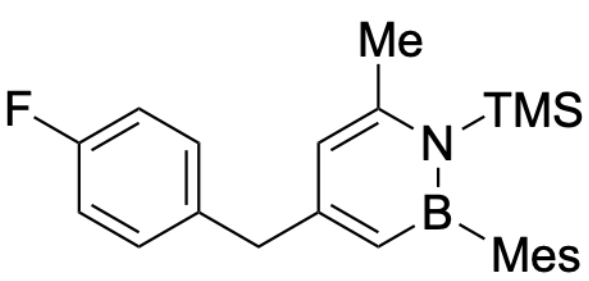
27

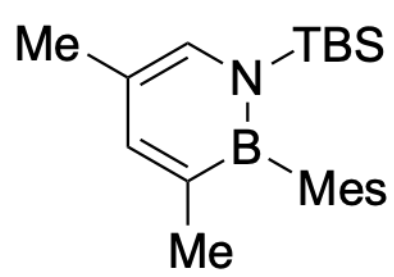
28

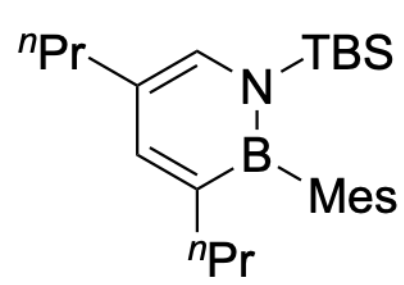
29

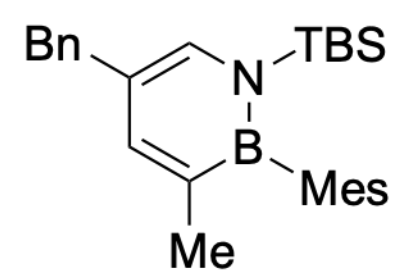
30

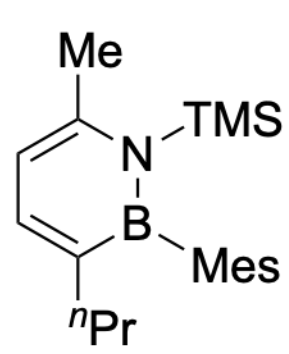
31

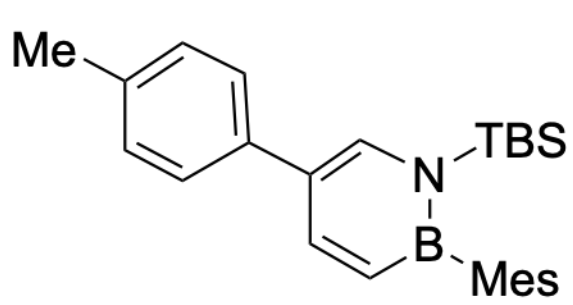
32

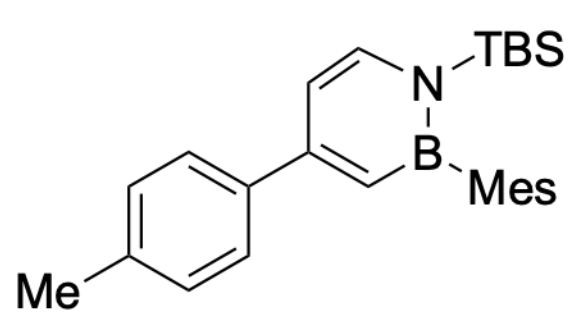
33

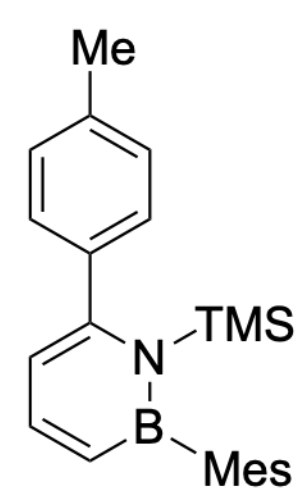
34

C4-Bpin-1,2-azaborine **9**[3], C5-Bpin-1,2-azaborine **10**[4], and C5-*p*-Tol-1,2-azaborine **32**[4] were prepared according to previously reported procedures and the characterization data obtained were consistent with those reported in the literature.

Synthesis of *N*-TBS-*B*-Mes-1,2-azaborine **1**

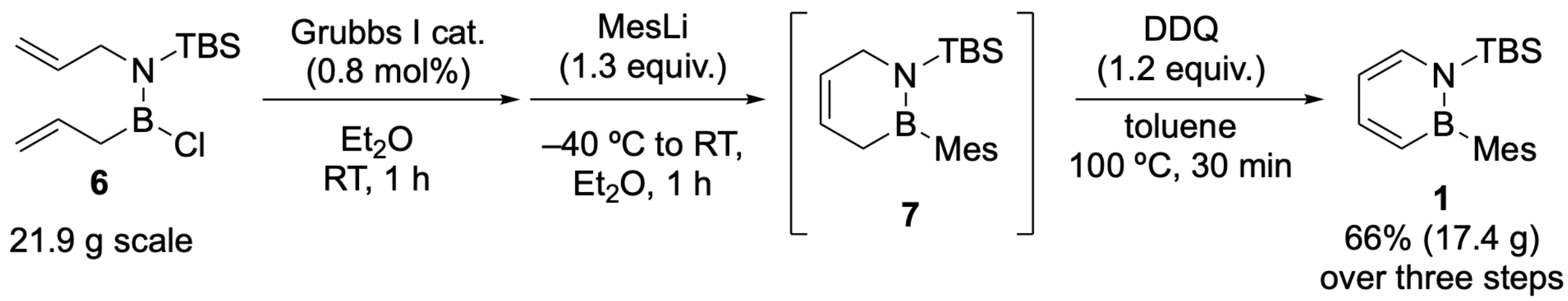

Ring-closing metathesis and MesLi addition steps:

In a glove box, an oven-dried 500 mL round bottom flask was charged with stir bar, compound **6**[2] (21.9 g, 85.0 mmol, 1.00 equiv.), and Et_2O (30 mL). Then, Grubbs I catalyst (559 mg, 0.680 mmol, 0.80 mol%) was added portionwise, and evolution of ethylene gas was observed. The reaction mixture was allowed to stir at room temperature for 1 hour. At the conclusion of the reaction, precooled Et_2O (120 mL, stored at –40 ºC in the glovebox freezer) and solid MesLi (13.9 g, 110 mmol, 1.30 equiv.) were added portionwise. (Caution! Addition of MesLi in large portions can result in significant heat generation and refluxing of diethyl ether.) The mixture was stirred at room temperature for 1 hr. Upon completion, the reaction mixture was filtered through a pad of silica under air. The filtrate was concentrated and the resulting crude material **7** was used directly in the subsequent oxidation step.

Oxidation step:

In a glove box, an oven-dried 500 mL round bottom flask was charged with stir bar, the crude mixture from the previous step, toluene (100 mL), and 2,3-dichloro-5,6-dicyano-*p*-benzoquinone (23.1 g, 102 mmol, 1.20 equiv.). The reaction mixture was allowed to stir at 100 ºC for 30 minutes.

At the conclusion of the reaction, the reaction mixture was filtered through a pad of silica twice. The filtrate was concentrated and purified by column chromatography using hexane to afford product **1** (17.4 g, 55.9 mmol, 66%) as a white solid.

***N*-TBS-*B*-Mes-1,2-azaborine 1**

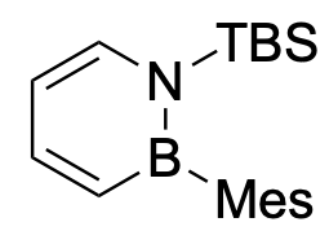

^{1}H NMR (500 MHz, $CDCl_3$) δ 7.57 (dd, *J* = 10.9, 6.3 Hz, 1H), 7.45 (d, *J* = 6.8 Hz, 1H), 6.80 (s, 2H), 6.64 (d, *J* = 10.9 Hz, 1H), 6.41–6.37 (m, 1H), 2.30 (s, 3H), 2.09 (s, 6H), 0.92 (s, 9H), 0.01 (s, 6H); **^{13}C NMR** (126 MHz, $CDCl_3$) δ 142.7, 139.0, 138.4, 136.5, 127.0, 111.6, 27.6, 23.6, 21.3, 19.4, –3.14; **^{11}B NMR** ($CDCl_3$, 160 MHz) δ 39.3.

As a comparison, the previous synthetic route to *N*-TBS-*B*-Mes-1,2-azaborine **1** is shown here (Scheme S1).

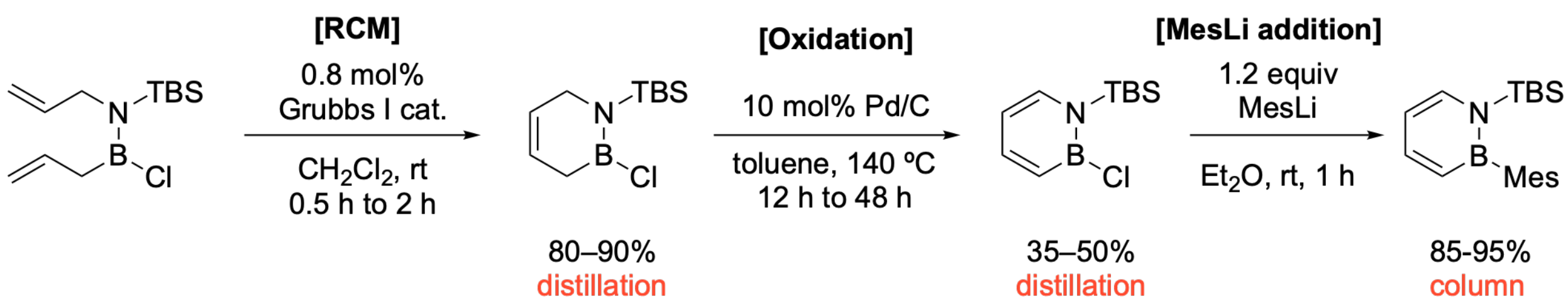

Scheme S1. Previously reported synthetic route to *N*-TBS-*B*-Mes-1,2-azaborine **1**.

<u>Synthesis of C3-Br-1,2-azaborine **8**</u>

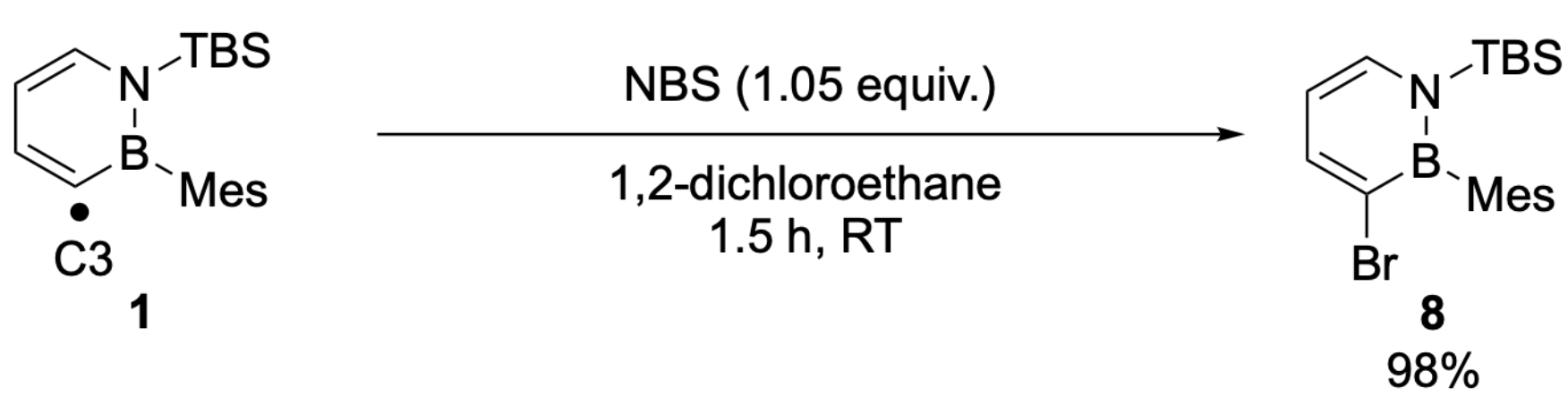

A 250 mL round bottom flask was charged with a magnetic stir bar, azaborine **1** (3.00 g, 9.64 mmol, 1.00 equiv.), N-Bromosuccinimide (1.80 g, 10.1 mmol, 1.05 equiv.), and 30 mL 1,2-dichloroethane. The reaction mixture was then allowed to stir for 1.5 hours. At the conclusion of the reaction, the reaction mixture was concentrated *in vacuo* and purified by silica gel

chromatography using gradient of 5% CH_2Cl_2 in hexane to obtain the product **8** as white solid (1.45 g, 4.60 mmol, 88%).

C3-Br-1,2-azaborine 8

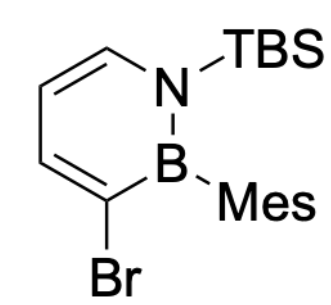

^{1}H NMR ($CDCl_3$, 500 MHz) δ 7.95 (d, J = 7.1, 1H), 7.52 (d, J = 7.1, 1H), 6.87 (s, 2H), 6.34 (t, J = 6.9 Hz, 1H), 2.37 (s, 3H), 2.16 (s, 6H), 1.00 (s, 9H), 0.06 (s, 6H); **^{13}C NMR** ($CDCl_3$, 126 MHz) δ 144.3, 138.9, 137.9, 137.0, 127.0, 111.6, 27.6, 22.9, 21.5, 19.3, –3.0 (the signals for the two carbons adjacent to boron were not observed); **^{11}B NMR** ($CDCl_3$, 160 MHz) δ 39.3; **IR** 3060, 2951, 2860, 1612, 1527, 1388, 1322, 1264, 1220, 1107,1030, 970, 821, 765 cm^{-1}; **HRMS** (DART) calcd. for $C_{19}H_{30}BBrNSi$ [$M+H^+$]: 390.1419, found 390.1430.

Synthesis of C6-Bpin-*N*-H-1,2-azaborine

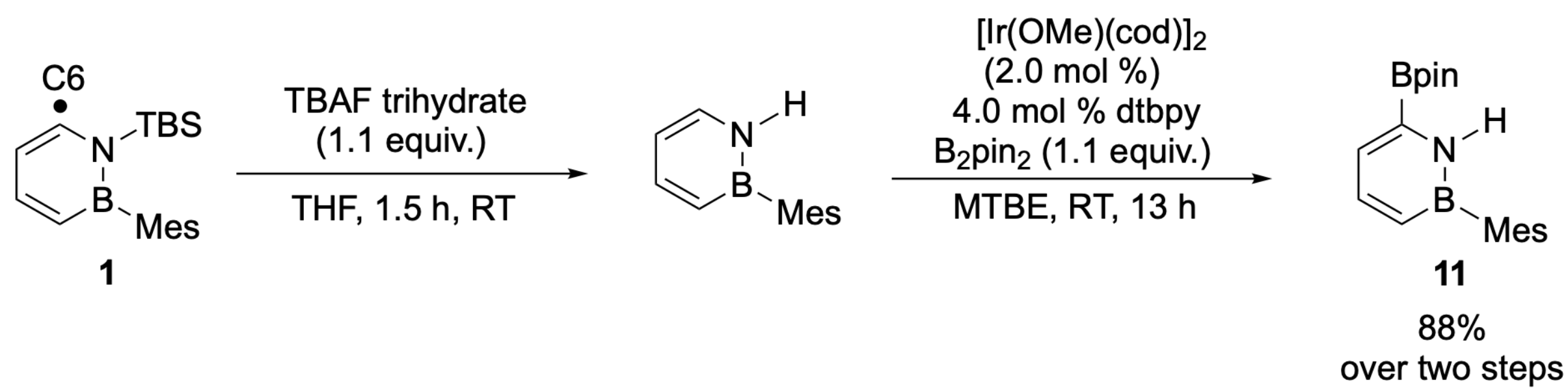

Step 1:

In a glove box, a 100 mL round bottom flask was charged with a magnetic stir bar, azaborine **1** (8.00 g, 25.7 mmol, 1.00 equiv.) and THF (50 mL). TBAF trihydrate (8.92 g, 28.3 mmol, 1.1 equiv) was added to the stirred solution at room temperature, and the reaction mixture was allowed to stir for 1.5 hours. At the conclusion of the reaction, the reaction mixture was concentrated *in vacuo* and purified by silica gel chromatography using gradient of 0–10% Et_2O in hexane to obtain the product as colorless oil (4.90 g, 24.9 mmol, 97%).

***N*-H-1,2-azaborine**

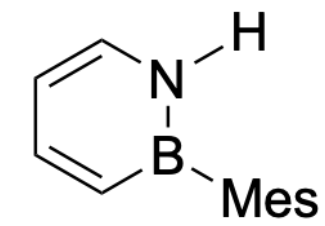

^{1}H NMR ($CDCl_3$, 500 MHz) δ 7.89 (br s, 1H), 7.73 (dd, *J* = 11.2, 6.5 Hz, 1H), 7.40 (t, *J* = 7.1 Hz, 1H), 6.92–6.84 (m, 3zH), 6.38 (t, *J* = 6.6 Hz, 1H), 2.31 (s, 3H), 2.17 (s, 6H); **^{13}C NMR** ($CDCl_3$, 126 MHz) δ 143.9, 140.4, 137.4, 133.9, 127.3, 110.6, 23.2, 21.3. (the signals for the two carbons adjacent to boron were not observed); **^{11}B NMR** ($CDCl_3$, 160 MHz) δ 36.1.

Step 2:

Inside a glove box, a 500 mL round bottle flask was charged with a magnetic stir bar, $[IrCOD(OMe)]_2$ (161 mg, 0.243 mmol, 2.0 mol%), 4,4'-di-*tert*-butyl-2,2'-bipyridine (dtbbpy) (130 mg, 0.485 mmol, 4.0 mol%), bis(pinacolato)diboron (B_2pin_2) (3.39 g, 13.3 mmol, 1.10 equiv.) and 30 mL methyl *tert*-butyl ether. The catalyst mixture was stirred for 15 minutes during which it turned the color from light yellow to dark brown. *N*-H-1,2-azaborine (2.39 g, 12.1 mmol, 1.00 equiv.) was dissolved in 10 ml methyl *tert*-butyl ether and added to the round bottom flask. The reaction mixture was allowed to stir at room temperature for 13 hours. At the conclusion of the reaction, the reaction mixture was concentrated *in vacuo* and purified by silica gel chromatography using 10% Et_2O in hexane to obtain the product **11** as white solid (3.58 g, 11.1 mmol, 91%).

C6-Bpin-*N*-H-1,2-azaborine 11

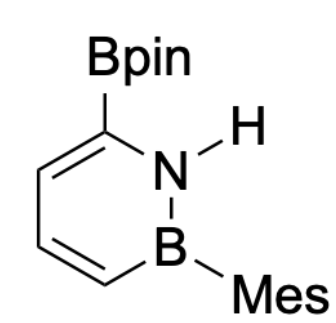

^{1}H NMR ($CDCl_3$, 500 MHz) δ 8.39 (br s, 1H), 7.72 (dd, *J* = 11.1, 6.5 Hz, 1H), 7.05 (d, *J* = 11.1 Hz, 1H), 6.96 (d, *J* = 6.5 Hz, 1H), 6.88 (s, 2H), 2.31 (s, 3H), 2.17 (s, 6H), 1.33 (s, 9H); **^{13}C NMR** ($CDCl_3$, 126 MHz) δ 142.6, 140.4, 137.2, 127.2, 119.8, 84.8, 25.0, 23.3, 21.3. (the signals for the three carbons adjacent to boron were not observed); **^{11}B NMR** ($CDCl_3$, 160 MHz) δ 35.8, 29.3.

General Procedure for C3-Substituted Compounds **13**, **14**, and **15** via Negishi Cross-coupling

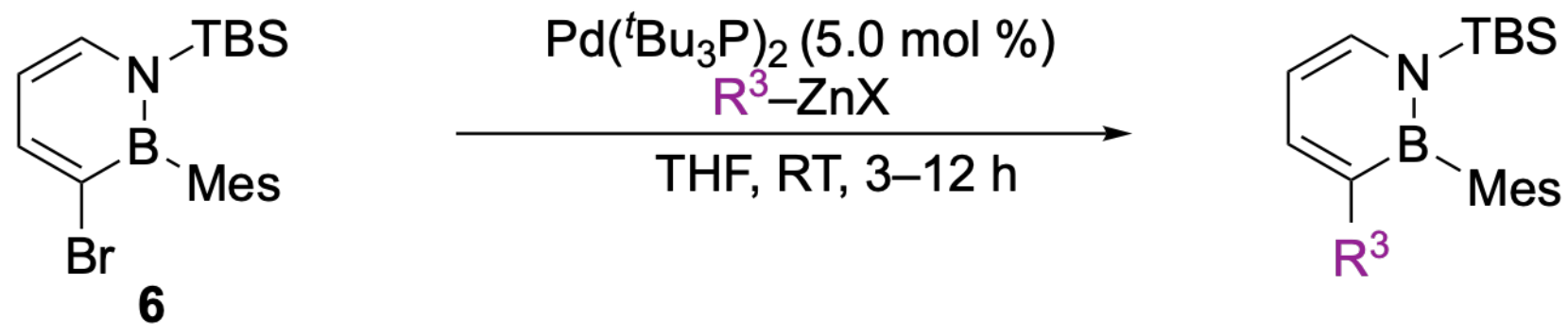

In adaptation of a previously reported Negishi Cross-coupling method:[5]

The synthesis of C3-Me azaborine **13** is representative. In a glovebox, a 250 ml round bottom flask was charged with a magnetic stir bar, C3-Br-azaborine **6** (3.00 g, 7.69 mmol, 1.00 equiv.), Pd(P^{t}Bu$_3$)2 (117 mg, 0.230 mmol, 3.0 mol%), and 25 mL THF. The resulting mixture was allowed to stir for 5 minutes before adding dimethylzinc solution (9.6 mL, 11.5 mmol, 1.2 M in toluene, 1.5 equiv.). The reaction mixture was allowed to stir at room temperature for 3 hours. At the conclusion of the reaction, 10 mL dichloromethane was added to quench the reaction. The solid was filtered off and the filtrate was concentrated in *vacuo*. The crude residue was then purified by silica gel chromatography using 5% dichloromethane in hexane to obtain the product **13** as colorless oil (2.11g, 6.48 mmol, 84%).

C3-methyl-1,2-azaborine 13

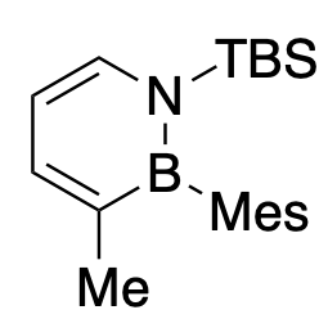

^{1}H NMR ($CDCl_3$, 500 MHz) δ 7.33 (d, J = 6.8 Hz, 1H), 7.30 (d, J = 6.8 Hz, 1H), 6.80 (s, 2H), 6.31 (t, J = 6.8 Hz, 1H), 2.31 (s, 3H), 2.05 (s, 6H), 1.81 (s, 3H), 0.94 (s, 9H), –0.02 (s, 6H). **^{13}C NMR** ($CDCl_3$, 126 MHz) δ 140.2, 138.6, 136.3, 135.8, 127.0, 111.2, 27.8, 22.9, 21.7, 21.4, 19.4, –3.0 (the signals for the two carbons adjacent to boron were not observed); **^{11}B NMR** ($CDCl_3$, 160 MHz) δ 40.5; **IR**; 2961, 2931, 2859, 1614, 1525, 1473, 1351, 1264, 1229, 1051, 970, 820, 809, 785, 761 cm^{-1}; **HRMS** (DART) calcd. for $C_{20}H_{33}BNSi$ [M+H$^+$]: 326.2470, found 326.2481

C3-propyl-1,2-azaborine 14

The desired product was obtained by column chromatography (silica gel, 0–5% diethyl ether in hexane) as colorless oil with a yield of 67%.

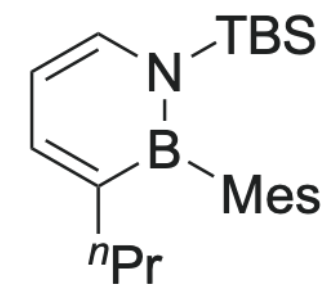

^{1}H NMR ($CDCl_3$, 500 MHz) δ 7.34 (d, *J* = 6.6 Hz, 1H), 7.31 (d, *J* = 6.9 Hz, 1H), 6.77 (s, 2H), 6.35 (t, *J* = 6.7 Hz, 1H), 2.30 (s, 3H), 2.11–2.06 (m, 2H), 2.05 (s, 6H), 1.37–1.25 (m, 2H), 0.93 (s, 9H), 0.77 (t, *J* = 7.3 Hz, 3H), –0.04 (s, 6H); **^{13}C NMR** ($CDCl_3$, 126 MHz) δ 139.5, 138.9, 136.2, 135.9, 126.9, 111.2, 36.9, 27.8, 23.3, 23.2, 21.4, 19.5, 14.3, –2.9 (the signals for the two carbons adjacent to boron were not observed); **^{11}B NMR** ($CDCl_3$, 160 MHz) δ 40.2; **IR** 3067, 2959, 2930, 2859, 1610, 1523, 1472, 1391, 1322, 1264, 1228, 1196, 1165, 1110,1030, 1008, 976, 892, 837, 821, 766, 739 cm^{-1}; **HRMS** (DART) calcd. for $C_{22}H_{37}BNSi$ [M+H^+]: 354.2783, found 354.2779.

C3-benzyl-1,2-azaborine 15

The desired product was obtained by column chromatography (silica gel, 0–5% diethyl ether in hexane) as a colorless oil with a yield of 80%.

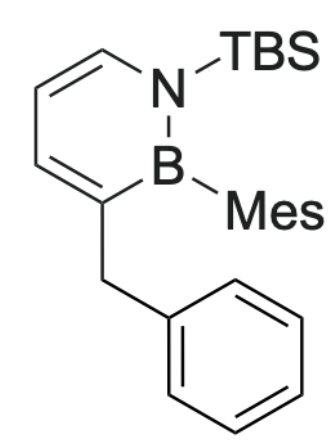

^{1}H NMR ($CDCl_3$, 500 MHz) δ 7.32 (dd, *J* = 6.9, 1.2 Hz, 1H), 7.22 (dd, *J* = 6.5, 1.3 Hz, 1H), 7.17–7.06 (m, 3H), 6.88–6.81 (m, 2H), 6.75 (s, 2H), 6.33 (t, *J* = 6.7 Hz, 1H), 3.41 (s, 2H), 2.31 (s, 3H), 1.91 (s, 6H), 0.93 (s, 9H), –0.03 (s, 6H); **^{13}C NMR** ($CDCl_3$, 126 MHz) δ 142.3, 140.6, 139.0, 136.4, 136.4, 129.5, 127.9, 127.0, 125.3, 111.2, 41.4, 27.8, 23.0, 21.4, 19.5, –2.9 (the signals for the two carbons adjacent to boron were not observed); **^{11}B NMR** ($CDCl_3$, 160 MHz) δ 40.7; **IR** 3061, 2985, 2926, 2859, 1610, 1527, 1472, 1371, 1289, 1228, 1196, 1165, 1093, 976, 811, 766, 728 cm^{-1}; **HRMS** (DART) calcd. for $C_{26}H_{37}BNSi$ [M+H^+]: 402.2783, found 402.2791.

Synthesis of C3-*p*-OMe-Ph-1,2-azaborine **17**

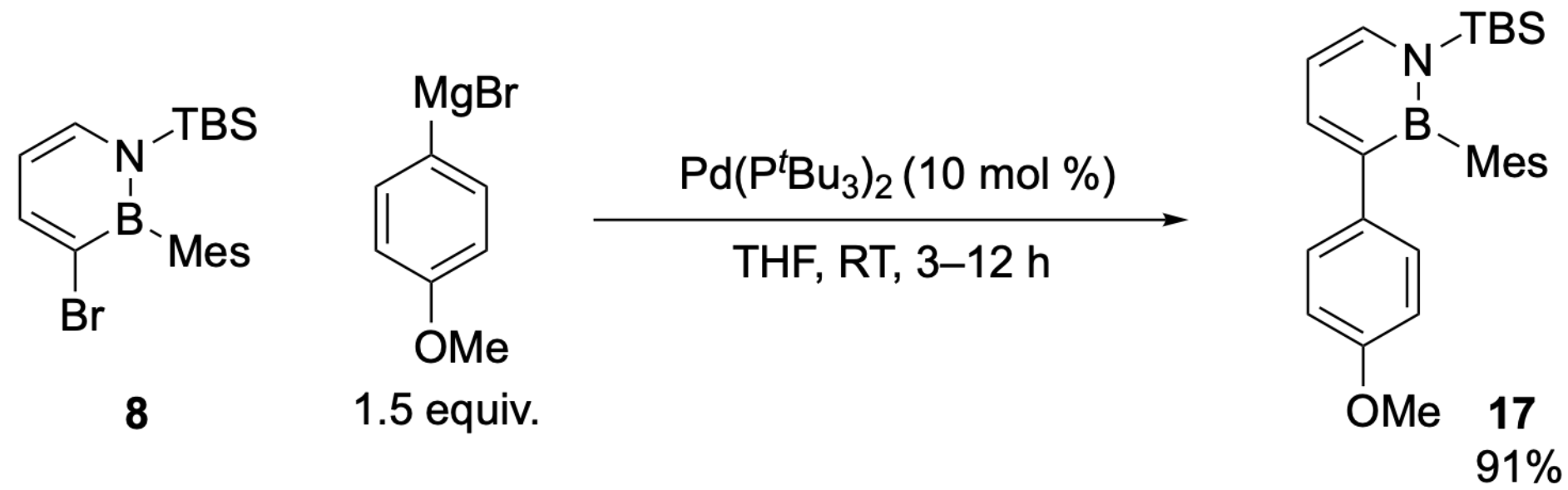

In a glove box, a 20 mL vial was charged with a magnetic stir bar, C3-Br azaborine **8** (300 mg, 7.69 mmol, 1.00 equiv.), Pd(P^{t}Bu$_3$)$_2$ (39 mg, 0.077 mmol, 10 mol%), and 6 mL toluene. (4-methoxyphenyl)magnesium bromide (2.3 mL, 1.15 mmol, 1.5 equiv., 0.50 M in THF) was then added. The resulting mixture was then allowed to stir at room temperature for 40 hours. At the conclusion of the reaction, the reaction mixture was quenched with H_2O, extracted 3 times with ethyl acetate, dried over $MgSO_4$, and concentrated *in vacuo*. The crude mixture was then purified by silica gel chromatography using 10% dichloromethane in hexane to obtain the product **16** as white solid (292 mg, 0.699 mmol, 91%).

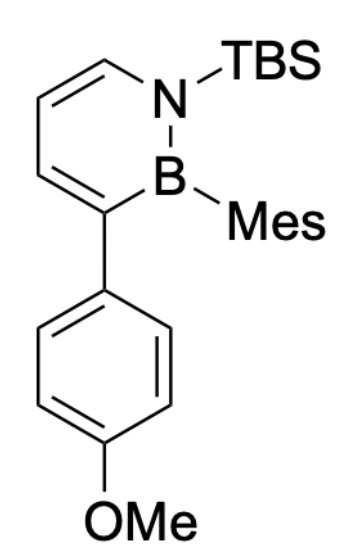

^{1}H NMR ($CDCl_3$, 500 MHz) δ 7.49 (d, J = 6.7 Hz, 1H), 7.44 (d, J = 6.7 Hz, 1H), 6.84 (d, J = 8.7 Hz, 2H), 6.66 (s, 2H), 6.62 (d, J = 8.7 Hz, 2H), 6.48 (t, J = 6.8 Hz, 1H), 3.72 (s, 3H), 2.24 (s, 3H), 1.95 (s, 6H), 0.91 (s, 9H), 0.00 (s, 6H); **^{13}C NMR** ($CDCl_3$, 126 MHz) δ 157.5, 141.0, 139.2, 138.4, 137.5, 136.5, 129.1, 127.0, 113.0, 111.5, 55.2, 27.7, 23.2, 21.4, 19.5, –2.9. (the signals for the two carbons adjacent to boron were not observed); **^{11}B NMR** ($CDCl_3$, 160 MHz) δ 40.0; **IR** 2958, 2930, 2857, 1604, 1519, 1506, 1338, 1285, 1261, 1240, 1175, 1092, 1039, 1013, 998, 969, 822, 806, 784, 750 cm^{-1}; **HRMS** (DART) calcd. for $C_{26}H_{37}BNOSi$ [M+H$^+$]: 418.2732, found 418.2742.

Synthesis of C4-Substituted Compounds **18** and **33** via Suzuki Cross-coupling

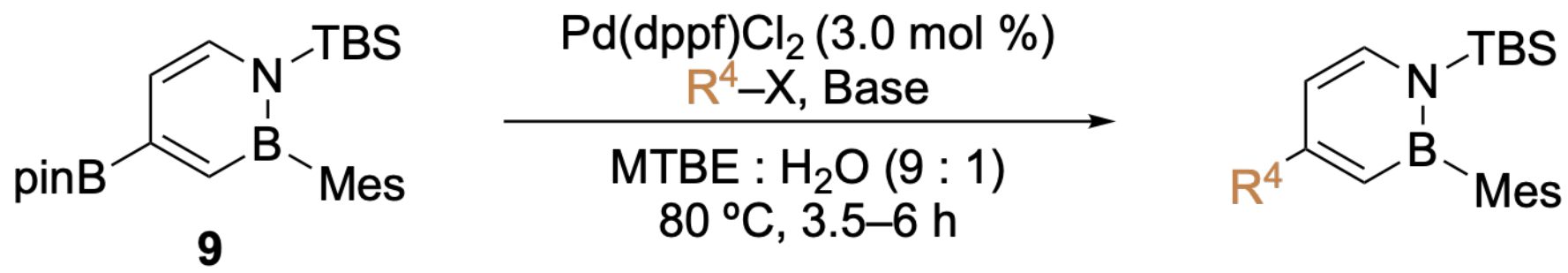

C4-methyl-1,2-azaborine 18

Inside a drybox, a 15 mL pressure vessel was charged with a magnetic stir bar, C4-Bpin-azaborine **9** (87.5 mg, 0.200 mmol, 1.0 equiv.), methyl iodide (75 µL, 1.2 mmol, 6.0 equiv.), Pd(dppf)Cl_2 (4.9 mg, 6.0 µmol, 3.0 mol%), and 3.6 mL methyl *tert*-butyl ether. $K_3PO_4 \cdot H_2O$ (4.0 equiv., 184.2 mg, 0.800 mmol) was dissolved in 0.40 mL H_2O and was added into the pressure vessel. The reaction mixture was sealed and allowed to stir at 80 °C in an oil bath for 6 hours. At the conclusion of the reaction, 3 mL of water was added into the reaction mixture and the aqueous phase was extracted 3 x 3 mL with diethyl ether. The combined organic extracts were washed with brine, dried over $MgSO_4$, vacuum-filtered through a fritted funnel, and concentrated *in vacuo*. The crude residue was purified by flash column chromatography using 100% hexane to give the desired product **18** as white solid (50.5 mg, 0.155 mmol, 78%).

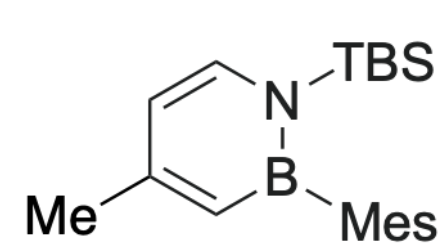

^{1}H NMR ($CDCl_3$, 500 MHz) δ 7.36 (d, J = 6.9 Hz, 1H), 6.80 (s, 2H), 6.37 (d, J = 2.1 Hz, 1H), 6.24 (dd, J = 6.9, 2.1 Hz, 1H), 2.31 (s, 3H), 2.25 (s, 3H), 2.11 (s, 6H), 0.94 (s, 9H), 0.01 (s, 6H); **^{13}C NMR** ($CDCl_3$, 126 MHz) δ 153.0, 138.9, 138.3, 136.3, 126.9, 114.0, 27.6, 24.5, 23.6, 21.3, 19.4, –3.2 (the signals for the two carbons adjacent to boron were not observed); **^{11}B NMR** ($CDCl_3$, 160 MHz) δ 39.4; **IR** 2929, 2858, 1625, 1504, 1436, 1310, 1264, 1187, 1125, 840, 829, 801 cm^{-1}; **HRMS** (DART) calcd. for $C_{20}H_{33}BNSi$ [$M+H^+$]: 326.2470, found 326.2468.

C4-*p*-Tolyl-1,2-azaborine 33

Inside a drybox, a 15 mL pressure vessel was charged with a magnetic stir bar, C4-Bpin-1,2-azaborine **7** (150 mg, 0.343 mmol, 1.0 equiv.), 4-bromotoluene (58.7 mg, 0.343 mmol, 1.0 equiv), Pd(dppf)Cl_2 (8.4 mg, 10 μmol, 3.0 mol %). KOH (77.0 mg, 1.37 mmol, 4.0 equiv.), and 3.6 mL methyl *tert*-butyl ether. 0.40 mL H_2O was added into the pressure vessel and then the reaction mixture was sealed and allowed to stir at 80 °C in an oil bath for 3.5 hours. After cooling, 3 mL of water was added into the reaction mixture and the aqueous phase was extracted 3 x 3 mL with diethyl ether; the combined organic extracts were washed with brine and dried over $MgSO_4$, vacuum-filtered through a fritted funnel, and concentrated *in vacuo*. The crude residue was purified by flash column chromatography using 0–5% diethyl ether in hexane to give the desired product **38** as colorless solid (120.0 mg, 0.299 mmol, 87%)

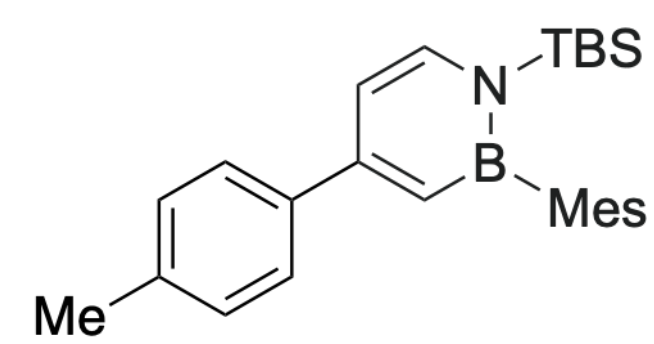

^{1}H NMR ($CDCl_3$, 500 MHz) δ 7.60 (d, *J* = 8.3 Hz, 2H), 7.55 (d, *J* = 7.2 Hz, 1H), 7.24 (d, *J* = 7.9 Hz, 2H), 6.89 (d, *J* = 2.4 Hz, 1H), 6.84 (s, 2H), 6.72 (dd, *J* = 7.1, 2.4, Hz, 1H), 2.40 (s, 3H), 2.34 (s, 3H), 2.16 (s, 6H), 0.98 (s, 9H), 0.07 (s, 6H); **^{13}C NMR** ($CDCl_3$, 126 MHz) δ 153.2, 139.3, 139.1, 139.0, 138.0, 136.5, 129.4, 127.0, 126.8, 110.8, 27.6, 23.6, 21.4, 21.3, 19.4, –3.2 (the signals for the two carbons adjacent to boron were not observed); **^{11}B NMR** ($CDCl_3$, 160 MHz) δ 40.2; **IR** 2960, 2928, 2857, 1615, 1494, 1446, 1264, 1000, 840, 823, 804, 787, 734, 705 cm^{-1}; **HRMS** (DART) calcd. for $C_{26}H_{37}BNSi$ [$M+H^+$]: 402.2783, found 402.2781.

<u>General Procedures for C5-Substituted Compounds **20**–**23** via Suzuki Cross-coupling</u>

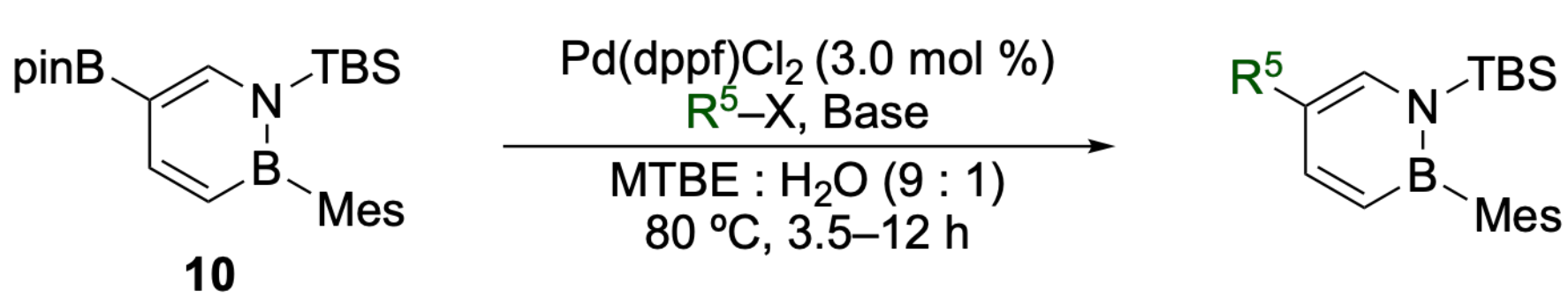

The synthesis of C5-Me-1.2-azaborine **20** is representative. Inside a drybox, a 15 mL pressure vessel was charged with a magnetic stir bar, C5-Bpin-1,2-azaborine **10** (150 mg, 0.343 mmol, 1.0 equiv.), methyl iodide (219 mg, 1.54 mmol, 4.5 equiv.), Pd(dppf)Cl_2 (3.0 mol %, 8.4 mg, 10.3 μmol), $K_3PO_4 \cdot H_2O$ (316.0 mg, 1.37 mmol, 4.0 equiv.), and 4.5 mL MTBE. 0.5 mL H_2O was added into the pressure vessel. The reaction mixture was sealed and allowed to stir at 80 °C in an oil bath for 6 hours. At the conclusion of the reaction, 5 mL of water was added into the reaction mixture and the aqueous phase was extracted 3 x 5 mL with diethyl ether. The combined organic extracts were washed with brine, dried over $MgSO_4$, vacuum-filtered through a fritted funnel, and concentrated *in vacuo*. The crude residue was purified by flash column chromatography with 0–2% of diethyl ether in hexane to give the desired product **20** as colorless oil (101 mg, 0.310 mmol, 90%).

C5-Me-1,2-azaborine 20

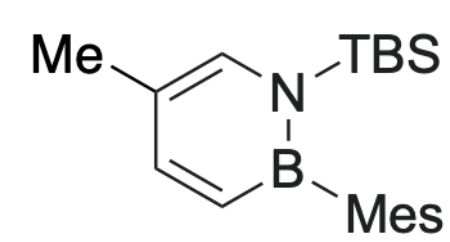

^{1}H NMR ($CDCl_3$, 500 MHz) δ 7.47 (d, J = 11.0 Hz, 1H), 7.22 (s, 1H), 6.79 (s, 2H), 6.59 (d, J = 11.0 Hz, 1H), 2.30 (s, 3H), 2.17 (s, 3H), 2.09 (s, 6H), 0.92 (s, 9H), 0.00 (s, 6H); **^{13}C NMR** ($CDCl_3$, 126 MHz) δ 145.8, 139.0, 136.4, 135.9, 127.0, 119.0, 27.6, 23.6, 21.3, 19.3, 19.2, –3.1 (the signals for the two carbons adjacent to boron were not observed); **^{11}B NMR** ($CDCl_3$, 160 MHz) δ 38.3; **IR** 2960, 2928, 2859, 1625, 1504, 1438, 1370, 1264, 1188, 1126, 829, 801, 783 cm^{-1}; **HRMS** (DART) calcd. for $C_{20}H_{33}BNSi$ [$M+H^+$]: 326.2470, found 326.2479.

C5-ethyl-1,2-azaborine 21. Step 1: According to general procedure (4.0 equiv. vinyl–Br and 4.0 equiv. KOH was used), the C5-vinyl precursor was purified by flash column chromatography using 5% dichloromethane in hexane to give colorless oil.

Step 2: To a 20 ml vial with a stir bar, C5-vinyl-*N*–TBS-*B*–Mes azaborine was added together with Pd/C (5 mol % Pd). The vial was vacuumed and recharged with H_2 balloon before 2.0 mL of dichloromethane was added as solvent. The mixture was allowed to be stirred at room temperature for 2 hours. The crude mixture was then filtered and concentrated *in vacuo*. The crude residue was purified by flash column chromatography using 100% hexane to afford the desired product as a white solid (average yield over two steps was 72%).

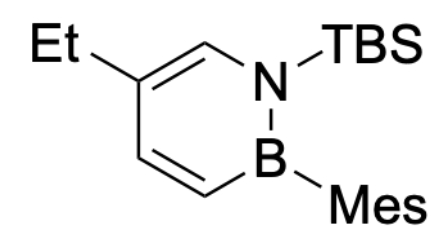

^{1}H NMR ($CDCl_3$, 600 MHz) δ 7.51 (dd, *J* = 11.0, 1.7 Hz, 1H), 7.23 (d, *J* = 1.7 Hz, 1H), 6.80 (s, 2H), 6.61 (d, *J* = 11.0 Hz, 1H), 2.49 (q, *J* = 7.5 Hz, 2H), 2.30 (s, 3H), 2.10 (s, 6H), 1.22 (t, *J* = 7.5 Hz, 3H), 0.93 (s, 9H), 0.01 (s, 6H); **^{13}C NMR** ($CDCl_3$, 151 MHz) δ 144.9, 139.1, 136.4, 135.3, 127.0, 125.4, 27.7, 27.0, 23.6, 21.3, 19.4, 15.8, –3.1 (the signals for the two carbons adjacent to boron were not observed); **^{11}B NMR** ($CDCl_3$, 160 MHz) δ 39.4; **IR** 2961, 2930, 2853, 1622, 1609, 1502, 1472, 1362, 1264, 1008, 839, 825, 799 cm^{-1}; **HRMS** (DART) calcd. for $C_{21}H_{35}BNSi$ [M+H$^+$]: 340.2626, found 340.2629.

C5-CH$_2$-ester-1,2-azaborine 22

Slight modification of the procedure reported by Gooßen[6]: Inside a drybox, a 15 mL pressure vessel was charged with a magnetic stir bar, C5-Bpin-1,2-azaborine **10** (150.0 mg, 0.343 mmol, 1.0 equiv.), methyl-2-bromoacetate (52.5 mg, 0.343 mmol, 1.0 equiv.), $Pd(OAc)_2$ (2.3 mg, 10 µmol, 3.0 mol%) and tri(*o*-tolyl)phosphine (9.4 mg, 31 µmol, 9.0 mol%), $K_3PO_4 \cdot H_2O$ (316 mg, 1.37 mmol, 4.0 equiv.), and 3.0 mL THF. 0.5 mL H_2O was added into the pressure vessel. The reaction mixture was sealed and allowed to stir at 60 °C in an oil bath for 16 hours. At the conclusion of the reaction, 4 mL water was added into the reaction mixture and the aqueous phase was extracted 3 x 4 mL with ethyl acetate; the combined organic extracts were washed with brine, dried over $MgSO_4$, vacuum-filtered through a fritted funnel, and concentrated *in vacuo*. The crude

residue was purified by flash column chromatography using 5% diethyl ether in hexane to give the desired product **22** as colorless oil with an average yield of 61%. (first batch: 85.5 mg, 0.223 mmol, 65%; second batch: 75.0 mg, 0.195 mmol, 57%).

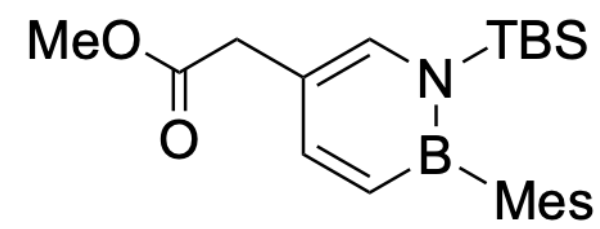

^{1}H NMR ($CDCl_3$, 500 MHz) δ 7.54 (d, *J* = 11.1 Hz, 1H), 7.38 (s, 1H), 6.81 (s, 2H), 6.66 (d, *J* = 11.1 Hz, 1H), 3.73 (s, 3H), 3.47 (s, 2H), 2.31 (s, 3H), 2.11 (s, 6H), 0.94 (s, 9H), 0.03 (s, 6H). **^{13}C NMR** ($CDCl_3$, 126 MHz) δ 172.7, 144.8, 139.0, 137.8, 136.6, 127.0, 116.5, 52.0, 39.2, 27.5, 23.5, 21.3, 19.2, –3.2 (the signals for the two carbons adjacent to boron were not observed); **^{11}B NMR** ($CDCl_3$, 160 MHz) δ 39.6; **IR** 2955, 2931, 2859, 1741, 1623, 1502, 1449, 1379, 1265, 1187, 1009, 934, 847, 836, 785 cm^{-1}; **HRMS** (DART) calcd. for $C_{22}H_{35}BNO_2Si$ [M+H$^+$]: 384.2586, found 384.2586.

C5-*p*-F-Bn-1,2-azaborine 23

According to general procedure (1.0 equiv. *p*-F-Bn–Br was used), the product was purified by flash column chromatography using 100% hexane to give the desired product **22** as white solid with an average yield of 82% (first batch: 116.3 mg, 0.276 mmol, 81%; second batch:119.2 mg, 0.284 mmol, 83%).

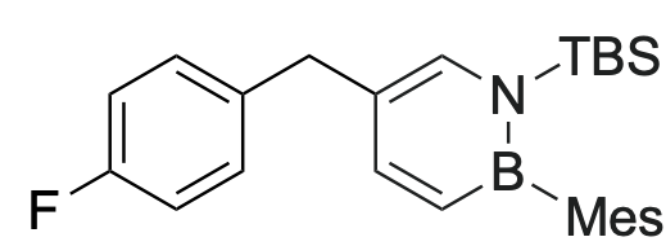

^{1}H NMR ($CDCl_3$, 600 MHz) δ 7.44 (dd, *J* = 11.1, 1.8 Hz, 1H), 7.17 (dd, *J* = 8.4, 5.6 Hz, 2H), 7.12 (d, *J* = 1.7 Hz, 1H), 7.01 (t, *J* = 8.7 Hz, 2H), 6.79 (s, 2H), 6.62 (d, *J* = 11.1 Hz, 1H), 3.79 (s, 2H), 2.30 (s, 3H), 2.09 (s, 6H), 0.85 (s, 9H), –0.03 (s, 6H); **^{13}C NMR** ($CDCl_3$, 151 MHz) δ 161.58 (d, $^1J_{CF}$ = 243.8 Hz), 145.0, 139.0, 136.98(d, $^4J_{CF}$ = 3.0 Hz), 136.9, 136.5, 130.43(d, $^3J_{CF}$ = 8.0 Hz), 127.0, 122.8, 115.3 (d, $^2J_{CF}$ = 20.8 Hz) , 39.1, 27.6, 23.6, 21.3, 19.2, –3.2 (the signals for the two carbons adjacent to boron were not observed); **^{11}B NMR** ($CDCl_3$, 160 MHz) δ 39.6; **^{19}F NMR** ($CDCl_3$, 470 MHz) δ –117.5 (m); **IR**

2959, 2929, 2858, 1622, 1507, 1448, 1264, 1223, 1123, 849, 836, 805, 784 cm^{-1}; **HRMS** (DART) calcd. for $C_{26}H_{36}BNFSi$ [$M+H^+$]: 420.2689, found 420.2694.

General Procedures of C6-Substituted Compounds **24**, **25**, and **34** via Suzuki Cross-coupling

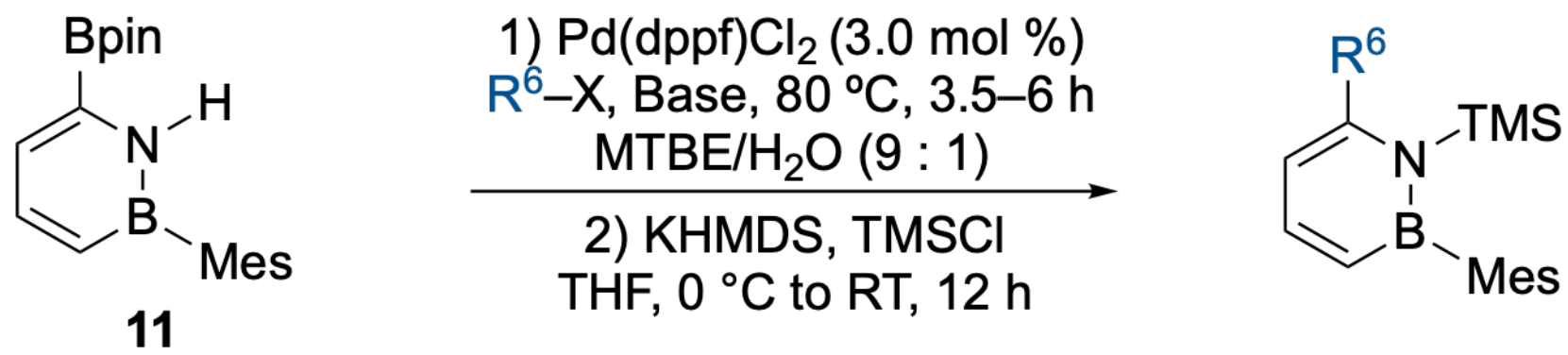

The synthesis of C6-Me 1,2-azaborine **24** is represetative.

Step 1: Inside a drybox, a 15 mL pressure vessel was charged with a magnetic stir bar, C6-Bpin-*N*-H-1,2-azaborine **11** (590 mg, 1.83 mmol, 1.0 equiv.), methyl iodide (1.17 g, 8.22 mmol ,4.5 equiv.), $Pd(dppf)Cl_2$ (44.7 mg, 54.8 µmol, 3.0 mol%). $K_3PO_4 \cdot H_2O$ (1.26 g, 5,48 mmol, 3.0 equiv.), and 4.5 mL of MTBE. 0.5 mL H_2O was added into the pressure vessel and the reaction was sealed and allowed to stir at 80 °C in an oil bath for 6 hours. At the conclusion of the reaction, 10 mL of water was added into the reaction mixture and the aqueous phase was extracted 3 x 10 mL with diethyl ether; the combined organic extracts were washed with brine, dried over $MgSO_4$, vacuum-filtered through a fritted funnel, and concentrated *in vacuo*. The crude residue was purified by flash column chromatography to give the precursor.

Step 2: A 20 mL vial was charged with the C6-Me N–H precursor and 6.0 mL THF. The solution was cooled down to 0 °C before KHMDS (3.5 mL, 1.0 M in THF, 2.0 equiv.) was added via syringe dropwise with N_2 flow. The reaction mixture was warmed up to room temperature and allowed to stir for additional 30 minutes before TMSCl (564 mg, 5.19 mmol, 10.0 equiv.) was added in one portion. The reaction mixture was allowed to stir at room temperature for 12 hours. At the conclusion of the reaction, the reaction mixture was concentrated *in vacuo* and purified by

flash column chromatography using 100 % hexane to give the desired product **29** (359 mg, 1.27 mmol, 69%).

C6-Me-1,2-azaborine 24

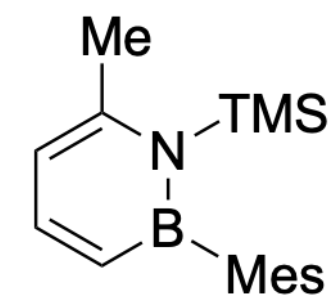

^{1}H NMR ($CDCl_3$, 500 MHz) δ 7.45 (dd, *J* = 10.9, 6.5 Hz, 1H), 6.78 (s, 2H), 6.41 (d, *J* = 10.9 Hz, 1H), 6.26 (d, *J* = 6.5 Hz, 1H), 2.55 (s, 3H), 2.29 (s, 3H), 2.10 (s, 6H), 0.17 (s, 9H); **^{13}C NMR** ($CDCl_3$, 126 MHz) δ 146.8, 143.4, 138.6, 136.3, 127.0, 114.8, 25.3, 23.5, 21.3, 4.8 (the signals for the two carbons adjacent to boron were not observed); **^{11}B NMR** ($CDCl_3$, 160 MHz) δ 40.6; **IR** 2974, 2915, 2853, 1601, 1520, 1446, 1400, 1258, 1155, 1088, 845, 802, 764 cm^{-1}; **HRMS** (DART) calcd. for $C_{17}H_{27}BNSi$ [M+H$^+$]: 284.2000, found 284.2000.

C6-ethyl-1,2-azaborine 25

According to general procedure (For step1: 5.0 equiv. vinyl–Br was used as electrophile; 4.0 equiv. of KOH was used as base; reaction took 3.5 hours), after hydrogenation followed by TMS protection, the crude was purified by flash column chromatography using 100% hexane to give the desired product **30** as white solid with an average yield of 68% over three steps.

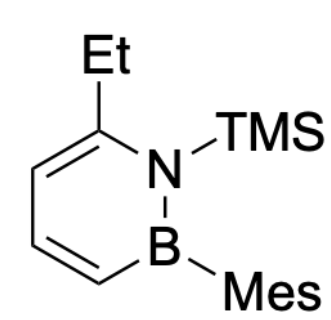

^{1}H NMR ($CDCl_3$, 500 MHz) δ 7.52 (dd, *J* = 10.8, 6.6 Hz, 1H), 6.80 (s, 2H), 6.42 (d, *J* = 10.8 Hz, 1H), 6.33 (d, *J* = 6.6 Hz, 1H), 2.87 (q, *J* = 7.5 Hz, 2H), 2.30 (s, 3H), 2.12 (s, 6H), 1.29 (t, *J* = 7.5 Hz , 3H), 0.15 (s, 9H); **^{13}C NMR** ($CDCl_3$, 126 MHz) δ 153.5, 143.4, 138.7, 136.4, 127.1, 112.6, 30.0, 23.5, 21.3, 16.1, 4.7 (the signals for the two carbons adjacent to boron were not observed); **^{11}B NMR** ($CDCl_3$, 160 MHz) δ 40.5; **IR** 2959, 2923, 2851, 1603, 1520, 1455, 1402, 1371, 1258, 1092, 1048, 846, 801, 770 cm^{-1}; **HRMS** (DART) calcd. for $C_{18}H_{29}BNSi$ [M+H$^+$]: 298.2157, found 298.2172.

C6-*p*-tolyl-1,2-azaborine 34

According to general procedure (For step 1: 1.0 equiv. Tolyl–Br was used as electrophile; 4.0 equiv. of KOH was used as base; 3.5 hours), after TMS protection, the crude was purified by flash column chromatography using 100% hexane to give the desired product **40** as white solid with an average yield of 70% over two steps.

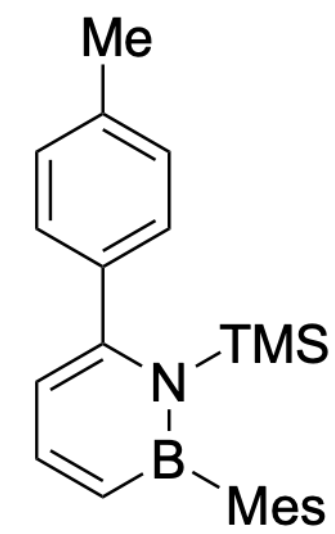

^{1}H NMR ($CDCl_3$, 500 MHz) δ 7.56 (dd, *J* = 10.9, 6.5 Hz, 1H), 7.32 (d, *J* = 7.9 Hz, 2H), 7.20 (d, *J* = 7.9 Hz, 2H), 6.85 (s, 2H), 6.63 (d, *J* = 10.9 Hz, 1H), 6.30 (d, *J* = 6.5 Hz, 1H), 2.41 (s, 3H), 2.31 (s, 3H), 2.26 (s, 6H), –0.15 (s, 9H); **^{13}C NMR** ($CDCl_3$, 126 MHz) δ 152.5, 142.8, 140.9, 139.7, 138.0, 137.0, 129.3, 128.7, 127.4, 116.1, 29.9, 24.0, 21.4, 4.6 (the signals for the two carbons adjacent to boron were not observed); **^{11}B NMR** ($CDCl_3$, 160 MHz) δ 42.0; **IR** 2960, 2930, 2859, 1731, 1613, 1445, 1392, 1264, 1155, 1018, 840, 808, 762 cm^{-1}; **HRMS** (DART) calcd. for $C_{23}H_{31}BNSi$ [$M+H^+$]: 360.2277, found 360.2269.

<u>Procedures for C4C6-difunctionalized 1,2-azaborine **26** and **27**</u>

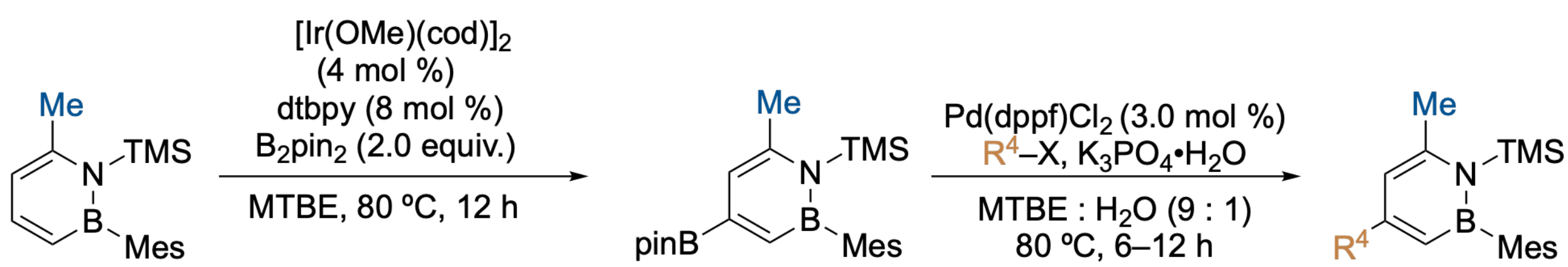

Step 1 (selective C4-borylation): Inside a N_2 glovebox, to a 15 mL pressure vessel containing a magnetic stir bar was charged with $[Ir(OMe)(cod)]_2$ (14 mg, 21 μmol, 4 mol%), 4,4'-*di*-tert-butyl-2,2'-bipyridine (dtbbpy) (11 mg, 44 μmol, 8.0 mol%), bis(pinacolato)diboron (B_2pin_2) (292 mg, 1.09 mmol, 2.0 equiv.) and 4.0 mL MTBE. The catalyst mixture was stirred for 15 minutes during

which it turned color from light yellow to dark brown. Then C6-methyl 1,2-azaborine **24** (154 mg, 0.544 mmol, 1.0 equiv.) was added to the reaction flask. The reaction was allowed to stir at 80°C in an oil bath for 12 hours. At the conclusion of the reaction, the reaction mixture was concentrated *in vacuo* and the crude residue was purified by flash column chromatography using 0–10% of diethyl ether in hexane to give the desired C4-Bpin-C6-methyl-1,2-azaborine precursor as pale yellow oil (185.8 mg, 84%).

C4-benzyl-C6-methyl-1,2-azaborine 26

According to general Suzuki coupling procedure, a 15 mL pressure vessel was charged with a magnetic stir bar, C4-Bpin-C6-Me-1,2-azaborine precursor from step 1 (150 mg, 0.366 mmol, 1.0 equiv.), benzyl bromide (69.0 mg, 0.403 mmol, 1.1 equiv.), Pd(dppf)Cl_2 (9.0 mg, 11 μmol, 3 mol %), $K_3PO_4 \cdot H_2O$ (233 mg, 1.10 mmol, 3.0 equiv.), and 2.5 mL methyl tert-butyl ether. 0.25 mL H_2O was added into the pressure vessel. The reaction mixture was sealed and stirred at 80 °C in an oil bath for 12 hours. At the conclusion of the reaction, water was added into the reaction mixture and the aqueous phase was extracted with diethyl ether. The combined organic extracts were washed with brine, dried over $MgSO_4$, vacuum-filtered through a fritted funnel, and concentrated *in vacuo*. The crude residue was purified by flash column chromatography using 0–2% diethyl ether in hexane to give the desired product **26** as a white solid (91.3 mg, 0.245 mmol, 67%).

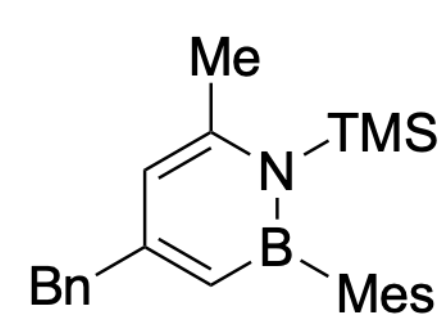

^{1}H NMR ($CDCl_3$, 600 MHz) δ 7.28–7.25 (m, 2H), 7.21–7.17 (m, 3H), 6.77 (s, 2H), 6.18 (s, 1H), 6.08 (s, 1H), 3.75 (s, 2H) 2.48 (s, 3H), 2.29 (s, 3H), 2.09 (s, 6H), 0.14 (s, 9H); **^{13}C NMR** ($CDCl_3$, 126 MHz) δ 156.0, 147.1, 140.5, 138.6, 136.2, 129.3, 128.4, 126.9, 126.1, 116.3, 44.7, 25.2, 23.5, 21.3, 4.7. (the signals for the two carbons adjacent to boron were not observed); **^{11}B NMR** ($CDCl_3$, 160 MHz) δ 40.5; **IR** 3026, 2916, 1611,

1515, 1495, 1453, 1375, 1347, 1257, 1167, 1030, 1013, 967, 846, 769, 714 cm^{-1}; **HRMS** (DART) calcd. for $C_{24}H_{33}BNSi$ [M+H$^+$]: 374.2470, found 374.2463.

C4-*p*-F-benzyl-C6-methyl-1,2-azaborine 27

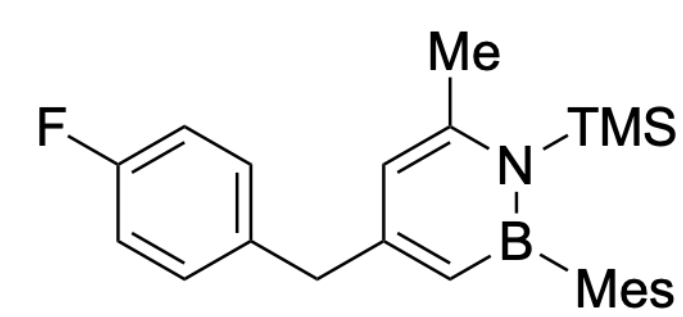

According to general Suzuki coupling procedure, a 15 mL pressure vessel was charged with a magnetic stir bar, C4-Bpin-C6-Me-1,2-azaborine precursor from step 1 (246 mg, 0.602 mmol, 1.0 equiv.), *p*-F-benzyl bromide (125.1 mg, 0.662 mmol, 1.1 equiv.), Pd(dppf)Cl_2 (15 mg, 18 µmol, 3 mol %). $K_3PO_4 \cdot H_2O$ (553 mg, 1.81 mmol, 4.0 equiv.), and 4.5 mL methyl tert-butyl ether. 0.50 mL H_2O was added into the pressure vessel. The reaction mixture was sealed and stirred at 80 °C in an oil bath for 6 hours. At the conclusion of the reaction, water was added into the reaction mixture and the aqueous phase was extracted with diethyl ether. The combined organic extracts were washed with brine, dried over $MgSO_4$, vacuum-filtered through a fritted funnel, and concentrated *in vacuo*. The crude residue was purified by flash column chromatography using 0–2% diethyl ether in hexane to give the desired product **27** as a white solid (194.7 mg, 0.497 mmol, 83%).

^{1}H NMR ($CDCl_3$, 500 MHz) δ 7.14 (dd, J = 8.5, 5.6 Hz, 2H), 6.95 (t, J = 8.7 Hz, 2H), 6.76 (s, 2H), 6.13 (d, J = 2.1 Hz, 1H), 6.04 (d, J = 2.1 Hz, 1H), 3.71 (s, 2H), 2.48 (s, 3H), 2.28 (s, 3H), 2.08 (s, 6H), 0.14 (s, 9H); **^{13}C NMR** ($CDCl_3$, 126 MHz) δ 161.5, ($^1J_{CF}$ = 243.3 Hz), 155.9, 147.2, 138.5, 136.3, 136.1, 130.6, ($^3J_{CF}$ = 7.7 Hz), 127.0, 116.1, 115.18, ($^2J_{CF}$ = 21.1 Hz), 43.7, 25.2, 23.5, 21.3, 4.7 (the signals for the two carbons adjacent to boron were not observed); **^{11}B NMR** ($CDCl_3$, 160 MHz) δ 40.3; **^{19}F NMR** ($CDCl_3$, 470 MHz) δ –117.7 (m); **IR** 2955, 2914, 2854, 1610, 1509, 1346, 1221, 1205, 1172, 1014, 845, 821 cm^{-1}; **HRMS** (DART) calcd. for $C_{24}H_{32}BNFSi$ [M+H$^+$]: 392.2376, found 392.2347.

Procedures for Synthesis of C3,C5-Di-Substituted Compounds **28**–**30**.

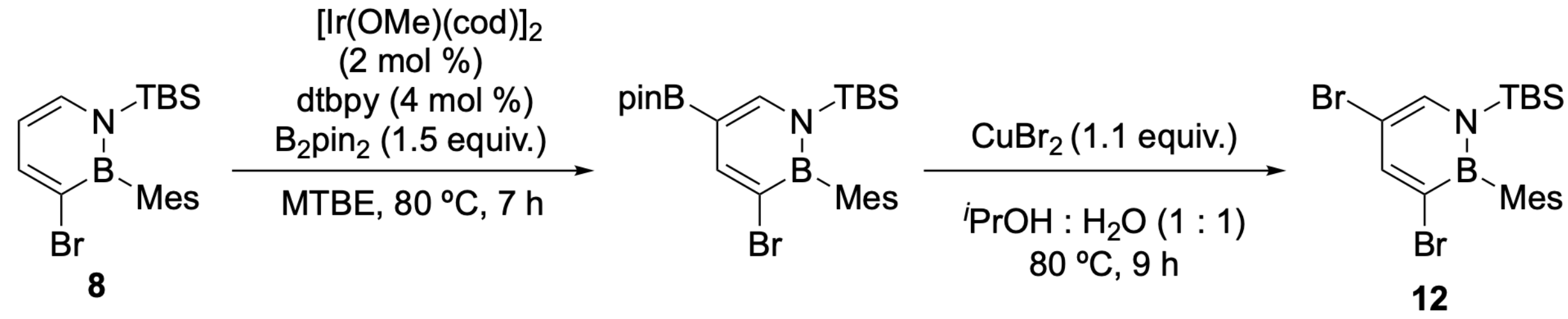

Synthesis of C3C5-bisBr-1,2-azaborine **12**

Step 1: Inside a glovebox, a 15 ml pressure vessel was charged with a magnetic stir bar $[Ir(OMe)(cod)]_2$ (17 mg, 26 μmol, 2 mol%), 4,4'-di-tert-butyl-2,2'-bipyridine (dtbbpy) (14 mg, 51 μmol, 4 mol%), and bis(pinacolato)diboron (B_2pin_2) (488 mg, 1.92 mmol, 1.5 equiv.), and 5.0 mL MTBE. The catalyst mixture was stirred for 15 minutes during which it turned color from light yellow to dark brown. C3-Br 1,2-azaborine **8** (500 mg, 1.28 mmol, 1.0 equiv.) was then added and the resulting mixture was sealed and stirred at 80 ºC in an oil for 7 hours. At the conclusion of the reaction, the reaction mixture was concentrated *in vacuo* and purified by silica gel chromatography using gradient of 0–10% Et_2O in hexane to obtain the C3-Br C5-Bpin 1,2-azaborine as white solid (642 mg, 1.24 mmol, 97%).

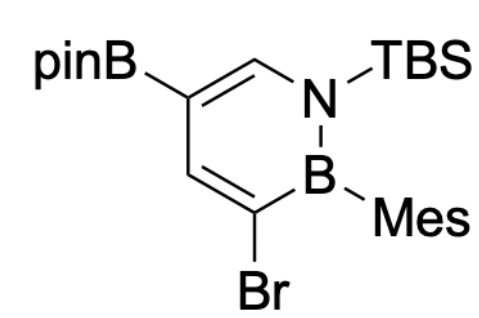

^{1}H NMR ($CDCl_3$, 500 MHz) δ 8.21 (s, 1H), 8.00 (s, 1H), 6.80 (s, 2H), 2.31 (s, 3H), 2.07 (s, 6H), 1.33 (s, 12H), 0.94 (s, 9H), 0.02 (s, 6H); **^{13}C NMR** ($CDCl_3$, 126 MHz) δ 148.1, 147.4, 138.9, 137.2, 127.0, 83.7, 27.5, 25.0, 22.8, 21.5, 19.3, –3.0. (the signals for the three carbons adjacent to boron were not observed); **^{11}B NMR** ($CDCl_3$, 160 MHz) δ 40.0, 30.5.

Step 2: In a glove box, 100 ml round bottom flask was charged with a magnetic stir bar, C3-Br C5-BPin 1,2-azaborine (480 mg, 930 mmol, 1.0 equiv.), and 10 mL isopropanol. Copper(II)

bromide (228 mg, 1.02 mmol, 1.1 equiv.) was dissolved in 10 mL water and the solution was added to the round bottom flask. The flask was sealed and the resulting mixture was stirred at 80 °C in an oil bath for 9 h. At the conclusion of the reaction, the reaction mixture was extracted with ethyl acetate, washed with brine, dried over anhydrous $MgSO_4$, and concentrated *in vacuo*. The crude residue was purified by flash column chromatography using 0–2% diethyl ether in hexane to give the desired product **12** as white solid (340 mg, 0.724 mmol, 78%).

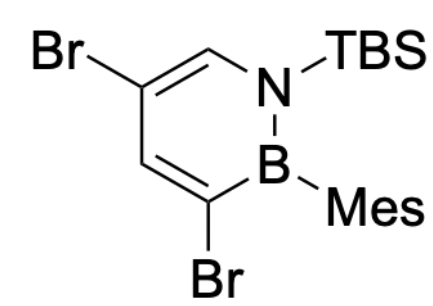

^{1}H NMR ($CDCl_3$, 500 MHz) δ 7.98 (d, J = 1.7 Hz, 1H), 7.57 (d, J = 1.7 Hz, 1H), 6.79 (s, 2H), 2.29 (s, 3H), 2.05 (s, 6H), 0.93 (s, 9H), 0.01 (s, 6H); **^{13}C NMR** ($CDCl_3$, 126 MHz) δ 147.0, 139.0, 137.9, 137.5, 127.2, 104.6, 27.5, 22.9, 21.5, 19.4, –3.1 (the signals for the two carbons adjacent to boron were not observed); **^{11}B NMR** ($CDCl_3$, 160 MHz) δ 39.1; **IR** 2960, 2931, 2860, 1609, 1588, 1472, 1401, 1375, 1314, 1129, 1072, 1006, 975, 872, 839, 820, 790, 751 cm^{-1}; **HRMS** (DART) calcd. for $C_{19}H_{29}BNSiBr_2$ [M+H$^+$]: 468.0524, found 468.0525.

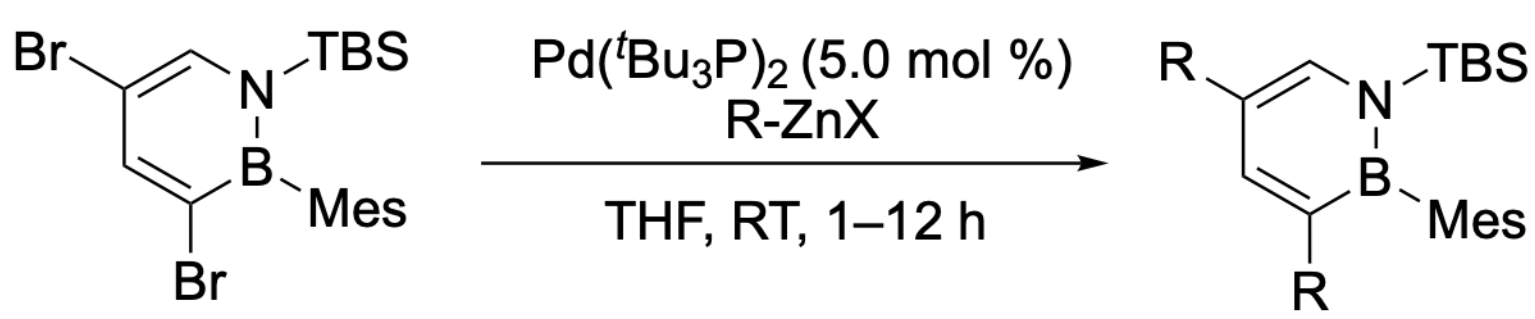

12

C3,C5-*bis*-methyl-1,2-azaborine 28

According to general Negishi coupling procedure (2.5 equiv. Me_2Zn, 1.2 M in toluene was used as organozinc reagent), the crude residue was purified by flash column chromatography using 100% hexane to give the desired product **28** as a colorless oil (98.0 mg, 0.357 mmol, 73%).

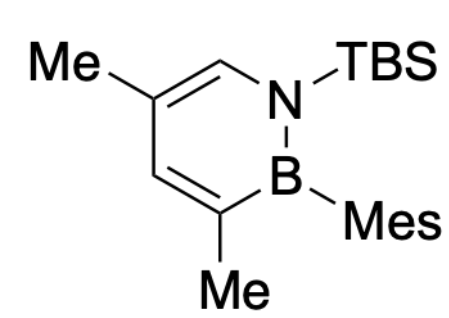

^{1}H NMR ($CDCl_3$, 600 MHz) δ 7.23 (s, 1H), 7.06 (s, 1H), 6.78 (s, 2H), 2.30 (s, 3H), 2.14 (s, 3H), 2.04 (s, 6H), 1.79 (s, 3H), 0.93 (s, 9H), –0.04 (s, 6H). **^{13}C**

NMR ($CDCl_3$, 151 MHz) δ 143.5, 138.7, 136.1, 133.2, 126.9, 118.6, 27.8, 22.9, 21.6, 21.4, 19.4, 19.3, –2.9. (the signals for the two carbons adjacent to boron were not observed); **^{11}B NMR** ($CDCl_3$, 160 MHz) δ 39.3; **IR** 2959, 2930, 2858, 1629, 1609, 1523, 1473, 1463, 1446, 1351, 1285, 1258, 1169, 1147, 1115, 976, 924, 827, 784 cm^{-1}; **HRMS** (DART) calcd. for $C_{21}H_{35}BNSi$ [M+H$^+$]: 340.2626, found 340.2633.

C3,C5-*bis*-^{n}propyl-1,2-azaborine 29

According to general Negishi coupling procedure (6.0 equiv. ^{n}Pr–ZnBr, 0.5 M in THF was used as organozinc reagent), the crude was purified by flash column chromatography using 100% hexane to give the desired product **29** as a colorless oil (98.0 mg, 0.247 mmol, 78%).

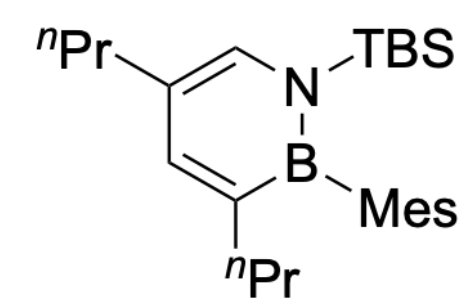

^{1}H NMR ($CDCl_3$, 500 MHz) δ 7.23 (s, 1H), 7.06 (s, 1H), 6.76 (s, 2H), 2.40 (t, *J* = 13.0 Hz, 2H), 2.29 (s, 3H), 2.08 (t, *J* = 14.7 Hz, 2H), 2.04 (s, 6H), 1.60–1.52 (m, 2H), 1.30–1.22 (m, 2H), 0.95 (t, *J* = 7.3 Hz, 3H), 0.92 (s, 9H), 0.77 (t, *J* = 7.3 Hz, 3H), –0.05 (s, 6H); **^{13}C NMR** ($CDCl_3$, 126 MHz) δ 142.1, 139.0, 136.0, 133.4, 126.9, 123.3, 37.0, 36.3, 27.9, 24.4, 23.4, 23.3, 21.4, 19.4, 14.31, 14.0, –2.9 (the signals for the two carbons adjacent to boron were not observed); **^{11}B NMR** ($CDCl_3$, 160 MHz) δ 39.5; **IR** 2958, 2929, 2860, 1624, 1609, 1518, 1472, 1402, 1374, 1278, 1263, 1167, 1148, 848, 784 cm^{-1}; **HRMS** (DART) calcd. for $C_{25}H_{43}BNSi$ [M+H$^+$]: 396.3252, found 396.3254.

<u>Synthesis of C3-Me C5-Bn 1,2-azaborine **30**</u>

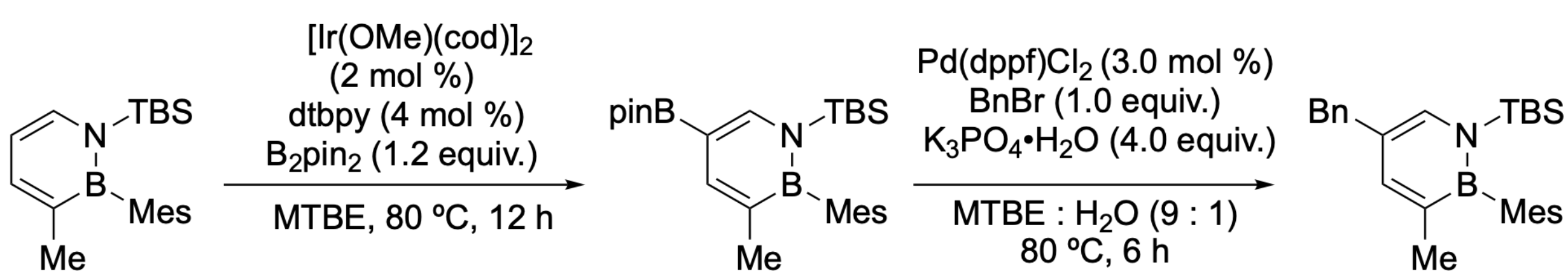

13 **30**

Step 1: In a glove box, a 15 mL pressure vessel was charged with C3-methyl-1,2-azaborine **13** (131 mg, 0.402 mmol, 1.0 equiv.), $[Ir(OMe)(cod)]_2$ (5.3 mg, 8.0 μmol, 2 mol%), 4,4'-di-tert-butyl-2,2'-bipyridine (dtbbpy) (4.3 mg, 16 μmol, 4 mol%), bis(pinacolato)diboron (B_2pin_2) (123 mg, 0.482 mmol, 1.2 equiv.) and 2.0 mL methyl tert-butyl ether. The reaction mixture was sealed and stirred at 80 °C in an oil bath for 12 hours. At the conclusion of the reaction, the reaction mixture was concentrated *in vacuo*. The crude residue was purified by flash column chromatography using 0 –10 % dichloromethane in hexane to give the desired C3-methyl-C5-Bpin product as a white solid (136 mg, 0.301 mmol, 75%).

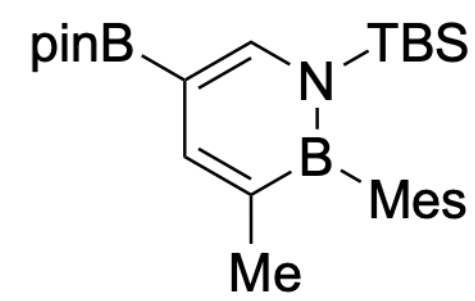

^{1}H NMR ($CDCl_3$, 500 MHz) δ 7.90 (s, 1H), 7.65 (s, 1H), 6.78 (s, 2H), 2.30 (s, 3H), 2.03 (s, 6H), 1.80 (s, 3H), 1.33 (s, 12H), 0.93 (s, 9H), –0.01 (s, 6H). **^{13}C NMR** ($CDCl_3$, 126 MHz) δ 146.0, 144.0, 138.5, 136.3, 127.0, 83.3, 27.7, 25.0, 22.8, 21.4, 21.4, 19.4, –3.0. (the signals for the three carbons adjacent to boron were not observed); **^{11}B NMR** ($CDCl_3$, 160 MHz) δ 41.2, 31.0.

Step 2: In a glove box, a 15 mL pressure vessel was charged with a magnetic stir bar, C3-methyl-C5-Bpin-1,2-azaborine (120 mg, 0.266 mmol, 1.0 equiv.), benzyl bromide (54.7 mg, 0.320 mmol, 1.2 equiv.), $Pd(dppf)Cl_2$ (6.5 mg, 8.0 μmol, 3 mol%), $K_3PO_4 \cdot H_2O$ (194 mg, 0.80 mmol, 3.0 equiv.) and 2.5 mL methyl tert-butyl ether. 0.25 mL H_2O was added into the pressure vessel and the reaction mixture was sealed and allowed to stir at 80 °C in an oil bath for 14 hours. At the conclusion of the reaction, water was added into the reaction mixture and the aqueous phase was extracted with diethyl ether. The combined organic extracts were washed with brine, dried over $MgSO_4$, vacuum-filtered through a fritted funnel, and concentrated *in vacuo*. The crude residue was purified by flash column chromatography using 0–2% diethyl ether in hexane to give the desired product **35** as a white solid (84 mg, 0.201 mmol, 76%).

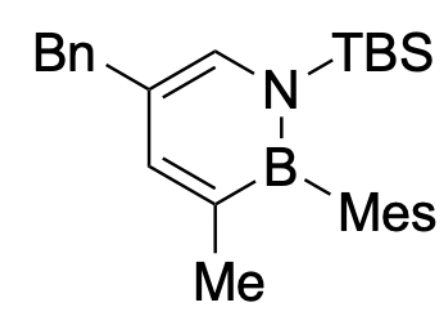

^{1}H NMR ($CDCl_3$, 500 MHz) δ 7.34–7.26 (m, 2H), 7.24–7.19 (m, 4H), 6.96 (s, 1H), 6.76 (s, 2H), 3.78 (s, 2H), 2.28 (s, 3H), 2.03 (s, 6H), 1.75 (s, 3H), 0.84 (s, 9H), –0.09 (s, 6H); **^{13}C NMR** ($CDCl_3$, 126 MHz) δ 142.8, 141.5, 138.7, 136.2, 134.4, 129.2, 128.5, 127.0, 126.1, 122.6, 40.0, 27.7, 22.9, 21.7, 21.4, 19.3, –3.0 (the signals for the two carbons adjacent to boron were not observed); **^{11}B NMR** $CDCl_3$, 160 MHz) δ 39.5; **IR** 2958, 2930, 2857, 1625, 1541, 1473, 1455, 1281, 1263, 1164, 1110, 828, 784 cm^{-1}; **HRMS** (DART) calcd. for $C_{27}H_{39}BNSi$ [M+H^{+}]: 416.2939, found 416.2941.

<u>Procedures for C3C6-disubstituted 1,2-azaborine **31**</u>

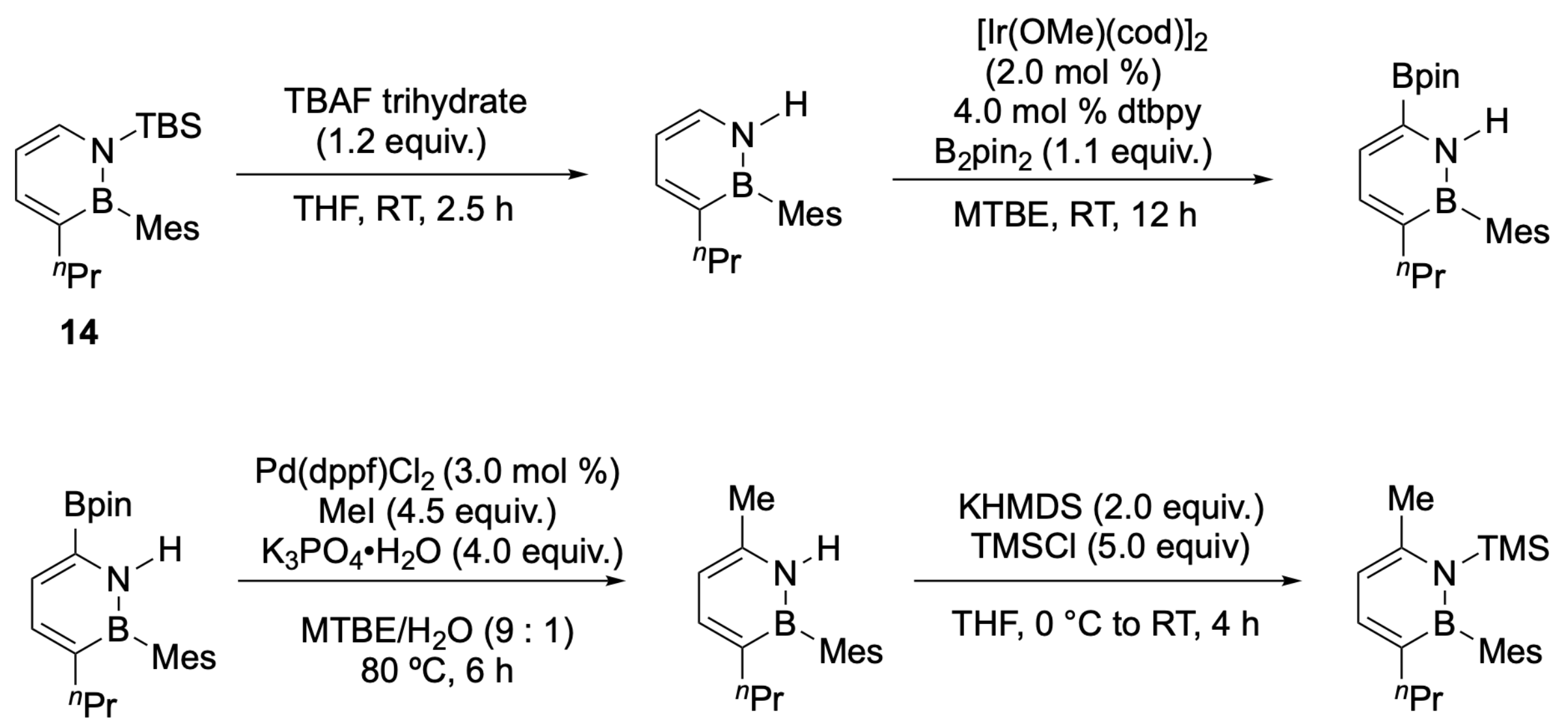

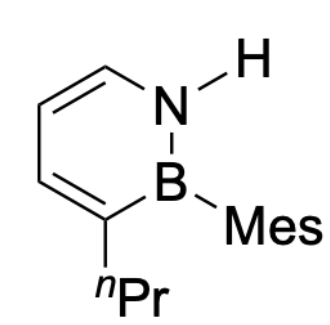

Step 1: A 50 mL RBF was charged with a magnetic stir bar. C3-propyl 1,2-azaborine **14** (488 mg, 1.38 mmol, 1.0 equiv.) and 8 mL THF. TBAF trihydrate (523 mg, 1.66 mmol, 1.2 equiv.) was then added and the reaction mixture was allowed to stir at room temperature for 2.5 hours. At the conclusion of the reaction, the reaction mixture was concentrated *in vacuo* and the residue was purified by flash column chromatography using 0 – 7% diethyl ether in hexane to give the desired C3-propyl N–H 1,2-azaborine product as a pale yellow oil (309 mg, 1.29 mmol, 94%).

C3-^{n}propyl C6-Bpin N-H-1,2-azaborine

Step-2: In a glovebox, a 50 mL round bottom flask was charged with a magnetic stir bar, C3-propyl N–H 1,2-azaborine (309 mg, 1.29 mmol, 1.0 equiv.), $[Ir(OMe)(cod)]_2$ (17 mg, 0.026 mmol, 2.0 mol%), 4,4'-di-tert-butyl-2,2'-bipyridine (dtbbpy) (14 mg, 0.052 mmol, 4.0 mol%), bis(pinacolato)diboron (B_2pin_2) (361 mg, 1.42 mmol, 1.1 equiv.) and 10.0 mL MTBE. The reaction mixture was allowed to stir at room temperature for 12 hours. At the conclusion of the reaction, the reaction mixture was concentrated *in vacuo* and the residue was purified by flash column chromatography using 5–10% diethyl ether in hexane to give the desired C3-propyl C6-Bpin N–H 1,2-azaborine product as a white solid (281 mg, 1.18 mmol, 60%).

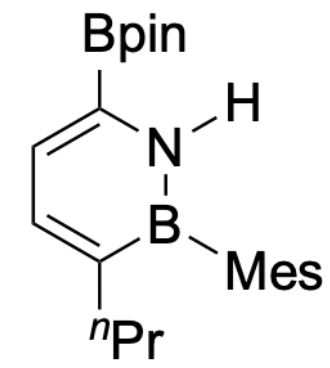

C3-^{n}propyl C6-methyl N-H-1,2-azaborine

Step 3: A 15 mL pressure vessel was charged with a magnetic stir bar, C3-propyl C6-Bpin N–H 1,2-azaborine (281 mg, 0.770 mmol, 1.0 equiv.), Methyl iodide (492 mg, 3.47 mmol, 4.5 equiv.), $Pd(dppf)Cl_2$ (19 mg, 23 μmol, 3.0 mol%), $K_3PO_4 \cdot H_2O$ (709 mg, 2.31 mmol, 4.0 equiv.), and 5.4 mL methyl tert-butyl ether. 0.6 mL H_2O was added into the pressure vessel and the reaction mixture was sealed and allowed to stir at 80 °C in an oil bath for 6 hours. At the conclusion of the reaction, water was added into the reaction mixture and the aqueous phase was extracted with diethyl ether. The combined organic extracts were washed with brine, dried over $MgSO_4$, vacuum-filtered through a fritted funnel, and concentrated *in vacuo*. The crude residue was purified by flash column chromatography using 0–2% diethyl ether in hexane to give the desired product C3-propyl C6-methyl N–H 1,2-azaborine as a white solid (169 mg, 0.667 mmol, 86%).

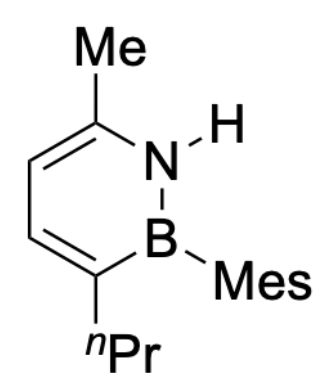

C3-propyl-C6-methyl-1,2-azaborine 31

Step 4: A 20 mL vial was charged with a magnetic stir bar, C3-propyl C6-methyl *N*–H 1,2-azaborine (105 mg, 0.416 mmol, 1.0 equiv.), and 1.0 mL THF. The resulting mixture was cooled down to 0°C before KHMDS (1.7 mL, 0.832 mmol, 2.0 equiv., 0.5M in toluene) was added dropwise via syringe over 5 minutes. The reaction mixture was gradually warmed up to room temperature while being stirred for additional 1 hour. Then, TMSCl (0.26 mL, 2.1 mmol, 5.0 equiv.) was added in one portion and the reaction was allowed to stir at room temperature for three hours. At the conclusion of the reaction, the reaction mixture was concentrated *in vacuo* the crude residue was purified by flash column chromatography using 100% hexane to give the desired product **36** as a colorless oil (85.8 mg, 0.264 mmol, 63%).

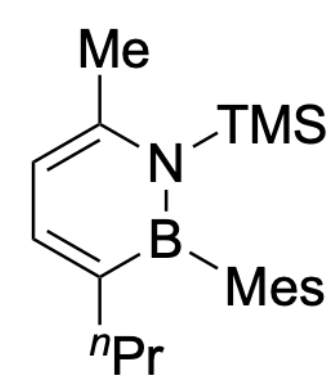

^{1}H NMR ($CDCl_3$, 500 MHz) δ 7.22 (d, J = 6.8 Hz, 1xH), 6.74 (s, 2H), 6.21 (d, J = 6.8 Hz, 1H), 2.52 (s, 3H), 2.29 (s, 3H), 2.03 (s, 6H), 1.95 (t, J = 6.7 Hz, 2H), 1.29–1.15 (m, 2H), 0.72 (t, J = 7.3 Hz, 3H), 0.13 (s, 9H); **^{13}C NMR** ($CDCl_3$, 126 MHz) δ 143.8, 140.6, 138.6, 136.1, 126.9, 114.5, 36.5, 25.0, 23.4, 23.2, 21.4, 14.3, 5.0 (the signals for the two carbons adjacent to boron were not observed); **^{11}B NMR** ($CDCl_3$, 160 MHz) δ 40.7; **IR** 2956, 2924, 2869, 1606, 1538, 1453, 1372, 1357, 1257, 1159, 1068, 1024, 843, 809, 767 cm^{-1}; **HRMS** (DART) calcd. for $C_{20}H_{33}BNSi$ [M+H$^+$]: 326.2470, found 326.2462.

General Procedure for 1,2-Azaborine Photoisomerization

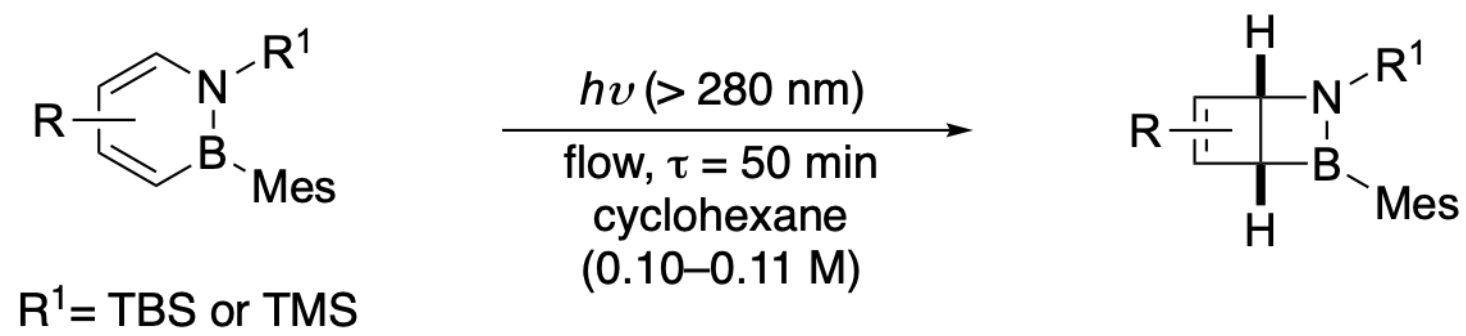

The flow photoreactor setup used in this work was assembled in accordance with prior system designs by Berry and Booker-Milburn and Seeberger and Hartmann.[2] Prior to each use of the reactor, 6 mL of anhydrous cyclohexane were flushed through the tubing. The total volume of the reaction tube is 5.0 mL. An efficient photoisomerization reaction typically generates the Dewar isomer as the only product with no 1,2-azaborine starting material left, in which case, purification is not required. All the NMR spectra of Dewar compounds shown in this work are the "crude" NMR taken after the removal of cyclohexane solvent. Yield was calculated based on the product mass recovered after removing the cyclohexane solvent. The photoisomerization of each 1,2-azaborine compound was carried out at least 2 times. The average yield was reported.

The preparation of Parent Dewar **1a** is representative. The first syringe with anhydrous cyclohexane (3.2 mL) solution of azaborine **12** (100 mg, 0.321 mmol) was injected into FEP tubing at 0.1 mL / min by syringe pump into the flow photoreactor. The latter syringe with 5.5 mL of pure cyclohexane was then injected at the same rate. After 1.0 mL of cyclohexane in the latter syringe was injected, the solution from the tubing was collected into a 20 mL vial. The solution collected in the vial was concentrated *in vacuo* under N_2 to afford the product as pale yellow liquid with 93% average yield (Batch 1: 93.2 mg, 0.300 mmol, 93%; Batch 2: 92.1 mg, 0.296 mmol, 92%).

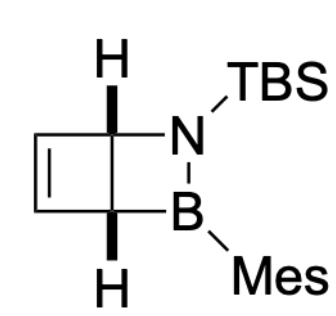

^{1}H NMR ($CDCl_3$, 500 MHz) δ 6.74 (s, 2H), 6.55 (dt, *J* = 2.4, 1.2 Hz, 1H), 6.36 (d, *J* = 2.3 Hz, 1H), 4.78 (t, *J* = 2.2 Hz, 1H), 3.08–2.92 (m, 1H), 2.25 (s, 3H), 2.20 (s, 6H), 0.90 (s, 9H), –0.06 (s, 3H), –0.11 (s, 3H); **^{13}C NMR** ($CDCl_3$, 126 MHz) δ

143.7, 139.3, 138.3, 137.1, 126.9, 63.4, 26.5, 22.4, 21.3, 18.4, –5.4, –5.7 (the signals for the two carbons adjacent to boron were not observed); **^{11}B NMR** ($CDCl_3$, 160 MHz) δ 52.5.

C3-methyl-BN-Dewar benzene 13a

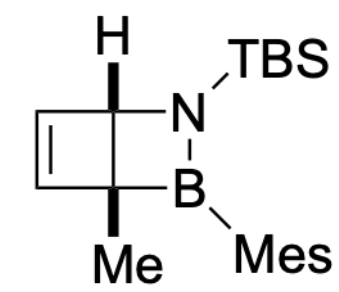

91% average yield (Batch 1: 29.0 mg, 0.891 mmol, 89%; Batch 2: 30.2 mg, 0.928 mmol, 93%). **^{1}H NMR** ($CDCl_3$, 500 MHz) δ 6.75 (d, J = 5.3 Hz, 2H), 6.54 (d, J = 2.4, 1H), 6.38 (d, J = 2.4 Hz, 1H), 4.34 (d, J = 2.5 Hz, 1H), 2.27 (d, J = 6.8 Hz, 6H), 2.16 (s, 3H), 1.31 (s, 3H), 0.90 (s, 6H), –0.08 (s, 3H), –0.14 (s, 3H). **^{13}C NMR** ($CDCl_3$, 126 MHz) δ 148.7, 137.5, 137.0, 127.0, 126.9, 68.3, 26.5, 21.3, 18.4, 15.3, 1.2, –5.4, –5.6 (the signals for the two carbons adjacent to boron were not observed); **^{11}B NMR** ($CDCl_3$, 160 MHz) δ 53.3; **IR** 2952, 2929, 2857, 1611, 1471, 1458, 1389, 1356, 1251, 1178, 986, 884, 834, 802 cm^{-1}; **HRMS** (DART) calcd. for $C_{20}H_{33}BNSi$ [M+H$^+$]: 326.2470, found 326.2478.

C3-^{n}propyl-BN-Dewar benzene 14a

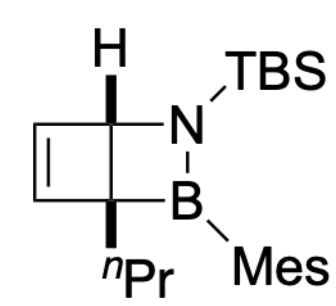

91% average yield (Batch 1: 35.2 mg, 0.100 mmol, 93%; Batch 2: 33.9 mg, 0.0960 mmol, 89%). **^{1}H NMR** ($CDCl_3$, 600 MHz) δ 6.73 (d, J = 10.7 Hz, 2H), 6.60 (t, J = 2.4 Hz, 1H), 6.41 (d, J = 2.3 Hz, 1H), 4.42 (d, J = 2.5 Hz, 1H), 2.26 (d, J = 2.8 Hz, 6H), 2.15 (s, 3H), 1.75 (ddd, J = 13.6, 10.0, 6.0 Hz, 1H), 1.58 (ddd, J = 13.6, 9.9, 5.9 Hz, 1H), 1.28 (m, 2H), 0.90 (s, 9H), 0.83 (t, J = 7.4 Hz,3H), –0.08 (s, 3H), –0.14 (s, 3H); **^{13}C NMR** ($CDCl_3$, 151 MHz) δ 147.2, 139.0, 137.8, 136.8, (126.93, 126.86 two diastereotopic 13C), 66.9, 32.4, 26.6, (22.77, 22.12 two diastereotopic 13C), 21.4, 21.0, 18.4 14.9, –5.47, –5.51 (the signals for the two carbons adjacent to boron were not observed); **^{11}B NMR** ($CDCl_3$, 160 MHz) δ

53.4; **IR** 2955, 2928, 2857, 1643, 1360, 1264, 992, 895, 833, 777, 732, 703 cm^{-1}; **HRMS** (DART) calcd. for $C_{30}H_{37}BNSi$ [M+H_3O^+]: 354.2783, found 354.2786.

C3-benzyl-BN-Dewar benzene 15a

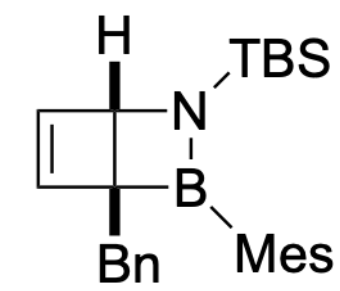

92% average yield (Batch 1: 41.8 mg, 0.104 mmol, 93%; Batch 2: 40.9 mg, 0.102 mmol, 91%). **^{1}H NMR** ($CDCl_3$, 500 MHz) δ 7.21–7.08 (m, 3H), 7.06–7.01 (m, 2H), 6.76 (d, *J* = 16.5 Hz, 2H), 6.45 (t, *J* = 2.4 Hz, 1H), 6.30 (d, *J* = 2.4 Hz, 1H), 4.47 (d, *J* = 2.5 Hz,1H), 3.14 (d, *J* = 14.1 Hz, 1H), 2.90 (d, *J* = 14.0 Hz, 1H), 2.29 (d, *J* = 3.3 Hz, 6H), 2.12 (s, 3H), 0.91 (s, 9H), –0.06 (s, 3H), –0.10 (s, 3H); **^{13}C NMR** ($CDCl_3$, 126 MHz) δ 146.1, 141.1, 137.7, 137.1, 129.0, 128.0, 127.0, 127.0, 125.6, 66.2, 36.3, 26.6, 22.9, 22.1, 21.4, 18.4. –5.49 (the signals for the two carbons adjacent to boron were not observed); **^{11}B NMR** ($CDCl_3$, 160 MHz) δ 52.8; **IR** 2968, 2920, 2858, 1743, 1488, 1363, 1208, 992, 895, 843, 795 cm^{-1}; **HRMS** (DART) calcd. for $C_{26}H_{37}BNSi$ [M+H_3O^+]: 402.2783, found 402.2779.

C3-Br-BN-Dewar benzene 16a

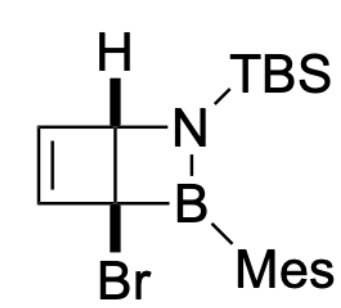

91% average yield (Batch 1: 92.2 mg, 0.236 mmol, 92%; Batch 2: 90.3 mg, 0.231 mmol, 90%). **^{1}H NMR** ($CDCl_3$, 500 MHz) δ 6.79 (s, 1H), 6.74 (s, 1H), 6.58–6.50 (m, 1H), 4.77 (d, *J* = 1.5 Hz, 1H), 2.40 (s, 3H), 2.26 (s, 3H), 2.16 (s, 3H), 0.90 (s, 9H), –0.04 (s, 3H), –0.08 (s, 3H).; **^{13}C NMR** ($CDCl_3$, 126 MHz) δ 144.4, 140.0, 138.0, 127.2, 127.0, 71.8, 26.4, 21.4, 18.3, –5.5, –5.6 (the signals for the two carbons adjacent to boron were not observed); **^{11}B NMR** ($CDCl_3$, 160 MHz) δ 49.7. **IR** 2955, 2928, 2857, 1611, 1470, 1438, 1390, 1362, 1272, 1254, 1229, 1177, 1085, 1000, 807, 759 cm^{-1}; **HRMS** (DART) calcd. for $C_{19}H_{30}BBrNSi$ [M+H^+]: 390.1423, found 390.1430.

C3-*p*-OMe-Ph BN-Dewar 17a

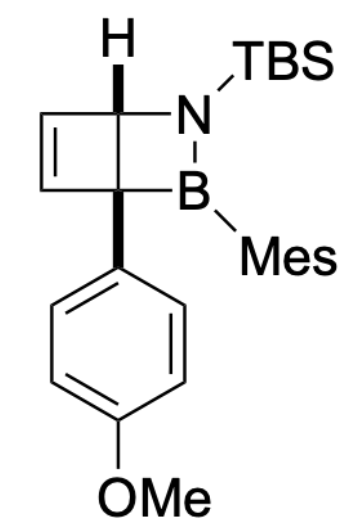

Retention time = 20 mins, CH_2Cl_2 was used for solvent.

89% average yield (Batch 1: 37.1 mg, 0.0888 mmol, 89%; Batch 2: 37.7 mg, 0.0903 mmol, 90%). **^{1}H NMR** ($CDCl_3$, 500 MHz) δ 7.05 (d, *J* = 8.6 Hz, 2H), 6.76 (d, *J* = 8.6 Hz, 2H), 6.77–6.67 (m, 3H), 6.53 (d, *J* = 2.3 Hz, 1H), 4.84 (s, 1H), 3.74 (s, 3H), 2.25 (s, 6H), 2.05 (s, 3H), 0.92 (s, 9H), –0.03 (s, 3H), –0.12 (s, 3H); **^{13}C NMR** ($CDCl_3$, 126 MHz) δ 157.1, 147.0, 138.2, 137.0, 133.5, 128.9, 127.6, 126.9, 113.7, 68.7, 55.2, 26.4, 21.2, 18.2, –5.6, –5.7 (the signals for the two carbons adjacent to boron were not observed); **^{11}B NMR** ($CDCl_3$, 160 MHz) δ 52.4; **IR** 2953, 2929, 2856, 1610, 1510, 1464, 1440, 1360, 1284, 1244, 1219, 1177, 1037, 997, 832 cm^{-1}; **HRMS** (DART) calcd. for $C_{26}H_{37}BNOSi$ [M+H$^+$]: 418.2732, found 418.2749.

C4-methyl-BN-Dewar benzene 18a

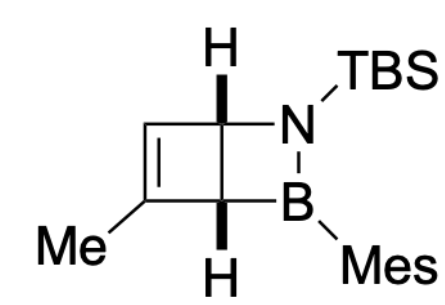

93% average yield (Batch 1: 38.7 mg, 0.119 mmol, 91%; Batch 2: 39.9 mg, 0.123 mmol, 94%). **^{1}H NMR** ($CDCl_3$, 500 MHz) δ 6.79 (s, 2H), 6.07 (t, *J* = 1.4 Hz, 1H), 4.64 (d, *J* = 2.3 Hz, 1H), 2.90 (d, *J* = 2.3 Hz, 1H), 2.29 (s, 3H), 2.23 (s, 6H), 1.86–1.77 (s, 3H), 0.92 (s, 9H), –0.06 (s, 3H), –0.10 (s, 3H); **^{13}C NMR** ($CDCl_3$, 126 MHz) δ 153.7, 138.0, 136.9, 131.6, 126.9, 59.1, 29.9, 26.5, 22.3, 21.3, 18.2, –5.4, –5.8 (the signals for the two carbons adjacent to boron were not observed); **^{11}B NMR** ($CDCl_3$, 160 MHz) δ 52.2; **IR** 2953, 2929, 2857, 1610, 1470, 1462, 1351, 1253, 1174, 994, 847, 818, 778, 740 cm^{-1}; **HRMS** (DART) calcd. for $C_{20}H_{33}BNSi$ [M+H$_3$O$^+$]: 326.2470, found 326.2482.

C4-Bpin-BN-Dewar benzene 19a

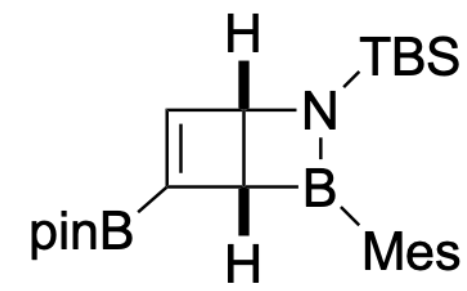

93% average yield (Batch 1: 100.0 mg, 0.229 mmol, 93%; Batch 2: 99.1 mg, 0.227 mmol, 92%). **^{1}H NMR** ($CDCl_3$, 600 MHz) δ 7.13 (s, 1H), 6.72 (s, 2H), 4.77 (d, *J* = 2.1 Hz, 1H), 3.14 (d, *J* = 2.1 Hz, 1H), 2.26 (s, 3H), 2.18 (br s, 6H), 1.25 (d, *J* = 6.0 Hz, 12H), 0.90 (s, 9H), –0.07 (s, 3H), –0.13 (s, 3H); **^{13}C NMR** ($CDCl_3$, 151 MHz) δ 153.8, 136.8, 126.7, 83.5, 63.5, 27.1, 26.5, 25.0, 24.7, 21.3, 18.3, –5.5, –5.6 (the signals for the three carbons adjacent to boron were not observed); **^{11}B NMR** ($CDCl_3$, 160 MHz) δ 49.2, 24.0; **IR** 2955, 2928, 2857, 1617, 1574, 1446, 1360, 1261, 1214, 1001, 895, 833, 785, 732 cm^{-1}; **HRMS** (DART) calcd. for $C_{25}H_{42}B_2NO_2Si$ [M+H$^+$]: 438.3165, found 438.3175.

C5-methyl-BN-Dewar benzene 20a

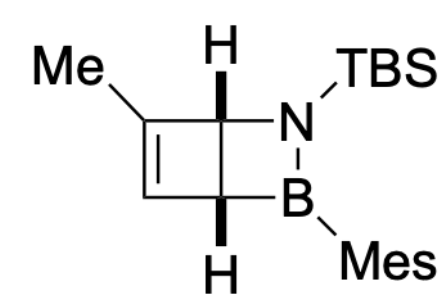

94% average yield (Batch 1: 70.3 mg, 0.215 mmol, 94%; Batch 2: 71.0 mg, 0.218 mmol, 95%). **^{1}H NMR** ($CDCl_3$, 500 MHz) δ 6.75 (s, 2H), 6.16 (dt, *J* = 2.8, 1.4 Hz,1H), 4.68 (t, *J* = 2.4 Hz, 1H), 2.86 (dt, *J* = 2.7, 1.4 Hz, 1H), 2.29 (s, 3H), 2.25 (s, 6H), 1.87 (t, *J* = 1.9 Hz, 3H), 0.93 (s, 9H), 0.14 (s, 3H), –0.14 (s, 3H); **^{13}C NMR** ($CDCl_3$, 126 MHz) δ 148.9, 138.1, 137.0, 134.7, 126.9, 64.6, 26.6, 22.4, 21.3, 18.4, 16.0, –3.3, –5.1 (the signals for the two carbons adjacent to boron were not observed); **^{11}B NMR** ($CDCl_3$, 160 MHz) δ 52.6; **IR** 2953, 2929, 2857, 1610, 1462, 1351, 1253, 1174, 995, 847, 832, 804, 778 cm^{-1}; **HRMS** (DART) calcd. for $C_{20}H_{33}BNSi$ [M+H$^+$]: 326.2470, found 326.2493.

C5-ethyl-Dewar benzene 21a

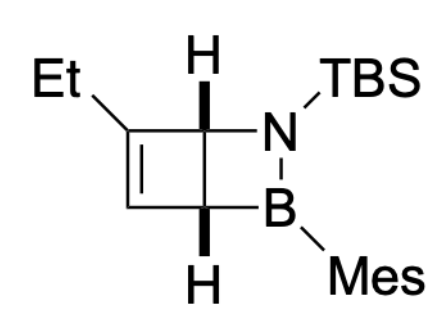

93% average yield (Batch 1: 50.1 mg, 0.148 mmol, 94%; Batch 2: 49.3 mg, 0.145 mmol, 93%). **^{1}H NMR** ($CDCl_3$, 500 MHz) δ 6.75 (s, 2H), 6.15–6.11 (m, 1H), 4.69 (t, *J* = 2.4 Hz, 1H), 2.86–2.82 (m, 1H), 2.26 (s, 3H), 2.21 (br s, 6H),

2.20–2.08 (m, 2H), 1.09 (t, J = 7.4 Hz, 3H), 0.91 (s, 9H), 0.10 (s, 3H), –0.19 (s, 3H); **^{13}C NMR** ($CDCl_3$, 126 MHz) δ 155.4, 138.2, 137.0, 132.6, 126.9, 63.3, 29.9, 26.6, 23.5, 21.3, 18.4, 11.8,–3.4, –5.1 (the signals for the two carbons adjacent to boron were not observed); **^{11}B NMR** ($CDCl_3$, 160 MHz) δ 52.8; **IR** 2959, 2927, 2858, 1462, 1441, 1374, 1348, 1259, 1108, 1074, 1016, 850, 836, 825, 779 cm^{-1}; **HRMS** (DART) calcd. for $C_{21}H_{35}BNSi$ [M+H$^+$]: 340.2626, found 340.2630.

C5-CH$_2$-ester-BN-Dewar benzene 22a

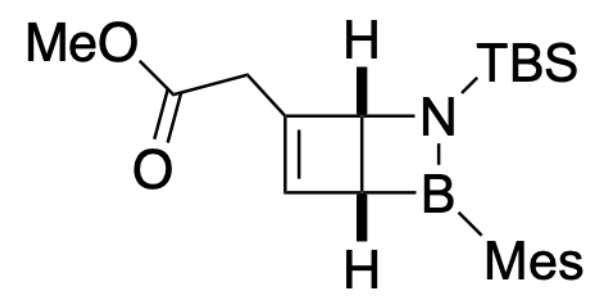

95% average yield (Batch 1: 157 mg, 0.410 mmol, 96%; Batch 2: 155 mg, 0.404 mmol, 95%). **^{1}H NMR** ($CDCl_3$, 500 MHz) δ 6.77 (s, 2H), 6.38 (dd, J = 2.9, 1.9 Hz, 1H), 4.84 (t, J = 2.4 Hz, 1H), 3.73 (s, 3H), 3.27 (dt, J = 16.9, 1.1 Hz, 1H), 3.19 (dt, J = 16.9, 2.2 Hz, 1H), 2.96 (dt, J = 2.3, 1.2 Hz, 1H), 2.27 (s, 3H), 2.22 (br s, 6H), 0.92 (s, 9H), 0.12 (s, 3H), –0.18 (s, 3H); **^{13}C NMR** ($CDCl_3$, 126 MHz) δ 171.2, 144.1, 138.4, 138.1, 137.2, 126.9, 63.7, 51.9, 35.9, 26.6, 21.3, 18.3, –3.4, –5.2 (the signals for the two carbons adjacent to boron were not observed); **^{11}B NMR** ($CDCl_3$, 160 MHz) δ 52.7; **IR** 2957, 2930, 2858, 1741, 1620, 1542, 1449, 1370, 1260, 1185, 1006, 845, 833, 770 cm^{-1}; **HRMS** (DART) calcd. for $C_{22}H_{35}BNO_2Si$ [M+H$^+$]: 384.2586, found 384.2591.

C5-*p*-F-benzyl-BN-Dewar 23a

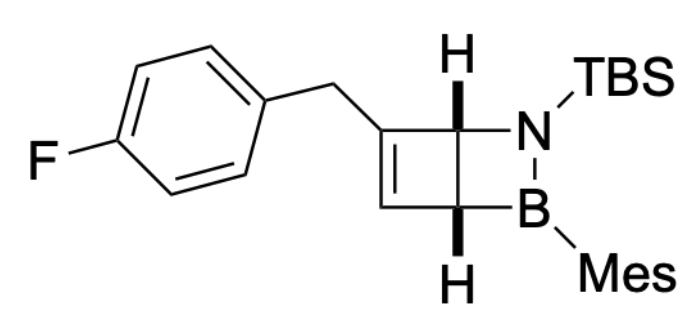

95% average yield (Batch 1: 125 mg, 0.298 mmol, 96%; Batch 2: 121 mg, 0.289 mmol, 93%). **^{1}H NMR** ($CDCl_3$, 500 MHz) δ 7.20 (dd, J = 8.5, 5.6 Hz, 2H), 7.01 (t, J = 8.7 Hz, 2H), 6.78 (s, 2H), 6.04 (dt, J = 2.1, 1.1 Hz, 1H), 4.74 (t, J = 2.4 Hz, 1H), 3.56 (d, J = 16.8 Hz, 1H), 3.43 (d, J = 16.9, 1H), 2.90 (dt, J = 2.5, 1.3 Hz, 1H), 2.28 (s, 3H), 2.25 (br s, 6H), 0.91 (s, 9H), 0.17 (s, 3H), -0.14 (s, 3H); **^{13}C NMR** ($CDCl_3$, 126 MHz) δ 161.5 (d, $^1J_{CF}$ = 243.9 Hz), 151.8, 138.1, 137.2, 135.8, 134.71 (d,

$^4J_{CF}$ = 3.3 Hz), 130.34 (d, $^3J_{CF}$ = 7.7 Hz), 127.0, 115.24 (d, $^2J_{CF}$ = 21.3 Hz), 63.3, 36.4, 26.6, 21.3, 18.3, –3.1, –5.0 (the signals for the two carbons adjacent to boron were not observed); **^{11}B NMR** ($CDCl_3$, 160 MHz) δ 52.6; **^{19}F NMR** ($CDCl_3$, 470 MHz) δ –117.4 (m); **IR** 3039, 2958, 2916, 2858, 1608, 1545, 1509, 1360, 1250, 1152, 1106, 932, 845, 797, 740 cm^{-1}; **HRMS** (DART) calcd. for $C_{26}H_{36}BNFSi$ [M+H$^+$]: 420.2689, found 420.2692.

C6-methyl-BN-Dewar benzene 24a

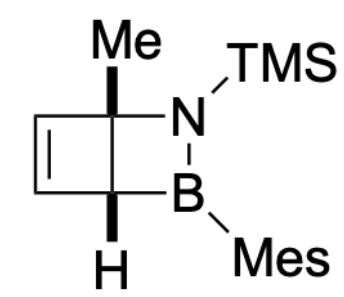

92% average yield (Batch 1: 78.0 mg, 0.275 mmol, 92%; Batch 2: 77.1 mg, 0.272 mmol, 91%). **^{1}H NMR** ($CDCl_3$, 500 MHz) δ 6.79 (s, 2H), 6.55 (dd, J = 2.4, 1.1 Hz, 1H), 6.41 (d, J = 2.3 Hz, 1H), 2.70 (d, J = 1.1 Hz, 1H), 2.30 (s, 3H), 2.25 (br s, 6H), 1.71 (s, 3H), 0.05 (s, 9H); **^{13}C NMR** ($CDCl_3$, 126 MHz) δ 141.7, 140.6, 137.1, 127.0, 72.2, 22.1, 21.8, 21.3, –0.2 (the signals for the two carbons adjacent to boron were not observed); **^{11}B NMR** ($CDCl_3$, 160 MHz) δ 51.3; **IR** 2955, 2918, 2858, 1739, 1609, 1520, 1443, 1358, 1252, 1155, 1029, 844, 803, 757 cm^{-1}; **HRMS** (DART) calcd. for $C_{17}H_{27}BNSi$ [M+H$^+$]: 284.2000, found 284.2007.

C6-ethyl-BN-Dewar benzene 25a

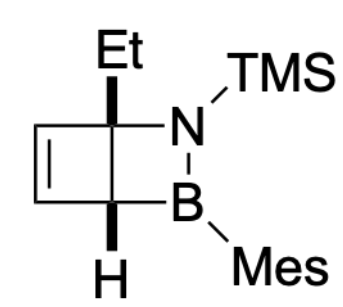

92% average yield (Batch 1: 55.5 mg, 0.187 mmol, 92%; Batch 2: 56.6 mg, 0.190 mmol, 94%). **^{1}H NMR** ($CDCl_3$, 500 MHz) δ 6.76 (s, 2H), 6.52 (dd, J = 2.4, 1.2 Hz, 1H), 6.35 (d, J = 2.4 Hz, 1H), 2.71 (d, J = 1.1 Hz, 1H), 2.27 (s, 3H), 2.25–2.18 (br s, 6H), 2.06–2.00 (m, 1H), 1.96–1.92 (m,1H), 0.99 (t, J = 7.4 Hz, 3H), 0.00 (s, 9H); **^{13}C NMR** ($CDCl_3$, 126 MHz) δ 141.0, 140.8, 137.2, 127.0, 76.8, 27.1, 27.1, 21.3, 10.1, –0.1 (the signals for the two carbons adjacent to boron were not observed); **^{11}B NMR** ($CDCl_3$, 160 MHz) δ 51.8; **IR** 2956, 2920, 2859, 1736, 1611, 1459, 1443, 1360, 1256, 1145, 1037, 847, 803, 781 cm^{-1}; **HRMS** (DART) calcd. for $C_{18}H_{29}BNSi$ [M+H$^+$]: 298.2157, found 298.2166.

C4-benzyl-C6-methyl-BN-Dewar 26a

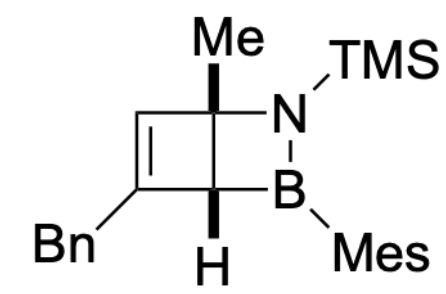

89% average yield (Batch 1: 34.2 mg, 0.0916 mmol, 89%; Batch 2: 34.3 mg, 0.0919 mmol, 89%).^{1}**H NMR** ($CDCl_3$, 500 MHz) δ 7.31–7.24 (m, 2H), 7.22–7.14 (m, 3H), 6.78 (s, 2H), 5.91 (d, J = 1.7 Hz, 1H), 3.48 (d, J = 16.8 Hz, 1H), 3.35 (d, J = 16.8 Hz, 1H), 2.56 (s, 1H), 2.27 (s, 3H), 2.23 (s, 6H), 1.63 (s, 3H), –0.03 (s, 9H). ^{13}**C NMR** ($CDCl_3$, 126 MHz) δ 152.7, 138.8, 138.1, 136.9, 134.3, 129.1, 128.4, 126.9, 126.1, 67.5, 39.1, 22.3, 22.1, 21.3, –0.2 (the signals for the two carbons adjacent to boron were not observed); ^{11}**B NMR** ($CDCl_3$, 160 MHz) δ 49.8; **IR** 3029, 2958, 2922, 2858, 1609, 1453, 1386, 1280, 1250, 1195, 1152, 1018, 969, 935, 909, 844 cm^{-1}; **HRMS** (DART) calcd. for $C_{24}H_{33}BNSi$ [M+H^{+}]: 374.2470, found 374.2475.

C4-*p*-F-benzyl-C6-methyl-BN-Dewar 27a

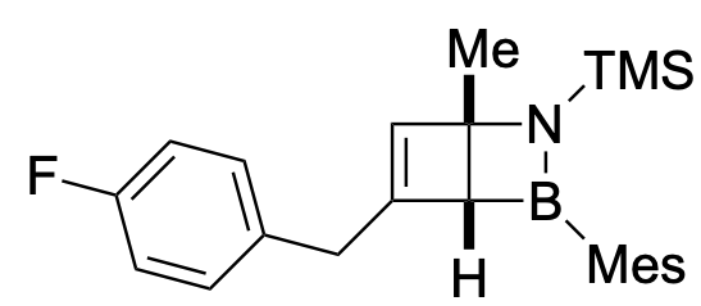

93% average yield (Batch 1: 64.1 mg, 0.164 mmol, 93%; Batch 2: 65.0 mg, 0.166 mmol, 94%). ^{1}**H NMR** ($CDCl_3$, 600 MHz) δ 7.14 (dd, J = 8.5, 5.6 Hz, 2H), 6.98 (t, J = 8.7 Hz, 2 H), 6.80 (s, 2H), 5.93 (t, J = 1.7 Hz, 1H), 3.46 (d, J = 16.9 Hz, 1H), 3.34 (d, J = 16.9 Hz, 1H), 2.57 (d, J = 1.1 Hz, 1H), 2.30 (s, 3H), 2.25 (s, 6H), 1.65 (s, 3H), –0.01 (s, 9H); ^{13}**C NMR** ($CDCl_3$, 151 MHz) δ 161.5 (d, $^{1}J_{CF}$ = 243.5 Hz), 152.6, 138.1, 137.0, 134.42, 134.40, 130.41 (d, $^{3}J_{CF}$ = 8.0 Hz) , 126.9, 115.15 (d, $^{2}J_{CF}$ = 20.9 Hz), 67.5, 38.2, 22.3, 22.0, 21.3, –0.2 (the signals for the two carbons adjacent to boron were not observed); ^{11}**B NMR** ($CDCl_3$, 160 MHz) δ 48.3; ^{19}**F NMR** ($CDCl_3$, 470 MHz) δ –117.6 (m); **IR** 3040, 2958, 2919, 2858, 1610, 1509, 1360, 1251, 1225, 1017, 970, 844, 797, 740 cm^{-1}; **HRMS** (DART) calcd. for $C_{24}H_{32}BNFSi$ [M+H^{+}]: 392.2376, found 392.2383.

C3,C5-*bis*-methyl-BN-Dewar benzene 28a

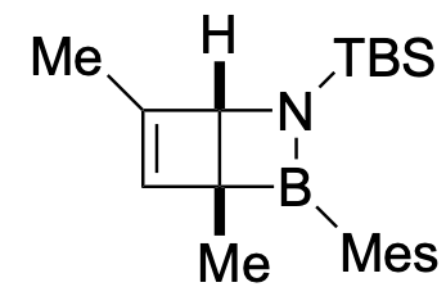

88% average yield (Batch 1: 30.3 mg, 0.0893 mmol, 89%; Batch 2: 29.8 mg, 0.878 mmol, 88%). **^{1}H NMR** ($CDCl_3$, 500 MHz) δ 6.74 (s, 2H), 6.12 (s, 1H), 4.17 (s, 1H), 2.27 (d, *J* = 9.7 Hz, 6H), 2.16 (s, 3H), 1.83 (s, 3H), 1.28 (s, 3H), 0.90 (s, 9H), 0.08 (s, 3H), –0.22 (s, 3H). **^{13}C NMR** ($CDCl_3$, 126 MHz) δ 147.3, 140.1, 136.9, 126.9, 69.7, 29.9, 26.7, 21.3, 18.4, 16.0, 15.9, 1.2, –3.1, –5.1 (the signals for the two carbons adjacent to boron were not observed); **^{11}B NMR** ($CDCl_3$, 160 MHz) δ 53.6; **IR** 2953, 2929, 2857, 1471, 1389, 1259, 1251, 1131, 1013, 986, 909, 854, 779, 734 cm^{-1}; **HRMS** (DART) calcd. for $C_{21}H_{35}BNSi$ [M+H^{+}]: 340.2626, found 340.2634.

C3,C5-*di*-^{n}propyl-BN-Dewar benzene 29a

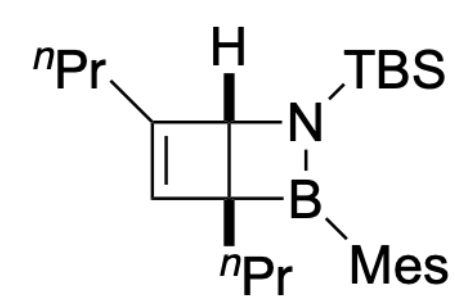

92% average yield (Batch 1: 38.7 mg, 0.0978 mmol, 92%; Batch 2: 38.3 mg, 0.969 mmol, 91%). **^{1}H NMR** ($CDCl_3$, 600 MHz) δ 6.73 (d, *J* = 5.6 Hz, 2H), 6.24–6.10 (m, 1H), 4.28 (dd, *J* = 2.8, 1.2 Hz, 1H), 2.26 (d, *J* = 6.2 Hz, 6H), 2.15 (s, 3H), 2.13–2.09 (m, 1H), 1.81–1.75 (m, 1H), 1.62–1.46 (m, 3H), 1.28 (m, 3H), 0.98 (t, *J* = 7.4 Hz, 3H), 0.91 (s, 9H), 0.84 (t, *J* = 7.4, Hz, 3H), 0.08 (s, 3H), –0.24 (s,3H); **^{13}C NMR** ($CDCl_3$, 151 MHz) δ 152.2, 138.6, 137.8, 137.2, 136.6, 126.9, 126.8, 67.0, 33.0, 32.3, 26.8, 22.9, 22.1, 21.4, 20.9, 20.9, 18.4, 14.9, 14.2, –3.0, –5.1 (the signals for the two carbons adjacent to boron were not observed); **^{11}B NMR** ($CDCl_3$, 160 MHz) δ 50.7; **IR** 2954, 2929, 2859, 1736, 1610, 1465, 1373, 1349, 1250, 1007, 992, 834, 809, 779, 743 cm^{-1}; **HRMS** (DART) calcd. for $C_{25}H_{43}BNSi$ [M+H^{+}]: 396.3252, found 396.3249.

C3-methyl-C5-benzyl-BN-Dewar 30a

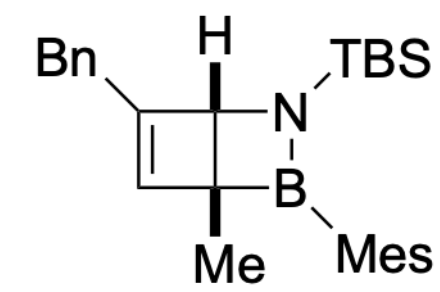

92% average yield (Batch 1: 43.8 mg, 0.105 mmol, 90%; Batch 2: 45.4 mg, 0.109 mmol, 93%). **^{1}H NMR** ($CDCl_3$, 500 MHz) δ 7.35–7.31 (m, 2H), δ 7.27–7.22 (m, 2H), 6.77 (s, 2H), 6.02 (s, 1H), 4.28 (s, 1H), 3.58 (d, J = 16.8 Hz, 1H), 3.42 (d, J = 16.8, 1H), 2.29 (d, J = 11.1 Hz, 6H), 2.20 (s, 3H), 1.32 (s, 3H), 0.91 (s, 9H), 0.15 (s, 3H), –0.19 (s, 3H). **^{13}C NMR** ($CDCl_3$, 126 MHz) δ 150.3, 140.9, 139.1, 136.9, 129.1, 128.5, 127.1, 126.1, 68.5, 37.1, 26.7, 21.3, 18.3, 15.9, –2.9, –5.1 (the signals for the two carbons adjacent to boron were not observed). **^{11}B NMR** ($CDCl_3$, 160 MHz) δ 52.8; **IR** 2950, 2930, 2856, 1471, 1456, 1435, 1375, 1349, 1250, 987, 909, 834, 778, 734 cm^{-1}; **HRMS** (DART) calcd. for $C_{27}H_{39}BNSi$ [M+H^{+}]: 416.2939, found 416.2943.

C3-^{n}propyl C6-methyl BN-Dewar 31a

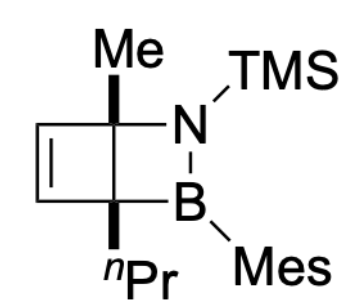

91% average yield (Batch 1: 47.0 mg, 0.144 mmol, 91%; Batch 2: 47.3 mg, 0.145 mmol, 92%). **^{1}H NMR** ($CDCl_3$, 500 MHz) δ 6.79–6.62 (d, J=13.0 Hz, 2H), 6.57 (d, J = 2.4 Hz, 1H), 6.39 (d, J = 2.4 Hz, 1H), 2.27 (d, J = 2.6 Hz, 6H), 2.14 (s, 3H), 1.62–1.56 (m, 1H), 1.54 (s, 3H), 1.46–1.40 (m, 1H), 1.36–1.30 (m, 1H), 1.26–1.20 (m, 1H), 0.81 (t, J = 7.3 Hz, 3H), –0.02 (s, 9H); **^{13}C NMR** ($CDCl_3$, 126 MHz) δ 144.8, 141.0, 139.4, 138.0, 136.8 , 126.9, 73.6, 30.6, 22.4, 22.0, 21.4, 20.7, 18.3, 15.1, –0.1 (the signals for the two carbons adjacent to boron were not observed); **^{11}B NMR** ($CDCl_3$, 160 MHz) δ 52.3; **IR** 2955, 2924, 2854, 1739, 1620, 1608, 1556, 1440, 1372, 1358, 1264, 850, 738 cm$^{–1}$; **HRMS** (DART) calcd. for $C_{20}H_{33}BNSi$ [M+H^{+}]: 326.2470, found 326.2478.

General Procedure for the Sequential Hydrogenation and Oxidation

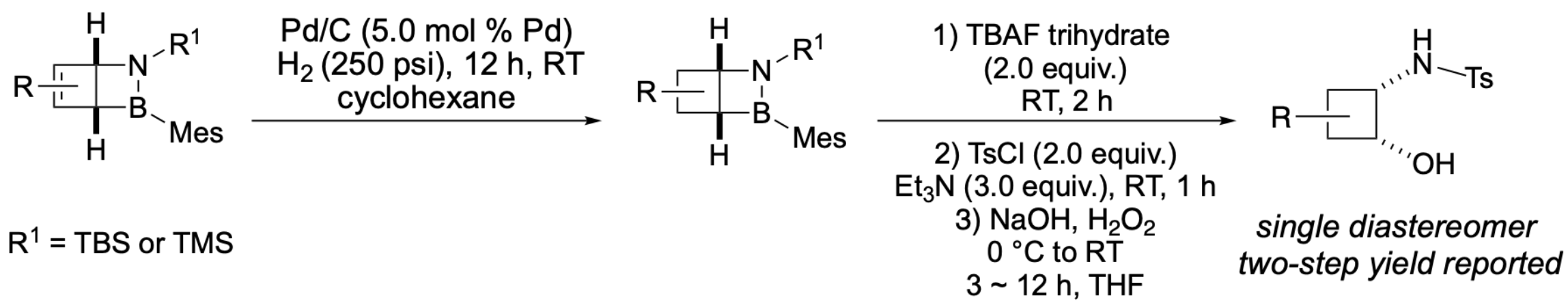

General Procedure for Hydrogenation:

In a glovebox, 4 mL or 20 mL scintillation vial was charged with magnetic stir bar, 10 wt. % Palladium on carbon (5.0 mol % of Pd) and cyclohexane solution of collected Dewar substrate (0.05–0.08 M). The vial was sealed with septum screw cap, and a 18G x 1 1/2 needle (1.2 mm x 40 mm) was fitted on the septum screw cap for atmosphere exchange. The vial was then placed in a Parr pressure vessel. The pressure vessel was sealed and transferred into a fume hood with the safety blast shield placed. The pressure vessel was pressurized, released and back-filled with hydrogen gas five times before charged with 250 psi hydrogen (caution: fix the hydrogen gas tank firmly during the whole process; apply hydrogen slowly through a pressure regulator; gently open the venting valve to release pressure). The pressure vessel was placed in the middle of stir plate and the reaction was allowed to vigorously stir for 12 hours. After 12 hours, the venting valve of pressure vessel was slowly opened to release H_2 carefully. The Parr pressure vessel was purged with N_2 for 5 minutes before brought into glovebox. The reaction mixture was filtered to remove the heterogenous catalyst and the filtrate was concentrated *in vacuo* under N_2 atmosphere. The hydrogenated intermediate was used directly for the subsequent oxidation step without further purification. Hydrogenation of each BN-Dewar compound was repeated at least twice.

General Procedure for Oxidation:

In a N_2 glovebox, 20 mL vial was charged with a magnetic stir bar, hydrogenated intermediate (1.0 equiv.) and THF (concentration is around 0.1 M). TBAF trihydrate (2.0 equiv.) was then added to the solution and the reaction mixture was allowed to stir for 2 hours at room temperature. At this point, an aliquot was checked by ^{11}B NMR spectra, which indicates the formation of tetra-coordinated borate species after ring-opening. Then, the ring-opened intermediate was trapped by 2.0 equiv. tosyl chloride with additive of 3.0 equiv. triethylamine. Precipitates were observed once the triethylamine was added. The reaction mixture was allowed to stir for 1 hour. Then, the crude mixture was filtered to remove the precipitates and the filtrate was combined in another 20 mL vial. The vial was cooled down to 0°C in an ice/water bath before saturated NaOH aqueous solution was added slowly (For base sensitive substrates, PH = 7.0 aqueous buffer was used instead). Then 25 equiv. hydrogen peroxide (H_2O_2) 30% (w/w) in water solution was added via syringe dropwise under 0°C. The reaction was allowed to stir for 3–12 hours. At the conclusion of the reaction, the reaction was cooled down to 0 °C and 25 equiv. $Na_2S_2O_3$ saturated aqueous solution was added slowly. The aqueous layer was extracted with diethyl ether or ethyl acetate 5 times. The combined organic phase was washed with brine, dried over MgSO4, vacuum-filtered through a fritted funnel, and concentrated *in vacuo*. The crude residue was purified by flash column chromatography (silica gel) or preparative thin layer chromatography (silica gel) to give the desired cyclobutane *β*-amino alcohol product. (Although the product was generally 254 nm UV-active, PMA (Phosphomolybdic Acid) or CAM (Cerium Ammonium Molybdate) stain could be very helpful to clearly identify the product spot during TLC analysis). Yields were reported over two steps starting from the BN-Dewar substrates.

2-(*tert*-butyldimethylsilyl)-3-mesityl-2-aza-3-borabicyclo[2.2.0]hexane 4

After the hydrogenation of Parent BN-Dewar **12a**, the heterogenous catalyst was removed by filtration and the filtrate was concentrated *in vacuo* under N_2 atmosphere to give pale yellow liquid. NMR spectra was obtained without further purification.

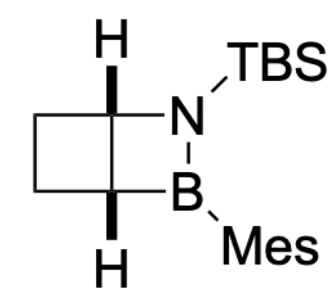

^{1}H NMR ($CDCl_3$, 500 MHz) δ 6.81 (s, 2H), 4.49 (td, J = 5.5, 3.9 Hz, 1H), 2.57 (tt, J = 10.9, 5.6 Hz, 1H), 2.49 (dt, J = 10.8, 3.8 Hz, 1H), 2.38 (br s, 6H), 2.29 (s, 3H) 2.27–2.18 (m, 1H), 2.15–1.97 (m, 2H), 0.91 (s, 9H), 0.04 (s, 3H), –0.08 (s, 3H); **^{13}C NMR** ($CDCl_3$, 126 MHz) δ 137.0, 127.0, 61.6, 30.4, 26.5, 22.7, 21.3, 18.4, 16.8, –5.0, –5.1 (the signals for the two carbons adjacent to boron were not observed); **^{11}B NMR** ($CDCl_3$, 160 MHz) δ 53.9; **IR** 2950, 2929, 2856, 1610, 1470, 1437, 1352, 1268, 1252, 1107, 1025, 980, 932, 846, 832, 775 cm^{-1}; **HRMS** (DART) calcd. for $C_{19}H_{33}BNSi$ [M+H$^+$]: 314.2470, found 314.2473.

***N*-(2-hydroxycyclobutyl)-4-methylbenzenesulfonamide 5**

Parent BN-Dewar benzene **1a** (623 mg, 2.0 mmol) was used for the telescoped hydrogenation and oxidation step. In adaptation of the general procedure, the crude residue was purified by flash column chromatography (silica gel, 25–40% ethyl acetate in hexane) to give the desired cyclobutane *β*-amino alcohol product as white solid (304 mg, 1.26 mmol, 63%).

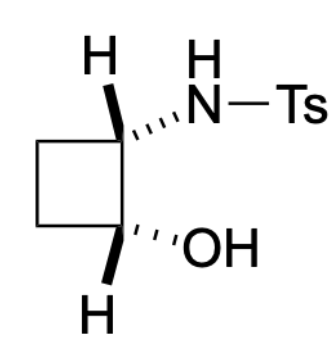

^{1}H NMR ($CDCl_3$, 600 MHz) δ 7.76 (d, J = 7.9 Hz, 2H), δ 7.29 (d, J = 7.9 Hz, 2H), 5.51 (d, J = 8.2 Hz, 1H), 4.29 (dd, J = 5.3, 2.9 Hz, 1H), 3.77 (dd, J = 8.2, 5.2 Hz, 1H), 2.58 (s, 1H), 2.41 (s, 3H) 2.12–1.90 (m, 3H), 1.78 (ddd, J = 9.0, 5.5, 2.9 Hz, 1H); **^{13}C NMR** ($CDCl_3$, 151 MHz) δ 143.7, 137.4, 129.84, 127.2, 70.2, 51.4, 26.6, 26.6, 21.7; **IR** 3492, 3275, 2955, 2930, 2872, 1598, 1495, 1337, 1307, 1152, 1087, 1018, 934, 812, 668, 551 cm^{-1}; **HRMS** (DART) calcd. for $C_{11}H_{16}NO_3S$ [M+H$^+$]: 242.0845, found 242.0853.

Crystal of **5** was grown using vapor diffusion method: 10 mg of **5** was dissolved in 0.3 mL of $CDCl_3$ in a 4 mL vial, and the uncapped 4 mL vial was placed inside a 20 mL vial with 3.5 mL of pentane. The 20 mL vial was capped and placed at ambient temperature.

***N*-(2-hydroxy-2-methylcyclobutyl)-4-methylbenzenesulfonamide 13b**

BN-Dewar benzene **13a** (38.4 mg, 0.118 mmol) was used for the telescoped hydrogenation and oxidation step. Two batches were set up in parallel. In adaptation of the general procedure, the crude residue was purified by flash column chromatography (silica gel, 25 – 40% ethyl acetate in hexane) to give the desired product as a white solid with 50% average yield (Batch 1: 16.1 mg, 0.0631 mmol, 53%; Batch 2: 14.2 mg, 0.0561 mmol, 48%).

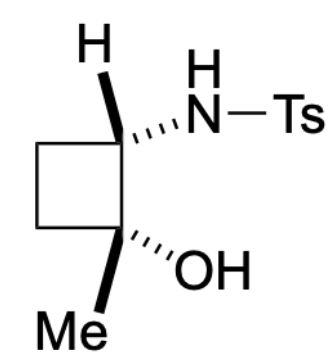

^{1}H NMR ($CDCl_3$, 600 MHz) δ 7.76 (d, *J* = 8.2 Hz, 2H), 7.30 (d, *J* = 8.2 Hz, 2H), 5.19 (s, 1H), 3.52 (ddd, *J* = 11.7, 8.2, 5.0 Hz, 1H), 2.42 (s, 3H), 2.11–1.97 (m, 1H), 1.97–1.87 (m, 1H), 1.85– 1.77 (m, 2H), 1.76–1.65 (m, 1H), 1.18 (s, 3H). **^{13}C NMR (**$CDCl_3$, 126 MHz**)** δ 143.7, 137.9, 129.8, 127.3, 55.0, 31.0, 29.9, 27.2, 25.3, 21.7. **IR** 3480, 3277, 2956, 2924, 2854, 1455, 1304, 1218, 1158, 1093, 940.9, 816, 667, 567, 551 cm^{-1}; **HRMS** (DART) calcd. for $C_{12}H_{18}NO_3S$ [M+H$^+$]: 256.1002, found 256.1007.

***N*-(2-hydroxy-2-propylcyclobutyl)-4-methylbenzenesulfonamide 14b**

BN-Dewar benzene **14a** (50.7 mg, 0.143 mmol) was used for the telescoped hydrogenation and oxidation step. Two batches were set up in parallel. In adaptation of the general procedure, the crude residue was purified by flash column chromatography (silica gel, 25 – 40% ethyl acetate in hexane) to give the desired product as a white solid with 59% average yield (Batch 1: 24.3 mg, 0.0858 mmol, 60%; Batch 2: 23.9 mg, 0.0844 mmol, 59%).

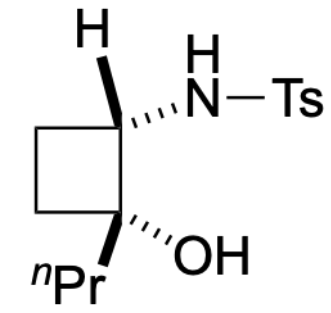

^{1}H NMR ($CDCl_3$, 600 MHz) δ 7.74 (d, *J* = 8.1 Hz, 2H), 7.28 (d, *J* = 8.1 Hz, 2H), 5.20 (d, *J* = 9.0 Hz, 1H), 3.54 (q, *J* = 8.1 Hz, 1H), 2.41 (s, 3H), 2.05–1.92 (m, 1H), 1.85 (s, 1H), 1.82–1.74 (m, 1H), 1.74–1.65 (m, 2H), 1.38–1.17 (m, 4H), 0.84 (t, *J* = 7.1 Hz, 3H); **^{13}C NMR** ($CDCl_3$, 151 MHz) δ 143.8, 138.2, 130.0, 127.5, 80.0, 54.1, 42.6, 29.6, 26.0, 21.9, 16.9, 14.6; **IR** 3485, 3274, 2956, 2930, 2871, 1599, 1495, 1334, 1304, 1158, 1093, 1019, 936, 815, 667, 550 cm^{-1}; **HRMS** (DART) calcd. for $C_{14}H_{22}NO_3S$ [$M+H^+$]: 284.1315, found 284.1321.

_N_-(2-benzyl-2-hydroxycyclobutyl)-4-methylbenzenesulfonamide 15b

BN-Dewar benzene **15a** (42.0 mg, 0.105 mmol) was used for the telescoped hydrogenation and oxidation step. Two batches were set up in parallel. In adaptation of the general procedure, the crude residue was purified by preparative thin layer chromatography (silica gel, 40% ethyl acetate in hexane) to give the desired product as a white solid with 64% average yield (Batch 1: 23.4 mg, 0.0707 mmol, 66%; Batch 2: 21.2 mg, 0.0640 mmol, 62%).

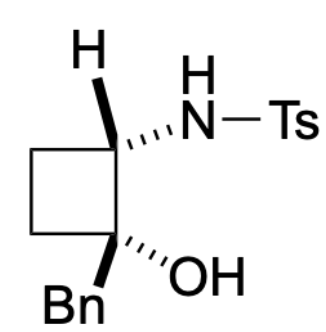

^{1}H NMR ($CDCl_3$, 500 MHz) δ 7.77 (d, *J* = 8.2 Hz, 2H), 7.37–7.18 (m, 5H), 7.12–7.03 (m, 2H), 5.23 (d, *J* = 9.3 Hz, 1H), 3.72 (td, *J* = 9.0, 7.2 Hz, 1H), 2.72 (d, *J* = 13.7 Hz, 1H), 2.56 (d, *J* = 13.7 Hz, 1H), 2.43 (s, 3H), 2.07–1.95 (m, 1H), 1.90–1.79 (m, 3H), 1.70–1.58 (m, 1H); **^{13}C NMR** ($CDCl_3$, 101 MHz) δ 143.6, 138.2, 136.1, 130.0, 129.8, 128.7, 127.3, 127.0, 79.4, 53.5, 45.9, 29.4, 26.2, 21.7; **IR** 3487, 3279, 2956, 2923, 2853, 1599, 1495, 1453, 1332, 1304, 1153, 1120, 1092, 1019, 937, 815, 703, 667, 572, 548 cm^{-1}; **HRMS** (DART) calcd. for $C_{18}H_{22}NO_3S$ [$M+H^+$]: 332.1315, found 332.1326.

***N*-(2-hydroxy-3-methylcyclobutyl)-4-methylbenzenesulfonamide 18b**

BN-Dewar benzene **18a** (43.1 mg, 0.132 mmol) was used for the telescoped hydrogenation and oxidation step. Two batches were set up in parallel. In adaptation of the general procedure, the crude residue was purified by flash column chromatography (silica gel, 25 – 40% ethyl acetate in hexane) to give the desired product as a white solid with 60% average yield (Batch 1: 20.3 mg, 60%; Batch 2: 20.7 mg, 61%).

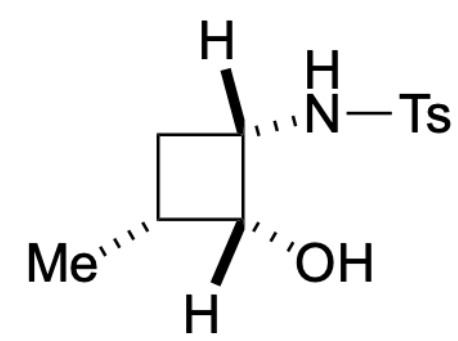

^{1}H NMR ($CDCl_3$, 400 MHz) δ 7.81–7.72 (d, *J* = 8.0 Hz, 2H), 7.30 (d, *J* = 8.0 Hz, 2H), 5.09 (d, *J* = 9.4 Hz, 1H), 4.15 (q, *J* = 4.9 Hz, 1H), 3.73–3.62 (m, 1H), 2.42 (s, 3H), 2.31–2.22 (m, 1H), 2.18–2.08 (m, 1H), 1.76 (br s, 1H), 1.60 (q, *J* = 10.1 Hz, 1H), 0.95 (d, *J* = 6.7 Hz, 3H); **^{13}C NMR** ($CDCl_3$, 101MHz) δ 143.7, 137.6, 129.8, 127.3, 72.6, 48.1, 36.2, 29.8, 21.7, 13.0; **IR** 3497, 3287, 2966, 2930, 2870, 1599, 1495, 1332, 1306, 1158, 1091, 1025, 921, 815, 671, 580, 550 cm^{-1}; **HRMS** (DART) calcd. for $C_{12}H_{18}NO_3S$ [M+H$^+$]: 256.1002, found 256.1008.

***N*-(2,3-dihydroxycyclobutyl)-4-methylbenzenesulfonamide 19b**

BN-Dewar benzene **19a** (47.7 mg, 0.109 mmol) was used for the telescoped hydrogenation and oxidation step. Two batches were set up in parallel. In adaptation of the general procedure (PH = 7.0 buffer was used in the oxidation step instead of aqueous NaOH solution), the crude residue was purified by preparative thin layer chromatography (silica gel, 72% ethyl acetate and 3% dichloromethane in hexane) to give the desired product as a white solid with 41% average yield (Batch 1: 11.5 mg, 0.0447 mmol, 42%; Batch 2: 11.0 mg, 0.0428 mmol, 41%).

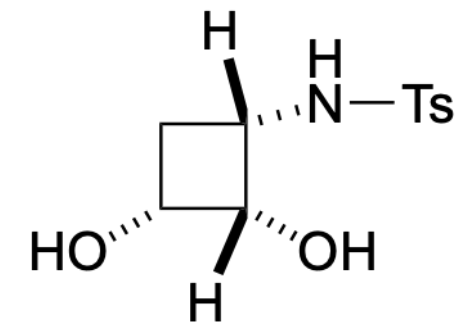

¹H NMR ($CDCl_3$, 600 MHz) δ 7.75 (d, *J* = 8.1 Hz, 2H), 7.30 (d, *J* = 8.1 Hz, 2H), 5.38 (d, *J* = 9.2 Hz, 1H), 4.24 (q, *J* = 4.5 Hz, 1H), 4.00 (d, *J* = 7.6 Hz, 1H), 3.53–3.48 (m, 1H), 3.03 (br s, 1H), 2.71 (br s, 1H), 2.57–2.50 (m, 1H), 2.43 (s, 3H), 1.85 (dt, *J* = 12.5, 7.9 Hz, 1H); **¹³C NMR** ($CDCl_3$, 126 MHz) δ 143.9, 137.2, 129.9, 127.3, 72.3, 63.6, 45.6, 38.7, 21.7; **IR** 3452, 3273, 2944, 1598, 1494, 1441, 1334, 1304, 1157, 1092, 1046, 916, 816, 667, 564, 548 cm^{-1}; **HRMS** (DART) calcd. for $C_{11}H_{16}NO_4S$ [$M+H^+$]: 258.1702, found 258.1708.

***N*-(2-hydroxy-4-methylcyclobutyl)-4-methylbenzenesulfonamide 20b**

BN-Dewar benzene **20a** (30.6 mg, 0.095 mmol) was used for the telescoped hydrogenation and oxidation step. Two batches were set up in parallel. In adaptation of the general procedure, the crude residue was purified by preparative thin layer chromatography (silica gel; 40% ethyl acetate in hexane) to give the desired product as a white solid with 59% average yield (Batch 1: 13.3 mg, 0.0521 mmol, 55%; Batch 2: 15.1 mg, 0.0592 mmol, 62%).

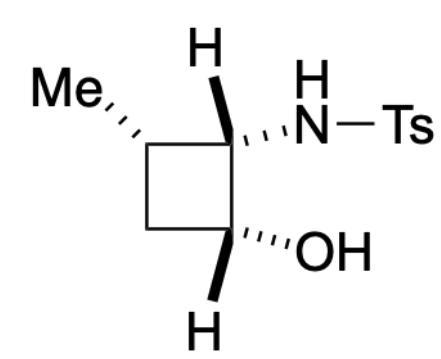

¹H NMR ($CDCl_3$, 500 MHz) δ 7.77 (d, *J* = 8.4 Hz, 2H), 7.30 (d, *J* = 8.4 Hz, 2H), 5.18 (d, *J* = 8.9 Hz, 1H), 4.26 (q, *J* = 6.2, 5.8 Hz, 1H), 3.93–3.84 (m, 1H), 2.43 (s, 3H), 2.42– 2.26 (m, 2H), 1.91 (s, 1H), 1.47–1.38 (m, 1H), 1.01 (d, *J* = 7.1 Hz, 3H); **¹³C NMR** ($CDCl_3$, 126 MHz) δ 143.7, 137.7, 129.8, 127.2, 68.0, 53.6, 35.4, 30.8, 21.7, 15.7; **IR** 3497, 3291, 2968, 2929, 2873, 1598, 1495, 1334, 1304, 1227, 1185, 1158, 1093, 922, 815, 670, 579, 549 cm^{-1}; **HRMS** (DART) calcd. for $C_{12}H_{18}NO_3S$ [$M+H^+$]: 256.10019, found 256.10075. Crystal of **20b** was grown using vapor diffusion method: 13.3 mg of **20b** was dissolved in 0.4 mL of $CDCl_3$ in a 4 mL vial, and the uncapped 4 mL vial was placed inside a 20 mL vial with 4.0 mL of pentane. The 20 mL vial was capped and placed at ambient temperature.

_N_-(2-ethyl-4-hydroxycyclobutyl)-4-methylbenzenesulfonamide 21b

The telescoped hydrogenation and oxidation sequence was performed in two batches using BN-Dewar benzene **21a** (34.4 mg, 0.101 mmol and 41.7 mg, 0.122 mmol). In adaptation of the general procedure, the crude residue was purified by flash column chromatography (silica gel; 40% ethyl acetate in hexane) to give the desired product as a white solid with 62% average yield (Batch 1: 16.4 mg, 0.0609 mmol, 60%; Batch 2: 21.2 mg, 0.0788 mmol, 64%).

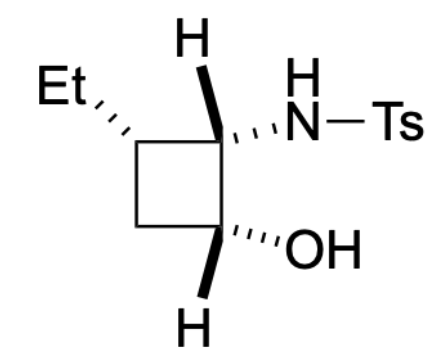

^{1}**H NMR** ($CDCl_3$, 500 MHz) δ 7.76 (d, J = 8.3 Hz, 2H), 7.30 (d, J = 8.1 Hz, 2H), 5.24 (d, J = 8.9 Hz, 1H), 4.23 (q, J = 6.9, Hz, 1H), 3.96–3.84 (m, 1H), 2.42 (s, 3H), 2.32–2.25 (m, 1H), 2.15–2.07 (m, 1H), 2.01–1.84 (br s, 1H), 1.56–1.34 (m, 3H), 0.77 (t, J = 7.3 Hz, 3H); ^{13}**C NMR** ($CDCl_3$, 126 MHz) δ 143.7, 137.5, 129.8, 127.3, 66.9, 54.0, 36.9, 34.1, 23.2, 21.7, 11.4; **IR** 3485, 3288, 2959, 2926, 2856, 1598, 1456, 1334, 1304, 1264, 1227, 1160, 1093, 814, 733, 703, 548 cm^{-1}; **HRMS** (DART) calcd. for $C_{13}H_{20}NO_3S$ [M+H$^+$]: 270.1158, found 270.1159.

methyl 2-(3-hydroxy-2-((4-methylphenyl)sulfonamido)cyclobutyl)acetate 22b

BN-Dewar benzene **22a** (55.8 mg, 0.146 mmol) was used for the telescoped hydrogenation and oxidation step. Two batches were set up in parallel. In adaptation of the general procedure (PH = 7.0 buffer was used in the oxidation step instead of aqueous NaOH solution), the crude residue was purified by flash column chromatography (silica gel, 40% ethyl acetate and 10% dichloromethane in hexane) to give the desired product as a white solid with 39% average yield (Batch 1: 17.8 mg, 0.0568 mmol, 40%; Batch 2: 17.2 mg, 0.0549 mmol, 38%).

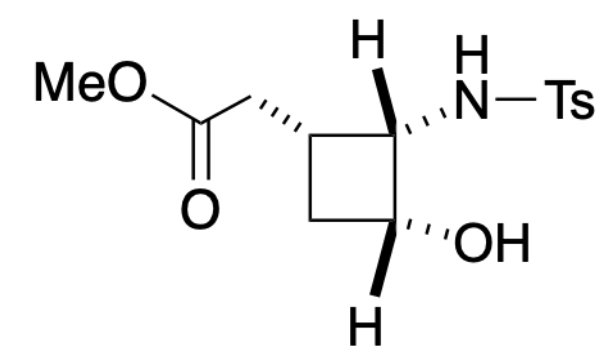

^{1}H NMR ($CDCl_3$, 600 MHz) δ 7.74 (d, J = 7.9 Hz, 2H), 7.31 (d, J = 7.9 Hz, 2H), 5.54 (d, J = 6.5 Hz, 1H), 4.28 (q, J = 6.1 Hz, 1H), 3.82 (q, J = 7.0, 6.5 Hz, 1H), 3.65 (s, 3H), 2.76–2.59 (m, 2H), 2.52 (dd, J = 16.2, 6.9 Hz, 1H), 2.42 (s, 3H), 2.41–2.36 (m, 2H), 1.70–1.61 (m, 1H); **^{13}C NMR** ($CDCl_3$, 151 MHz) δ 173.8, 143.8, 136.8, 129.9, 127.3, 67.0, 53.5, 51.9, 35.2, 33.7, 32.0, 21.7; **IR** 3497, 3294, 2951, 2851, 1734, 1598, 1495, 1441, 1336, 1202, 1160, 1092, 1047, 816, 671, 550 cm^{-1}; **HRMS** (DART) calcd. for $C_{14}H_{20}NO_5S$ [$M+H^+$]: 314.1052, found 314.1048.

***N*-(2-(4-fluorobenzyl)-4-hydroxycyclobutyl)-4-methylbenzenesulfonamide 23b**

The telescoped hydrogenation and oxidation sequence was performed in two batches using BN-Dewar benzene **23a** (92.3 mg, 0.219 mmol and 44.1 mg, 0.105 mmol). In adaptation of the general procedure, the crude residue was purified by flash column chromatography (silica gel, 25 – 40% ethyl acetate in hexane) to give the desired product as a white solid with 54% average yield (Batch 1: 42.8 mg, 0.123 mmol, 56%; Batch 2: 19.1 mg, 0.0547 mmol, 52%).

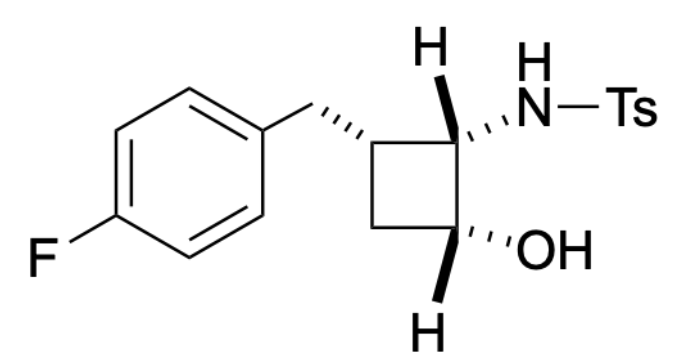

^{1}H NMR ($CDCl_3$, 500 MHz) δ 7.75 (d, J = 8.3 Hz, 2H), 7.30 (d, J = 8.0 Hz, 2H), 7.00 (dd, J = 8.5, 5.6 Hz, 2H), 6.89 (t, J = 8.7 Hz, 2H), 5.43 (d, J = 8.6 Hz, 1H), 4.28 (q, J = 6.7 Hz, 1H), 4.00–3.94 (m, 1H), 2.82–2.61 (m, 2H), 2.52–2.44 (m, 1H), 2.43. (s, 3H), 2.25–2.14 (m, 1H), 2.11–2.04 (br s, 1H) 1.71–1.55 (m, 1H); **^{13}C NMR** ($CDCl_3$, 126 MHz) δ161.5 (d, $^{1}J_{CF}$ = 243.7 Hz), 143.8, 137.4, 135.50 (d, $^{4}J_{CF}$ = 3.3 Hz), 129.99 (d, $^{2}J_{CF}$ = 7.9 Hz), 129.9, 127.3, 115.2 (d, $^{3}J_{CF}$ = 21.0 Hz), 66.8, 54.1, 36.5, 35.4, 34.0, 21.7; **^{19}F NMR** ($CDCl_3$, 470 MHz) δ –117.5 (m); **IR** 3484, 3285, 2927, 1599, 1508, 1445, 1332, 1303, 1220, 1154, 1092, 872, 813, 669, 574, 547 cm^{-1}; **HRMS** (DART) calcd. for $C_{18}H_{21}NO_3FS$ [$M+H^+$]: 350.1221, found 350.1226.

***N*-(2-hydroxy-1-methylcyclobutyl)-4-methylbenzenesulfonamide 24b**

BN-Dewar benzene **24a** (67.1 mg, 0.237 mmol) was used for the telescoped hydrogenation and oxidation step. Two batches were set up in parallel. In adaptation of the general procedure, the crude residue was purified by flash column chromatography (silica gel, 25 – 40% ethyl acetate in hexane) to give the desired product as a white solid with 54% average yield (Batch 1: 33.1 mg, 0.130 mmol, 55%; Batch 2: 31.7 mg, 0.124 mmol, 52%).

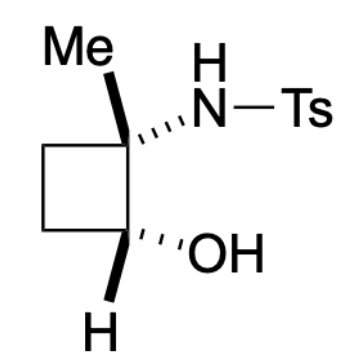

^{1}H NMR ($CDCl_3$, 500 MHz) δ 7.80 (d, J = 8.0 Hz, 2H), 7.29 (d, J = 8.0 Hz, 2H), 5.35 (s, 1H), 3.95 (t, J = 5.9 Hz, 1H), 2.75 (d, J = 3.7 Hz, 1H), 2.42 (s, 3H), 2.15–2.05 (m, 2H) 1.82–1.73 (m, 1H), 1.65–1.51 (m, 1H), 1.27 (s, 3H); **^{13}C NMR** ($CDCl_3$, 126 MHz) δ 143.3, 140.0, 129.7, 127.0, 74.1, 60.4, 29.2, 25.8, 25.1, 21.6; **IR** 3476, 3350, 2970, 2948, 1739, 1599, 1460, 1373, 1317, 1228, 1184, 1092, 948, 816, 737, 707, 666, 552 cm^{-1}; **HRMS** (DART) calcd. for $C_{12}H_{18}NO_3S$ [M+H$^+$]: 256.1002, found 256.1015. Crystal of **24b** was grown using vapor diffusion method: 15.1 mg of **24b** was dissolved in 0.4 mL of $CDCl_3$ in a 4 mL vial, and the uncapped 4 mL vial was placed inside a 20 mL vial with 4.0 mL of pentane. The 20 mL vial was capped and placed at ambient temperature.

***N*-(1-ethyl-2-hydroxycyclobutyl)-4-methylbenzenesulfonamide 25b**

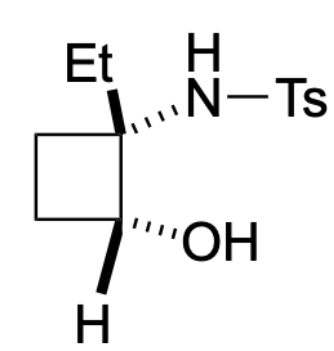

BN-Dewar benzene **25a** (62.8 mg, 0.211 mmol) was used for the telescoped hydrogenation and oxidation step. Two batches were set up in parallel. In adaptation of the general procedure, the crude residue was purified by flash column chromatography (silica gel, 25 – 40% ethyl acetate in hexane) to give the desired product as a white solid with 57% average yield (Batch 1: 31.2 mg, 0.116 mmol, 55%; Batch 2: 33.5 mg, 0.124 mmol, 59%).

^{1}H NMR ($CDCl_3$, 500 MHz) δ 7.81 (d, *J* = 8.0 Hz, 2H), 7.29 (d, *J* = 8.0 Hz, 2H), 5.26 (s, 1H), 4.00 (q, *J* = 6.1 Hz, 1H), 2.71 (d, *J* = 5.1 Hz, 1H), 2.42 (s, 3H), 2.13–2.04 (m, 2H), 1.89–1.65 (m, 2H), 1.63–1.40 (m, 2H), 0.68 (t, *J* = 7.4 Hz, 3H); **^{13}C NMR** ($CDCl_3$, 126 MHz) δ 143.2, 140.0, 129.7, 127.0, 72.0, 64.9, 30.9, 26.5, 26.1, 21.6, 7.7; **IR** 3460, 3300, 2969, 2947, 2879, 1741, 1599, 1458, 1379, 1319, 1286, 1231, 1183, 1092, 977, 816, 737, 707, 667, 551 cm^{-1}; **HRMS** (DART) calcd. for $C_{13}H_{20}NO_3S$ [M+H$^+$]: 270.1158, found 270.1166. Crystal of **25b** was grown using vapor diffusion method: 12.7 mg of **25b** was dissolved in 0.3 mL of $CDCl_3$ in a 4 mL vial, and the uncapped 4 mL vial was placed inside a 20 mL vial with 3.5 mL of pentane. The 20 mL vial was capped and placed at ambient temperature.

***N*-(3-benzyl-2-hydroxy-1-methylcyclobutyl)-4-methylbenzenesulfonamide 26b**

BN-Dewar benzene **26a** (34.2 mg, 0.0916 mmol) was used for the telescoped hydrogenation and oxidation step. Two batches were set up in parallel. In adaptation of the general procedure, the crude residue was purified by flash column chromatography (silica gel, 25 – 40% ethyl acetate in hexane) to give the desired product as a white solid with 44% average yield (Batch 1: 14.3 mg, 0.0414 mmol, 45%; Batch 2: 13.7 mg, 0.0397 mmol, 43%).

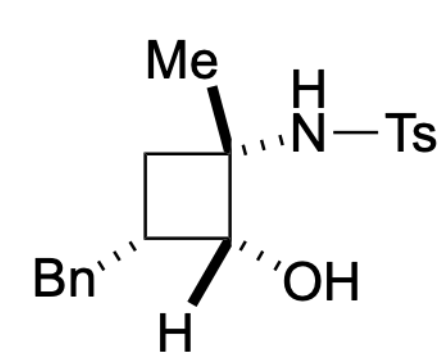

^{1}H NMR ($CDCl_3$, 500 MHz) δ 7.80 (d, *J* = 8.3 Hz, 2H), δ 7.30 (d, *J* = 8.3 Hz, 2H), 7.28–7.23 (m, 2H), 7.20–7.13 (m, 3H), 5.23 (d, *J* = 7.7 Hz, 1H), 4.04–4.00 (m, 1H), 2.91 (dd, *J* = 14.0, 8.1 Hz, 1H), 2.64 (dd, *J* = 14.0, 7.5 Hz, 2H), 2.49–2.43 (m, 1H), 2.43 (s, 3H), 1.99–1.87 (m, 2H), 1.26 (s, 3H). **^{13}C NMR (**$CDCl_3$, 126 MHz**)** δ 143.6, 140.7, 139.6, 129.7, 128.7, 128.5, 127.3, 126.1, 76.0, 55.8, 39.9, 35.1, 34.6, 23.6, 21.7; **IR** 3490, 3290, 2929, 1402, 1377, 1317, 1304, 1151, 1093, 1006, 815, 666, 553 cm^{-1} **HRMS** (DART) calcd. for $C_{19}H_{24}NO_3S$ [M+H$^+$]: 346.1471, found 346.1470.

***N*-(3-(4-fluorobenzyl)-2-hydroxy-1-methylcyclobutyl)-4-methylbenzenesulfonamide 27b**

BN-Dewar benzene **27a** (45.2 mg, 0.116 mmol) was used for the telescoped hydrogenation and oxidation step. Two batches were set up in parallel. In adaptation of the general procedure, the crude residue was purified by flash column chromatography (silica gel, 25 – 40% ethyl acetate in hexane) to give the desired product as a white solid with 54% average yield (Batch 1: 23.6 mg, 0.0650 mmol, 56%; Batch 2: 21.9 mg, 0.0603 mmol, 52%).

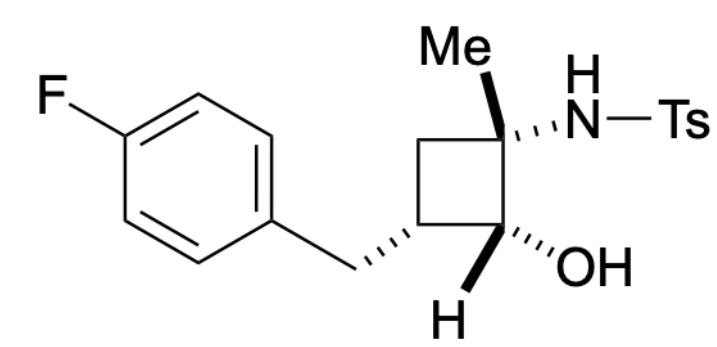

^{1}H NMR ($CDCl_3$, 500 MHz) δ 7.80 (d, J = 8.3 Hz, 2H), 7.29 (d, J = 8.0 Hz, 2H), 7.10 (dd, J = 8.5, 5.6 Hz, 2H), 6.92 (t, J = 8.7 Hz, 2H), 5.29 (s, 1H), 4.02 (dd, J = 5.9, 2.2 Hz, 1H), 2.90 (dd, J = 14.0, 8.2 Hz, 1H), 2.83–2.74 (br s, 1H), 2.59 (dd, J = 14.0, 7.4 Hz, 1H), 2.46–2.37 (m, 4H), 1.92 (d, J = 9.0 Hz, 2H), 1.24 (s, 3H); **^{13}C NMR** ($CDCl_3$, 126 MHz) δ 161.4 (d, $^1J_{CF}$ = 243.5 Hz), 143.6, 139.5, 136.38 (d, $^4J_{CF}$ = 3.0 Hz), 130.04 (d, $^3J_{CF}$ = 7.7 Hz), 129.7, 127.3, 115.2 (d, $^2J_{CF}$ = 21.0 Hz), 75.8, 55.8, 39.6, 35.2, 33.8, 23.6, 21.7; **^{19}F NMR** ($CDCl_3$, 470 MHz) δ –117.6 (m); **IR** 3501, 3284, 3029, 2970, 1738, 1600, 1509, 1375, 1317, 1218, 1152, 1093, 955, 815, 737, 706, 574, 553 cm^{-1}; **HRMS** (DART) calcd. for $C_{19}H_{23}NO_3FS$ [M+H$^+$]: 364.1377, found 364.1364.

***N*-(2-hydroxy-2,4-dimethylcyclobutyl)-4-methylbenzenesulfonamide 28b**

BN-Dewar benzene **28a** (30.3 mg, 0.0893 mmol) was used for the telescoped hydrogenation and oxidation step. Two batches were set up in parallel. In adaptation of the general procedure, the crude residue was purified by preparative thin layer chromatography (silica gel, 40% ethyl acetate in hexane) to give the desired product as a white solid with 54% average yield (Batch 1: 13.1 mg, 0.0486 mmol, 54%; Batch 2: 12.8 mg, 0.0475 mmol, 53%).

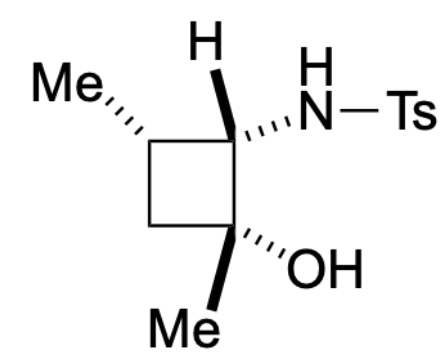

^{1}H NMR ($CDCl_3$, 500 MHz) δ 7.77 (d, J = 8.3 Hz, 2H), 7.31 (d, J = 8.3 Hz, 2H), 5.18 (d, J = 9.3 Hz, 1H), 3.62 (td, J = 8.0, 1.3 Hz, 1H), 2.43 (s, 3H), 2.02 (ddd, J = 12.6, 8.5, 1.5 Hz, 1H), 1.68–1.66 (br s, 1H), 1.50 (dd, J = 12.6, 1.1 Hz, 1H), 1.16 (s, 3H), 0.98 (d, J = 7.2 Hz, 3H). **^{13}C NMR (**$CDCl_3$, 126 MHz**)** δ 143.6, 137.9, 129.8, 127.3, 75.0, 57.0, 39.8, 28.6, 28.0, 21.7, 15.5; **IR** 3500, 3293, 2962, 2927, 1454, 1401, 1378, 1332, 1157, 1119, 1092, 1026, 669, 584 cm^{-1}; **HRMS** (DART) calcd. for $C_{13}H_{20}NO_3S$ [M+H$^+$]: 270.1158, found 270.1175.

***N*-(2-hydroxy-2,4-dipropylcyclobutyl)-4-methylbenzenesulfonamide 29b**

BN-Dewar benzene **29a** (42.6 mg, 0.108 mmol) was used for the telescoped hydrogenation and oxidation step. Two batches were set up in parallel. In adaptation of the general procedure, the crude residue was purified by preparative thin layer chromatography (silica gel, 40% ethyl acetate in hexane) to give the desired product as a white solid with 42% average yield (Batch 1: 14.7 mg, 0.0452 mmol, 42%; Batch 2: 15.1 mg, 0.0464 mmol, 43%).

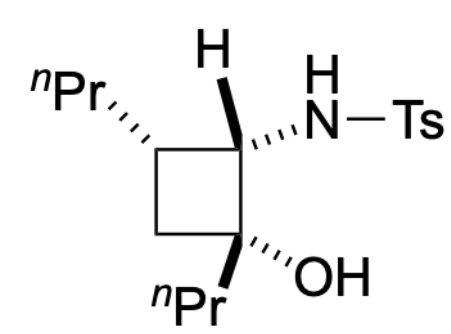

^{1}H NMR ($CDCl_3$, 500 MHz) δ 7.76 (d, J =8.0 Hz, 2H), 7.30 (d, J = 8.0 Hz, 2H), 5.17 (d, J = 9.3 Hz, 1H), 3.61 (t, J = 8.8 Hz, 1H), 2.42 (s, 3H), 2.21–2.14 (m, 1H), 2.03–1.97 (m, 1H), 1.65 (s, 1H), 1.47 (dd, J = 12.5, 6.3 Hz, 1H), 1.40–1.03 (m, 8H), 0.89–0.72 (m, 6H); **^{13}C NMR** ($CDCl_3$, 126 MHz) δ 143.6, 137.8, 129.7, 127.4, 76.4, 56.2, 42.8, 37.1, 33.6, 32.4, 21.6, 20.2, 16.5, 14.4, 14.2; **IR** 3483, 3303, 2958, 2869, 1739, 1598, 1455, 1366, 1304, 1264, 1228, 1159, 1093, 928, 813, 739, 706, 583, 549 cm^{-1}; **HRMS** (DART) calcd. for $C_{17}H_{28}NO_3S$ [M+H$^+$]: 326.1784, found 326.1796.

***N*-(4-benzyl-2-hydroxy-2-methylcyclobutyl)-4-methylbenzenesulfonamide 30b**

BN-Dewar benzene **30a** (45.4 mg, 0.109 mmol) was used for the telescoped hydrogenation and oxidation step. Two batches were set up in parallel. In adaptation of the general procedure (10 mol% of Pd/C, 16 hours condition was used for hydrogenation), the crude residue was purified by flash column chromatography (silica gel, 25 – 40% ethyl acetate in hexane) to give the desired product as white solid with 50% average yield (Batch 1: 19.5 mg, 0.0565 mmol, 52%; Batch 2: 18.3 mg, 0.0530 mmol, 49%).

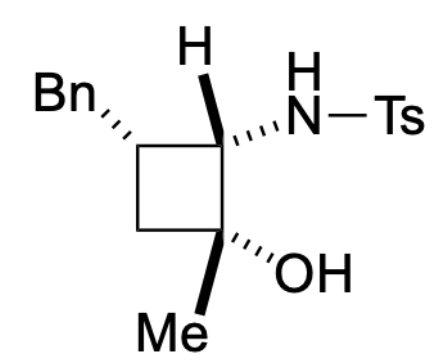

^{1}H NMR ($CDCl_3$, 500 MHz) δ 7.74 (d, *J* = 8.3 Hz, 2H), 7.33–7.28 (m, 2H), 7.25–7.19 (m, 2H), 7.18–7.14 (m, 1H), 7.03 (d, *J* = 8.3 Hz, 1H), 5.35 (d, *J* = 9.2 Hz, 1H), 3.70 (ddt, *J* = 9.9, 9.0, 1.4 Hz, 1H), 2.77–2.64 (m, 2H), 2.60–2.50 (m, 1H), 2.43 (s, 3H), 1.91–1.84 (m, 1H), 1.84 (s, 1H), 1.77–1.64 (m, 1H), 1.19 (s, 3H); **^{13}C NMR (**$CDCl_3$, 126 MHz**)** δ 143.7, 140.1, 137.6, 129.8, 128.8, 128.5, 127.3, 126.1, 74.1, 57.5, 38.1, 36.0, 35.0, 27.8, 21.7; **IR** 3493, 3287, 2963, 2925, 1495, 1400, 1303, 1090, 1019, 908, 814, 702, 665, 519 cm^{-1}; **HRMS** (DART) calcd. for $C_{19}H_{24}NO_3S$ [$M+H^+$]: 346.1471, found 346.1472.

***N*-(2-hydroxy-1-methyl-2-propylcyclobutyl)-4-methylbenzenesulfonamide 31b**

BN-Dewar benzene **31a** (43.0 mg, 0.133 mmol) was used for the telescoped hydrogenation and oxidation step. Two batches were set up in parallel. In adaptation of the general procedure, the crude residue was purified by flash column chromatography (silica gel, 25 – 40% ethyl acetate in hexane) to give the desired product as white solid with 59% average yield (Batch 1: 22.2 mg, 0.0747 mmol, 56%; Batch 2: 24.5 mg, 0.0825 mmol, 62%).

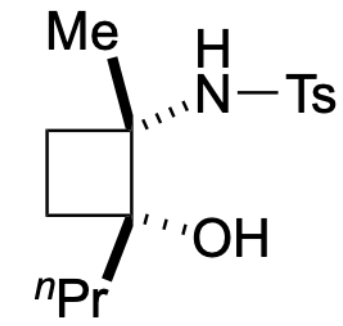

^{1}H NMR ($CDCl_3$, 500 MHz) δ 7.78 (d, J = 8.3 Hz, 2H), 7.28 (d, J = 8.0 Hz, 2H), 5.45 (s, 1H), 2.42 (s, 3H), 2.13–2.04 (m, 2H), 1.83–1.67 (m, 2H), 1.67–1.60 (m, 1H), 1.52–1.29 (m, 4H), 1.26 (s, 3H), 0.91 (t, J = 7.1 Hz, 3H); **^{13}C NMR** ($CDCl_3$, 126 MHz) δ 143.3, 139.9, 129.7, 127.3, 80.5, 61.1, 37.9, 31.6, 29.3, 21.7, 21.3, 16.4, 14.6; **IR** 3490, 3327, 2957, 2872, 1739, 1599, 1454, 1374, 1318, 1262, 1152, 1094, 942, 815, 737, 707, 587, 551 cm^{-1}; **HRMS** (DART) calcd. for $C_{15}H_{24}NO_3S$ [M+H$^+$]: 298.1471, found 298.1477.

Unsuccessful Substrates

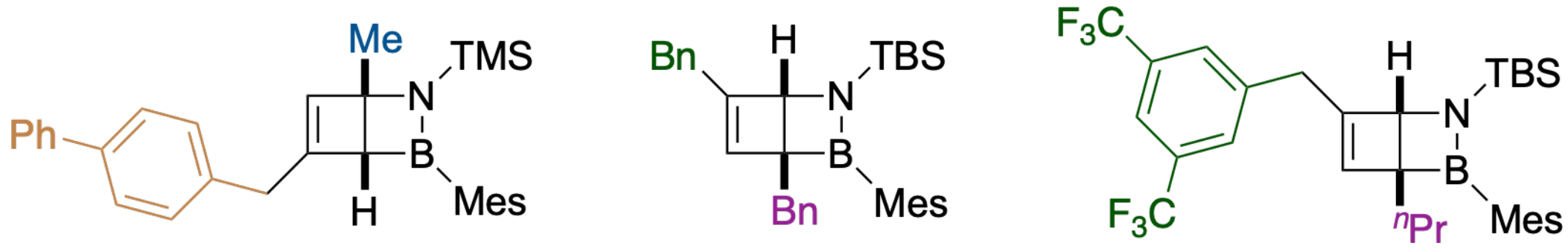

Figure S1. Unsuccessful substrates for the hydrogenation step

Due to steric hindrance, these substrates underwent only partial hydrogenation under the optimized conditions (Pd/C, 250 psi H_2).

Mesityl Group Migration

4-bromo-2-(tert-butyldimethylsilyl)-3-mesityl-2-aza-3-borabicyclo[2.2.0]hexane 36

In a glovebox, 10 wt. % Palladium on carbon (93.4 mg, 10 mol %, 0.077 mmol) and a cyclohexane solution (7.7 mL, 0.10 M) of the collected Dewar substrate **16a**, prepared by photoisomerization of C3-Br-1,2-azaborine **8** (300 mg, 0.769 mmol), were divided equally between two 20 mL scintillation vials, each equipped with a magnetic stir bar. The vials were sealed with septum screw cap, and a 18G x 1 1/2 needle (1.2 mm x 40 mm) was fitted on the septum screw cap for atmosphere exchange. The vials were then placed in a Parr pressure vessel. The pressure vessel was sealed and transferred into a fume hood with the safety blast shield placed. The pressure vessel was

pressurized, released and back-filled with hydrogen gas five times before charged with 500 psi hydrogen (caution: fix the hydrogen gas tank firmly during the whole process; apply hydrogen slowly through a pressure regulator; gently open the venting valve to release pressure). The pressure vessel was placed in the middle of stir plate and the reaction was allowed to vigorously stir for 15 hours. After 15 hours, the venting valve of pressure vessel was slowly opened to release H_2 carefully. The reaction mixture was filtered to remove the heterogenous catalyst and the filtrate was concentrated *in vacuo*. The crude mixture was then purified by silica gel column chromatography using hexane to obtain the product **36** as a colorless oil (173 mg, 0.441 mmol, 57%).

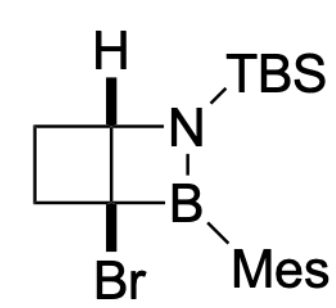

^{1}H NMR ($CDCl_3$, 600 MHz) δ 6.78 (s, 2H), 4.50 (d, J = 6.1 Hz, 1H), 2.78–2.67 (m, 2H), 2.58–2.49 (m, 1H), 2.42 (s, 6H), 2.26 (s, 3H), 2.10–2.01 (m, 1H), 0.89 (s, 6H), 0.03 (s, 3H), –0.14 (s, 3H). **^{13}C NMR** ($CDCl_3$, 126 MHz) δ 137.8, 127.0, 70.2, 29.9, 29.5, 26.4, 21.4, 18.3, –4.8, –5.4 (the signals for the two carbons adjacent to boron were not observed).; **^{11}B NMR** ($CDCl_3$, 160 MHz) δ 51.1.

2-hydroxy-2-mesitylcyclobutyl)-4-methylbenzenesulfonamide 38

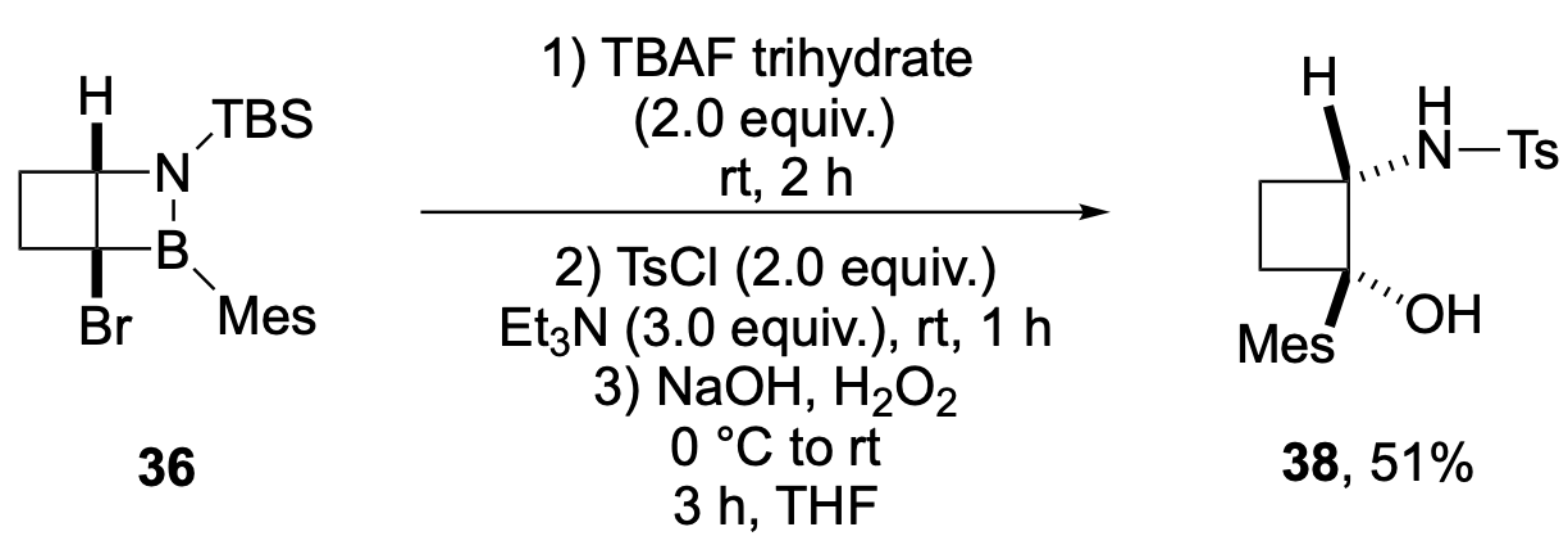

Hydrogenated BN-Dewar benzene **36** (39.2 mg, 0.0999 mmol) was used for the oxidation step following general procedure. The crude residue was purified by silica gel column chromatography

using 20–50% ethyl acetate in hexane to obtain the product **38** as a white solid (18.4 mg, 0.0512 mmol, 51%).

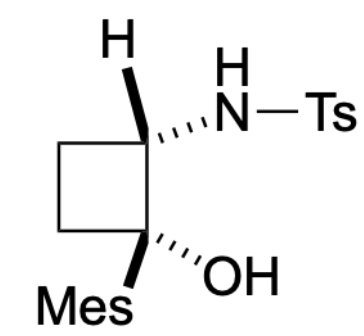

^{1}H NMR ($CDCl_3$, 500 MHz) δ 7.77 (d, *J* = 8.7 Hz, 2H), 7.30 (d, *J* = 7.9 Hz, 2H), 6.79 (s, 2H), 5.38 (br s, 1H), 4.39 (q, *J* = 8.8 Hz, 1H), 2.44 (s, 3H), 2.39 (s, 6H), 2.23 (s, 3H), 2.20–2.13 (m, 1H), 2.12–1.98 (m, 1H), 1.81 (q, *J* = 9.1 Hz, 1H), 1.61–1.50 (m, 1H).; **^{13}C NMR** ($CDCl_3$, 126 MHz) δ 143.7, 138.7, 138.1, 137.6, 137.0, 131.0, 129.8, 127.3, 84.0, 54.4, 34.1, 26.5, 22.4, 21.7, 20.6; **IR** 3272, 2923. 1702, 1610. 1598,. 1489, 1451, 1379, 1333, 1197, 1158, 1121, 1019, 941, 885, 815 cm^{-1}. **HRMS** (DART) calcd. for $C_{20}H_{24}NO_2S$ [M+H–H_2O]$^+$: 342.1522, found 342.1521.

2-Bpin-2-mesitylcyclobutyl)-4-methylbenzenesulfonamide 40

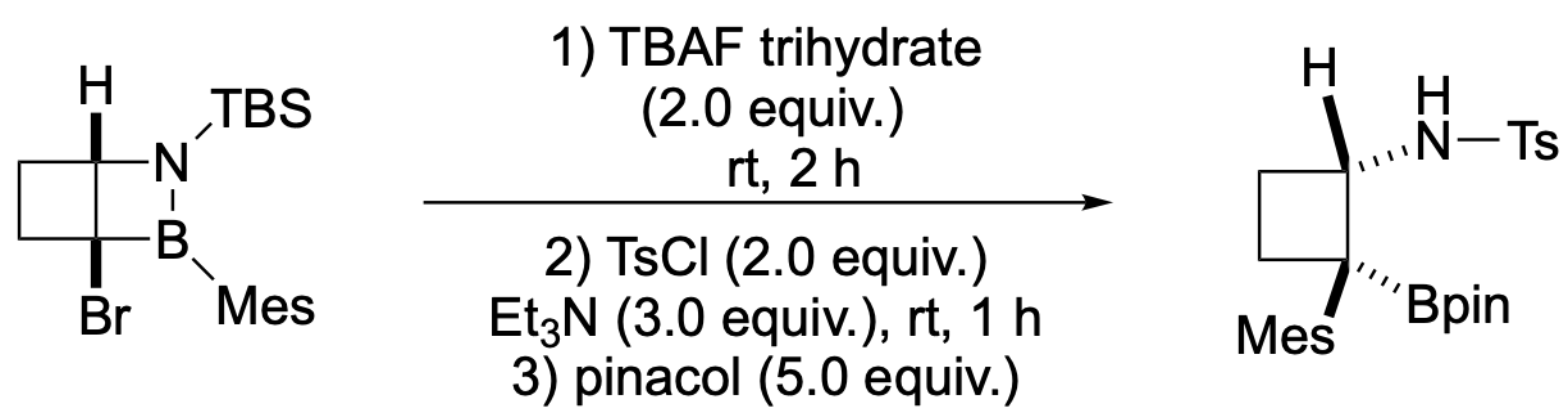

36 **40**, 43%

Hydrogenated BN-Dewar benzene **36** (39.2 mg, 0.0999 mmol) was used for the oxidation step following the general procedure. However, instead of acrodisc filtration followed by the NaOH/H_2O_2 oxidation steps, 5.0 equiv. of pinacol was directly added and the reaction mixture was allowed to stir for 3 hours at room temperature. At the conclusion of the reaction, the crude residue was concentrated and purified by silica gel column chromatography 10–20% ethyl acetate in hexane to obtain the product **40** as a colorless oil (20.2 mg, 0.043 mmol, 43%).

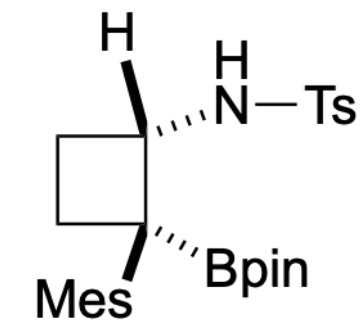

^{1}H NMR ($CDCl_3$, 500 MHz) δ 7.73 (d, J = 8.2 Hz, 2H), 7.28 (d, J = 8.2 Hz, 2H), 6.71 (s, 2H), 5.77 (d, J = 8.8 Hz, 1H), 3.99 (q, J = 8.5 Hz, 1H), 2.45 (t, J = 10.7 Hz, 1H), 2.42 (s, 3H), 2.19 (s, 3H), 2.15 (s, 6H), 1.91–1.75 (m, 2H), 1.66–1.55 (m, 1H), 1.26 (s, 6H), 1.25 (s, 6H).; **^{13}C NMR** ($CDCl_3$, 126 MHz) δ 143.4, 141.6, 138.2, 136.0, 134.3, 130.1, 129.7, 127.4, 84.3, 55.2, 28.6, 28.4, 24.80, 24.77, 22.8, 21.7, 20.6. (the signal for the carbon adjacent to boron were not observed); **^{11}B NMR** ($CDCl_3$, 160 MHz) δ 33.4.; **IR** 3280, 2977, 1708, 1417, 1362, 1317, 1247, 1222, 1150, 1091, 1011, 952, 859, 815 cm^{-1}; **HRMS** (DART) calcd. for $C_{26}H_{37}BNO_4S$ [M+H^{+}]: 470.2531, found 470.2525.

Product Derivatization

<u>Derivatizations of amine functionality of **41**</u>

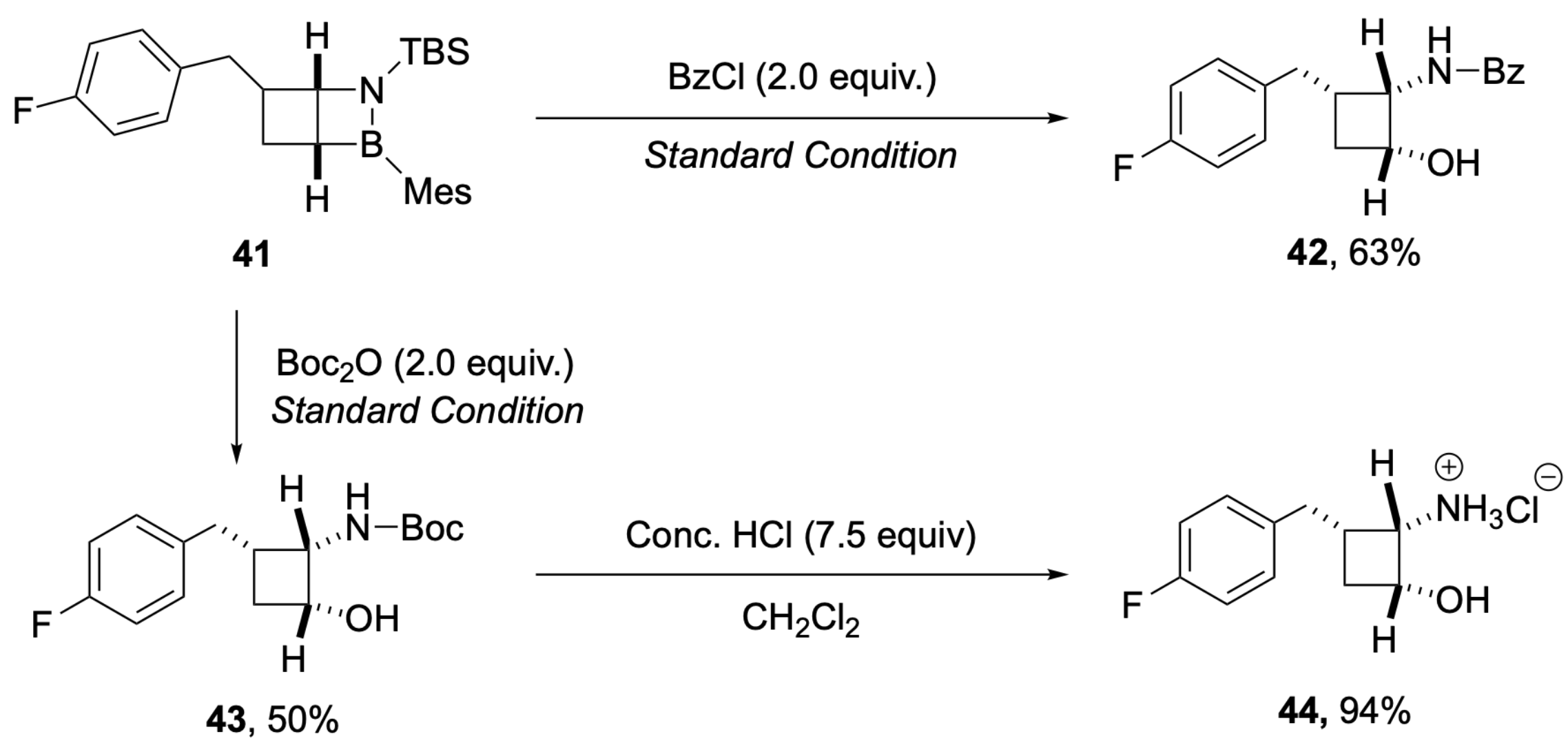

2-(*tert*-butyldimethylsilyl)-6-(4-fluorobenzyl)-3-mesityl-2-aza-3-borabicyclo[2.2.0]hexane 41

In a glovebox, a 20 mL scintillation vial was charged with magnetic stir bar, 10 wt. % Palladium on carbon (179.5 mg, 10% Pd purity, 10 mol %, 0.148 mmol) and cyclohexane solution (7.5 mL, 0.20 M) of collected Dewar substrate (620 mg, 1.48 mmol). The vial was sealed with septum screw

cap, and a 18G x 1 1/2 needle (1.2 mm x 40 mm) was fitted on the septum screw cap for atmosphere exchange. The vial was then placed in a Parr pressure vessel. The pressure vessel was sealed and transferred into a fume hood with the safety blast shield placed. The pressure vessel was pressurized, released and back-filled with hydrogen gas five times before charged with 500 psi hydrogen (caution: fix the hydrogen gas tank firmly during the whole process; apply hydrogen slowly through a pressure regulator; gently open the venting valve to release pressure). The pressure vessel was placed in the middle of stir plate and the reaction was allowed to vigorously stir for 12 hours. After 12 hours, the venting valve of pressure vessel was slowly opened to release H2 carefully. The Parr pressure vessel was purged with N2 for 5 minutes before brought into glovebox. The reaction mixture was filtered to remove the heterogenous catalyst and the filtrate was concentrated in vacuo under N2 atmosphere. The hydrogenated intermediate (471 mg, 1.12 mmol, 76%) was used directly for the subsequent oxidation step without further purification.

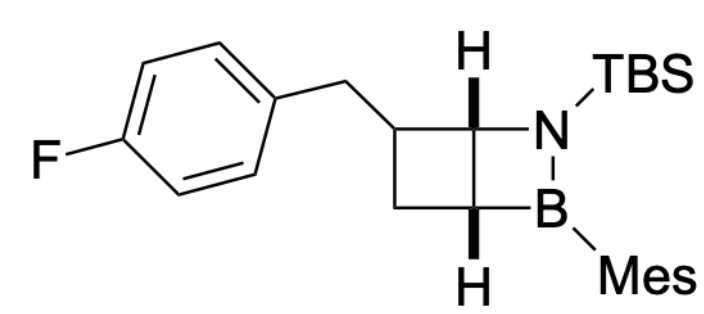

^{1}H NMR (THF–d_8, 500 MHz) δ 7.16 (dd, J = 8.5, 5.5 Hz, 2H), 6.96 (t, J = 8.8 Hz, 2H), 6.79–6.71 (br s, 2H), 4.70 (dd, J = 5.9, 3.9 Hz, 1H), 3.09–2.98 (m, 1H), 2.93–2.78 (m, 2H), 2.56 (br s, 3H), 2.40–2.34 (m, 2H), 2.21 (s, 6H), 2.04 (dt, J = 12.0, 10.4 Hz, 1H), 0.87 (s, 9H), 0.18 (s, 3H), –0.07 (s, 3H). **^{13}C NMR** (THF–d_8, 151 MHz)) δ 162.5 (d, $^{1}J_{CF}$ = 242.4 Hz), 138.1, 137.27 (d, $^{4}J_{CF}$ = 3.0 Hz), 131.04 (d, $^{3}J_{CF}$ = 7.6 Hz), 128.0, 115.81 (d, $^{2}J_{CF}$ = 21.3 Hz), 65.4, 41.6, 38.5, 27.2, 23.5, 21.5, 19.5, –3.3, –4.2 (the signals for the two carbons adjacent to boron were not observed); **^{11}B NMR** (THF-d_8, 160 MHz) 54.0; **^{19}F NMR** (THF-d_8, 470 MHz) δ –119.1 (m); **IR** 2985, 2956, 2923, 2858, 1745, 1609, 1509, 1445, 1352, 1273, 1217, 1193, 979, 845 cm^{-1}; **HRMS** (DART) calcd. for $C_{26}H_{37}BNFSi$ [M+H^{+}]: 422.1354, found 422.1362.

N-(2-(4-fluorobenzyl)-4-hydroxycyclobutyl)benzamide 42

General procedure for oxidation step was applied except for the addition of 2.0 equiv. benzoyl chloride (BzCl) instead of tosyl chloride (TsCl). The yield was reported based on the one-pot oxidation step. The crude residue was purified by preparative thin layer chromatography (silica gel, 50% ethyl acetate in hexane) to give the desired product **42** as a white solid (11.3 mg, 0.0378 mmol, 63%).

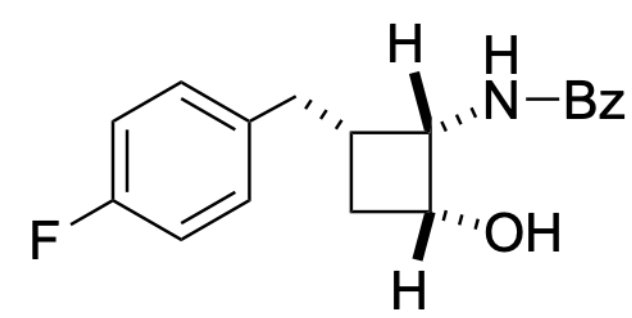

^{1}H NMR ($CDCl_3$, 600 MHz) δ 7.80 (d, J = 7.6 Hz, 2H), 7.52 (t, J = 7.4 Hz, 1H), 7.45 (t, J = 7.6 Hz, 2H), 7.09 (dd, J = 8.4, 5.6 Hz, 2H), 6.98 (d, J = 6.7 Hz, 1H), 6.93 (t, J = 8.7 Hz, 2H), 4.72 (dt, J = 7.5, 3.8 Hz, 1H), 4.57 (q, J = 6.5 Hz, 1H), 2.95 (dd, J = 11.5, 8.0 Hz, 1H), 2.69–2.59 (m, 2H), 2.45–2.33 (m, 1H), 1.78 (dt, J = 12.8, 6.4 Hz, 1H), 1.66–1.55 (br s, 1H); **^{13}C NMR** ($CDCl_3$, 151 MHz) δ 168.2, 161.5 (d, $^{1}J_{CF}$ = 243.5 Hz), 135.67 (d,$^{4}J_{CF}$ = 3.2 Hz), 134.5, 131.8, 130.02 (d, $^{3}J_{CF}$ = 8.0 Hz), 128.8, 127.1 ,115.28 (d, $^{2}J_{CF}$ = 21.2 Hz), 65.8, 52.2, 35.9, 35.7, 35.1; **^{19}F NMR** ($CDCl_3$, 470 MHz) δ –117.6 (m); **IR** 3391, 3353, 2963, 2929, 1789, 1735, 1637, 1603, 1509, 1278, 1157, 1088, 935, 815, 740, 575, 555 cm^{-1}; **HRMS** (DART) calcd. for $C_{18}H_{19}NO_2F$ [$M+H^+$]: 300.1394, found 300.1393.

tert-butyl (2-(4-fluorobenzyl)-4-hydroxycyclobutyl)carbamate 43

General procedure for oxidation step was applied except for the addition of 2.0 equiv. Di-tert-butyl dicarbonate (Boc_2O) instead of tosyl chloride (TsCl). The yield was reported based on the one-pot oxidation step. The crude residue was purified by column chlomatography (silica gel, 30% ethyl acetate in hexane) to give the desired product **43** as a white solid (166 mg, 0.562 mmol, 50%).

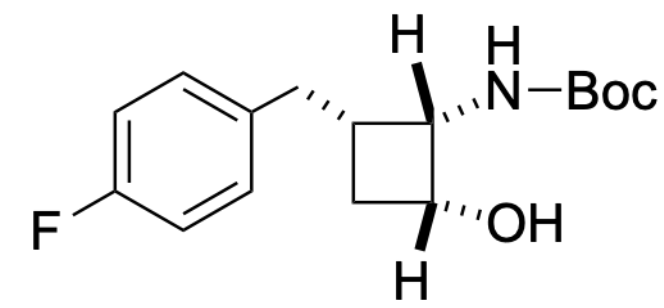

^{1}H NMR ($CDCl_3$, 500 MHz) δ 7.13–7.06 (m, 2H), 6.98–6.91 (m, 2H), 5.28 (s, 1H), 4.43 (s, 1H), 4.30 (s, 1H), 2.87 (dd, J = 14.0, 5.9 Hz, 1H), 2.62 (dd, J = 14.0, 10.1 Hz, 1H), 2.55–2.44 (m, 1H), 2.33–2.24 (m, 1H), 2.24–2.11 (m, 1H), 1.70–1.62 (m, 1H), 1.46 (s, 9H). **^{13}C NMR** ($CDCl_3$, 126 MHz) δ 161.5, (d, $^1J_{CF}$ = 210.4 Hz), 156.5, 135.93 (d, $^4J_{CF}$ = 3.4 Hz), 130.02 (d, $^3J_{CF}$ = 7.6 Hz), 115.20 (d, $^2J_{CF}$ = 20.2 Hz), 79.7, 65.9, 52.6, 35.8, 35.4, 34.8, 28.5. **^{19}F NMR** ($CDCl_3$, 470 MHz) δ –117.7. **IR** 3413, 2976, 2933, 1686, 1509, 1366, 1061, 1028, 848 cm^{-1}; **HRMS** (DART) calcd. for $C_{16}H_{23}NO_3F$ [M+H$^+$]: 296.1657, found 296.1655.

2-(4-fluorobenzyl)-4-hydroxycyclobutan-1-ammonium chloride 44

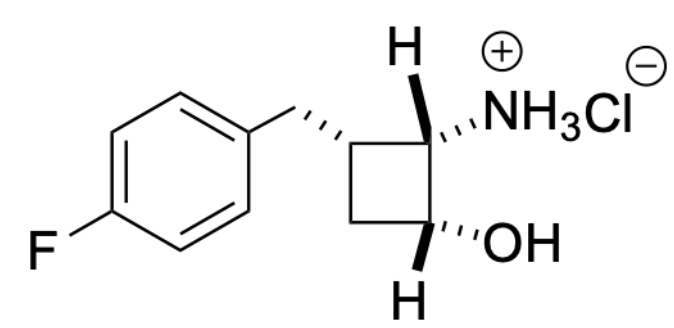

A 20 ml vial was charged with a magnetic stir bar, **43** (29.5 mg, 0.100 mol, 1.0 equiv.) and 1.0 mL CH_2Cl_2. Concentrated HCl (0.20 mL, 37% purity in H_2O, 7.5 equiv.) were added to the stirred solution and the reaction mixture was stirred for 1 hour at room temperature. At the conclusion of the reaction, the crude mixture was concentrated in vacuo. The solid was washed with hexane and ethyl acetate. The product **44** was isolated as a white solid (21.7 mg, 0.0940 mmol, 94%).**^{1}H NMR** (DMSO-d_6, 500 MHz) δ 8.33 (s, 3H), 7.21–7.12 (m, 2H), 7.12–7.06 (m, 2H), 5.74 (t, J = 6.3 Hz, 1H), 4.25–4.17 (m, 1H), 3.71 (s, 1H), 2.83 (dd, J = 13.6, 4.7 Hz, 1H), 2.70 (dd, J = 13.6, 11.6 Hz, 1H), 2.41–2.31 (m, 1H), 2.13–1.95 (m, 2H); ^{13}C NMR (DMSO-d_6, 126 MHz) δ 160.7 (d, $^1J_{CF}$ = 241.9 Hz), 135.6 (d, $^4J_{CF}$ = 3.0 Hz), 130.1 (d, $^3J_{CF}$ = 7.6 Hz), 115.1 (d, $^2J_{CF}$ = 20.2 Hz), 61.3, 53.4, 34.5, 33.3, 30.7; **^{19}F NMR** (DMSO-d_6, 470 MHz) δ –117.3. **IR** 3342, 3002, 2937, 1600, 1510, 1295, 1226, 1156, 1118, 1066, 1028, 841, 767, 674, 568, 519 cm^{-1}; **HRMS** (DART) calcd. for $C_{11}H_{15}NOF$ [M–Cl$^-$]: 196.1132, found 196.1136.

Derivatizations of products **24b**

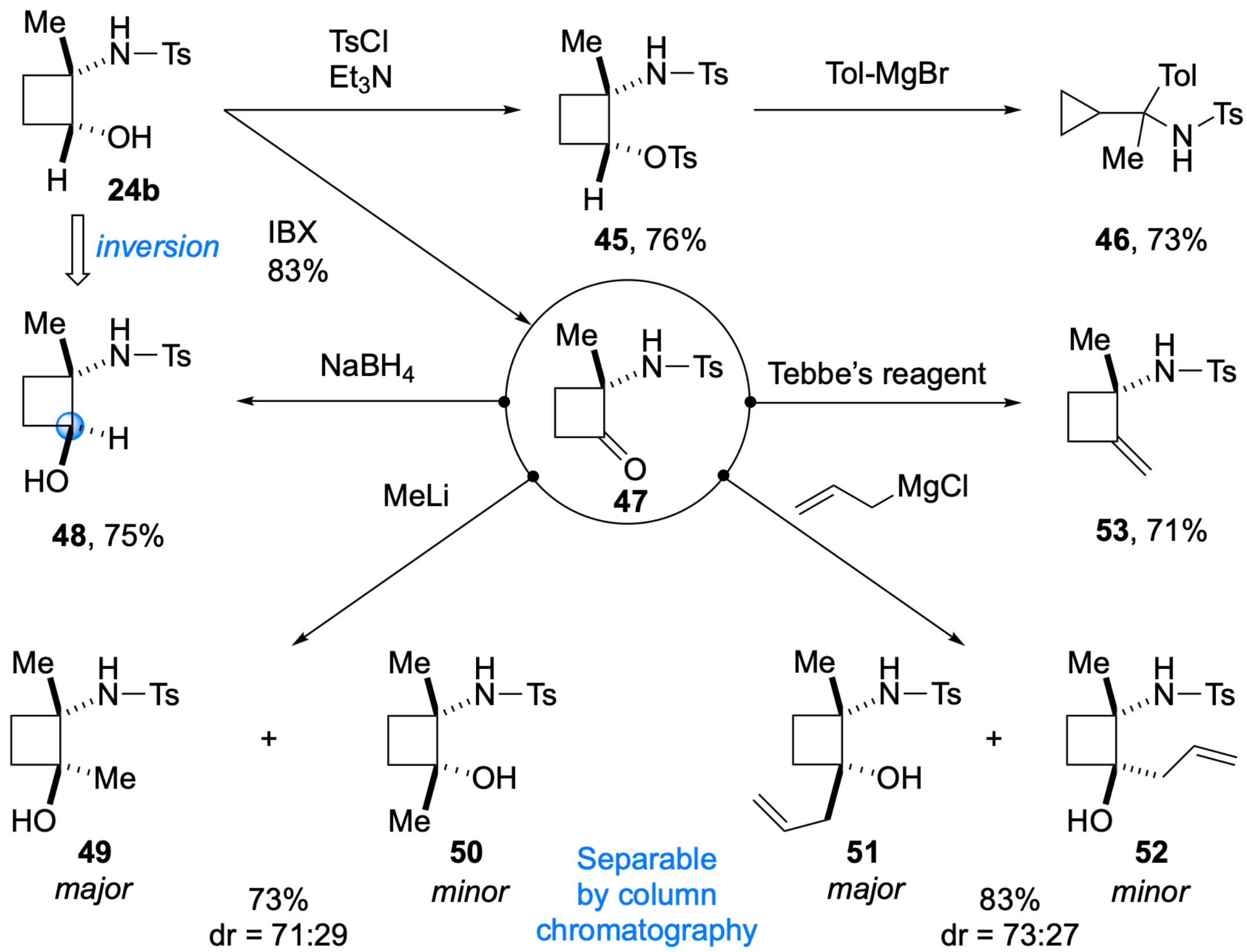

Procedure for 45

In a glovebox, a 20 ml vial was charged with a magnetic stir bar, **24b** (51.1 mg, 0.200 mmol, 1.0 equiv.) and 2.0 mL dichloromethane. Tosyl chloride (76.3 mg, 0.400 mmol, 2.0 equiv.) and triethylamine (81 mg, 0.80 mmol, 4.0 equiv.) were added to the stirred solution and the reaction miture was allowed to stir for 48 hours at room temperature. At the conclusion of the reaction, the crude mixture was concentrated *in vacuo*. The crude residue was purified by flash column chromatography using 30% ethyl acetate in hexane to give the desired product **45** as a colorless oil (62.1 mg, 0.152 mmol, 76%).

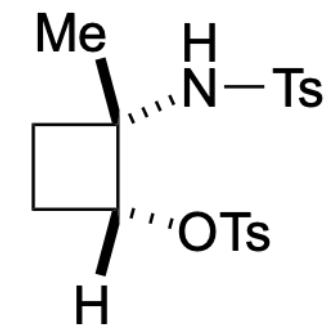

^{1}H NMR ($CDCl_3$, 500 MHz) δ 7.78 (d, *J* = 8.2 Hz, 2H), 7.76 (d, *J* = 8.1 Hz, 2H), 7.35 (d, *J* = 8.1 Hz, 2H), 7.28 (d, *J* = 8.2 Hz, 2H), 5.05 (s, 1H), 4.41 (t, *J* = 7.5 Hz, 1H), 2.46 (s, 3H), 2.41 (s, 3H), 2.40–2.33 (m, 1H), 2.06–1.95 (m, 2H), 1.52–1.44 (m, 1H), 1.27 (s, 3H); **^{13}C NMR** ($CDCl_3$, 126 MHz) δ 145.6, 143.4, 140.1, 133.1, 130.2, 129.8, 128.1, 126.9, 78.6, 62.2, 26.3, 26.2, 25.3, 21.8, 21.7; **IR** 3289, 2965, 1598, 1358, 1245, 1226, 1190, 1177, 1155, 1094, 1036, 1020, 979, 953, 900, 707, 554 cm^{-1}; **HRMS** (DART) calcd. for $C_{19}H_{24}NO_5S_2$ [M+H$^+$]: 410.1090, found 410.1103.

<u>Procedure for **46**</u>

In a glovebox, a 20 ml vial was charged with a magnetic stir bar, **45** (41.0 mg, 0.100 mmol, 1.0 equiv.) and 1.0 mL THF. *p*-Tolyl magnesium bromide (0.50 mL, 0.50 mmol, 5.0 equiv., 1.0 M in THF) was added to the stirred solution and the reaction mixture was allowed to stir for 2 hours at room temperature. At the conclusion of the reaction, water was added into the reaction mixture and the aqueous phase was extracted with ethyl acetate. The combined organic extracts were washed with brine, dried over $MgSO_4$, vacuum-filtered through a fritted funnel, and concentrated *in vacuo.* The crude residue was purified by flash column chromatography using 20% ethyl acetate in hexane to give the desired product **46** as a white solid (24.1 mg, 0.0732 mmol, 73%).

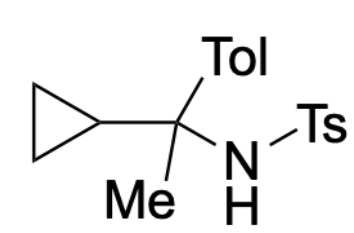

^{1}H NMR ($CDCl_3$, 600 MHz) δ 7.63 (d, *J* = 8.3 Hz, 2H), 7.23 (d, *J* = 8.3 Hz, 2H), 7.18 (d, *J* = 8.0 Hz, 2H), 7.01 (d, *J* = 8.0 Hz, 2H), 5.05 (s, 1H), 2.40 (s, 3H), 2.30 (s, 3H), 1.51 (s, 3H), 1.34–1.28 (m, 1H), 0.40–0.28 (m, 2H), 0.28–0.16 (m, 2H); **^{13}C NMR** ($CDCl_3$, 126 MHz) δ 142.7, 140.9, 140.4, 136.8, 129.3, 128.7, 127.2, 126.5, 61.8, 24.3, 22.7, 21.6, 21.0, 2.3, 2.1; **IR** 3274, 3005, 2922, 1361, 1322, 1184, 1157, 1109, 1021, 814, 664, 565 cm^{-1}; **HRMS** (DART) calcd. for $C_{19}H_{24}NO_2S$ [M+H$^+$]: 330.1522, found 330.1532.

Procedure for **47**

In a glove box, a 15 mL pressure vessel was charged with a magnetic stir bar, **24b** (266 mg, 1.04 mmol, 1.0 equiv.), 2-iodoxybenzoic acid (IBX) (778 mg, 1.25 mmol, 1.2 equiv., 45% wt%, stabilized with benzoic acid and isophthalic acid), and 5.0 mL ethyl acetate. The reaction mixture was sealed and allowed to stir at 80 ºC in an oil bath for 2.0 hours. At the conclusion of the reaction, the reaction mixture was cooled down to room temperature, filtered through a fritted funnel, and concentrated *in vacuo.* The crude residue was purified by flash column chromatography using 40% ethyl acetate in hexane to give the desired product **46** as a white solid (220 mg, 0.868 mmol, 83%).

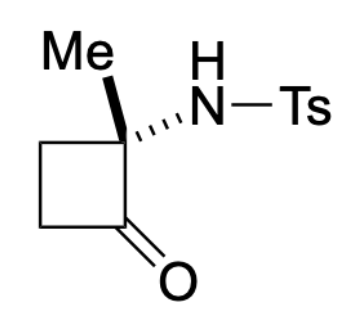

^{1}H NMR ($CDCl_3$, 500 MHz) δ 7.77 (d, *J* = 8.4 Hz, 2H), 7.30 (d, *J* = 8.4 Hz, 2H), 5.12 (s, 1H), 3.02–2.92 (m, 2H), 2.57 (q, *J* = 10.2 Hz, 1H), 2.42 (s, 3H), 2.06–1.97 (m, 1H), 1.39 (s, 3H); **^{13}C NMR** ($CDCl_3$, 126 MHz) δ 207.4, 143.7, 139.3, 129.8, 127.0, 73.5, 41.2, 27.0, 22.6, 21.7; **IR** 3273, 1792, 1323, 1273, 1208, 1186, 1153, 1094, 1061, 941, 884, 706, 668 cm^{-1}; **HRMS** (DART) calcd. for $C_{12}H_{16}NO_3S$ [M+H^{+}]: 254.0845, found 254.0854.

Procedure for **48**

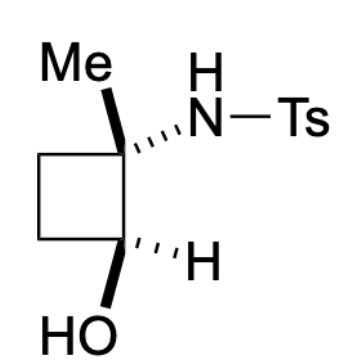

In a glovebox, a 20 ml vial was charged with a magnetic stir bar, **47** (25.3 mg, 0.100 mmol, 1.0 equiv.) and 1.0 mL THF. $NaBH_4$ (5.7 mg, 0.15 mmol, 1.5 equiv.) was then added to the stirred solution and the reaction mixture was allowed to stir for 2 hours at room temperature. At the conclusion of the reaction, water was added into the reaction mixture and the aqueous phase was extracted with ethyl acetate. The combined organic extracts were washed with brine, dried over $MgSO_4$, vacuum-filtered through a fritted funnel, and concentrated *in vacuo.* The crude residue was purified by flash column chromatography using 25–

50% ethyl acetate in hexane to give the desired product **48** as a white solid (19.1 mg, 0.0748 mmol, 75%).

^{1}H NMR ($CDCl_3$, 500 MHz) δ 7.76 (d, *J* = 8.0 Hz, 2H), 7.30 (d, *J* = 8.0 Hz, 2H), 4.79 (s, 1H), 4.35 (t, *J* = 7.4 Hz, 1H), 2.53 (s, 1H), 2.43 (s, 3H), 2.17–2.12 (m, 1H), 1.72–1.56 (m, 3H), 1.21 (s, 3H). ^{13}C NMR ($CDCl_3$, 126 MHz) δ 143.5, 139.9, 129.8, 126.9, 73.8, 61.4, 27.9, 24.6, 21.7, 19.1. **IR** 3266 2981, 2952, 1381, 1317, 1304, 1184, 1149, 1089, 1021, 877, 815, 707, 663, 549 cm^{-1}; **HRMS** (DART) calcd. for $C_{12}H_{18}NO_3S$ [M+H^{+}]: 256.1002, found 256.1015.

<u>Procedure for **49** and **50**</u>

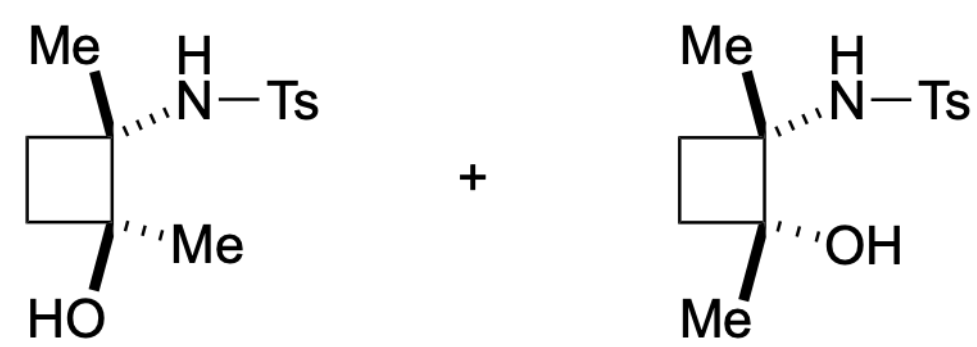

49 *major*   73% dr = 71:29   **50** *minor*

In a glovebox, a 20 ml vial was charged with a magnetic stir bar, **47** (25.3 mg, 0.100 mmol, 1.0 equiv.) and 1.0 mL diethyl ether. MeLi (0.16 mL, 0.50 mmol, 5.0 equiv., 3.1 M in diethoxymethane) was then added to the stirred solution and the reaction mixture was allowed to stir for 2 hours at room temperature. At the conclusion of the reaction, water was added into the reaction mixture and the aqueous phase was extracted with ethyl acetate. The combined organic extracts were washed with brine, dried over $MgSO_4$, vacuum-filtered through a fritted funnel, and concentrated *in vacuo.* The crude residue was purified by flash column chromatography using 40% ethyl acetate in hexane to give the mixture of desired products **49** and **50** as colorless oil (19.7 mg, 0.0731 mmol, 73%, dr = 71:29). The second column chrotomatography was conducted to isolate both diastereomers for characterization purpose.

***N*-(2-hydroxy-1,2-dimethylcyclobutyl)-4-methylbenzenesulfonamide 49 (*trans*-amino-alcohol)**

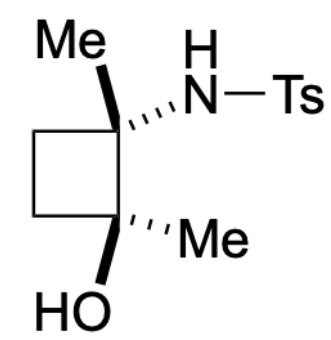

^{1}H NMR ($CDCl_3$, 500 MHz) δ 7.76 (d, *J* = 8.4 Hz, 2H), 7.28 (d, *J* = 8.4 Hz, 2H), 4.94–4.78 (m, 1H), 2.42 (s, 3H), 2.13 (s, 1H), 1.89–1.75 (m, 2H), 1.68–1.57 (m, 2H), 1.48 (s, 3H), 1.25 (s, 3H). **^{13}C NMR** ($CDCl_3$, 126 MHz) δ 143.3, 140.1, 129.7, 127.0, 76.5, 62.6, 30.5, 28.2, 24.4, 21.7, 20.4. **IR** 3489, 3274, 2977, 1454, 1406, 1376, 1304, 1247, 1193, 1150, 1121, 1091, 1012, 706, 664, 552 cm^{-1}; **HRMS** (DART) calcd. for $C_{13}H_{20}NO_3S$ [M+H$^+$]: 270.1158, found 270.1160.

***N*-(2-hydroxy-1,2-dimethylcyclobutyl)-4-methylbenzenesulfonamide 50 (*cis*-amino-alcohol)**

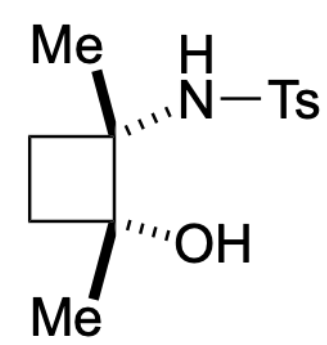

^{1}H NMR: ($CDCl_3$, 500 MHz) δ 7.79 (d, *J* = 8.3 Hz, 2H), 7.29 (d, *J* = 8.3 Hz, 2H), 5.32 (s, 1H), 2.42 (s, 3H), 2.13–2.06 (m, 2H), 1.86–1.74 (m, 2H), 1.67–1.60 (m, 1H), 1.24 (s, 6H). **^{13}C NMR** ($CDCl_3$, 126 MHz) δ 143.4, 139.9, 129.7, 127.2, 77.69, 61.4, 31.0, 30.9, 23.2, 21.7, 21.6. **IR** 3477, 3318, 2978, 2946, 1446, 1378, 1317, 1151, 664, 583 cm^{-1}; **HRMS** (DART) calcd. for $C_{13}H_{20}NO_3S$ [M+H$^+$]: 270.1158, found 270.1166.

Procedure for **51** and **52**

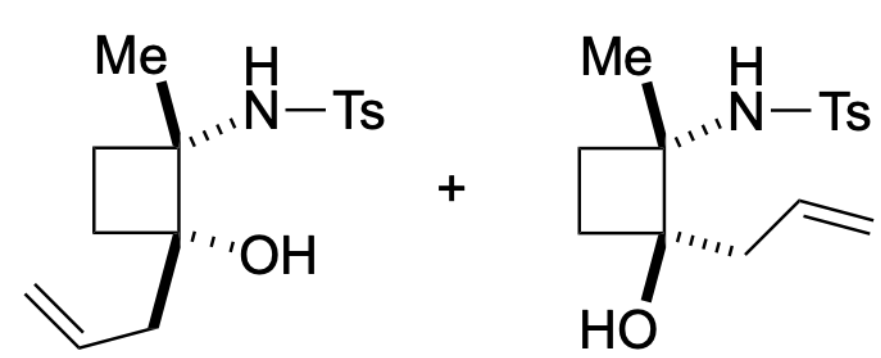

In a glovebox, a 20 ml vial was charged with a magnetic stir bar, **47** (25.3 mg, 0.100 mmol, 1.0 equiv.) and 1.0 mL THF. Allylmagnesium chloride (0.29 mL, 0.50 mmol, 5.0 equiv., 1.7 M in THF) was then added to the stirred solution and the reaction mixture was allowed to stir for 2 hours at room temperature. At the conclusion of the reaction, water was added into the reaction mixture and the aqueous phase was extracted with ethyl acetate. The combined organic extracts were washed with brine, dried over $MgSO_4$, vacuum-

filtered through a fritted funnel, and concentrated *in vacuo.* The crude residue was purified by flash column chromatography using 40% ethyl acetate in hexane to give the mixture of desired products **51** and **52** as colorless oil (24.6 mg, 0.0833 mmol, 83%, dr = 73:27). The second column chromatography was conducted to isolate both diastereomers for characterization purpose.

***N*-(2-allyl-2-hydroxy-1-methylcyclobutyl)-4-methylbenzenesulfonamide 51 (*cis*-amino-alcohol)**

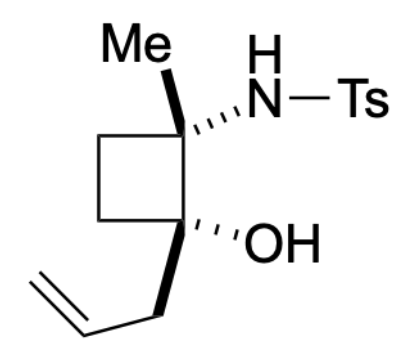

^{1}H NMR ($CDCl_3$, 500 MHz) δ 7.78 (d, *J* = 8.2 Hz, 2H), 7.28 (d, *J* = 8.2 Hz, 2H), 5.82–5.70 (m, 1H), 5.54 (s, 1H), 5.17 (d, *J* = 10.1 Hz, 1H), 5.12 (d, *J* = 17.1 Hz, 1H), 2.42 (s, 3H), 2.34 (s, 1H), 2.25 (d, *J* = 7.3 Hz, 2H), 2.09 (dd, *J* = 20.2, 9.7 Hz, 1H), 1.84–1.69 (m, 2H), 1.67–1.60 (m, 1H), 1.32 (s, 3H). **^{13}C NMR** ($CDCl_3$, 126 MHz) δ 143.3, 139.9, 132.4, 129.7, 127.3, 119.9, 78.8, 61.0, 40.6, 31.3, 29.3, 21.7, 21.4. **IR** 3489, 3312, 2979, 2947, 1433, 1374, 1150, 1118, 1093, 665, 553 cm^{-1}; **HRMS** (DART) calcd. for $C_{15}H_{22}NO_3S$ [M+H$^+$]: 296.1315, found 296.1327.

***N*-(2-allyl-2-hydroxy-1-methylcyclobutyl)-4-methylbenzenesulfonamide 52 (*trans*-amino-alcohol)**

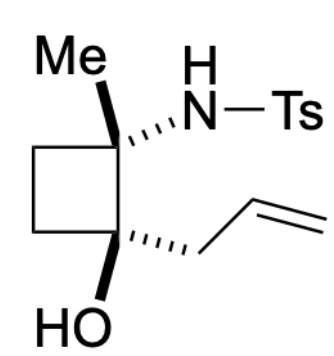

^{1}H NMR ($CDCl_3$, 500 MHz) 7.76 (d, *J* = 8.2 Hz, 2H), 7.29 (d, *J* = 8.2 Hz, 2H), 6.00–5.91 (m, 1H), 5.25–5.22 (m, 1H), 5.22–5.19 (m, 1H), 4.69 (s, 1H), 2.61 (ddd, *J* = 14.2, 6.7, 1.3 Hz, 1H), 2.46 (dt, *J* = 7.6, 1.3 Hz, 1H), 2.43 (s, 3H), 2.03 (s, 1H), 1.97–1.90 (m, 1H), 1.77–1.63 (m, 3H), 1.28 (s, 3H). ^{13}C NMR ($CDCl_3$, 126 MHz) δ 143.4, 140.0, 133.6, 129.7, 127.1, 119.5, 77.7, 62.8, 40.9, 28.1, 27.6, 21.7, 21.1. **IR** 3486, 3273, 2979, 2953, 1452, 1416, 1317, 1151, 1093, 946, 917, 664, 552 cm^{-1}; **HRMS** (DART) calcd. for $C_{15}H_{22}NO_3S$ [M+H$^+$]: 296.1315, found 296.1322.

Procedure for **53**

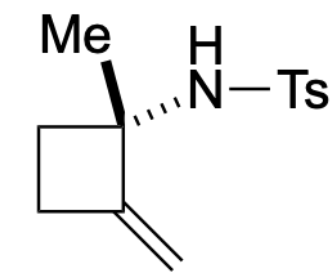

In a glovebox, a 20 ml vial was charged with a magnetic stir bar, **47** (25.3 mg, 0.100 mmol, 1.0 equiv.) and 2.0 mL THF. Tebbe reagent (57 mg, 0.20 mmol, 2.0 equiv.) was then added to the stirred solution and the reaction mixture was allowed to stir for 1 hour at room temperature. At the conclusion of the reaction, water was added into the reaction mixture and the aqueous phase was extracted with ethyl acetate. The combined organic extracts were washed with brine, dried over $MgSO_4$, vacuum-filtered through a fritted funnel, and concentrated *in vacuo.* The crude residue was purified by flash column chromatography using 25% ethyl acetate in hexane to give the desired product **53** as colorless oil (17.8 mg, 0.0708 mmol, 71%).

^{1}H NMR ($CDCl_3$, 500 MHz) δ 7.77 (d, J = 8.2 Hz, 2H), 7.28 (d, J = 8.2 Hz, 2H), 4.92 (td, J = 2.4, 0.9 Hz, 1H), 4.81 (s, 1H), 4.76 (td, J = 2.4, 0.9 Hz, 1H), 2.51–2.44 (m, 2H), 2.42 (s, 3H), 2.18–2.10 (m, 1H), 1.93–1.85 (m, 1H), 1.43 (s, 3H). **^{13}C NMR** ($CDCl_3$, 126 MHz) δ 154.6, 143.3, 139.9, 129.7, 127.3, 105.6, 62.1, 33.5, 25.7, 25.3, 21.7. **IR** 3268, 2971, 2927, 1599, 1373, 1185, 1121, 1094, 1021, 665, 577, 551 cm^{-1}; **HRMS** (DART) calcd. for $C_{13}H_{18}NO_2S$ [$M+H^+$]: 252.1053, found 252.1060.

Separation of *cis*- and *trans*-aminoalcols **49/50** and **51/52**.

To demonstrate that mixtures of **49/50** and **51/52** can be readily separated by silica gel column chromatography, the R_f value of *cis*-amino alcohol **29b** and *trans*-aminoalcohol **48** were measured on the same TLC plate. As shown in the Figure S2, **48** exhibited an R_f value of 0.49 while **24b** exhibited an R_f value of 0.33 using a 1:1 hexane : ethyl acetate eluent. A similar difference in R_f values was observed for mixtures of **49/50** and **51/52**, indicating that these products can be readily separated by silica gel column chromatography.

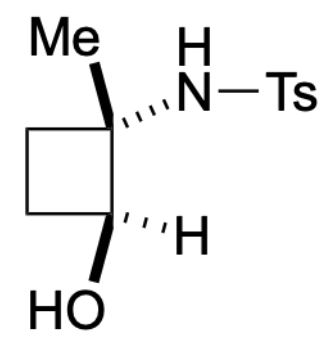

48

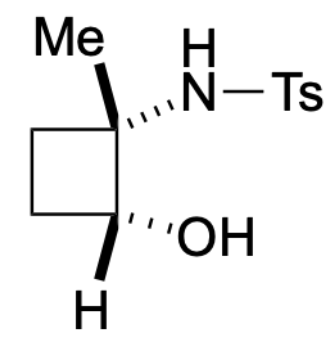

24b

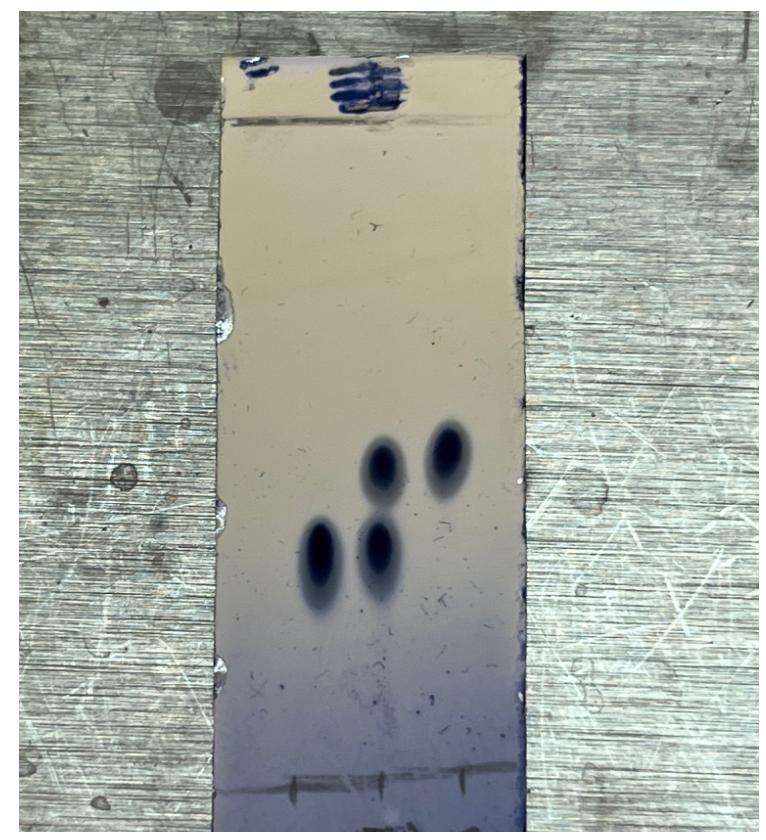

Figure S2. TLC analysis of **48** and **24b**

X-ray Crystallographic Data

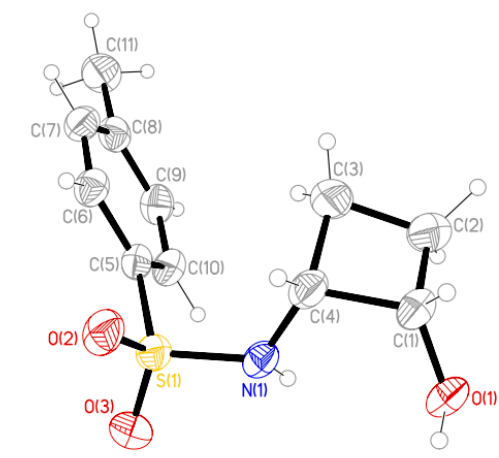

<u>Crystal data and structure refinement for **5**</u>

Identification code	C11H15NO3S	
Empirical formula	C11H15NO3S	
Formula weight	241.30	
Temperature	100(2) K	
Wavelength	0.71073 Å	
Crystal system	Monoclinic	
Space group	$P2_1/n$	
Unit cell dimensions	a = 6.5365(8) Å	α = 90°
	b = 7.4158(11) Å	β = 94.914(4)°
	c = 24.246(4) Å	γ = 90°
Volume	1171.0(3) $Å^3$	
Z	4	
Density (calculated)	1.369 Mg/m^3	
Absorption coefficient	0.268 mm^{-1}	
F(000)	512	
Crystal size	0.360 x 0.220 x 0.160 mm^3	
Theta range for data collection	1.686 to 28.303°.	
Index ranges	-8<=h<=7, -9<=k<=9, -32<=l<=32	
Reflections collected	29045	
Independent reflections	2894 [R(int) = 0.0461]	
Completeness to theta = 25.242°	99.9 %	
Absorption correction	Semi-empirical from equivalents	
Max. and min. transmission	0.7457 and 0.7007	
Refinement method	Full-matrix least-squares on F^2	
Data / restraints / parameters	2894 / 2 / 154	
Goodness-of-fit on F^2	1.043	
Final R indices [I>2sigma(I)]	R1 = 0.0372, wR2 = 0.0925	
R indices (all data)	R1 = 0.0517, wR2 = 0.1031	
Extinction coefficient	n/a	
Largest diff. peak and hole	0.361 and -0.327 e. $Å^{-3}$	

Crystal data and structure refinement for **20b**

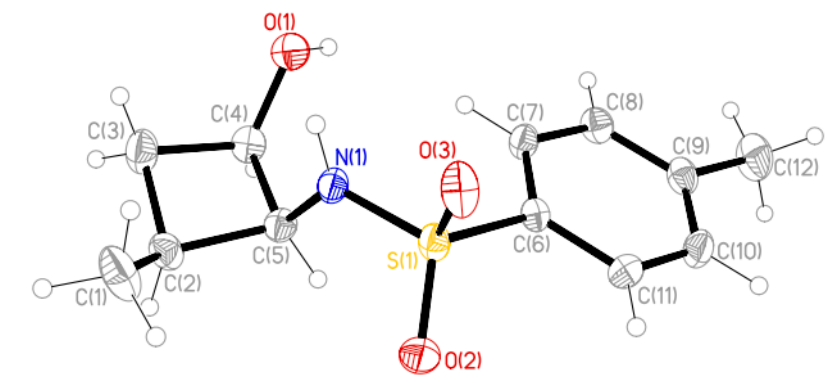

Identification code	C12H17NO3S	
Empirical formula	C12H17NO3S	
Formula weight	255.32	
aature	173(2) K	
Wavelength	0.71073 Å	
Crystal system	Monoclinic	
Space group	$P2_1/c$	
Unit cell dimensions	a = 6.4232(2) Å	α = 90º
	b = 24.0930(7) Å	β = 96.5920(10)º
	c = 8.1596(2) Å	γ = 90º
Volume	1254.38(6) $Å^3$	
Z	4	
Density (calculated)	1.352 Mg/m^3	
Absorption coefficient	0.254 mm^{-1}	
F(000)	544	
Crystal size	0.320 x 0.220 x 0.130 mm^3	
Theta range for data collection	2.651 to 28.298º.	
Index ranges	-8<=h<=8, -31<=k<=32, -10<=l<=10	
Reflections collected	14930	
Independent reflections	3120 [R(int) = 0.0436]	
Completeness to theta = 25.242º	100.0 %	
Absorption correction	Semi-empirical from equivalents	
Max. and min. transmission	0.7457 and 0.6973	
Refinement method	Full-matrix least-squares on F^2	
Data / restraints / parameters	3120 / 2 / 162	
Goodness-of-fit on F^2	1.057	
Final R indices [I>2sigma(I)]	R1 = 0.0393, wR2 = 0.0922	
R indices (all data)	R1 = 0.0520, wR2 = 0.0998	
Extinction coefficient	n/a	
Largest diff. peak and hole	0.359 and -0.363 e. $Å^{-3}$	

Crystal data and structure refinement for **24b**

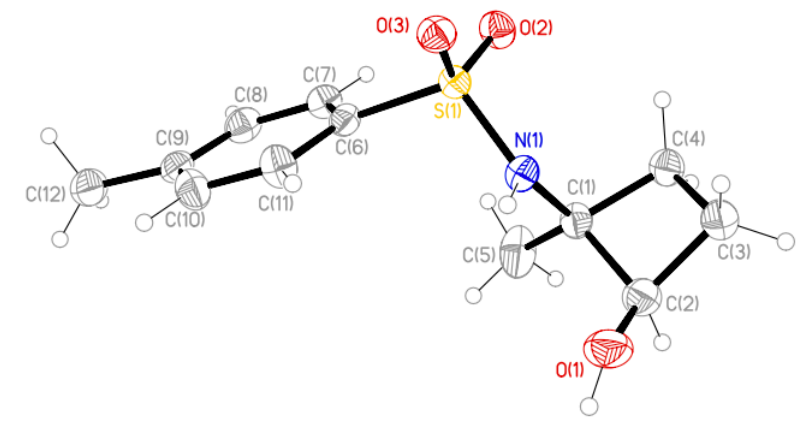

Identification code	C12H17NO3S	
Empirical formula	C12H17NO3S	
Formula weight	255.32	
Temperature	173(2) K	
Wavelength	1.54178 Å	
Crystal system	Monoclinic	
Space group	$P2_1/c$	
Unit cell dimensions	a = 10.7828(7) Å	α = 90°
	b = 16.1968(11) Å	β = 102.058(2)°
	c = 7.2382(5) Å	γ = 90°
Volume	1236.24(14) $Å^3$	
Z	4	
Density (calculated)	1.372 Mg/m^3	
Absorption coefficient	2.312 mm^{-1}	
F(000)	544	
Crystal size	0.360 x 0.220 x 0.100 mm^3	
Theta range for data collection	4.192 to 66.445 °.	
Index ranges	-12<=h<=12, -18<=k<=19, -8<=l<=8	
Reflections collected	18288	
Independent reflections	2166 [R(int) = 0.0268]	
Completeness to theta = 66.445°	99.4 %	
Absorption correction	Semi-empirical from equivalents	
Max. and min. transmission	0.7528 and 0.6080	
Refinement method	Full-matrix least-squares on F^2	
Data / restraints / parameters	2166 / 0 / 163	
Goodness-of-fit on F^2	1.101	
Final R indices [I>2sigma(I)]	R1 = 0.0432, wR2 = 0.1125	
R indices (all data)	R1 = 0.0434, wR2 = 0.1126	
Extinction coefficient	n/a	
Largest diff. peak and hole	0.754 and -0.313 e. $Å^{-3}$	

Crystal data and structure refinement for **25b**

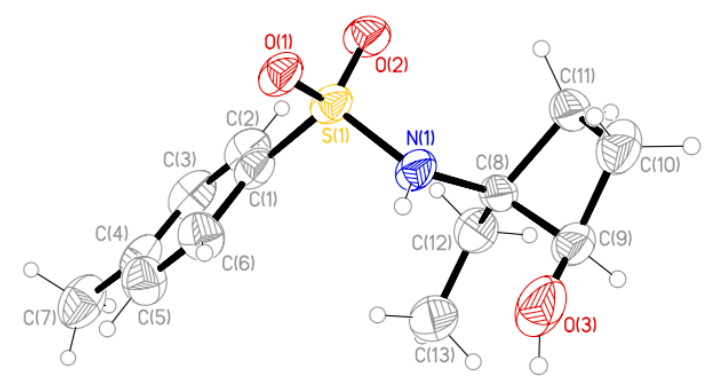

Identification code	C13H19NO3S	
Empirical formula	C13H19NO3S	
Formula weight	269.35	
Temperature	100(2) K	
Wavelength	0.71073 Å	
Crystal system	Monoclinic	
Space group	$P2_1/c$	
Unit cell dimensions	a = 11.1464(9) Å	α = 90°
	b = 16.7906(13) Å	β = 104.529(3)°
	c = 7.5194(6) Å	γ = 90°
Volume	1362.29(19) $Å^3$	
Z	4	
Density (calculated)	1.313 Mg/m^3	
Absorption coefficient	0.238 mm^{-1}	
F(000)	576	
Crystal size	0.380 x 0.280 x 0.140 mm^3	
Theta range for data collection	1.887 to 28.386°	
Index ranges	-14<=h<=14, -22<=k<=22, -8<=l<=10	
Reflections collected	27554	
Independent reflections	3390 [R(int) = 0.0342]	
Completeness to theta = 25.242°	100.0 %	
Absorption correction	Semi-empirical from equivalents	
Max. and min. transmission	0.7457 and 0.6319	
Refinement method	Full-matrix least-squares on F^2	
Data / restraints / parameters	3390 / 498 / 237	
Goodness-of-fit on F^2	1.057	
Final R indices [I>2sigma(I)]	R1 = 0.0384, wR2 = 0.0931	
R indices (all data)	R1 = 0.0539, wR2 = 0.1032	
Extinction coefficient	n/a	
Largest diff. peak and hole	0.232 and -0.340 e. $Å^{-3}$	

Crystal data and structure refinement for **38**

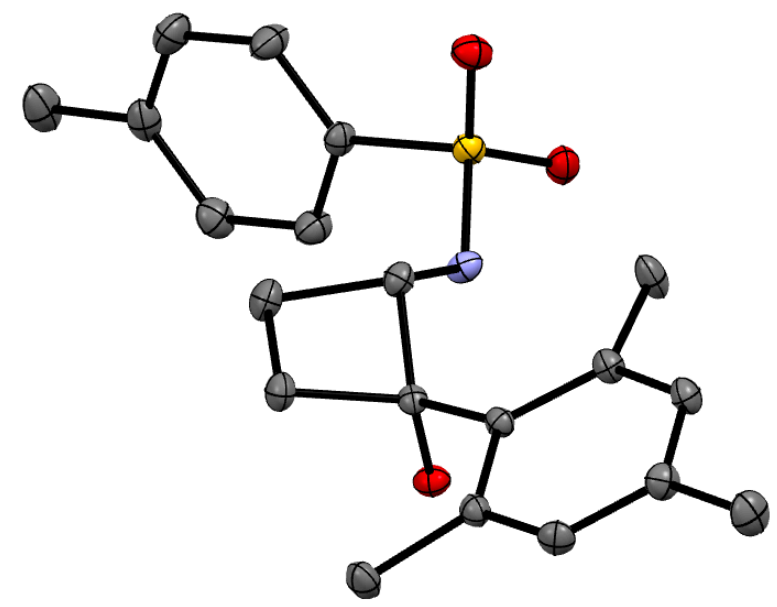

Identification code	C20H25NO3S	
Empirical formula	C20H25NO3S	
Formula weight	359.47	
Temperature	173(2) K	
Wavelength	0.71073 Å	
Crystal system	Monoclinic	
Space group	$P2_1/c$	
Unit cell dimensions	a = 9.6670(4) Å	a= 90º
	b = 17.2504(6) Å	b= 111.393(2)º
	c = 11.9009(5) Å	g = 90º
Volume	1847.85(13) $Å^3$	
Z	4	
Density (calculated)	1.292 Mg/m^3	
Absorption coefficient	0.194 mm^{-1}	
F(000)	768	
Crystal size	0.340 x 0.220 x 0.180 mm^3	
Theta range for data collection	2.263 to 28.296º	
Index ranges	-10<=h<=12, -23<=k<=22, -15<=l<=15	
Reflections collected	17352	
Independent reflections	4575 [R(int) = 0.0494]	
Completeness to theta = 25.242º	99.9 %	
Absorption correction	Semi-empirical from equivalents	
Max. and min. transmission	0.7457 and 0.7007	
Refinement method	Full-matrix least-squares on F^2	
Data / restraints / parameters	4575 / 0 / 237	
Goodness-of-fit on F^2	1.061	
Final R indices [I>2sigma(I)]	R1 = 0.0488, wR2 = 0.1232	
R indices (all data)	R1 = 0.0748, wR2 = 0.1360	
Extinction coefficient	n/a	
Largest diff. peak and hole	0.448 and -0.360 e. $Å^{-3}$	

Crystal data and structure refinement for **46**

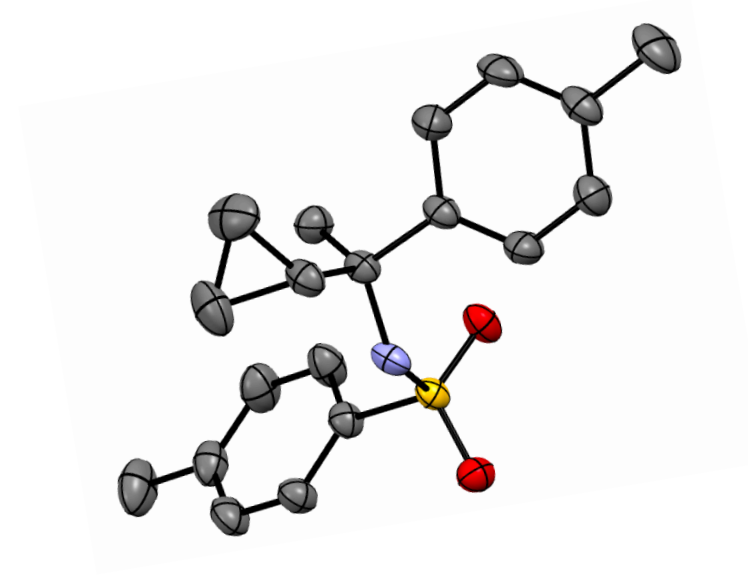

Identification code	C19H23NO2S	
Empirical formula	C19H23NO2S	
Formula weight	329.44	
Temperature	173(2) K	
Wavelength	1.54178 Å	
Crystal system	Monoclinic	
Space group	$P2_1/n$	
Unit cell dimensions	a = 9.7895(2) Å	a= 90º
	b = 9.8223(2) Å	b= 98.1010(10)º
	c = 18.2329(5) Å	g = 90º
Volume	1735.70(7) Å^3	
Z	4	
Density (calculated)	1.261 Mg/m^3	
Absorption coefficient	1.722 mm^{-1}	
F(000)	704	
Crystal size	0.320 x 0.260 x 0.140 mm^3	
Theta range for data collection	5.126 to 66.715º.	
Index ranges	-11<=h<=11, -11<=k<=11, -20<=l<=21	
Reflections collected	16299	
Independent reflections	2997 [R(int) = 0.0368]	
Completeness to theta = 66.715º	97.6 %	
Absorption correction	Semi-empirical from equivalents	
Max. and min. transmission	0.7528 and 0.6593	
Refinement method	Full-matrix least-squares on F^2	
Data / restraints / parameters	2997 / 503 / 260	
Goodness-of-fit on F^2	1.142	
Final R indices [I>2sigma(I)]	R1 = 0.0480, wR2 = 0.1130	
R indices (all data)	R1 = 0.0554, wR2 = 0.1166	
Extinction coefficient	n/a	
Largest diff. peak and hole	0.238 and -0.383 e. Å^{-3}	

Crystal data and structure refinement for **48**

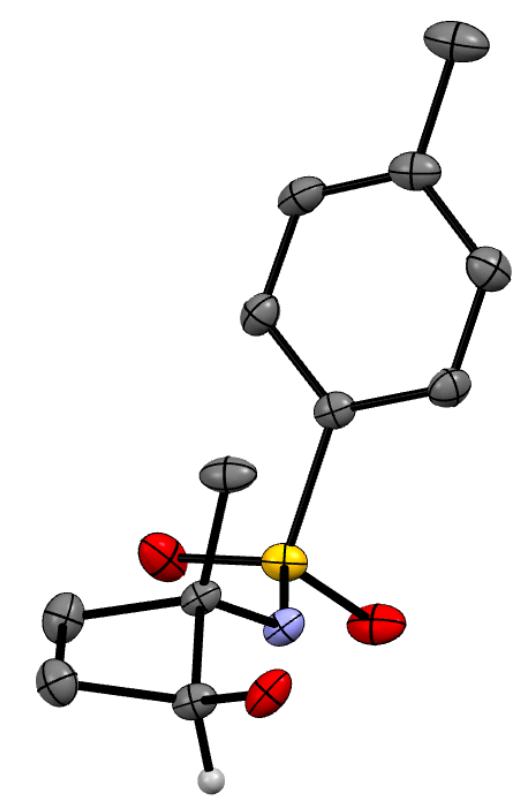

Identification code	C12H17NO3S	
Empirical formula	C12H17NO3S	
Formula weight	255.32	
Temperature	173(2) K	
Wavelength	0.71073 Å	
Crystal system	Triclinic	
Space group	P-1	
Unit cell dimensions	a = 6.4943(5) Å	a= 90.161(3)°
	b = 7.8464(6) Å	b= 99.301(3)°
	c = 13.7602(11) Å	g = 113.582(2)°
Volume	632.40(9) $Å^3$	
Z	2	
Density (calculated)	1.341 Mg/m^3	
Absorption coefficient	0.252 mm^{-1}	
F(000)	272	
Crystal size	0.320 x 0.180 x 0.120 mm^3	
Theta range for data collection	2.840 to 28.323°.	
Index ranges	-8<=h<=8, -10<=k<=10, -18<=l<=18	
Reflections collected	23226	
Independent reflections	3144 [R(int) = 0.0408]	
Completeness to theta = 25.242°	100.0 %	
Absorption correction	Semi-empirical from equivalents	
Max. and min. transmission	0.7457 and 0.6980	
Refinement method	Full-matrix least-squares on F^2	
Data / restraints / parameters	3144 / 0 / 163	
Goodness-of-fit on F^2	1.098	
Final R indices [I>2sigma(I)]	R1 = 0.0422, wR2 = 0.1087	
R indices (all data)	R1 = 0.0522, wR2 = 0.1140	
Extinction coefficient	n/a	
Largest diff. peak and hole	0.479 and -0.306 e. $Å^{-3}$	

Crystal data and structure refinement for **49**

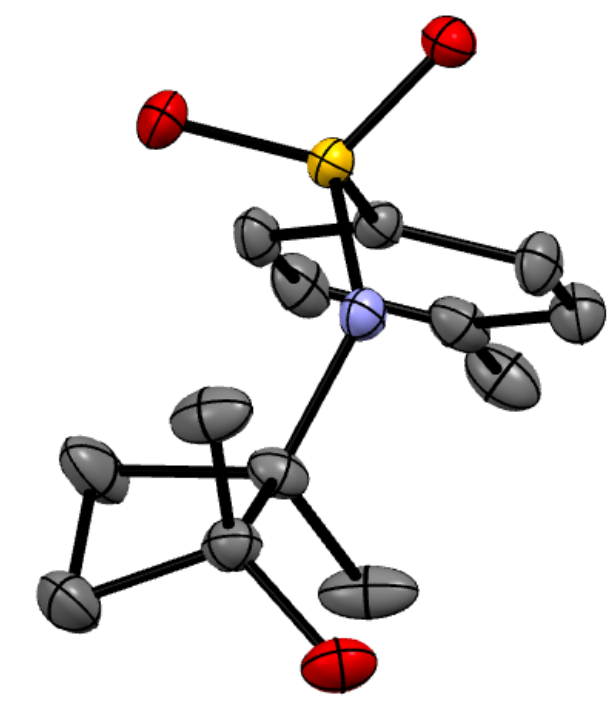

Identification code	C13H19NO3S	
Empirical formula	C13H19NO3S	
Formula weight	269.35	
Temperature	173(2) K	
Wavelength	1.54178 Å	
Crystal system	Monoclinic	
Space group	C2/c	
Unit cell dimensions	a = 21.6690(4) Å	a= 90°
	b = 10.5753(2) Å	b= 97.0300(10)°
	c = 24.0543(5) Å	g = 90°
Volume	5470.75(18) Å^3	
Z	16	
Density (calculated)	1.308 Mg/m^3	
Absorption coefficient	2.116 mm^{-1}	
F(000)	2304	
Crystal size	0.320 x 0.220 x 0.160 mm^3	
Theta range for data collection	3.703 to 66.671°.	
Index ranges	-25<=h<=25, -12<=k<=12, -28<=l<=28	
Reflections collected	44149	
Independent reflections	4841 [R(int) = 0.0517]	
Completeness to theta = 66.671°	99.8 %	
Absorption correction	Semi-empirical from equivalents	
Max. and min. transmission	0.7528 and 0.6154	
Refinement method	Full-matrix least-squares on F^2	
Data / restraints / parameters	4841 / 980 / 438	
Goodness-of-fit on F^2	1.209	
Final R indices [I>2sigma(I)]	R1 = 0.0574, wR2 = 0.1226	
R indices (all data)	R1 = 0.0601, wR2 = 0.1238	
Extinction coefficient	n/a	
Largest diff. peak and hole	0.335 and -0.276 e. Å^{-3}	

References

[1] F. N. Tebbe, G. W. Parshall, G. S. Reddy, *J. Am. Chem. Soc.* **1978**, *100*, 3611–3613.

[2] a)B. D. A. Hook, W. Dohle, P. R. Hirst, M. Pickworth, M. B. Berry, K. I. Booker-Milburn, *J. Org. Chem.* **2005**, *70*, 7558−7564; b) F. Wojcik, A. G. O'Brien, S. Götze, P. H. Seeberger, L. Hartmann, *Chem. Eur. J.* **2013**, *19*, 3090−3098; A. J. V. Marwitz, M. H. Matus, L. N. Zakharov, D. A. Dixon, S.-Y. Liu, *Angew. Chem. Int. Ed.* **2009**, *48*, 973−977.

[3] C. R. McConnell, F. Haeffner, A. W. Baggett, S.-Y. Liu, *J. Am. Chem. Soc*. **2019**, *141*, 9072–9078.

[4] T, Ozaki, S. K. Bentley, N. Rybansky, B. Li, S.-Y. Liu, *J. Am. Chem. Soc*. **2024**, *146,* 24748–24753.

[5] A. N. Brown, B. Li, and S. -Y. Liu, *J. Am. Chem. Soc.* **2015**, *137*, 8932–8935.

[6] L. J. Gooβen, *Chem. Commun*, **2001**, 669–670.

NMR Spectra

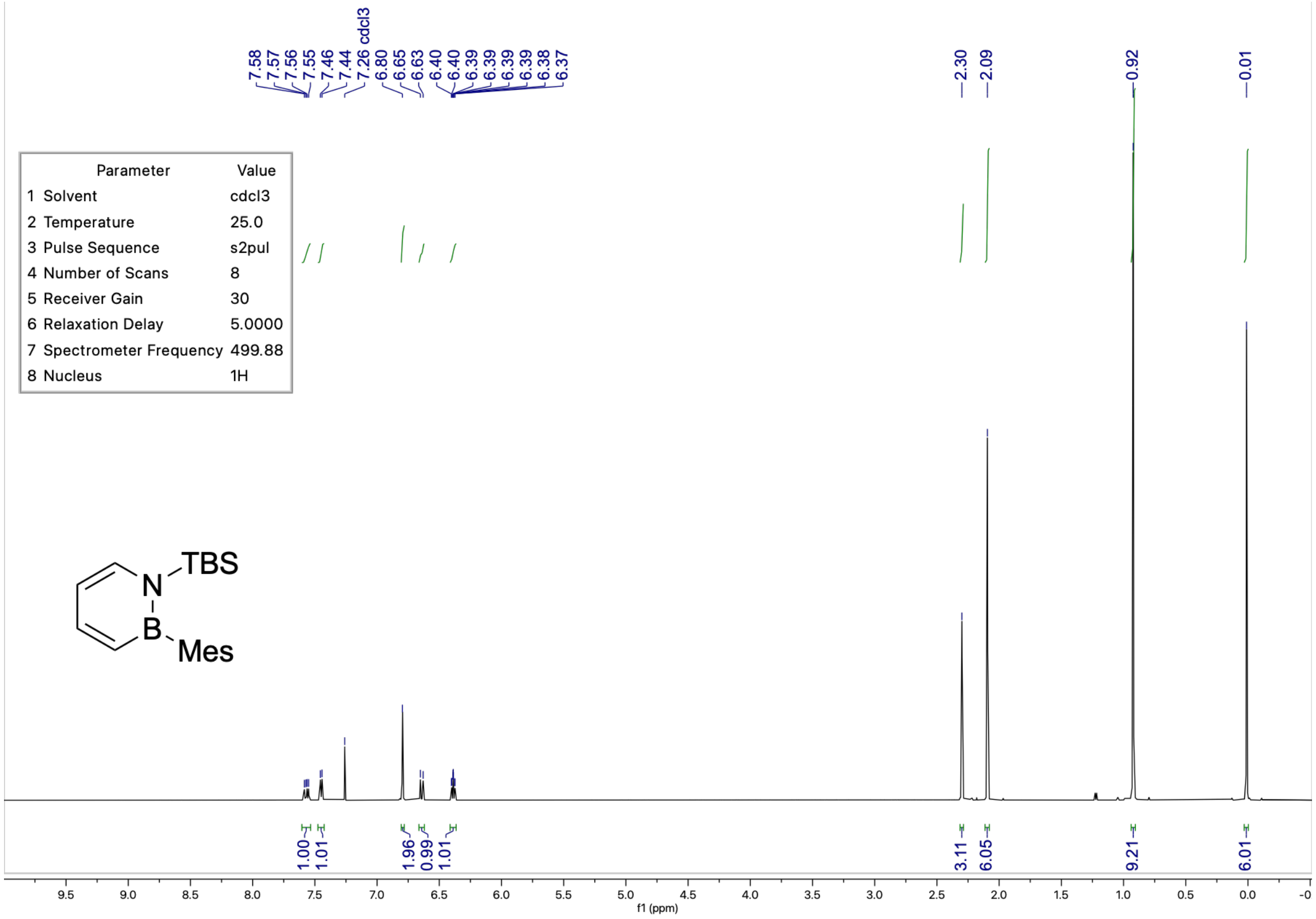

7.58
7.57
7.56
7.55
7.46
7.44
7.26 cdcl3
6.80
6.65
6.63
6.40
6.40
6.39
6.39
6.39
6.39
6.38
6.37
2.30
2.09
0.92
0.01
Parameter | Value
1 Solvent | cdcl3
2 Temperature | 25.0
3 Pulse Sequence | s2pul
4 Number of Scans | 8
5 Receiver Gain | 30
6 Relaxation Delay | 5.0000
7 Spectrometer Frequency | 499.88
8 Nucleus | 1H
N
TBS
B
Mes
1.00
1.01
1.96
0.99
1.01
3.11
6.05
9.21
6.01
f1 (ppm)

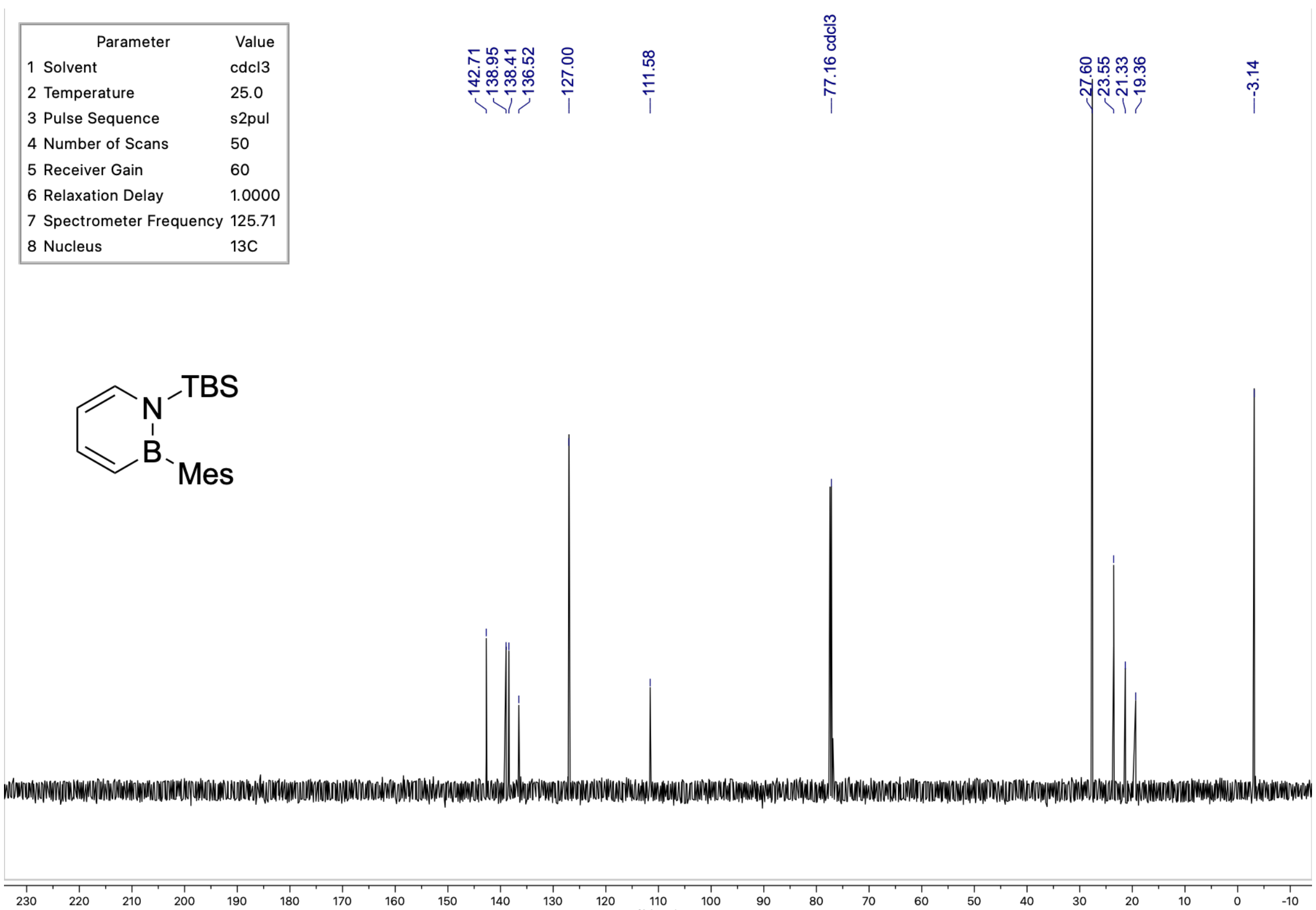

Parameter | Value
1 Solvent | cdcl3
2 Temperature | 25.0
3 Pulse Sequence | s2pul
4 Number of Scans | 50
5 Receiver Gain | 60
6 Relaxation Delay | 1.0000
7 Spectrometer Frequency | 125.71
8 Nucleus | 13C
142.71
138.95
138.41
136.52
127.00
111.58
77.16 cdcl3
27.60
23.55
21.33
19.36
-3.14
N
TBS
B
Mes
f1 (ppm)

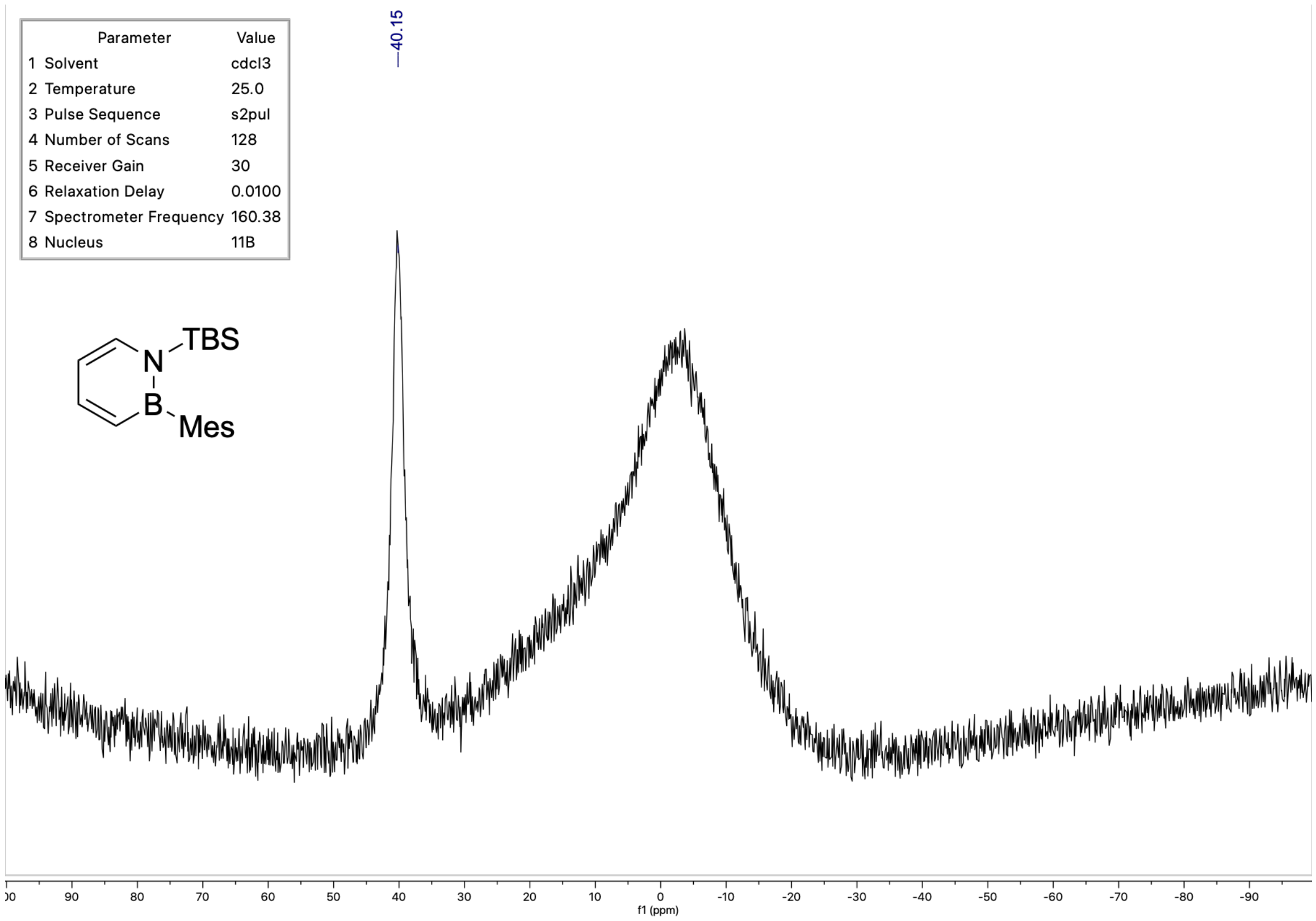

Parameter Value
1 Solvent cdcl3
2 Temperature 25.0
3 Pulse Sequence s2pul
4 Number of Scans 128
5 Receiver Gain 30
6 Relaxation Delay 0.0100
7 Spectrometer Frequency 160.38
8 Nucleus 11B
40.15
N
TBS
B
Mes
f1 (ppm)

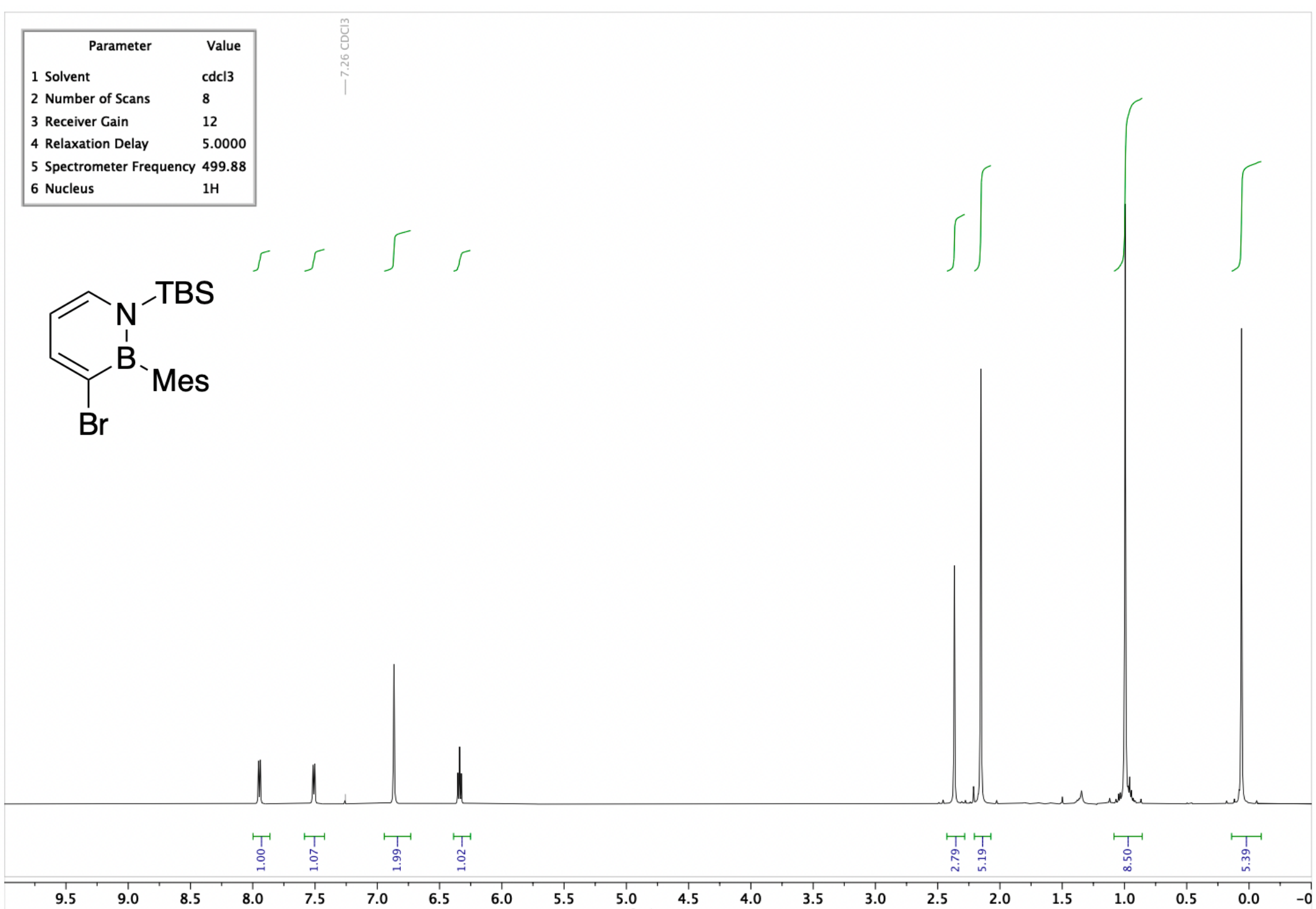

Parameter Value
1 Solvent cdcl3
2 Number of Scans 8
3 Receiver Gain 12
4 Relaxation Delay 5.0000
5 Spectrometer Frequency 499.88
6 Nucleus 1H
7.26 CDCl3
N
TBS
B
Mes
Br
1.00
1.07
1.99
1.02
2.79
5.19
8.50
5.39
f1 (ppm)

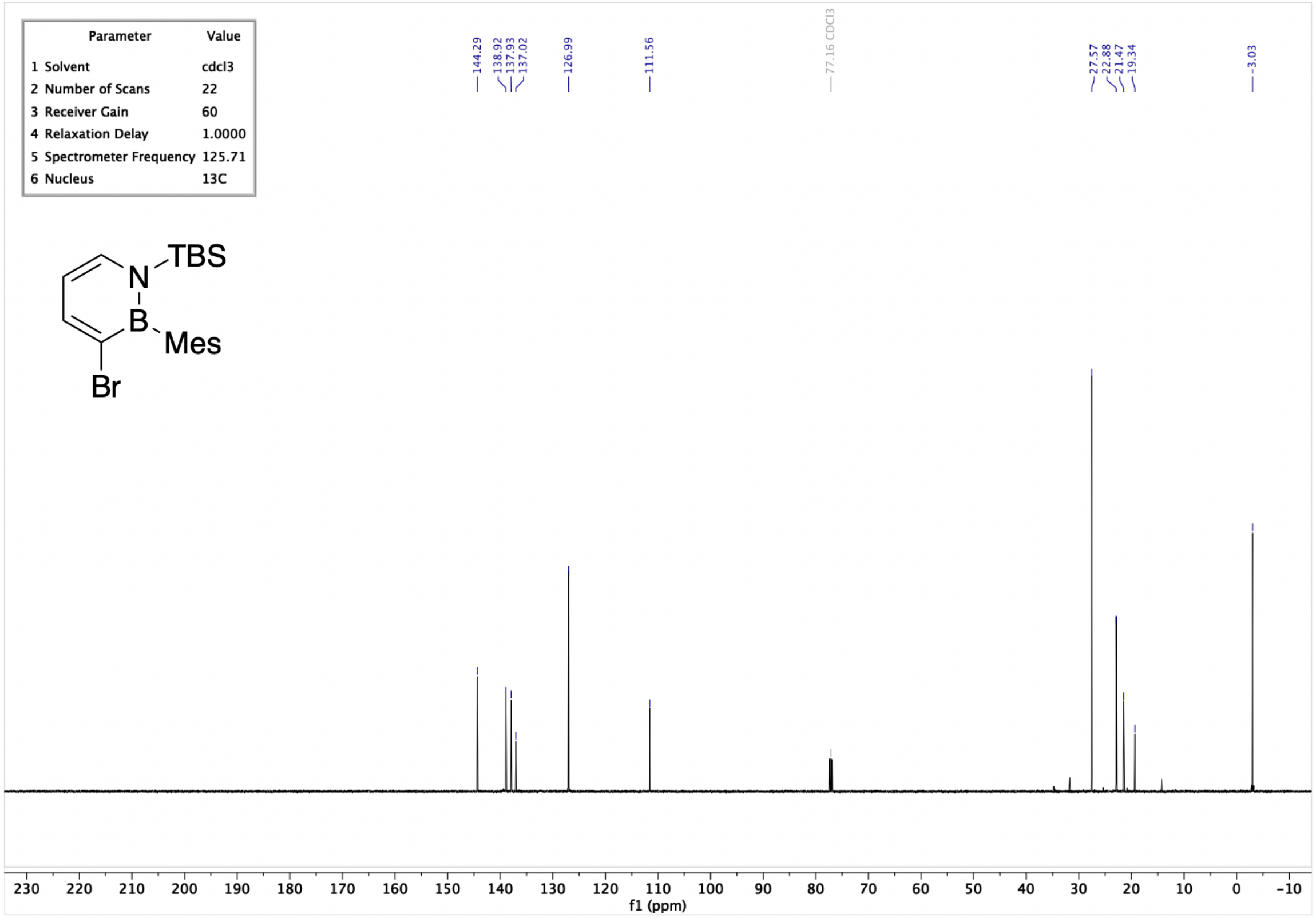
Parameter Value
1 Solvent cdcl3
2 Number of Scans 22
3 Receiver Gain 60
4 Relaxation Delay 1.0000
5 Spectrometer Frequency 125.71
6 Nucleus 13C
TBS
N
B
Mes
Br
144.29
138.92
137.93
137.02
126.99
111.56
77.16 CDCl3
27.57
22.88
21.47
19.34
-3.03
f1 (ppm)

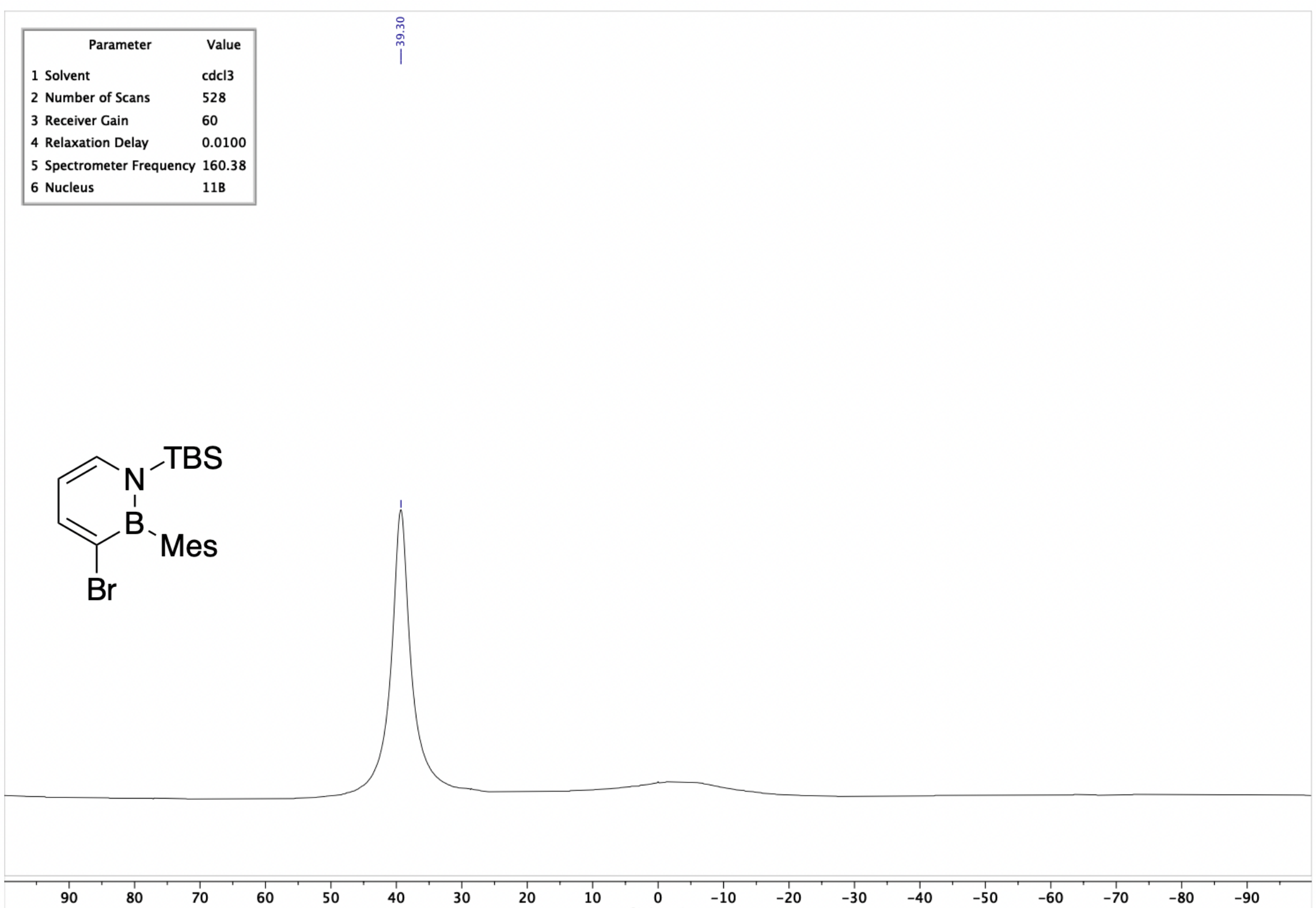
Parameter Value
1 Solvent cdcl3
2 Number of Scans 528
3 Receiver Gain 60
4 Relaxation Delay 0.0100
5 Spectrometer Frequency 160.38
6 Nucleus 11B
39.30
TBS
N
B
Mes
Br
f1 (ppm)

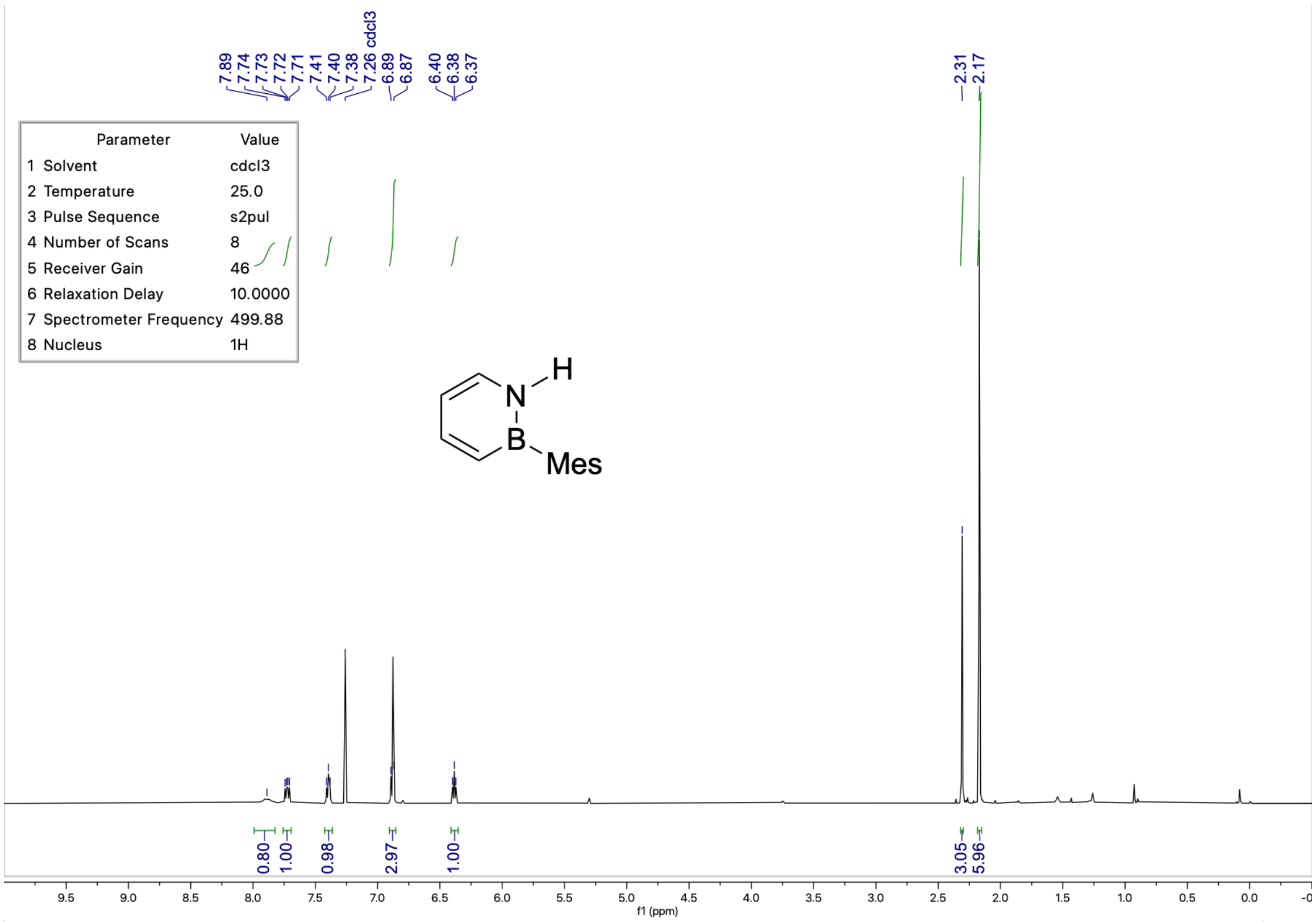
7.89 7.74 7.73 7.72 7.71 7.41 7.40 7.38 7.26 cdcl3 6.89 6.87 6.40 6.38 6.37
2.31 2.17
Parameter Value
1 Solvent cdcl3
2 Temperature 25.0
3 Pulse Sequence s2pul
4 Number of Scans 8
5 Receiver Gain 46
6 Relaxation Delay 10.0000
7 Spectrometer Frequency 499.88
8 Nucleus 1H
N H
B Mes
0.80 1.00 0.98 2.97 1.00 3.05 5.96
f1 (ppm)

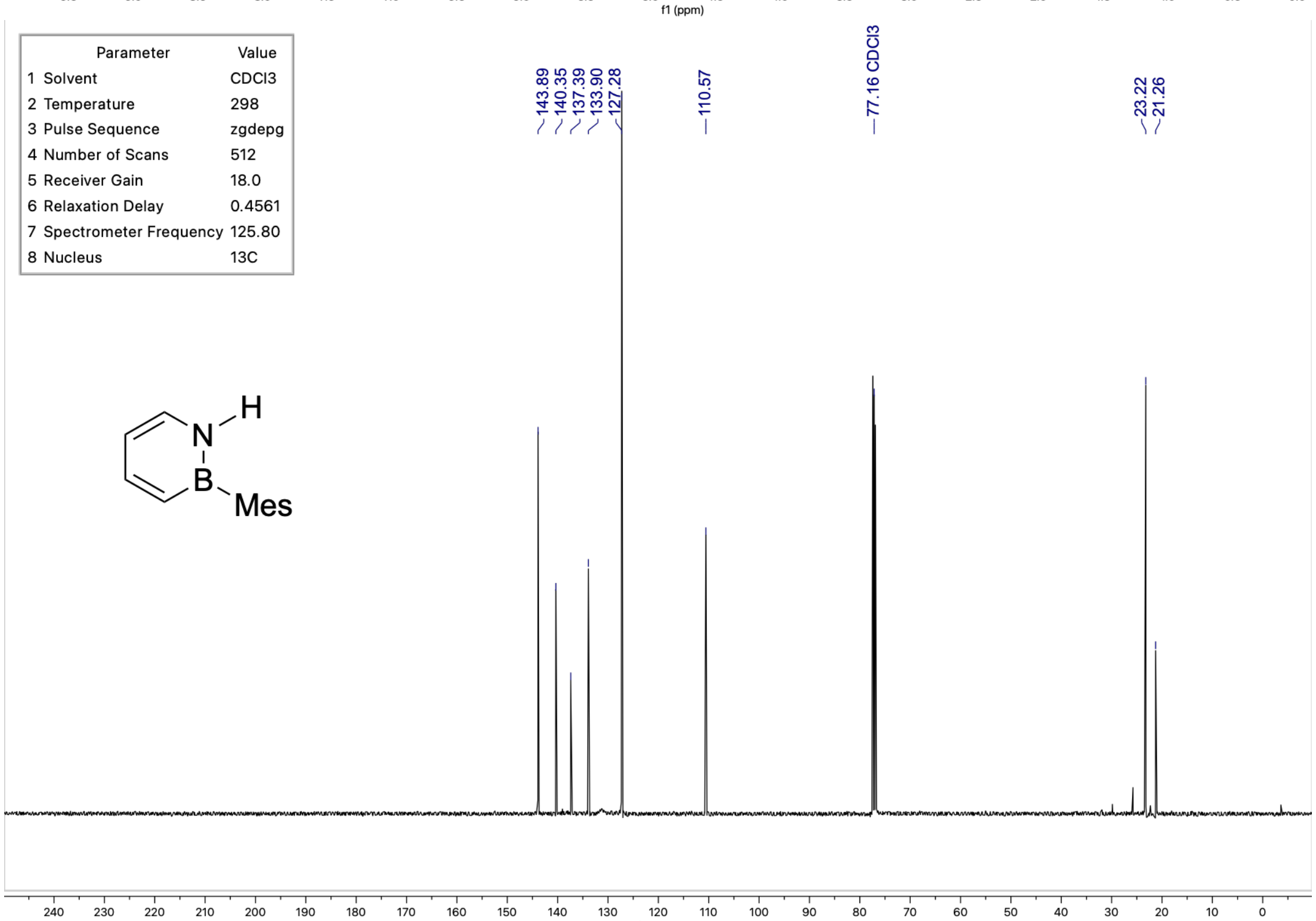
Parameter Value
1 Solvent CDCl3
2 Temperature 298
3 Pulse Sequence zgdepg
4 Number of Scans 512
5 Receiver Gain 18.0
6 Relaxation Delay 0.4561
7 Spectrometer Frequency 125.80
8 Nucleus 13C
143.89 140.35 137.39 133.90 127.28
110.57
77.16 CDCl3
23.22 21.26
N H
B Mes
f1 (ppm)

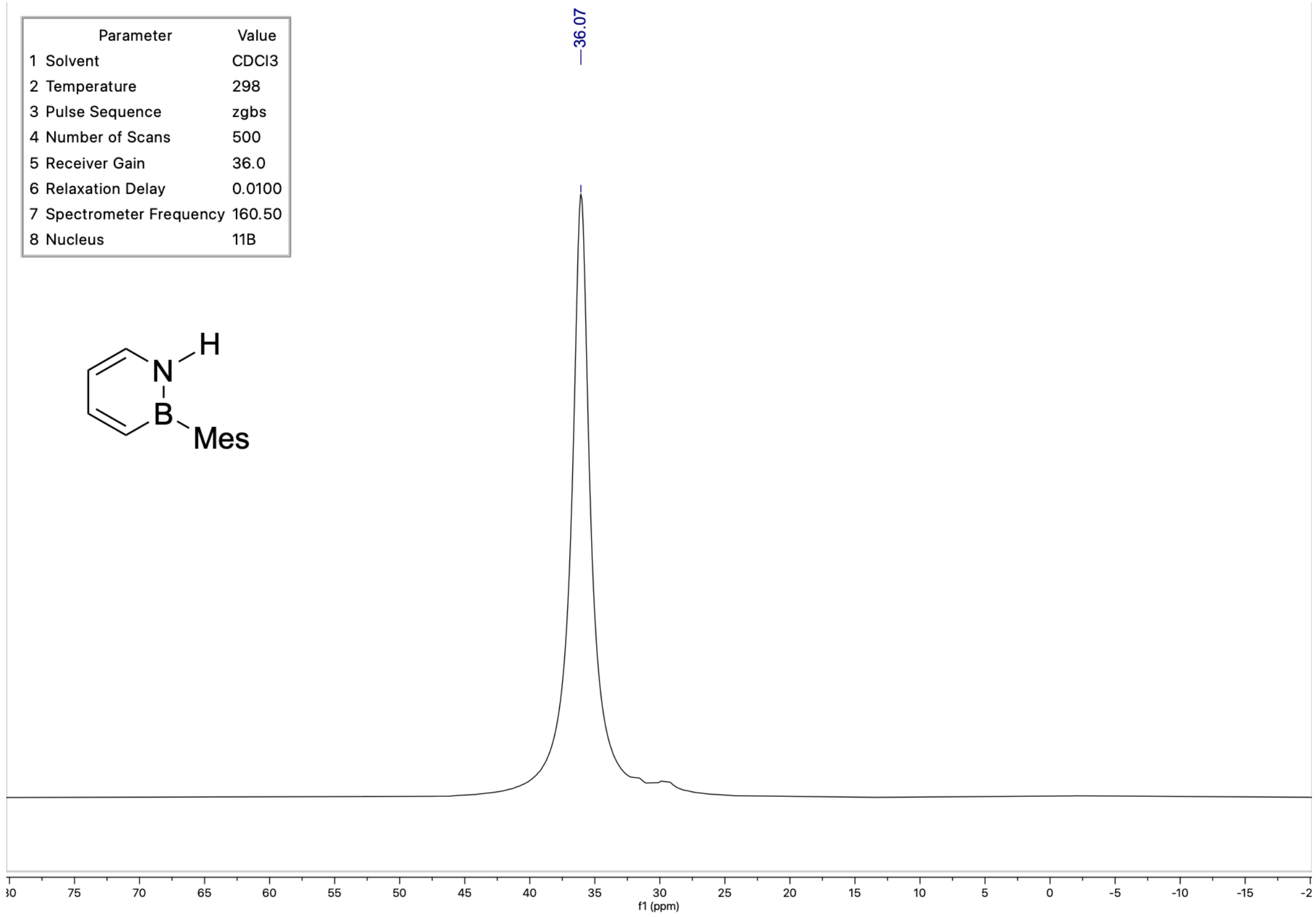

Parameter Value
1 Solvent CDCl3
2 Temperature 298
3 Pulse Sequence zgbs
4 Number of Scans 500
5 Receiver Gain 36.0
6 Relaxation Delay 0.0100
7 Spectrometer Frequency 160.50
8 Nucleus 11B
36.07
Mes
f1 (ppm)

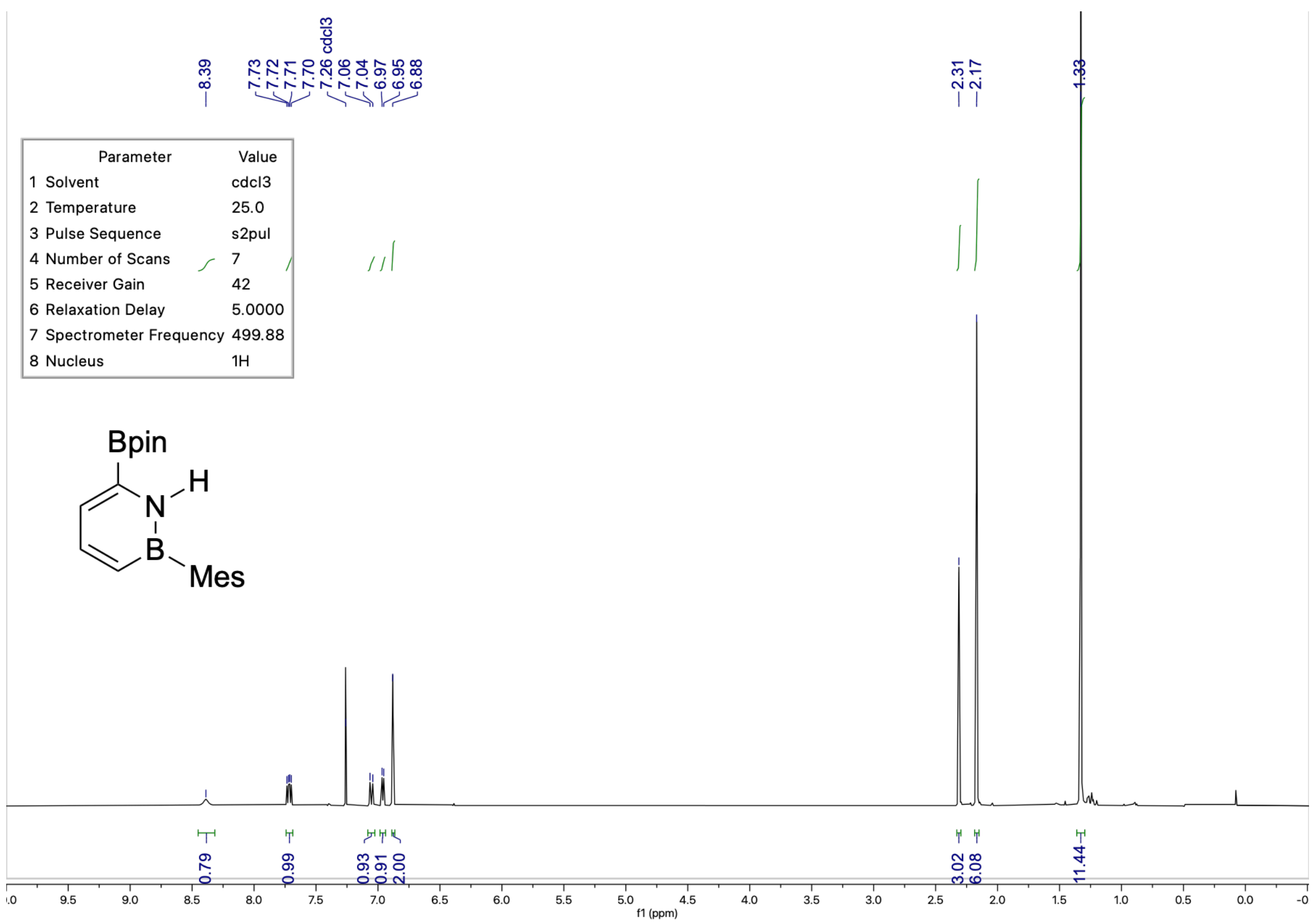

Parameter Value
1 Solvent cdcl3
2 Temperature 25.0
3 Pulse Sequence s2pul
4 Number of Scans 7
5 Receiver Gain 42
6 Relaxation Delay 5.0000
7 Spectrometer Frequency 499.88
8 Nucleus 1H
8.39
7.73
7.72
7.71
7.70
7.26 cdcl3
7.06
7.04
6.97
6.95
6.88
2.31
2.17
1.33
Bpin
Mes
0.79
0.99
0.93
0.91
2.00
3.02
6.08
11.44
f1 (ppm)

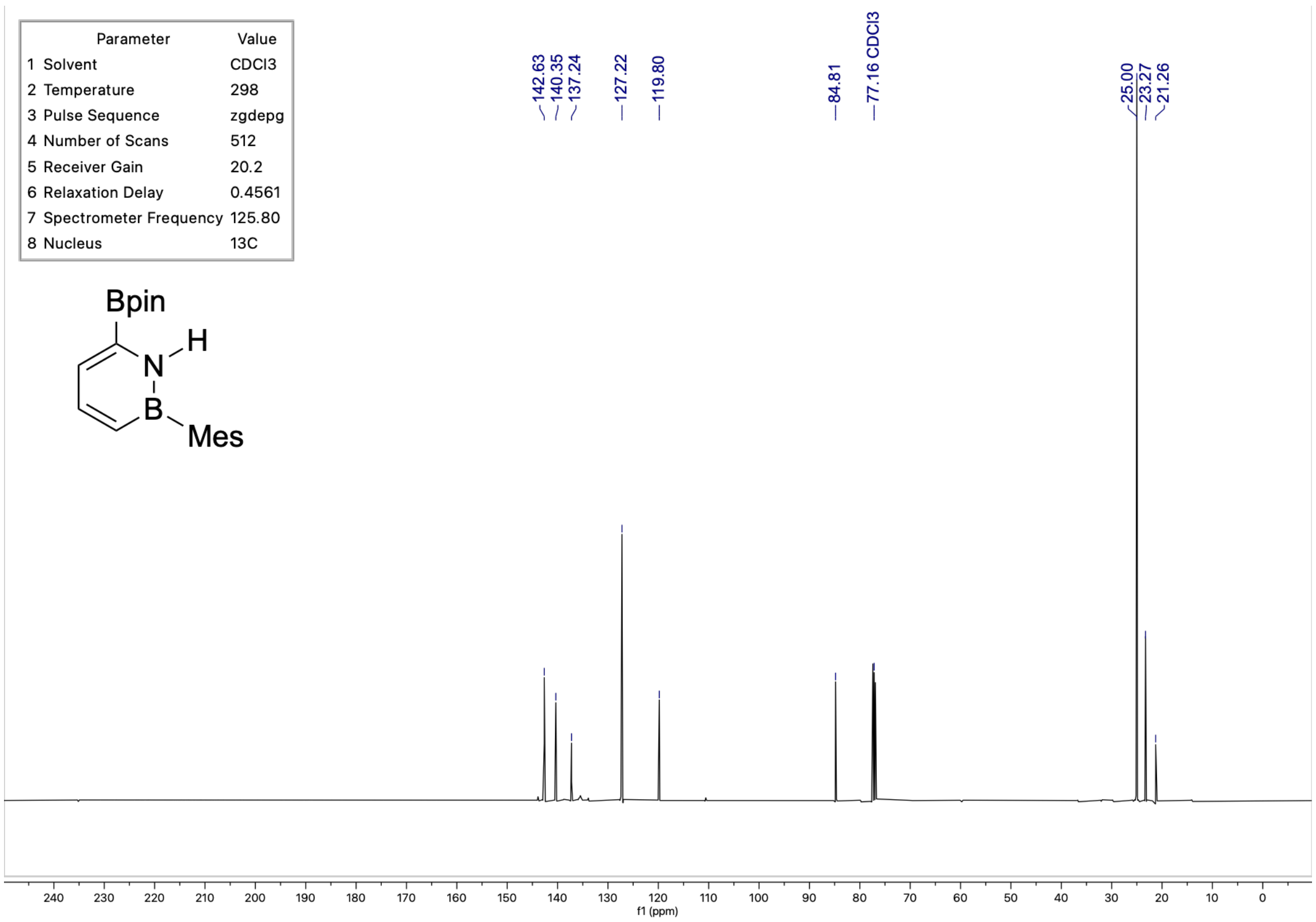
Parameter Value
1 Solvent CDCl3
2 Temperature 298
3 Pulse Sequence zgdepg
4 Number of Scans 512
5 Receiver Gain 20.2
6 Relaxation Delay 0.4561
7 Spectrometer Frequency 125.80
8 Nucleus 13C
Bpin
H
N
B
Mes
142.63
140.35
137.24
127.22
119.80
84.81
77.16 CDCl3
25.00
23.27
21.26
f1 (ppm)

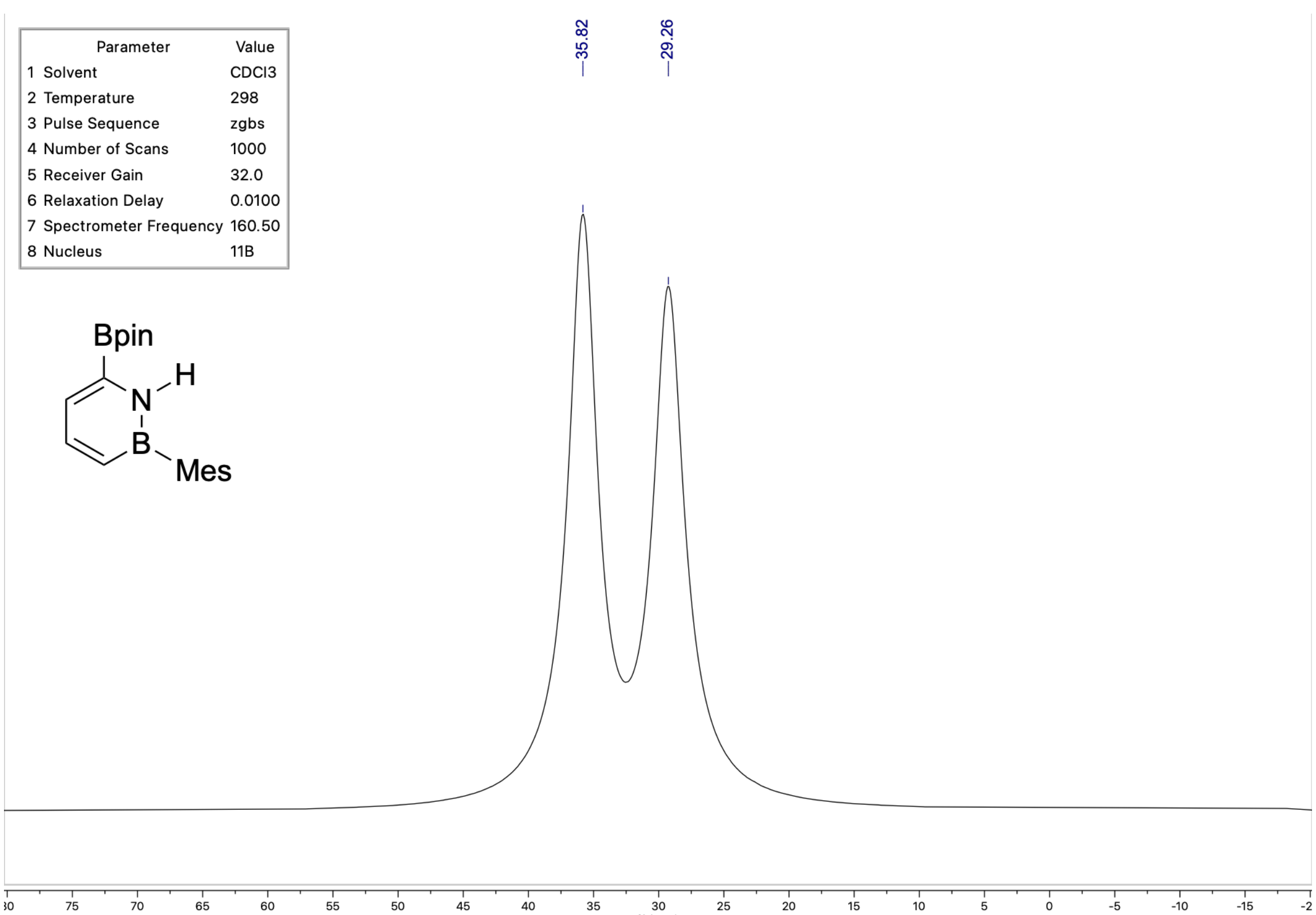
Parameter Value
1 Solvent CDCl3
2 Temperature 298
3 Pulse Sequence zgbs
4 Number of Scans 1000
5 Receiver Gain 32.0
6 Relaxation Delay 0.0100
7 Spectrometer Frequency 160.50
8 Nucleus 11B
Bpin
H
N
B
Mes
35.82
29.26
f1 (ppm)

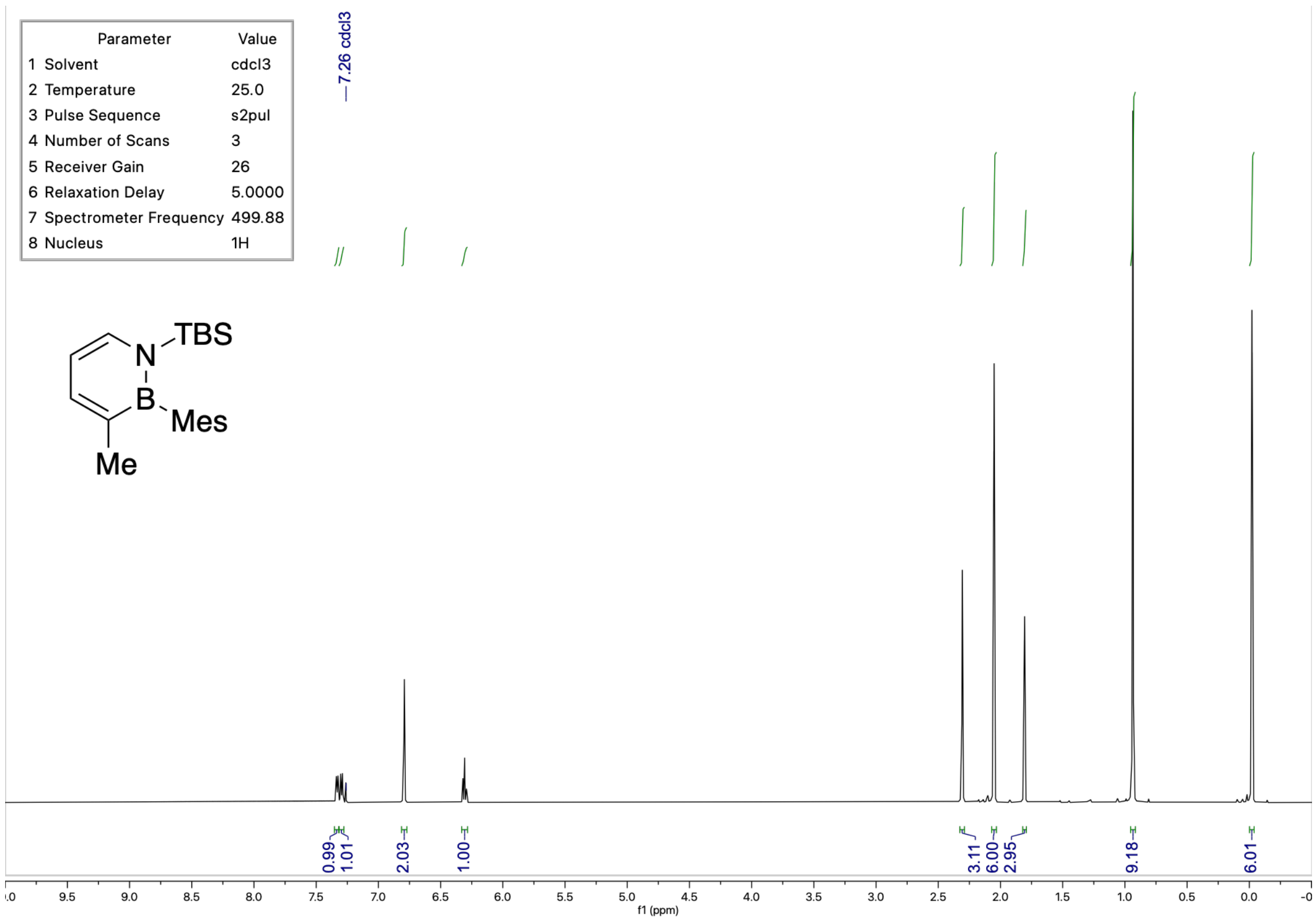
Parameter Value
1 Solvent cdcl3
2 Temperature 25.0
3 Pulse Sequence s2pul
4 Number of Scans 3
5 Receiver Gain 26
6 Relaxation Delay 5.0000
7 Spectrometer Frequency 499.88
8 Nucleus 1H
TBS
N
B
Mes
Me
7.26 cdcl3
0.99
1.01
2.03
1.00
3.11
6.00
2.95
9.18
6.01
f1 (ppm)

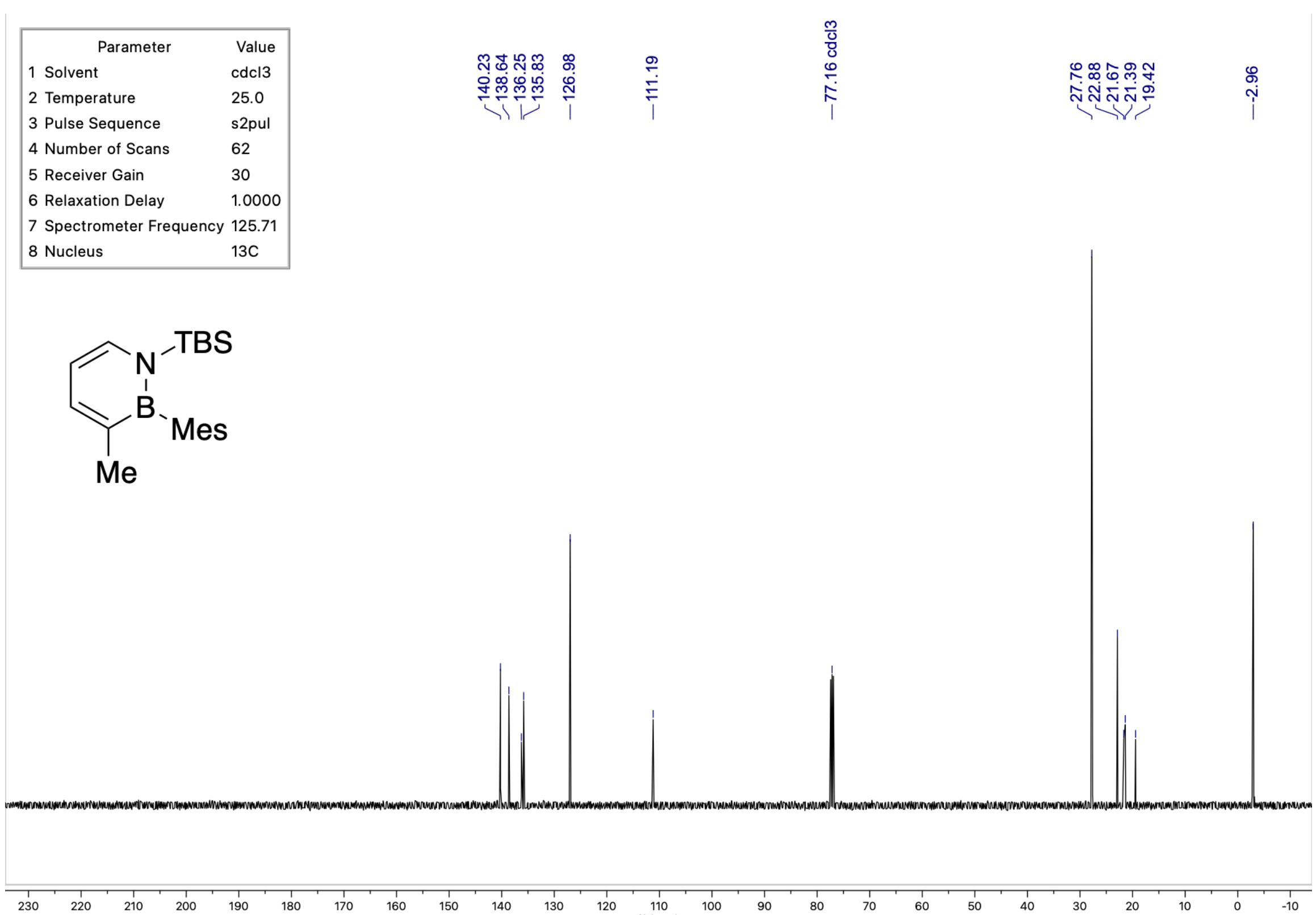
Parameter Value
1 Solvent cdcl3
2 Temperature 25.0
3 Pulse Sequence s2pul
4 Number of Scans 62
5 Receiver Gain 30
6 Relaxation Delay 1.0000
7 Spectrometer Frequency 125.71
8 Nucleus 13C
TBS
N
B
Mes
Me
140.23
138.64
136.25
135.83
126.98
111.19
77.16 cdcl3
27.76
22.88
21.67
21.39
19.42
-2.96
f1 (ppm)

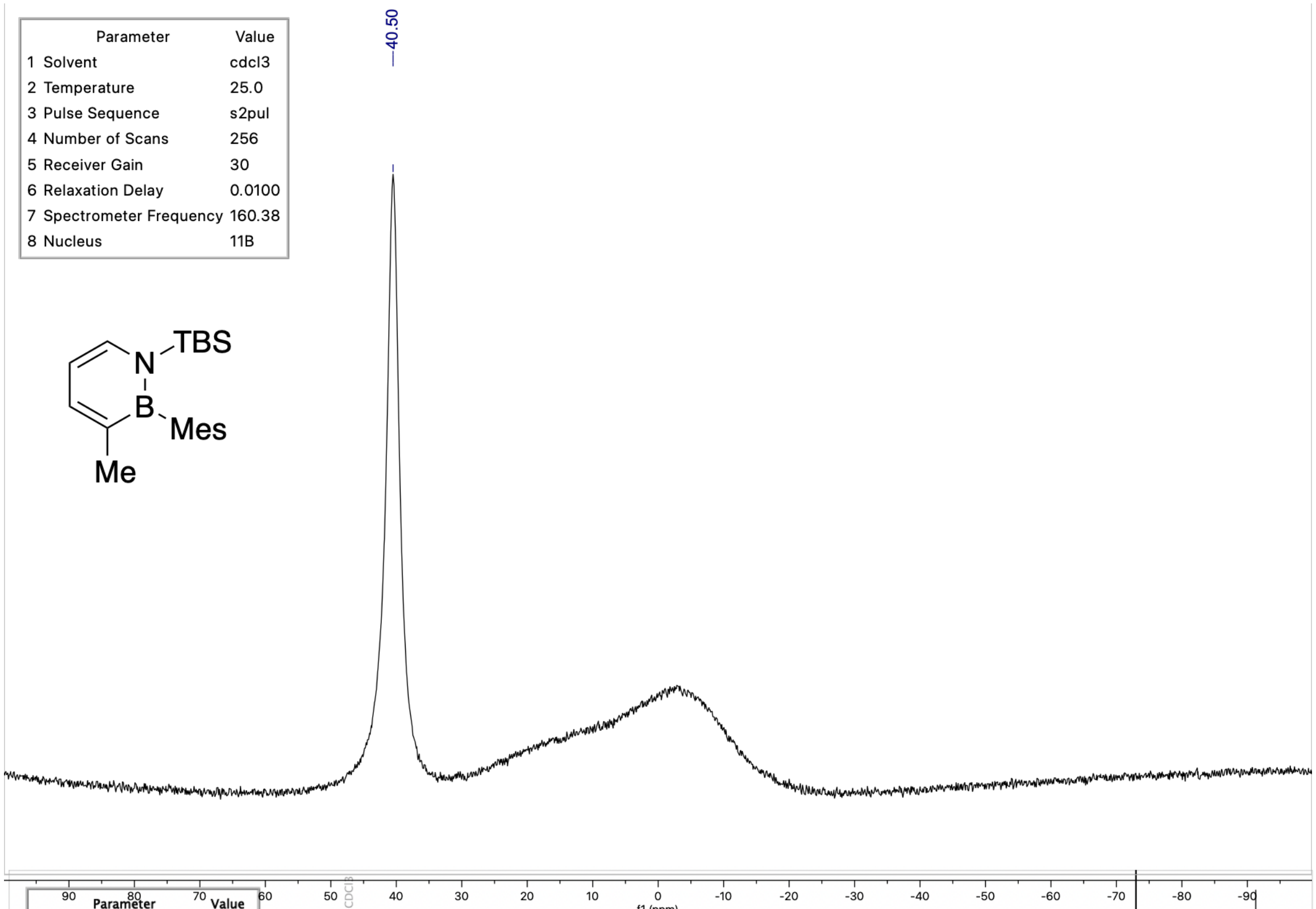

Parameter Value
1 Solvent cdcl3
2 Temperature 25.0
3 Pulse Sequence s2pul
4 Number of Scans 256
5 Receiver Gain 30
6 Relaxation Delay 0.0100
7 Spectrometer Frequency 160.38
8 Nucleus 11B
N TBS
B Mes
Me
40.50
f1 (ppm)

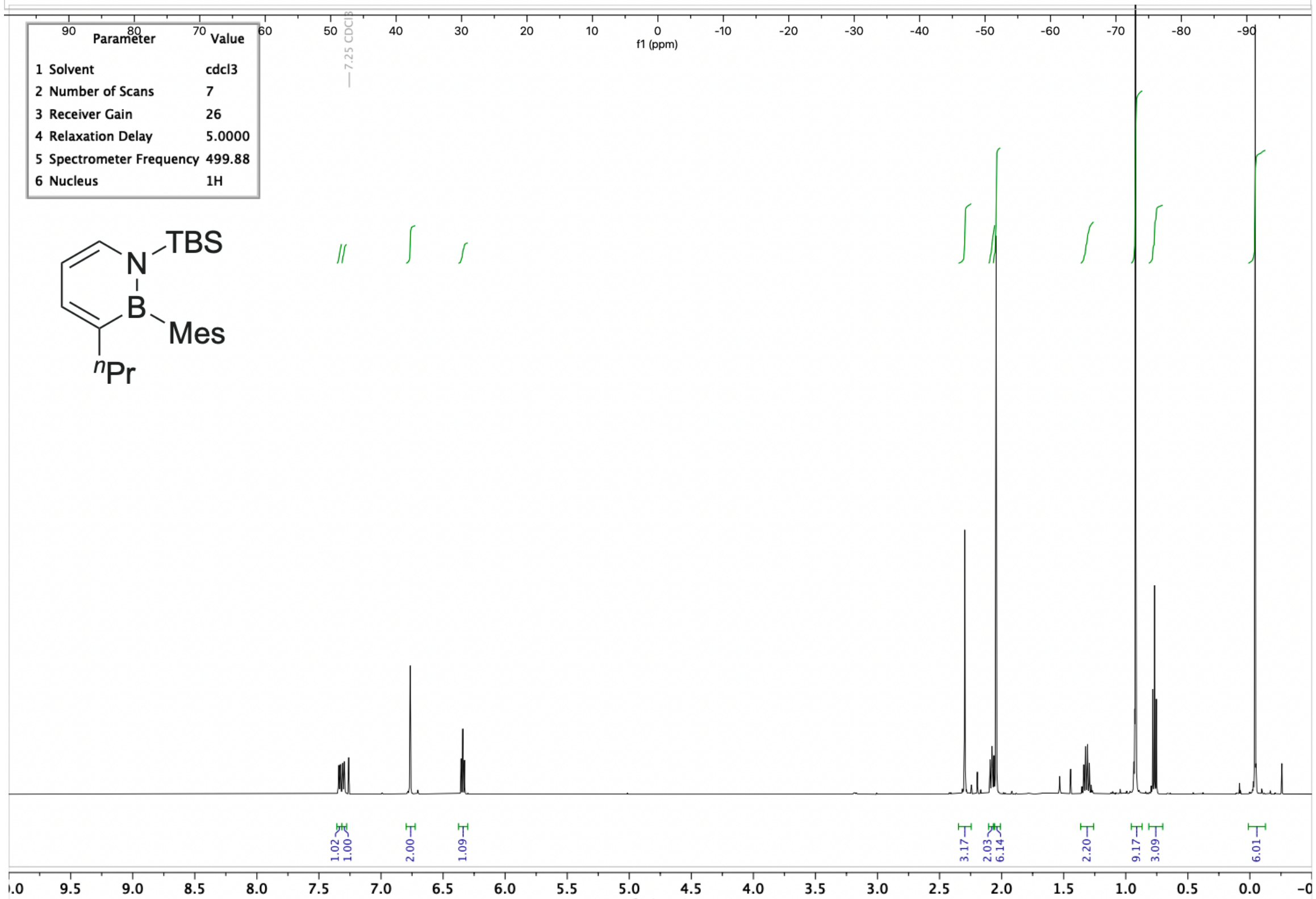

Parameter Value
1 Solvent cdcl3
2 Number of Scans 7
3 Receiver Gain 26
4 Relaxation Delay 5.0000
5 Spectrometer Frequency 499.88
6 Nucleus 1H
N TBS
B Mes
nPr
7.25 CDCl3
1.02
1.00
2.00
1.09
3.17
2.03
6.14
2.20
9.17
3.09
6.01
f1 (ppm)

Parameter	Value
1 Solvent	cdcl3
2 Number of Scans	68
3 Receiver Gain	60
4 Relaxation Delay	1.0000
5 Spectrometer Frequency	125.71
6 Nucleus	13C

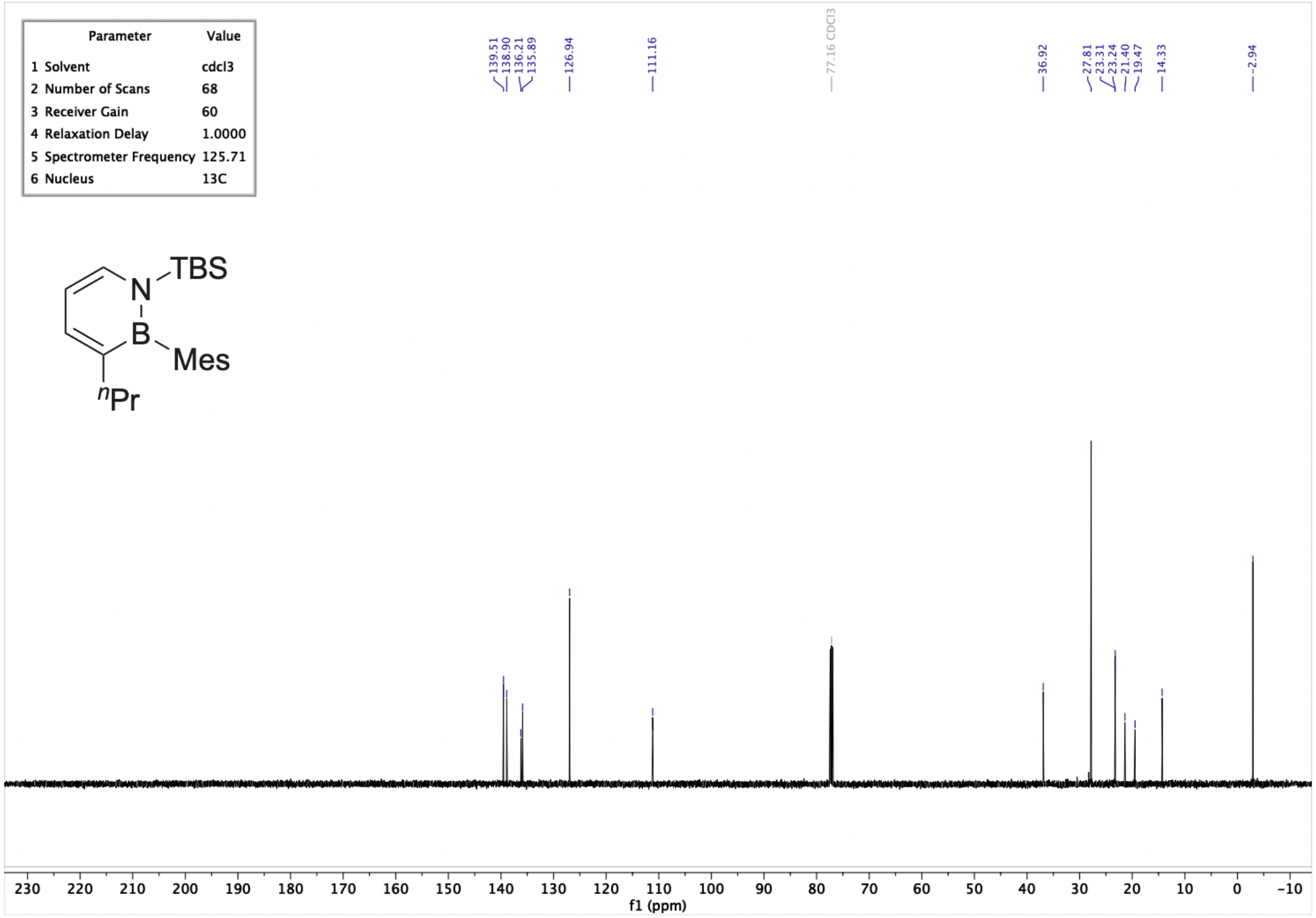

Parameter	Value
1 Solvent	cdcl3
2 Number of Scans	110
3 Receiver Gain	60
4 Relaxation Delay	0.0100
5 Spectrometer Frequency	160.38
6 Nucleus	11B

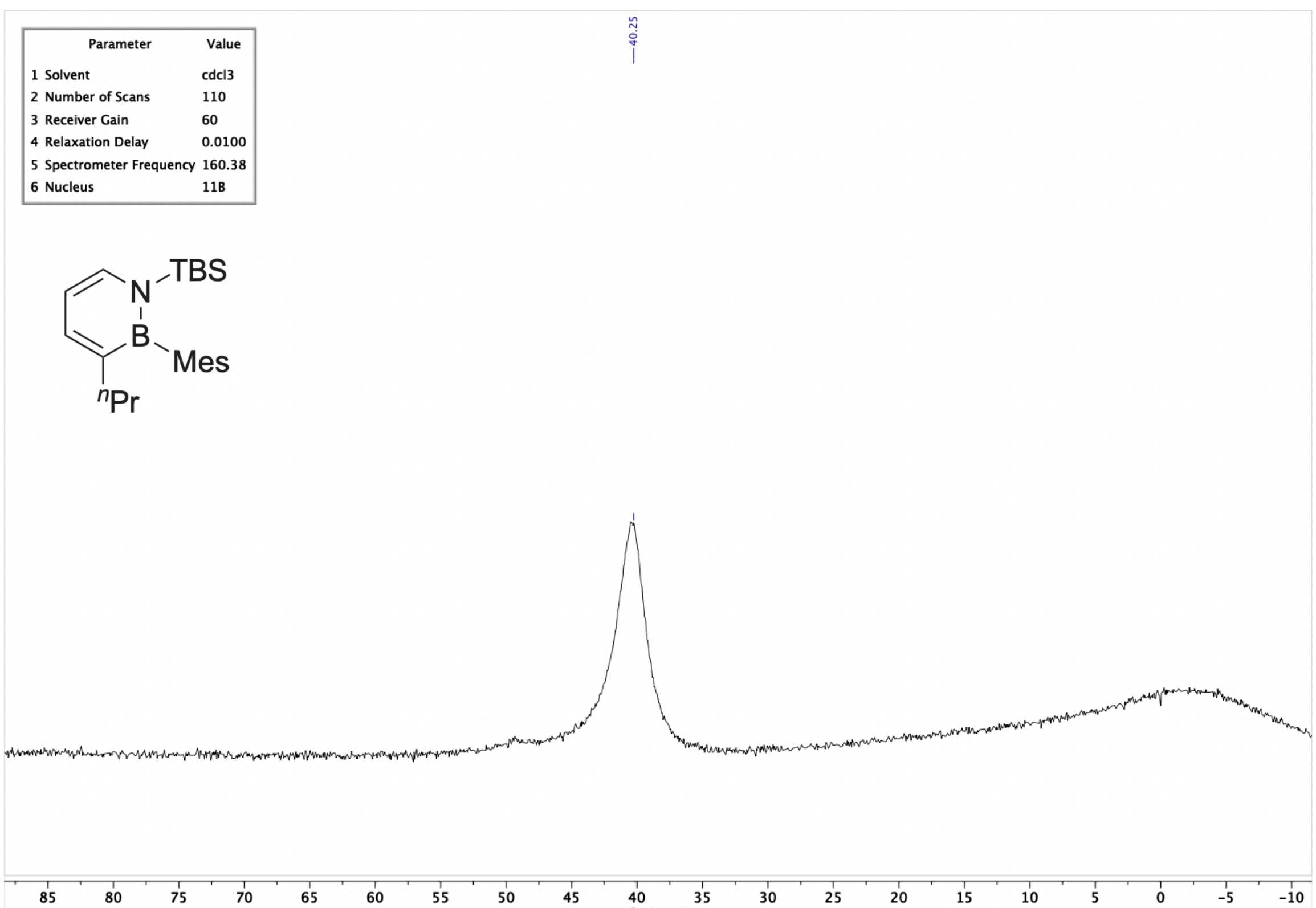

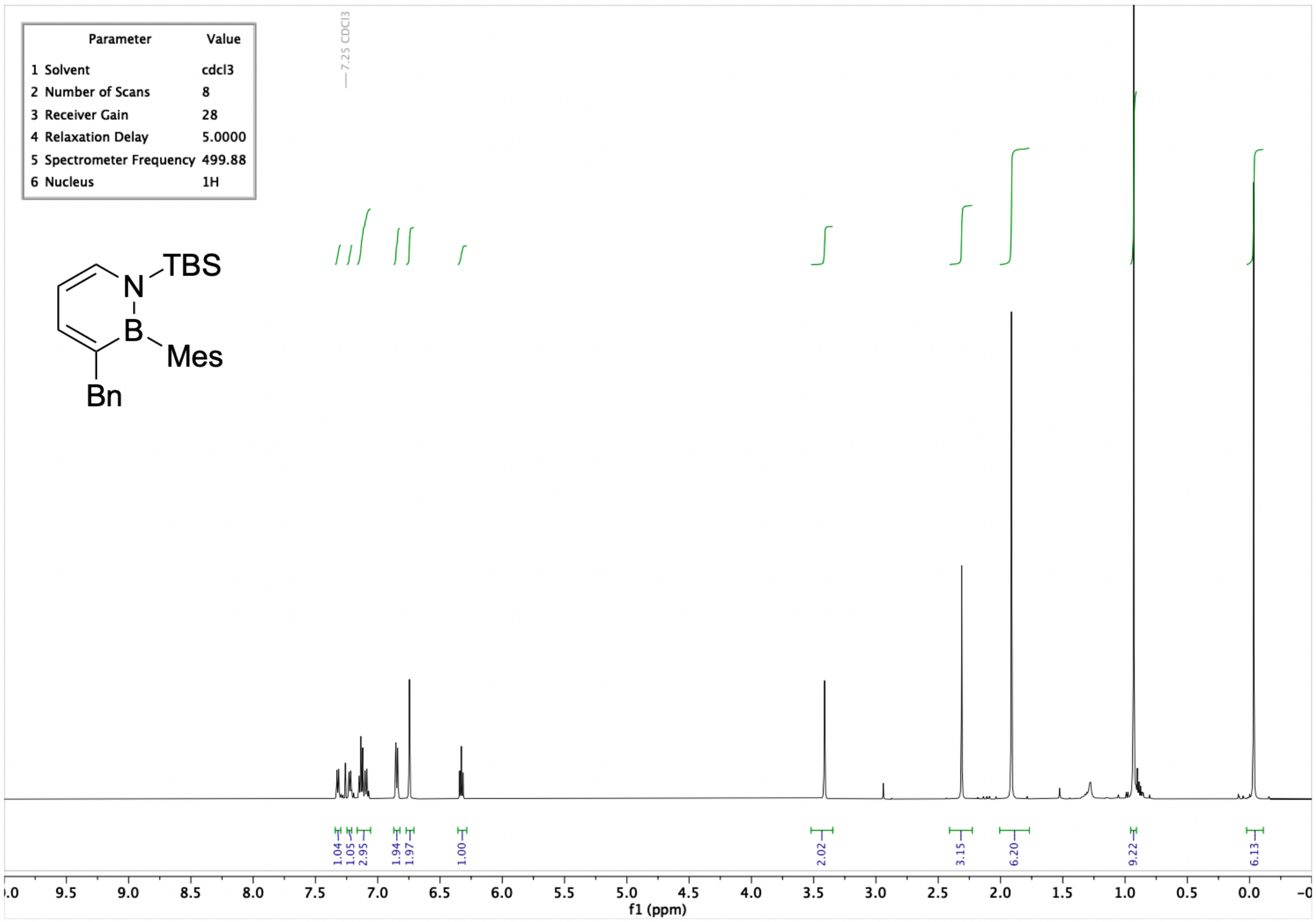

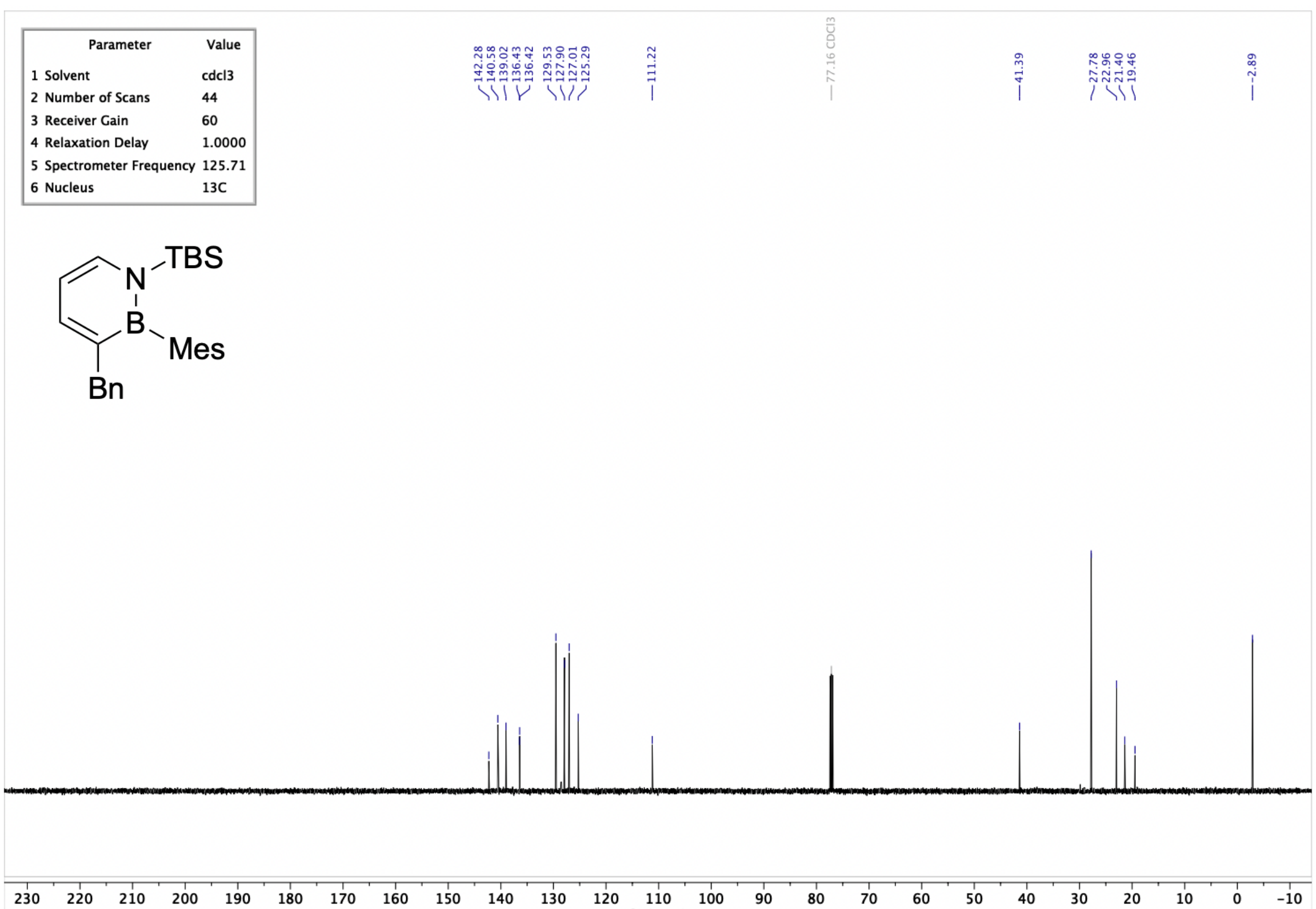

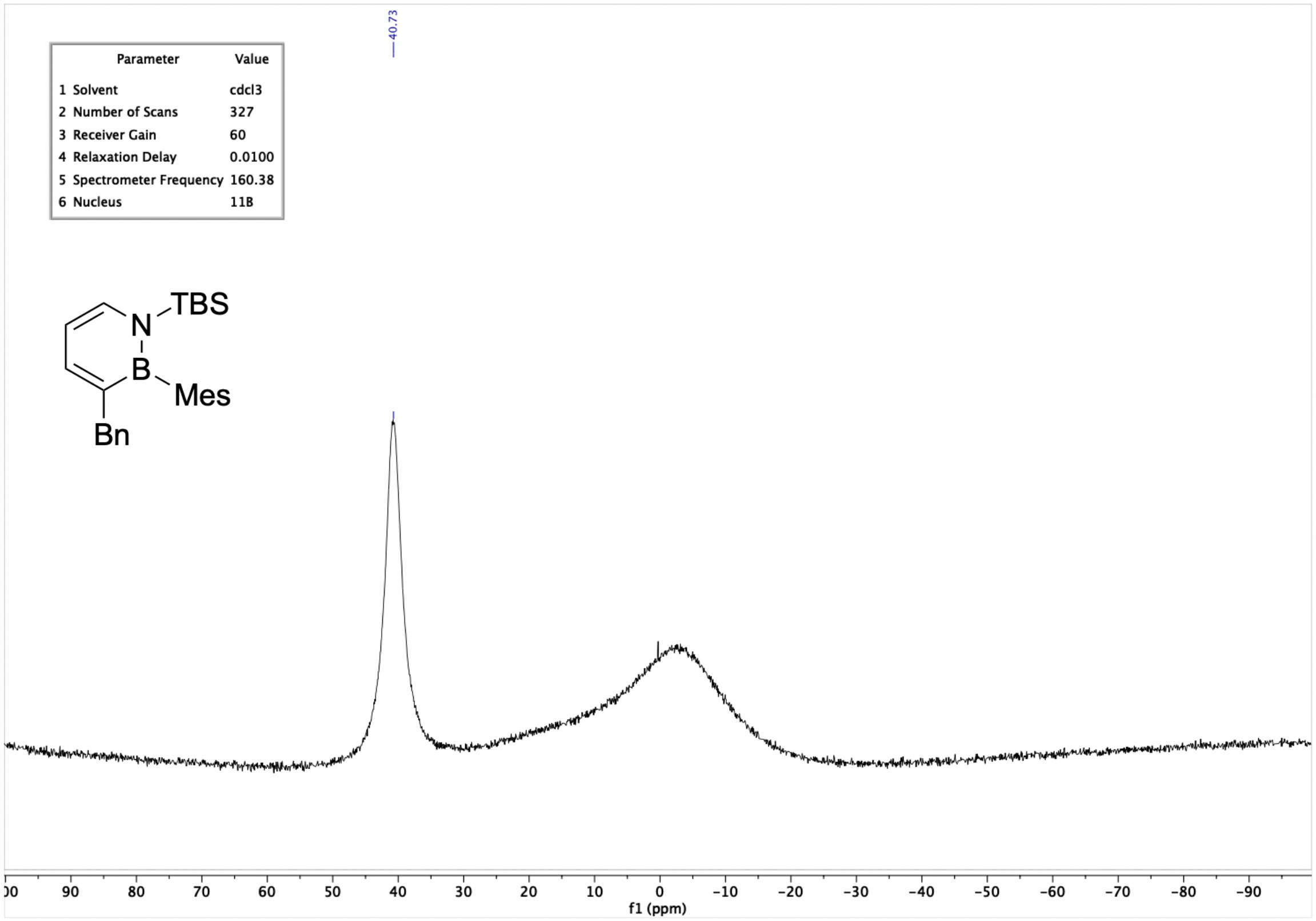
Parameter Value
1 Solvent cdcl3
2 Number of Scans 327
3 Receiver Gain 60
4 Relaxation Delay 0.0100
5 Spectrometer Frequency 160.38
6 Nucleus 11B
TBS
N
B
Mes
Bn
40.73
f1 (ppm)

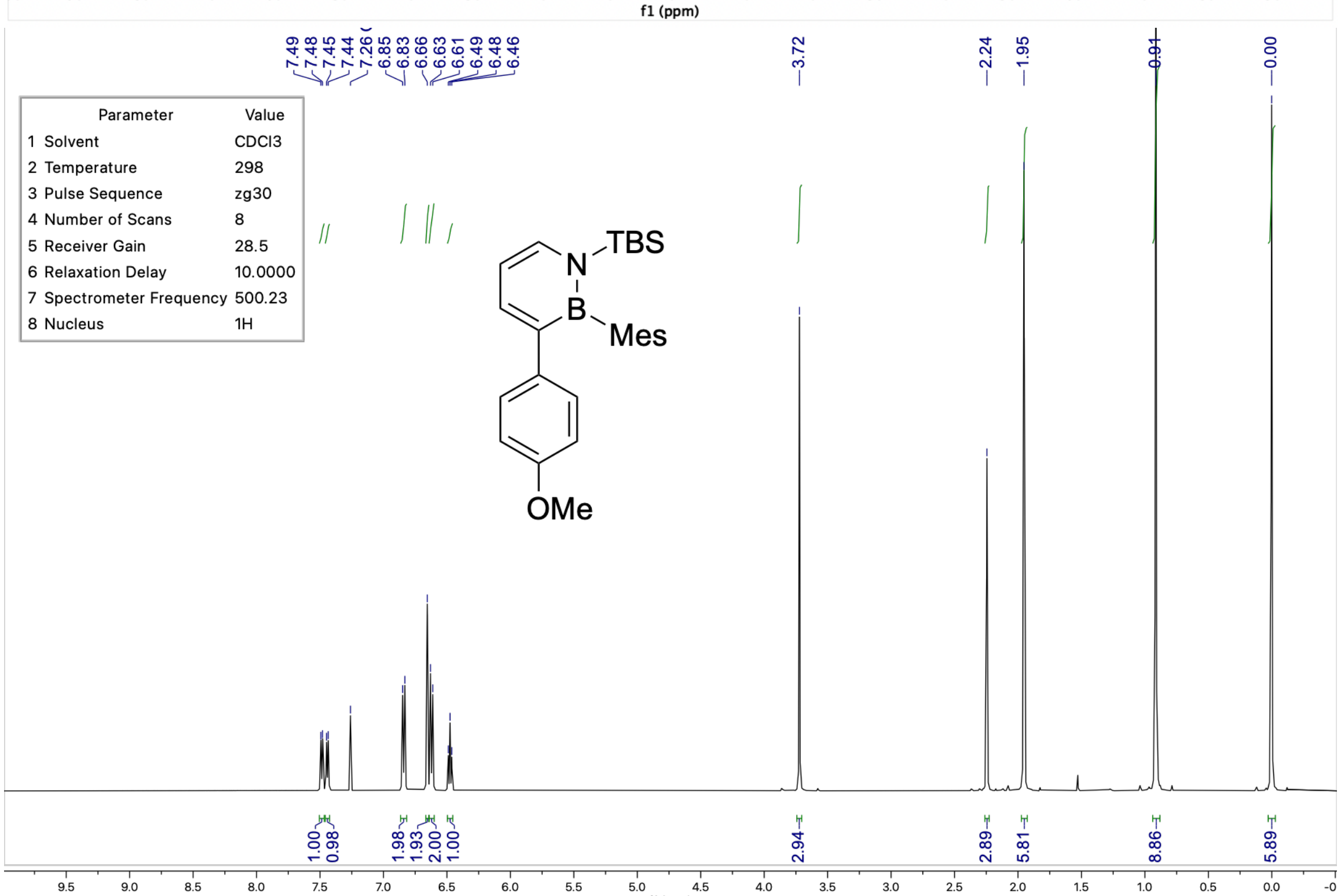
Parameter Value
1 Solvent CDCl3
2 Temperature 298
3 Pulse Sequence zg30
4 Number of Scans 8
5 Receiver Gain 28.5
6 Relaxation Delay 10.0000
7 Spectrometer Frequency 500.23
8 Nucleus 1H
TBS
N
B
Mes
OMe
7.49 7.48 7.45 7.44 7.26 6.85 6.83 6.66 6.63 6.61 6.49 6.48 6.46
3.72
2.24
1.95
0.91
0.00
1.00 0.98 1.98 1.93 2.00 1.00 2.94 2.89 5.81 8.86 5.89
f1 (ppm)

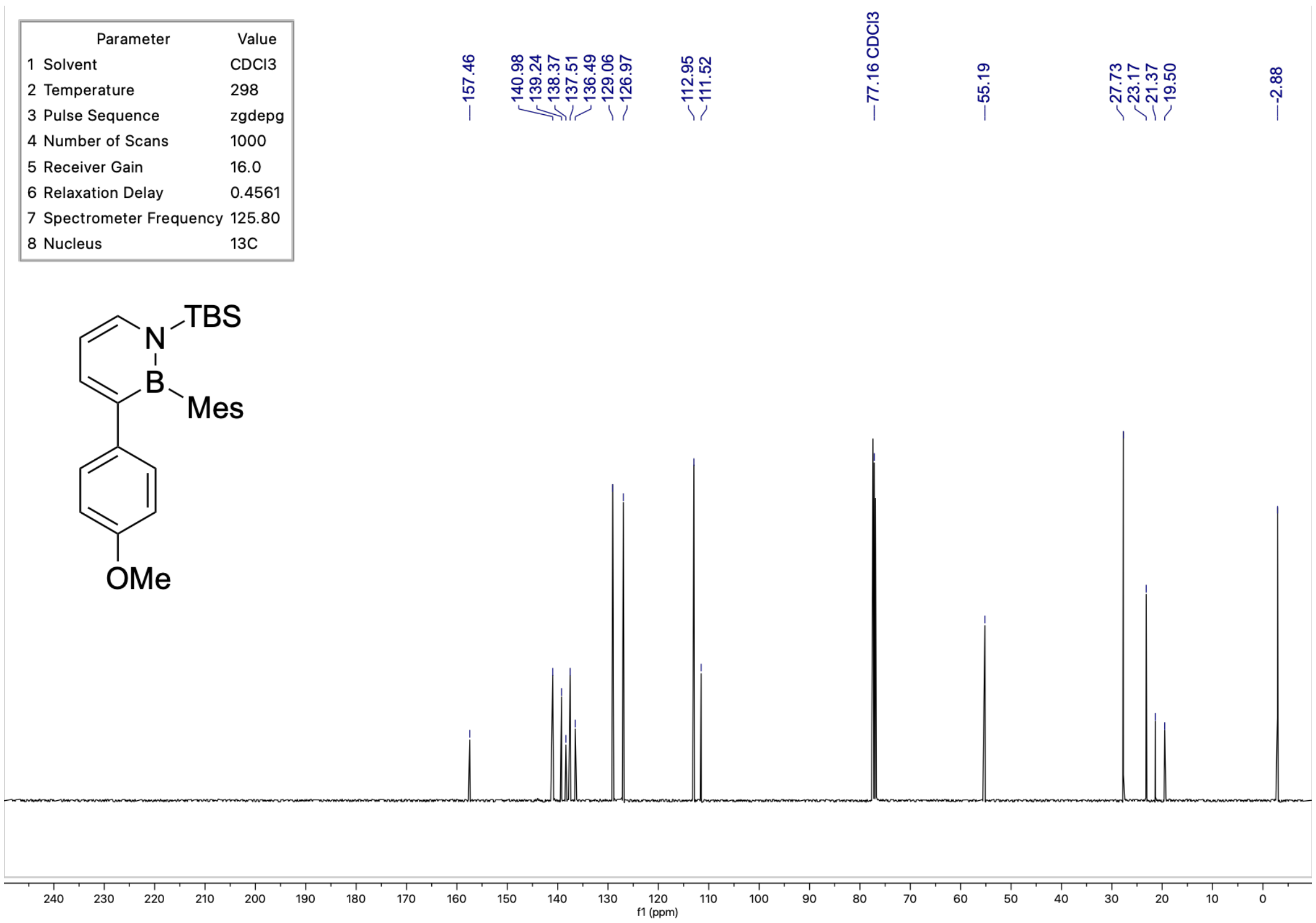
Parameter Value
1 Solvent CDCl3
2 Temperature 298
3 Pulse Sequence zgdepg
4 Number of Scans 1000
5 Receiver Gain 16.0
6 Relaxation Delay 0.4561
7 Spectrometer Frequency 125.80
8 Nucleus 13C
157.46
140.98
139.24
138.37
137.51
136.49
129.06
126.97
112.95
111.52
77.16 CDCl3
55.19
27.73
23.17
21.37
19.50
-2.88
N
TBS
B
Mes
OMe
f1 (ppm)

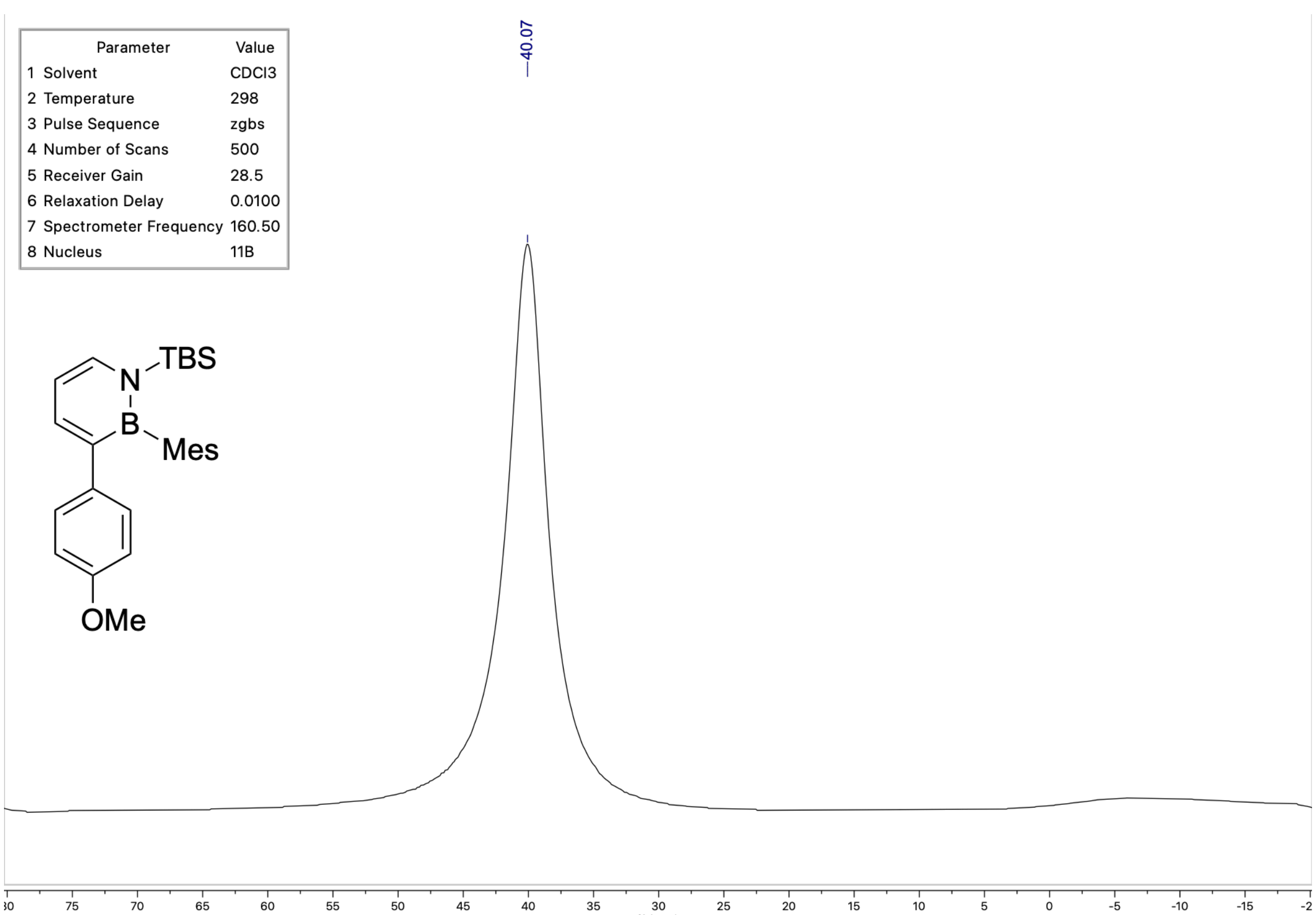
Parameter Value
1 Solvent CDCl3
2 Temperature 298
3 Pulse Sequence zgbs
4 Number of Scans 500
5 Receiver Gain 28.5
6 Relaxation Delay 0.0100
7 Spectrometer Frequency 160.50
8 Nucleus 11B
40.07
N
TBS
B
Mes
OMe
f1 (ppm)

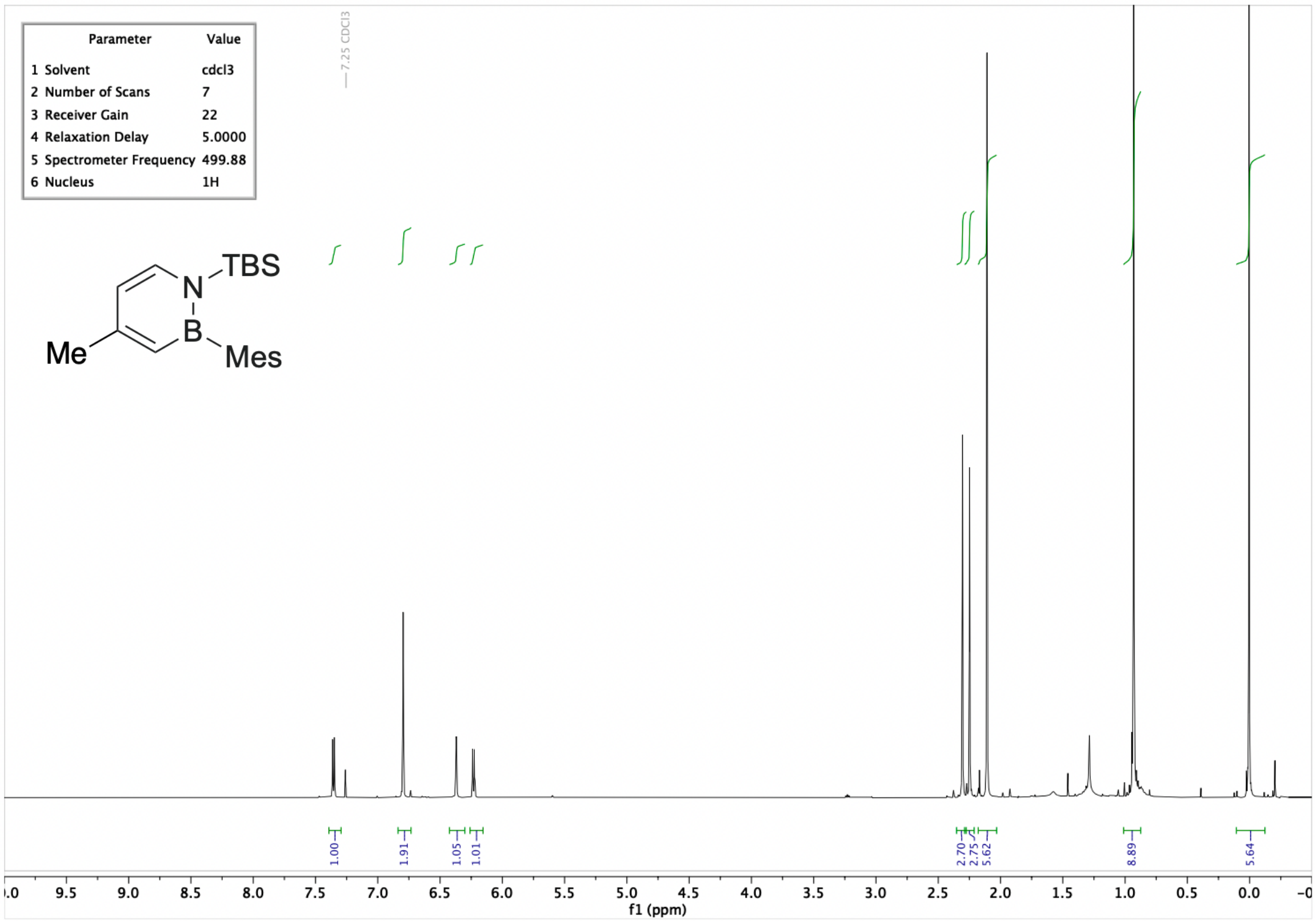

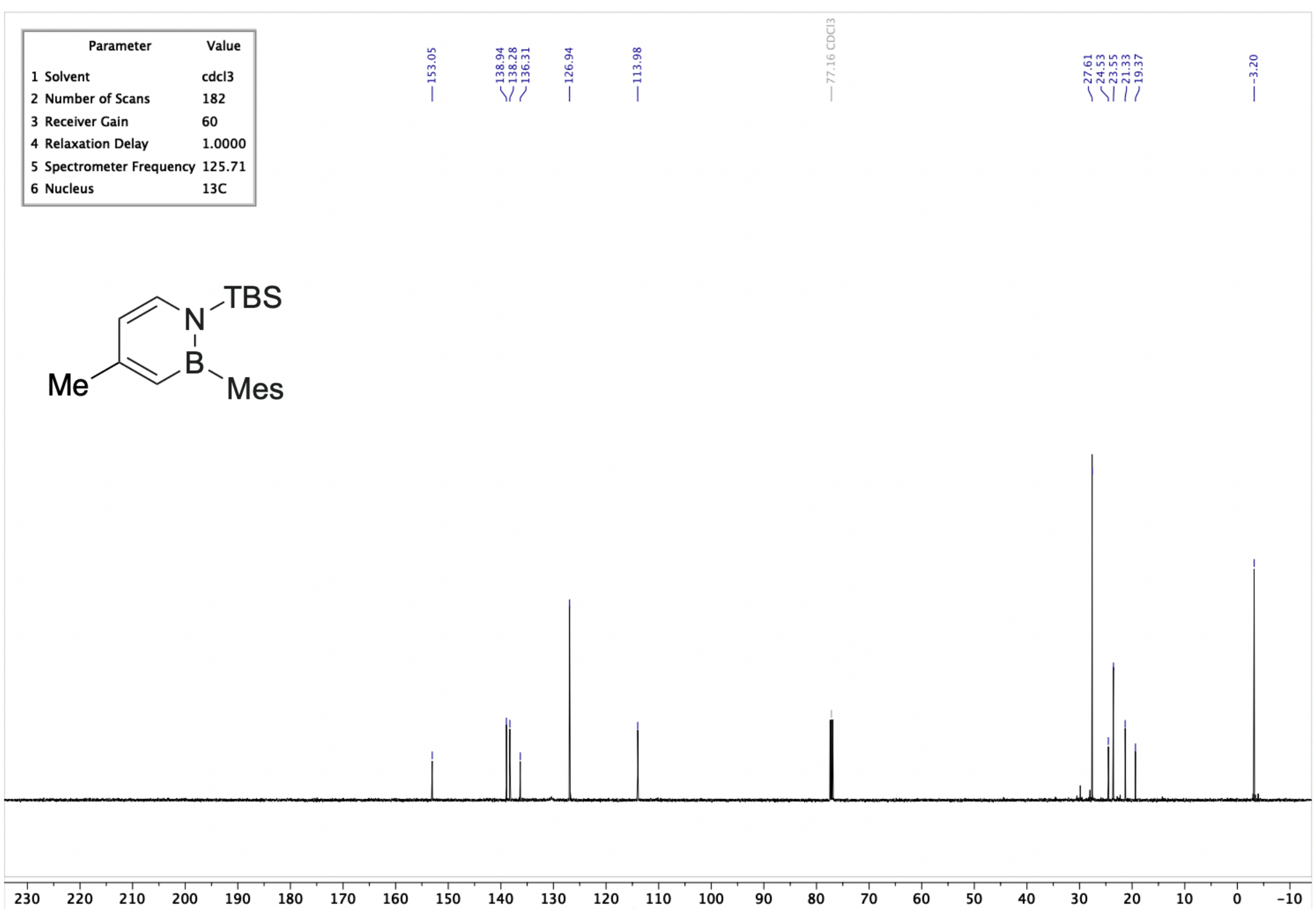

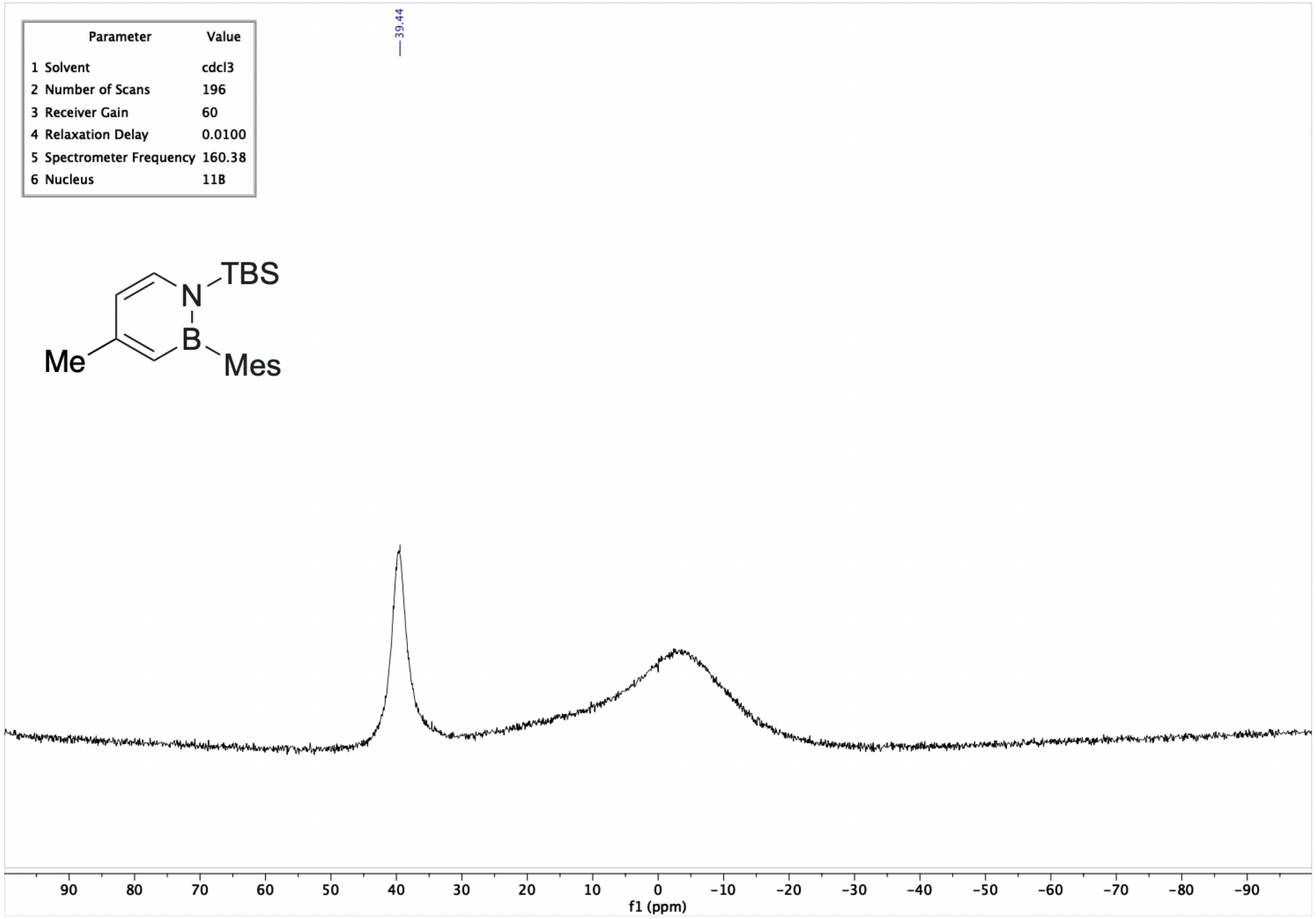

Parameter Value
1 Solvent cdcl3
2 Number of Scans 196
3 Receiver Gain 60
4 Relaxation Delay 0.0100
5 Spectrometer Frequency 160.38
6 Nucleus 11B
39.44
TBS
N
B
Me
Mes
90 80 70 60 50 40 30 20 10 0 −10 −20 −30 −40 −50 −60 −70 −80 −90
f1 (ppm)

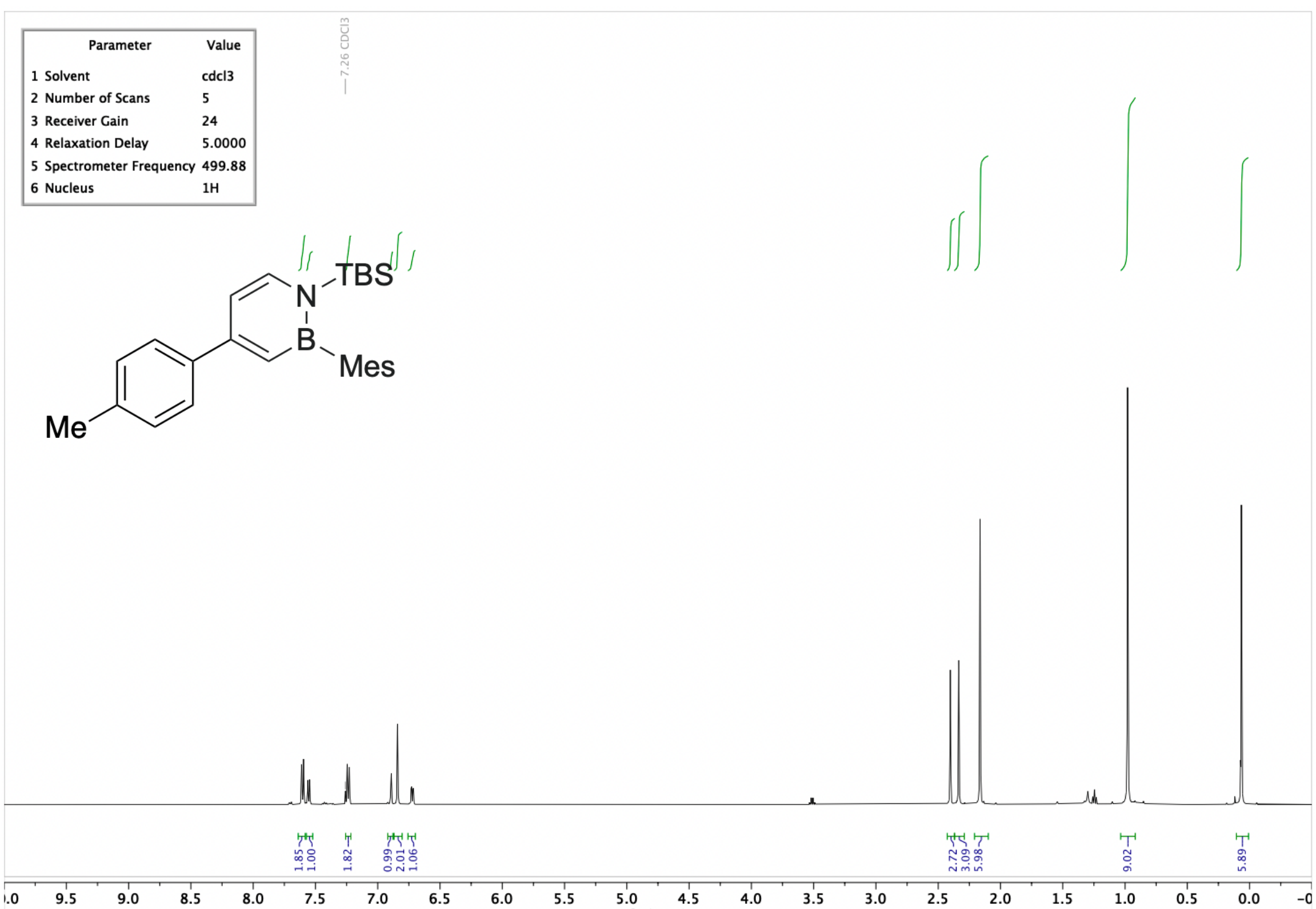

Parameter Value
1 Solvent cdcl3
2 Number of Scans 5
3 Receiver Gain 24
4 Relaxation Delay 5.0000
5 Spectrometer Frequency 499.88
6 Nucleus 1H
7.26 CDCl3
TBS
N
B
Mes
Me
1.85
1.00
1.82
0.99
2.01
1.06
2.72
3.09
5.98
9.02
5.89
9.5 9.0 8.5 8.0 7.5 7.0 6.5 6.0 5.5 5.0 4.5 4.0 3.5 3.0 2.5 2.0 1.5 1.0 0.5 0.0
f1 (ppm)

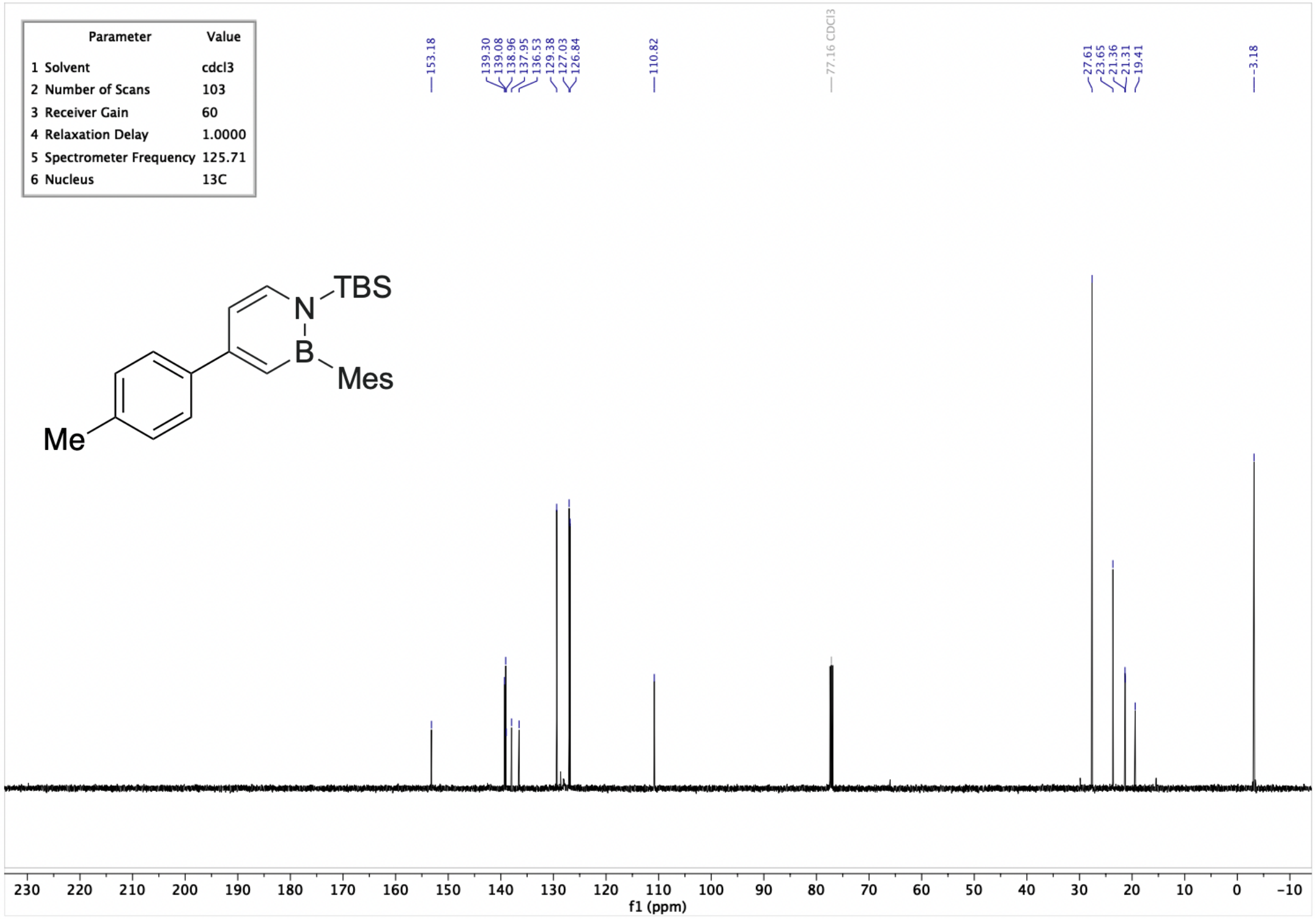

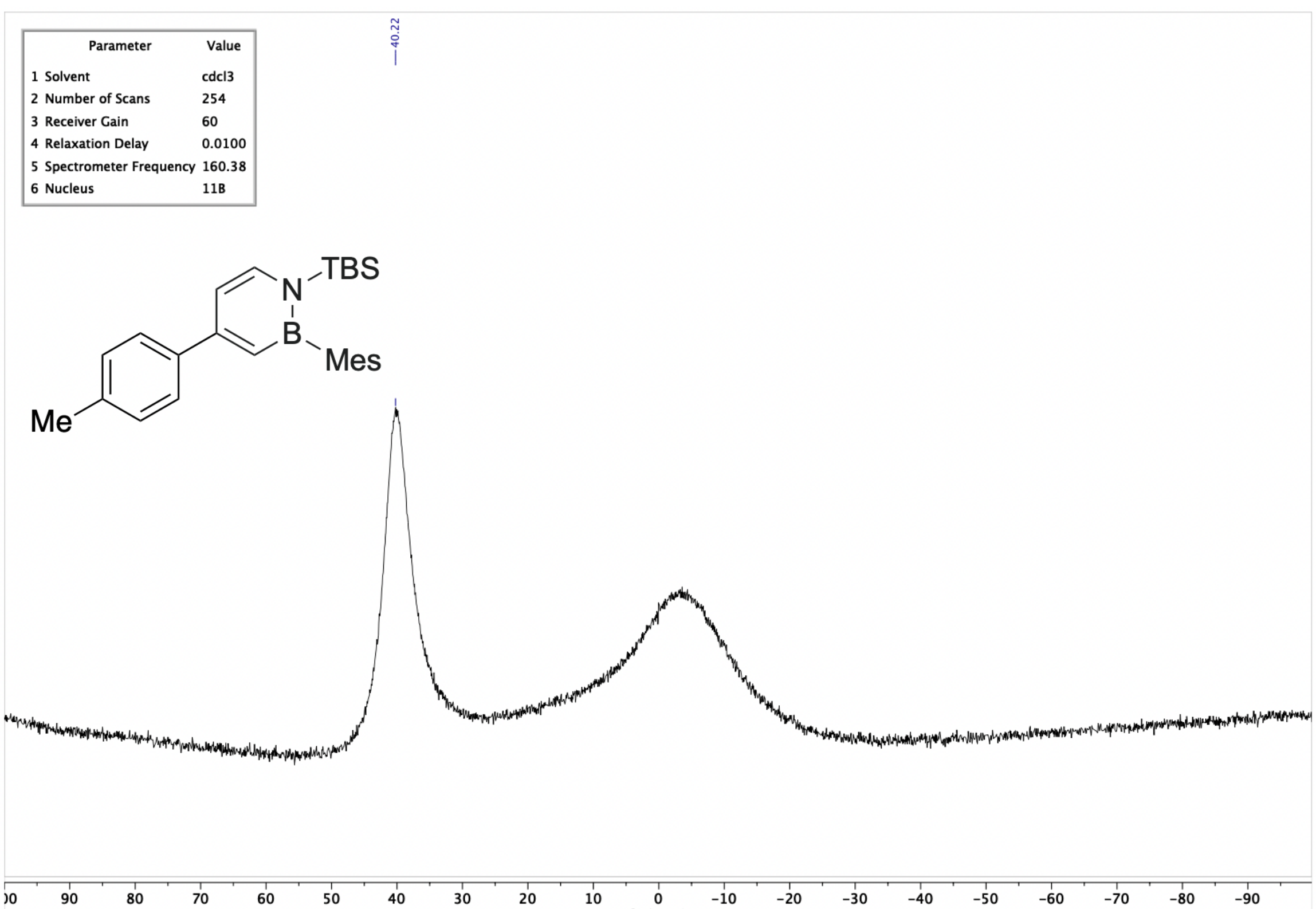

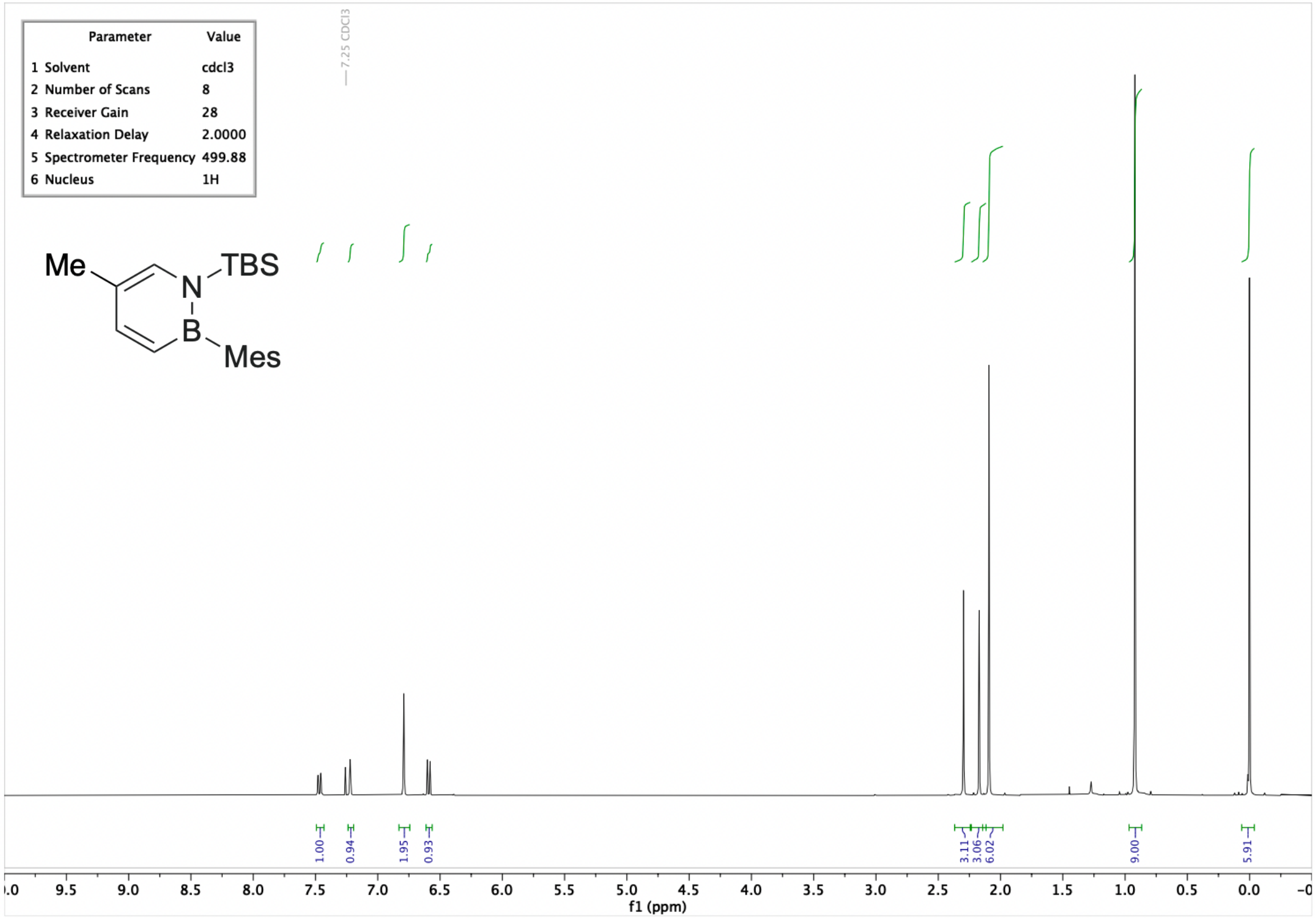
Parameter Value
1 Solvent cdcl3
2 Number of Scans 8
3 Receiver Gain 28
4 Relaxation Delay 2.0000
5 Spectrometer Frequency 499.88
6 Nucleus 1H
Me
N
TBS
B
Mes
f1 (ppm)

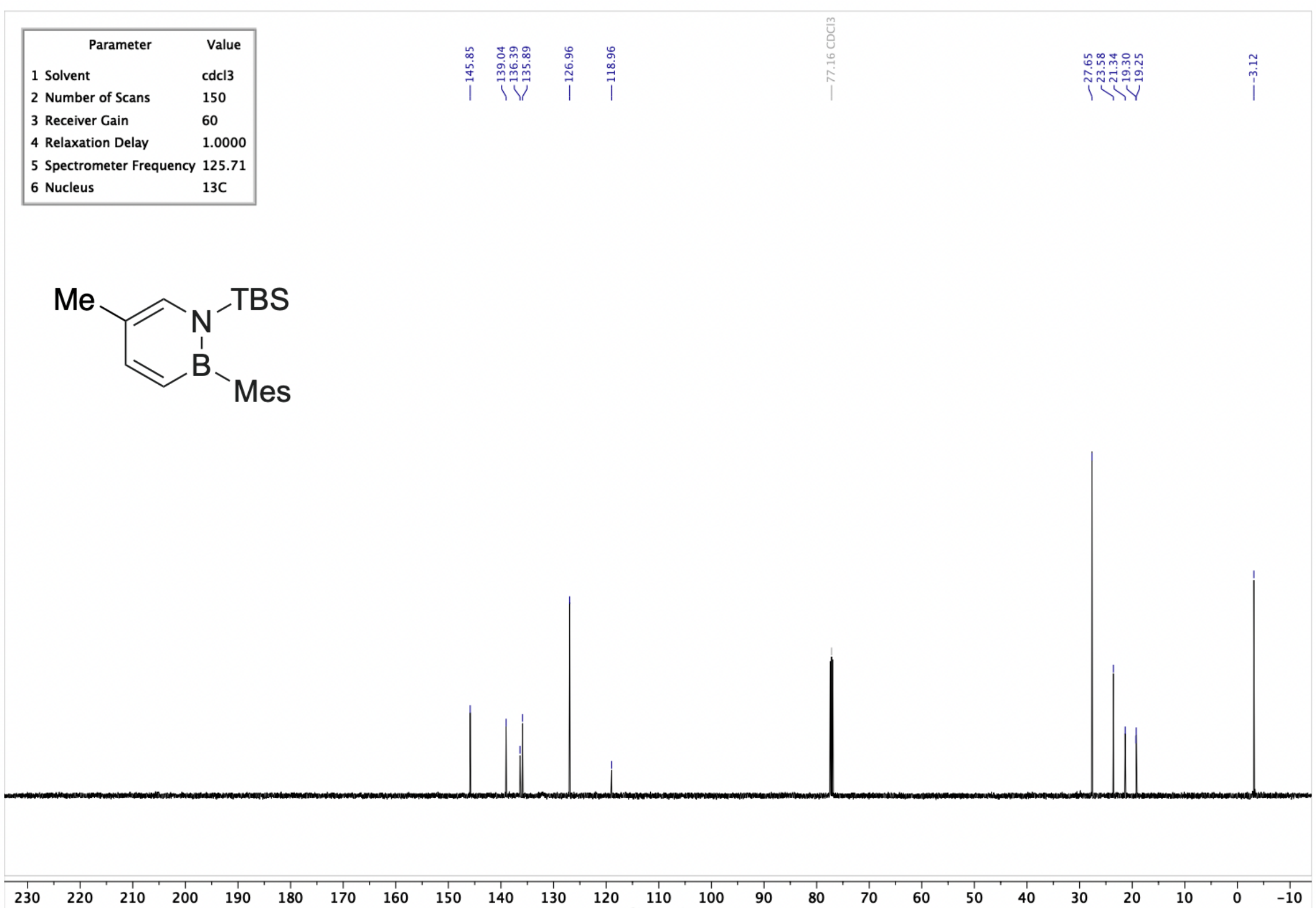
Parameter Value
1 Solvent cdcl3
2 Number of Scans 150
3 Receiver Gain 60
4 Relaxation Delay 1.0000
5 Spectrometer Frequency 125.71
6 Nucleus 13C
145.85
139.04
136.39
135.89
126.96
118.96
77.16 CDCl3
27.65
23.58
21.34
19.30
19.25
-3.12
Me
N
TBS
B
Mes
f1 (ppm)

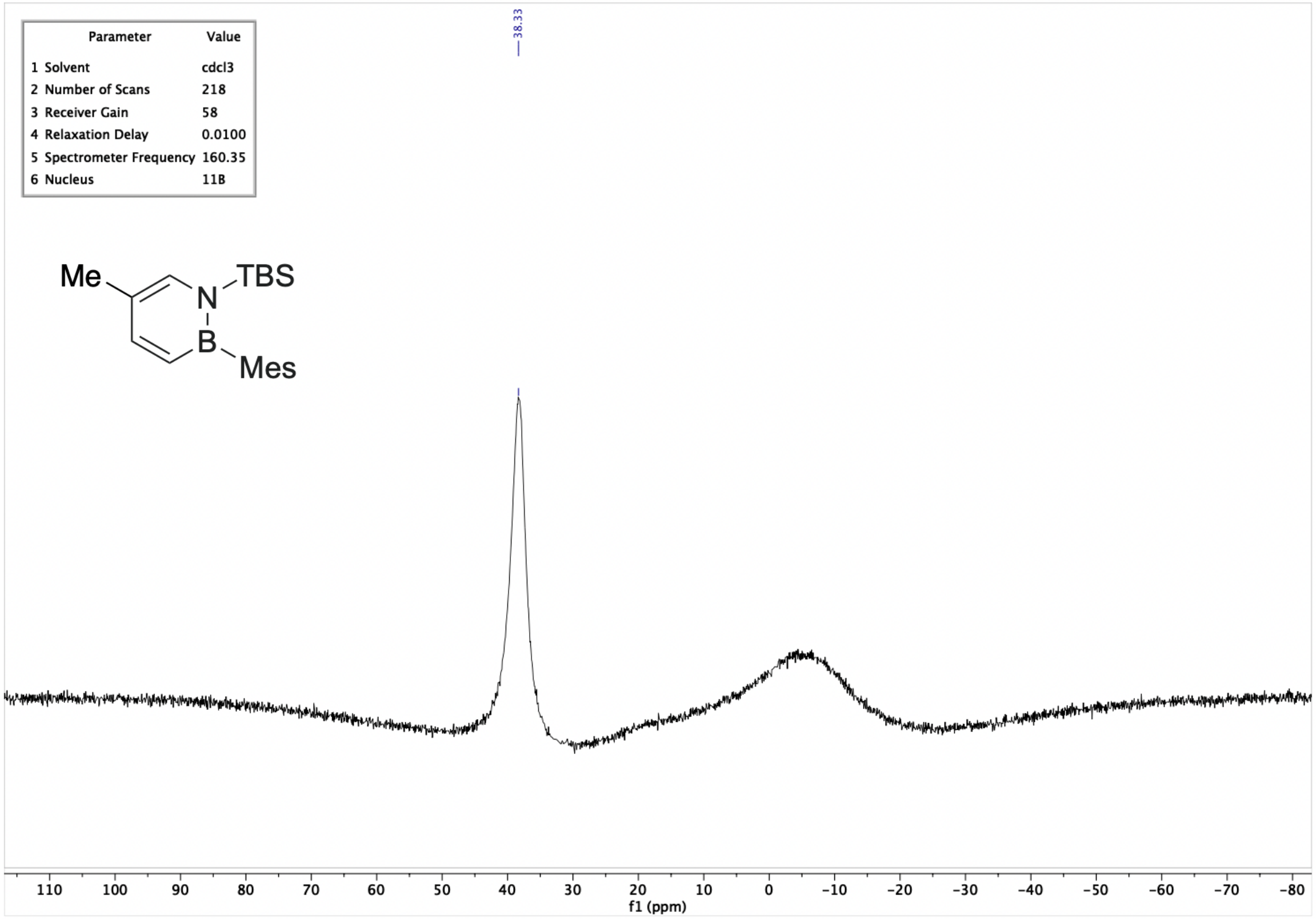
Parameter Value
1 Solvent cdcl3
2 Number of Scans 218
3 Receiver Gain 58
4 Relaxation Delay 0.0100
5 Spectrometer Frequency 160.35
6 Nucleus 11B
38.33
Me
N
TBS
B
Mes
f1 (ppm)

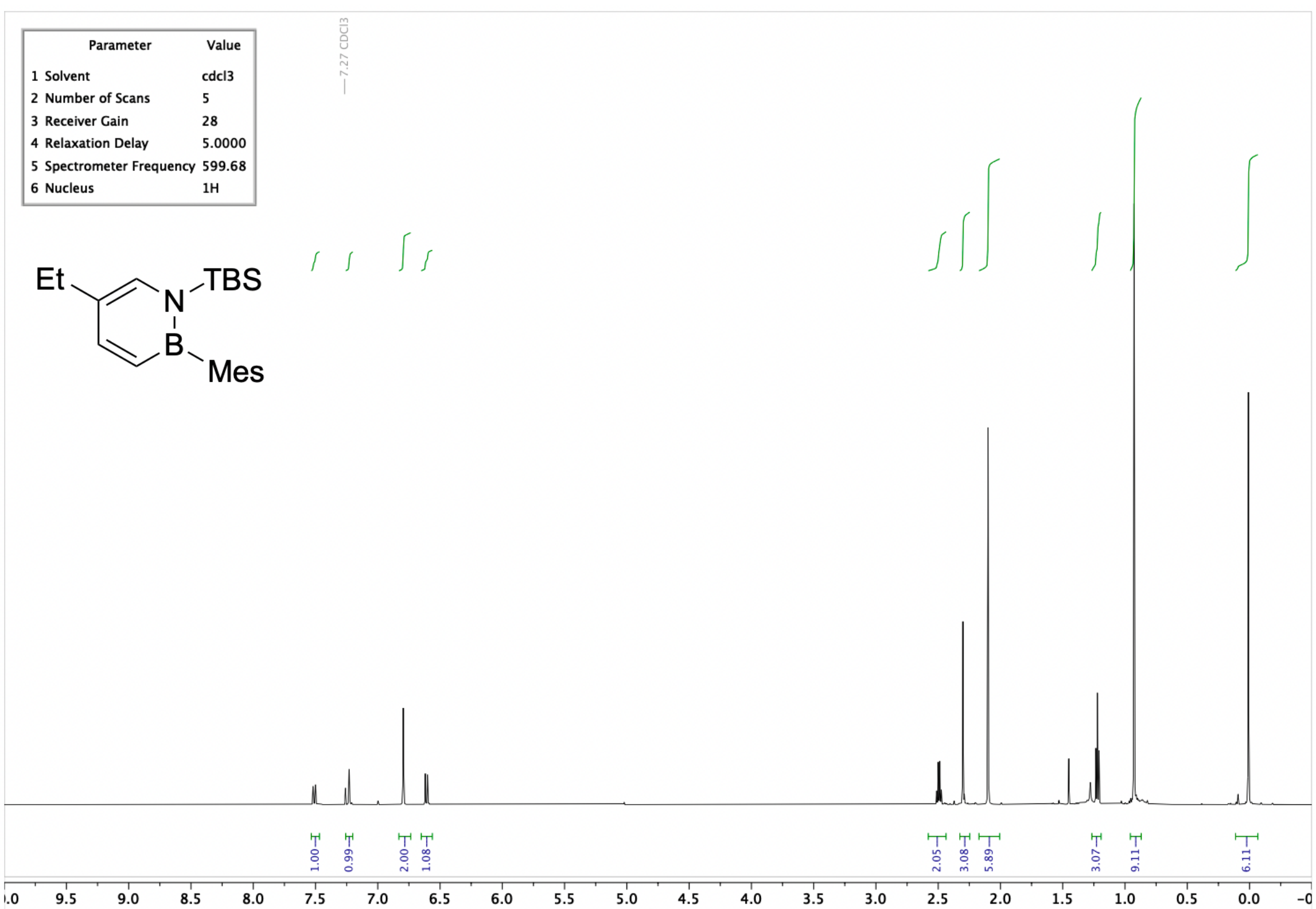
Parameter Value
1 Solvent cdcl3
2 Number of Scans 5
3 Receiver Gain 28
4 Relaxation Delay 5.0000
5 Spectrometer Frequency 599.68
6 Nucleus 1H
7.27 CDCl3
Et
N
TBS
B
Mes
1.00
0.99
2.00
1.08
2.05
3.08
5.89
3.07
9.11
6.11
f1 (ppm)

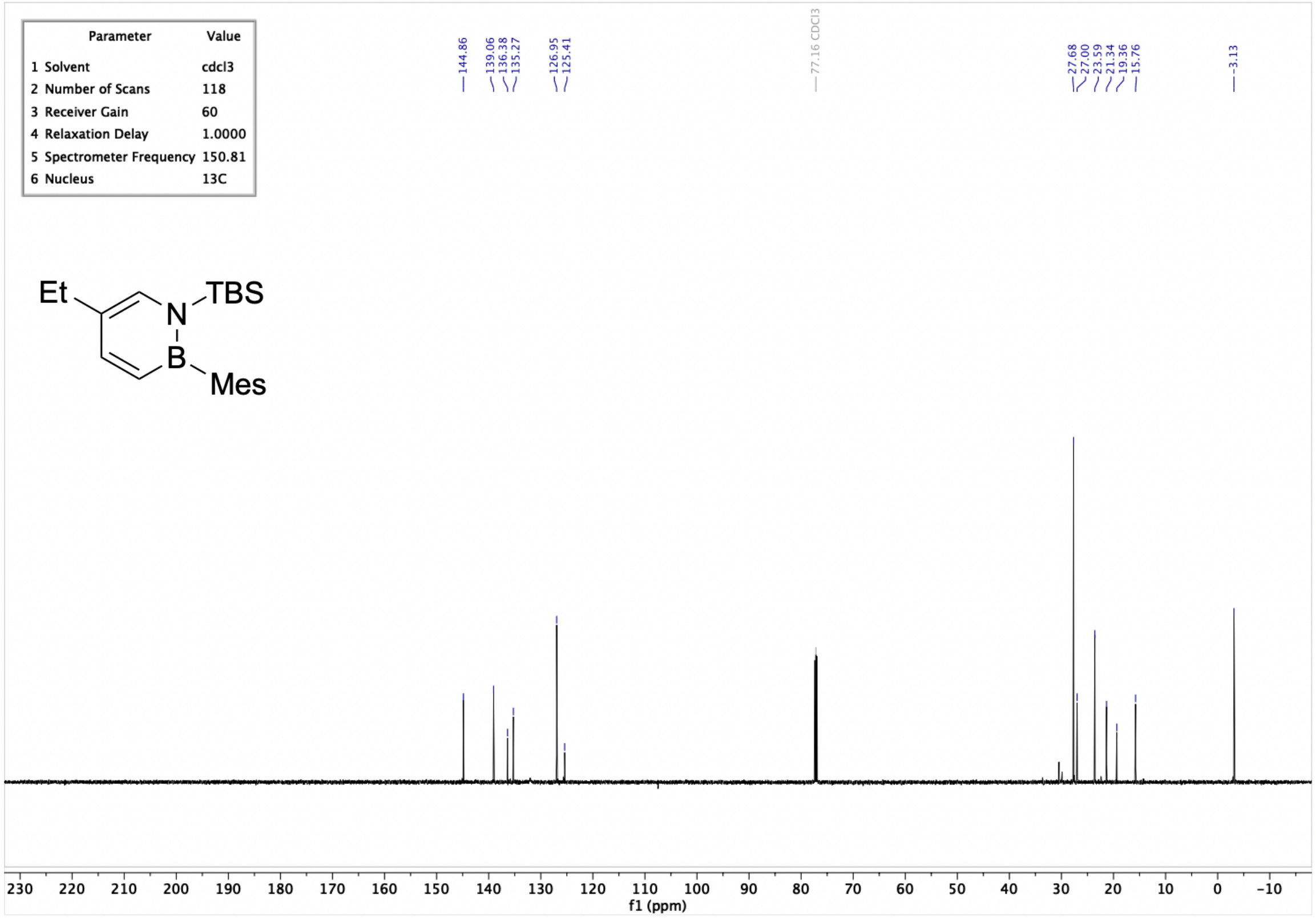
Parameter Value
1 Solvent cdcl3
2 Number of Scans 118
3 Receiver Gain 60
4 Relaxation Delay 1.0000
5 Spectrometer Frequency 150.81
6 Nucleus 13C
144.86
139.06
136.38
135.27
126.95
125.41
77.16 CDCl3
27.68
27.00
23.59
21.34
19.36
15.76
-3.13
Et
N
TBS
B
Mes
f1 (ppm)

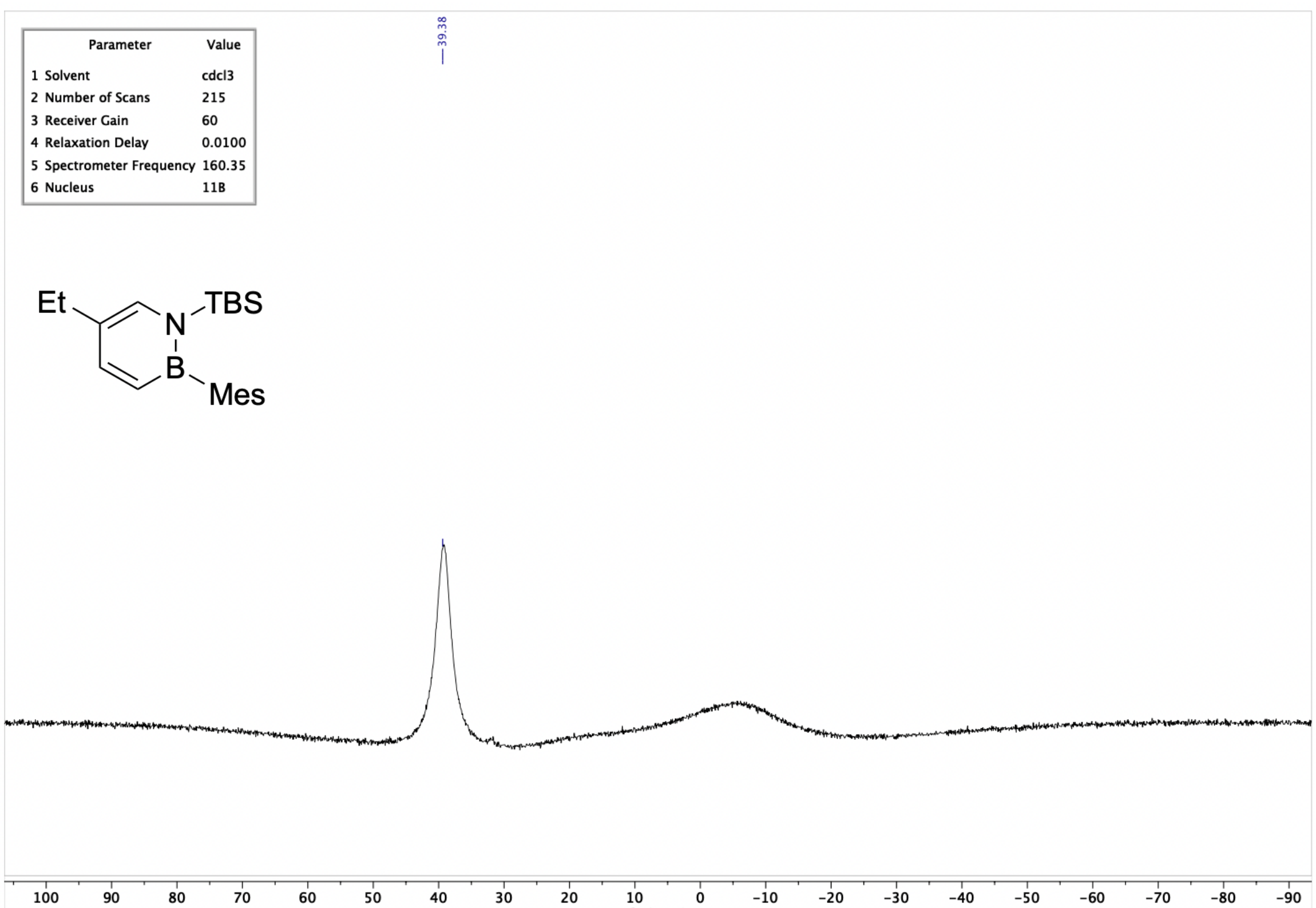
Parameter Value
1 Solvent cdcl3
2 Number of Scans 215
3 Receiver Gain 60
4 Relaxation Delay 0.0100
5 Spectrometer Frequency 160.35
6 Nucleus 11B
39.38
Et
N
TBS
B
Mes
f1 (ppm)

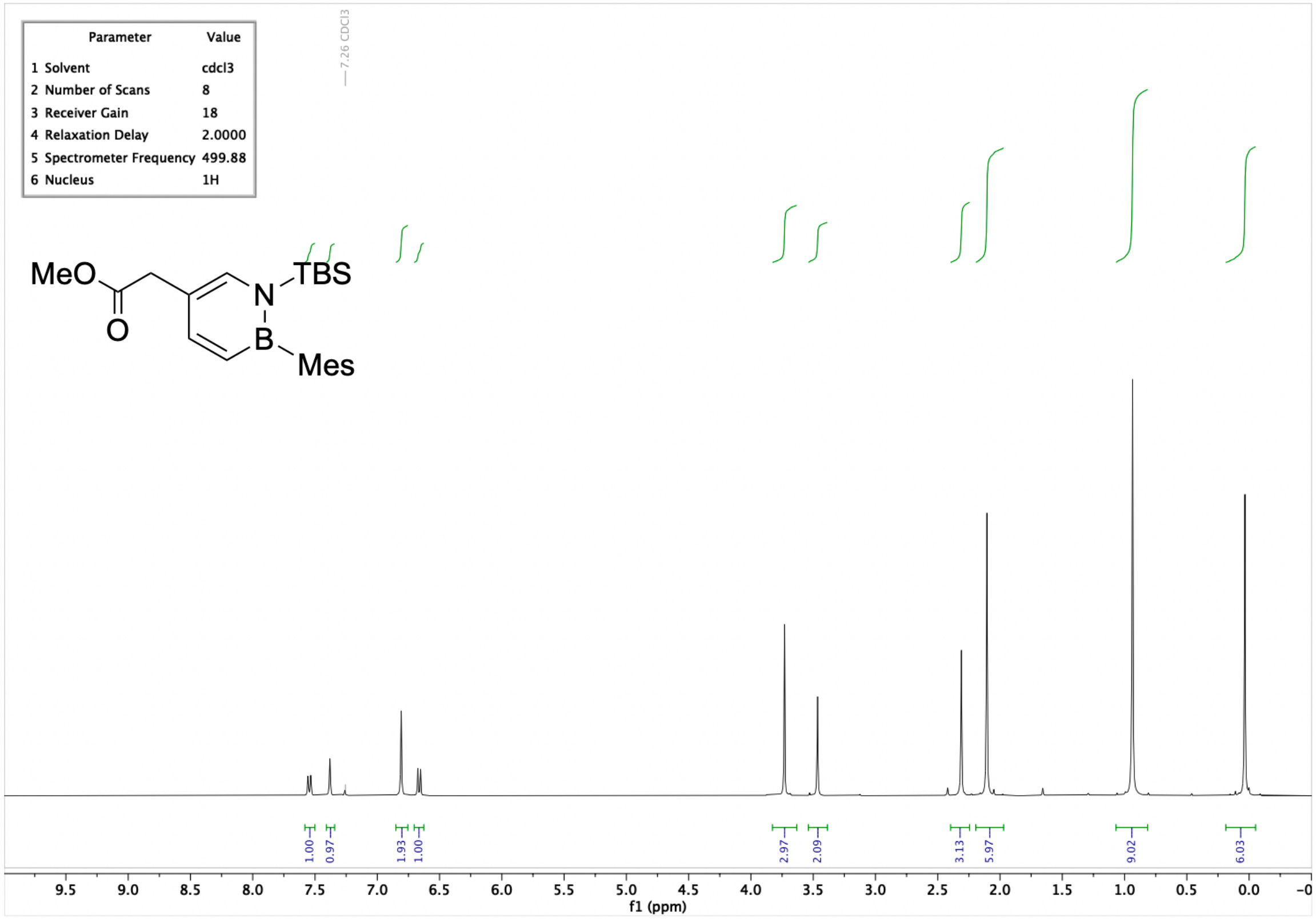
Parameter Value
1 Solvent cdcl3
2 Number of Scans 8
3 Receiver Gain 18
4 Relaxation Delay 2.0000
5 Spectrometer Frequency 499.88
6 Nucleus 1H
MeO
O
N
TBS
B
Mes
f1 (ppm)

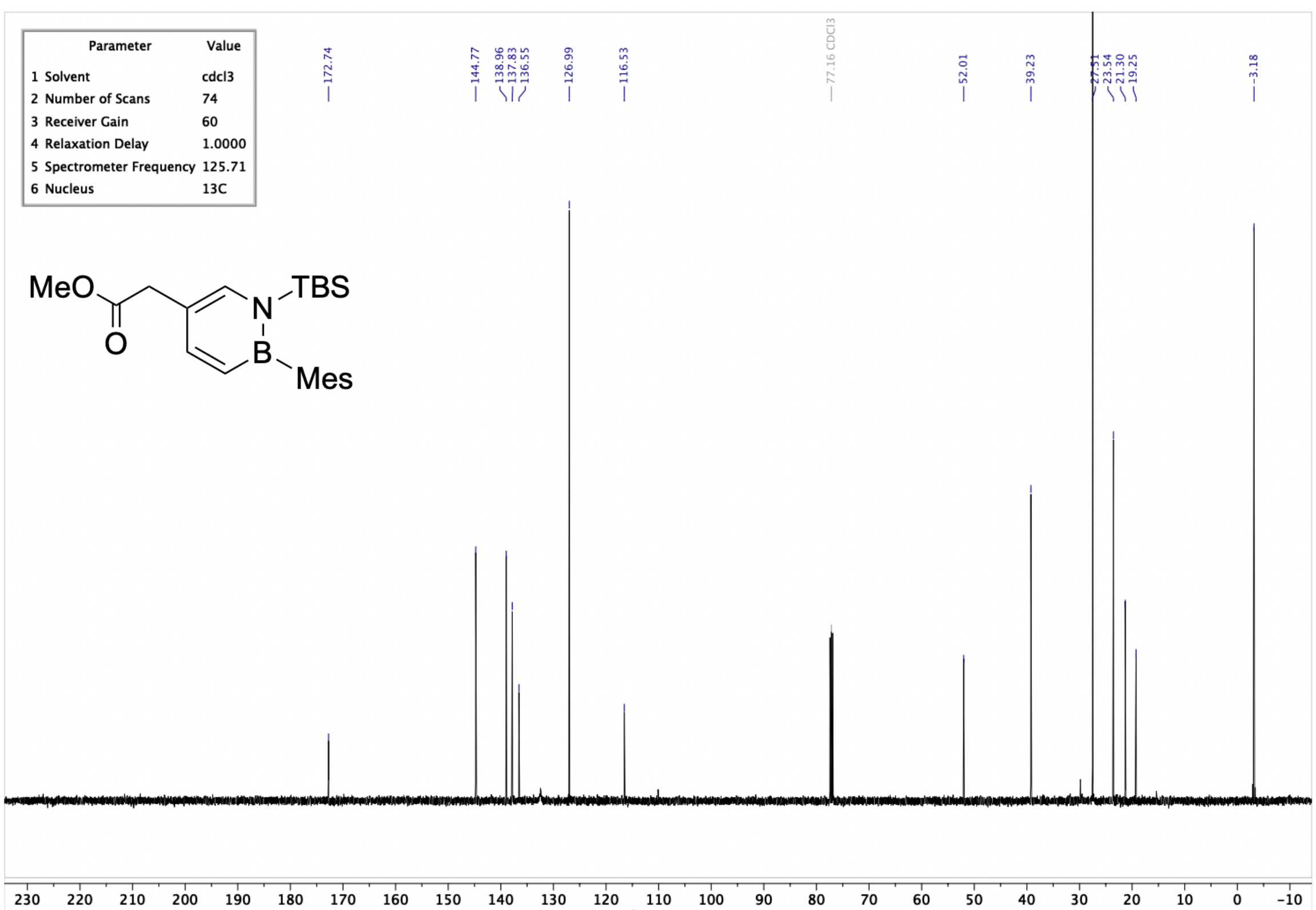
Parameter Value
1 Solvent cdcl3
2 Number of Scans 74
3 Receiver Gain 60
4 Relaxation Delay 1.0000
5 Spectrometer Frequency 125.71
6 Nucleus 13C
MeO
O
N
TBS
B
Mes
172.74
144.77
138.96
137.83
136.55
126.99
116.53
52.01
39.23
23.54
21.30
19.25
-3.18
f1 (ppm)

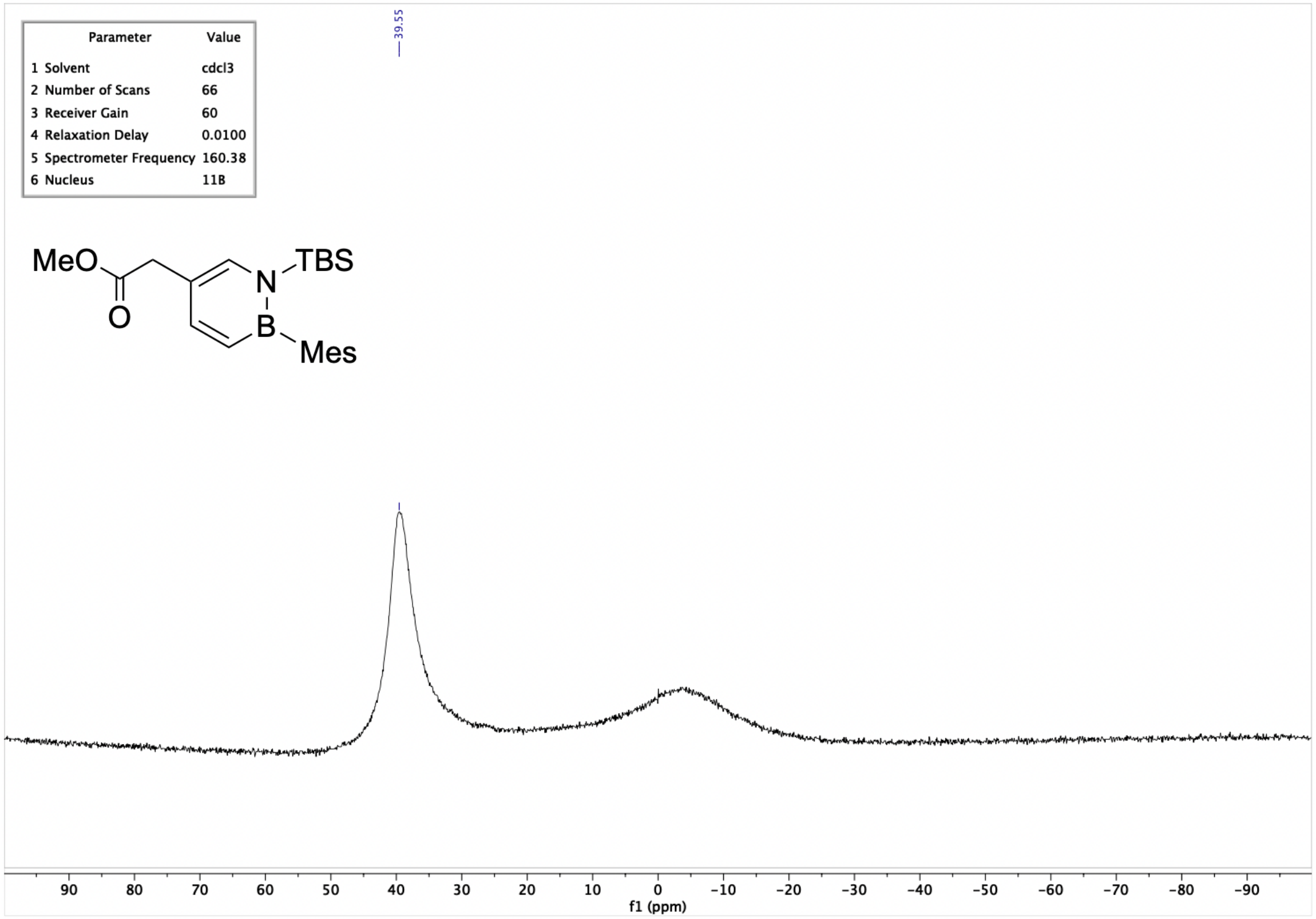

Parameter Value
1 Solvent cdcl3
2 Number of Scans 66
3 Receiver Gain 60
4 Relaxation Delay 0.0100
5 Spectrometer Frequency 160.38
6 Nucleus 11B
MeO
O
N
TBS
B
Mes
39.55
f1 (ppm)

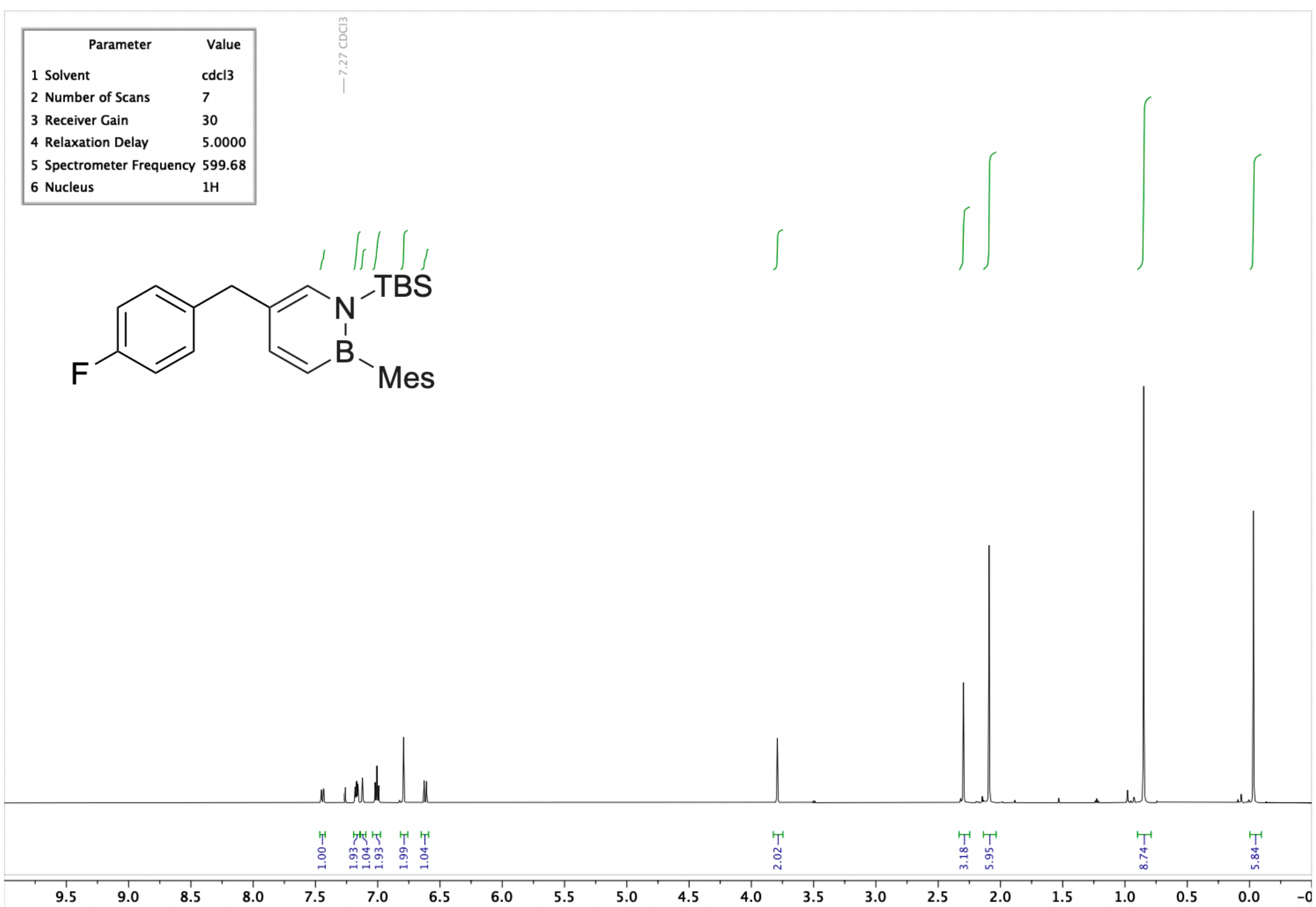

Parameter Value
1 Solvent cdcl3
2 Number of Scans 7
3 Receiver Gain 30
4 Relaxation Delay 5.0000
5 Spectrometer Frequency 599.68
6 Nucleus 1H
7.27 CDCl3
F
N
TBS
B
Mes
1.00
1.93
1.04
1.93
1.99
1.04
2.02
3.18
5.95
8.74
5.84
f1 (ppm)

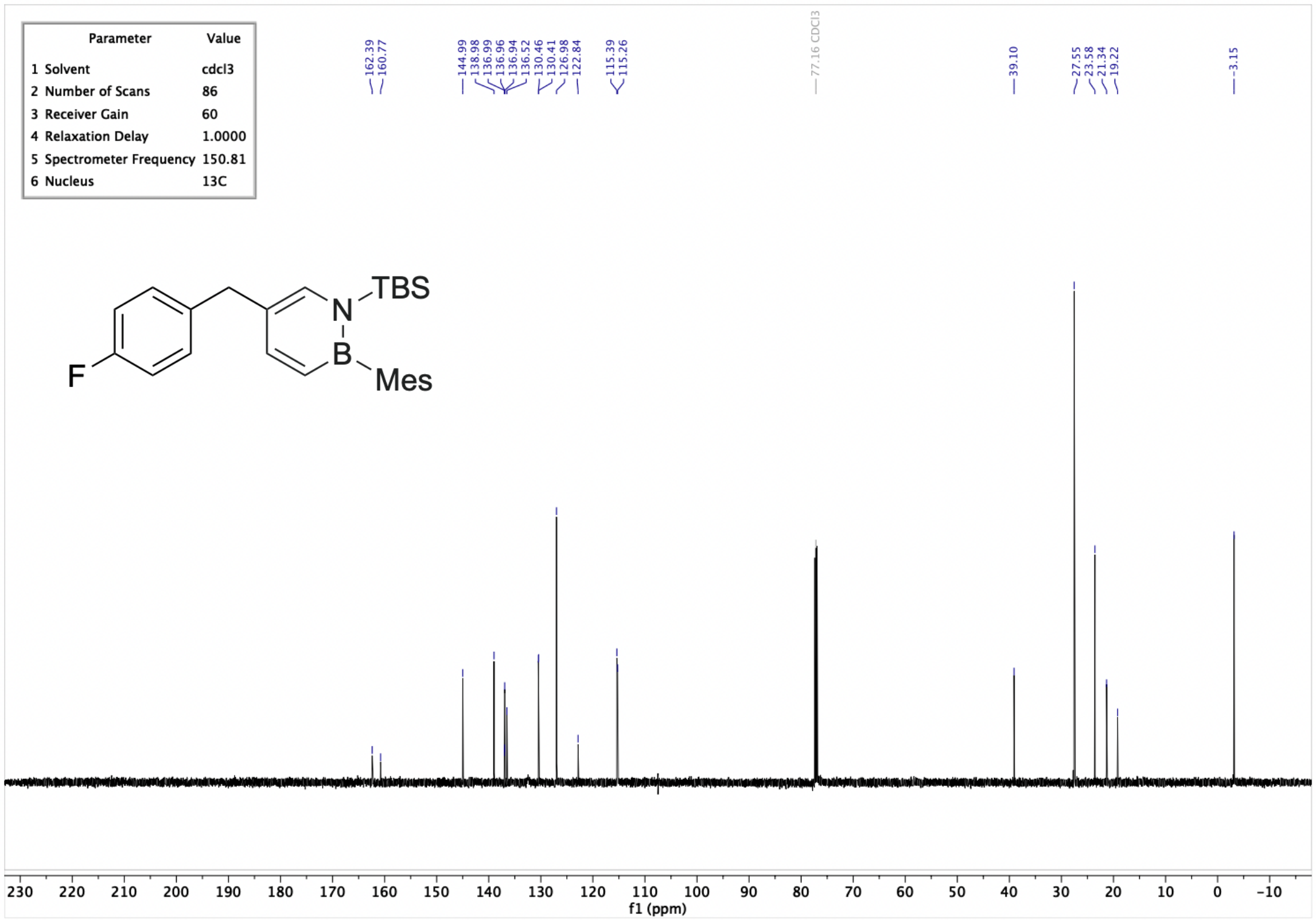
Parameter Value
1 Solvent cdcl3
2 Number of Scans 86
3 Receiver Gain 60
4 Relaxation Delay 1.0000
5 Spectrometer Frequency 150.81
6 Nucleus 13C
162.39
160.77
144.99
138.98
136.99
136.96
136.94
136.52
130.46
130.41
126.98
122.84
115.39
115.26
77.16 CDCl3
39.10
27.55
23.58
21.34
19.22
-3.15
TBS
N
B
Mes
F
f1 (ppm)

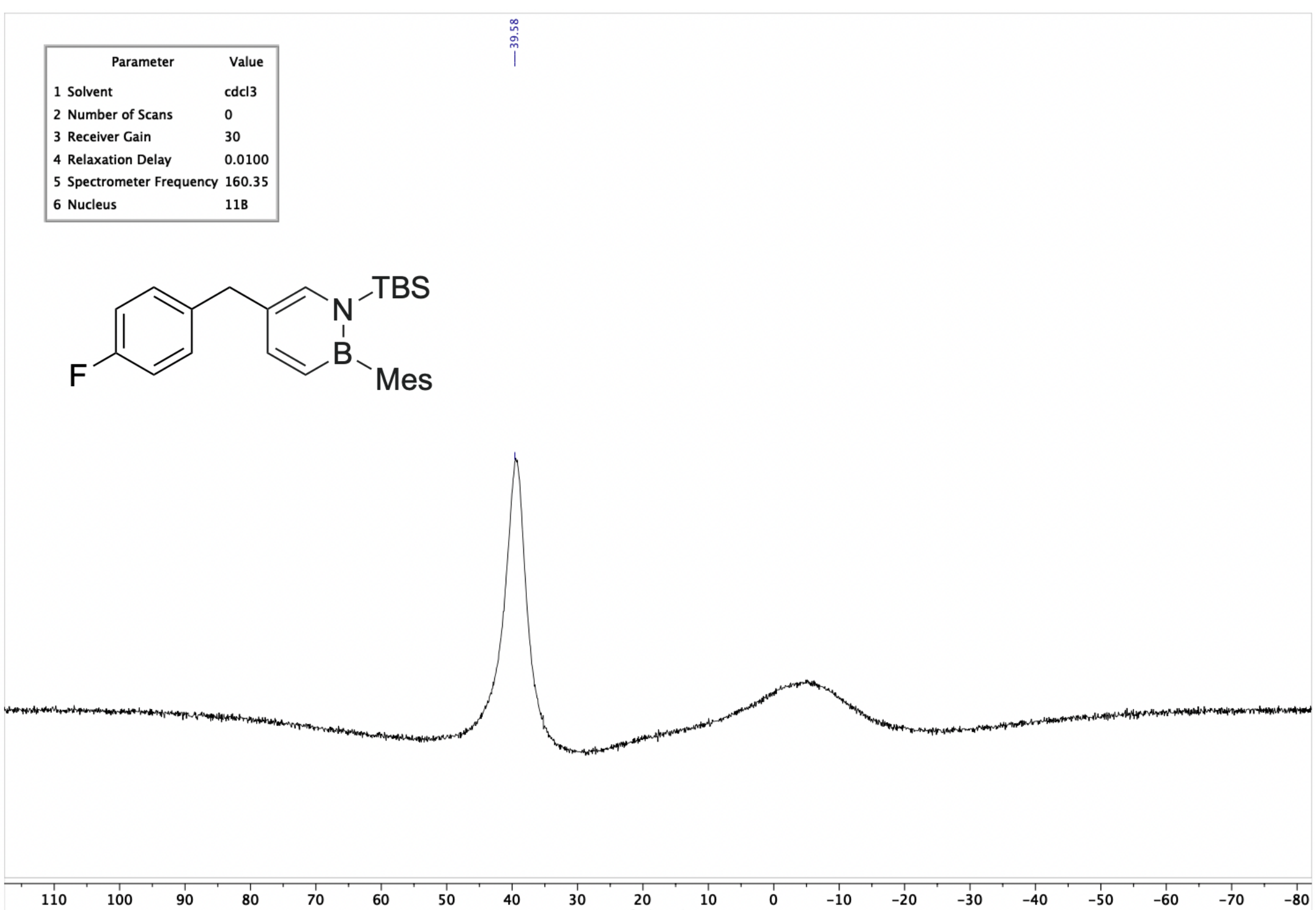
Parameter Value
1 Solvent cdcl3
2 Number of Scans 0
3 Receiver Gain 30
4 Relaxation Delay 0.0100
5 Spectrometer Frequency 160.35
6 Nucleus 11B
39.58
TBS
N
B
Mes
F
f1 (ppm)

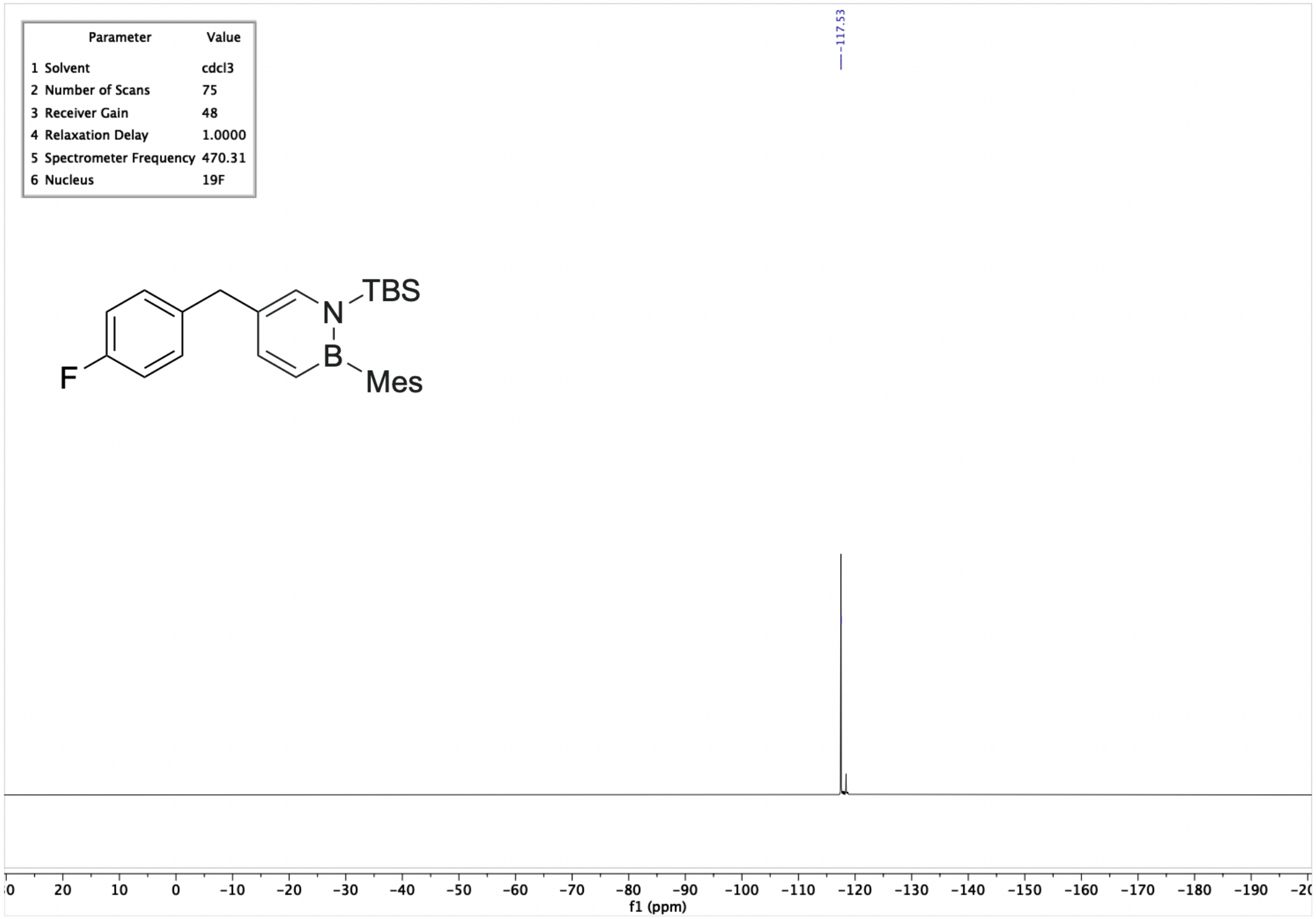

Parameter Value
1 Solvent cdcl3
2 Number of Scans 75
3 Receiver Gain 48
4 Relaxation Delay 1.0000
5 Spectrometer Frequency 470.31
6 Nucleus 19F
TBS
N
B
Mes
F
-117.53
f1 (ppm)

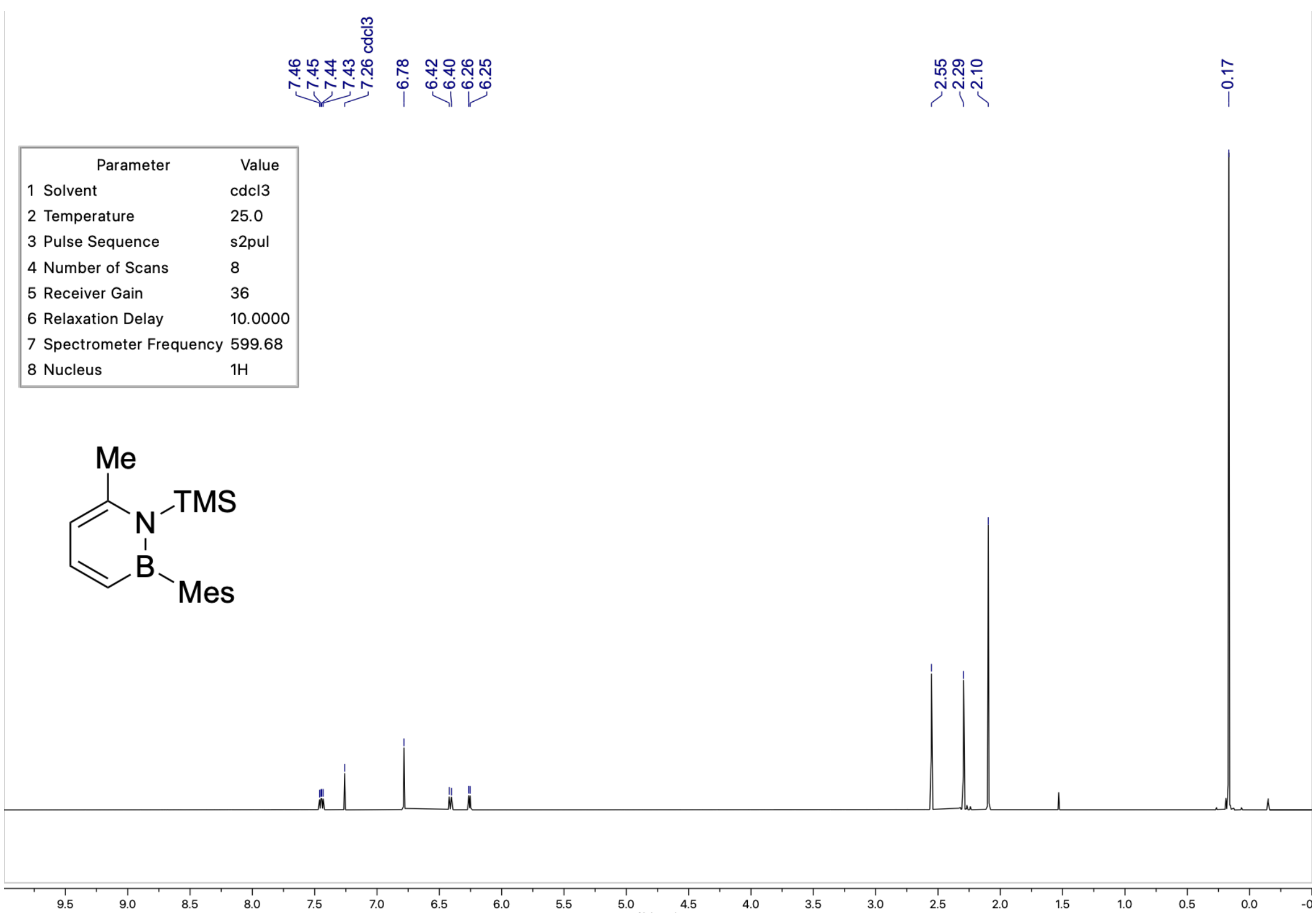

7.46
7.45
7.44
7.43
7.26 cdcl3
6.78
6.42
6.40
6.26
6.25
2.55
2.29
2.10
0.17
Parameter Value
1 Solvent cdcl3
2 Temperature 25.0
3 Pulse Sequence s2pul
4 Number of Scans 8
5 Receiver Gain 36
6 Relaxation Delay 10.0000
7 Spectrometer Frequency 599.68
8 Nucleus 1H
Me
TMS
N
B
Mes
f1 (ppm)

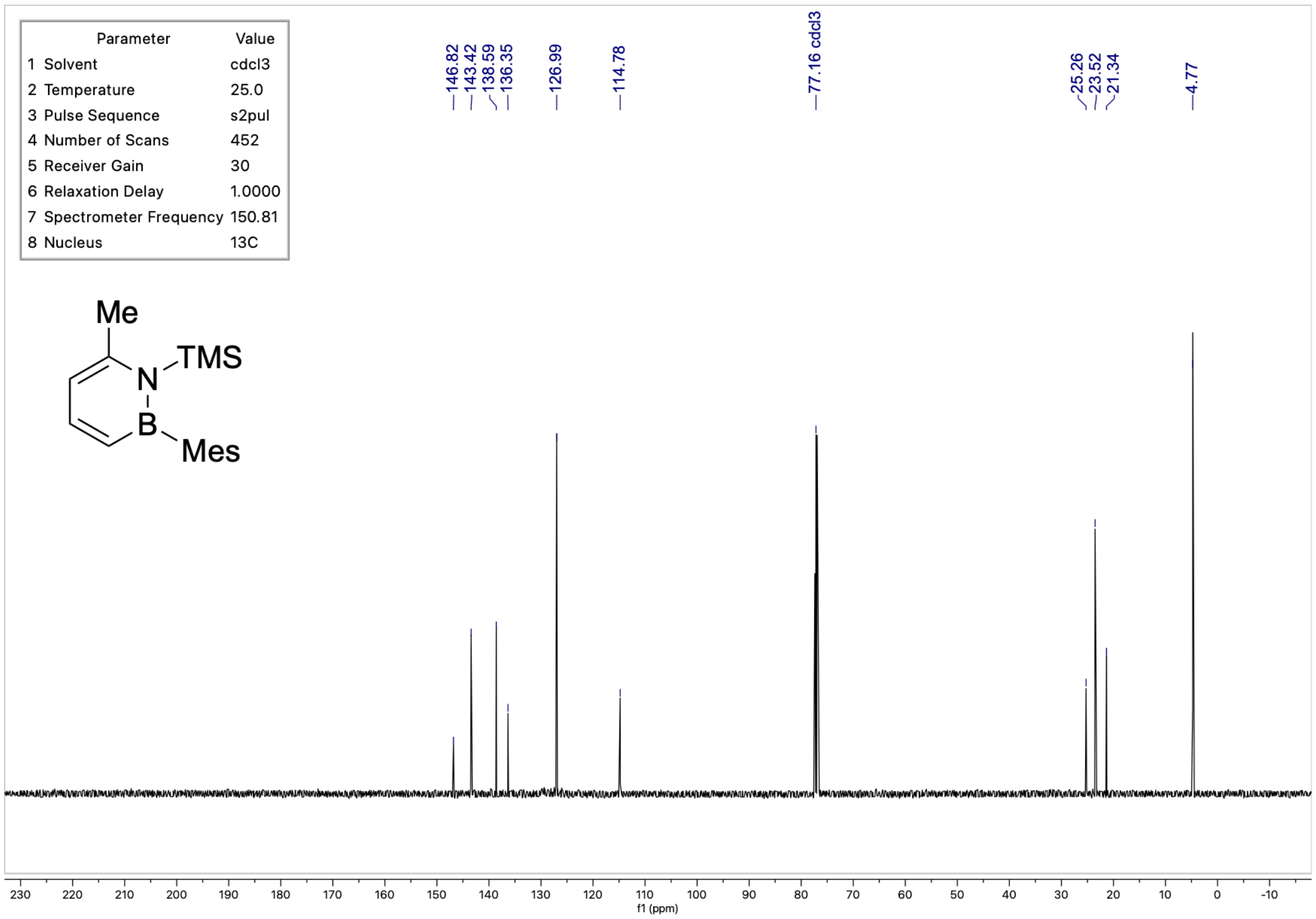
Parameter Value
1 Solvent cdcl3
2 Temperature 25.0
3 Pulse Sequence s2pul
4 Number of Scans 452
5 Receiver Gain 30
6 Relaxation Delay 1.0000
7 Spectrometer Frequency 150.81
8 Nucleus 13C
146.82
143.42
138.59
136.35
126.99
114.78
77.16 cdcl3
25.26
23.52
21.34
4.77
Me
N
TMS
B
Mes
f1 (ppm)

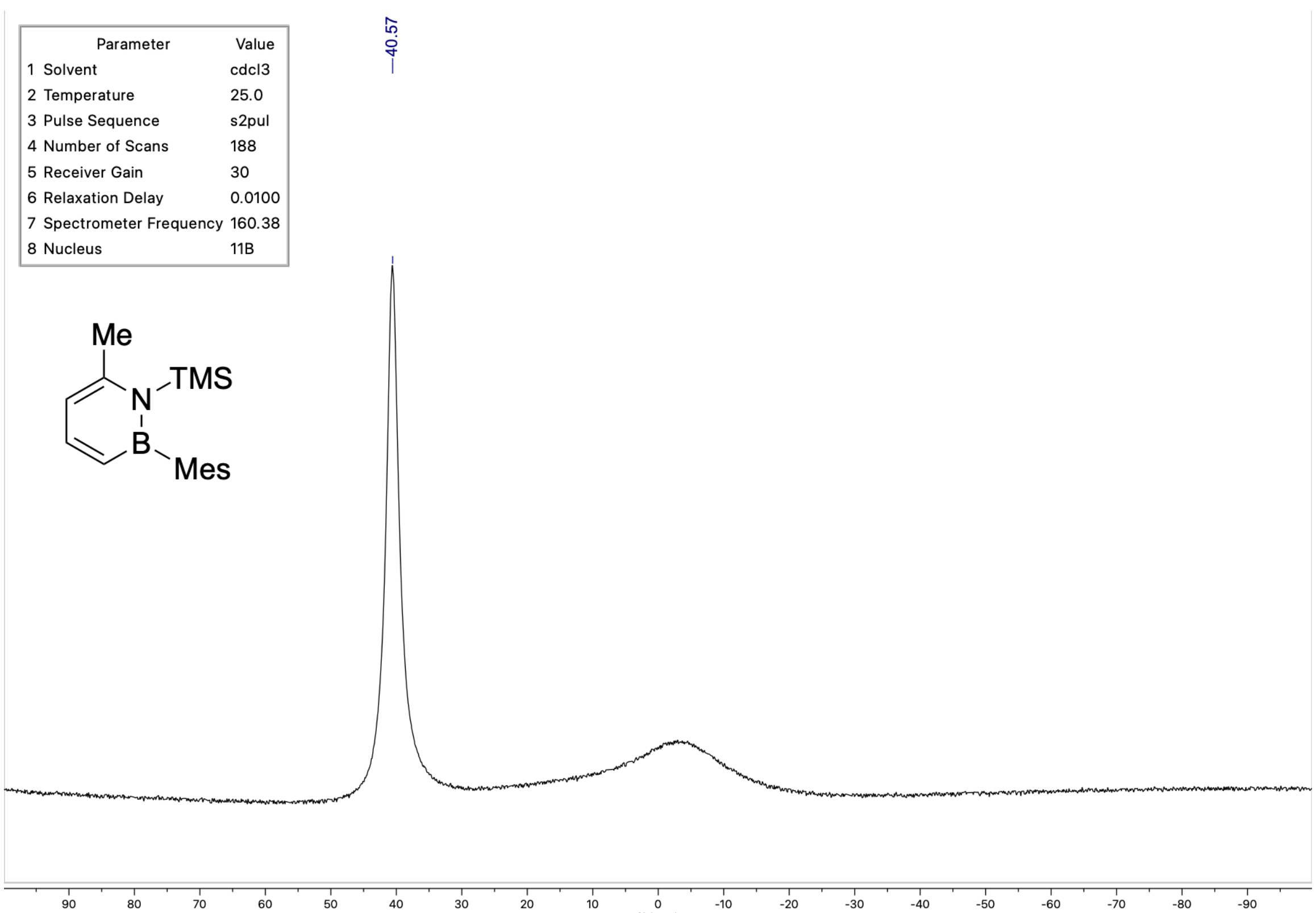
Parameter Value
1 Solvent cdcl3
2 Temperature 25.0
3 Pulse Sequence s2pul
4 Number of Scans 188
5 Receiver Gain 30
6 Relaxation Delay 0.0100
7 Spectrometer Frequency 160.38
8 Nucleus 11B
40.57
Me
N
TMS
B
Mes
f1 (ppm)

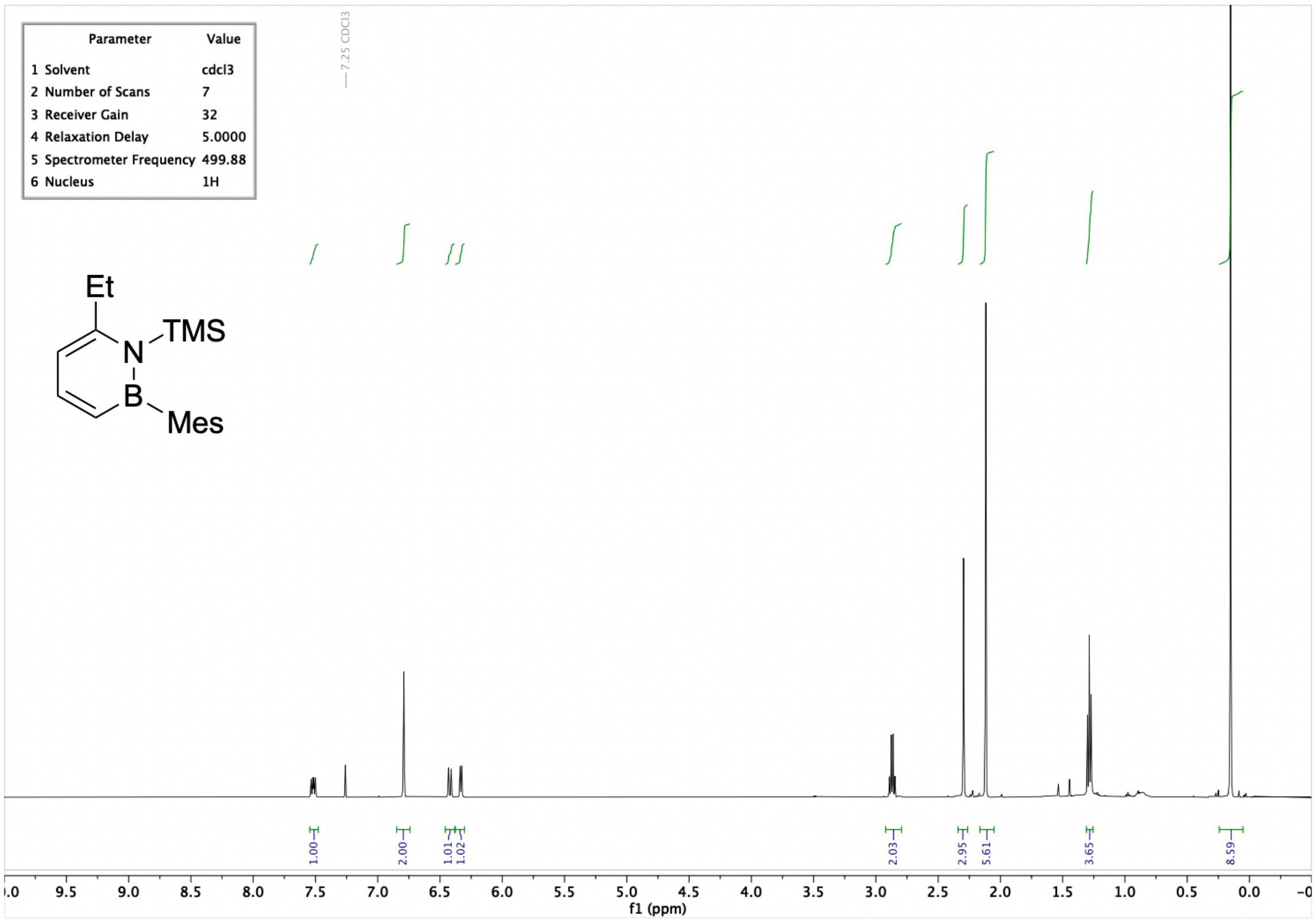

Parameter Value
1 Solvent cdcl3
2 Number of Scans 7
3 Receiver Gain 32
4 Relaxation Delay 5.0000
5 Spectrometer Frequency 499.88
6 Nucleus 1H
7.25 CDCl3
Et
N
TMS
B
Mes
1.00
2.00
1.01
1.02
2.03
2.95
5.61
3.65
8.59
f1 (ppm)

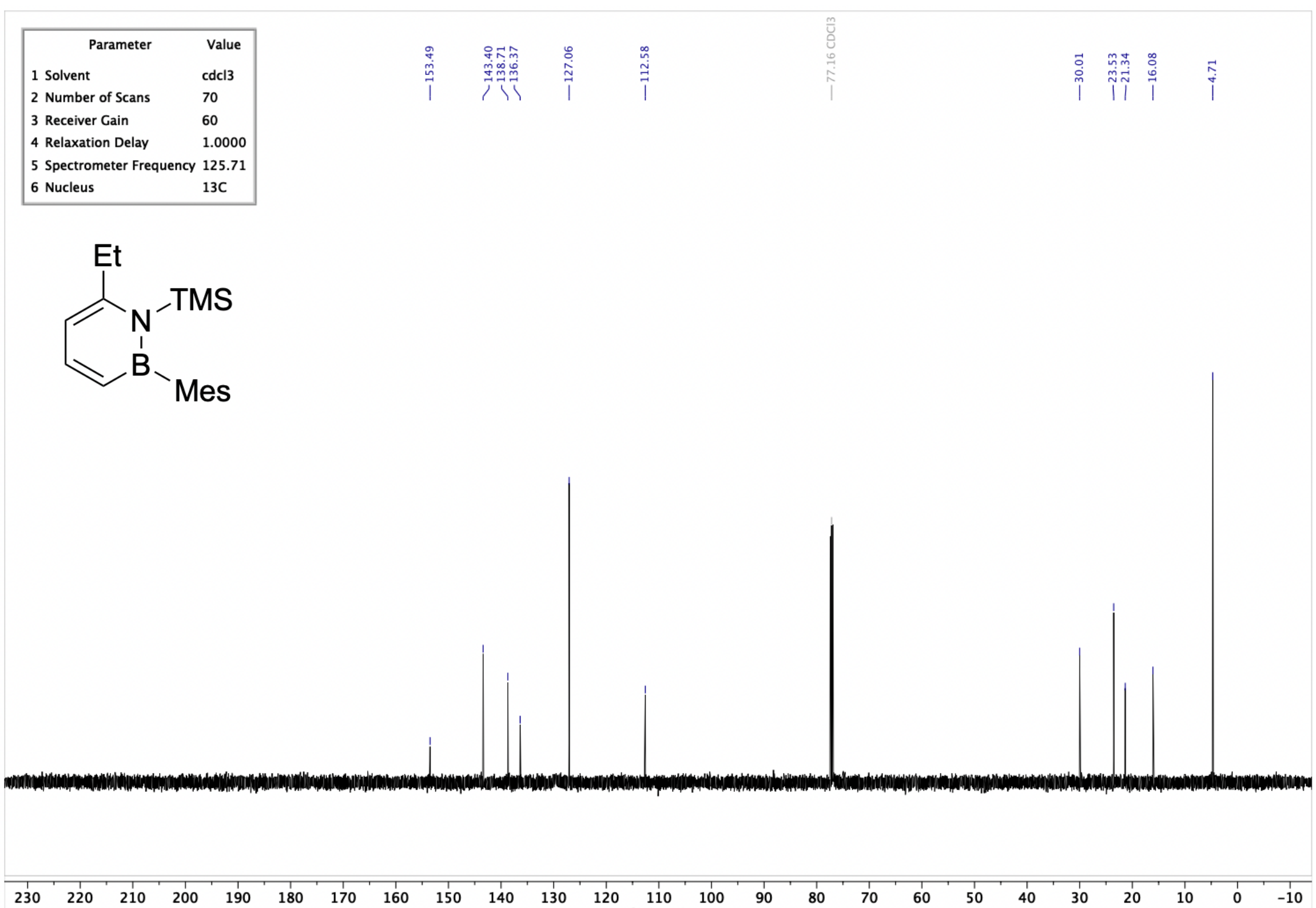

Parameter Value
1 Solvent cdcl3
2 Number of Scans 70
3 Receiver Gain 60
4 Relaxation Delay 1.0000
5 Spectrometer Frequency 125.71
6 Nucleus 13C
153.49
143.40
138.71
136.37
127.06
112.58
77.16 CDCl3
30.01
23.53
21.34
16.08
4.71
Et
N
TMS
B
Mes
f1 (ppm)

Parameter	Value
1 Solvent	cdcl3
2 Number of Scans	335
3 Receiver Gain	60
4 Relaxation Delay	0.0100
5 Spectrometer Frequency	160.38
6 Nucleus	11B

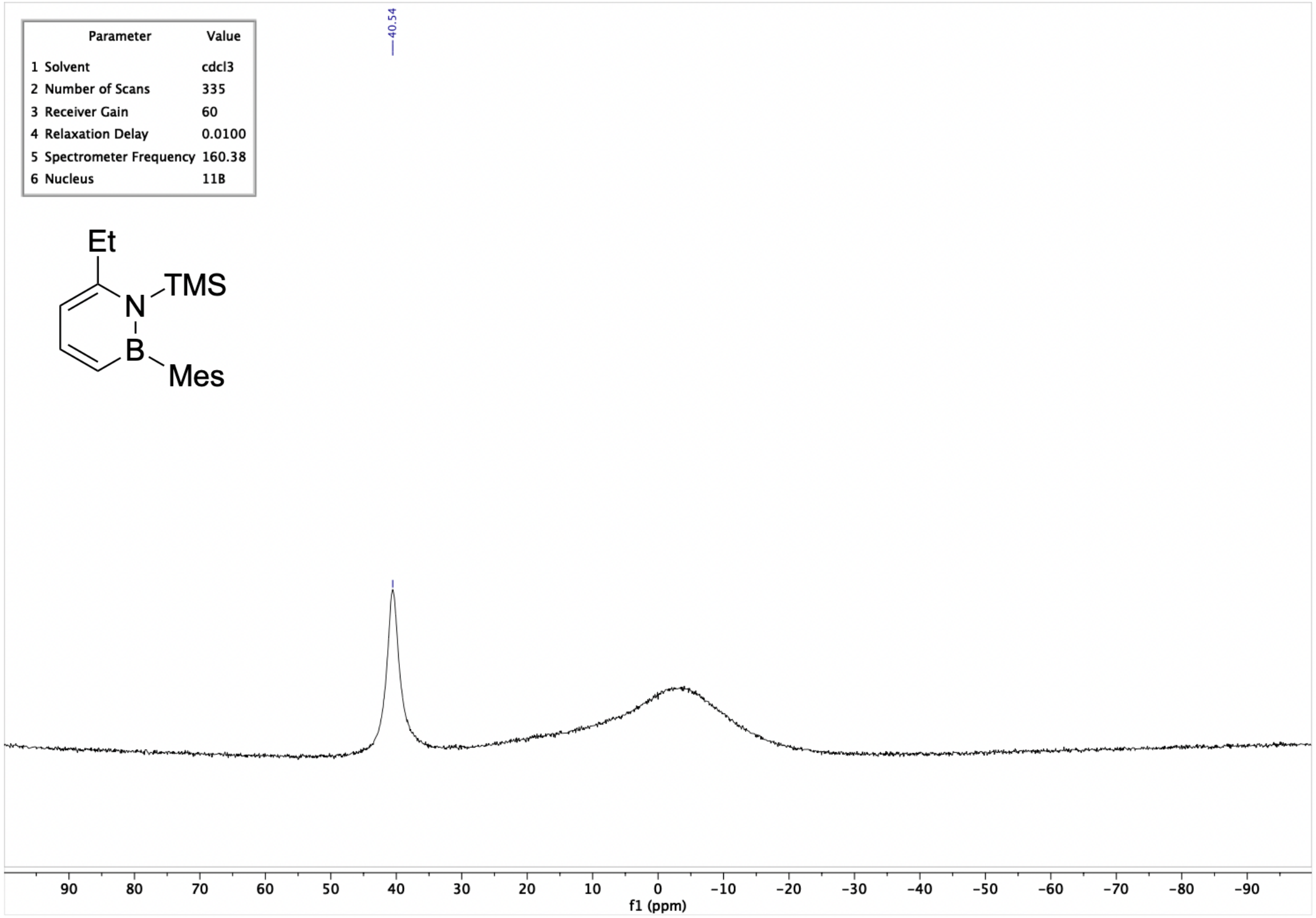

Parameter	Value
1 Solvent	cdcl3
2 Number of Scans	7
3 Receiver Gain	36
4 Relaxation Delay	5.0000
5 Spectrometer Frequency	499.88
6 Nucleus	1H

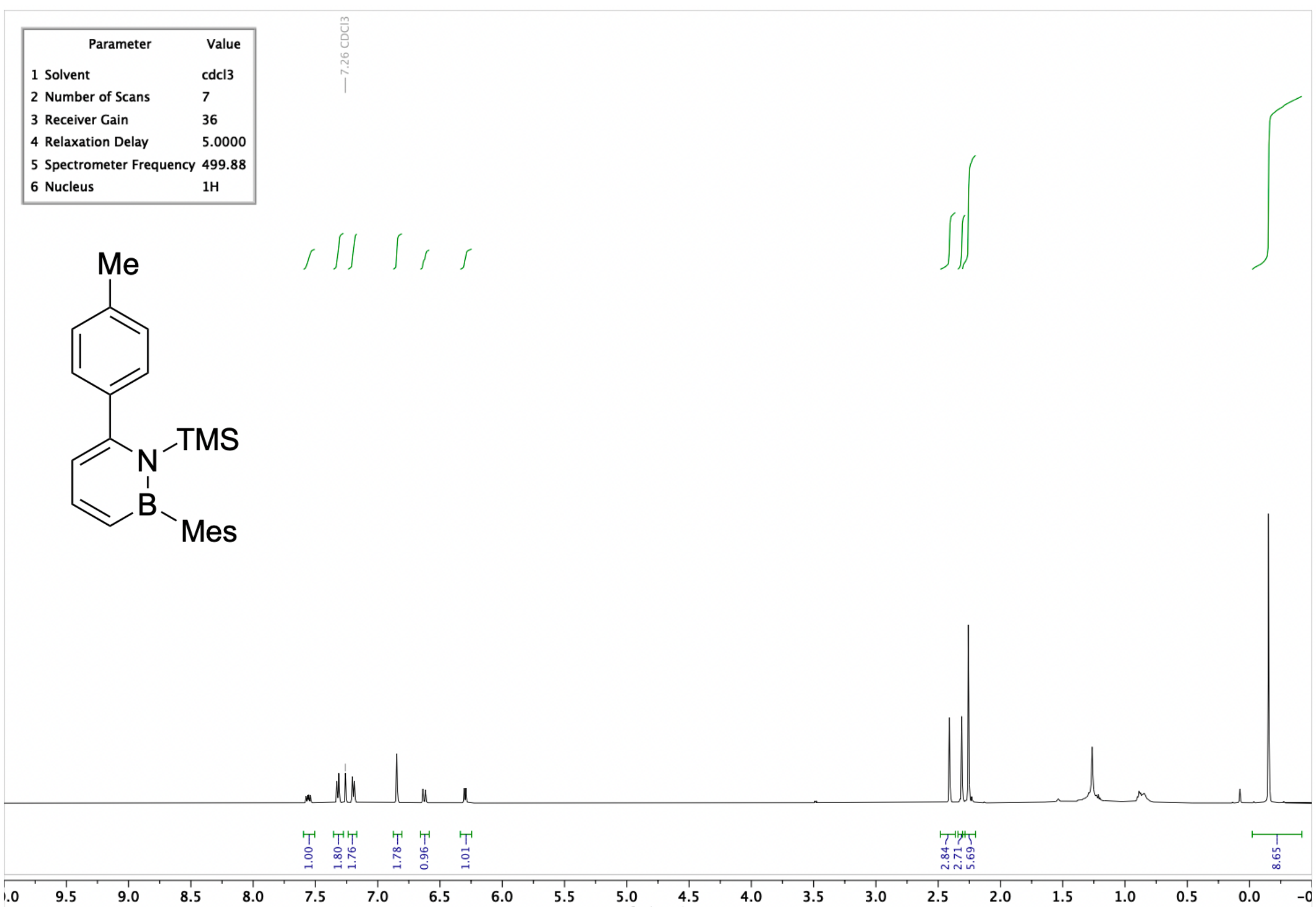

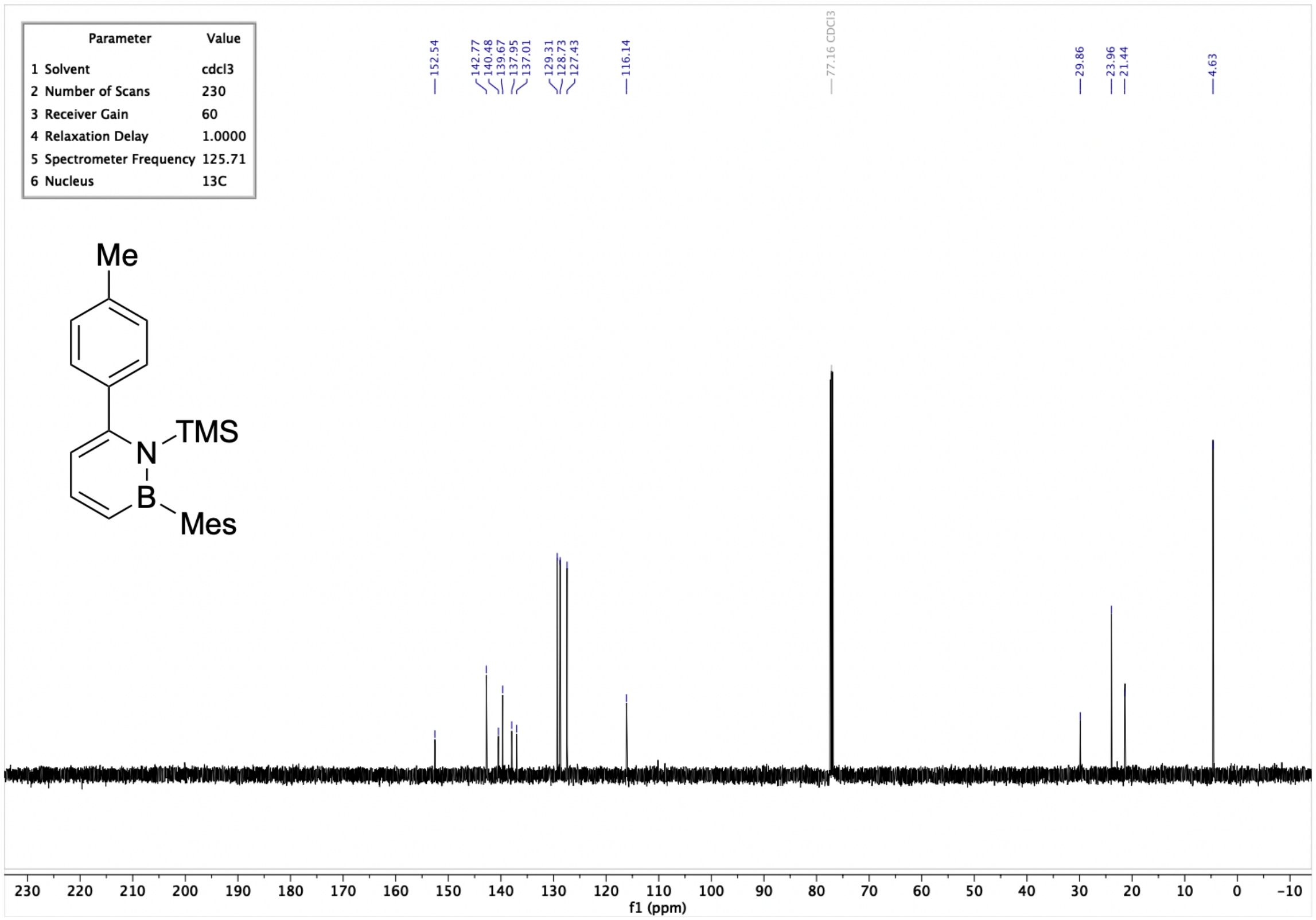

Parameter Value
1 Solvent cdcl3
2 Number of Scans 230
3 Receiver Gain 60
4 Relaxation Delay 1.0000
5 Spectrometer Frequency 125.71
6 Nucleus 13C
152.54
142.77
140.48
139.67
137.95
137.01
129.31
128.73
127.43
116.14
77.16 CDCl3
29.86
23.96
21.44
4.63
Me
TMS
N
B
Mes
f1 (ppm)

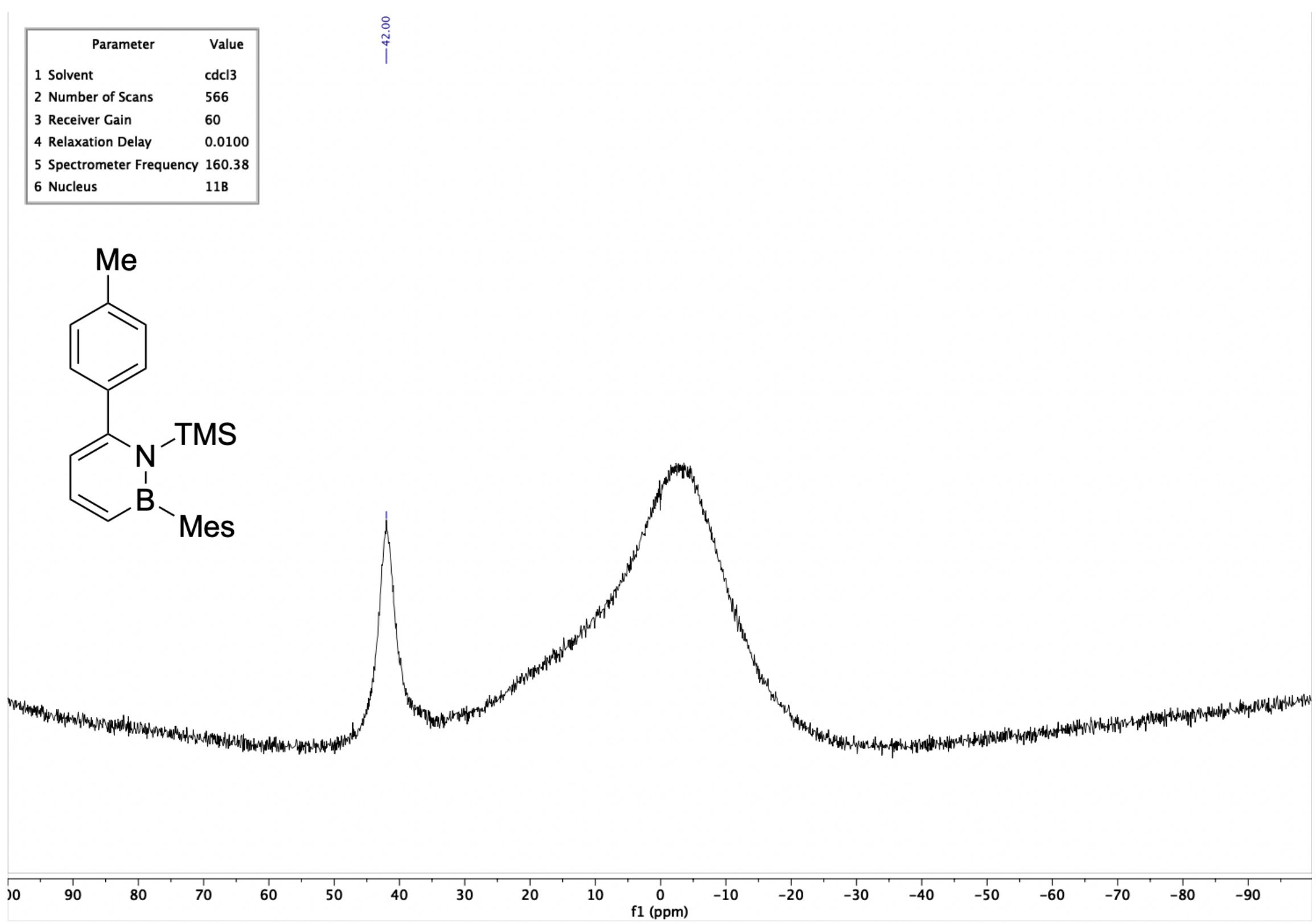

Parameter Value
1 Solvent cdcl3
2 Number of Scans 566
3 Receiver Gain 60
4 Relaxation Delay 0.0100
5 Spectrometer Frequency 160.38
6 Nucleus 11B
42.00
Me
TMS
N
B
Mes
f1 (ppm)

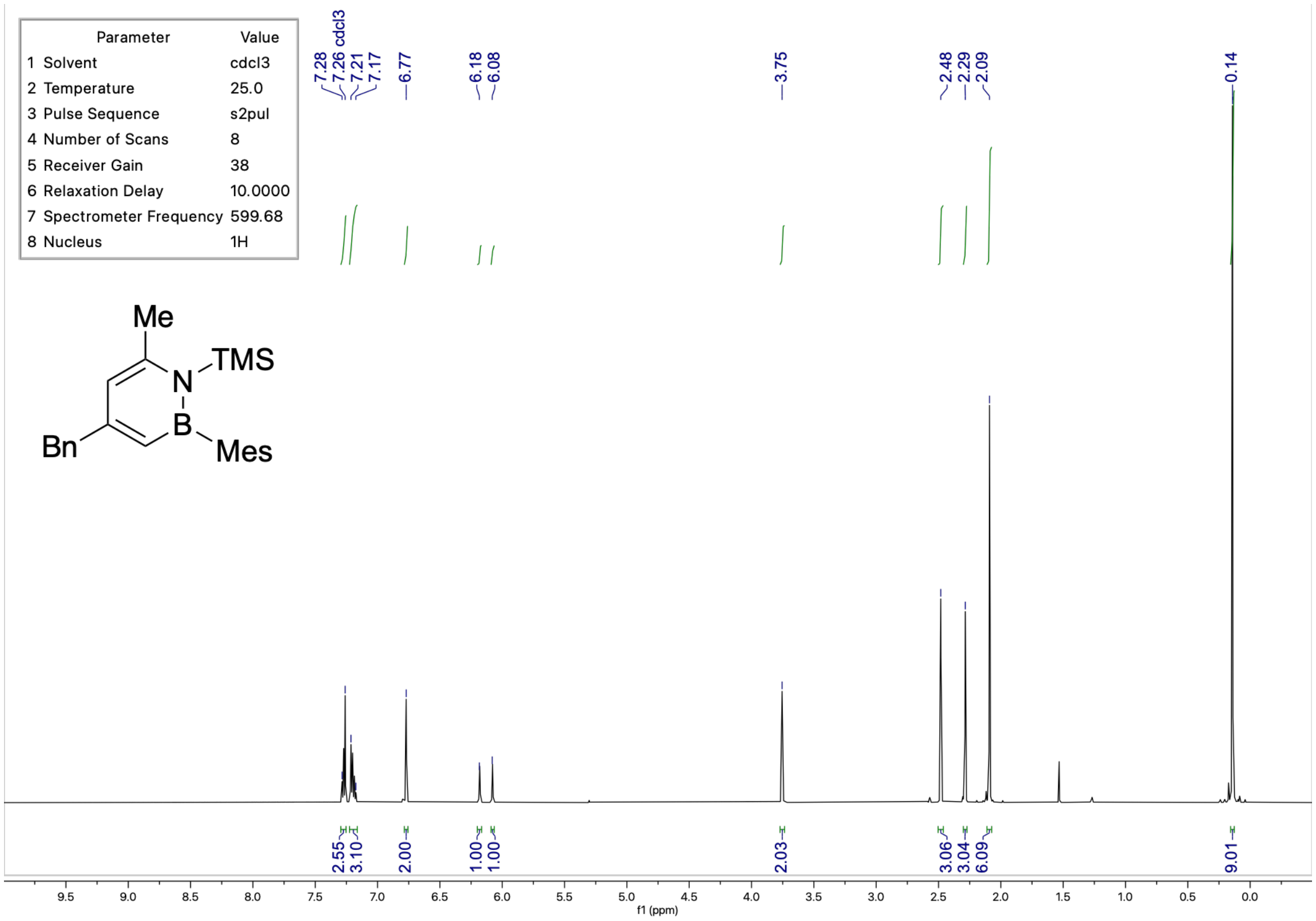

Parameter Value
1 Solvent cdcl3
2 Temperature 25.0
3 Pulse Sequence s2pul
4 Number of Scans 8
5 Receiver Gain 38
6 Relaxation Delay 10.0000
7 Spectrometer Frequency 599.68
8 Nucleus 1H
Me
N
TMS
B
Bn
Mes
7.28
7.26 cdcl3
7.21
7.17
6.77
6.18
6.08
3.75
2.48
2.29
2.09
0.14
2.55
3.10
2.00
1.00
1.00
2.03
3.06
3.04
6.09
9.01
f1 (ppm)

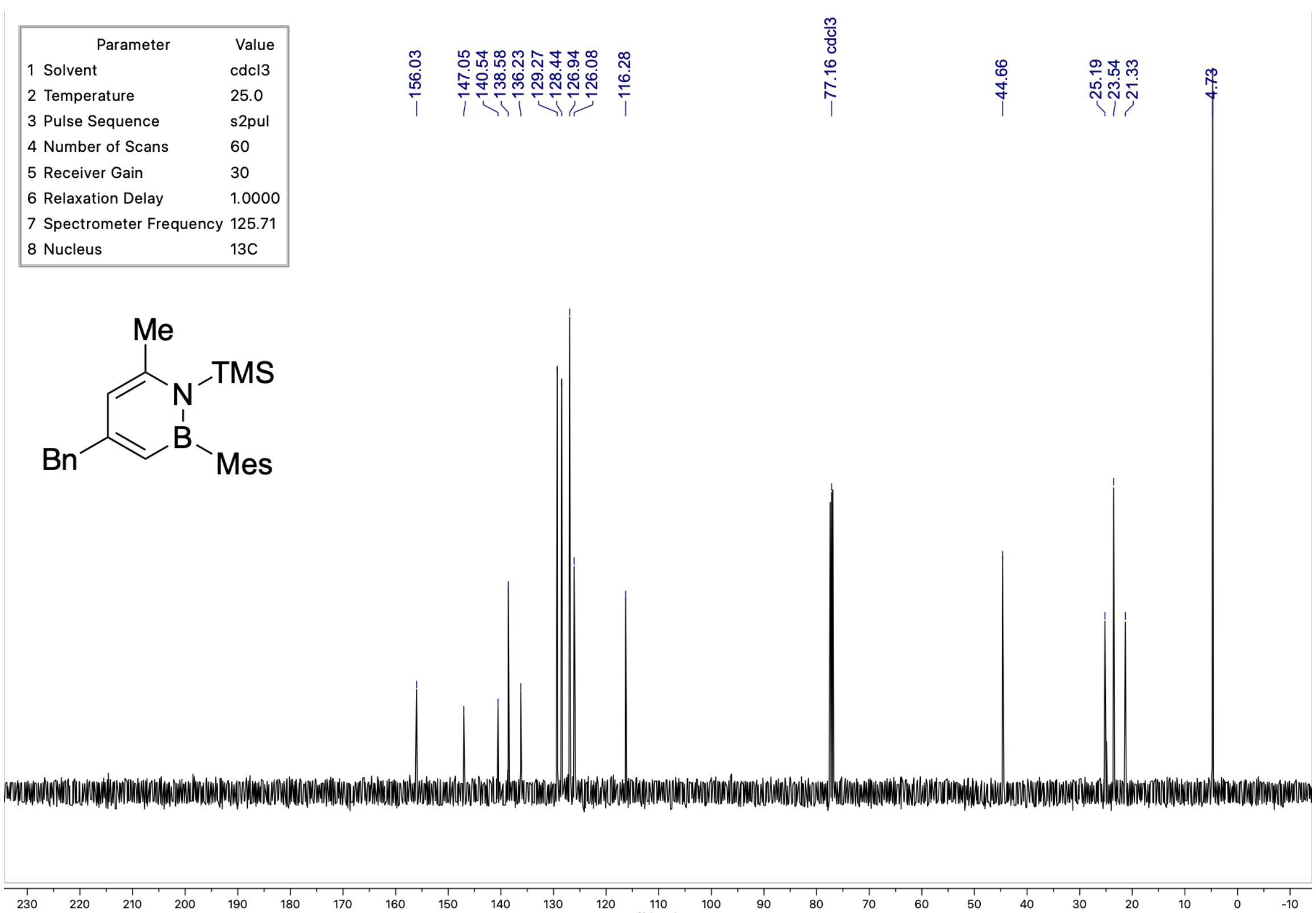

Parameter Value
1 Solvent cdcl3
2 Temperature 25.0
3 Pulse Sequence s2pul
4 Number of Scans 60
5 Receiver Gain 30
6 Relaxation Delay 1.0000
7 Spectrometer Frequency 125.71
8 Nucleus 13C
Me
N
TMS
B
Bn
Mes
156.03
147.05
140.54
138.58
136.23
129.27
128.44
126.94
126.08
116.28
77.16 cdcl3
44.66
25.19
23.54
21.33
4.73
f1 (ppm)

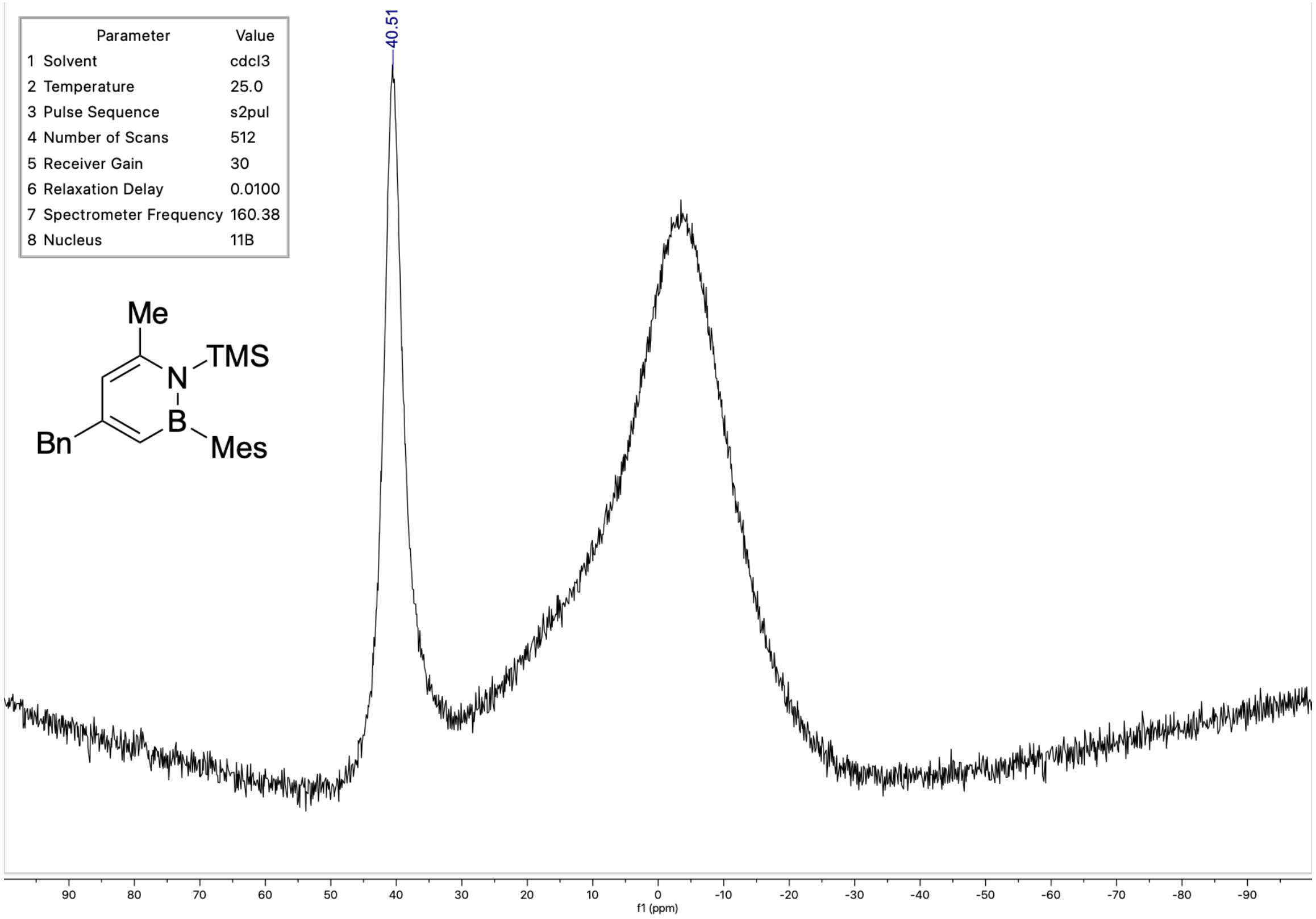

Parameter Value
1 Solvent cdcl3
2 Temperature 25.0
3 Pulse Sequence s2pul
4 Number of Scans 512
5 Receiver Gain 30
6 Relaxation Delay 0.0100
7 Spectrometer Frequency 160.38
8 Nucleus 11B
40.51
Me
TMS
N
B
Bn
Mes
f1 (ppm)

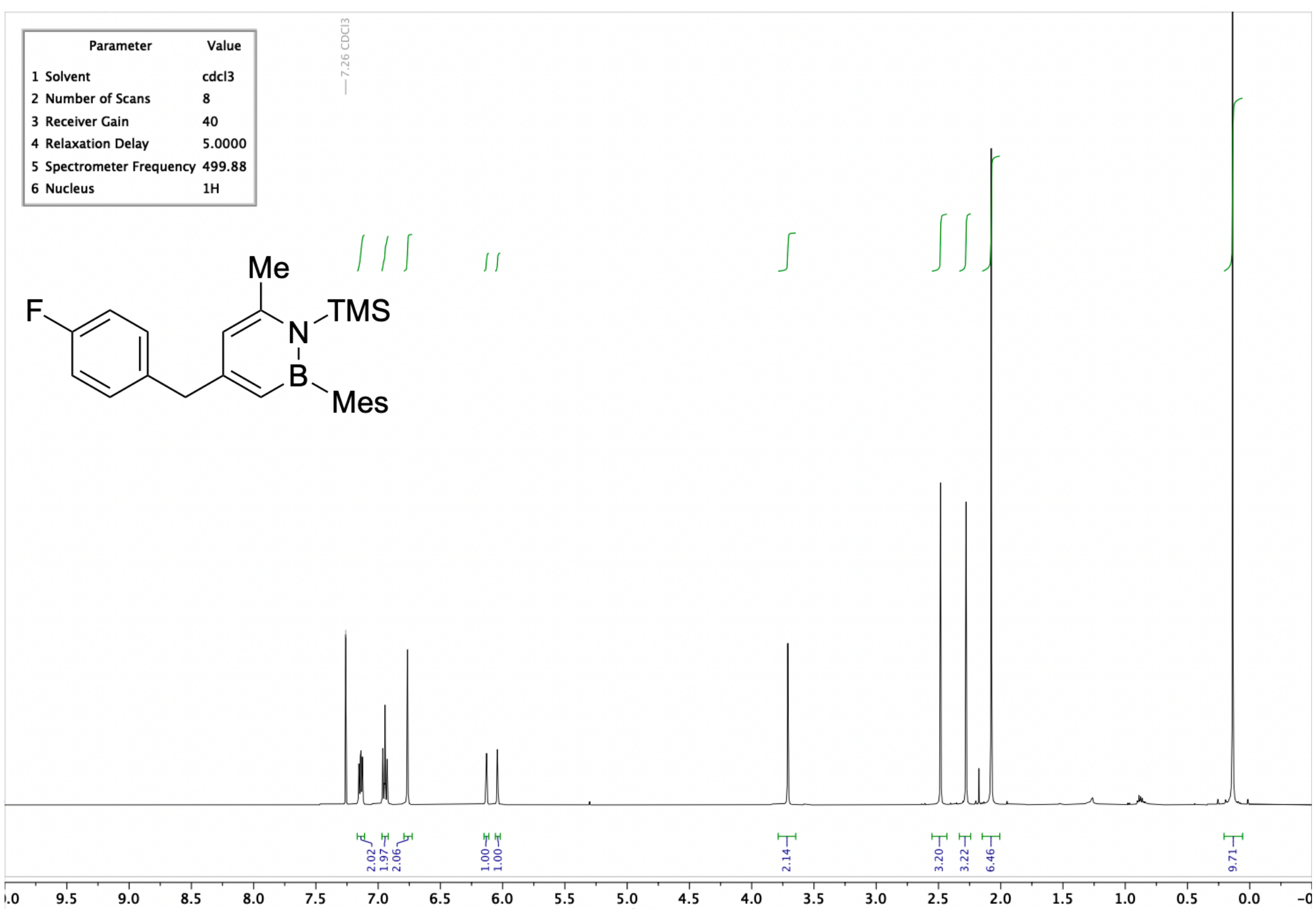

Parameter Value
1 Solvent cdcl3
2 Number of Scans 8
3 Receiver Gain 40
4 Relaxation Delay 5.0000
5 Spectrometer Frequency 499.88
6 Nucleus 1H
7.26 CDCl3
Me
F
TMS
N
B
Mes
2.02
1.97
2.06
1.00
1.00
2.14
3.20
3.22
6.46
9.71
f1 (ppm)

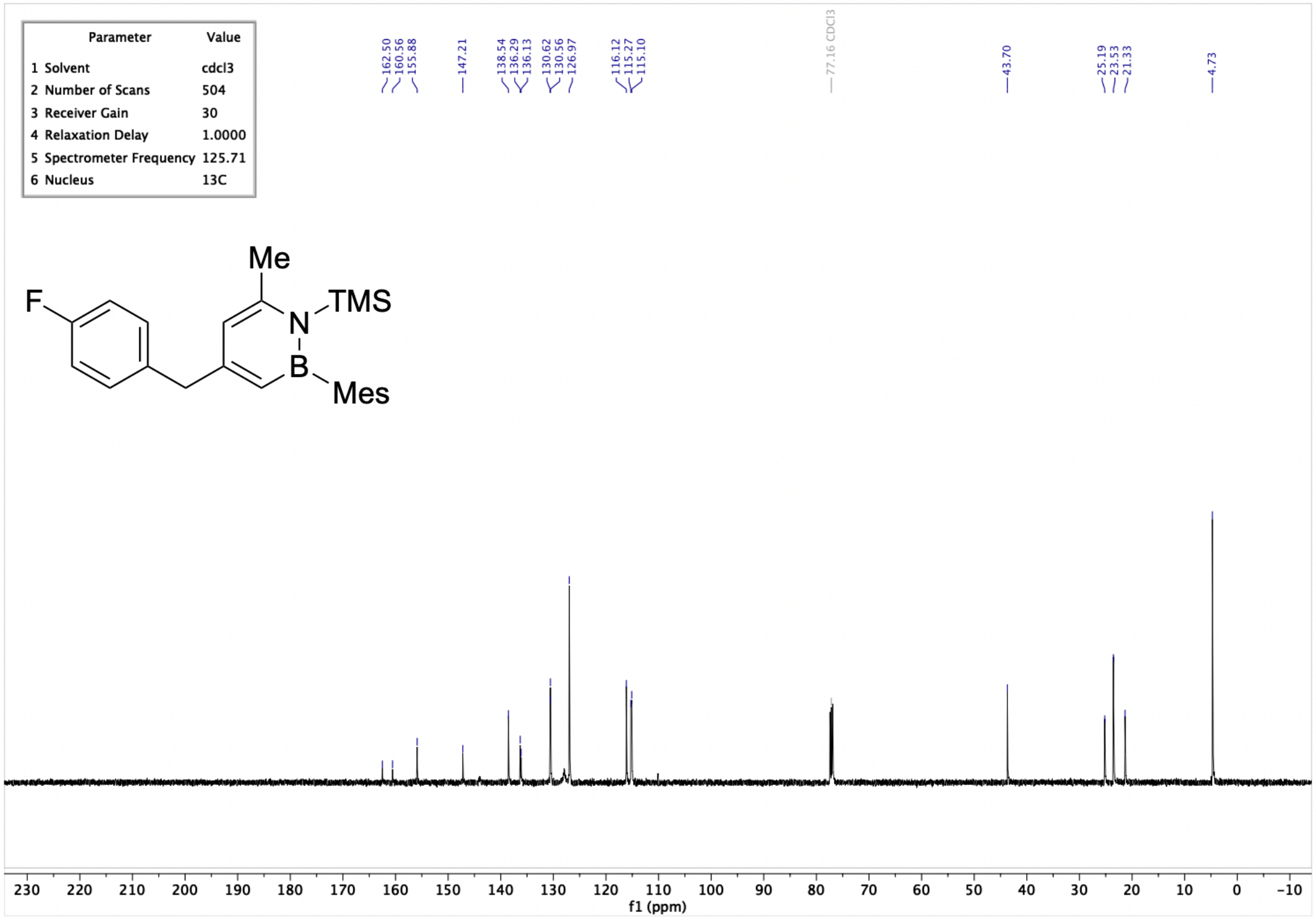

Parameter Value
1 Solvent cdcl3
2 Number of Scans 504
3 Receiver Gain 30
4 Relaxation Delay 1.0000
5 Spectrometer Frequency 125.71
6 Nucleus 13C
162.50
160.56
155.88
147.21
138.54
136.29
136.13
130.62
130.56
126.97
116.12
115.27
115.10
77.16 CDCl3
43.70
25.19
23.53
21.33
4.73
Me
F
N
TMS
B
Mes
f1 (ppm)

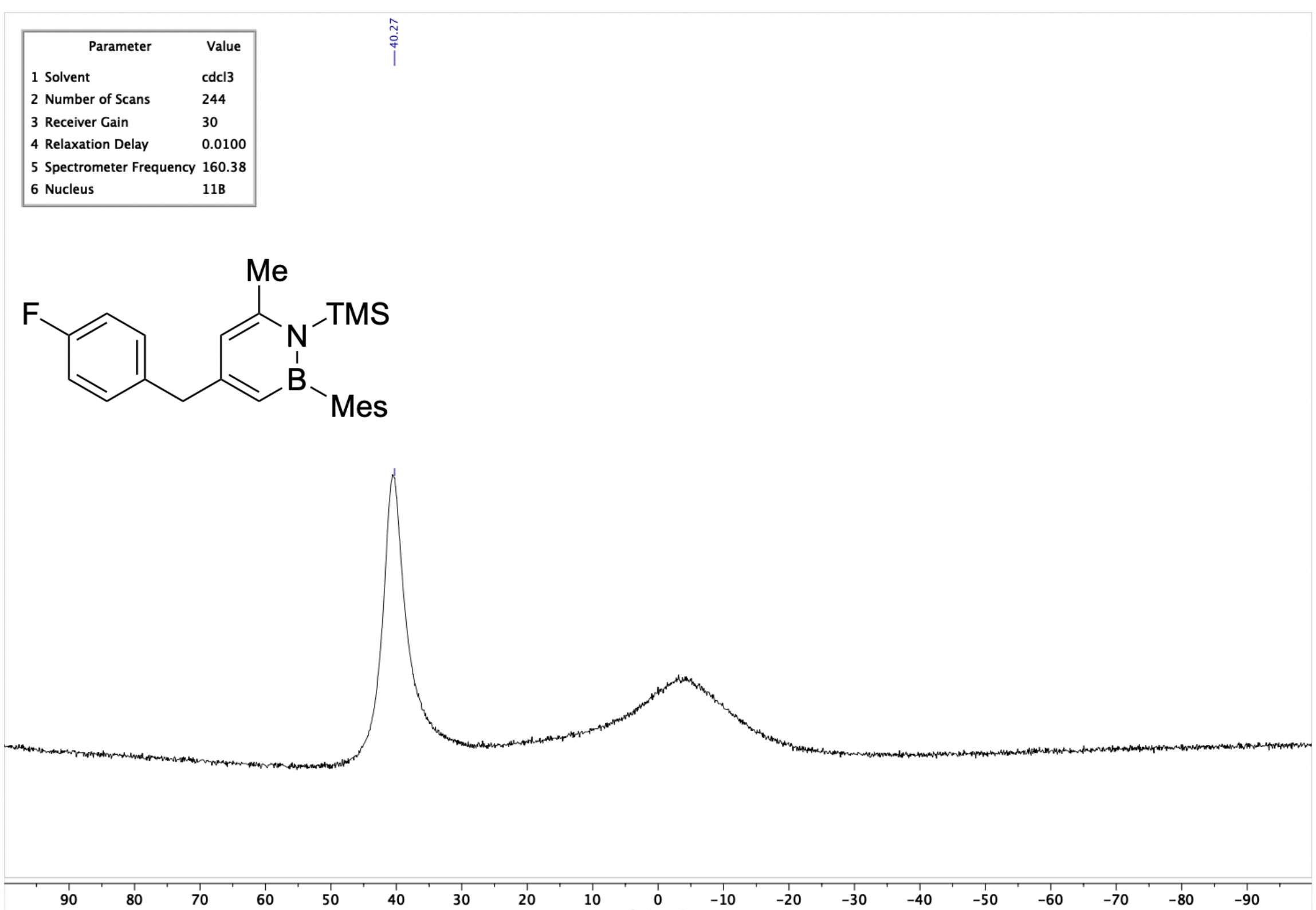

Parameter Value
1 Solvent cdcl3
2 Number of Scans 244
3 Receiver Gain 30
4 Relaxation Delay 0.0100
5 Spectrometer Frequency 160.38
6 Nucleus 11B
40.27
Me
F
N
TMS
B
Mes
f1 (ppm)

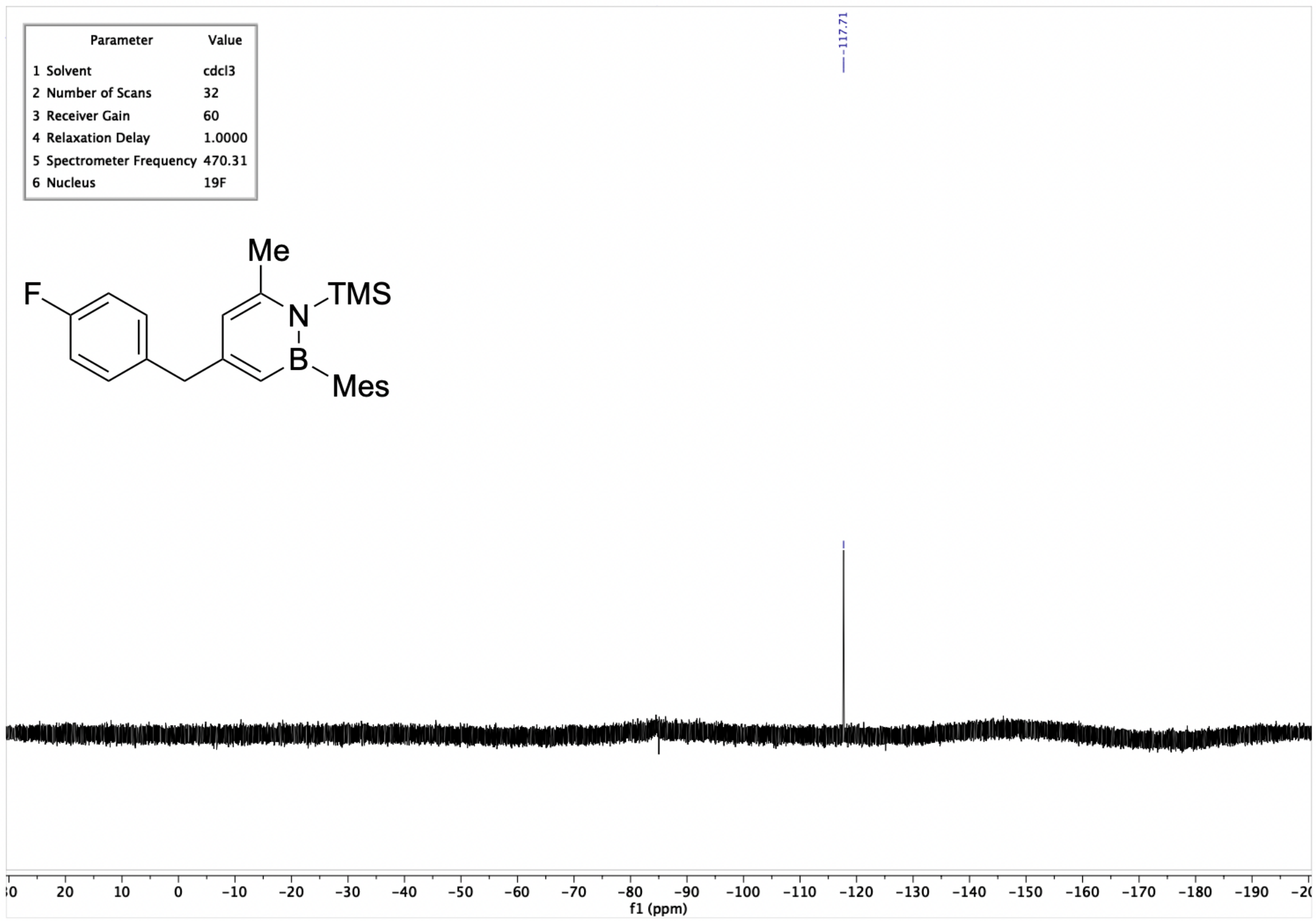
Parameter Value
1 Solvent cdcl3
2 Number of Scans 32
3 Receiver Gain 60
4 Relaxation Delay 1.0000
5 Spectrometer Frequency 470.31
6 Nucleus 19F
-117.71
F
Me
N
TMS
B
Mes
f1 (ppm)

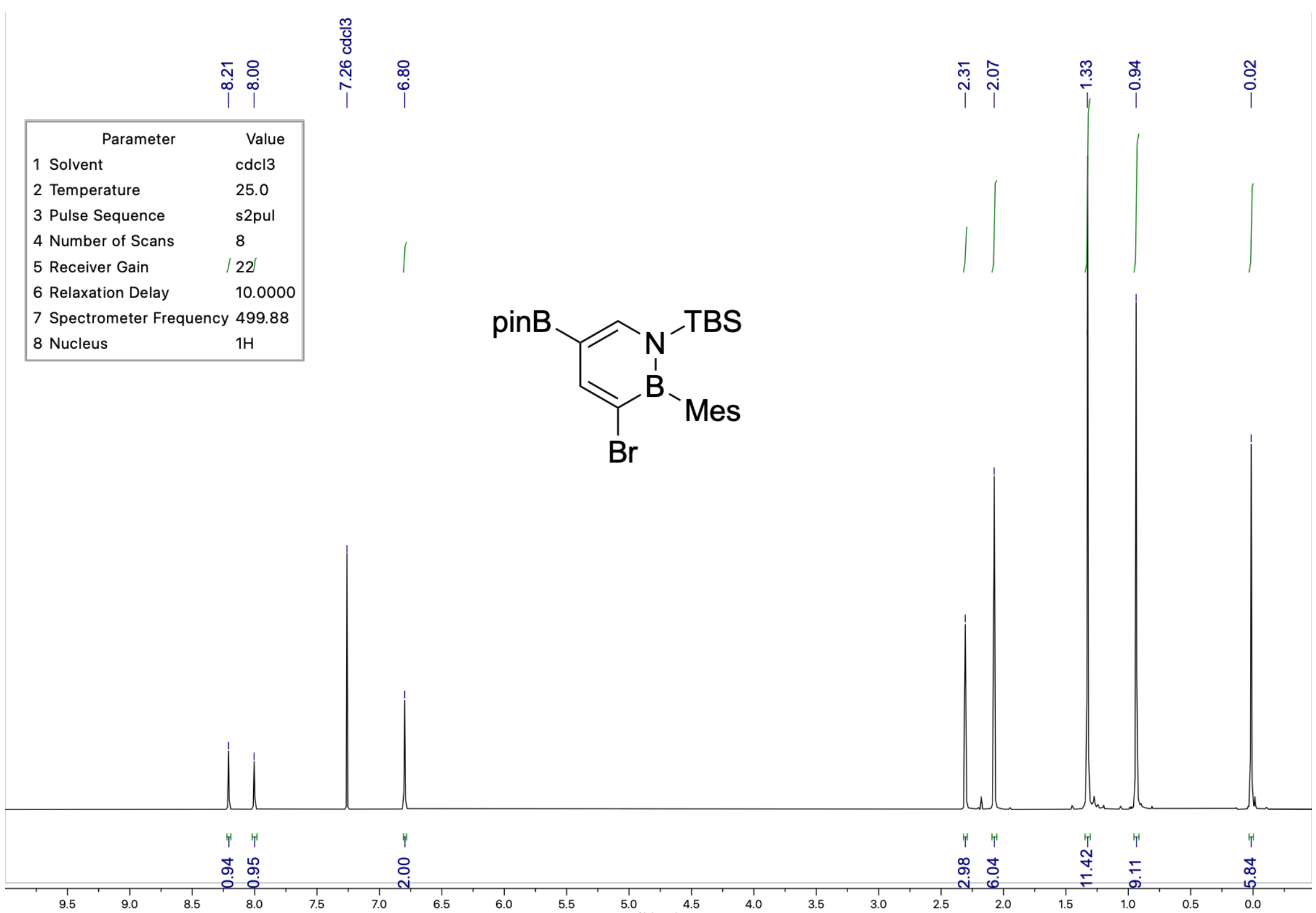
8.21
8.00
7.26 cdcl3
6.80
2.31
2.07
1.33
0.94
0.02
Parameter Value
1 Solvent cdcl3
2 Temperature 25.0
3 Pulse Sequence s2pul
4 Number of Scans 8
5 Receiver Gain 22
6 Relaxation Delay 10.0000
7 Spectrometer Frequency 499.88
8 Nucleus 1H
pinB
N
TBS
B
Mes
Br
0.94
0.95
2.00
2.98
6.04
11.42
9.11
5.84
f1 (ppm)

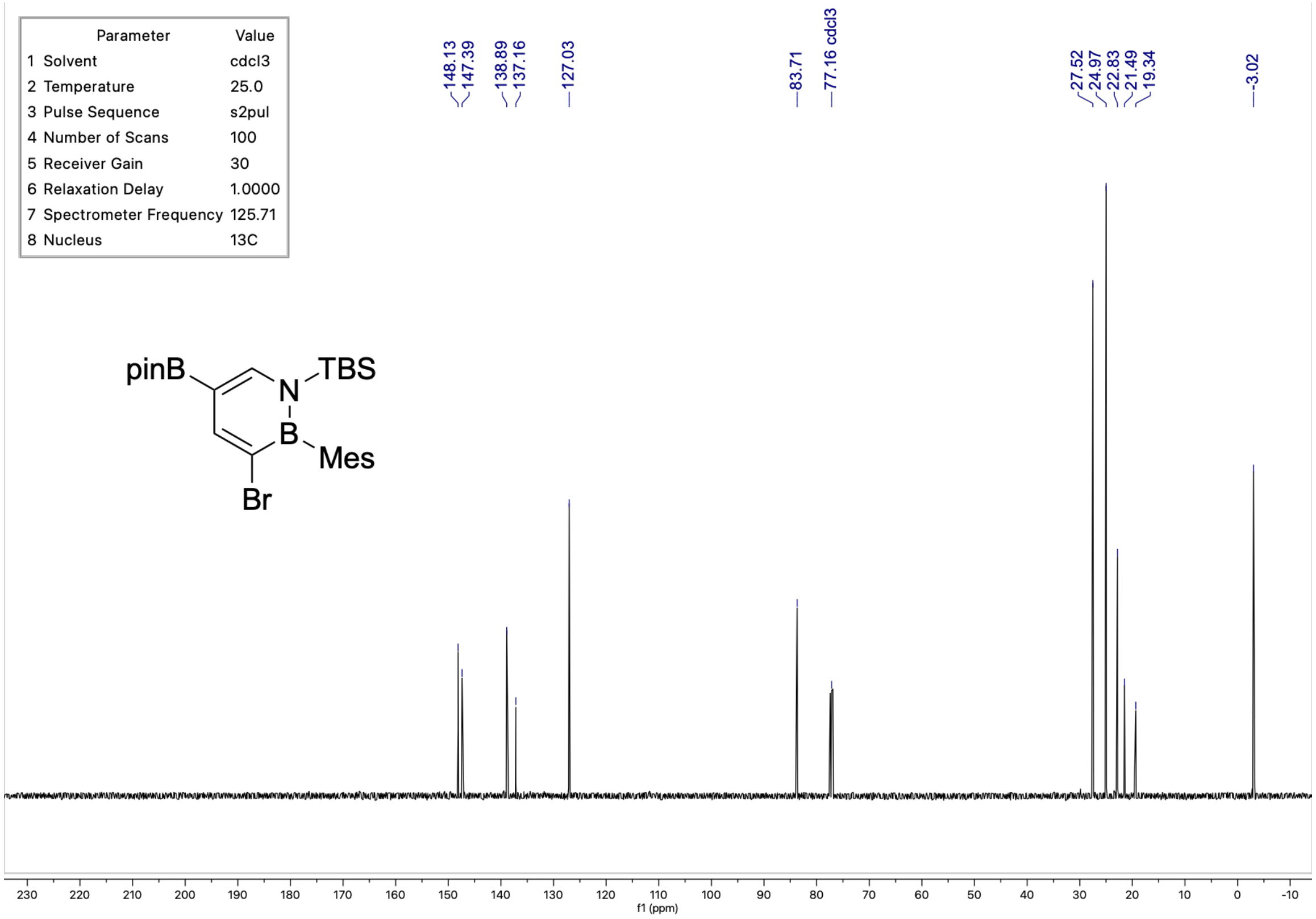
Parameter Value
1 Solvent cdcl3
2 Temperature 25.0
3 Pulse Sequence s2pul
4 Number of Scans 100
5 Receiver Gain 30
6 Relaxation Delay 1.0000
7 Spectrometer Frequency 125.71
8 Nucleus 13C
148.13
147.39
138.89
137.16
127.03
83.71
77.16 cdcl3
27.52
24.97
22.83
21.49
19.34
-3.02
pinB
N
TBS
B
Mes
Br
f1 (ppm)

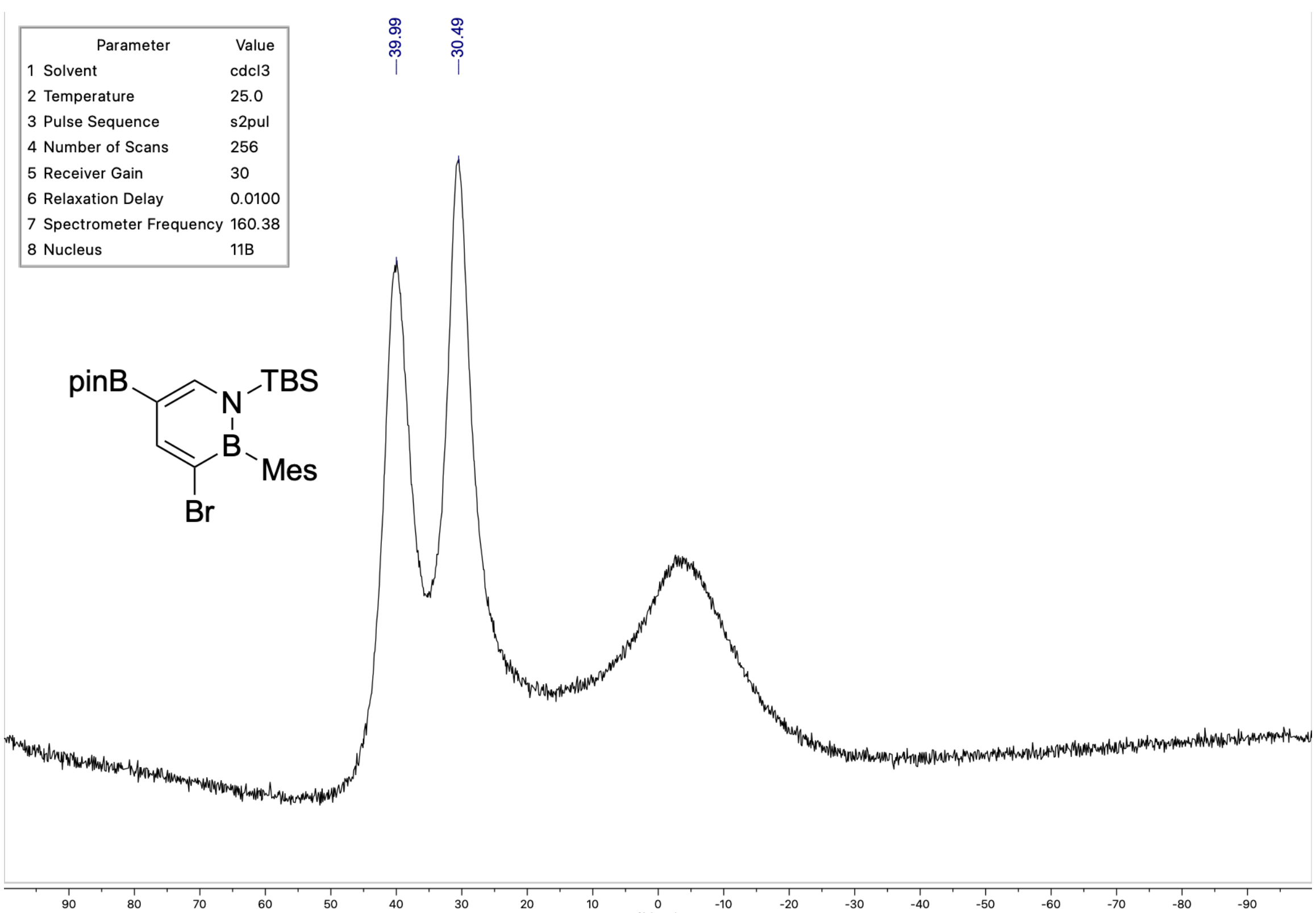
Parameter Value
1 Solvent cdcl3
2 Temperature 25.0
3 Pulse Sequence s2pul
4 Number of Scans 256
5 Receiver Gain 30
6 Relaxation Delay 0.0100
7 Spectrometer Frequency 160.38
8 Nucleus 11B
39.99
30.49
pinB
N
TBS
B
Mes
Br
f1 (ppm)

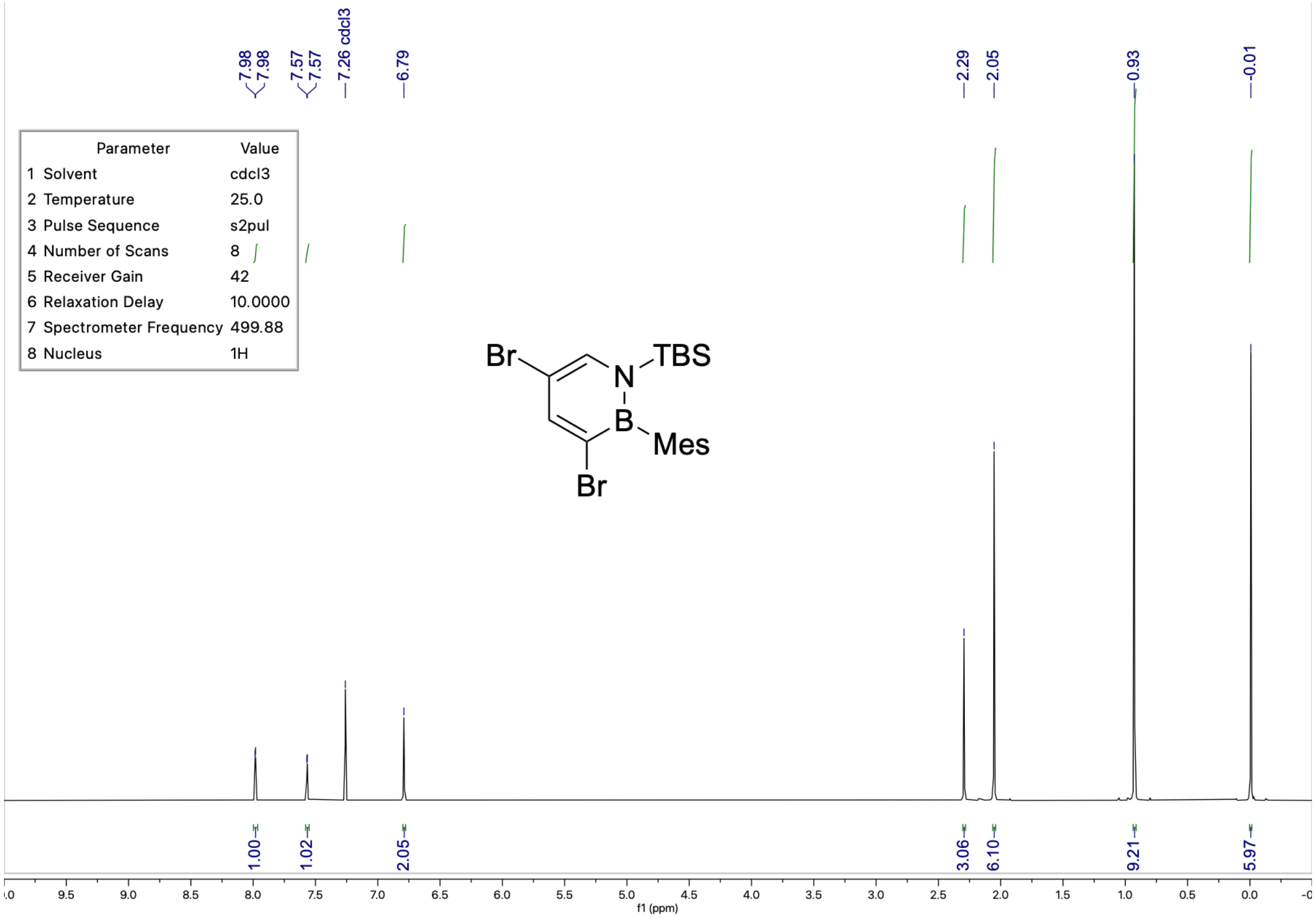
7.98
7.98
7.57
7.57
7.26 cdcl3
6.79
2.29
2.05
0.93
-0.01
Parameter Value
1 Solvent cdcl3
2 Temperature 25.0
3 Pulse Sequence s2pul
4 Number of Scans 8
5 Receiver Gain 42
6 Relaxation Delay 10.0000
7 Spectrometer Frequency 499.88
8 Nucleus 1H
Br
N
TBS
B
Mes
Br
1.00
1.02
2.05
3.06
6.10
9.21
5.97
f1 (ppm)

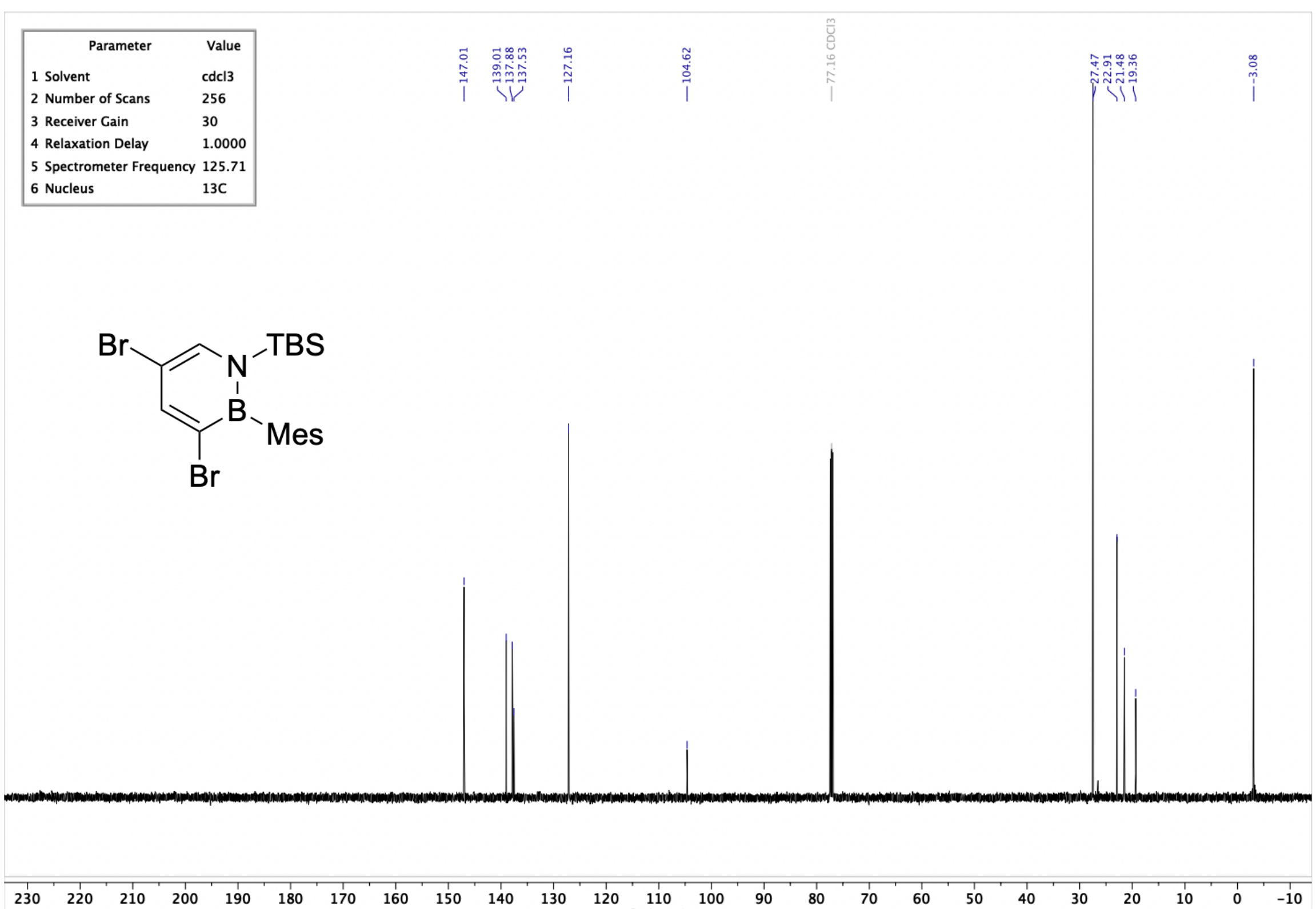
Parameter Value
1 Solvent cdcl3
2 Number of Scans 256
3 Receiver Gain 30
4 Relaxation Delay 1.0000
5 Spectrometer Frequency 125.71
6 Nucleus 13C
147.01
139.01
137.88
137.53
127.16
104.62
77.16 CDCl3
27.47
22.91
21.48
19.36
-3.08
Br
N
TBS
B
Mes
Br
f1 (ppm)

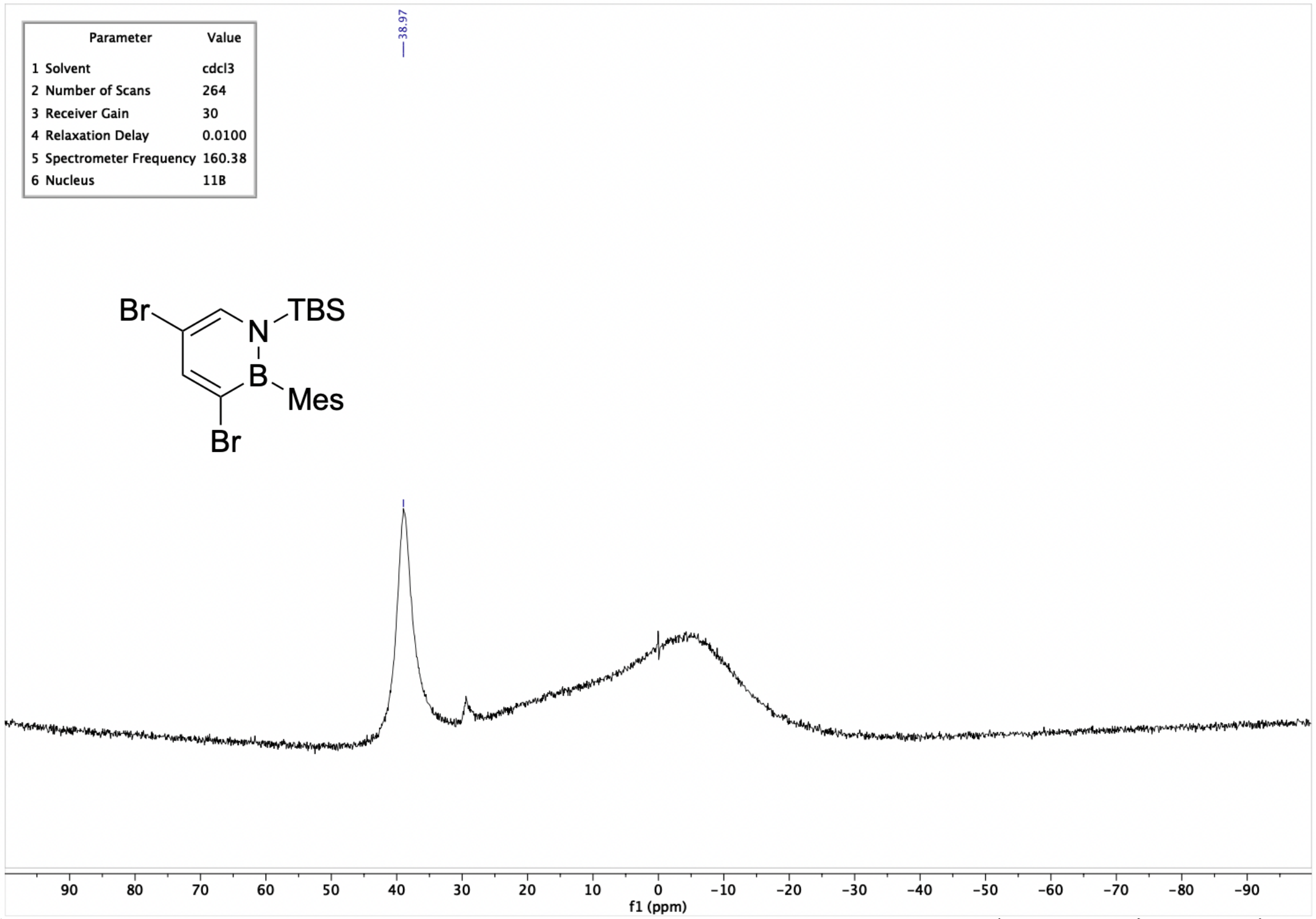
Parameter Value
1 Solvent cdcl3
2 Number of Scans 264
3 Receiver Gain 30
4 Relaxation Delay 0.0100
5 Spectrometer Frequency 160.38
6 Nucleus 11B
38.97
Br
N
TBS
B
Mes
Br
f1 (ppm)

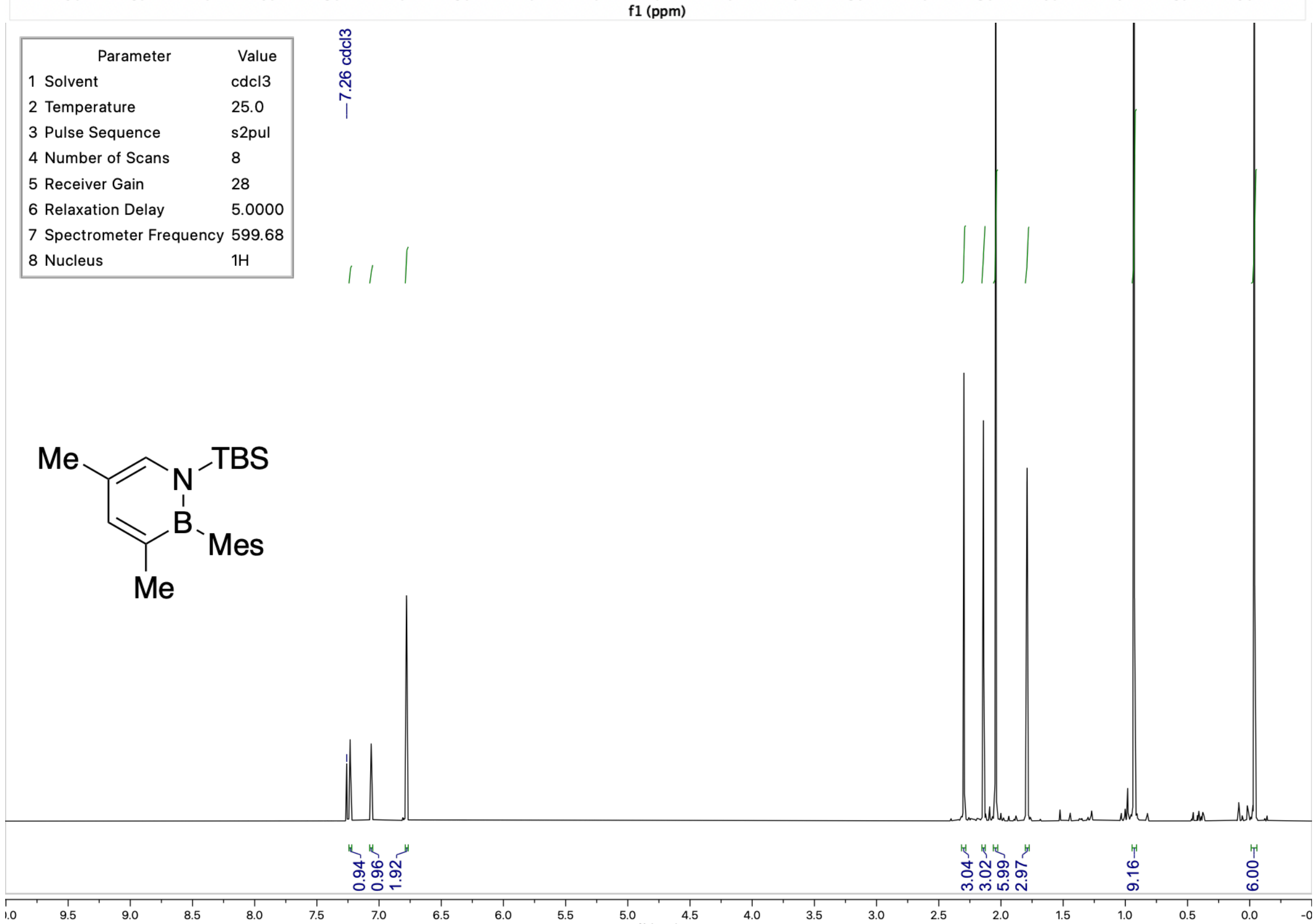
Parameter Value
1 Solvent cdcl3
2 Temperature 25.0
3 Pulse Sequence s2pul
4 Number of Scans 8
5 Receiver Gain 28
6 Relaxation Delay 5.0000
7 Spectrometer Frequency 599.68
8 Nucleus 1H
7.26 cdcl3
Me
N
TBS
B
Mes
Me
0.94
0.96
1.92
3.04
3.02
5.99
2.97
9.16
6.00
f1 (ppm)

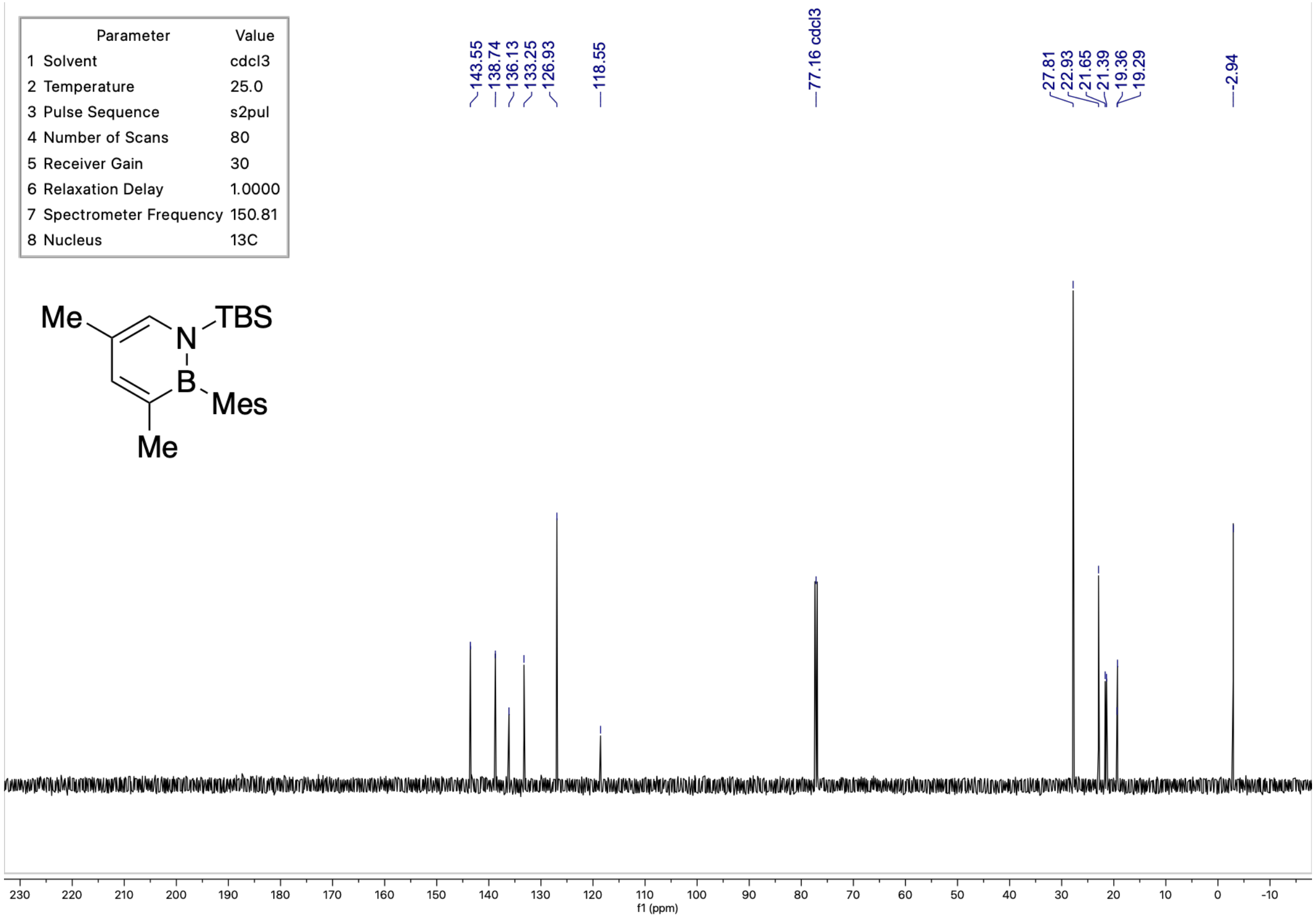
Parameter Value
1 Solvent cdcl3
2 Temperature 25.0
3 Pulse Sequence s2pul
4 Number of Scans 80
5 Receiver Gain 30
6 Relaxation Delay 1.0000
7 Spectrometer Frequency 150.81
8 Nucleus 13C
143.55
138.74
136.13
133.25
126.93
118.55
77.16 cdcl3
27.81
22.93
21.65
21.39
19.36
19.29
-2.94
Me
N
TBS
B
Mes
Me
f1 (ppm)

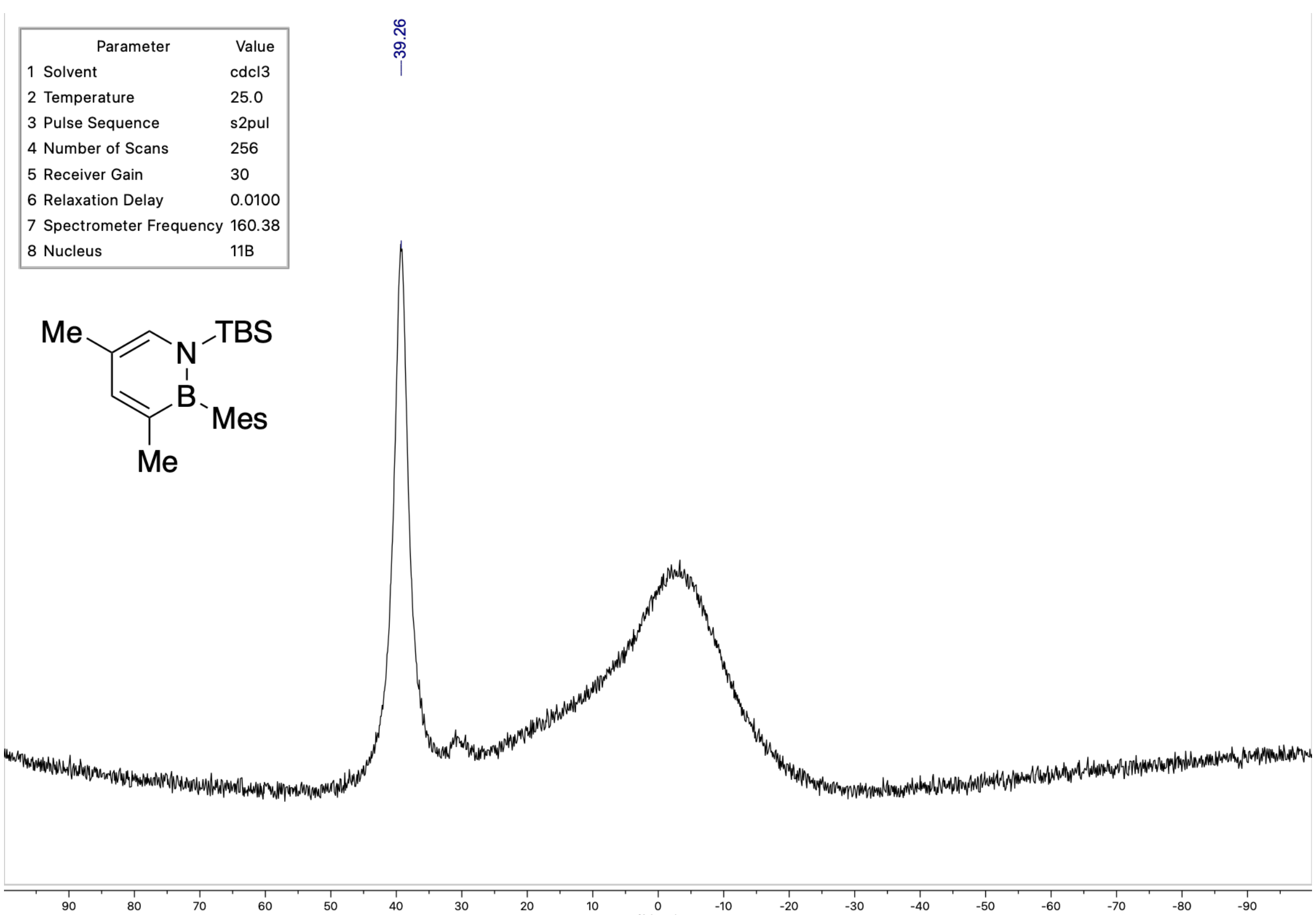
Parameter Value
1 Solvent cdcl3
2 Temperature 25.0
3 Pulse Sequence s2pul
4 Number of Scans 256
5 Receiver Gain 30
6 Relaxation Delay 0.0100
7 Spectrometer Frequency 160.38
8 Nucleus 11B
39.26
Me
N
TBS
B
Mes
Me
f1 (ppm)

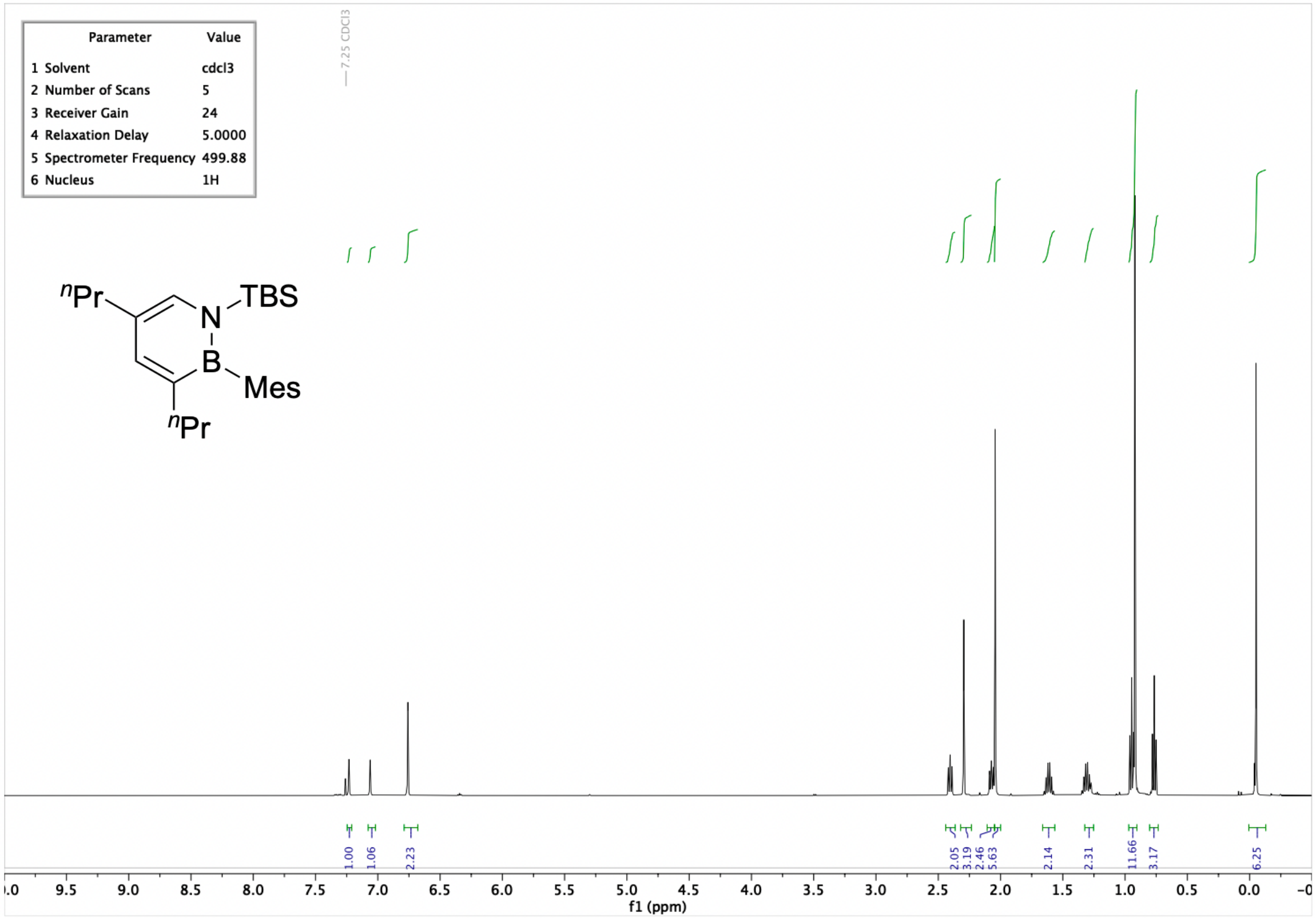
Parameter Value
1 Solvent cdcl3
2 Number of Scans 5
3 Receiver Gain 24
4 Relaxation Delay 5.0000
5 Spectrometer Frequency 499.88
6 Nucleus 1H
nPr
N
TBS
B
Mes
nPr
7.25 CDCl3
f1 (ppm)

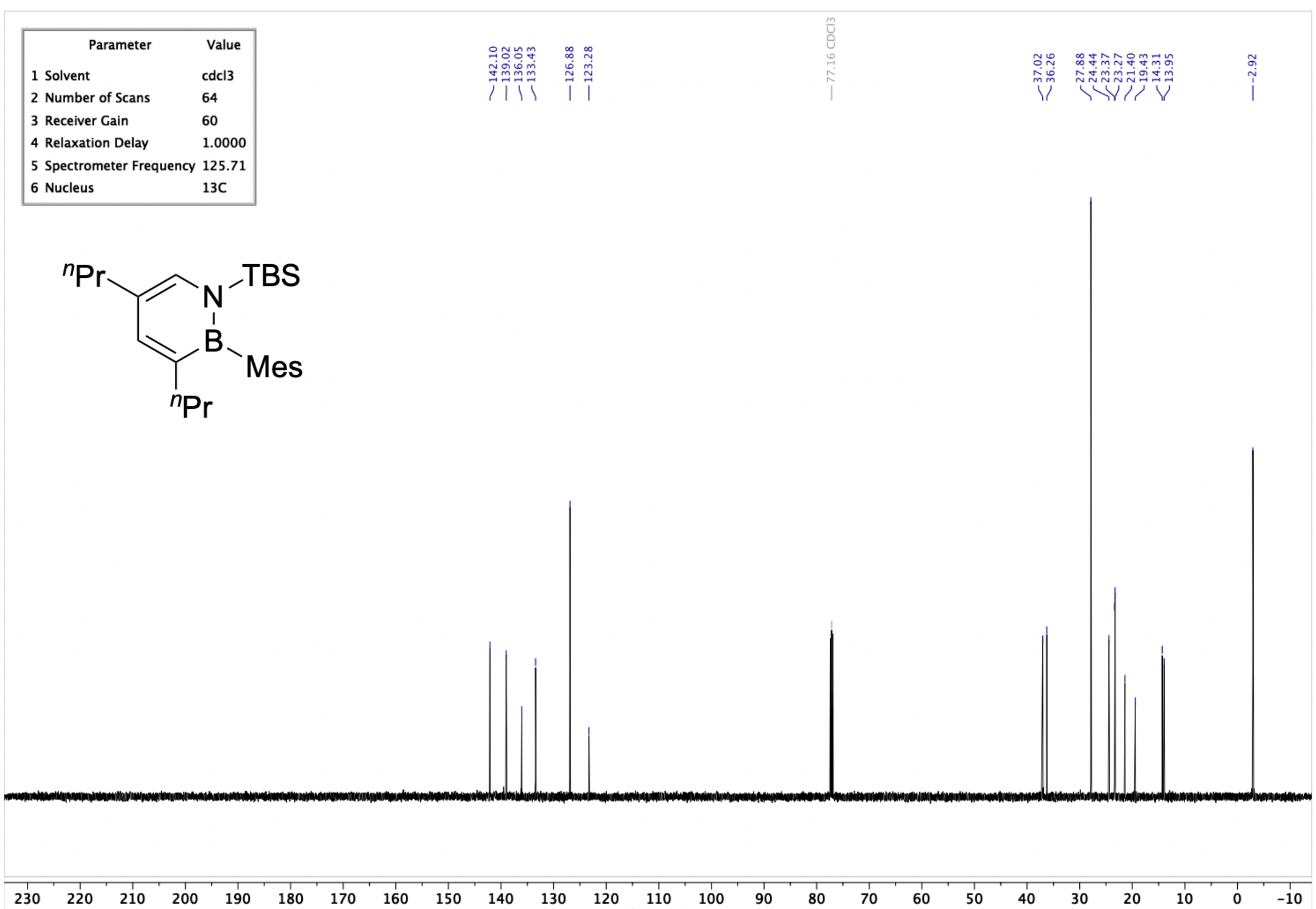
Parameter Value
1 Solvent cdcl3
2 Number of Scans 64
3 Receiver Gain 60
4 Relaxation Delay 1.0000
5 Spectrometer Frequency 125.71
6 Nucleus 13C
nPr
N
TBS
B
Mes
nPr
142.10
139.02
136.05
133.43
126.88
123.28
77.16 CDCl3
37.02
36.26
27.88
24.44
23.37
23.27
21.40
19.43
14.31
13.95
-2.92
f1 (ppm)

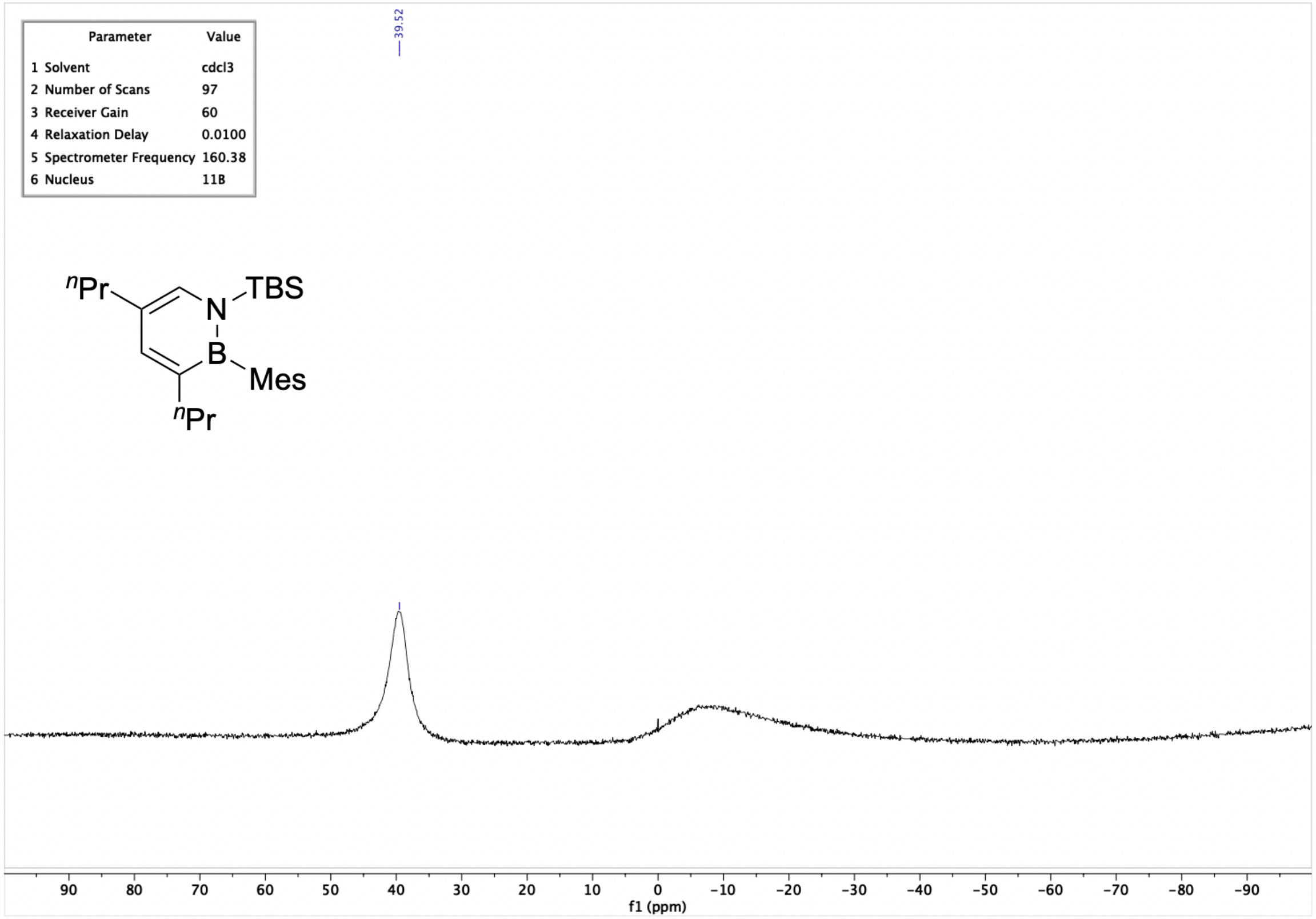

Parameter Value
1 Solvent cdcl3
2 Number of Scans 97
3 Receiver Gain 60
4 Relaxation Delay 0.0100
5 Spectrometer Frequency 160.38
6 Nucleus 11B
39.52
nPr
N
TBS
B
Mes
nPr
f1 (ppm)

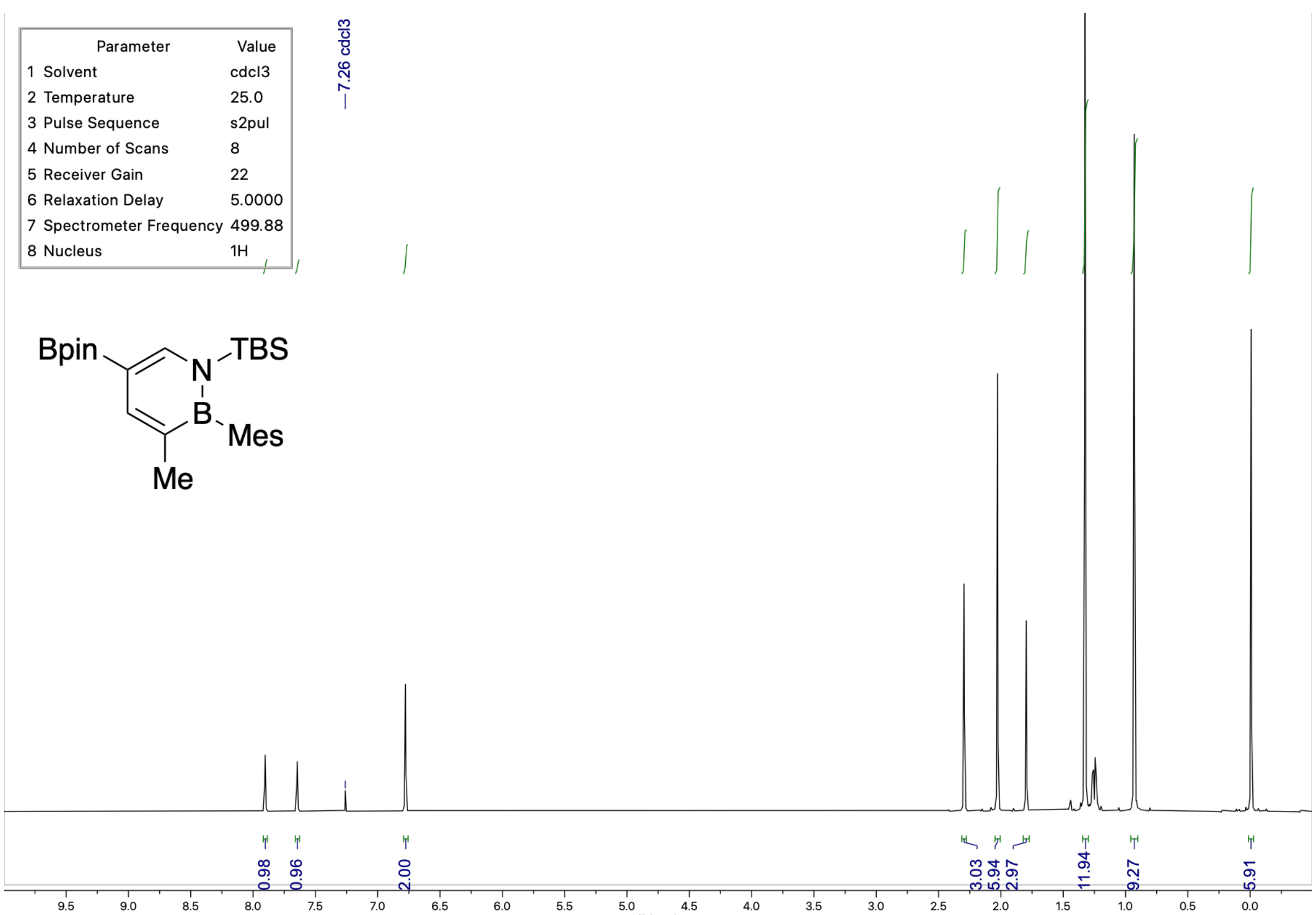

Parameter Value
1 Solvent cdcl3
2 Temperature 25.0
3 Pulse Sequence s2pul
4 Number of Scans 8
5 Receiver Gain 22
6 Relaxation Delay 5.0000
7 Spectrometer Frequency 499.88
8 Nucleus 1H
7.26 cdcl3
Bpin
N
TBS
B
Mes
Me
0.98
0.96
2.00
3.03
5.94
2.97
11.94
9.27
5.91
f1 (ppm)

Parameter	Value
1 Solvent	cdcl3
2 Temperature	25.0
3 Pulse Sequence	s2pul
4 Number of Scans	84
5 Receiver Gain	30
6 Relaxation Delay	1.0000
7 Spectrometer Frequency	125.71
8 Nucleus	13C

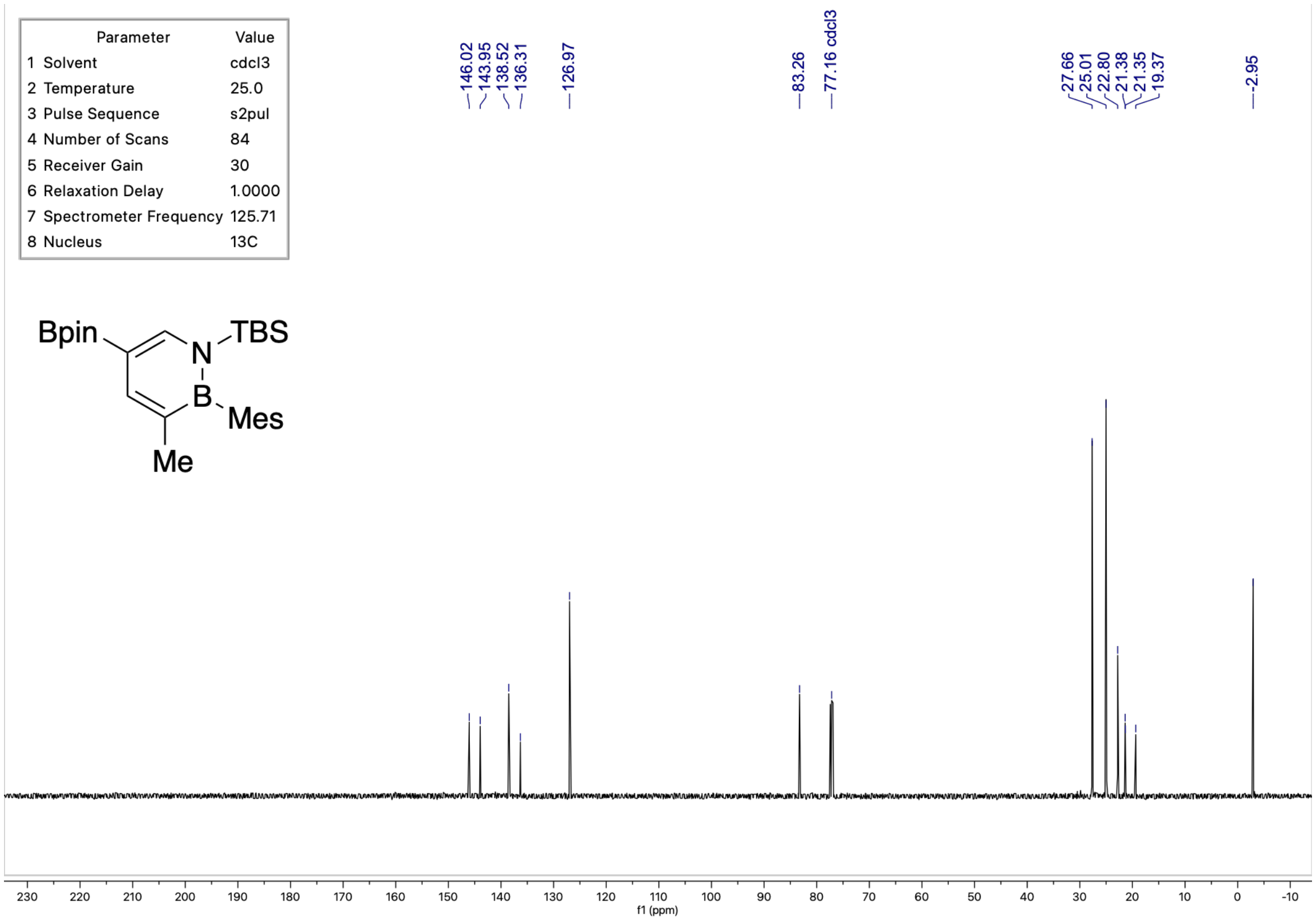

Parameter	Value
1 Solvent	cdcl3
2 Temperature	25.0
3 Pulse Sequence	s2pul
4 Number of Scans	256
5 Receiver Gain	30
6 Relaxation Delay	0.0100
7 Spectrometer Frequency	160.38
8 Nucleus	11B

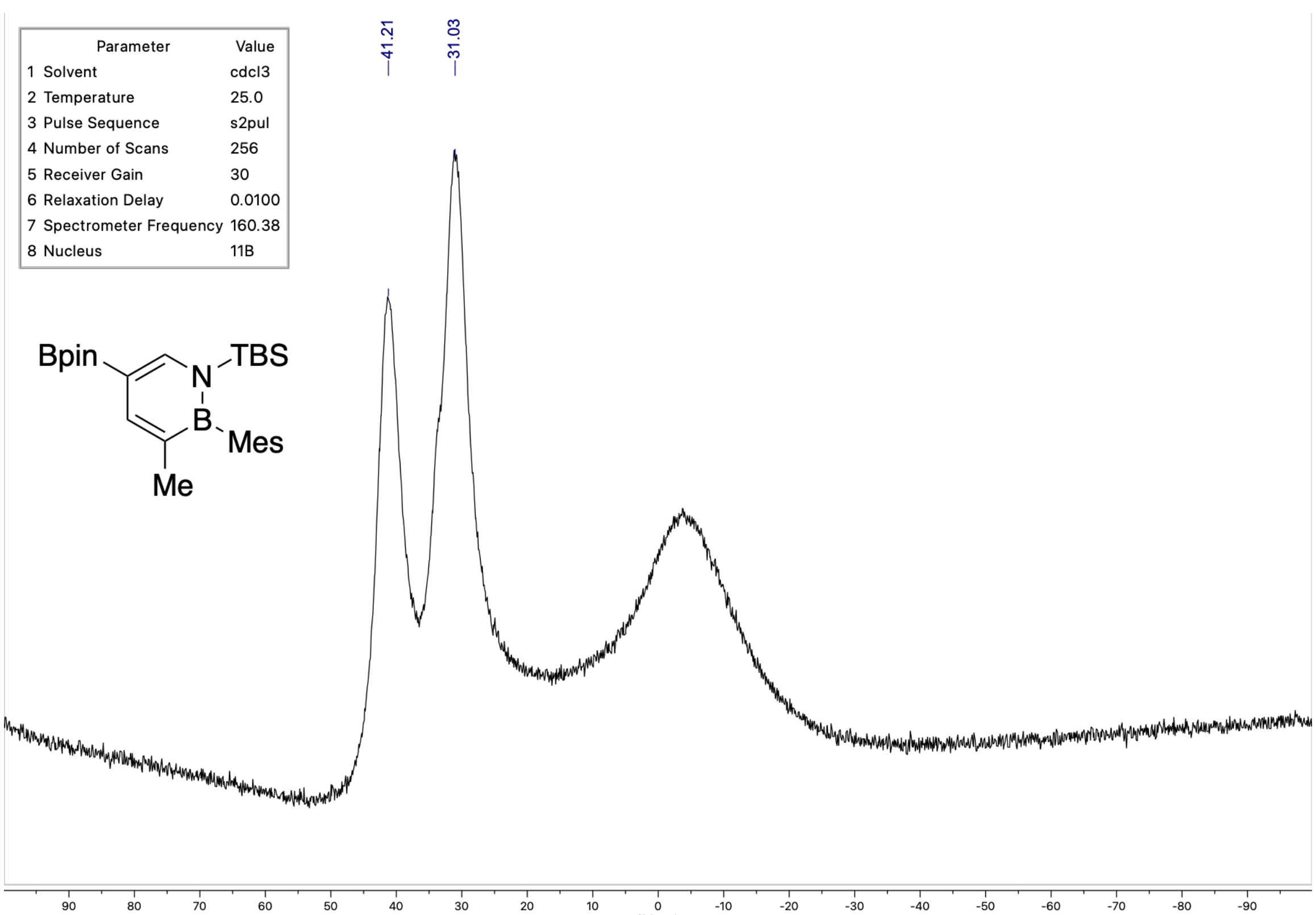

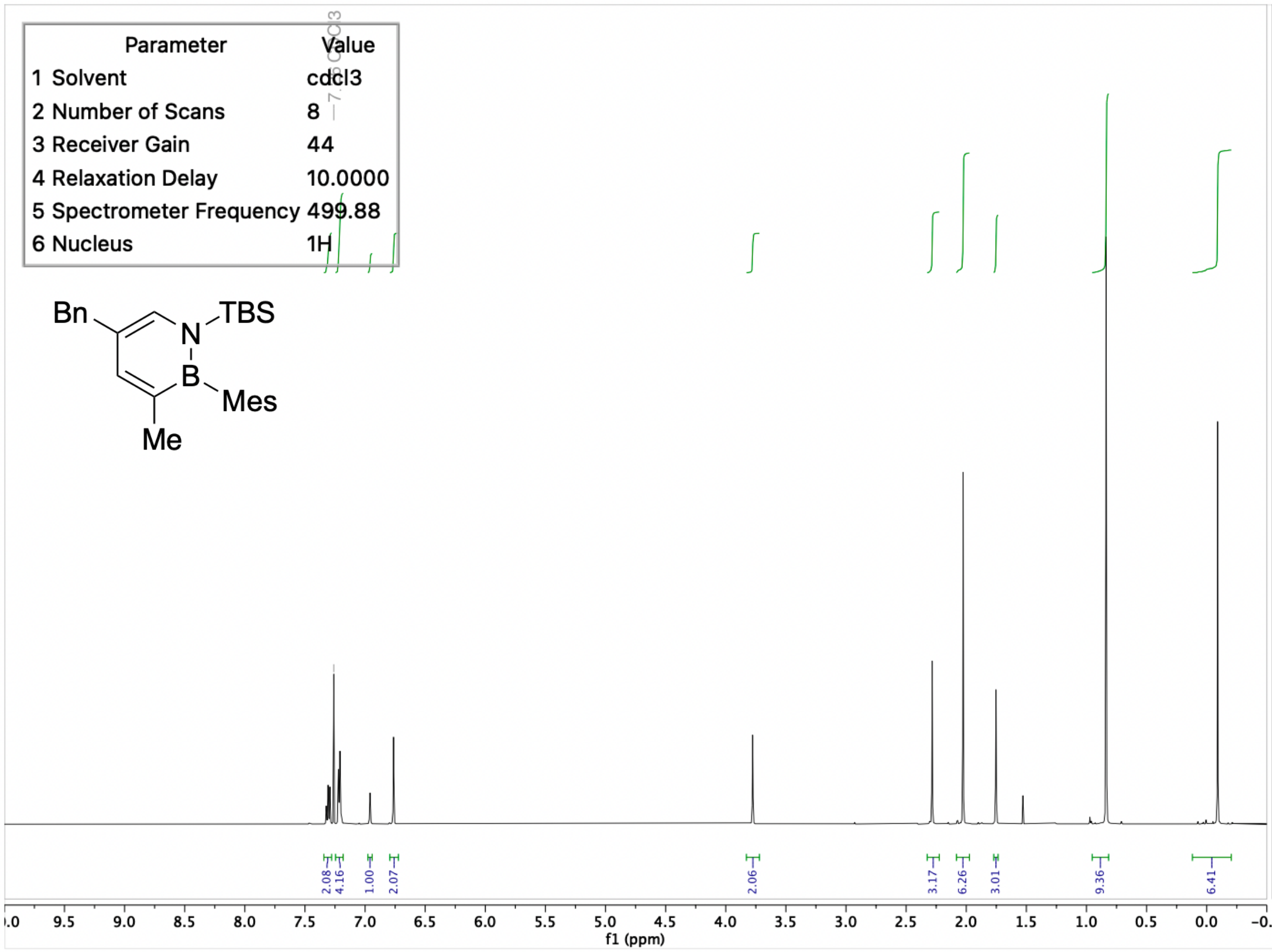

Parameter Value
1 Solvent cdcl3
2 Number of Scans 8
3 Receiver Gain 44
4 Relaxation Delay 10.0000
5 Spectrometer Frequency 499.88
6 Nucleus 1H
Bn
N
TBS
B
Mes
Me
2.08
4.16
1.00
2.07
2.06
3.17
6.26
3.01
9.36
6.41
f1 (ppm)

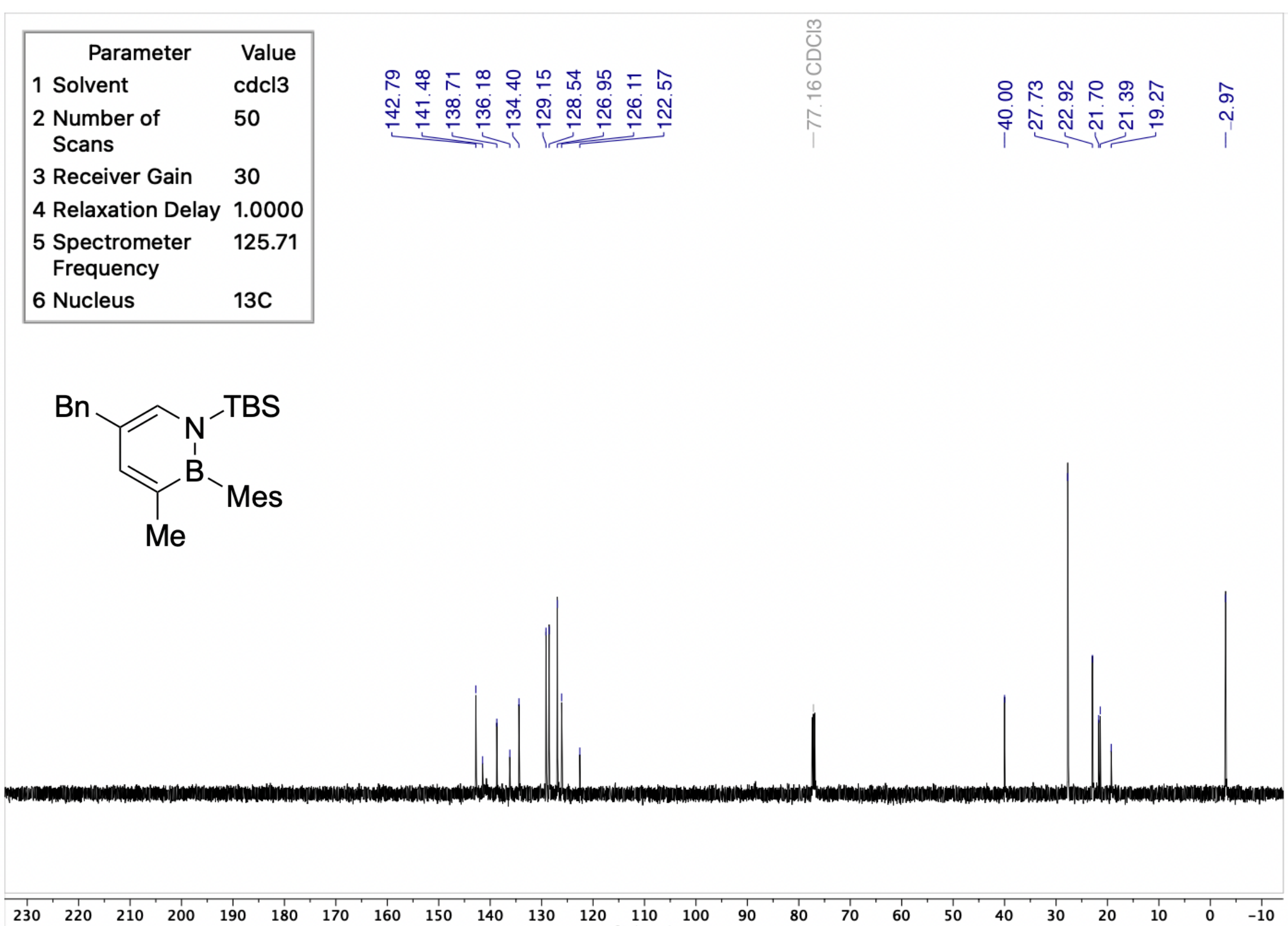

Parameter Value
1 Solvent cdcl3
2 Number of Scans 50
3 Receiver Gain 30
4 Relaxation Delay 1.0000
5 Spectrometer Frequency 125.71
6 Nucleus 13C
142.79
141.48
138.71
136.18
134.40
129.15
128.54
126.95
126.11
122.57
77.16 CDCl3
40.00
27.73
22.92
21.70
21.39
19.27
-2.97
Bn
N
TBS
B
Mes
Me
f1 (ppm)

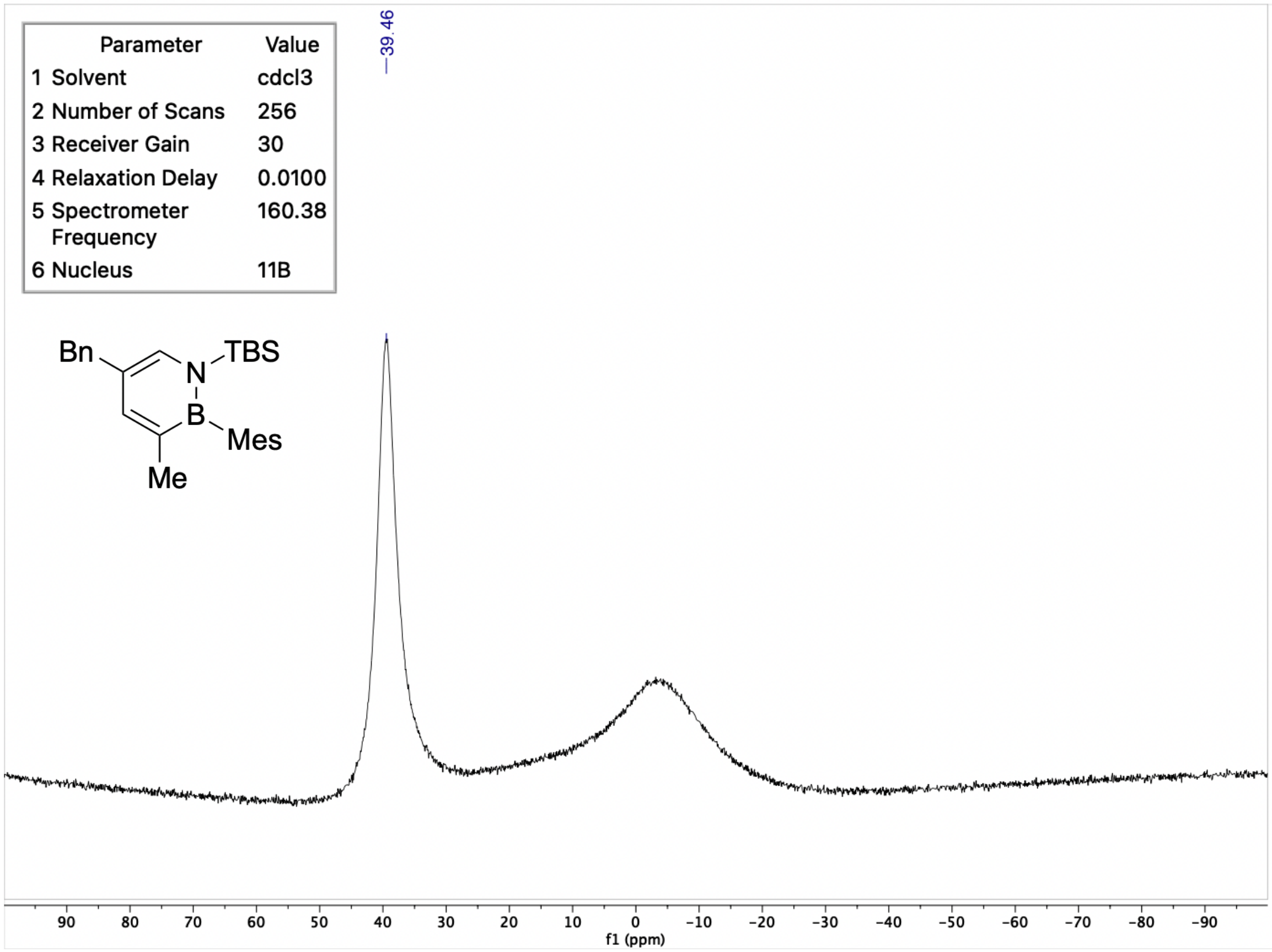
Parameter Value
1 Solvent cdcl3
2 Number of Scans 256
3 Receiver Gain 30
4 Relaxation Delay 0.0100
5 Spectrometer Frequency 160.38
6 Nucleus 11B
39.46
Bn
N
TBS
B
Mes
Me
f1 (ppm)

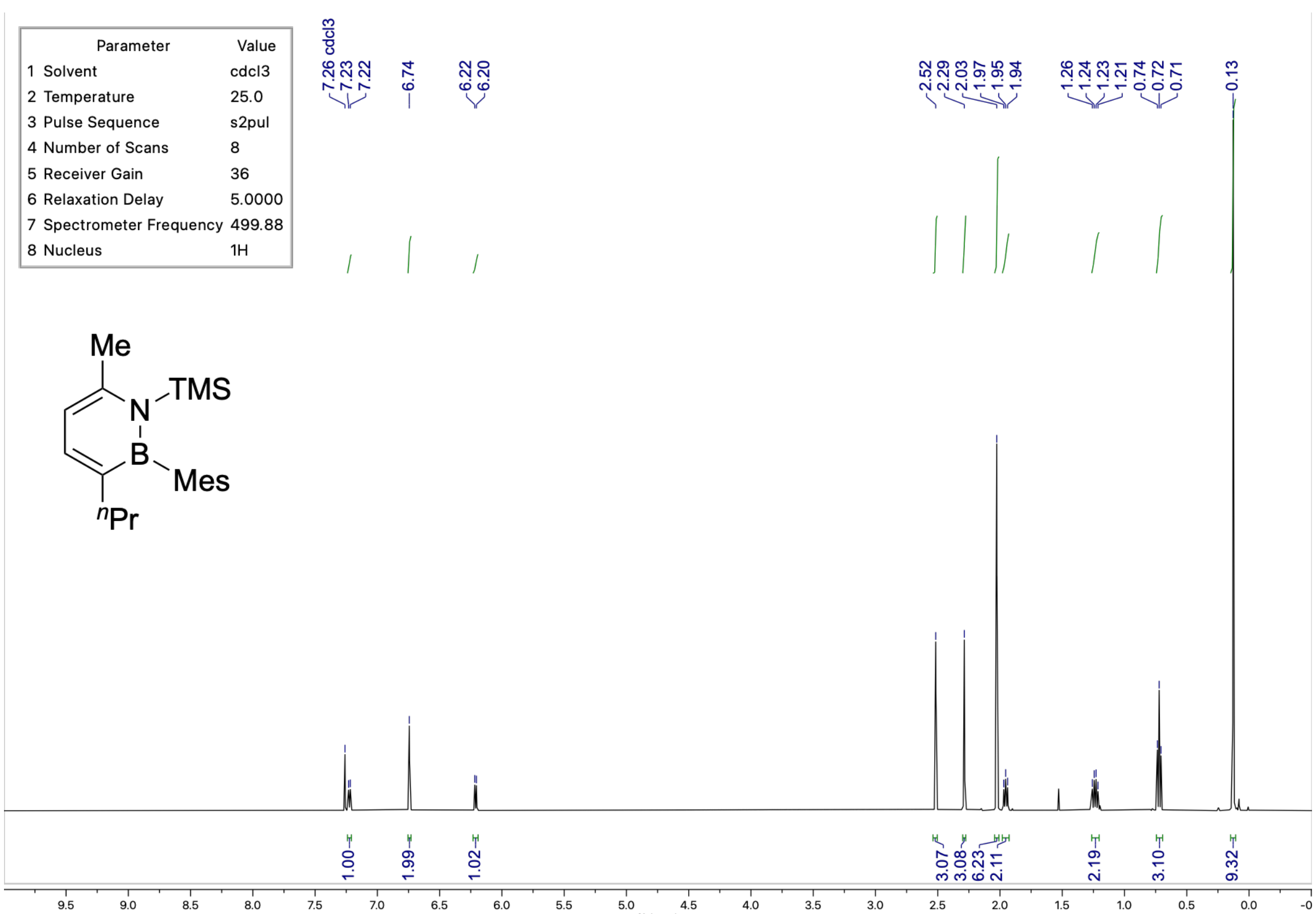
Parameter Value
1 Solvent cdcl3
2 Temperature 25.0
3 Pulse Sequence s2pul
4 Number of Scans 8
5 Receiver Gain 36
6 Relaxation Delay 5.0000
7 Spectrometer Frequency 499.88
8 Nucleus 1H
7.26 cdcl3
7.23
7.22
6.74
6.22
6.20
2.52
2.29
2.03
1.97
1.95
1.94
1.26
1.24
1.23
1.21
0.74
0.72
0.71
0.13
Me
N
TMS
B
Mes
nPr
1.00
1.99
1.02
3.07
3.08
6.23
2.11
2.19
3.10
9.32
f1 (ppm)

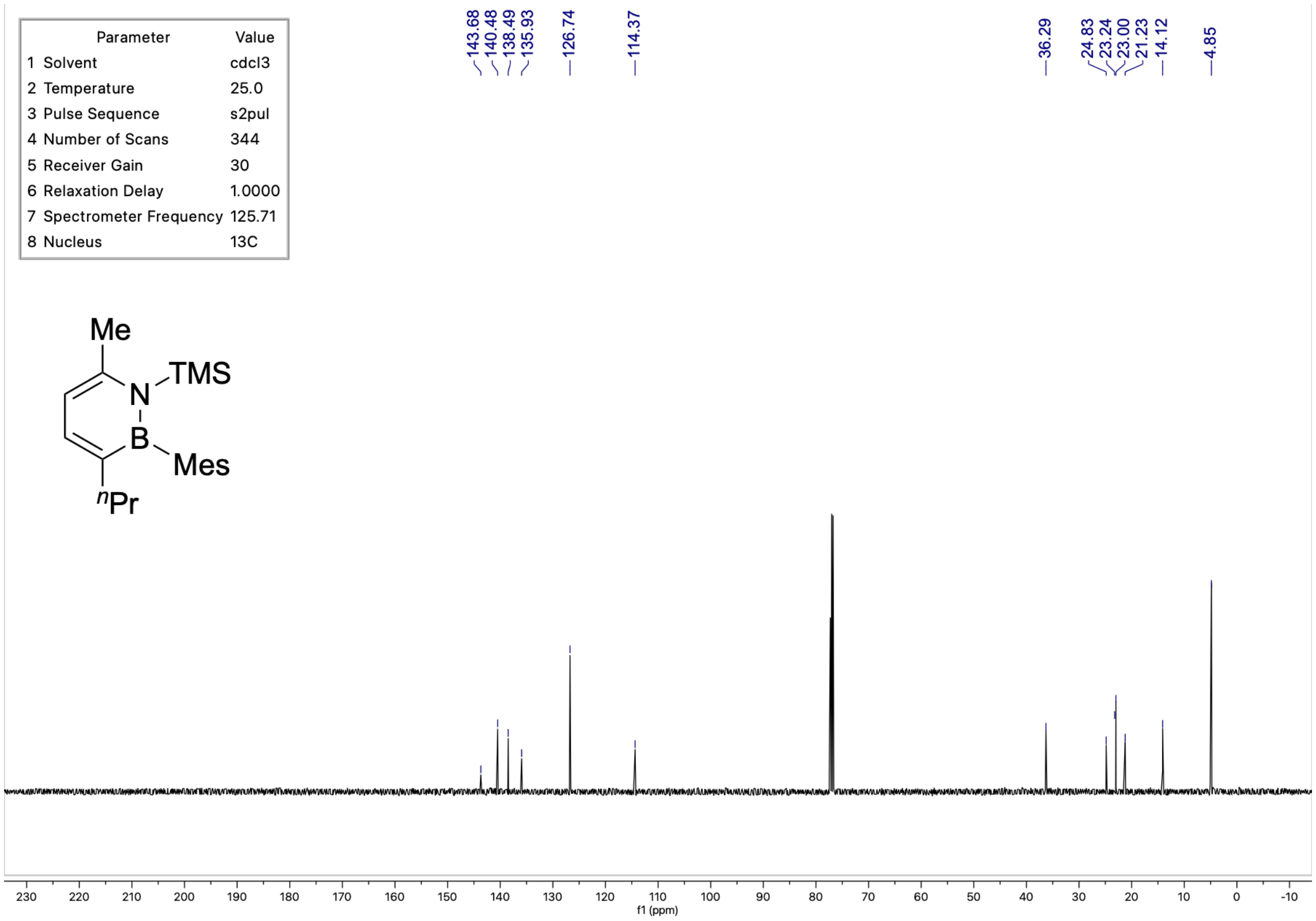
Parameter Value
1 Solvent cdcl3
2 Temperature 25.0
3 Pulse Sequence s2pul
4 Number of Scans 344
5 Receiver Gain 30
6 Relaxation Delay 1.0000
7 Spectrometer Frequency 125.71
8 Nucleus 13C
143.68
140.48
138.49
135.93
126.74
114.37
36.29
24.83
23.24
23.00
21.23
14.12
4.85
Me
N
TMS
B
Mes
nPr
f1 (ppm)

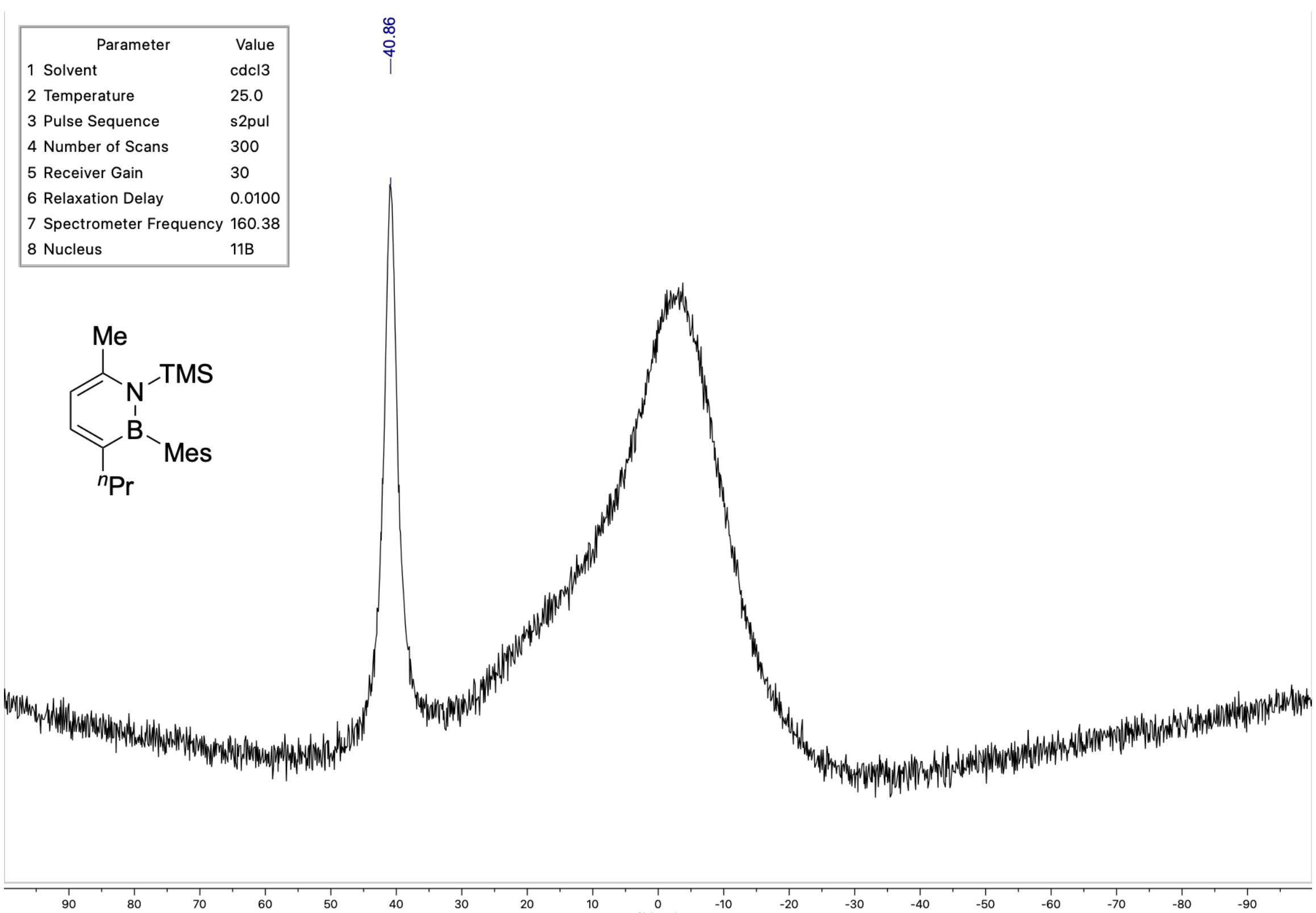
Parameter Value
1 Solvent cdcl3
2 Temperature 25.0
3 Pulse Sequence s2pul
4 Number of Scans 300
5 Receiver Gain 30
6 Relaxation Delay 0.0100
7 Spectrometer Frequency 160.38
8 Nucleus 11B
40.86
Me
N
TMS
B
Mes
nPr
f1 (ppm)

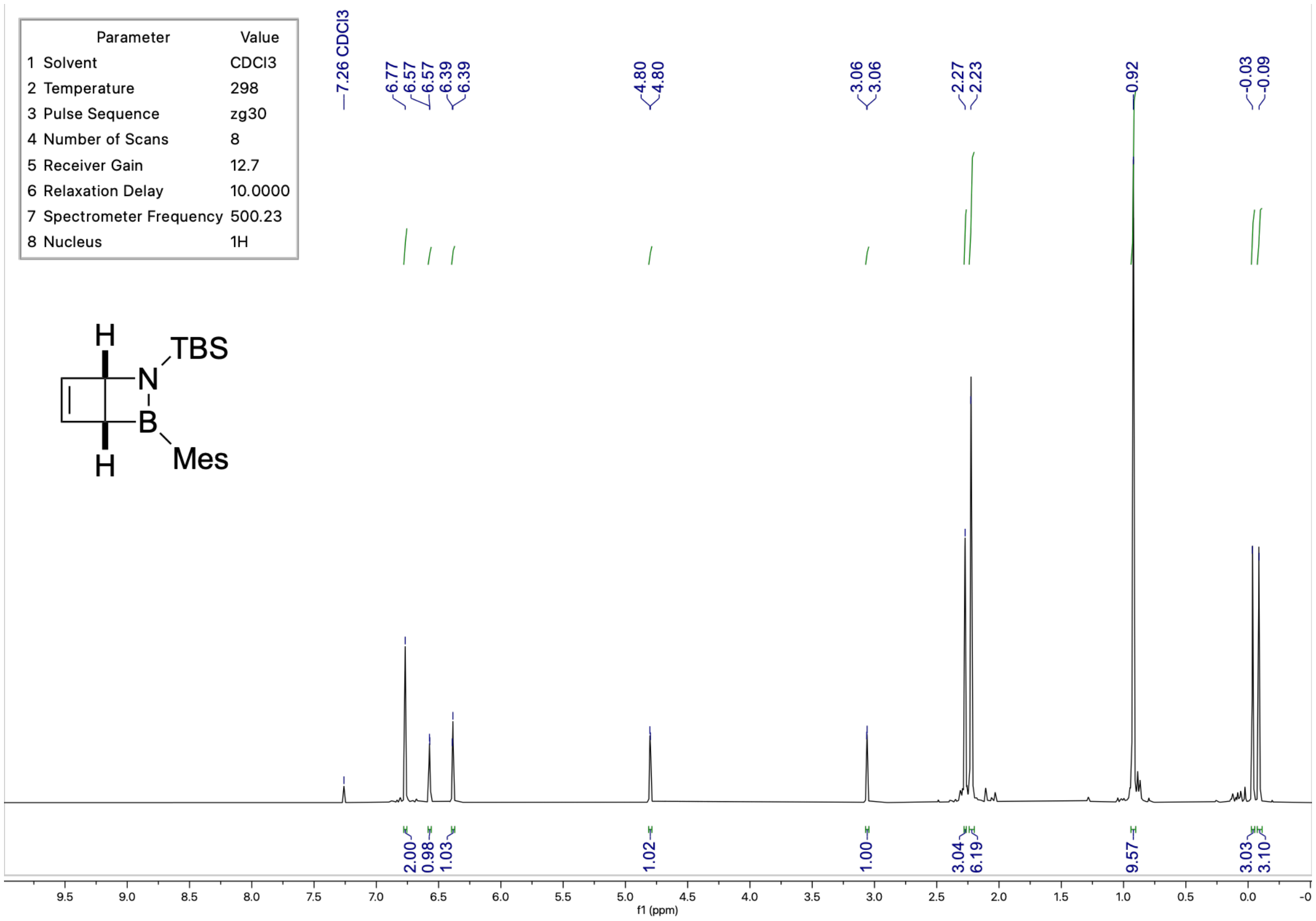
Parameter Value
1 Solvent CDCl3
2 Temperature 298
3 Pulse Sequence zg30
4 Number of Scans 8
5 Receiver Gain 12.7
6 Relaxation Delay 10.0000
7 Spectrometer Frequency 500.23
8 Nucleus 1H
TBS
Mes
f1 (ppm)

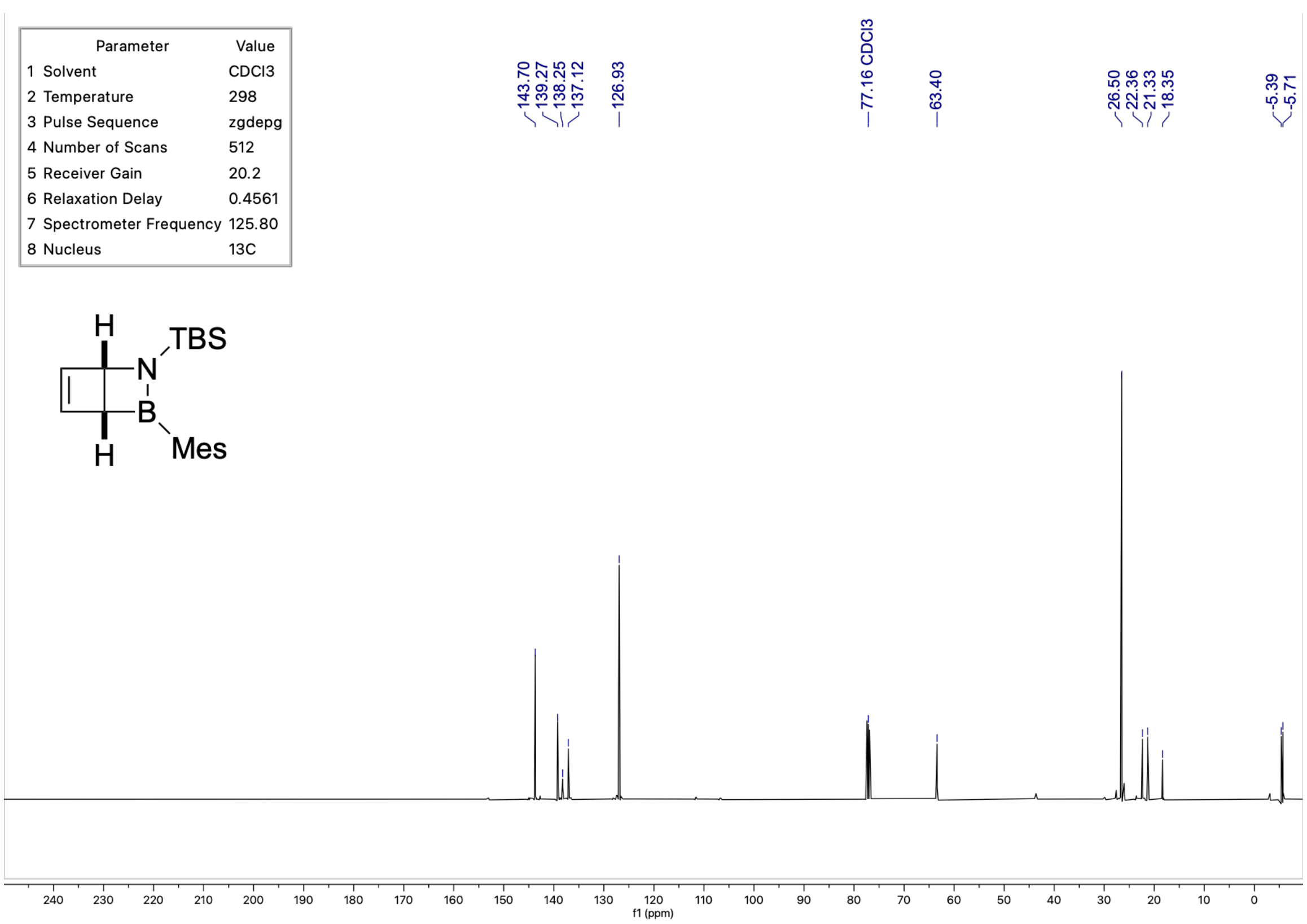
Parameter Value
1 Solvent CDCl3
2 Temperature 298
3 Pulse Sequence zgdepg
4 Number of Scans 512
5 Receiver Gain 20.2
6 Relaxation Delay 0.4561
7 Spectrometer Frequency 125.80
8 Nucleus 13C
TBS
Mes
f1 (ppm)

Parameter	Value
1 Solvent	CDCl3
2 Temperature	298
3 Pulse Sequence	zgbs
4 Number of Scans	1000
5 Receiver Gain	22.6
6 Relaxation Delay	0.0100
7 Spectrometer Frequency	160.50
8 Nucleus	11B

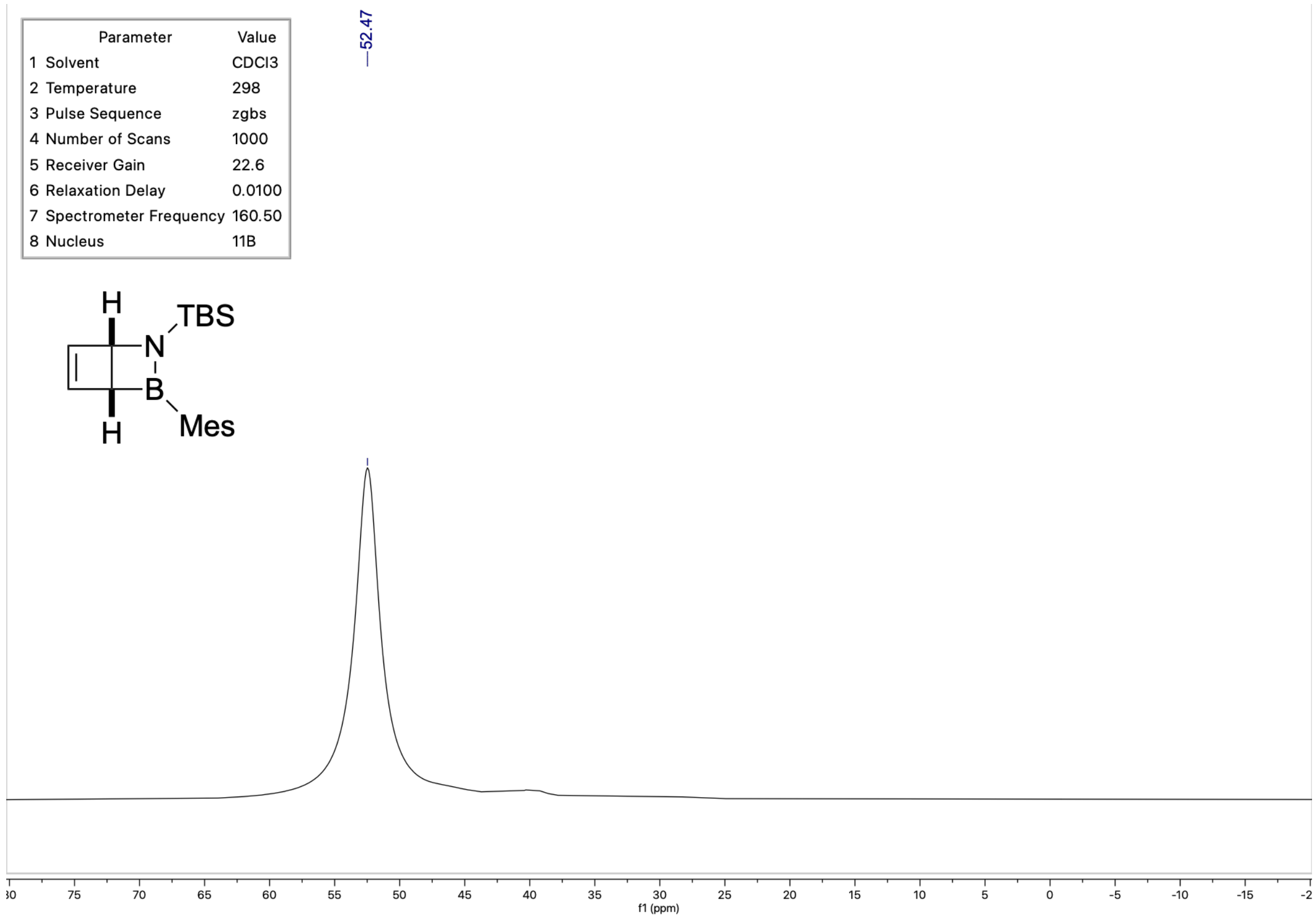

Parameter	Value
1 Solvent	cdcl3
2 Temperature	25.0
3 Pulse Sequence	s2pul
4 Number of Scans	8
5 Receiver Gain	34
6 Relaxation Delay	10.0000
7 Spectrometer Frequency	499.88
8 Nucleus	1H

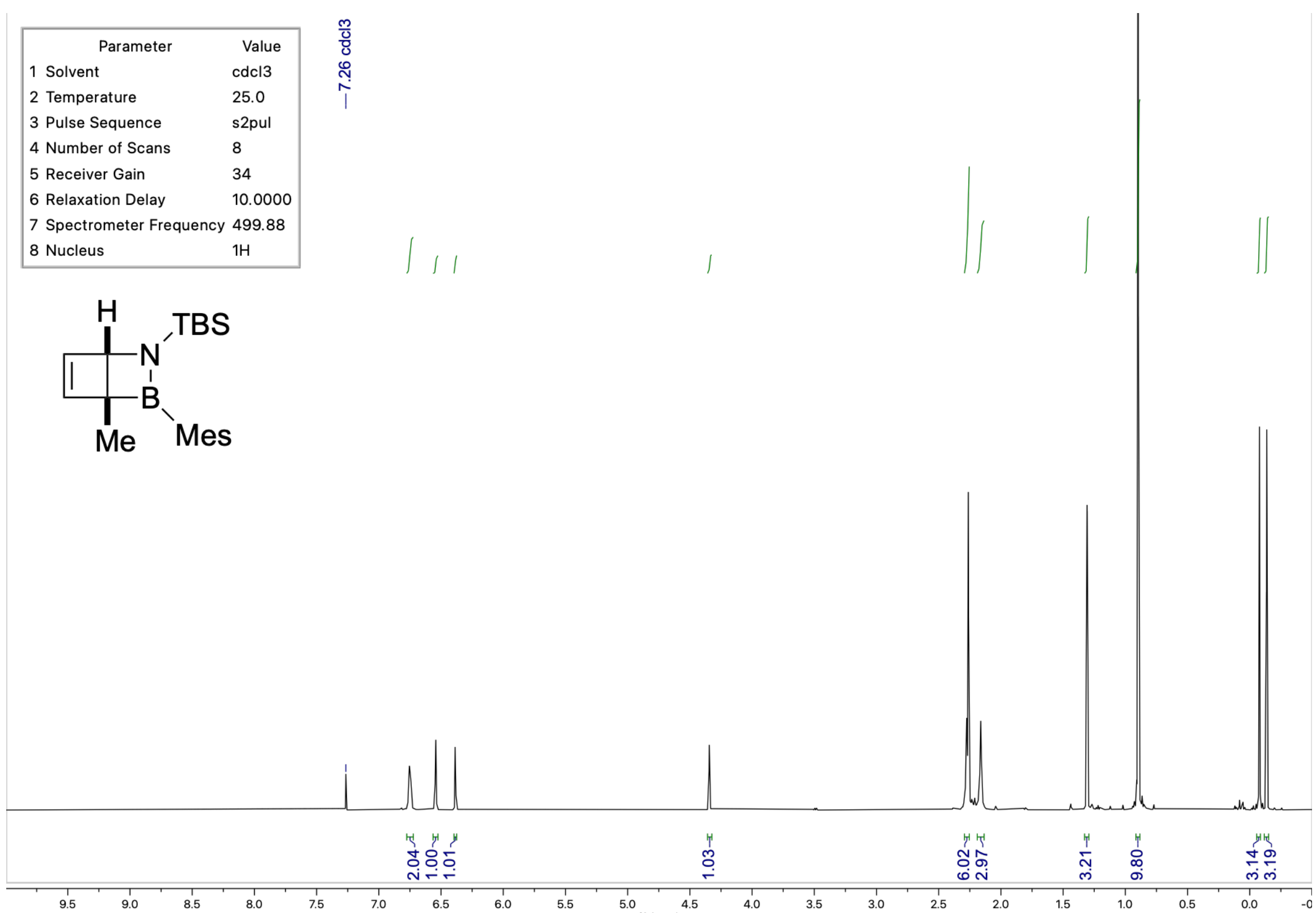

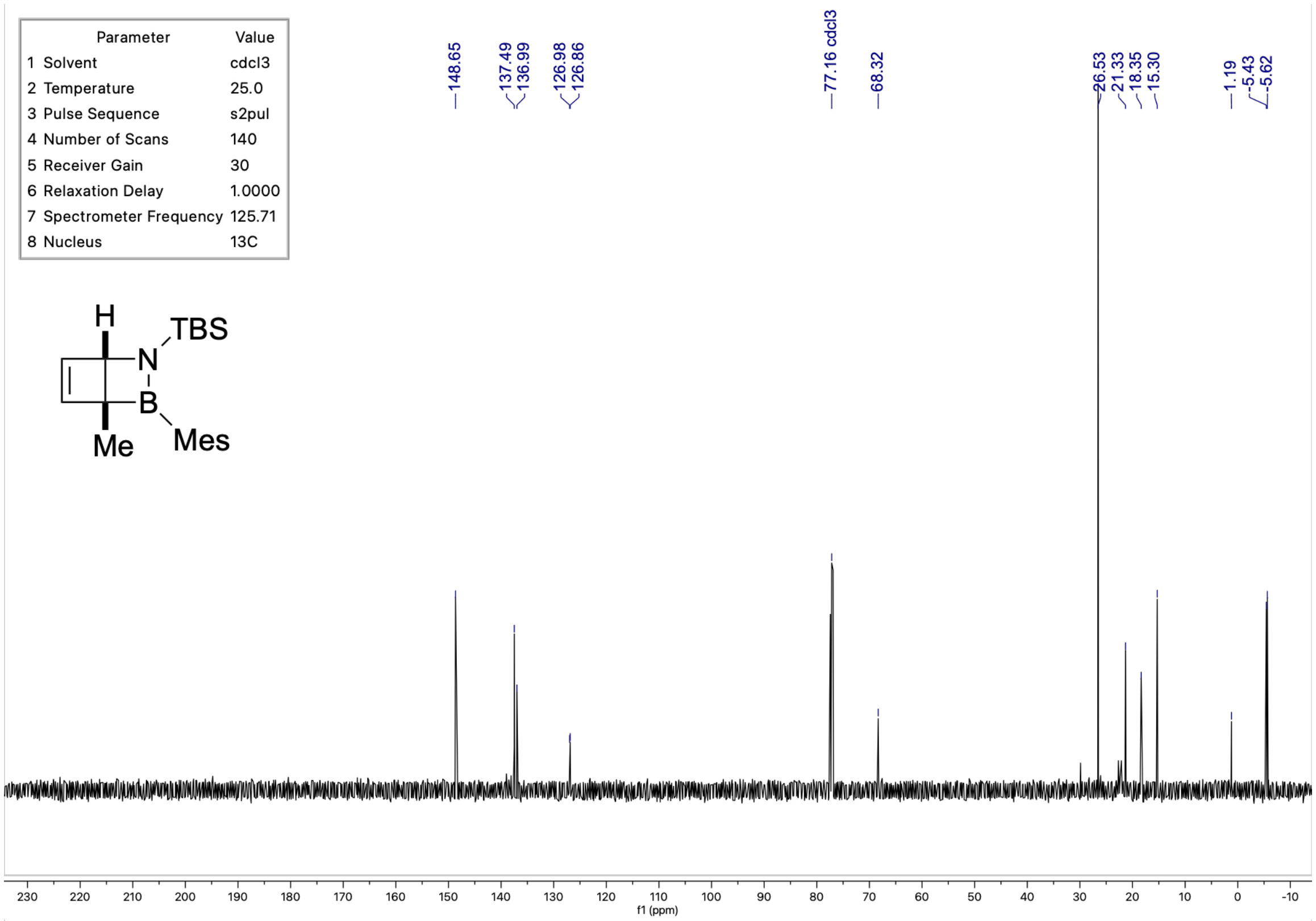
Parameter Value
1 Solvent cdcl3
2 Temperature 25.0
3 Pulse Sequence s2pul
4 Number of Scans 140
5 Receiver Gain 30
6 Relaxation Delay 1.0000
7 Spectrometer Frequency 125.71
8 Nucleus 13C
H
TBS
N
B
Me
Mes
148.65
137.49
136.99
126.98
126.86
77.16 cdcl3
68.32
26.53
21.33
18.35
15.30
1.19
-5.43
-5.62
f1 (ppm)

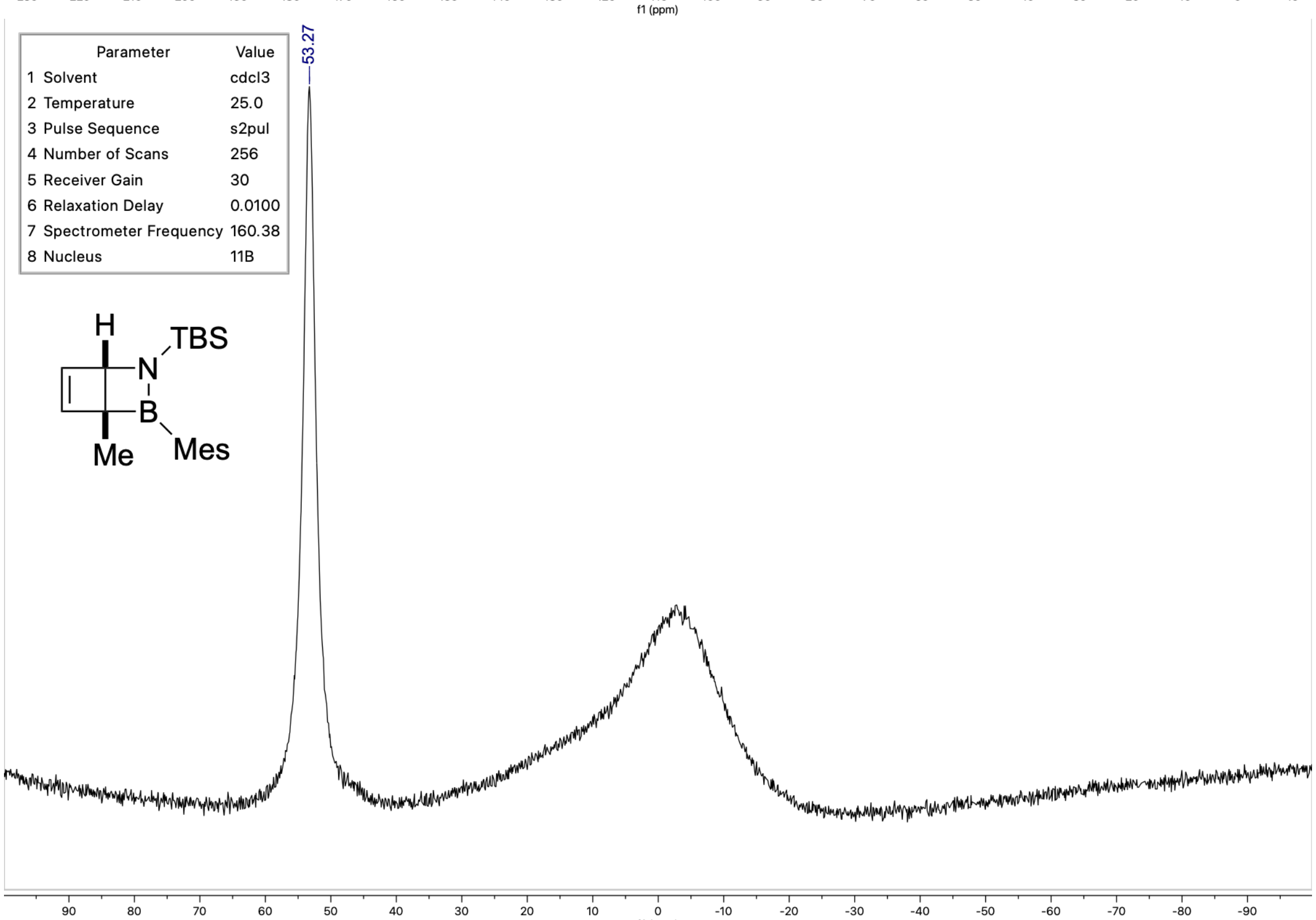
Parameter Value
1 Solvent cdcl3
2 Temperature 25.0
3 Pulse Sequence s2pul
4 Number of Scans 256
5 Receiver Gain 30
6 Relaxation Delay 0.0100
7 Spectrometer Frequency 160.38
8 Nucleus 11B
H
TBS
N
B
Me
Mes
53.27
f1 (ppm)

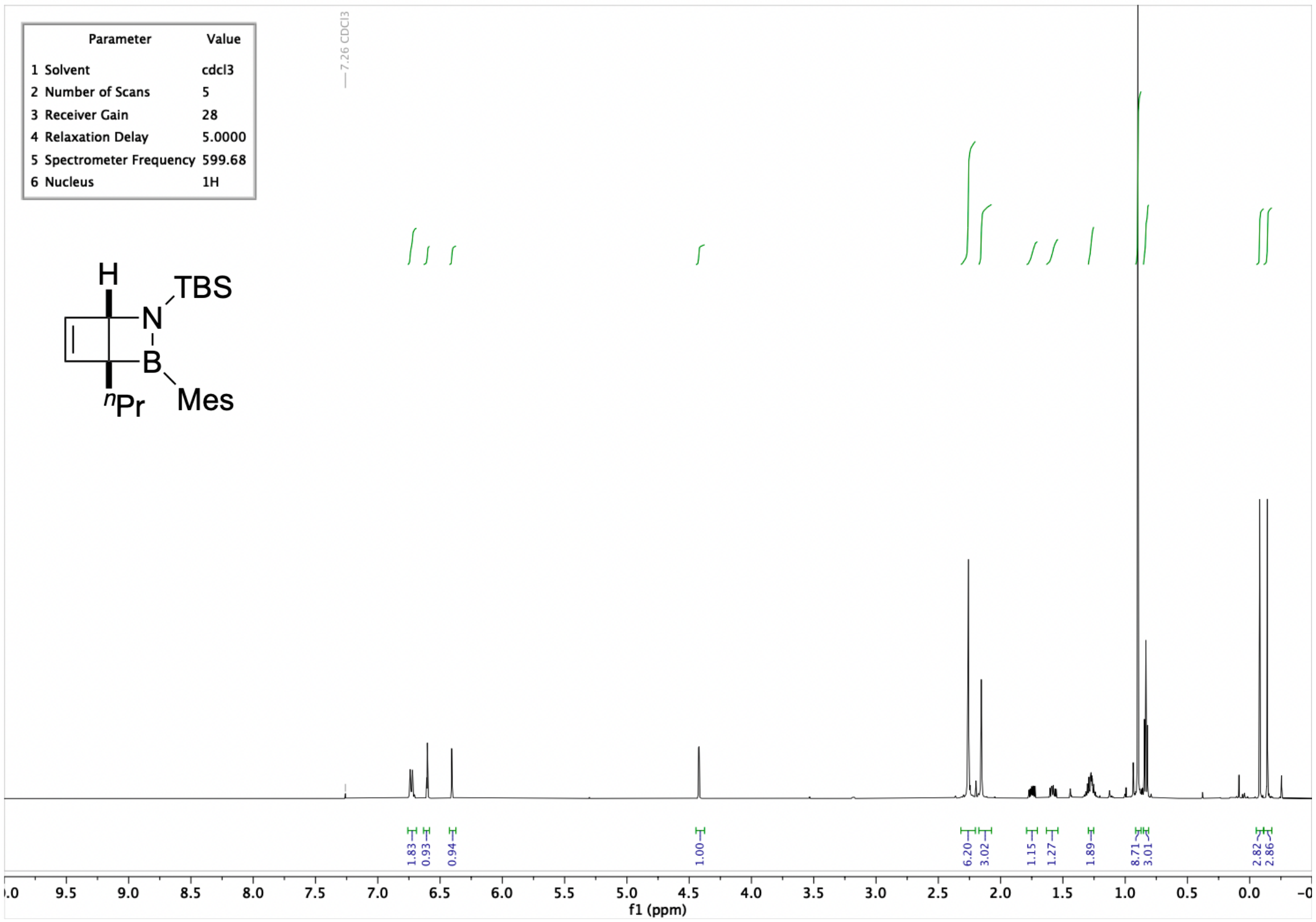
Parameter Value
1 Solvent cdcl3
2 Number of Scans 5
3 Receiver Gain 28
4 Relaxation Delay 5.0000
5 Spectrometer Frequency 599.68
6 Nucleus 1H
7.26 CDCl3
H
TBS
N
B
nPr
Mes
1.83
0.93
0.94
1.00
6.20
3.02
1.15
1.27
1.89
8.71
3.01
2.82
2.86
f1 (ppm)

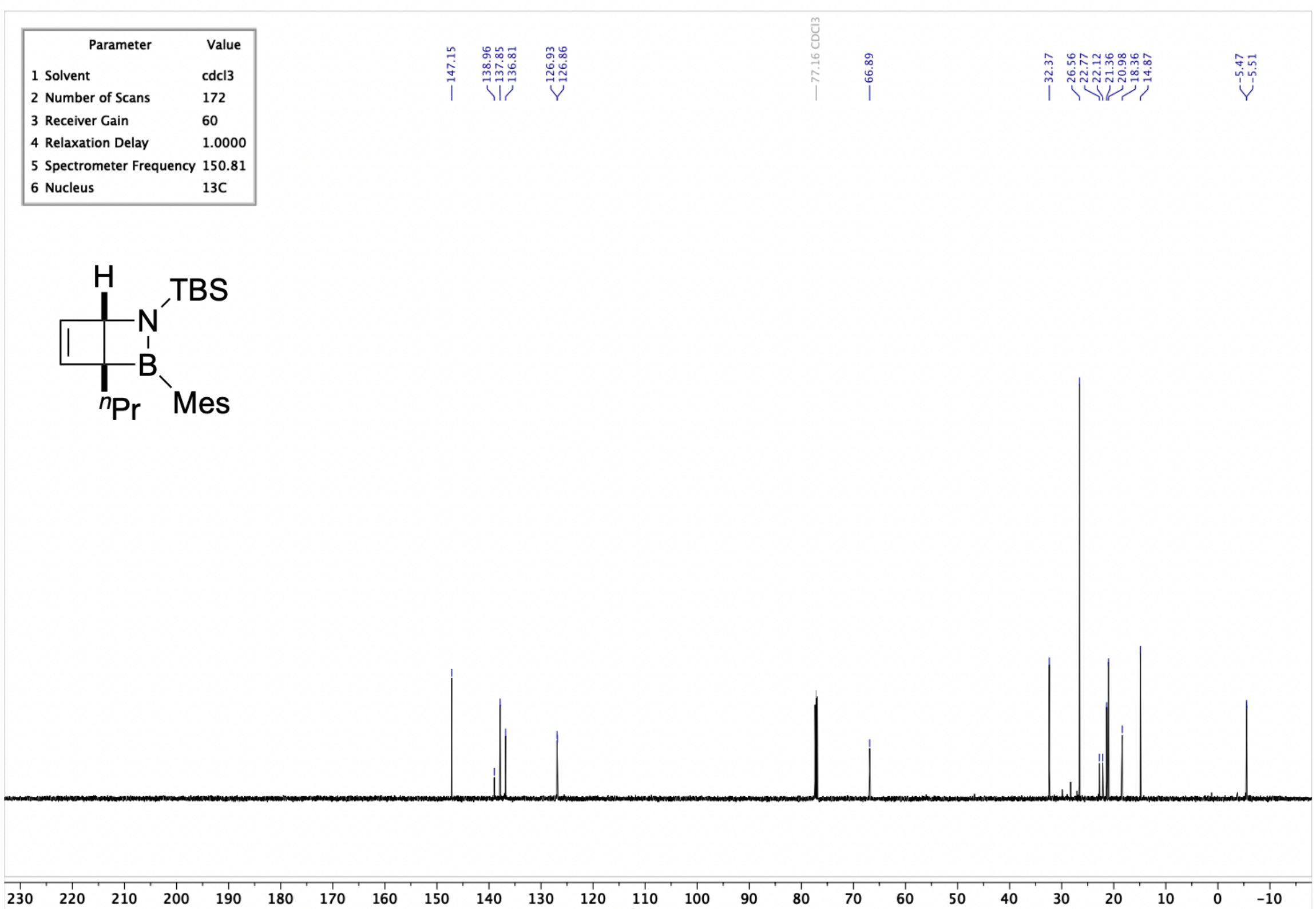
Parameter Value
1 Solvent cdcl3
2 Number of Scans 172
3 Receiver Gain 60
4 Relaxation Delay 1.0000
5 Spectrometer Frequency 150.81
6 Nucleus 13C
147.15
138.96
137.85
136.81
126.93
126.86
77.16 CDCl3
66.89
32.37
26.56
22.77
22.12
21.36
20.98
18.36
14.87
-5.47
-5.51
H
TBS
N
B
nPr
Mes
f1 (ppm)

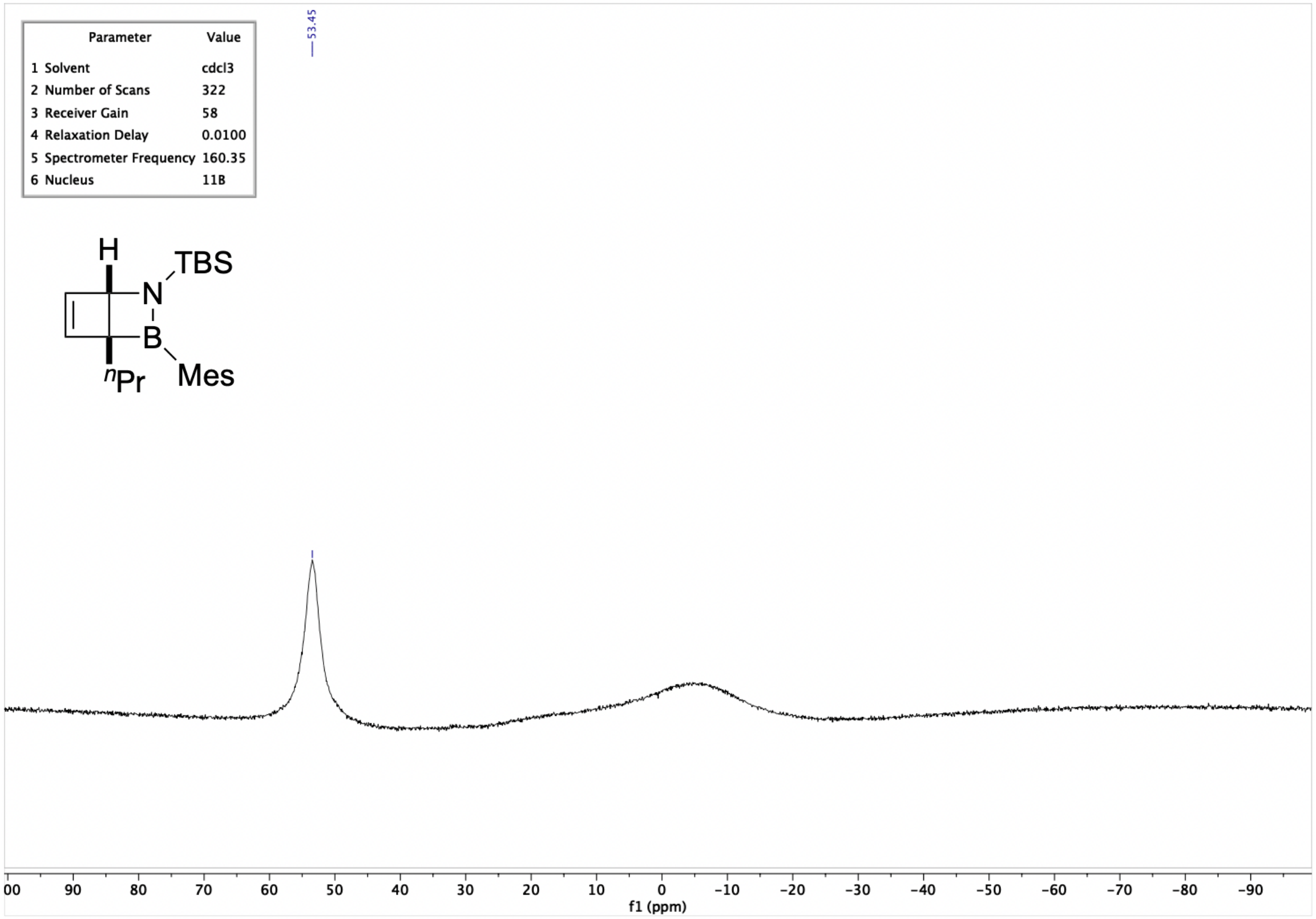
Parameter Value
1 Solvent cdcl3
2 Number of Scans 322
3 Receiver Gain 58
4 Relaxation Delay 0.0100
5 Spectrometer Frequency 160.35
6 Nucleus 11B
53.45
H
TBS
N
B
ⁿPr
Mes
f1 (ppm)

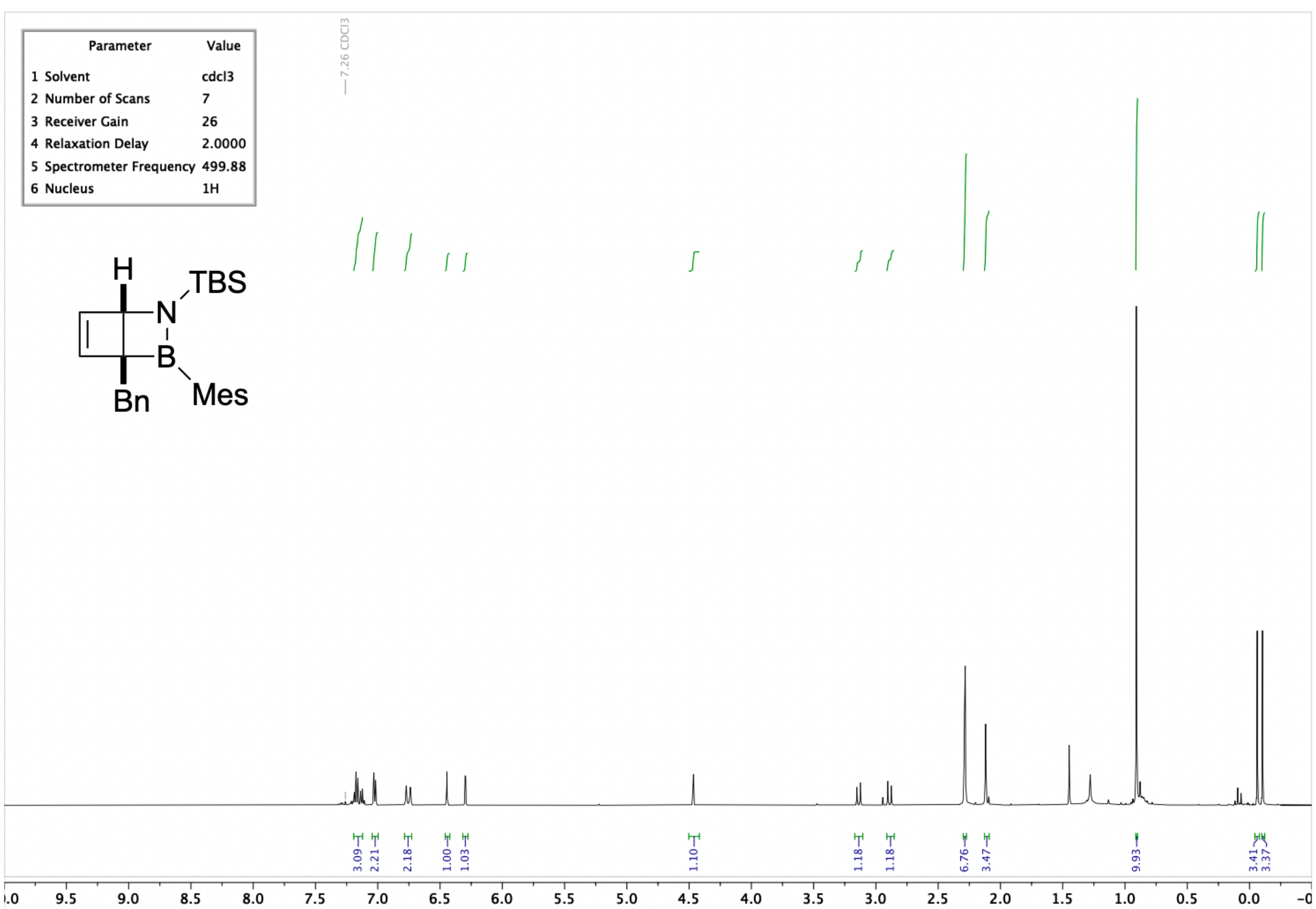
Parameter Value
1 Solvent cdcl3
2 Number of Scans 7
3 Receiver Gain 26
4 Relaxation Delay 2.0000
5 Spectrometer Frequency 499.88
6 Nucleus 1H
7.26 CDCl3
H
TBS
N
B
Bn
Mes
3.09
2.21
2.18
1.00
1.03
1.10
1.18
1.18
6.76
3.47
9.93
3.41
3.37
f1 (ppm)

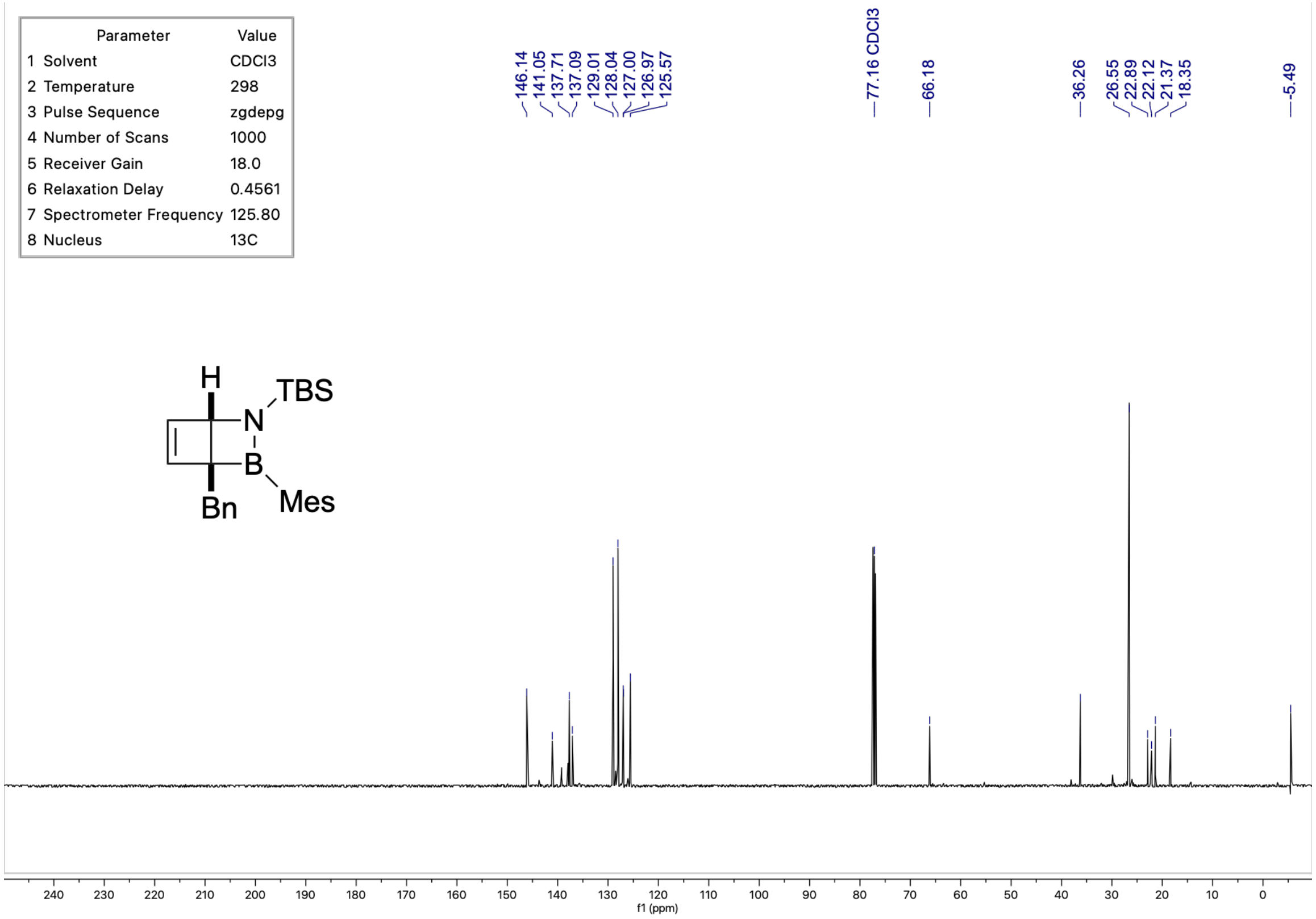

Parameter Value
1 Solvent CDCl3
2 Temperature 298
3 Pulse Sequence zgdepg
4 Number of Scans 1000
5 Receiver Gain 18.0
6 Relaxation Delay 0.4561
7 Spectrometer Frequency 125.80
8 Nucleus 13C
146.14
141.05
137.71
137.09
129.01
128.04
127.00
126.97
125.57
77.16 CDCl3
66.18
36.26
26.55
22.89
22.12
21.37
18.35
-5.49
H
TBS
N
B
Bn
Mes
f1 (ppm)

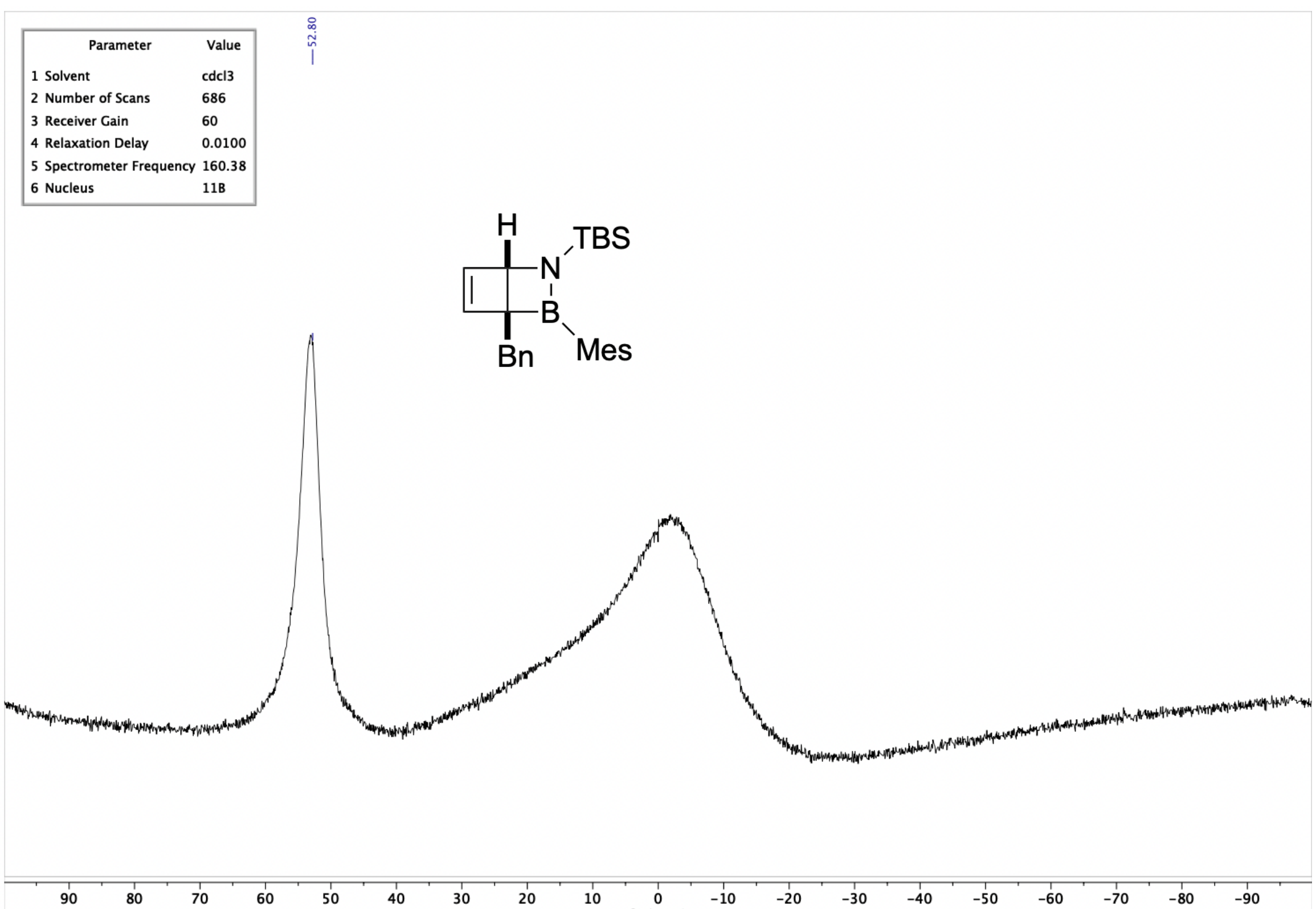

Parameter Value
1 Solvent cdcl3
2 Number of Scans 686
3 Receiver Gain 60
4 Relaxation Delay 0.0100
5 Spectrometer Frequency 160.38
6 Nucleus 11B
52.80
H
TBS
N
B
Bn
Mes
f1 (ppm)

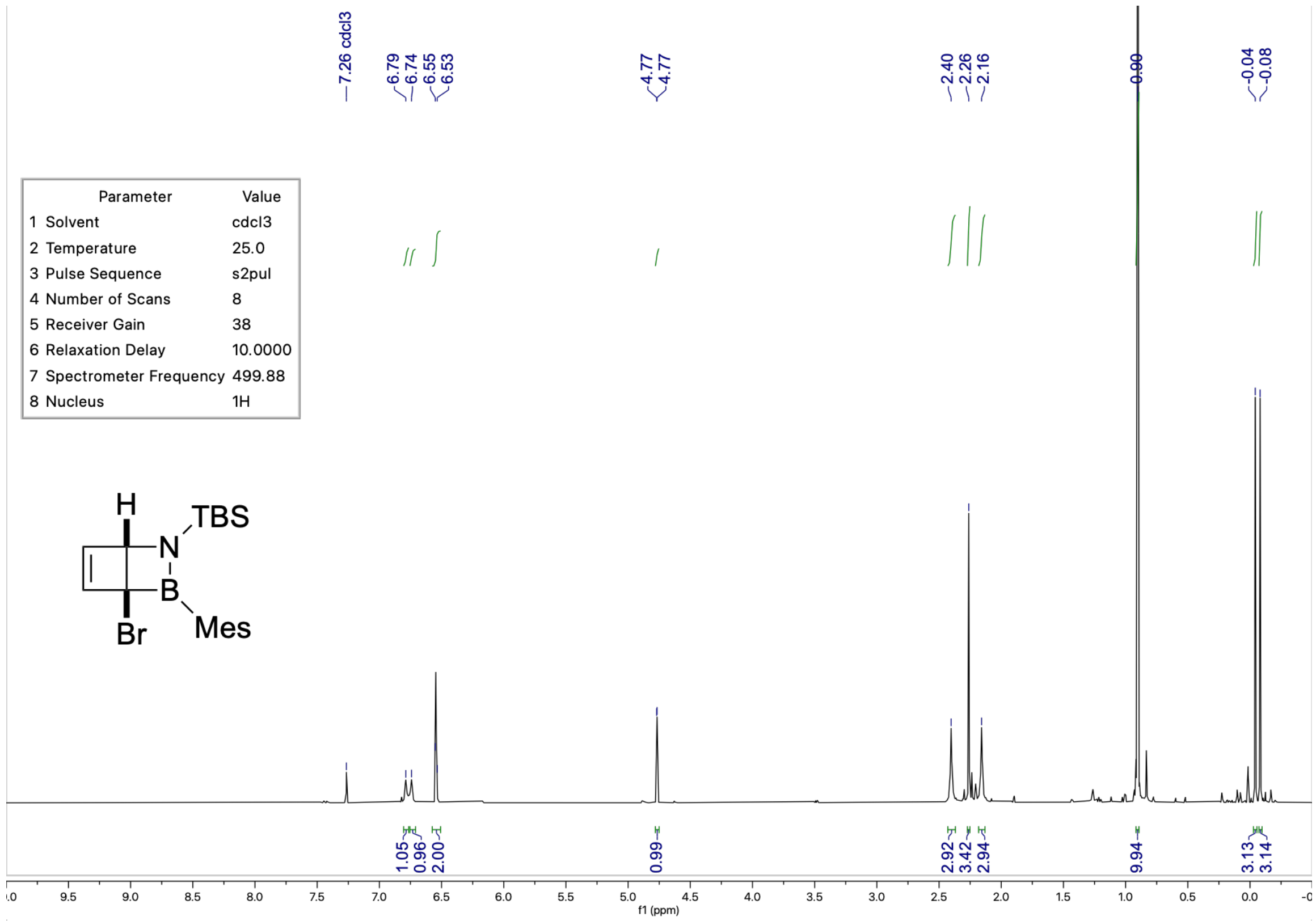

	Parameter	Value
1	Solvent	cdcl3
2	Temperature	25.0
3	Pulse Sequence	s2pul
4	Number of Scans	8
5	Receiver Gain	38
6	Relaxation Delay	10.0000
7	Spectrometer Frequency	499.88
8	Nucleus	1H

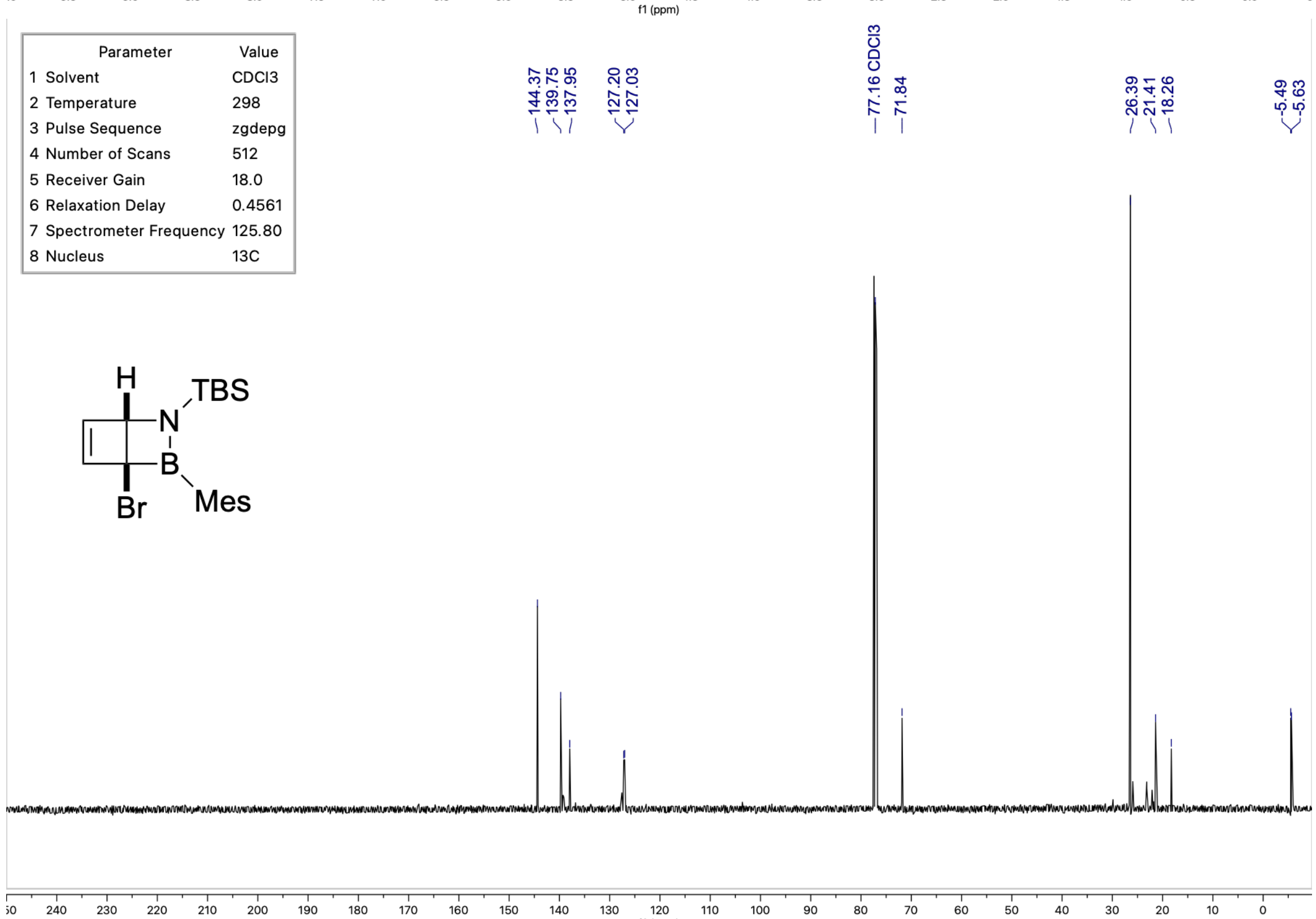

	Parameter	Value
1	Solvent	CDCl3
2	Temperature	298
3	Pulse Sequence	zgdepg
4	Number of Scans	512
5	Receiver Gain	18.0
6	Relaxation Delay	0.4561
7	Spectrometer Frequency	125.80
8	Nucleus	13C

Parameter	Value
1 Solvent	CDCl3
2 Temperature	298
3 Pulse Sequence	zgbs
4 Number of Scans	500
5 Receiver Gain	28.5
6 Relaxation Delay	0.0100
7 Spectrometer Frequency	160.50
8 Nucleus	11B

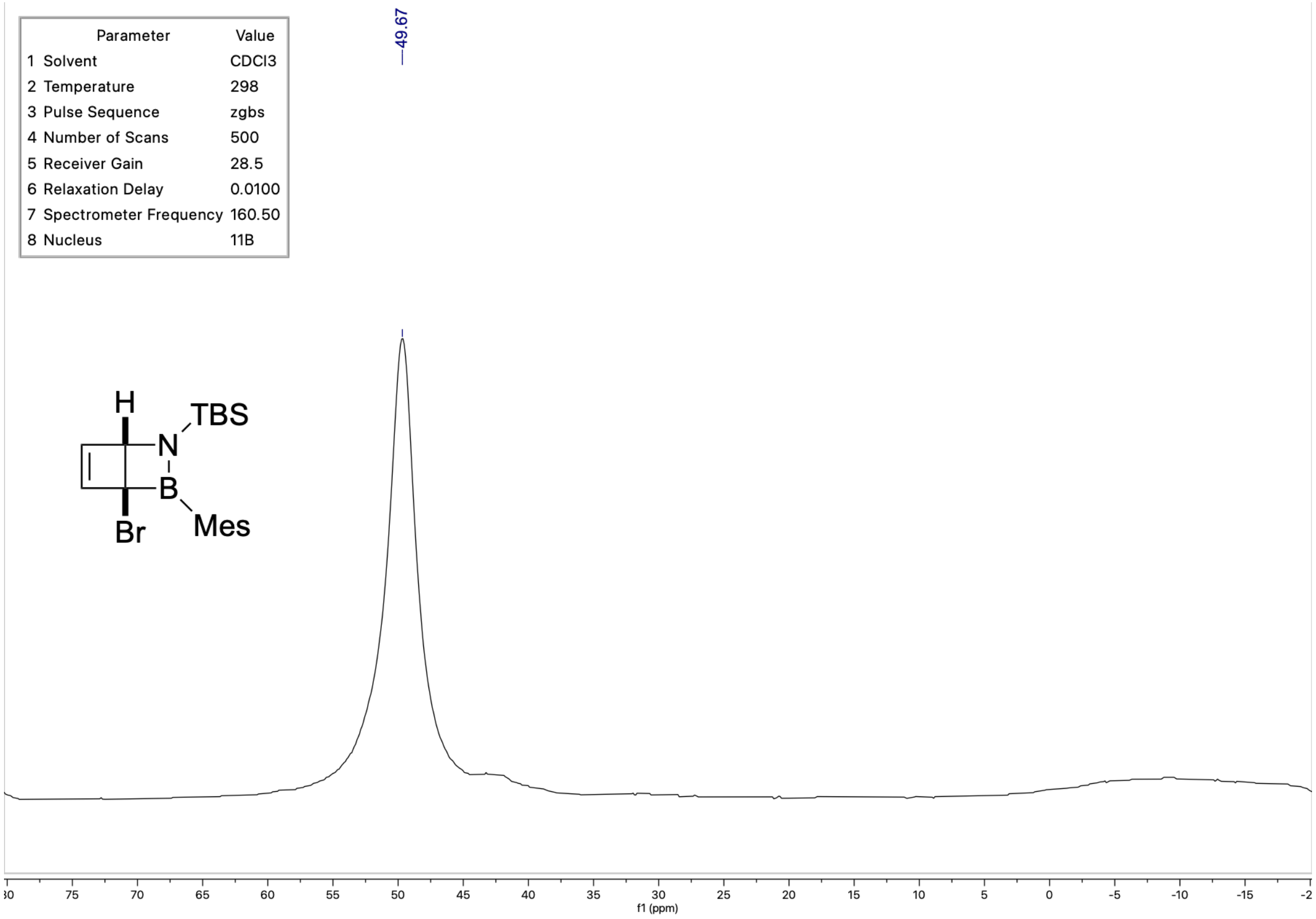

Parameter	Value
1 Solvent	CDCl3
2 Temperature	298
3 Pulse Sequence	zg30
4 Number of Scans	8
5 Receiver Gain	22.6
6 Relaxation Delay	10.0000
7 Spectrometer Frequency	500.23
8 Nucleus	1H

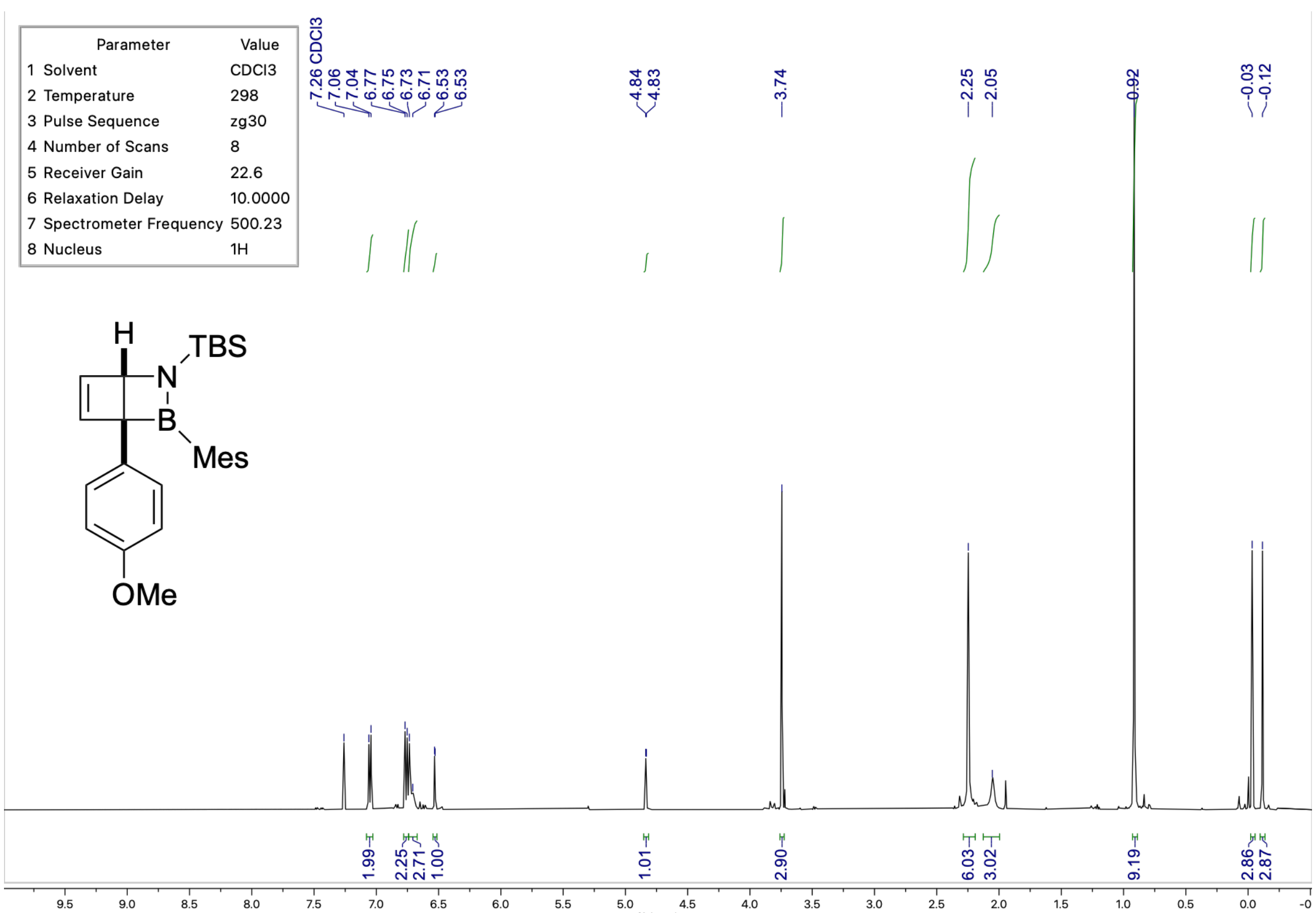

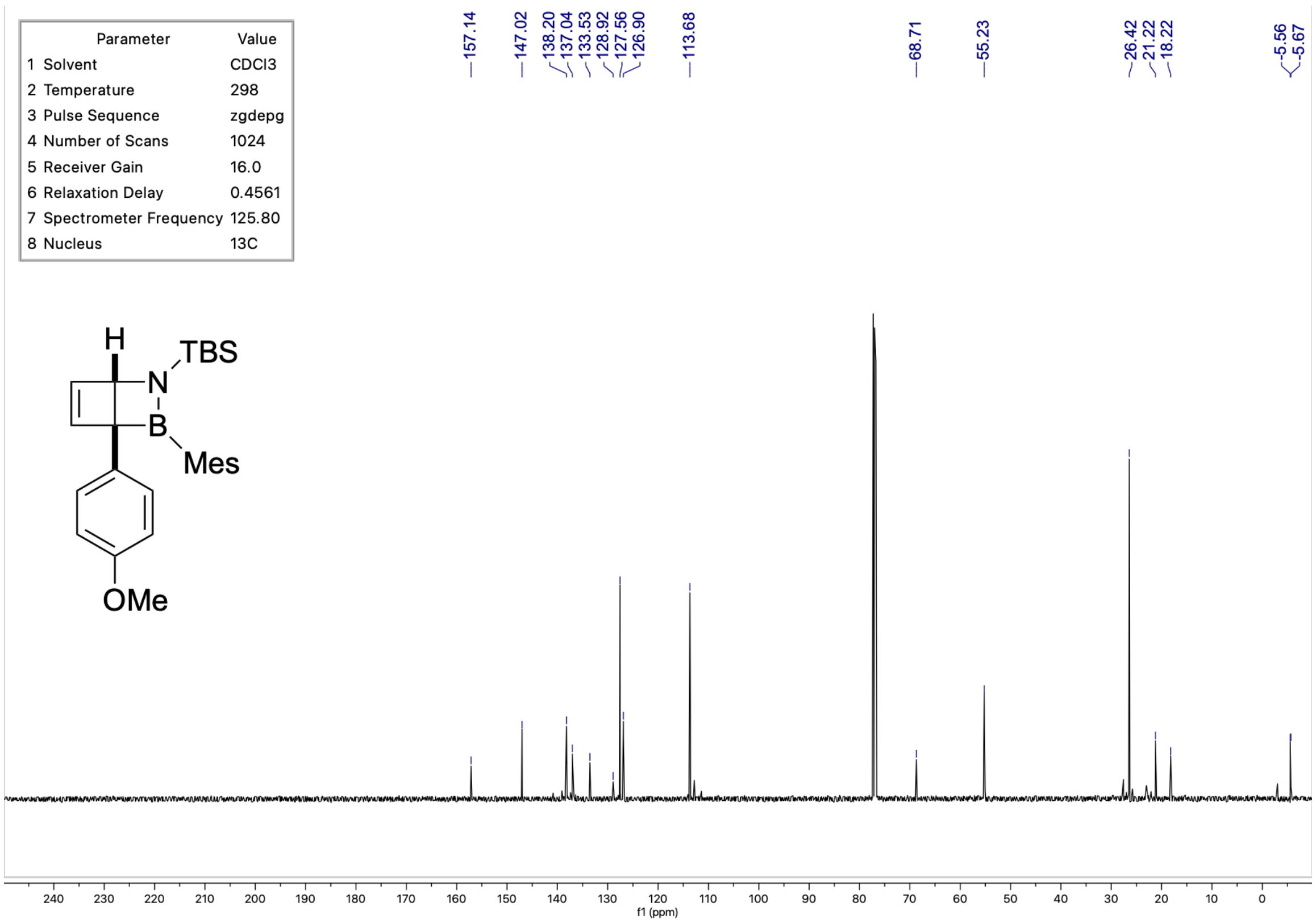
Parameter Value
1 Solvent CDCl3
2 Temperature 298
3 Pulse Sequence zgdepg
4 Number of Scans 1024
5 Receiver Gain 16.0
6 Relaxation Delay 0.4561
7 Spectrometer Frequency 125.80
8 Nucleus 13C
H
TBS
N
B
Mes
OMe
157.14
147.02
138.20
137.04
133.53
128.92
127.56
126.90
113.68
68.71
55.23
26.42
21.22
18.22
-5.56
-5.67
f1 (ppm)

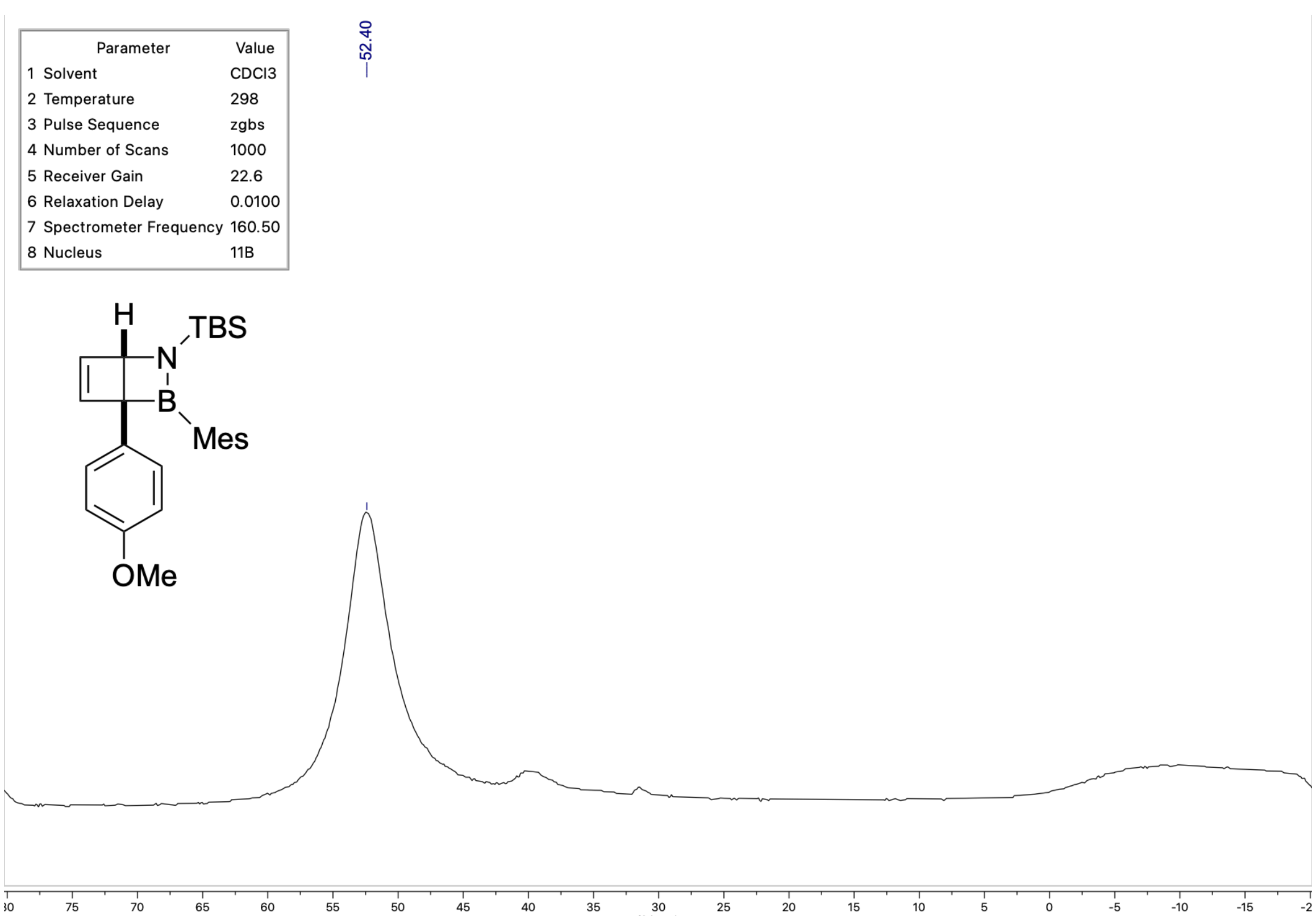
Parameter Value
1 Solvent CDCl3
2 Temperature 298
3 Pulse Sequence zgbs
4 Number of Scans 1000
5 Receiver Gain 22.6
6 Relaxation Delay 0.0100
7 Spectrometer Frequency 160.50
8 Nucleus 11B
H
TBS
N
B
Mes
OMe
52.40
f1 (ppm)

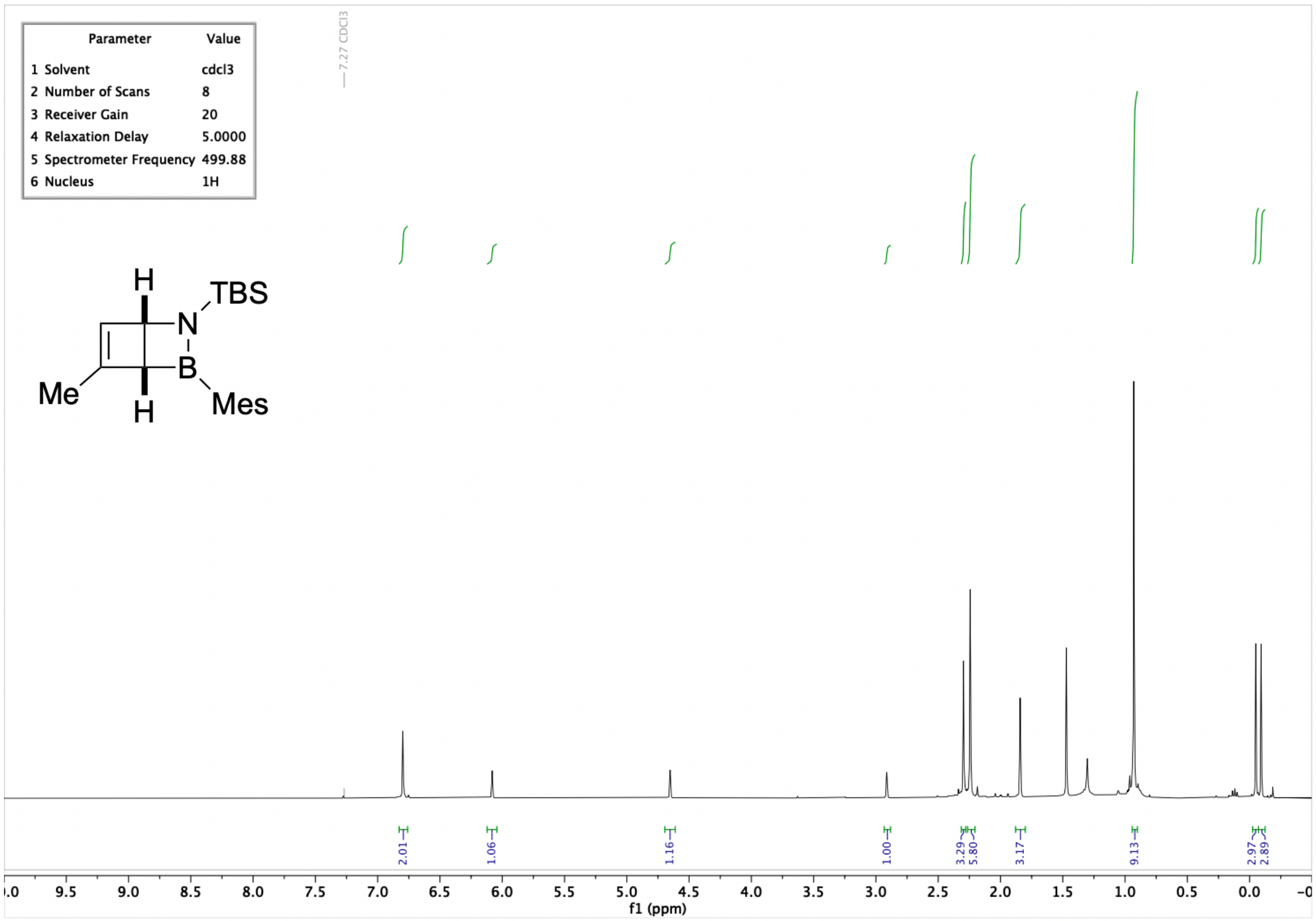

Parameter Value
1 Solvent cdcl3
2 Number of Scans 8
3 Receiver Gain 20
4 Relaxation Delay 5.0000
5 Spectrometer Frequency 499.88
6 Nucleus 1H
H
TBS
N
B
Me
H
Mes
7.27 CDCl3
2.01
1.06
1.16
1.00
3.29
5.80
3.17
9.13
2.97
2.89
f1 (ppm)

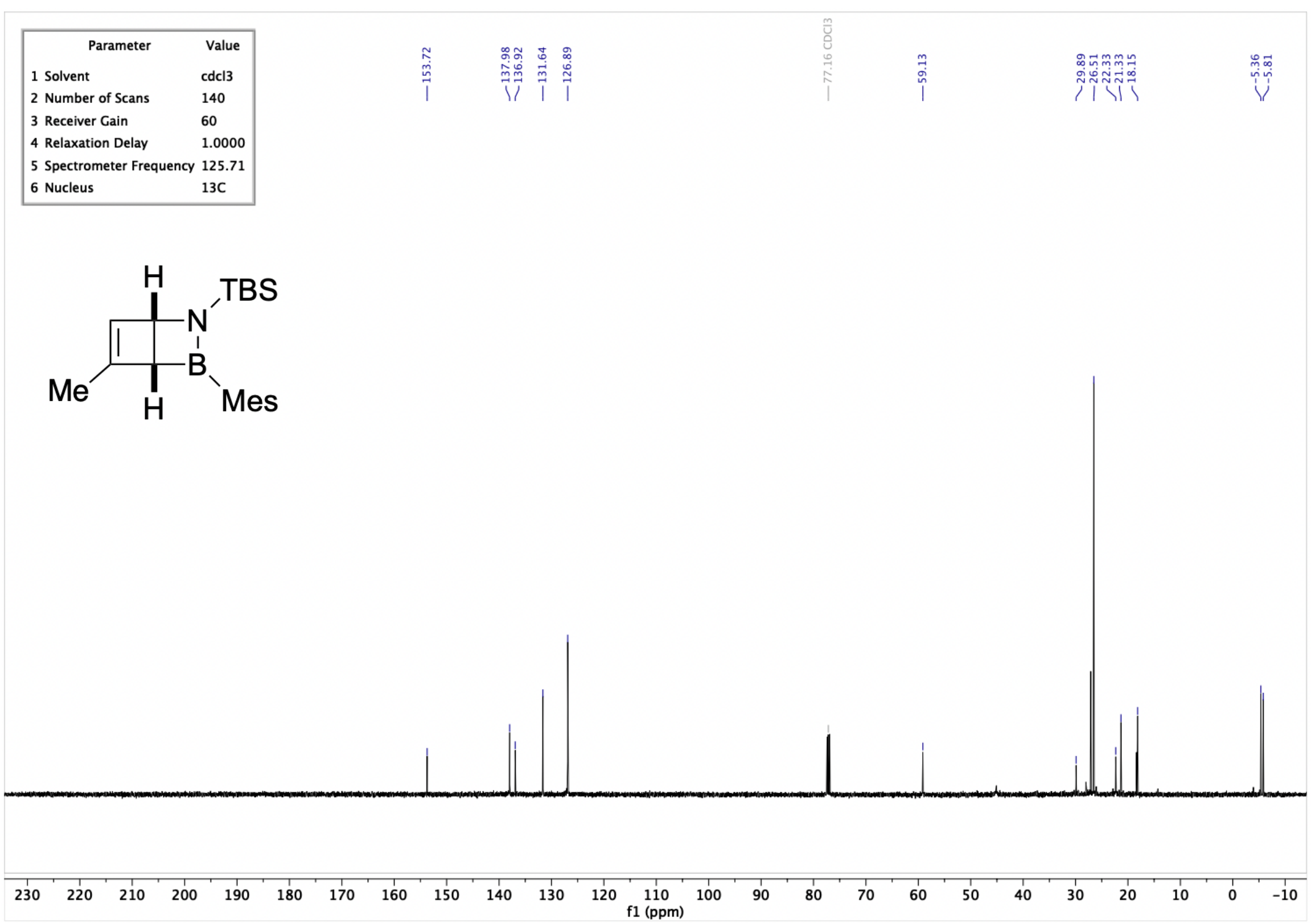

Parameter Value
1 Solvent cdcl3
2 Number of Scans 140
3 Receiver Gain 60
4 Relaxation Delay 1.0000
5 Spectrometer Frequency 125.71
6 Nucleus 13C
H
TBS
N
B
Me
H
Mes
153.72
137.98
136.92
131.64
126.89
77.16 CDCl3
59.13
29.89
26.51
22.33
21.33
18.15
-5.36
-5.81
f1 (ppm)

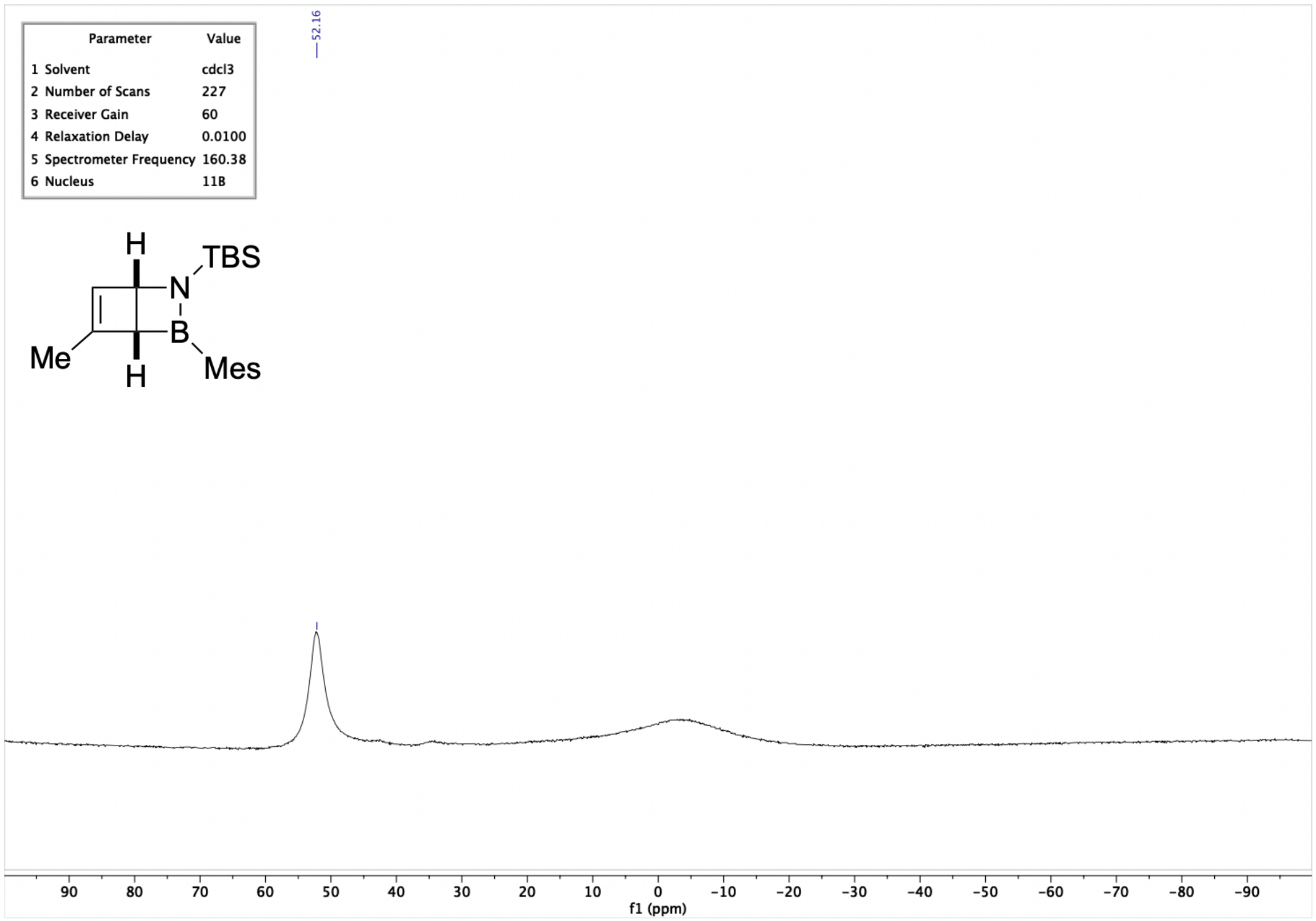
Parameter Value
1 Solvent cdcl3
2 Number of Scans 227
3 Receiver Gain 60
4 Relaxation Delay 0.0100
5 Spectrometer Frequency 160.38
6 Nucleus 11B
H
TBS
N
B
Me
H
Mes
52.16
90 80 70 60 50 40 30 20 10 0 −10 −20 −30 −40 −50 −60 −70 −80 −90
f1 (ppm)

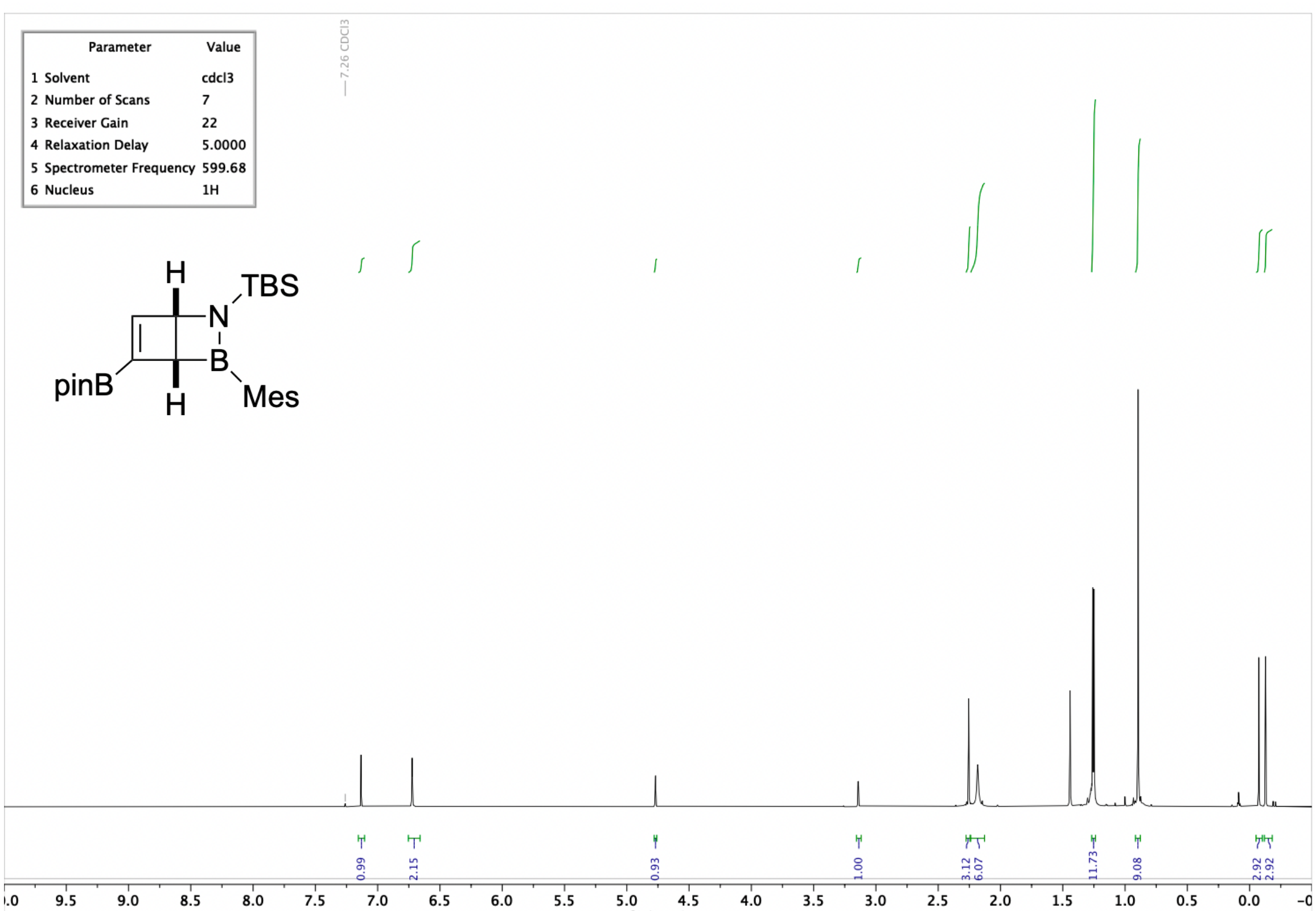
Parameter Value
1 Solvent cdcl3
2 Number of Scans 7
3 Receiver Gain 22
4 Relaxation Delay 5.0000
5 Spectrometer Frequency 599.68
6 Nucleus 1H
H
TBS
N
B
pinB
H
Mes
7.26 CDCl3
0.99
2.15
0.93
1.00
3.12
6.07
11.73
9.08
2.92
2.92
9.5 9.0 8.5 8.0 7.5 7.0 6.5 6.0 5.5 5.0 4.5 4.0 3.5 3.0 2.5 2.0 1.5 1.0 0.5 0.0
f1 (ppm)

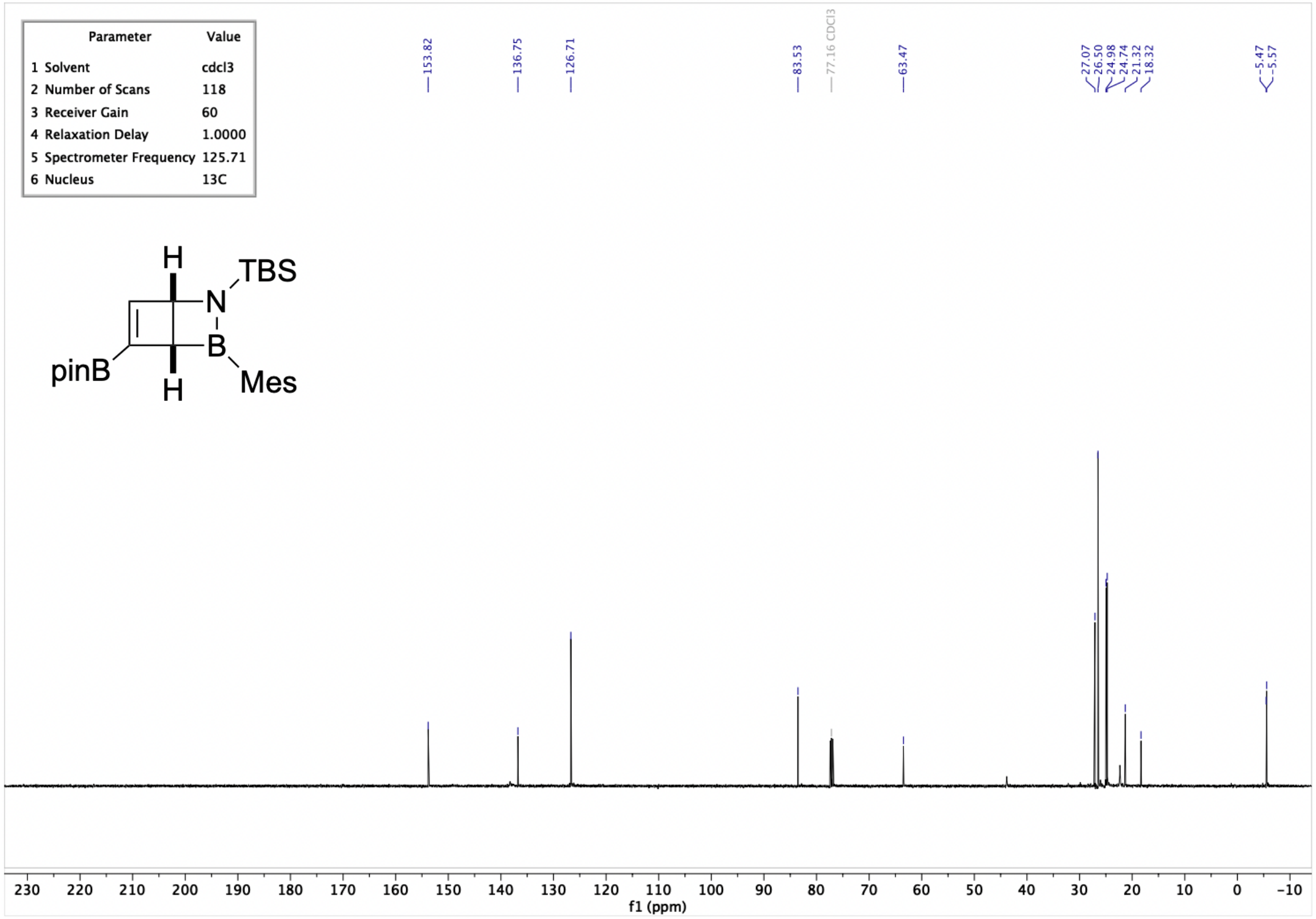

Parameter Value
1 Solvent cdcl3
2 Number of Scans 118
3 Receiver Gain 60
4 Relaxation Delay 1.0000
5 Spectrometer Frequency 125.71
6 Nucleus 13C
TBS
pinB
Mes
f1 (ppm)

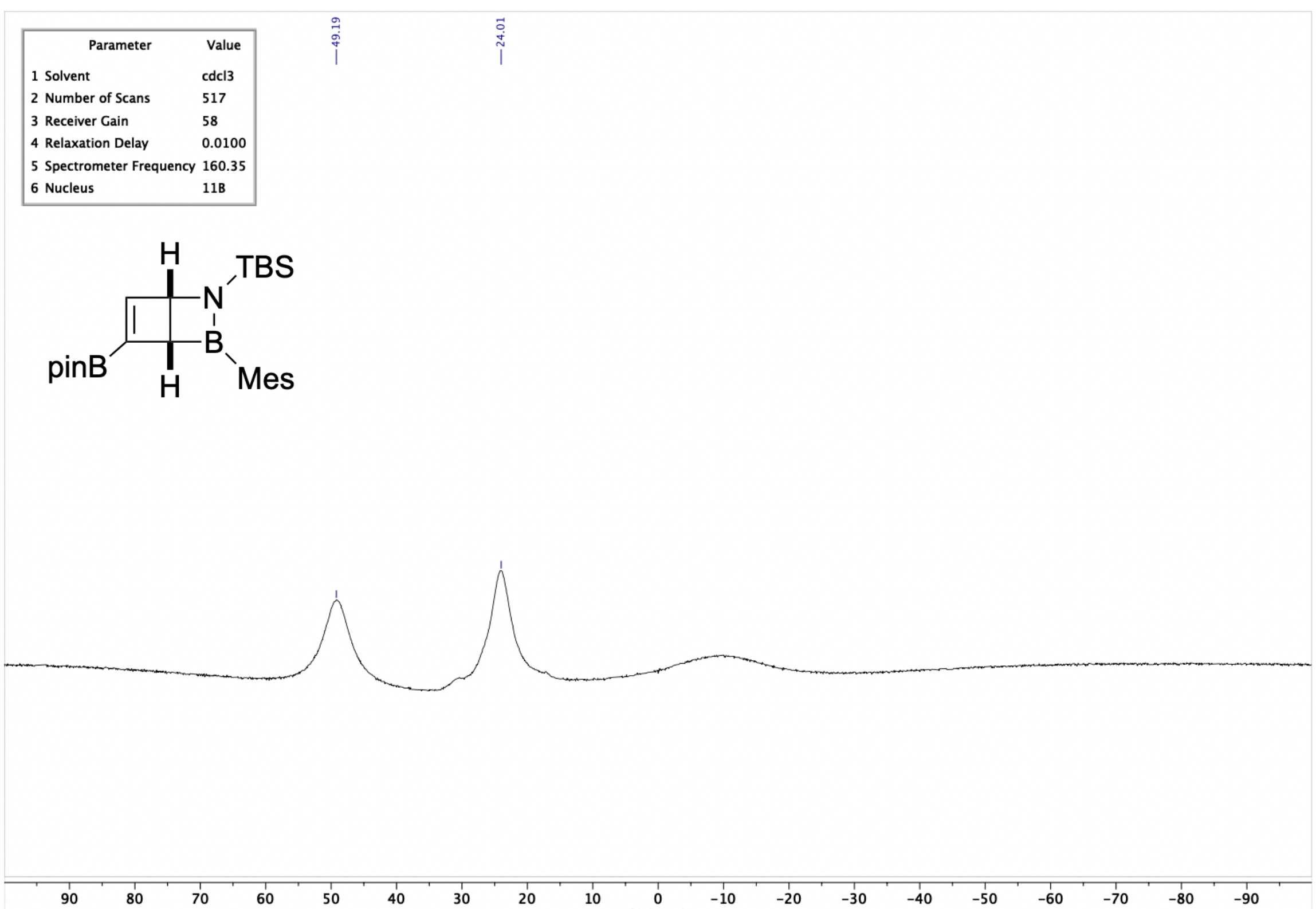

Parameter Value
1 Solvent cdcl3
2 Number of Scans 517
3 Receiver Gain 58
4 Relaxation Delay 0.0100
5 Spectrometer Frequency 160.35
6 Nucleus 11B
TBS
pinB
Mes
49.19
24.01
f1 (ppm)

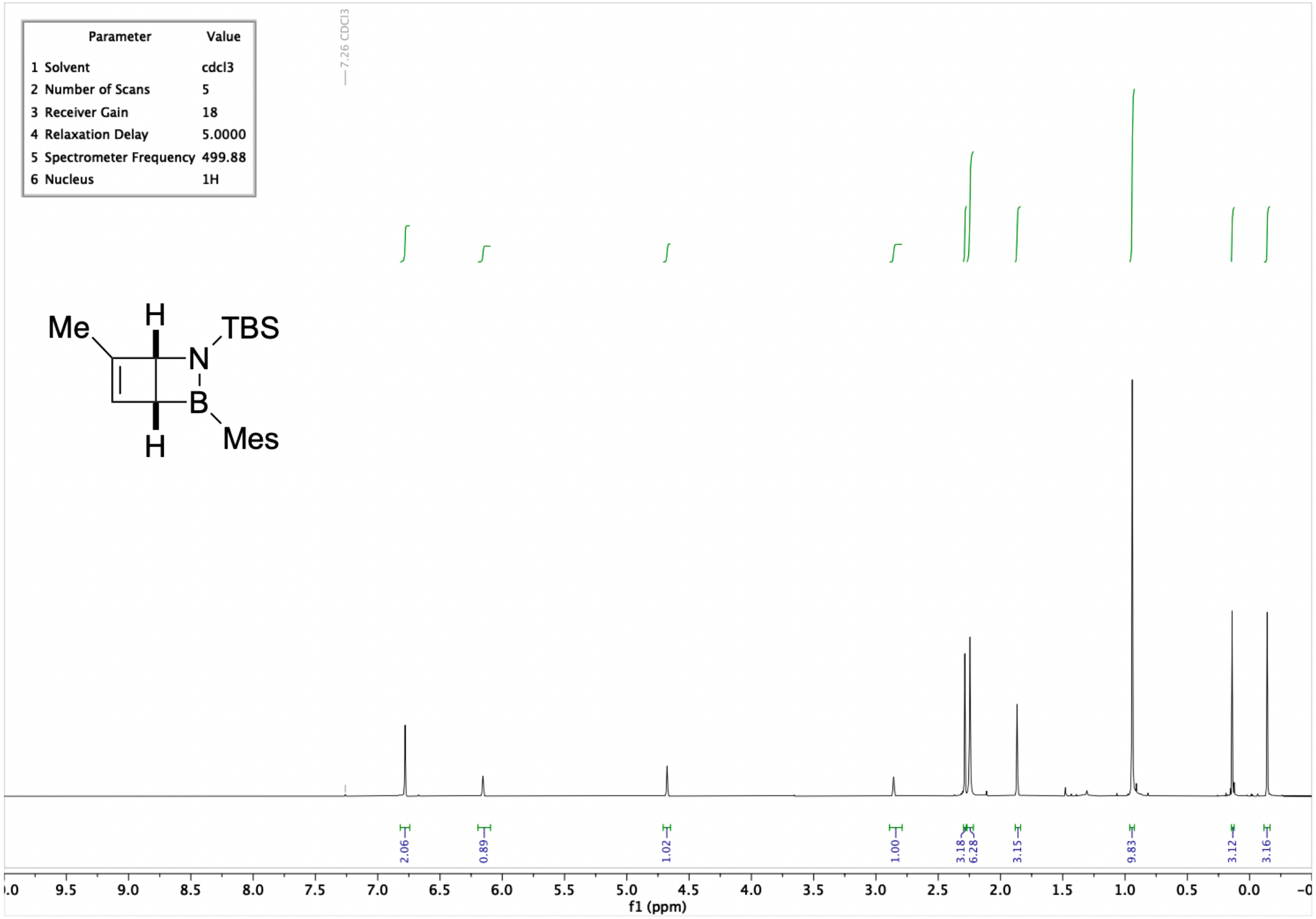

Parameter Value
1 Solvent cdcl3
2 Number of Scans 5
3 Receiver Gain 18
4 Relaxation Delay 5.0000
5 Spectrometer Frequency 499.88
6 Nucleus 1H
7.26 CDCl3
Me
H
TBS
N
B
H
Mes
2.06
0.89
1.02
1.00
3.18
6.28
3.15
9.83
3.12
3.16
f1 (ppm)

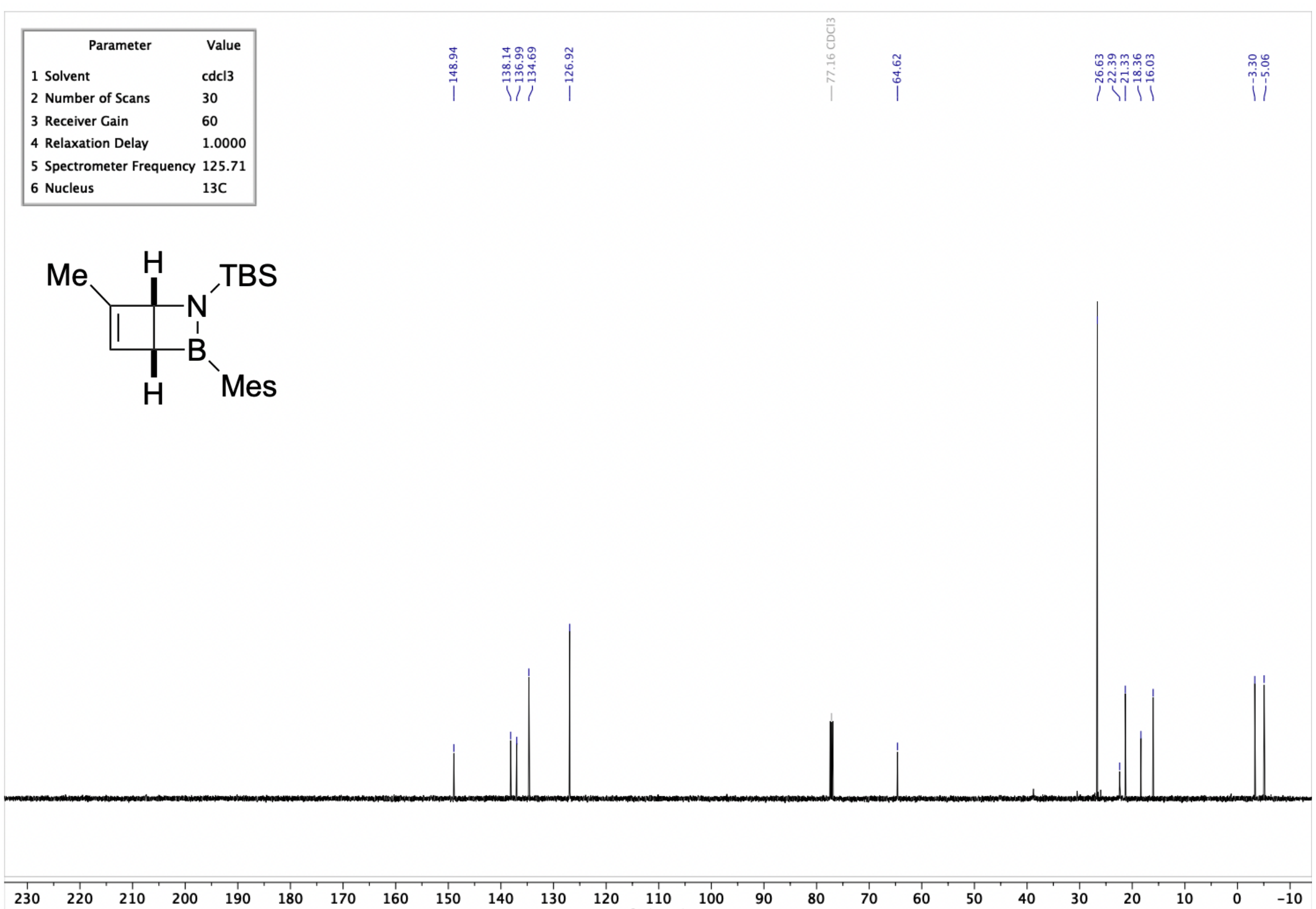

Parameter Value
1 Solvent cdcl3
2 Number of Scans 30
3 Receiver Gain 60
4 Relaxation Delay 1.0000
5 Spectrometer Frequency 125.71
6 Nucleus 13C
148.94
138.14
136.99
134.69
126.92
77.16 CDCl3
64.62
26.63
22.39
21.33
18.36
16.03
-3.30
-5.06
Me
H
TBS
N
B
H
Mes
f1 (ppm)

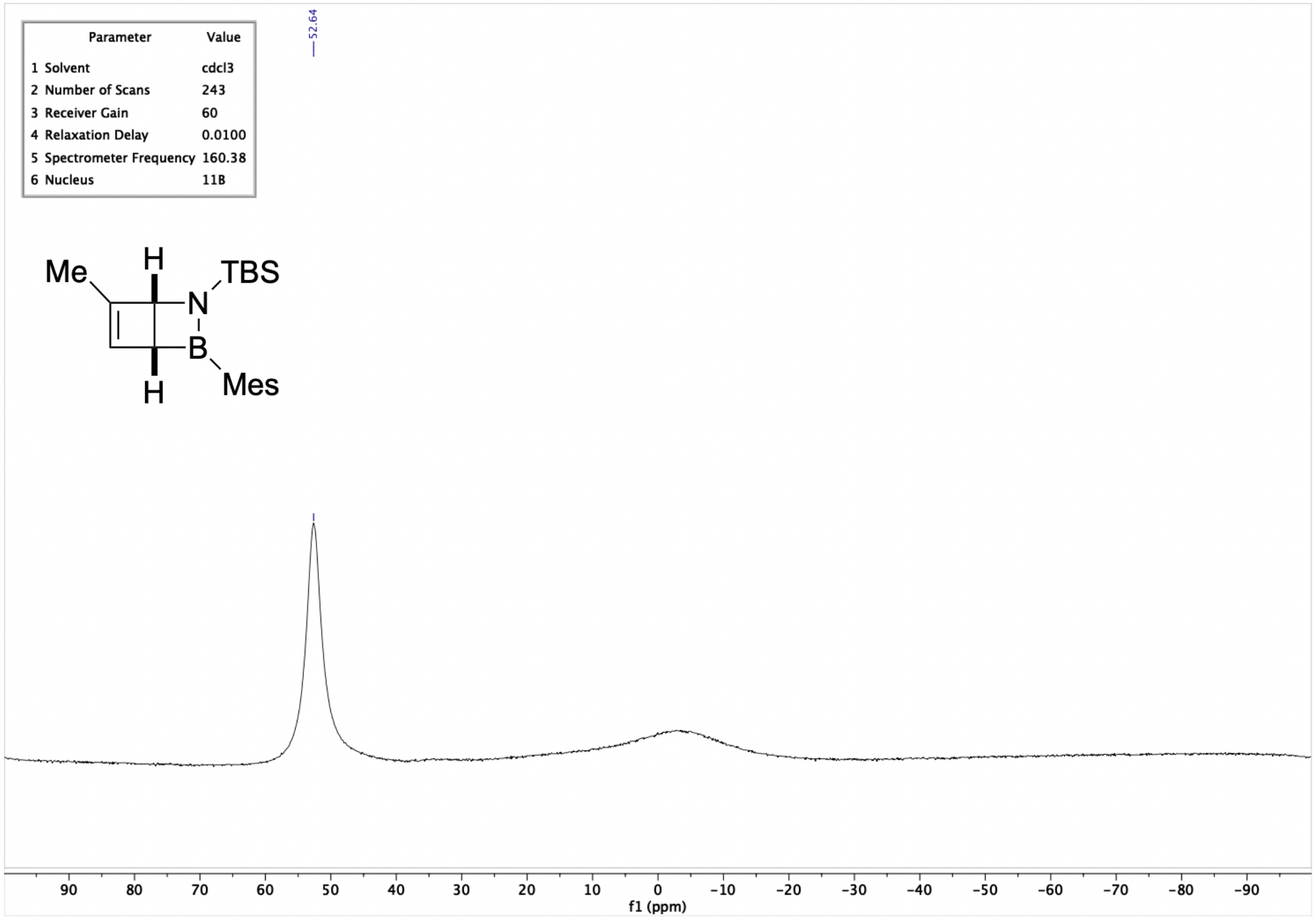
Parameter Value
1 Solvent cdcl3
2 Number of Scans 243
3 Receiver Gain 60
4 Relaxation Delay 0.0100
5 Spectrometer Frequency 160.38
6 Nucleus 11B
52.64
Me
H
TBS
N
B
H
Mes
f1 (ppm)

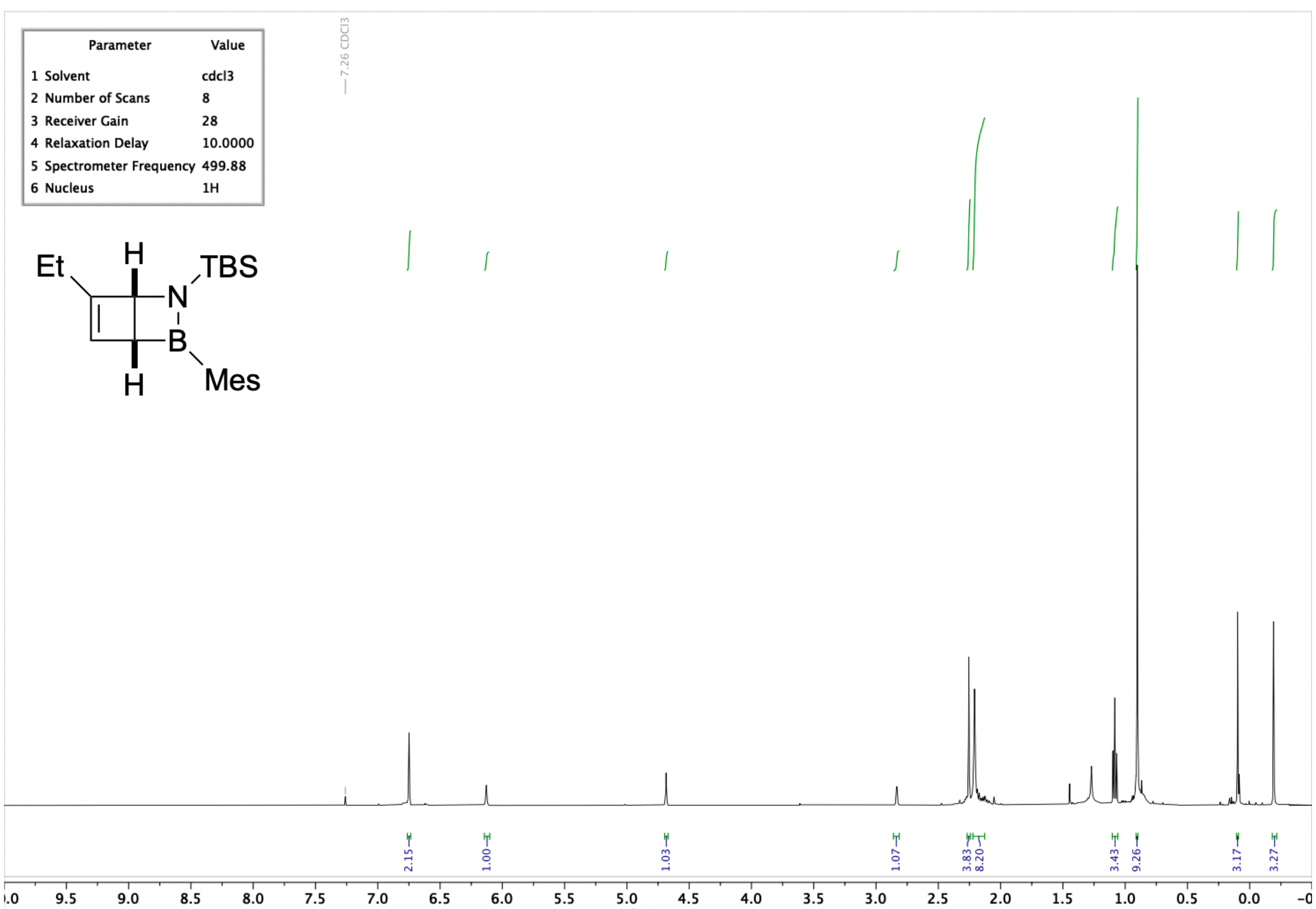
Parameter Value
1 Solvent cdcl3
2 Number of Scans 8
3 Receiver Gain 28
4 Relaxation Delay 10.0000
5 Spectrometer Frequency 499.88
6 Nucleus 1H
7.26 CDCl3
Et
H
TBS
N
B
H
Mes
2.15
1.00
1.03
1.07
3.83
8.20
3.43
9.26
3.17
3.27
f1 (ppm)

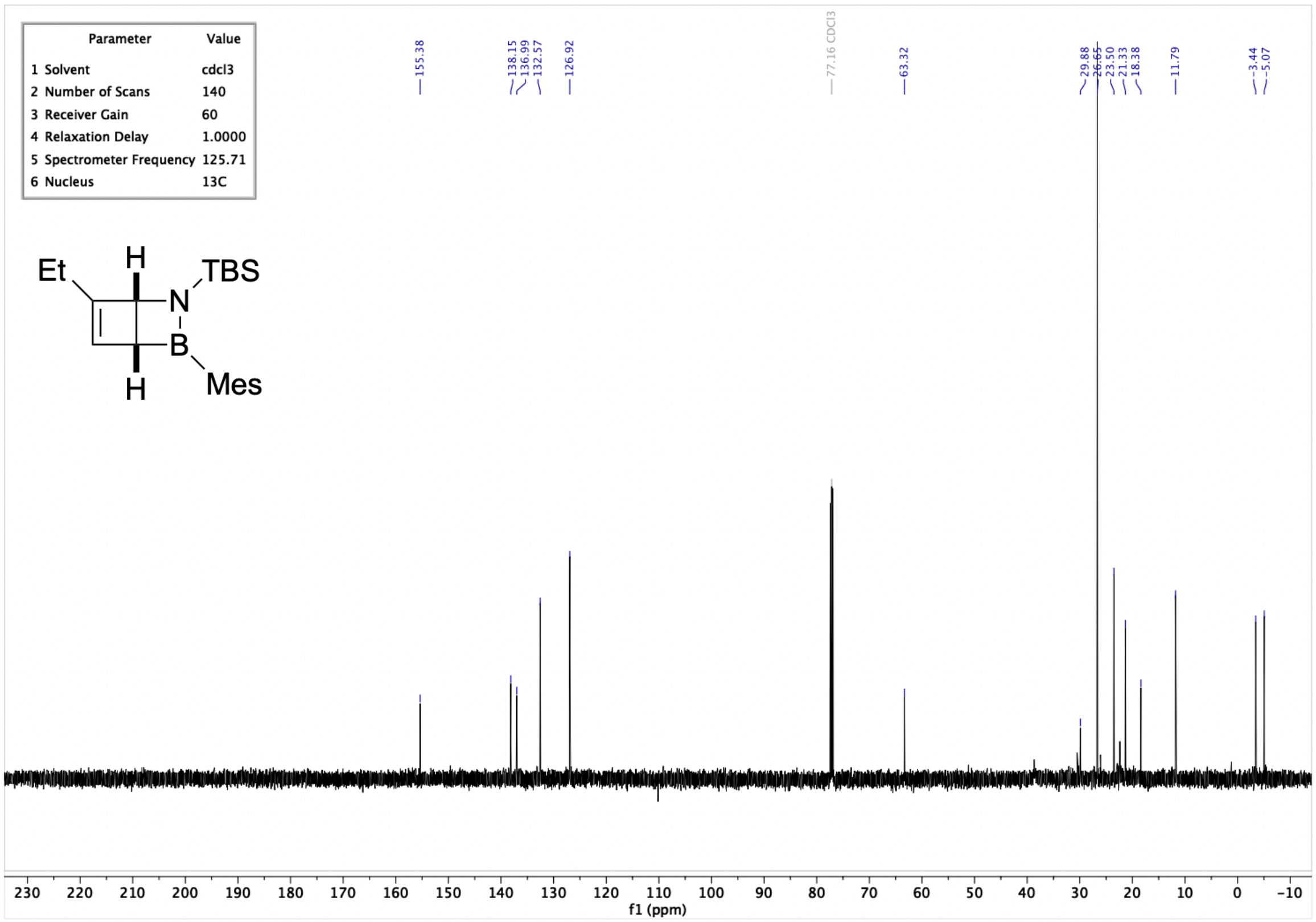

Parameter Value
1 Solvent cdcl3
2 Number of Scans 140
3 Receiver Gain 60
4 Relaxation Delay 1.0000
5 Spectrometer Frequency 125.71
6 Nucleus 13C
Et
H
TBS
N
B
H
Mes
155.38
138.15
136.99
132.57
126.92
77.16 CDCl3
63.32
29.88
26.65
23.50
21.33
18.38
11.79
-3.44
-5.07
f1 (ppm)

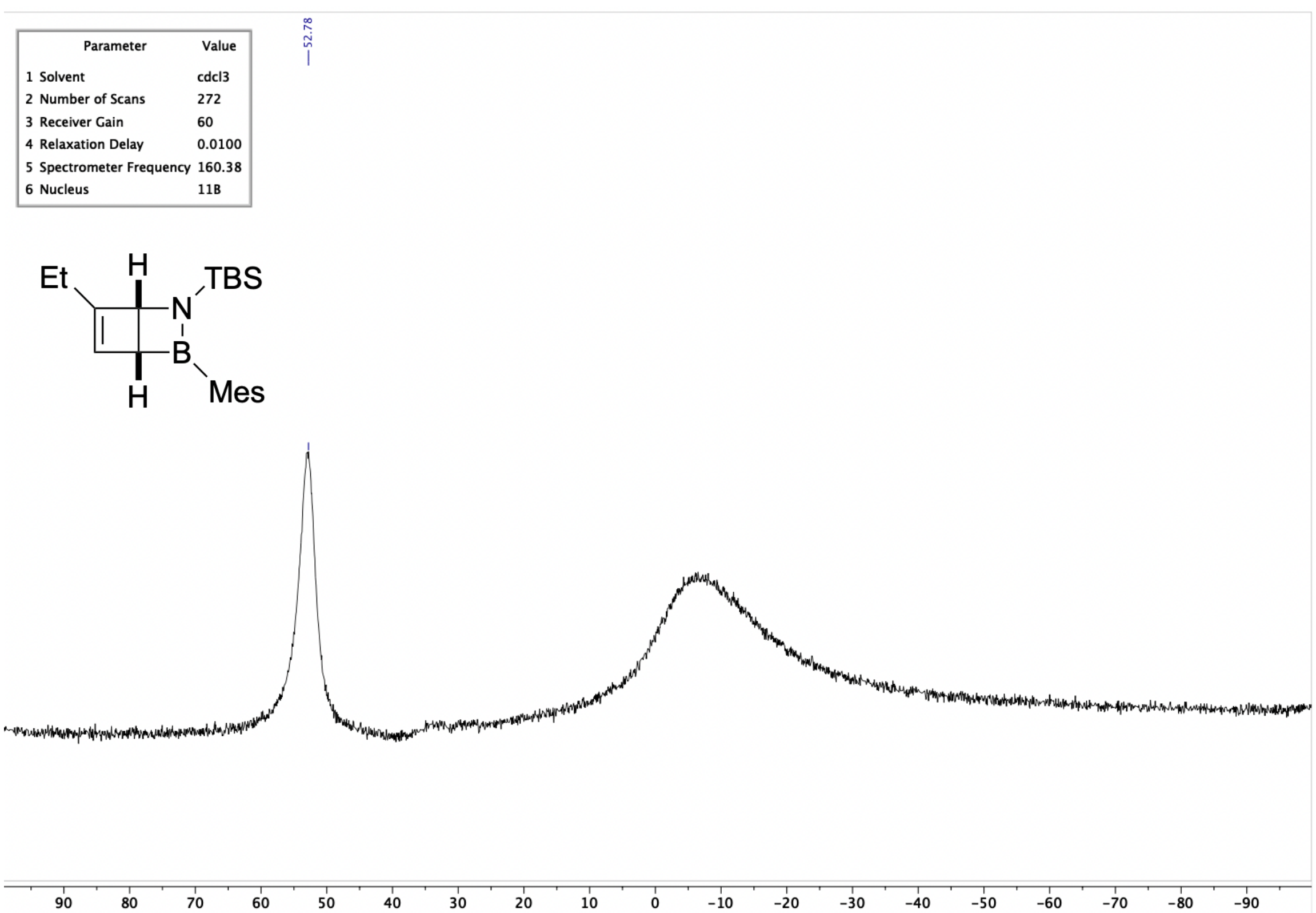

Parameter Value
1 Solvent cdcl3
2 Number of Scans 272
3 Receiver Gain 60
4 Relaxation Delay 0.0100
5 Spectrometer Frequency 160.38
6 Nucleus 11B
Et
H
TBS
N
B
H
Mes
52.78
f1 (ppm)

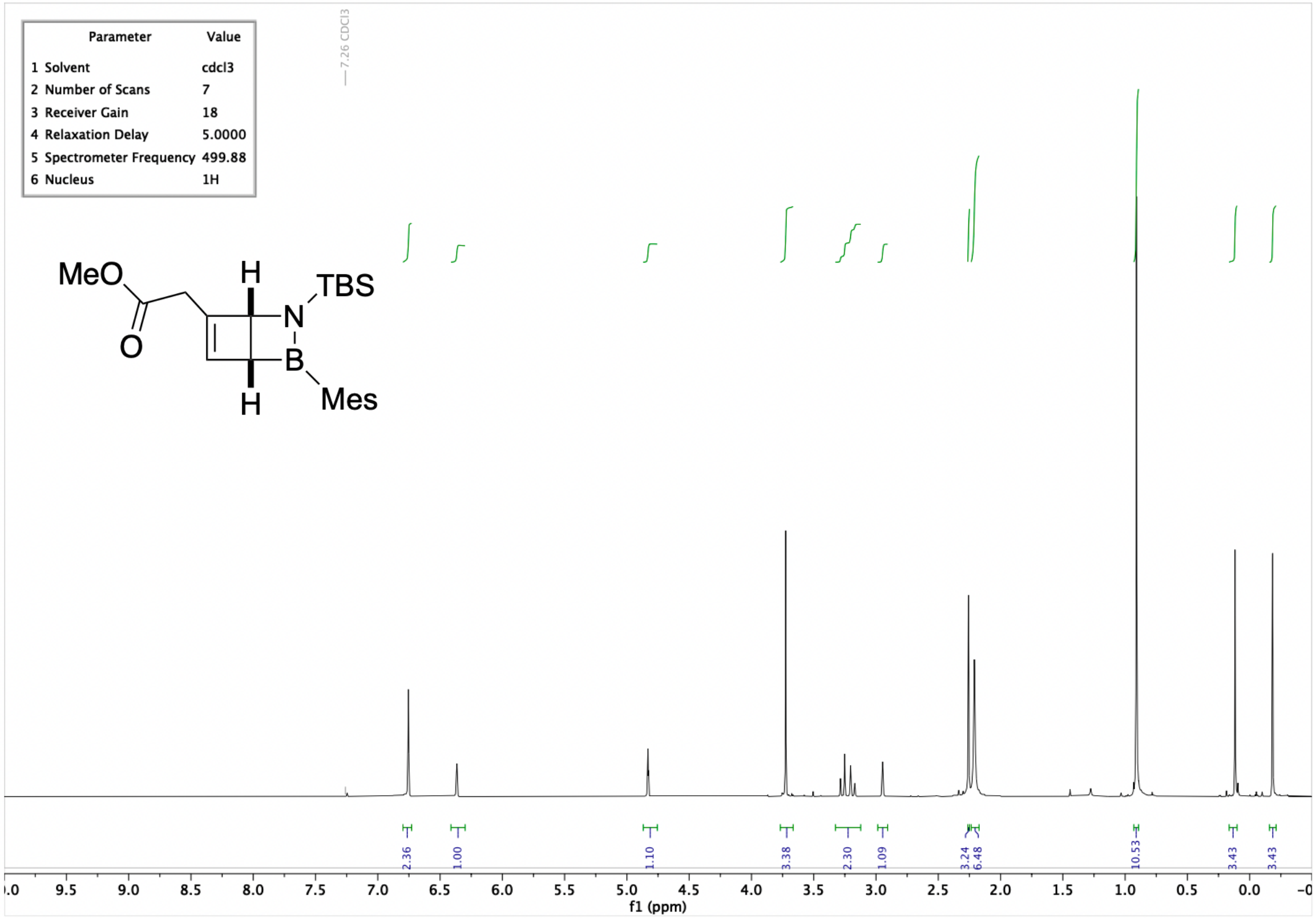

Parameter Value
1 Solvent cdcl3
2 Number of Scans 7
3 Receiver Gain 18
4 Relaxation Delay 5.0000
5 Spectrometer Frequency 499.88
6 Nucleus 1H
7.26 CDCl3
MeO
O
H
H
N
TBS
B
Mes
2.36
1.00
1.10
3.38
2.30
1.09
3.24
6.48
10.53
3.43
3.43
f1 (ppm)

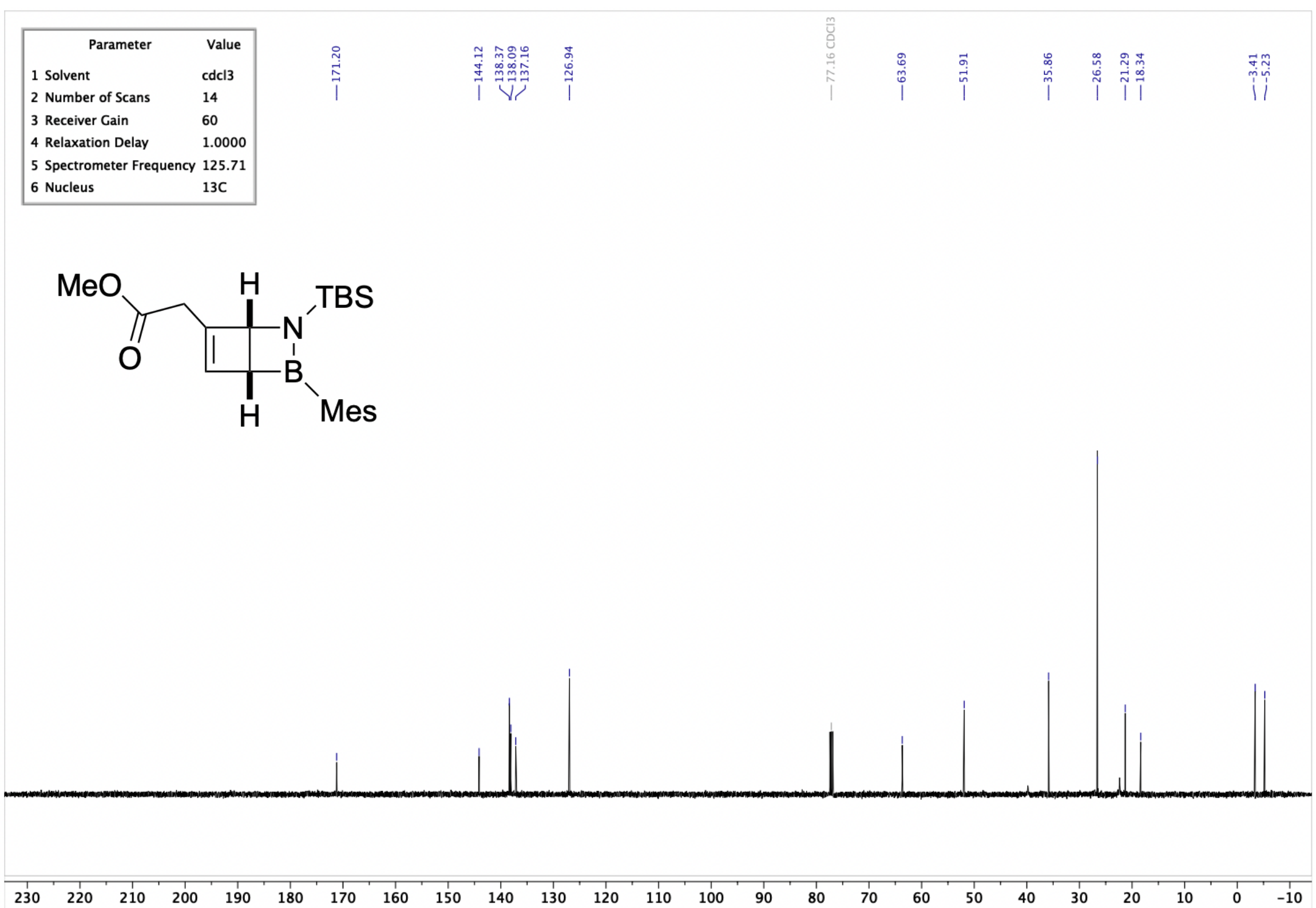

Parameter Value
1 Solvent cdcl3
2 Number of Scans 14
3 Receiver Gain 60
4 Relaxation Delay 1.0000
5 Spectrometer Frequency 125.71
6 Nucleus 13C
171.20
144.12
138.37
138.09
137.16
126.94
77.16 CDCl3
63.69
51.91
35.86
26.58
21.29
18.34
-3.41
-5.23
MeO
O
H
H
N
TBS
B
Mes
f1 (ppm)

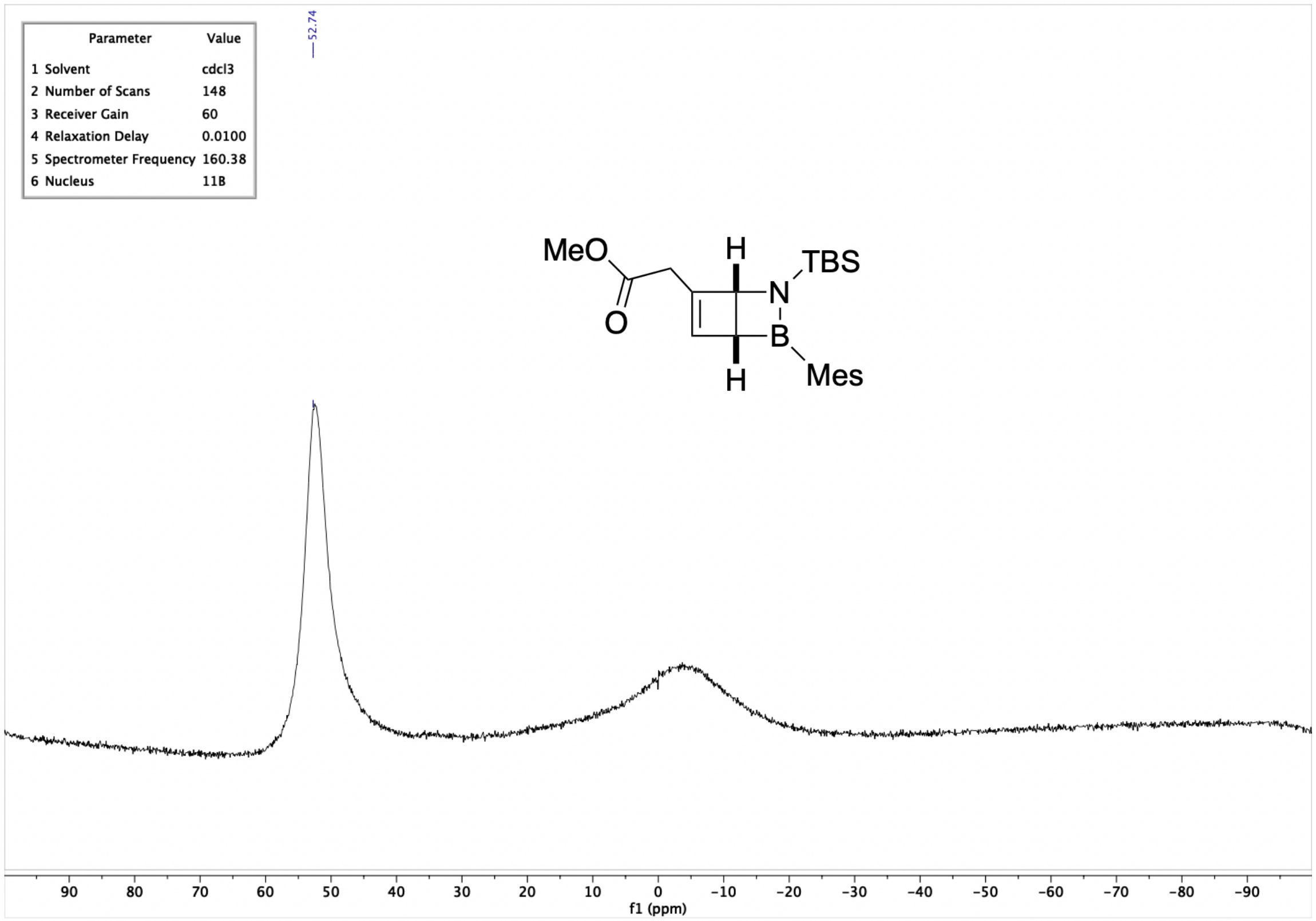

Parameter Value
1 Solvent cdcl3
2 Number of Scans 148
3 Receiver Gain 60
4 Relaxation Delay 0.0100
5 Spectrometer Frequency 160.38
6 Nucleus 11B
52.74
MeO
O
H
H
TBS
N
B
Mes
f1 (ppm)

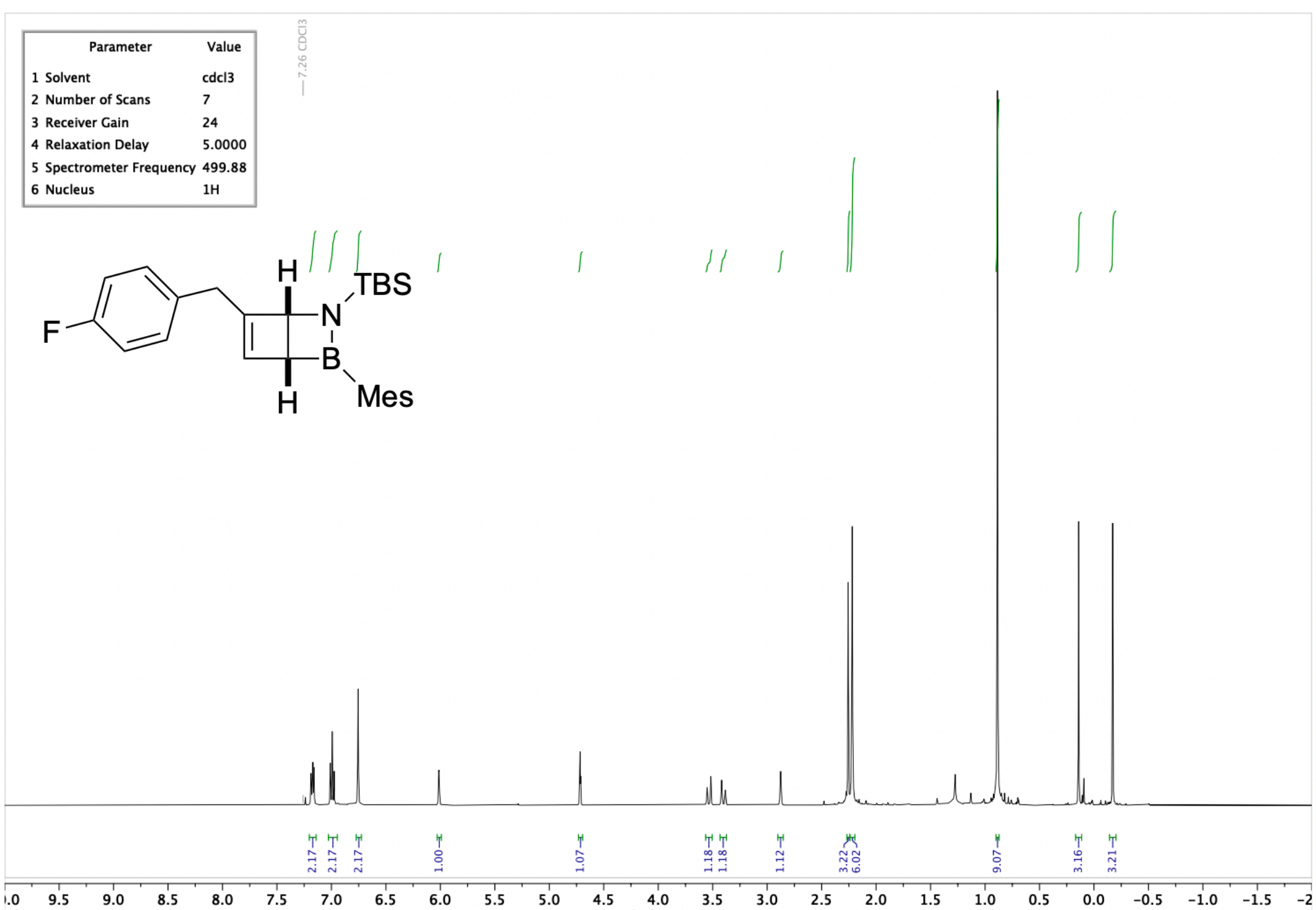

Parameter Value
1 Solvent cdcl3
2 Number of Scans 7
3 Receiver Gain 24
4 Relaxation Delay 5.0000
5 Spectrometer Frequency 499.88
6 Nucleus 1H
7.26 CDCl3
F
H
H
TBS
N
B
Mes
2.17
2.17
2.17
1.00
1.07
1.18
1.18
1.12
3.22
6.02
9.07
3.16
3.21
f1 (ppm)

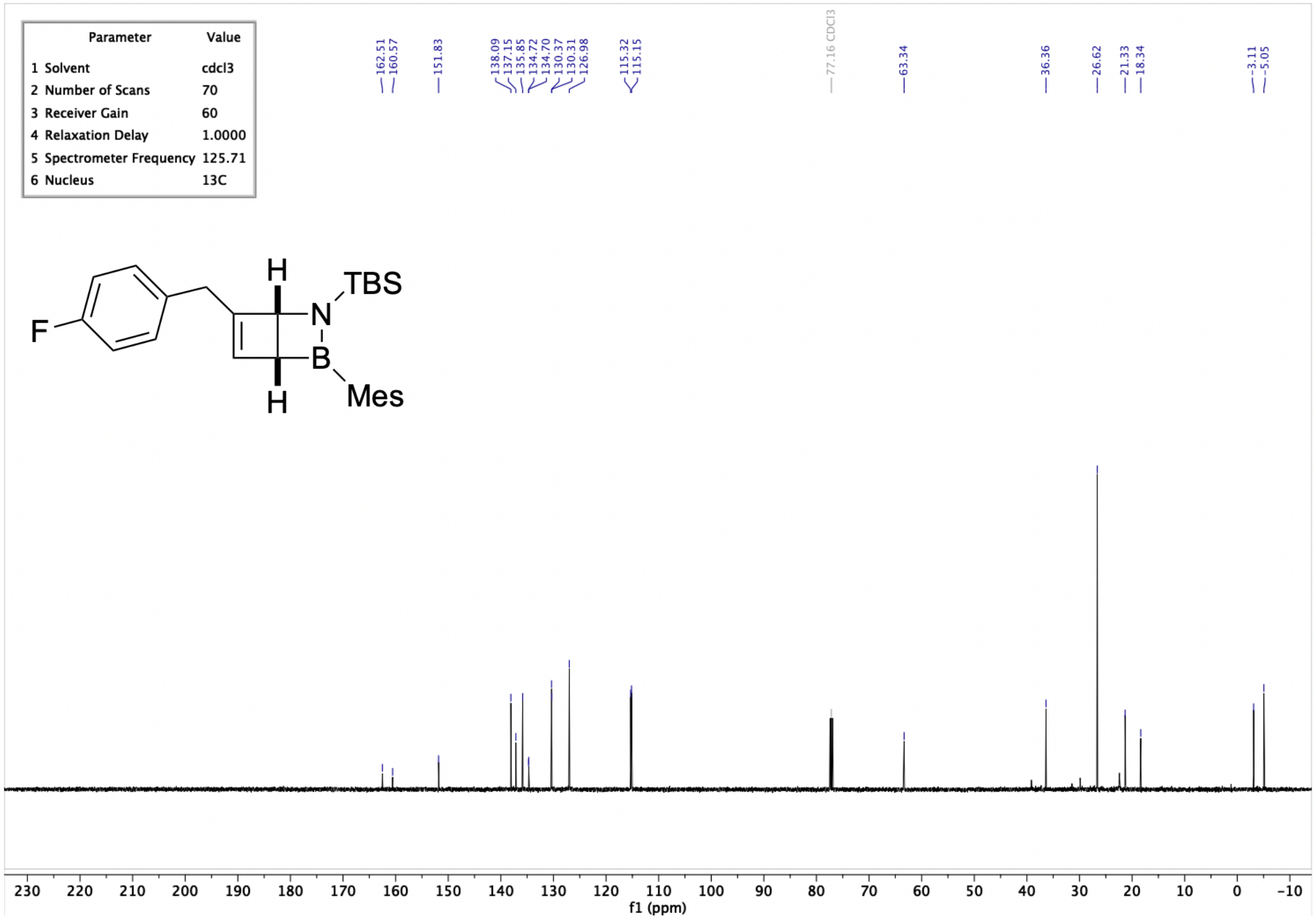
Parameter Value
1 Solvent cdcl3
2 Number of Scans 70
3 Receiver Gain 60
4 Relaxation Delay 1.0000
5 Spectrometer Frequency 125.71
6 Nucleus 13C
162.51
160.57
151.83
138.09
137.15
135.85
134.72
134.70
130.37
130.31
126.98
115.32
115.15
77.16 CDCl3
63.34
36.36
26.62
21.33
18.34
-3.11
-5.05
F
H
TBS
N
B
H
Mes
f1 (ppm)

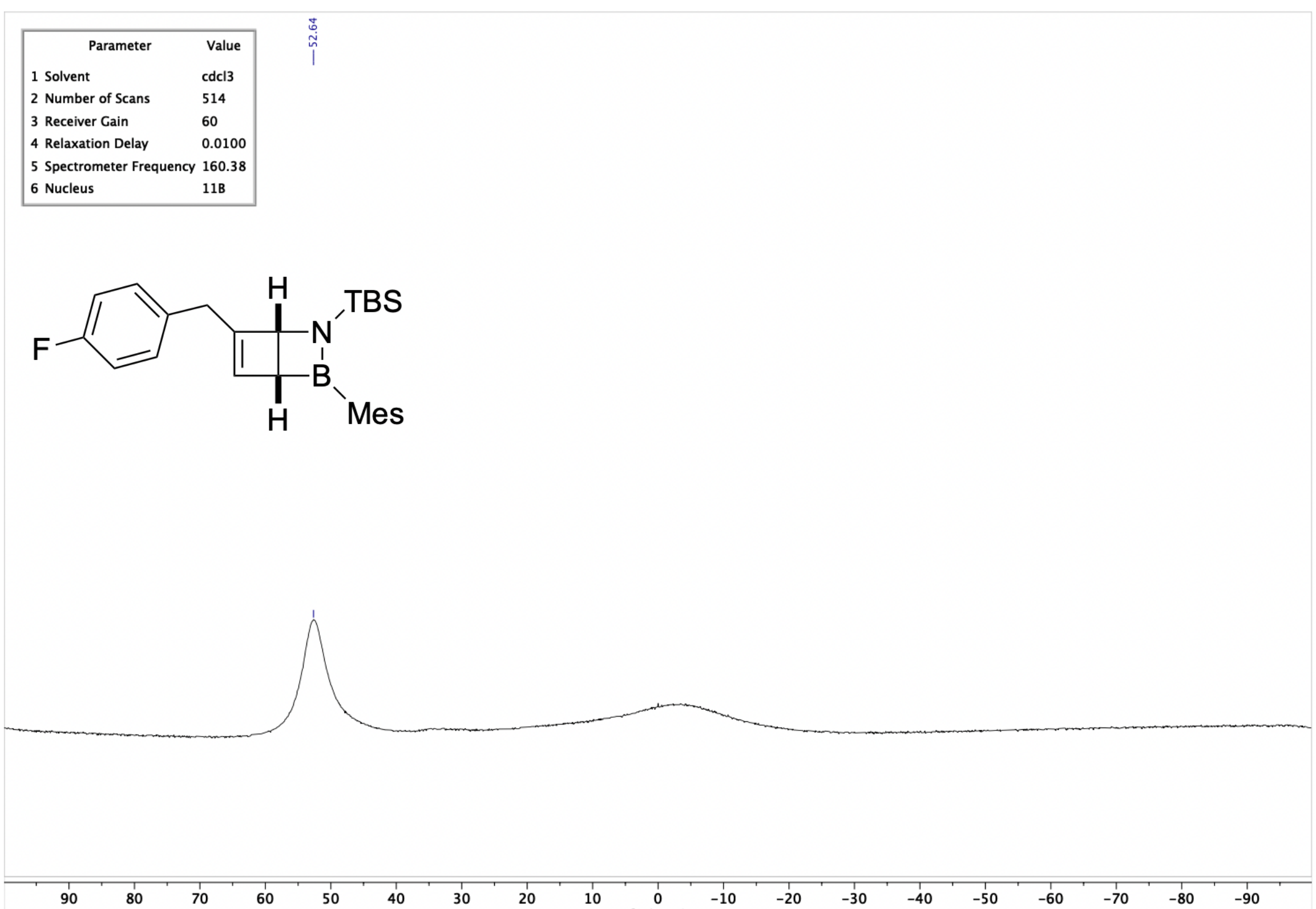
Parameter Value
1 Solvent cdcl3
2 Number of Scans 514
3 Receiver Gain 60
4 Relaxation Delay 0.0100
5 Spectrometer Frequency 160.38
6 Nucleus 11B
52.64
F
H
TBS
N
B
H
Mes
f1 (ppm)

Parameter	Value
1 Solvent	cdcl3
2 Number of Scans	100
3 Receiver Gain	58
4 Relaxation Delay	1.0000
5 Spectrometer Frequency	470.31
6 Nucleus	19F

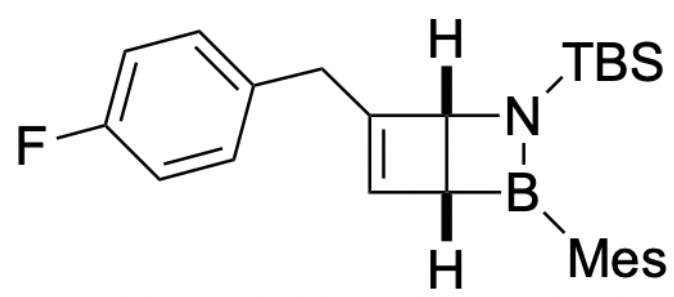

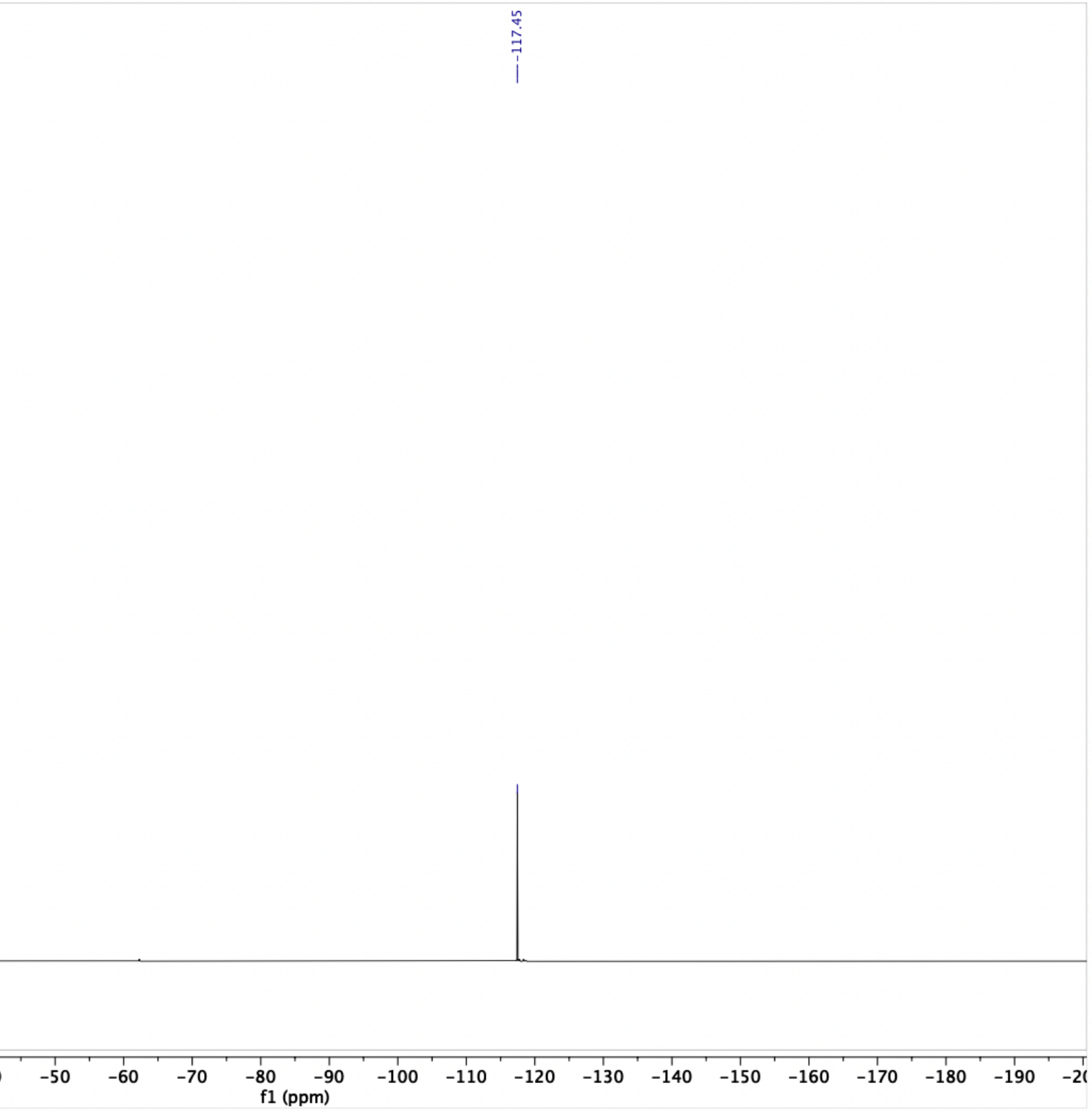

Parameter	Value
1 Solvent	cdcl3
2 Temperature	25.0
3 Pulse Sequence	s2pul
4 Number of Scans	8
5 Receiver Gain	20
6 Relaxation Delay	5.0000
7 Spectrometer Frequency	499.88
8 Nucleus	1H

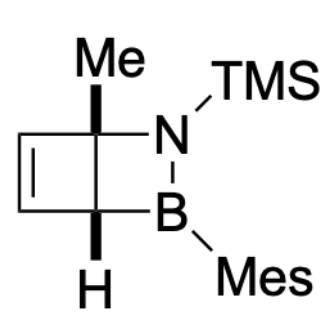

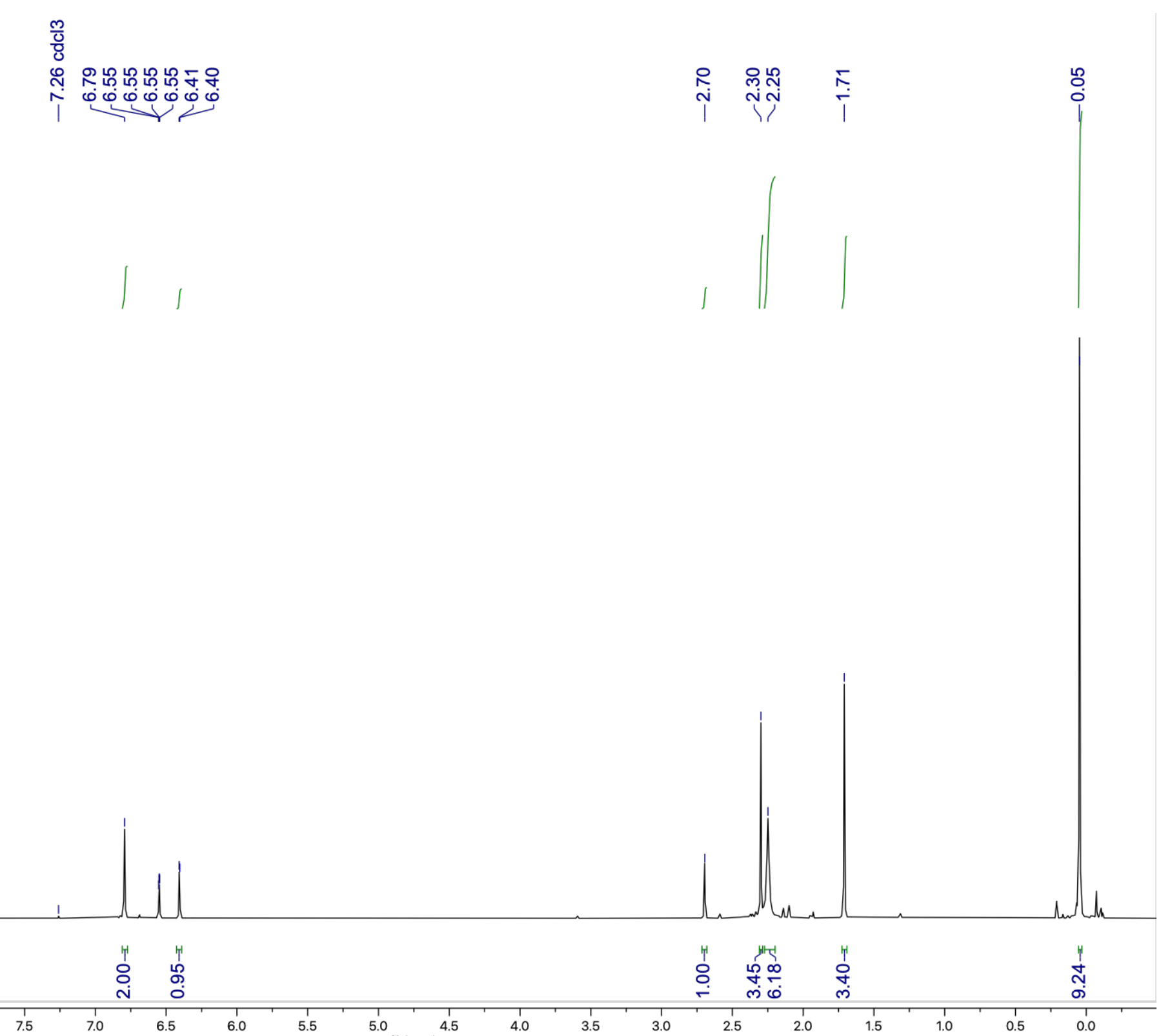

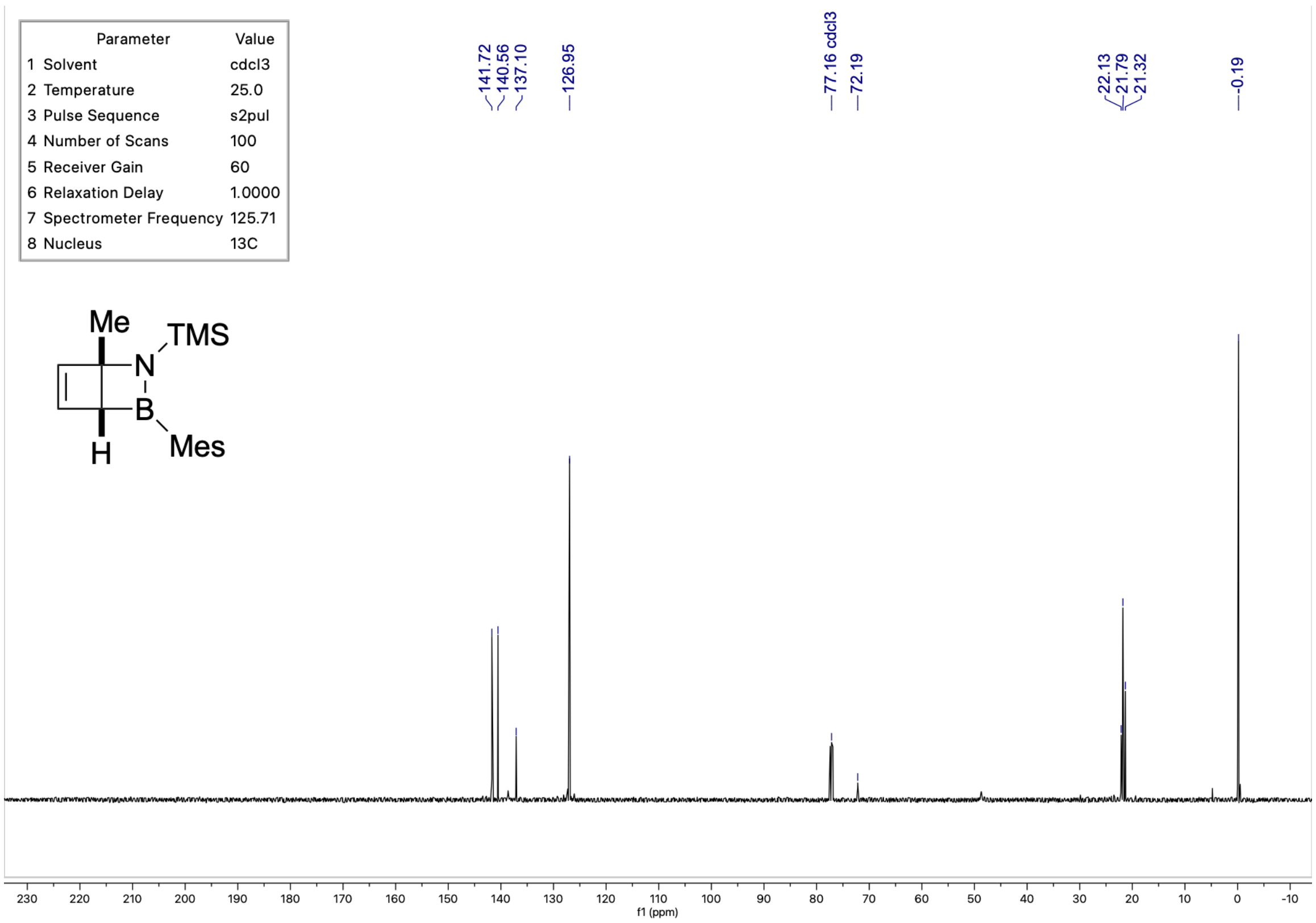
Parameter Value
1 Solvent cdcl3
2 Temperature 25.0
3 Pulse Sequence s2pul
4 Number of Scans 100
5 Receiver Gain 60
6 Relaxation Delay 1.0000
7 Spectrometer Frequency 125.71
8 Nucleus 13C
141.72
140.56
137.10
126.95
77.16 cdcl3
72.19
22.13
21.79
21.32
-0.19
Me
TMS
N
B
H
Mes
f1 (ppm)

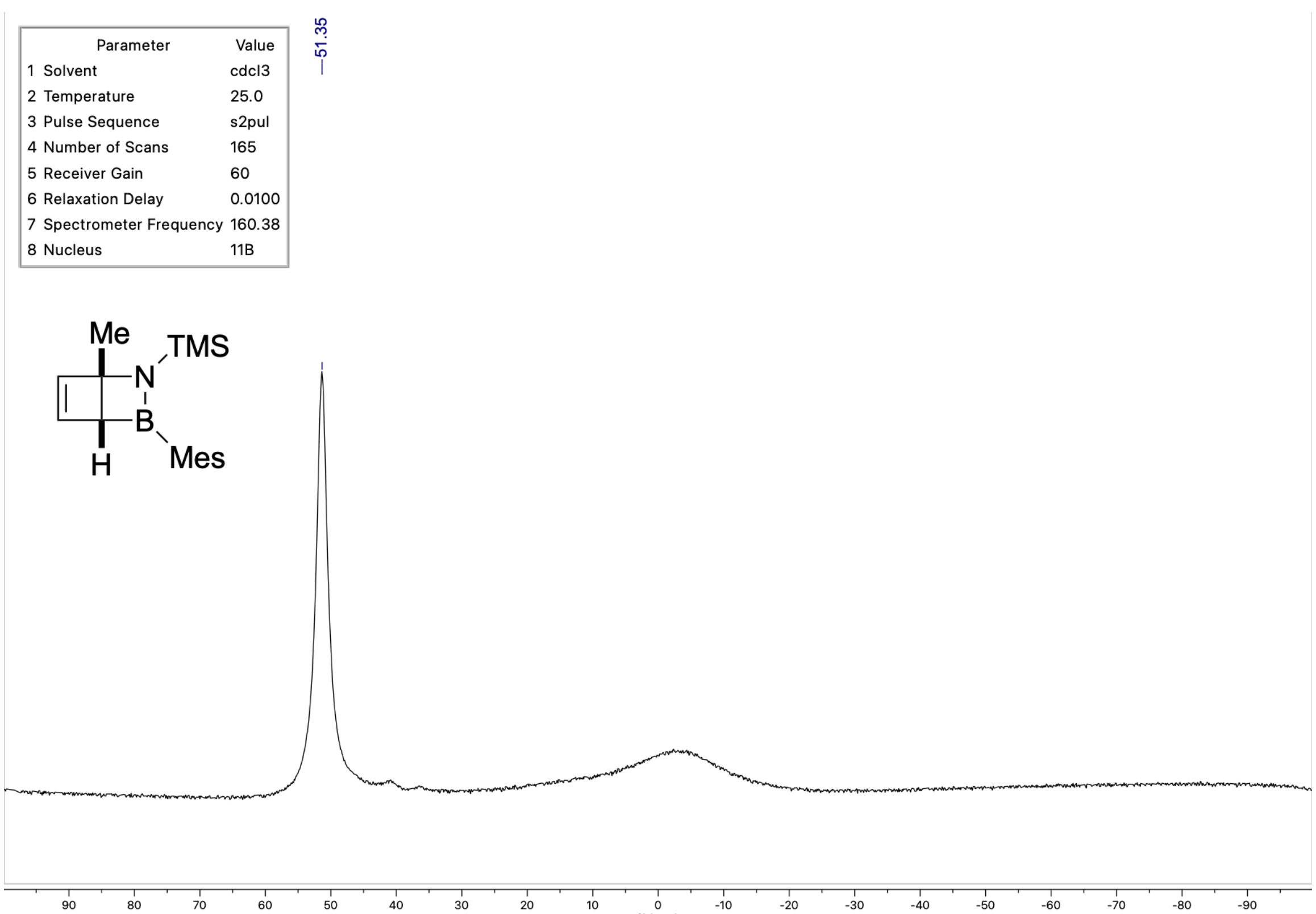
Parameter Value
1 Solvent cdcl3
2 Temperature 25.0
3 Pulse Sequence s2pul
4 Number of Scans 165
5 Receiver Gain 60
6 Relaxation Delay 0.0100
7 Spectrometer Frequency 160.38
8 Nucleus 11B
51.35
Me
TMS
N
B
H
Mes
f1 (ppm)

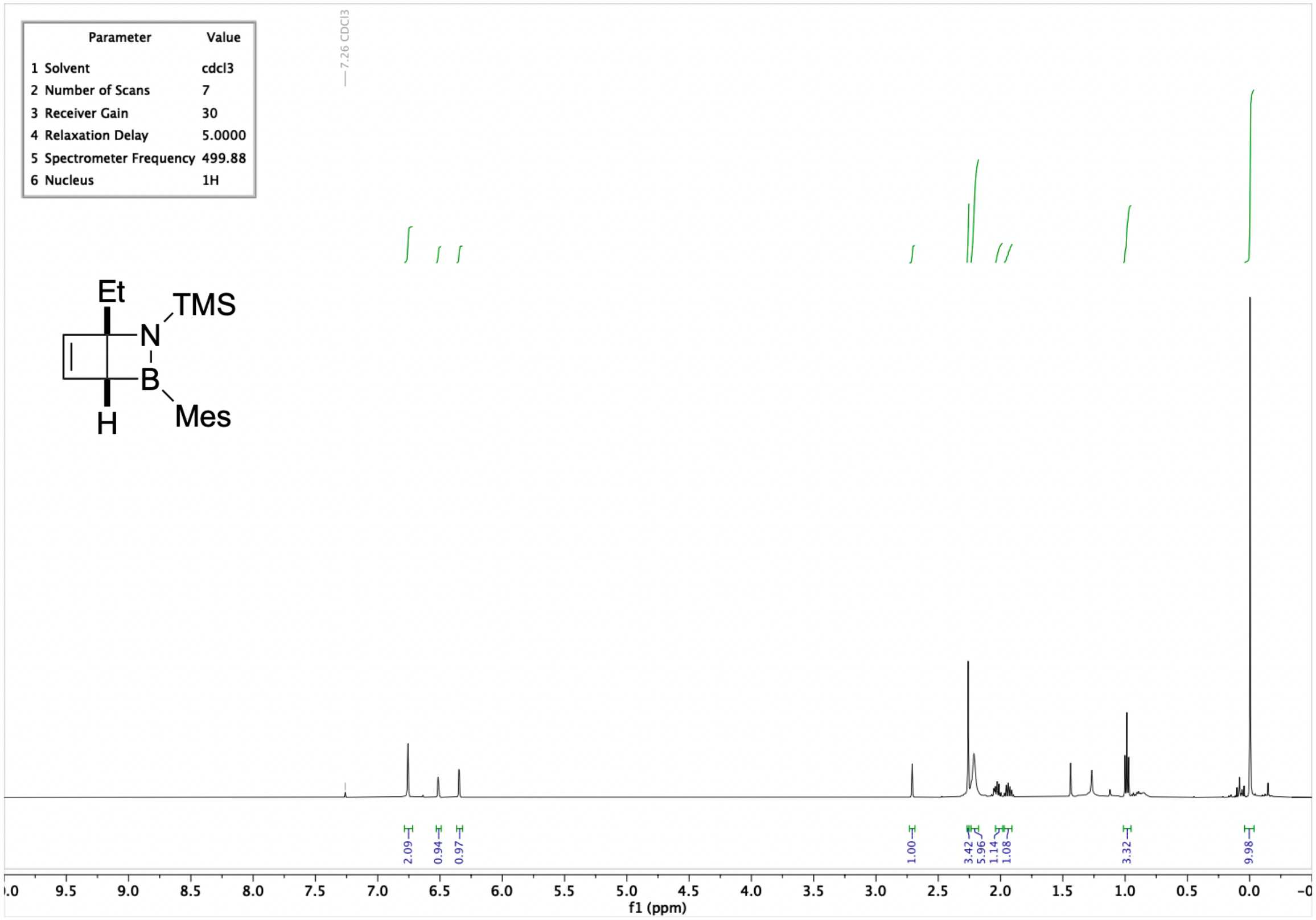
Parameter Value
1 Solvent cdcl3
2 Number of Scans 7
3 Receiver Gain 30
4 Relaxation Delay 5.0000
5 Spectrometer Frequency 499.88
6 Nucleus 1H
7.26 CDCl3
Et
TMS
N
B
H
Mes
2.09
0.94
0.97
1.00
3.42
5.96
1.14
1.08
3.32
9.98
f1 (ppm)

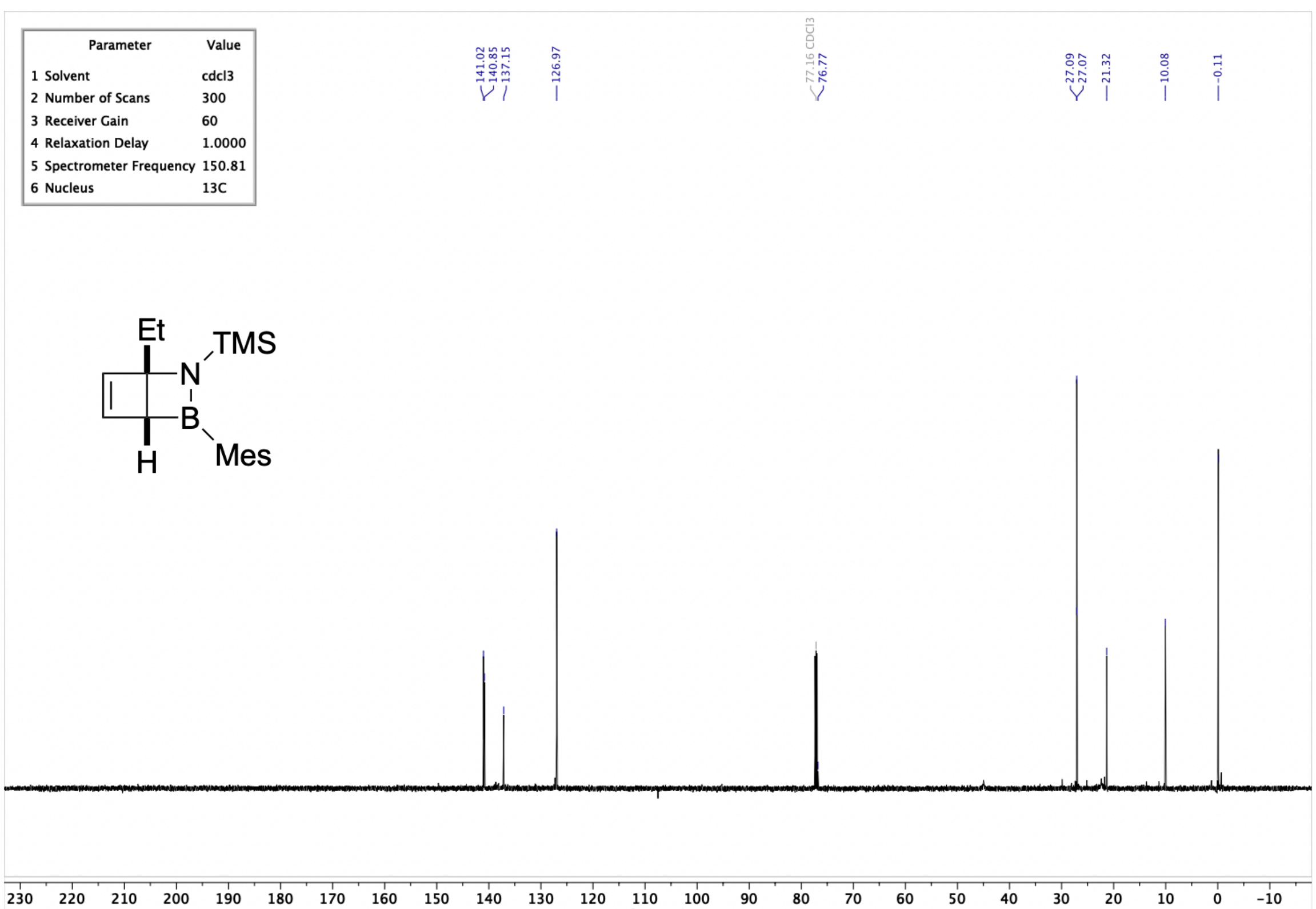
Parameter Value
1 Solvent cdcl3
2 Number of Scans 300
3 Receiver Gain 60
4 Relaxation Delay 1.0000
5 Spectrometer Frequency 150.81
6 Nucleus 13C
141.02
140.85
137.15
126.97
77.16 CDCl3
76.77
27.09
27.07
21.32
10.08
-0.11
Et
TMS
N
B
H
Mes
f1 (ppm)

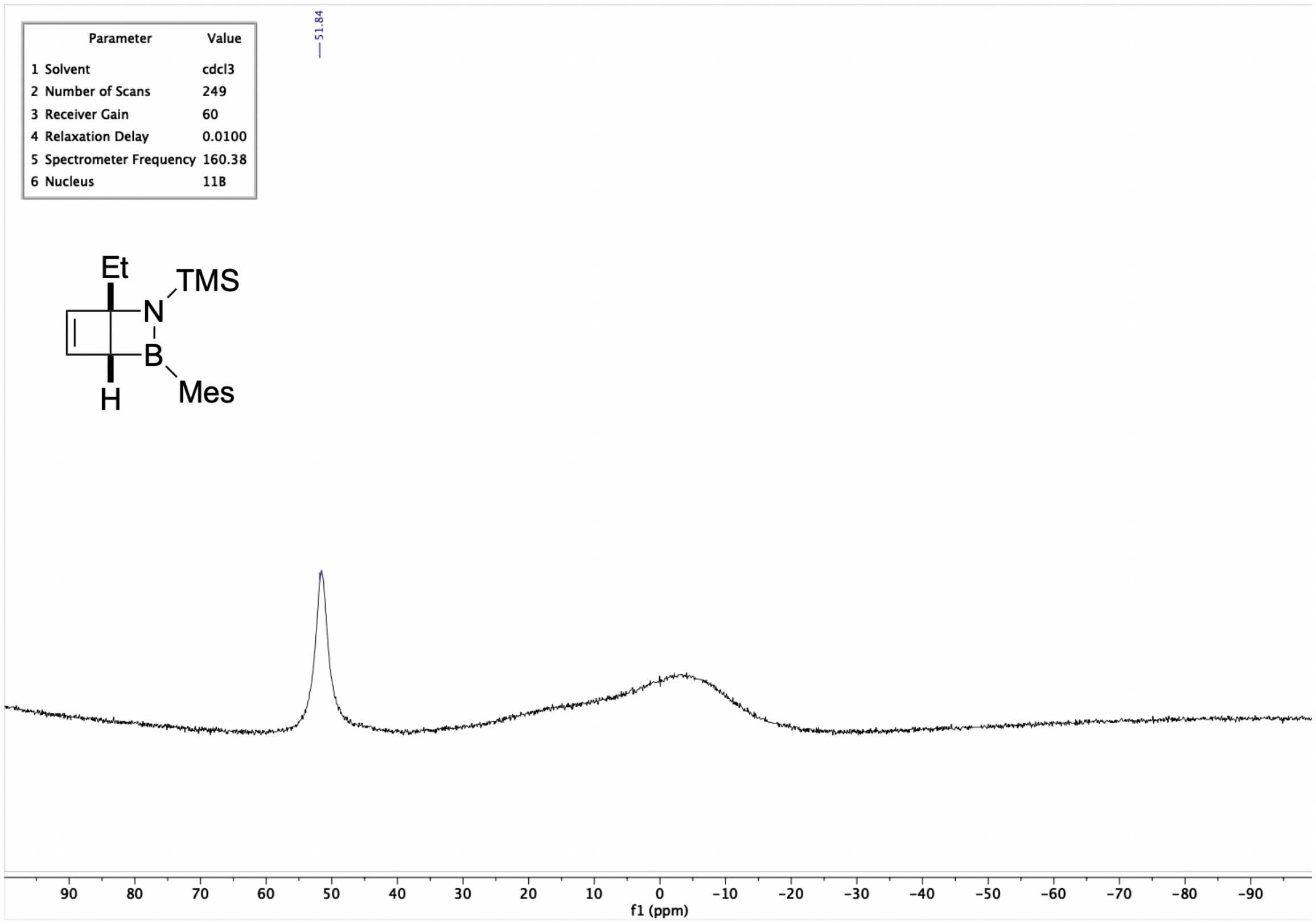

Parameter Value
1 Solvent cdcl3
2 Number of Scans 249
3 Receiver Gain 60
4 Relaxation Delay 0.0100
5 Spectrometer Frequency 160.38
6 Nucleus 11B
51.84
Et
TMS
N
B
H
Mes
f1 (ppm)

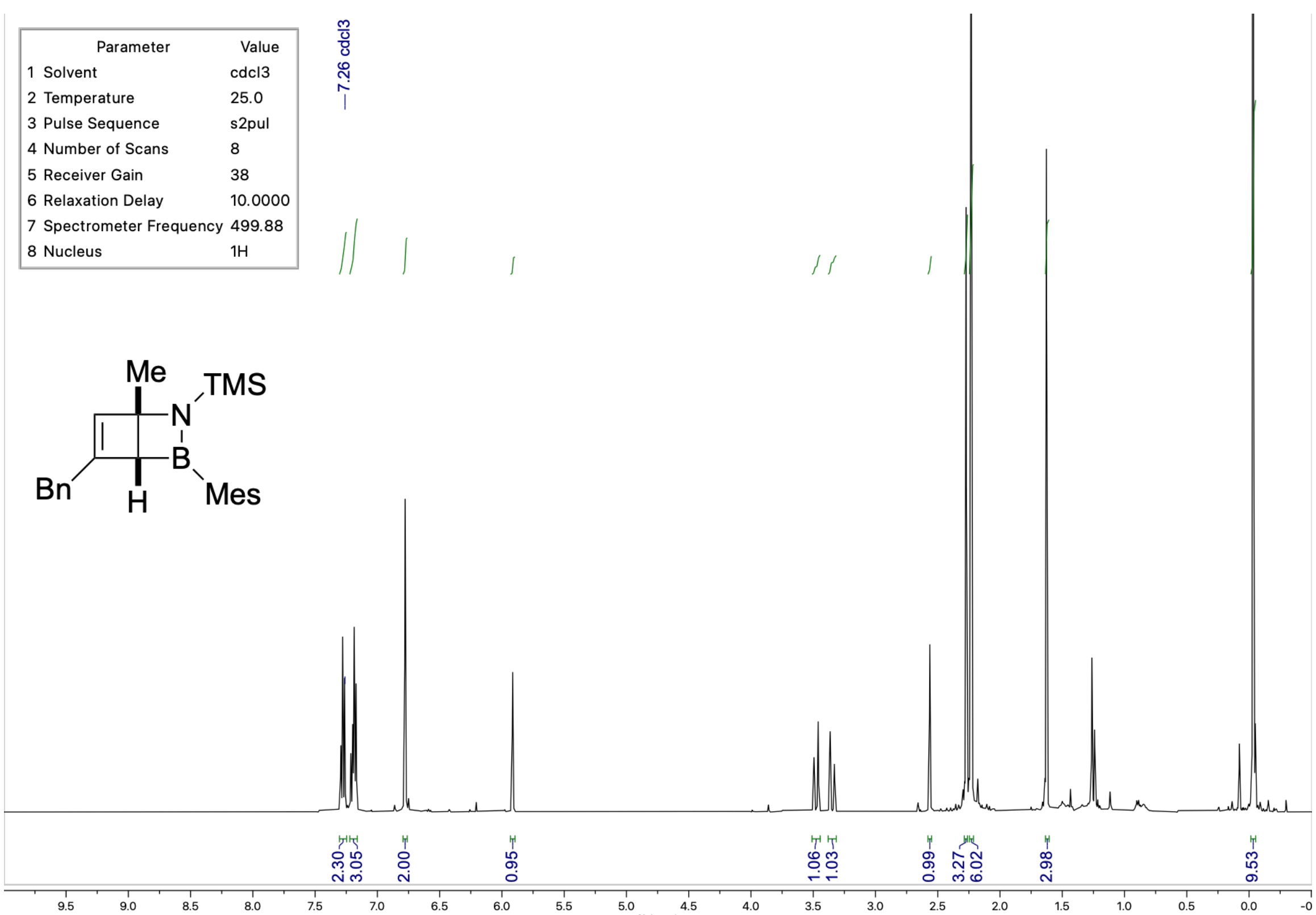

Parameter Value
1 Solvent cdcl3
2 Temperature 25.0
3 Pulse Sequence s2pul
4 Number of Scans 8
5 Receiver Gain 38
6 Relaxation Delay 10.0000
7 Spectrometer Frequency 499.88
8 Nucleus 1H
7.26 cdcl3
Me
TMS
N
B
Bn
H
Mes
2.30
3.05
2.00
0.95
1.06
1.03
0.99
3.27
6.02
2.98
9.53
f1 (ppm)

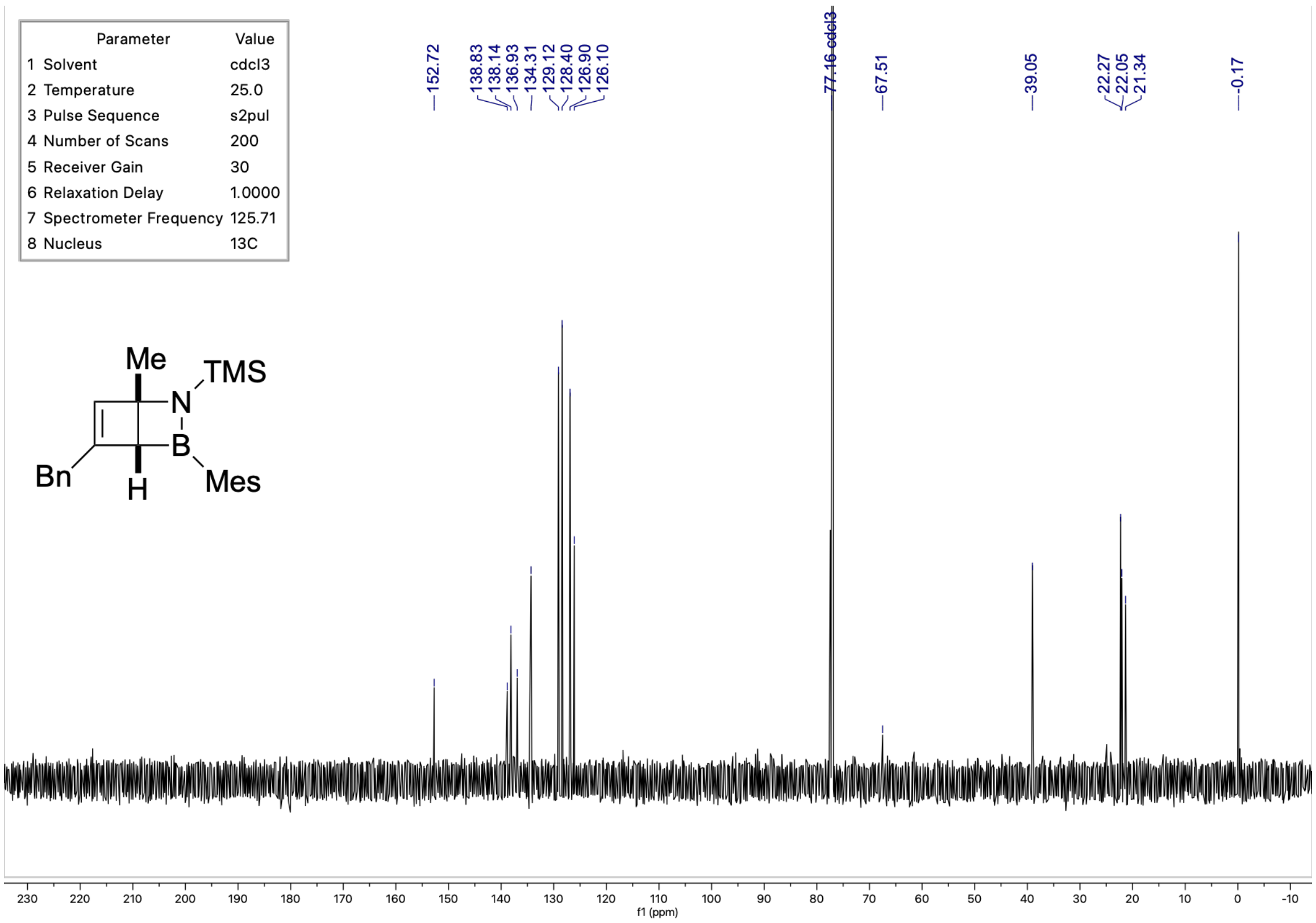
Parameter Value
1 Solvent cdcl3
2 Temperature 25.0
3 Pulse Sequence s2pul
4 Number of Scans 200
5 Receiver Gain 30
6 Relaxation Delay 1.0000
7 Spectrometer Frequency 125.71
8 Nucleus 13C
152.72
138.83
138.14
136.93
134.31
129.12
128.40
126.90
126.10
77.16 cdcl3
67.51
39.05
22.27
22.05
21.34
-0.17
Me
TMS
N
B
Bn
H
Mes
f1 (ppm)

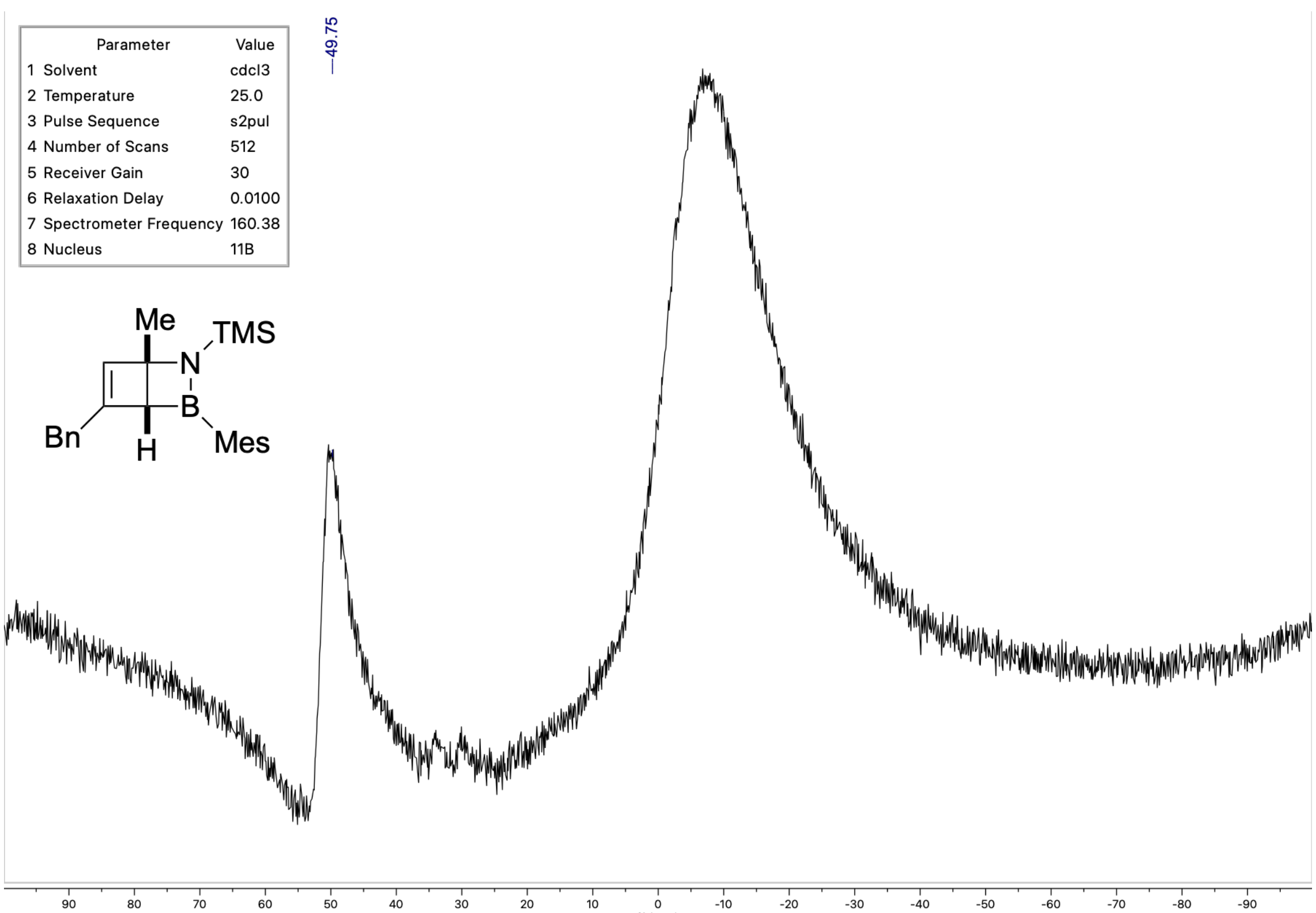
Parameter Value
1 Solvent cdcl3
2 Temperature 25.0
3 Pulse Sequence s2pul
4 Number of Scans 512
5 Receiver Gain 30
6 Relaxation Delay 0.0100
7 Spectrometer Frequency 160.38
8 Nucleus 11B
49.75
Me
TMS
N
B
Bn
H
Mes
f1 (ppm)

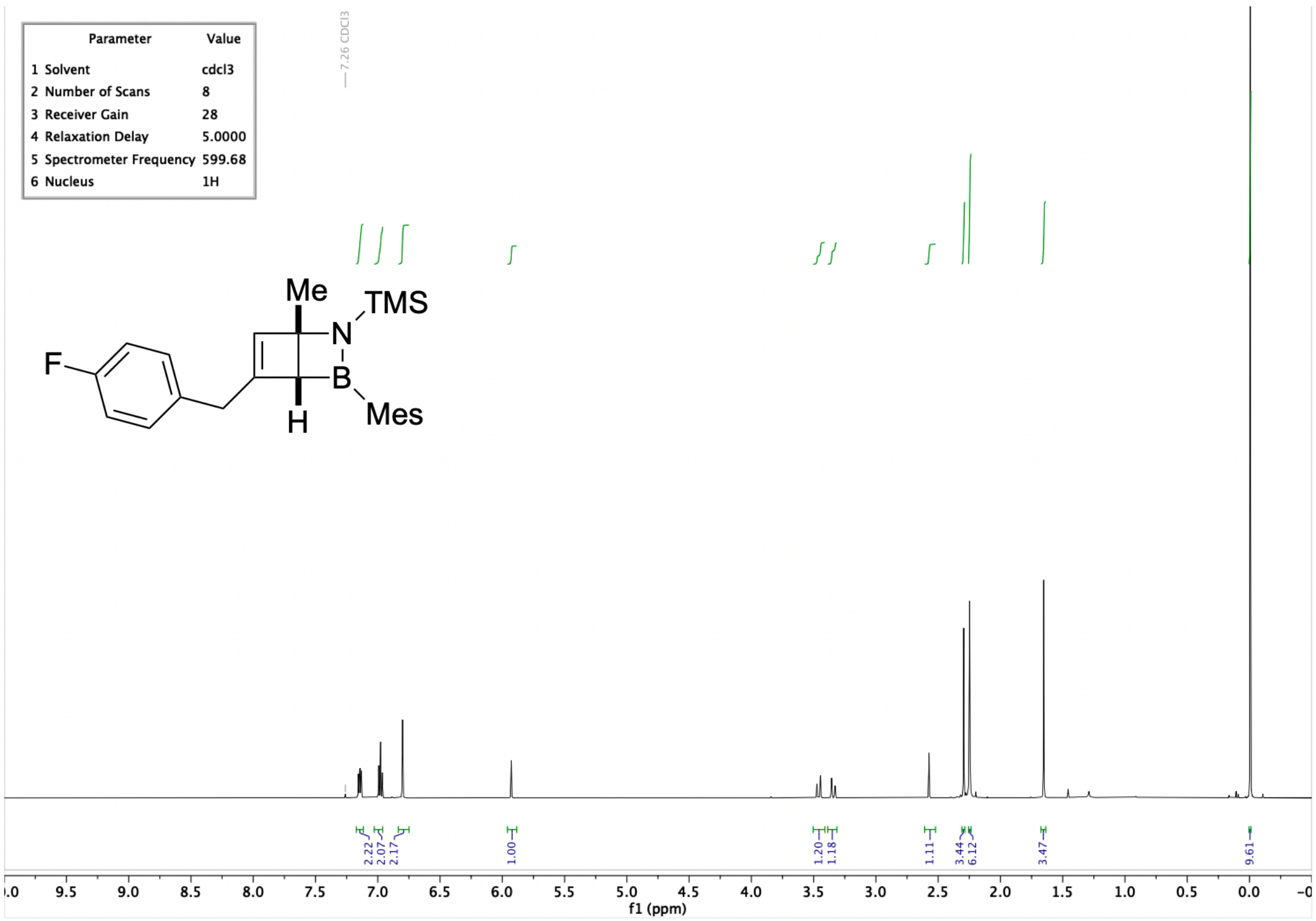
Parameter Value
1 Solvent cdcl3
2 Number of Scans 8
3 Receiver Gain 28
4 Relaxation Delay 5.0000
5 Spectrometer Frequency 599.68
6 Nucleus 1H
7.26 CDCl3
F
Me
TMS
N
B
H
Mes
2.22
2.07
2.17
1.00
1.20
1.18
1.11
3.44
6.12
3.47
9.61
f1 (ppm)

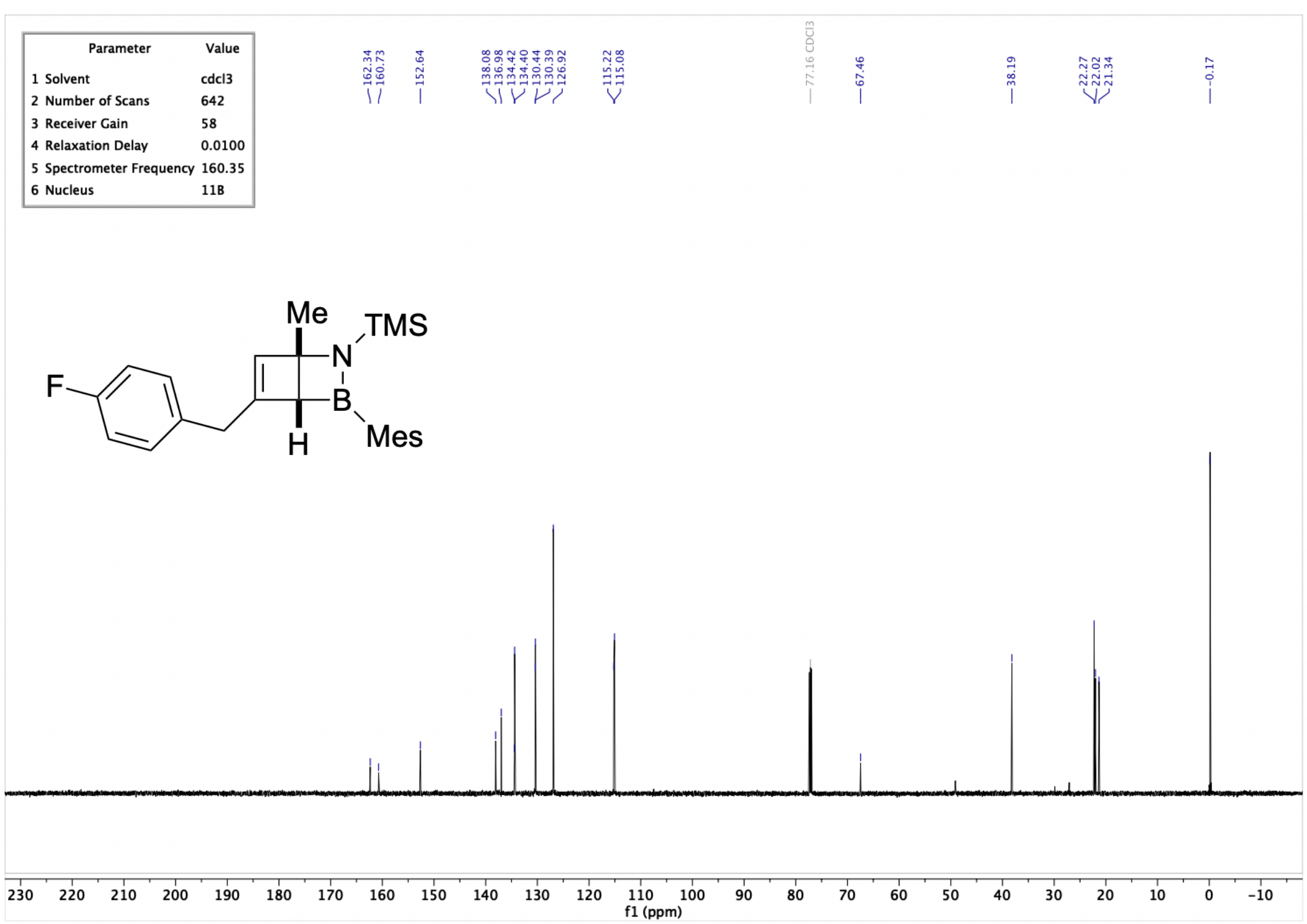
Parameter Value
1 Solvent cdcl3
2 Number of Scans 642
3 Receiver Gain 58
4 Relaxation Delay 0.0100
5 Spectrometer Frequency 160.35
6 Nucleus 11B
162.34
160.73
152.64
138.08
136.98
134.42
134.40
130.44
130.39
126.92
115.22
115.08
77.16 CDCl3
67.46
38.19
22.27
22.02
21.34
-0.17
F
Me
TMS
N
B
H
Mes
f1 (ppm)

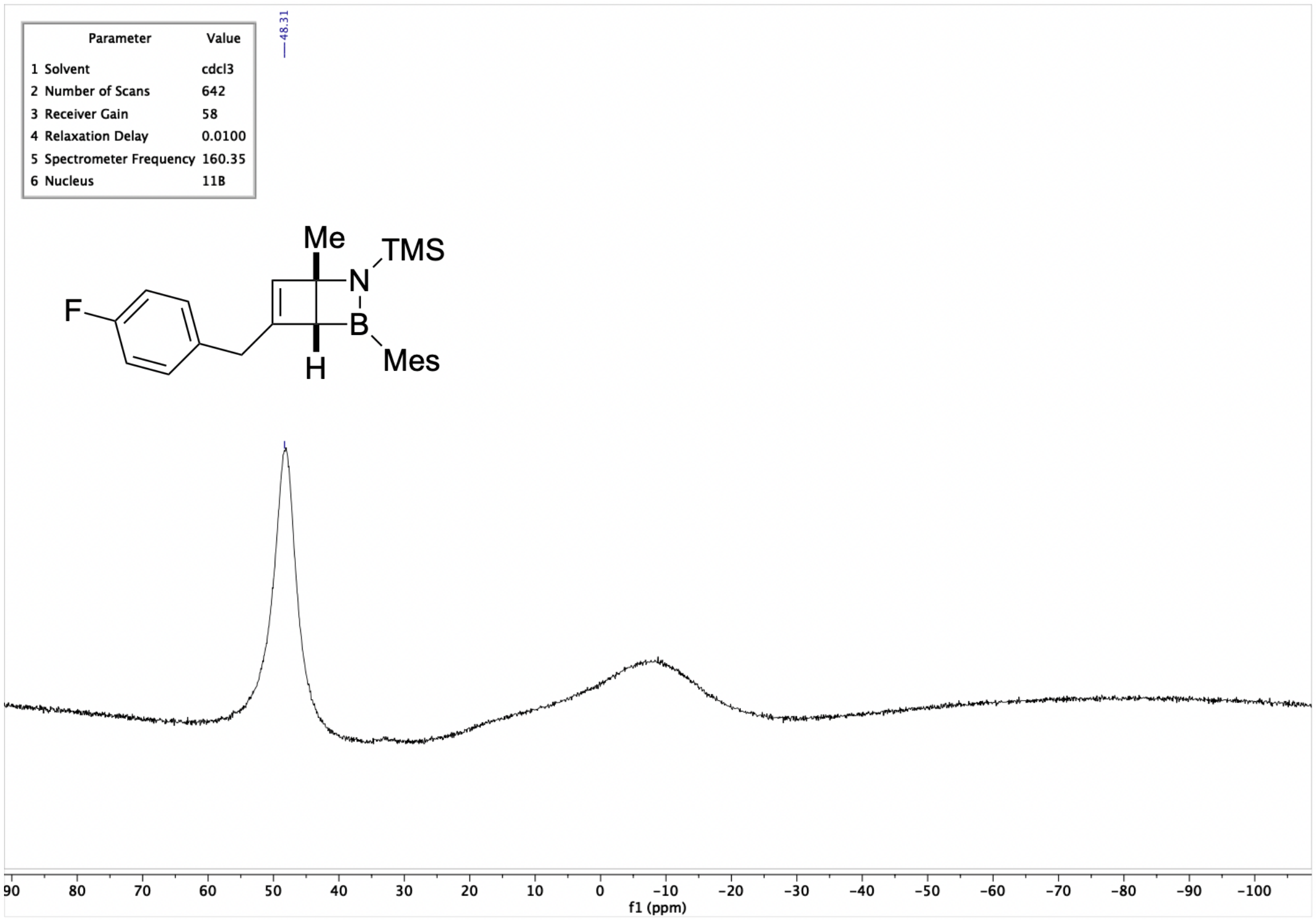

Parameter Value
1 Solvent cdcl3
2 Number of Scans 642
3 Receiver Gain 58
4 Relaxation Delay 0.0100
5 Spectrometer Frequency 160.35
6 Nucleus 11B
48.31
Me
TMS
N
B
F
H
Mes
90 80 70 60 50 40 30 20 10 0 -10 -20 -30 -40 -50 -60 -70 -80 -90 -100
f1 (ppm)

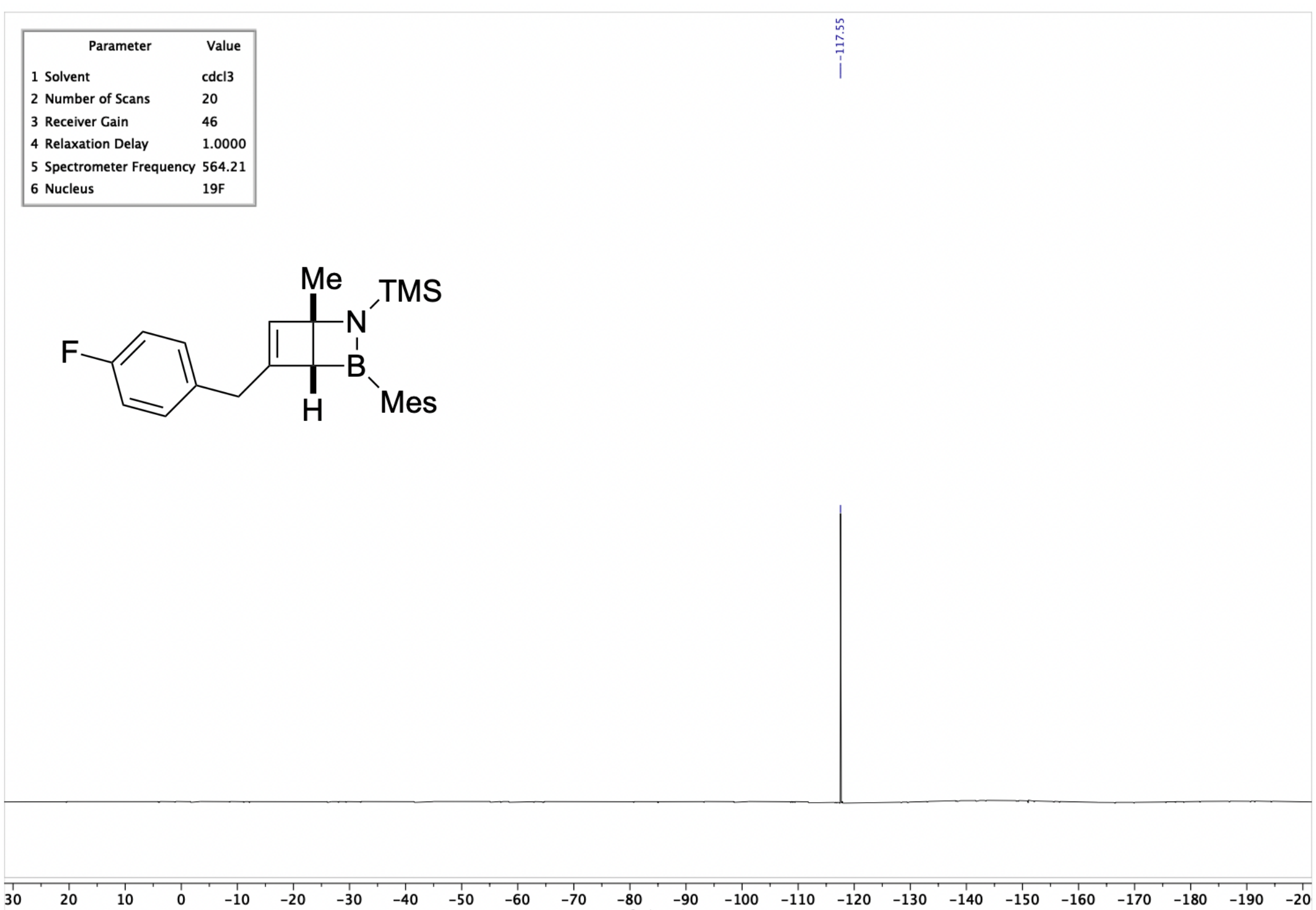

Parameter Value
1 Solvent cdcl3
2 Number of Scans 20
3 Receiver Gain 46
4 Relaxation Delay 1.0000
5 Spectrometer Frequency 564.21
6 Nucleus 19F
-117.55
Me
TMS
N
B
F
H
Mes
30 20 10 0 -10 -20 -30 -40 -50 -60 -70 -80 -90 -100 -110 -120 -130 -140 -150 -160 -170 -180 -190 -20
f1 (ppm)

Parameter	Value
1 Solvent	cdcl3
2 Temperature	25.0
3 Pulse Sequence	s2pul
4 Number of Scans	8
5 Receiver Gain	32
6 Relaxation Delay	10.0000
7 Spectrometer Frequency	499.88
8 Nucleus	1H

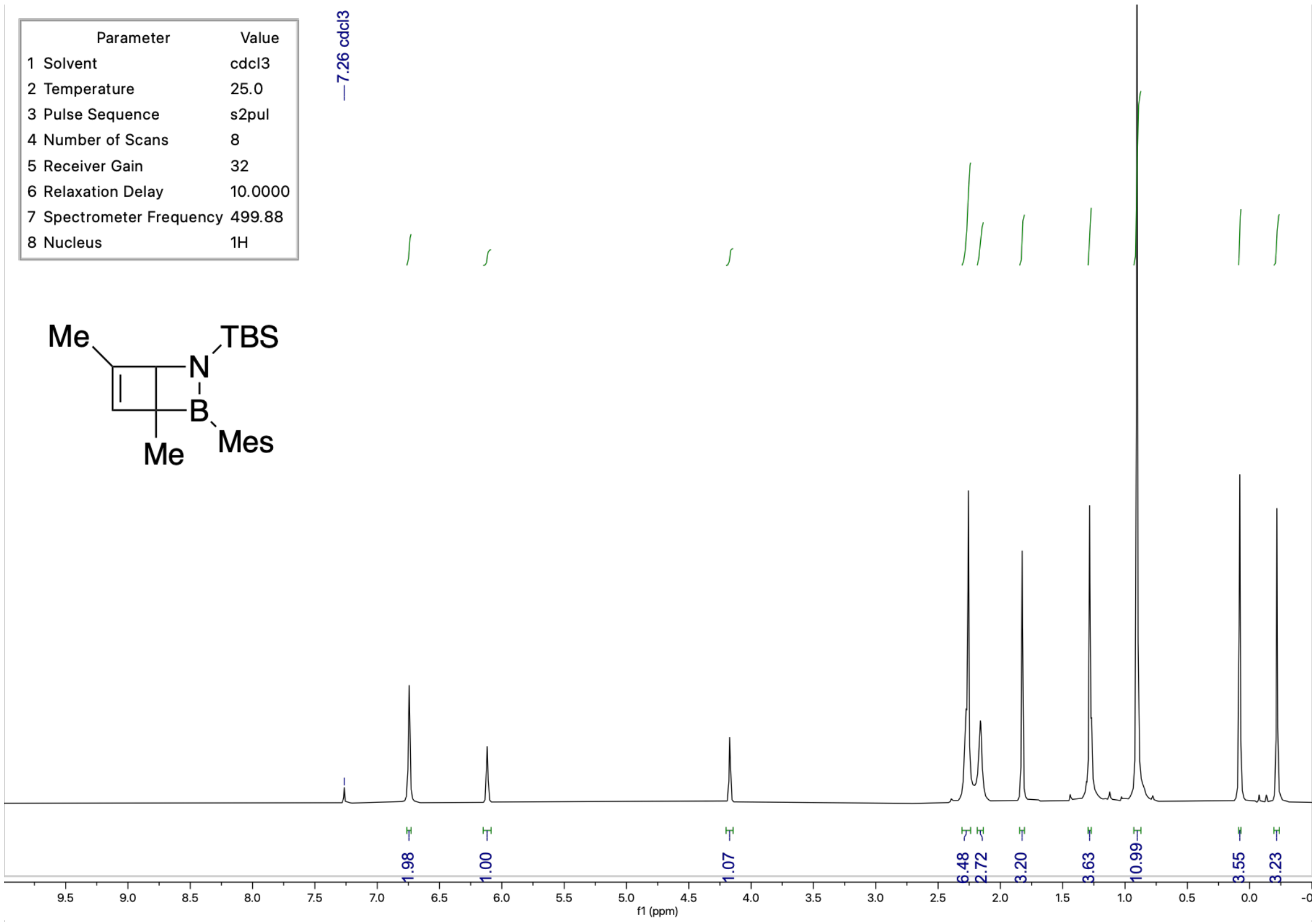

Parameter	Value
1 Solvent	cdcl3
2 Temperature	25.0
3 Pulse Sequence	s2pul
4 Number of Scans	200
5 Receiver Gain	30
6 Relaxation Delay	1.0000
7 Spectrometer Frequency	125.71
8 Nucleus	13C

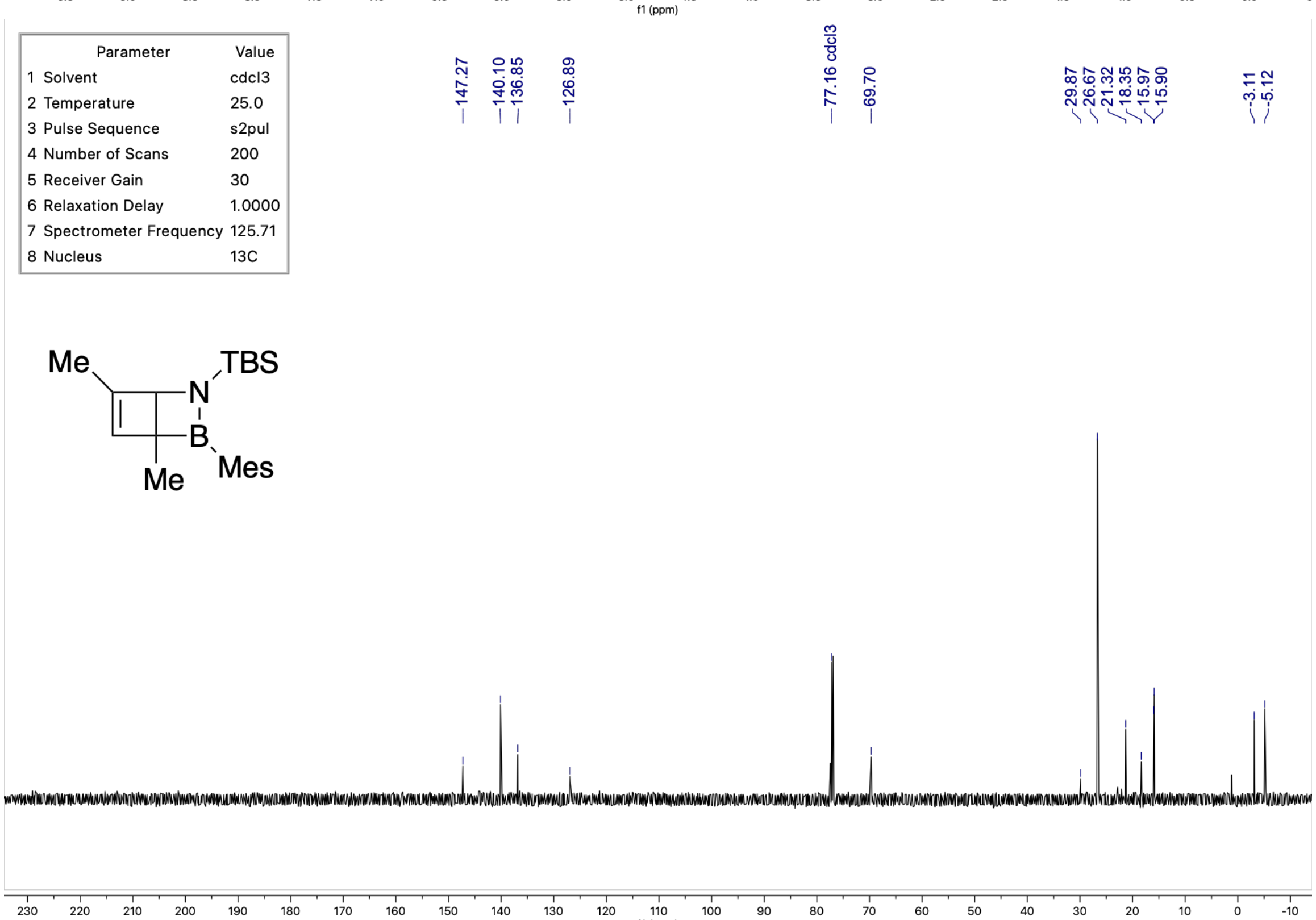

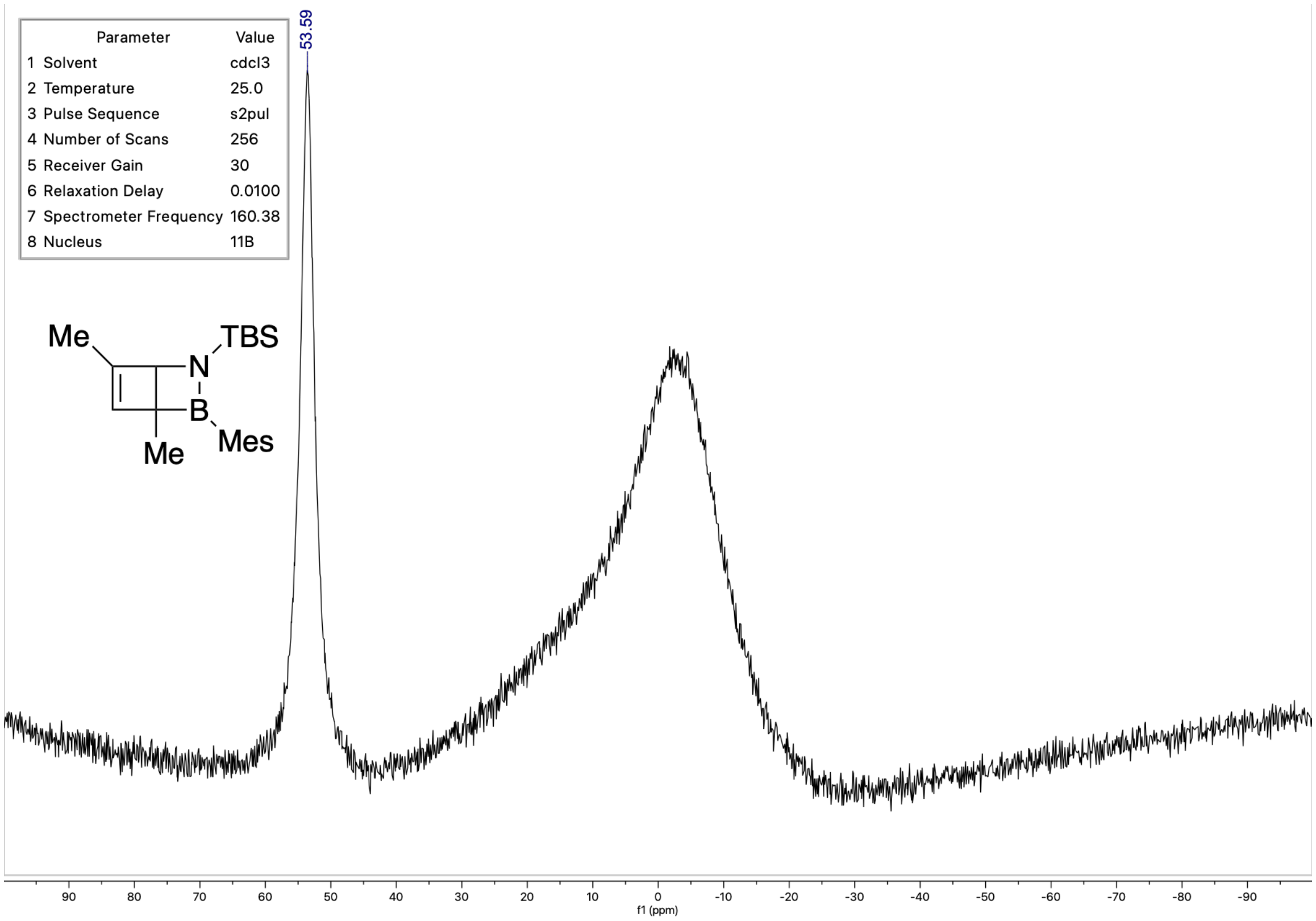
Parameter Value
1 Solvent cdcl3
2 Temperature 25.0
3 Pulse Sequence s2pul
4 Number of Scans 256
5 Receiver Gain 30
6 Relaxation Delay 0.0100
7 Spectrometer Frequency 160.38
8 Nucleus 11B
53.59
Me
TBS
N
B
Me
Mes
f1 (ppm)

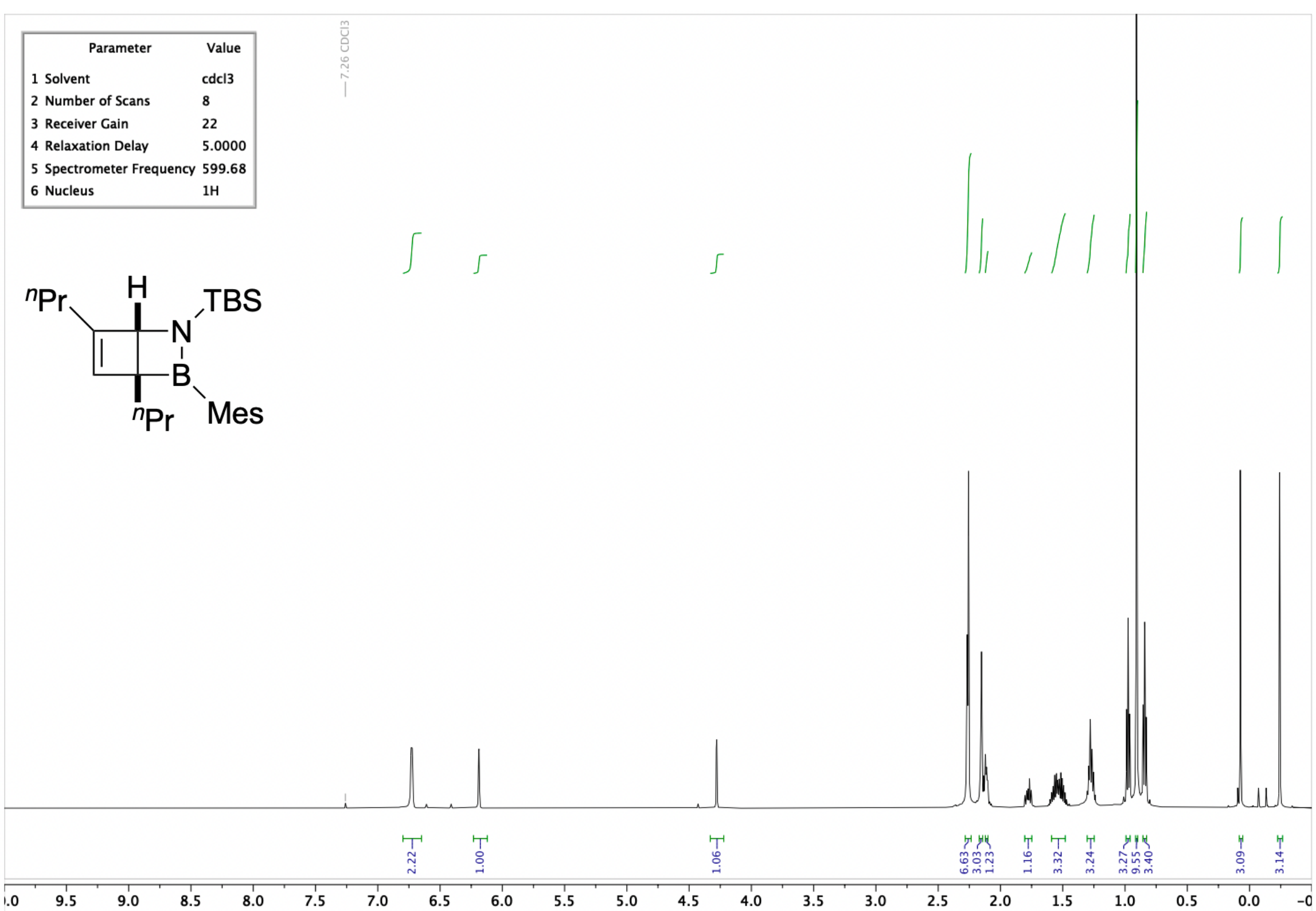
Parameter Value
1 Solvent cdcl3
2 Number of Scans 8
3 Receiver Gain 22
4 Relaxation Delay 5.0000
5 Spectrometer Frequency 599.68
6 Nucleus 1H
7.26 CDCl3
nPr
H
TBS
N
B
nPr
Mes
2.22
1.00
1.06
6.63
3.03
1.23
1.16
3.32
3.24
3.27
9.55
3.40
3.09
3.14
f1 (ppm)

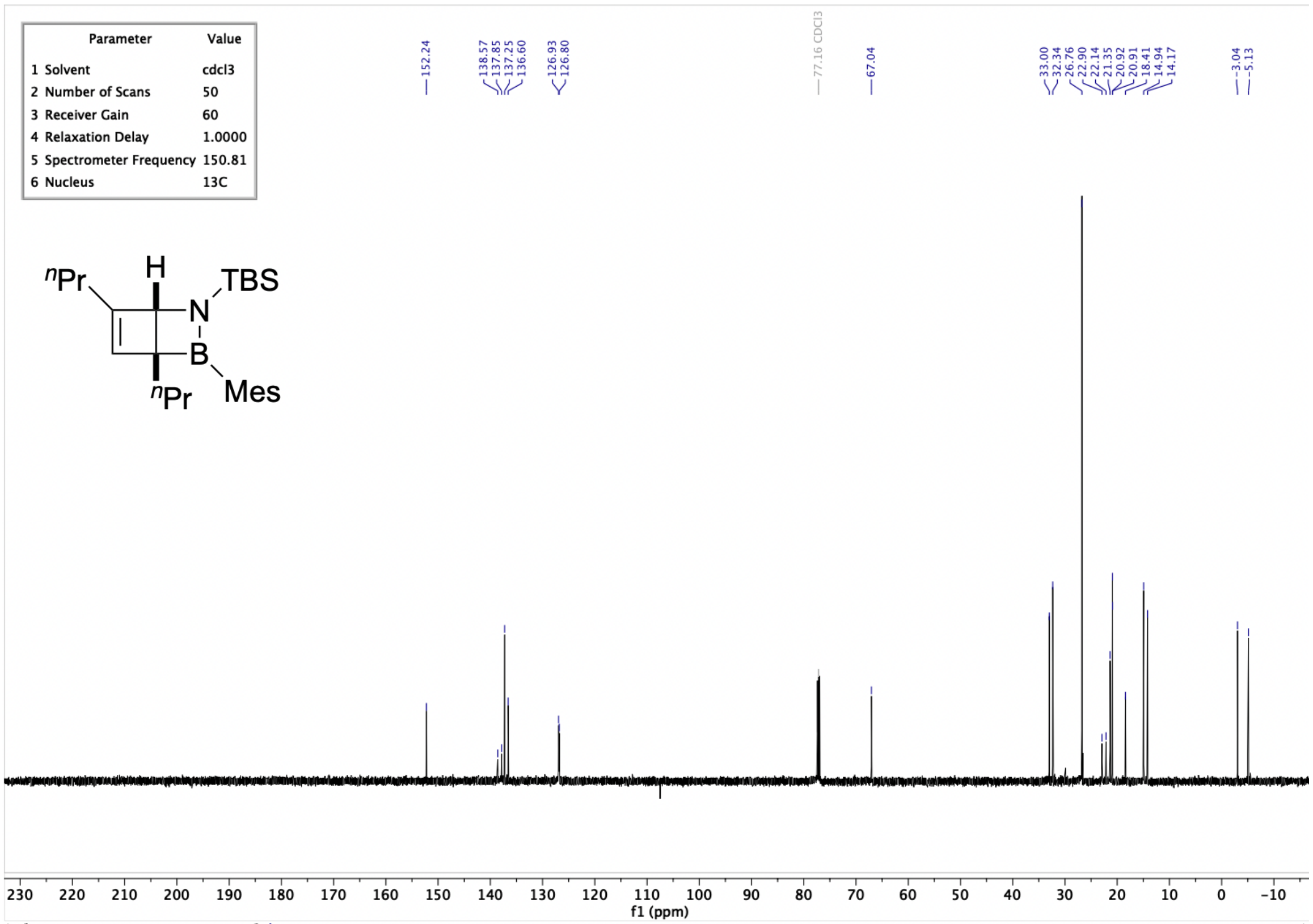
Parameter Value
1 Solvent cdcl3
2 Number of Scans 50
3 Receiver Gain 60
4 Relaxation Delay 1.0000
5 Spectrometer Frequency 150.81
6 Nucleus 13C
152.24
138.57
137.85
137.25
136.60
126.93
126.80
77.16 CDCl3
67.04
33.00
32.34
26.76
22.90
22.14
21.35
20.92
20.91
18.41
14.94
14.17
-3.04
-5.13
ⁿPr
H
TBS
N
B
ⁿPr
Mes
f1 (ppm)

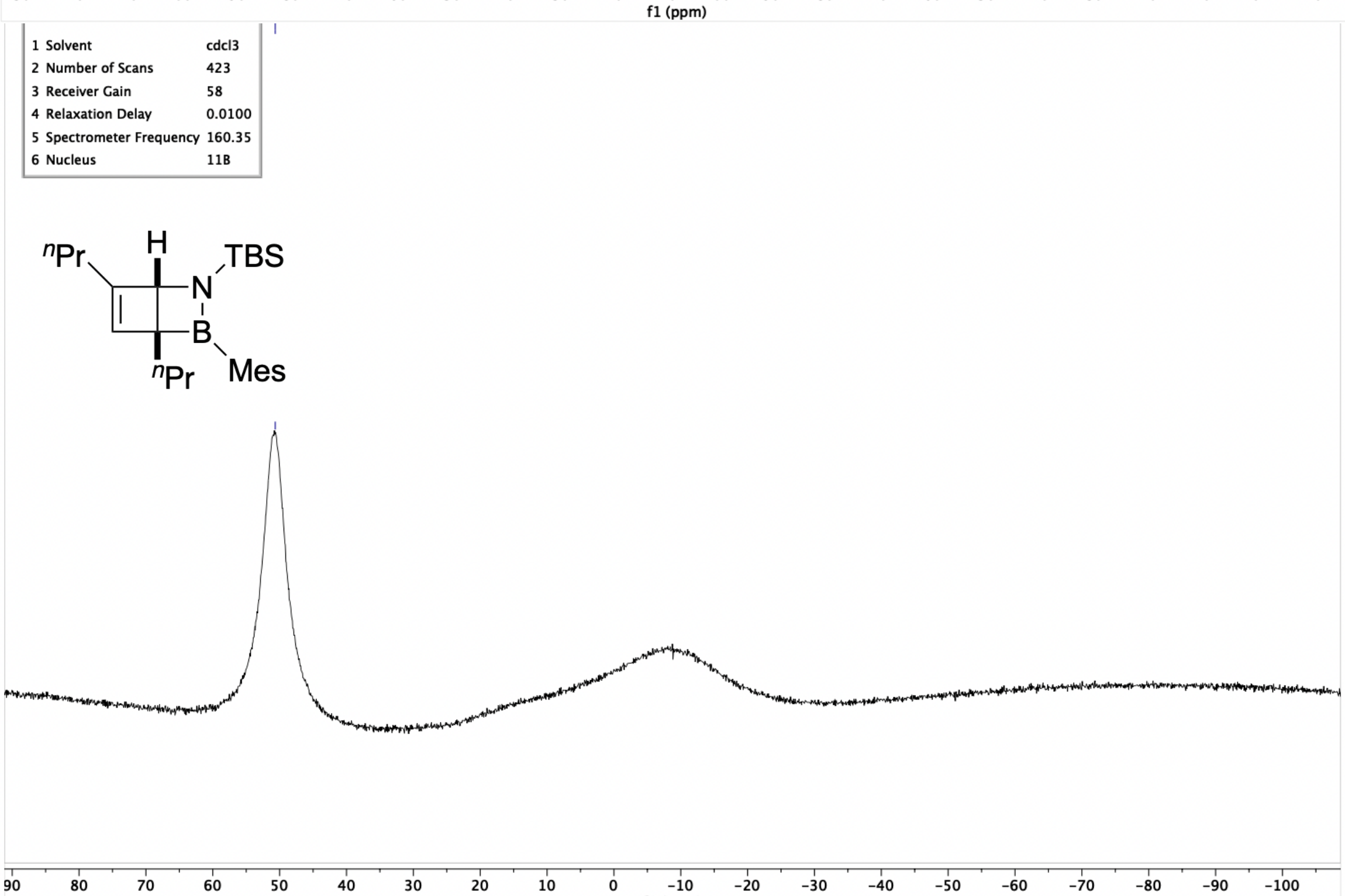
1 Solvent cdcl3
2 Number of Scans 423
3 Receiver Gain 58
4 Relaxation Delay 0.0100
5 Spectrometer Frequency 160.35
6 Nucleus 11B
ⁿPr
H
TBS
N
B
ⁿPr
Mes
f1 (ppm)

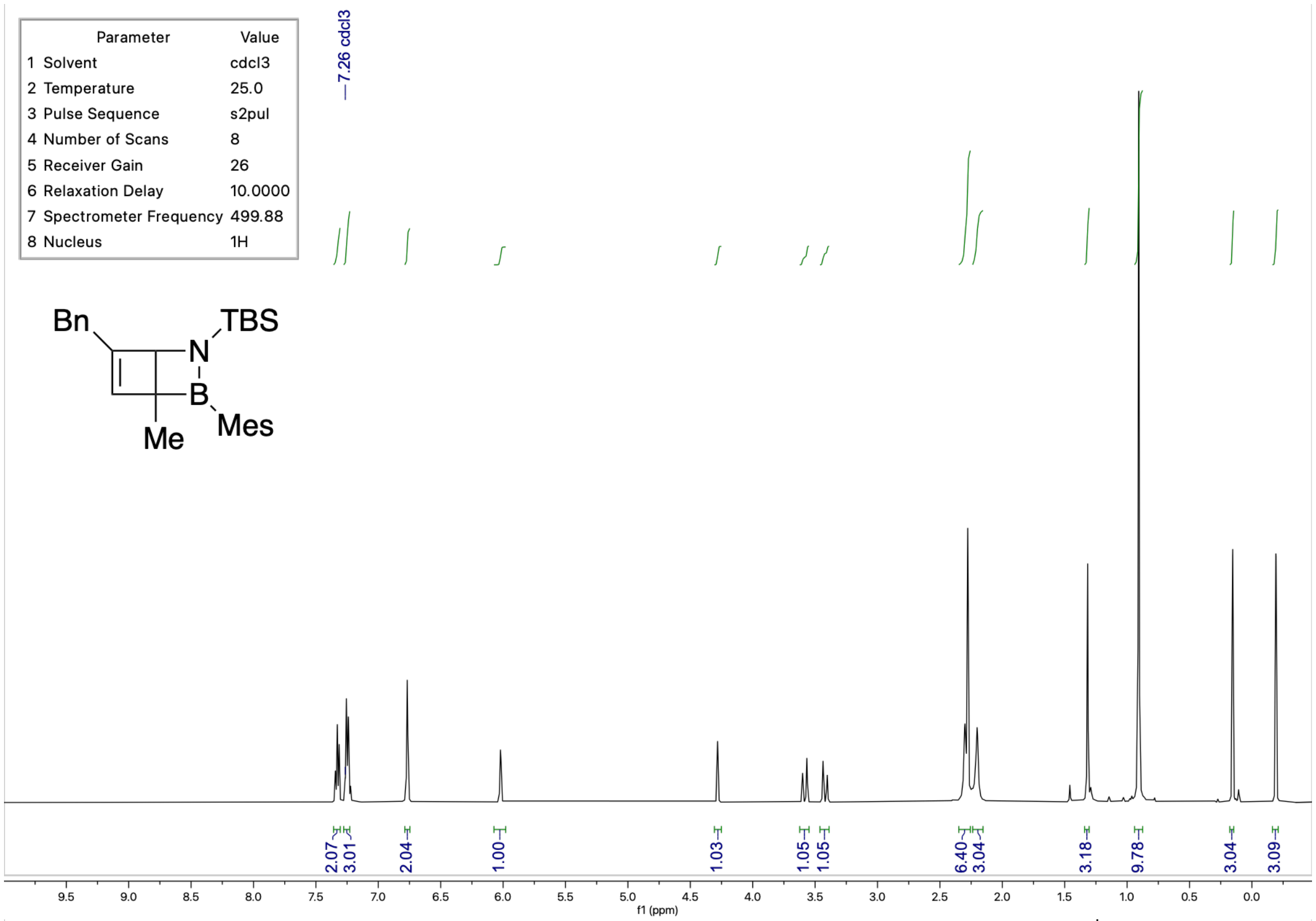

Parameter Value
1 Solvent cdcl3
2 Temperature 25.0
3 Pulse Sequence s2pul
4 Number of Scans 8
5 Receiver Gain 26
6 Relaxation Delay 10.0000
7 Spectrometer Frequency 499.88
8 Nucleus 1H
Bn
TBS
N
B
Mes
Me
7.26 cdcl3
2.07
3.01
2.04
1.00
1.03
1.05
1.05
6.40
3.04
3.18
9.78
3.04
3.09
f1 (ppm)

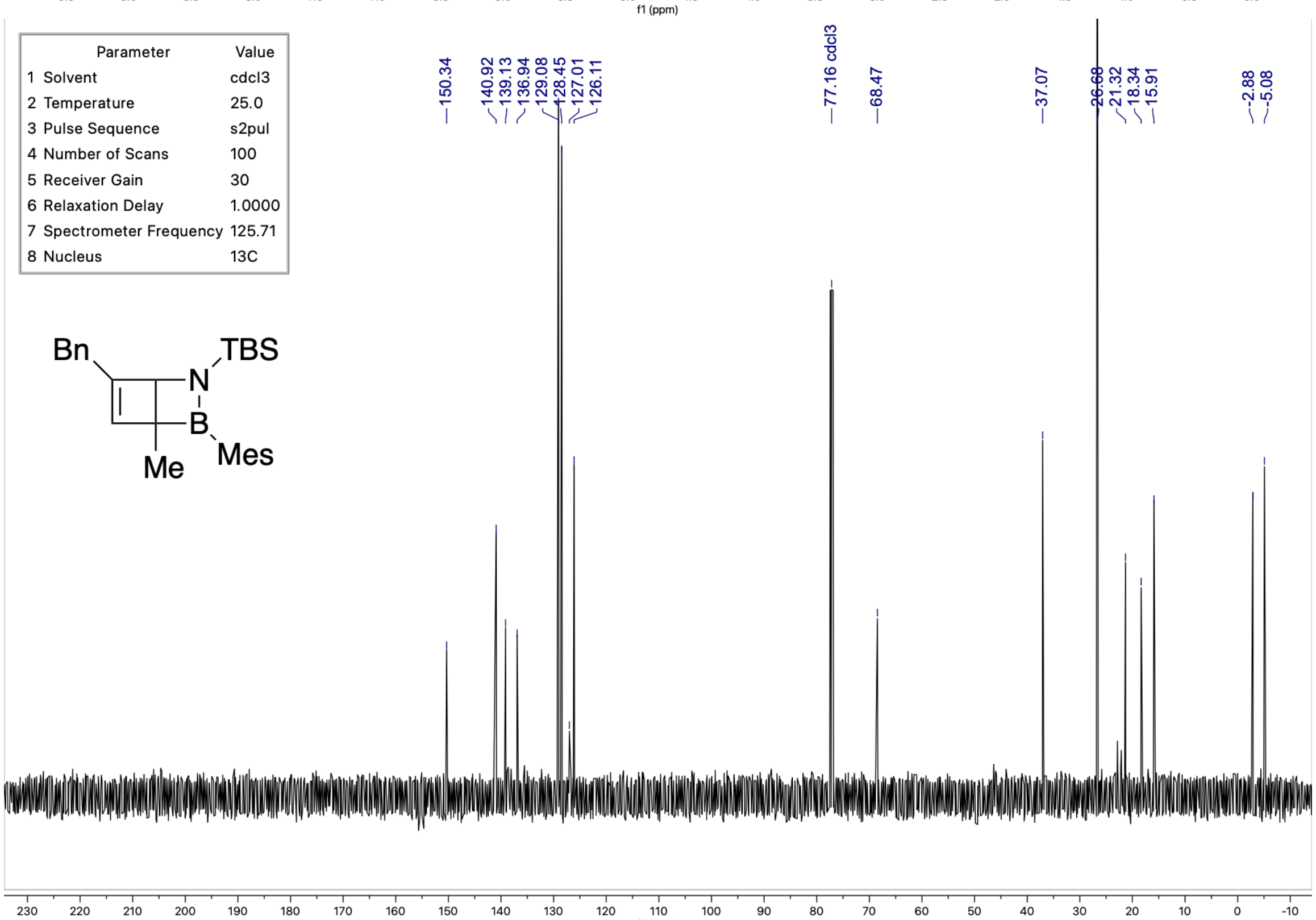

Parameter Value
1 Solvent cdcl3
2 Temperature 25.0
3 Pulse Sequence s2pul
4 Number of Scans 100
5 Receiver Gain 30
6 Relaxation Delay 1.0000
7 Spectrometer Frequency 125.71
8 Nucleus 13C
Bn
TBS
N
B
Mes
Me
150.34
140.92
139.13
136.94
129.08
128.45
127.01
126.11
77.16 cdcl3
68.47
37.07
26.68
21.32
18.34
15.91
-2.88
-5.08
f1 (ppm)

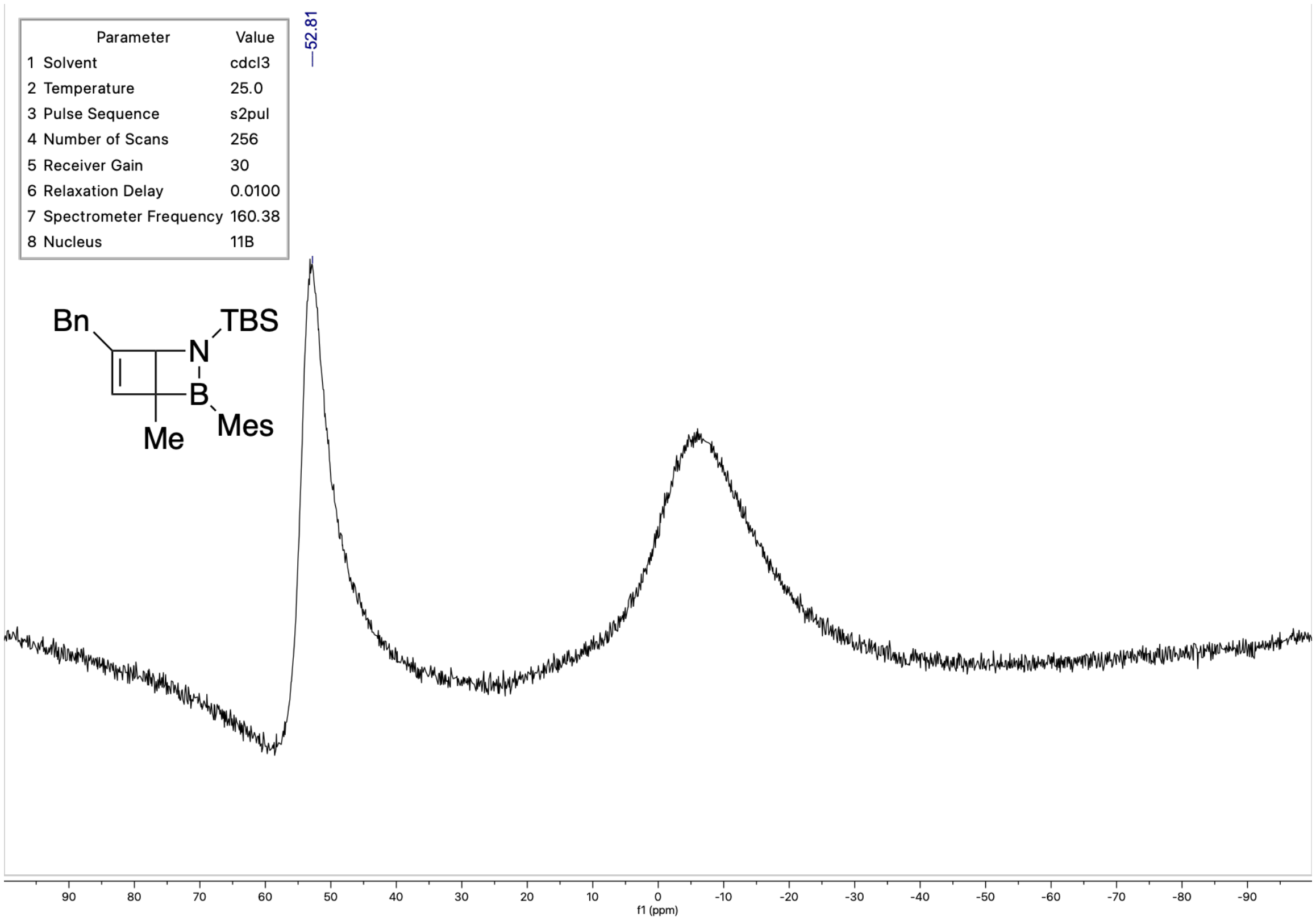
Parameter Value
1 Solvent cdcl3
2 Temperature 25.0
3 Pulse Sequence s2pul
4 Number of Scans 256
5 Receiver Gain 30
6 Relaxation Delay 0.0100
7 Spectrometer Frequency 160.38
8 Nucleus 11B
52.81
Bn
TBS
N
B
Me
Mes
f1 (ppm)

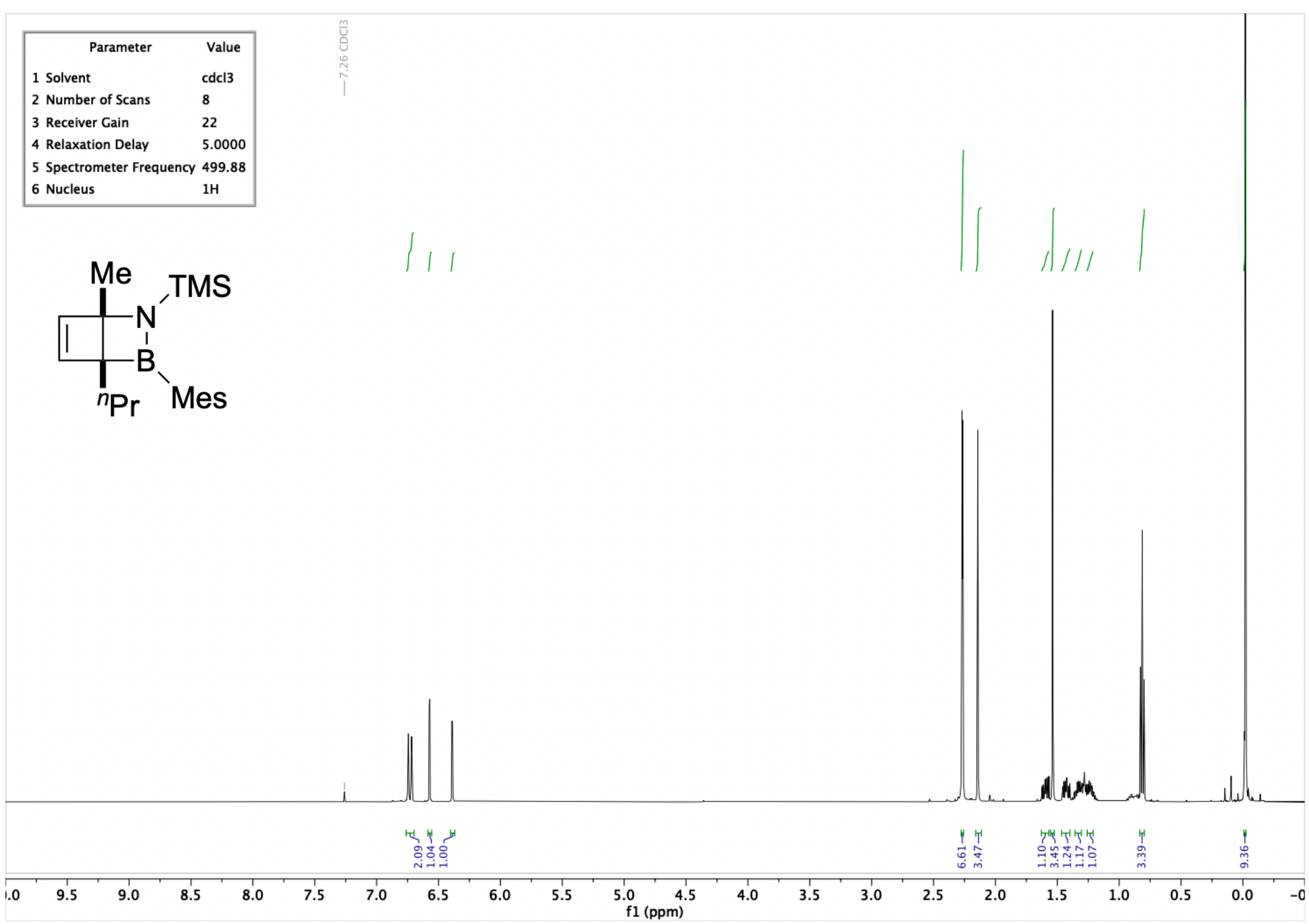
Parameter Value
1 Solvent cdcl3
2 Number of Scans 8
3 Receiver Gain 22
4 Relaxation Delay 5.0000
5 Spectrometer Frequency 499.88
6 Nucleus 1H
7.26 CDCl3
Me
TMS
N
B
nPr
Mes
2.09
1.04
1.00
6.61
3.47
1.10
3.45
1.24
1.17
1.07
3.39
9.36
f1 (ppm)

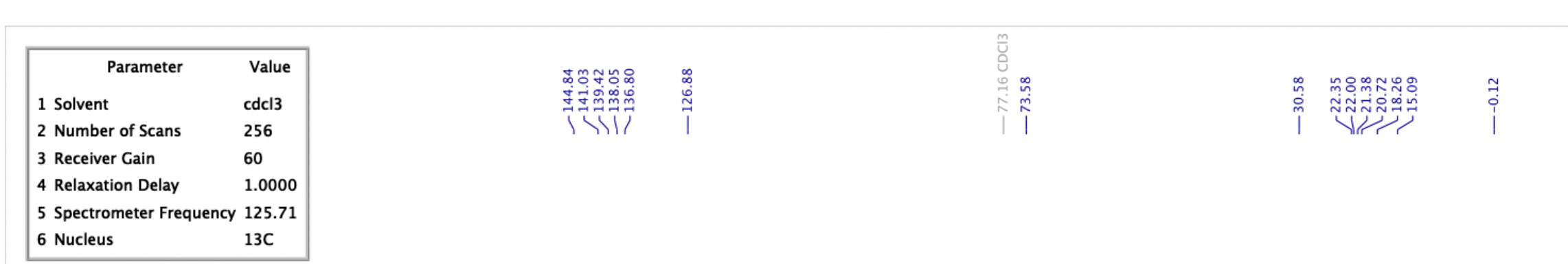
Parameter Value
1 Solvent cdcl3
2 Number of Scans 256
3 Receiver Gain 60
4 Relaxation Delay 1.0000
5 Spectrometer Frequency 125.71
6 Nucleus 13C
144.84
141.03
139.42
138.05
136.80
126.88
77.16 CDCl3
73.58
30.58
22.35
22.00
21.38
20.72
18.26
15.09
-0.12

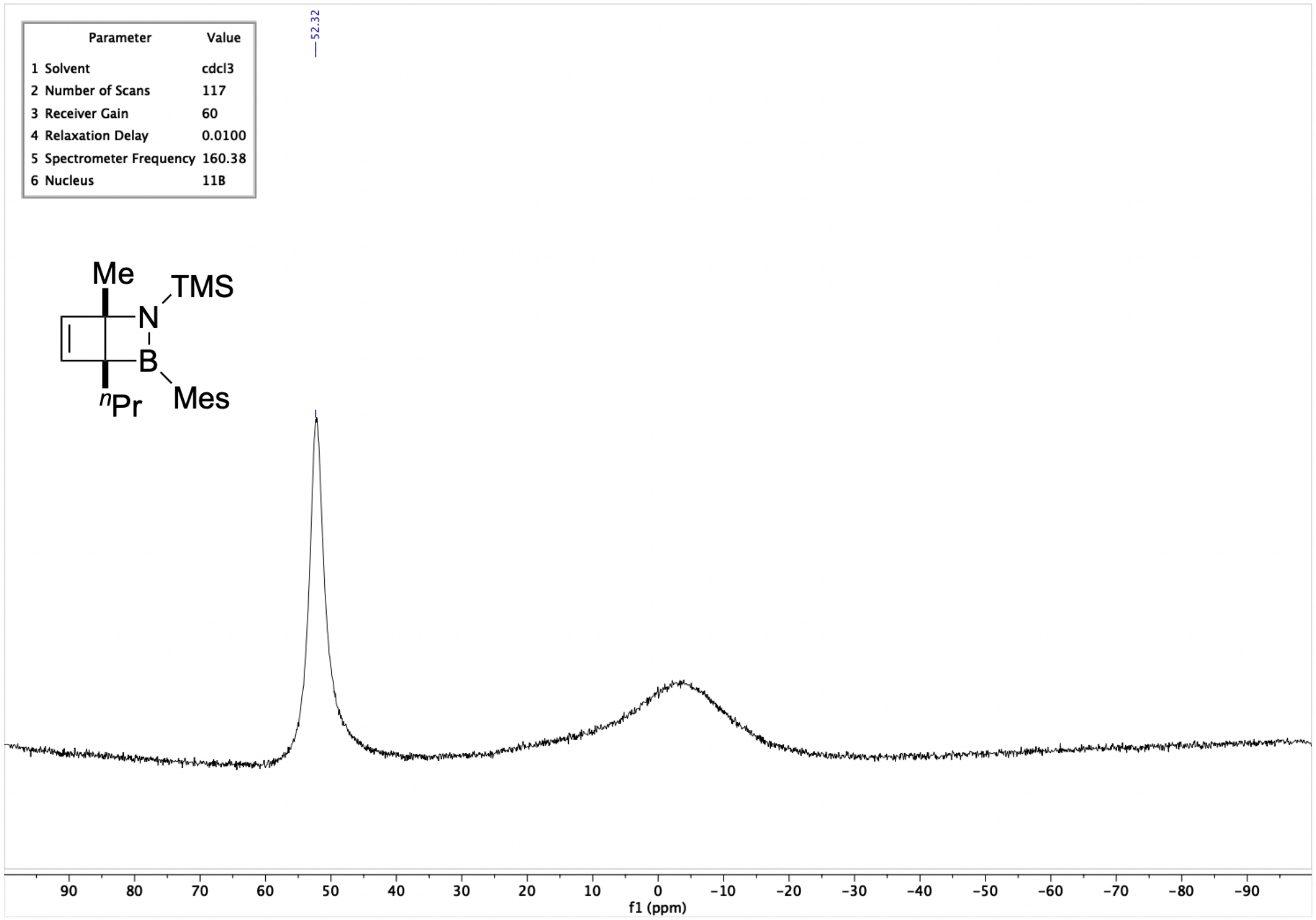

Parameter Value
1 Solvent cdcl3
2 Number of Scans 117
3 Receiver Gain 60
4 Relaxation Delay 0.0100
5 Spectrometer Frequency 160.38
6 Nucleus 11B
52.32
Me
TMS
N
B
nPr
Mes
f1 (ppm)

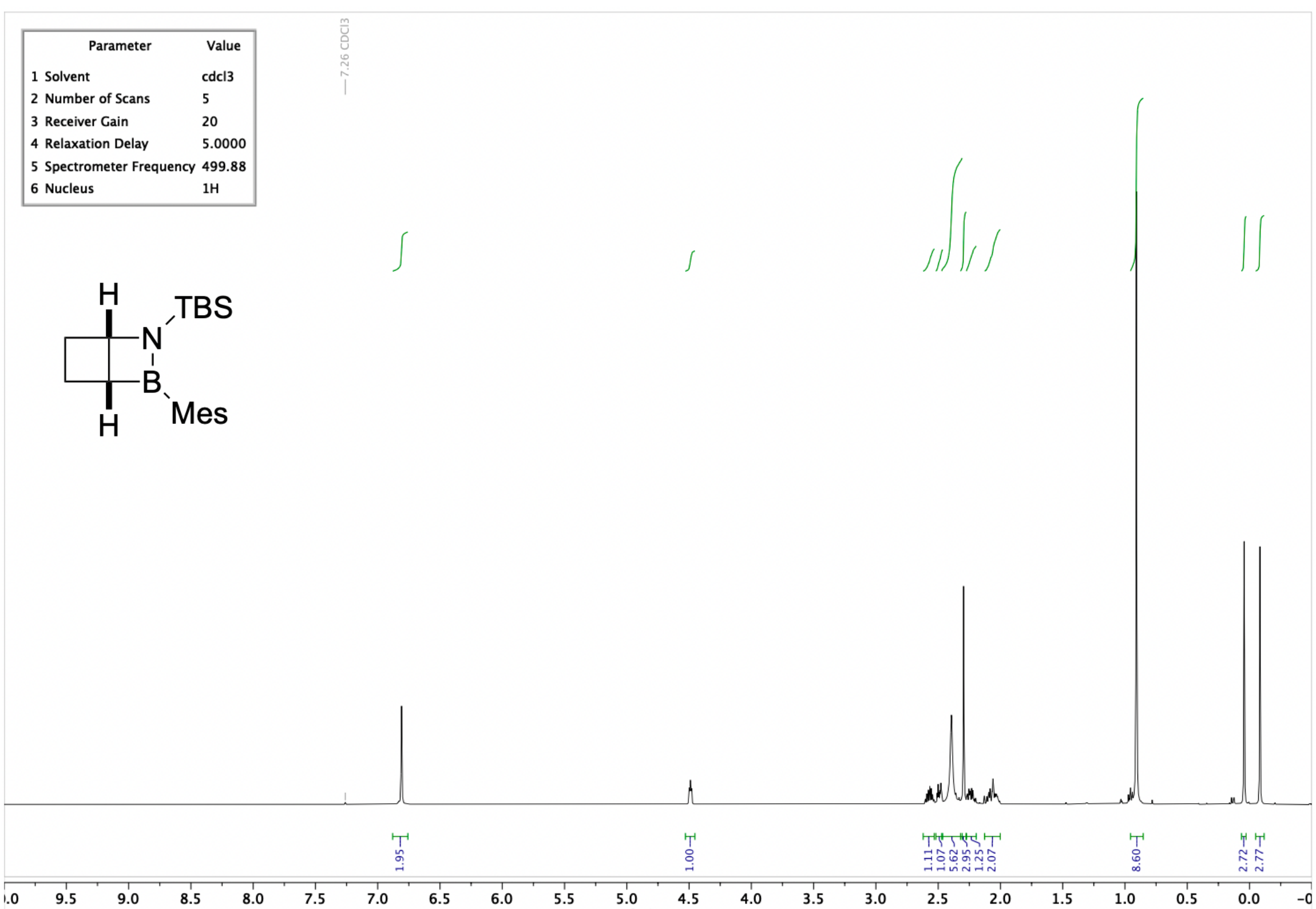

Parameter Value
1 Solvent cdcl3
2 Number of Scans 5
3 Receiver Gain 20
4 Relaxation Delay 5.0000
5 Spectrometer Frequency 499.88
6 Nucleus 1H
7.26 CDCl3
H
TBS
N
B
H
Mes
1.95
1.00
1.11
1.07
5.62
2.95
1.25
2.07
8.60
2.72
2.77
f1 (ppm)

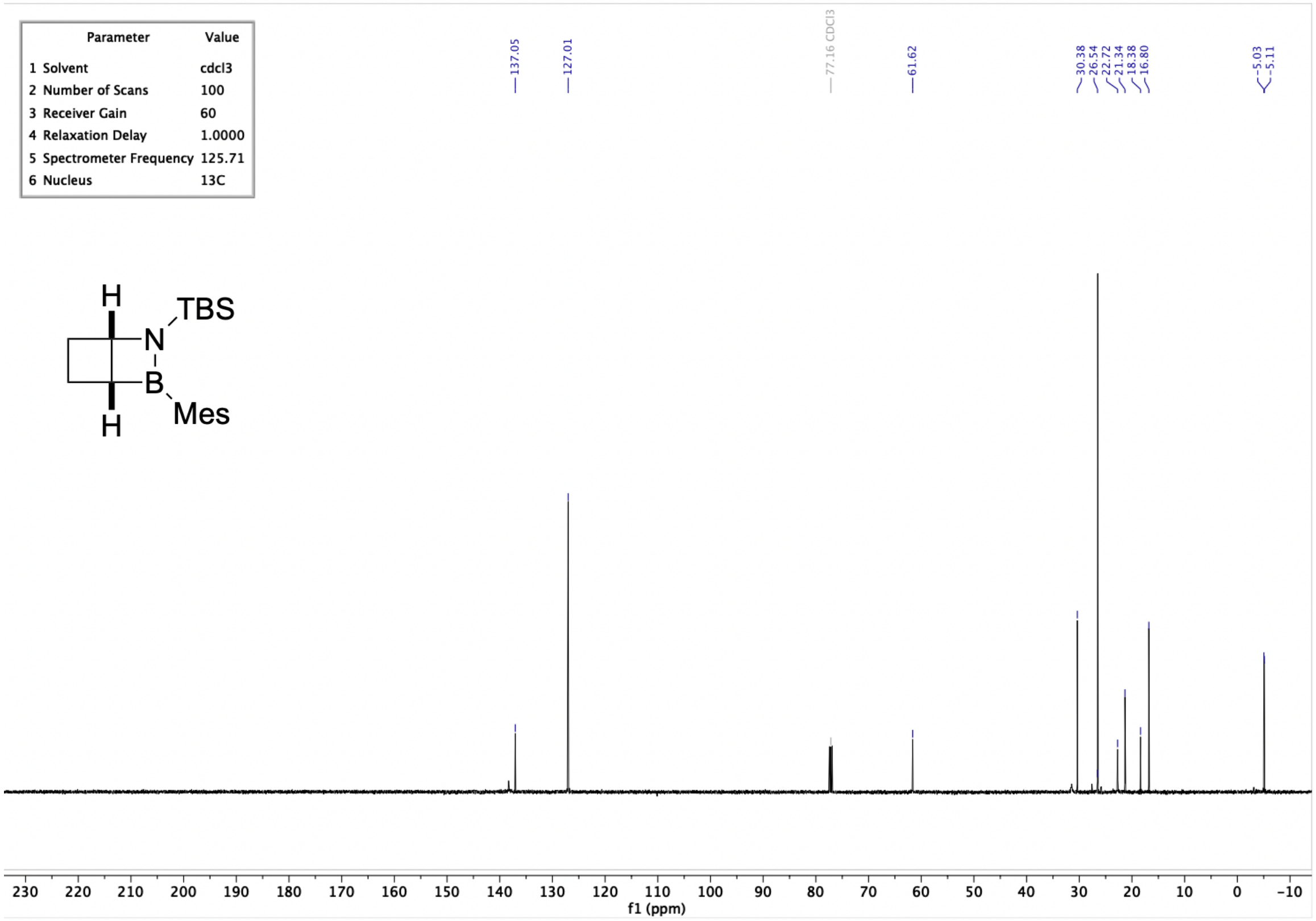
Parameter Value
1 Solvent cdcl3
2 Number of Scans 100
3 Receiver Gain 60
4 Relaxation Delay 1.0000
5 Spectrometer Frequency 125.71
6 Nucleus 13C
137.05
127.01
77.16 CDCl3
61.62
30.38
26.54
22.72
21.34
18.38
16.80
-5.03
-5.11
H
TBS
N
B
Mes
H
f1 (ppm)

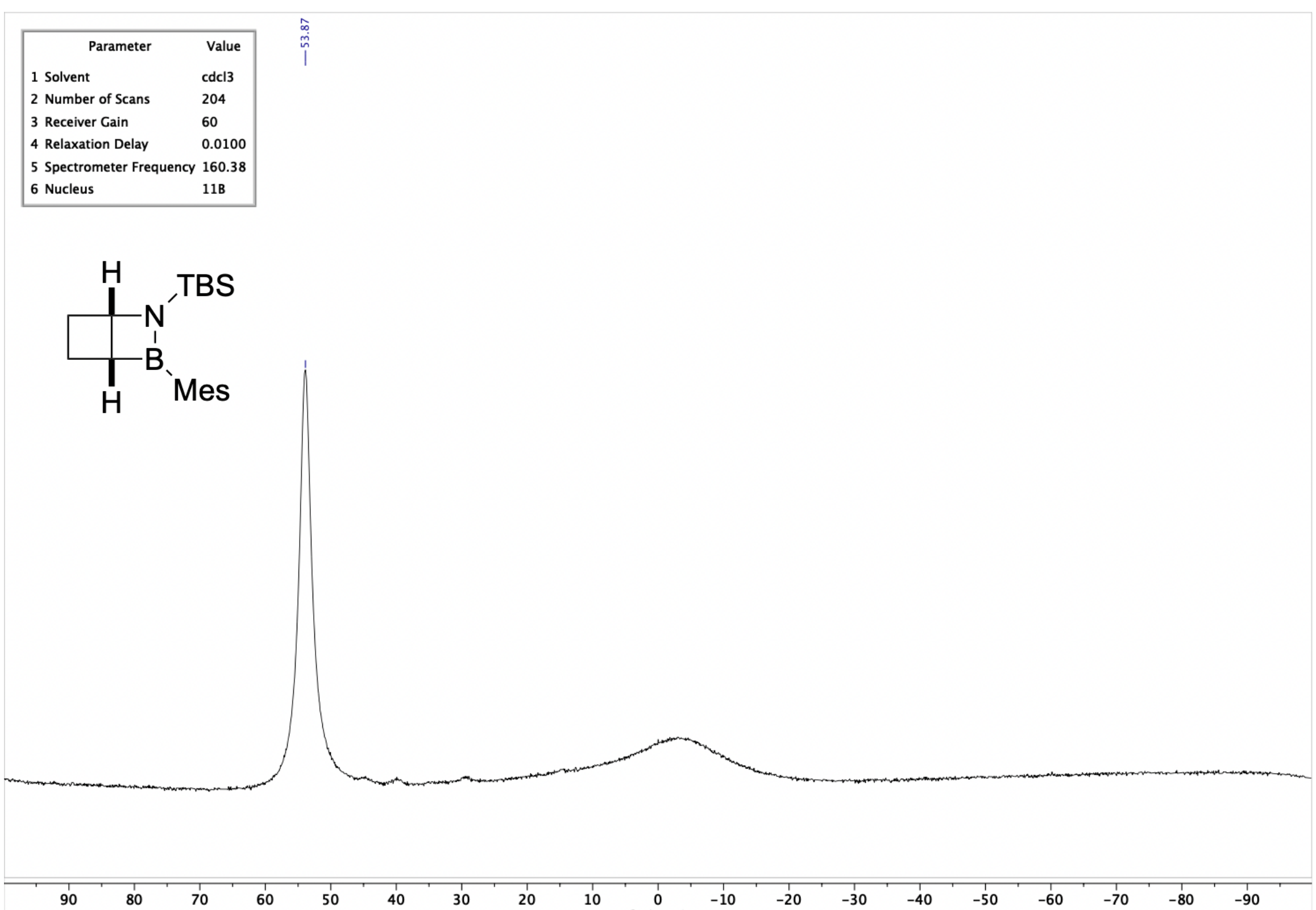
Parameter Value
1 Solvent cdcl3
2 Number of Scans 204
3 Receiver Gain 60
4 Relaxation Delay 0.0100
5 Spectrometer Frequency 160.38
6 Nucleus 11B
53.87
H
TBS
N
B
Mes
H
f1 (ppm)

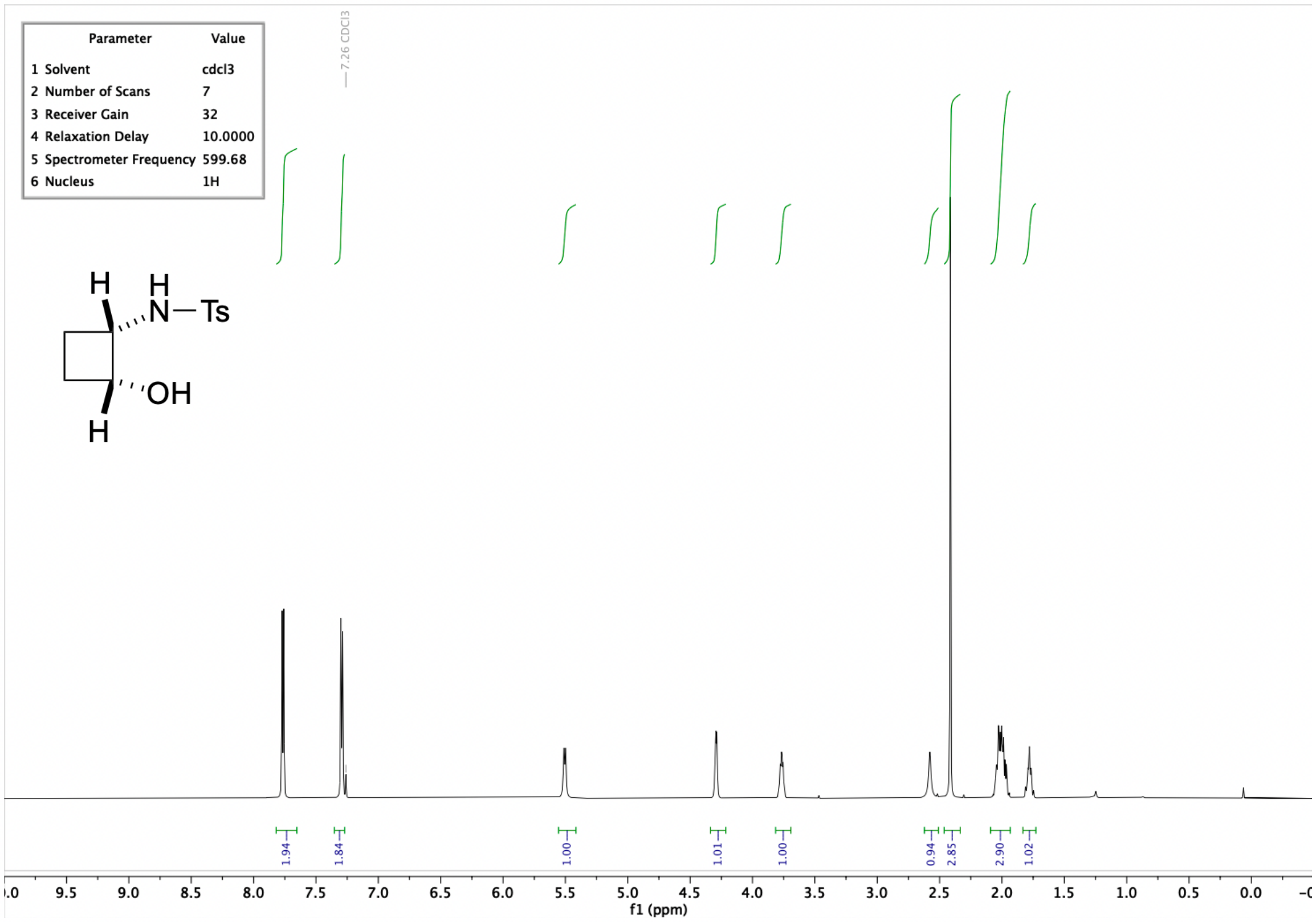
Parameter Value
1 Solvent cdcl3
2 Number of Scans 7
3 Receiver Gain 32
4 Relaxation Delay 10.0000
5 Spectrometer Frequency 599.68
6 Nucleus 1H
7.26 CDCl3
f1 (ppm)

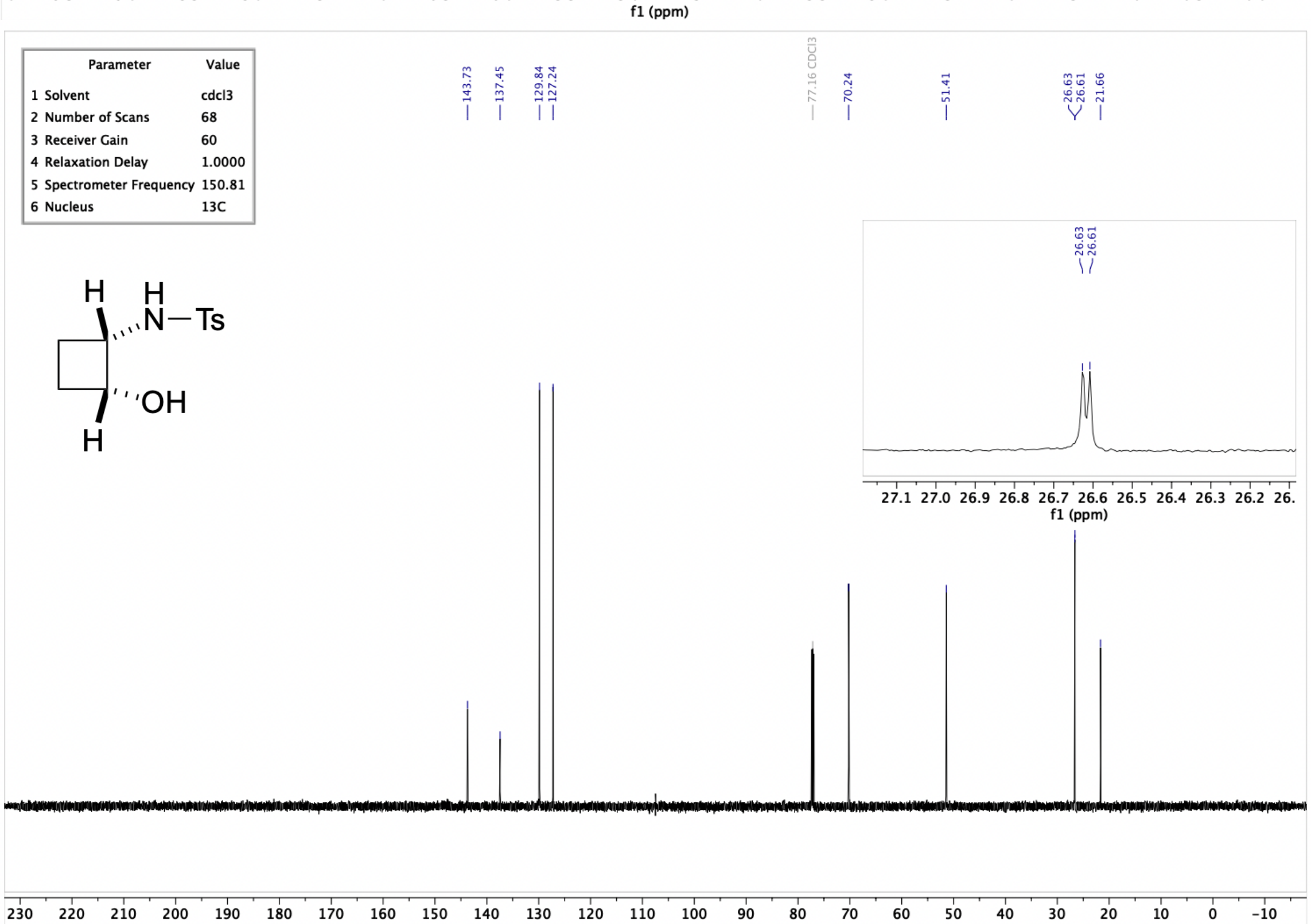
Parameter Value
1 Solvent cdcl3
2 Number of Scans 68
3 Receiver Gain 60
4 Relaxation Delay 1.0000
5 Spectrometer Frequency 150.81
6 Nucleus 13C
143.73
137.45
129.84
127.24
77.16 CDCl3
70.24
51.41
26.63
26.61
21.66
f1 (ppm)

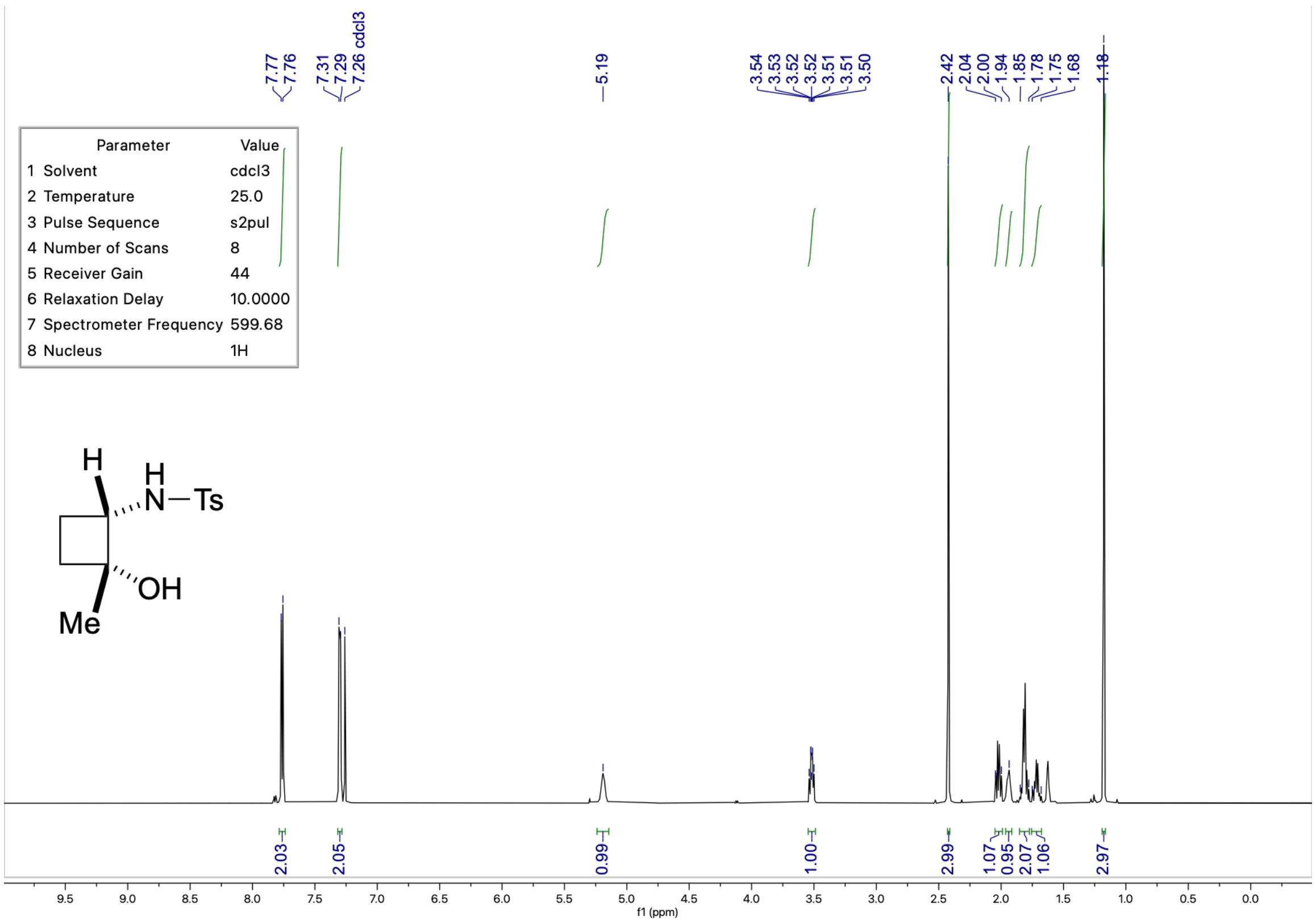
7.77
7.76
7.31
7.29
7.26 cdcl3
5.19
3.54
3.53
3.52
3.52
3.51
3.51
3.50
2.42
2.04
2.00
1.94
1.85
1.78
1.75
1.68
1.18
Parameter Value
1 Solvent cdcl3
2 Temperature 25.0
3 Pulse Sequence s2pul
4 Number of Scans 8
5 Receiver Gain 44
6 Relaxation Delay 10.0000
7 Spectrometer Frequency 599.68
8 Nucleus 1H
H
H
N—Ts
OH
Me
2.03
2.05
0.99
1.00
2.99
1.07
0.95
2.07
1.06
2.97
f1 (ppm)

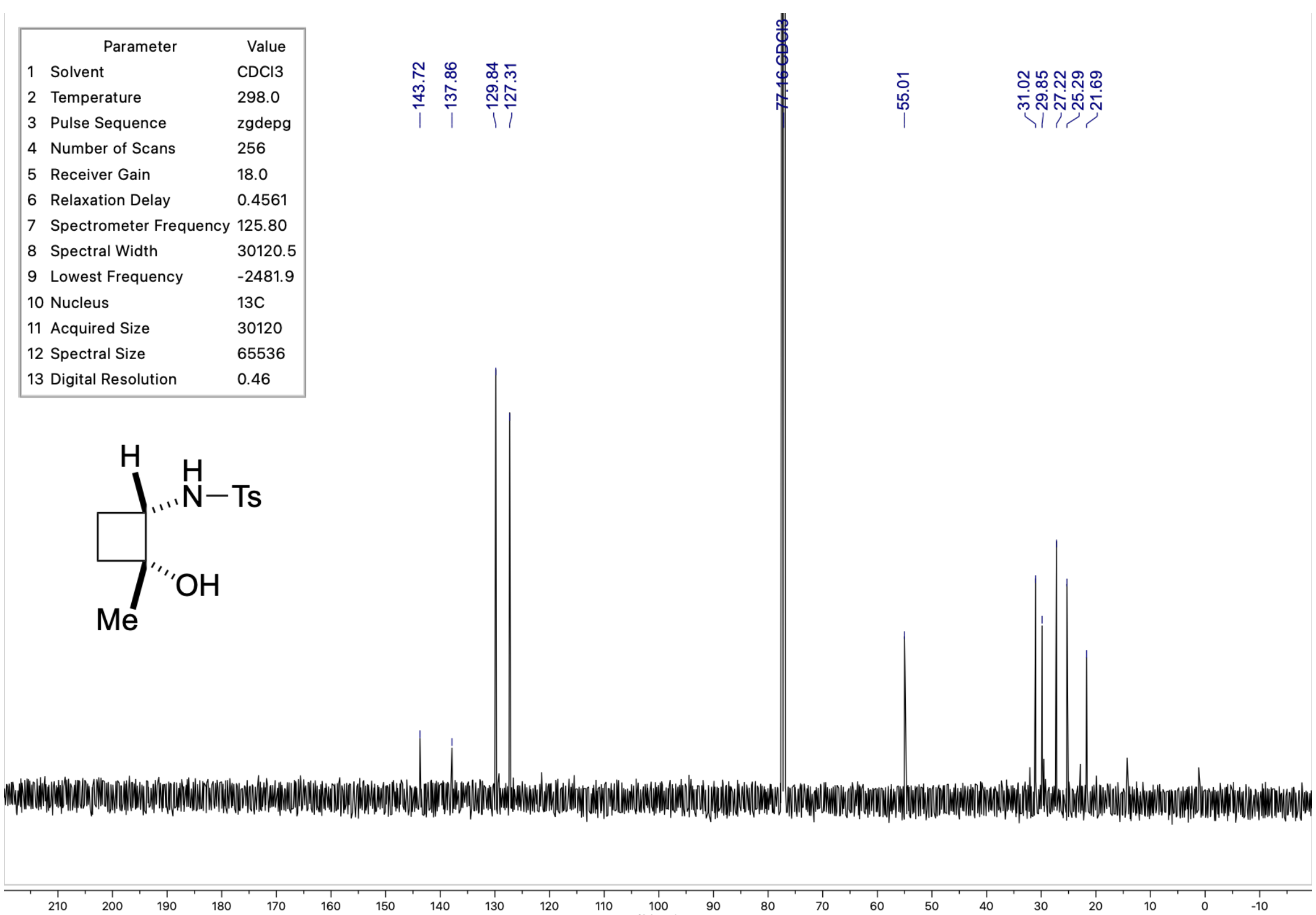
Parameter Value
1 Solvent CDCl3
2 Temperature 298.0
3 Pulse Sequence zgdepg
4 Number of Scans 256
5 Receiver Gain 18.0
6 Relaxation Delay 0.4561
7 Spectrometer Frequency 125.80
8 Spectral Width 30120.5
9 Lowest Frequency -2481.9
10 Nucleus 13C
11 Acquired Size 30120
12 Spectral Size 65536
13 Digital Resolution 0.46
143.72
137.86
129.84
127.31
77.16 CDCl3
55.01
31.02
29.85
27.22
25.29
21.69
H
H
N—Ts
OH
Me
f1 (ppm)

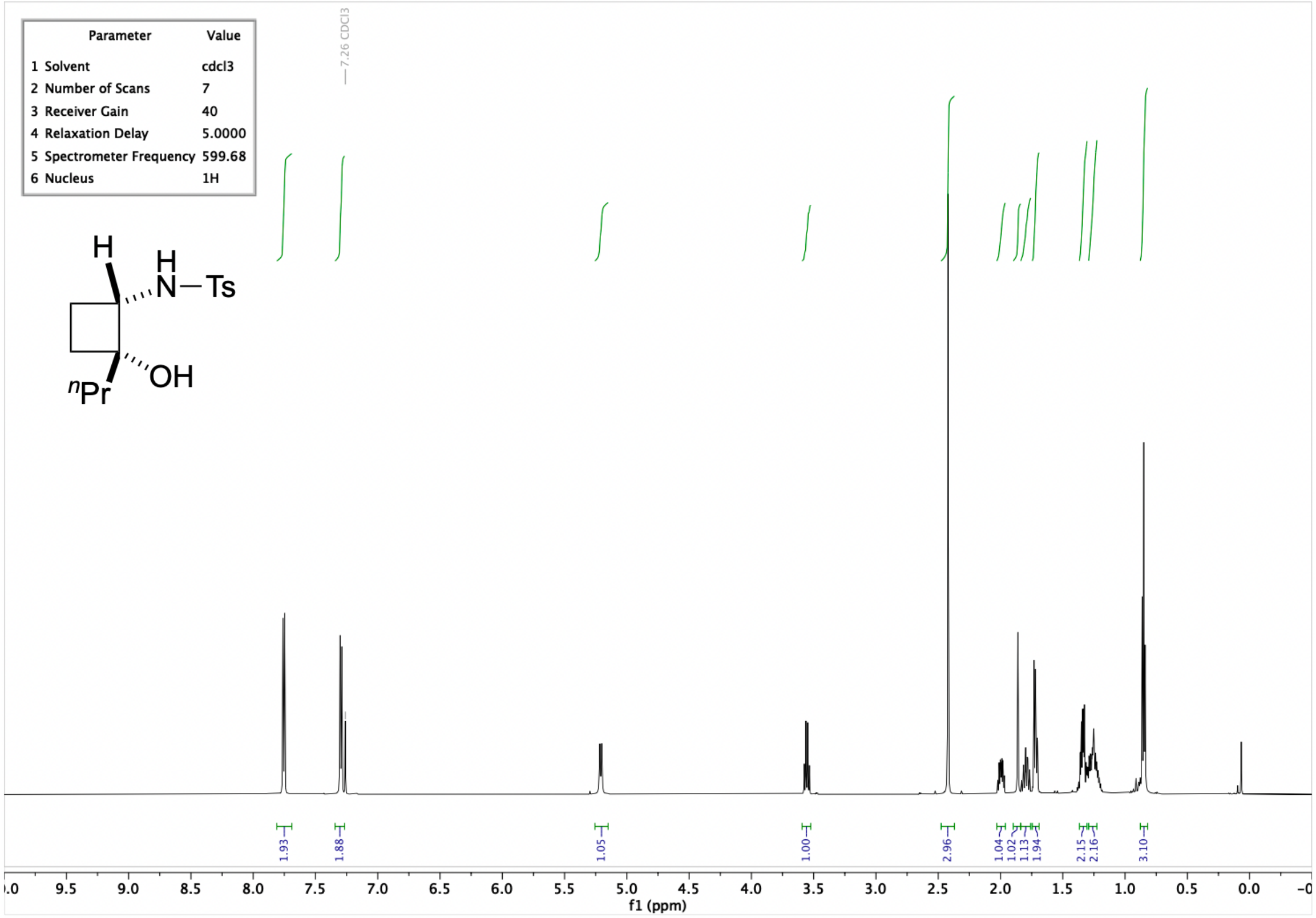

Parameter Value
1 Solvent cdcl3
2 Number of Scans 7
3 Receiver Gain 40
4 Relaxation Delay 5.0000
5 Spectrometer Frequency 599.68
6 Nucleus 1H
7.26 CDCl3
1.93
1.88
1.05
1.00
2.96
1.04
1.02
1.13
1.94
2.15
2.16
3.10
f1 (ppm)

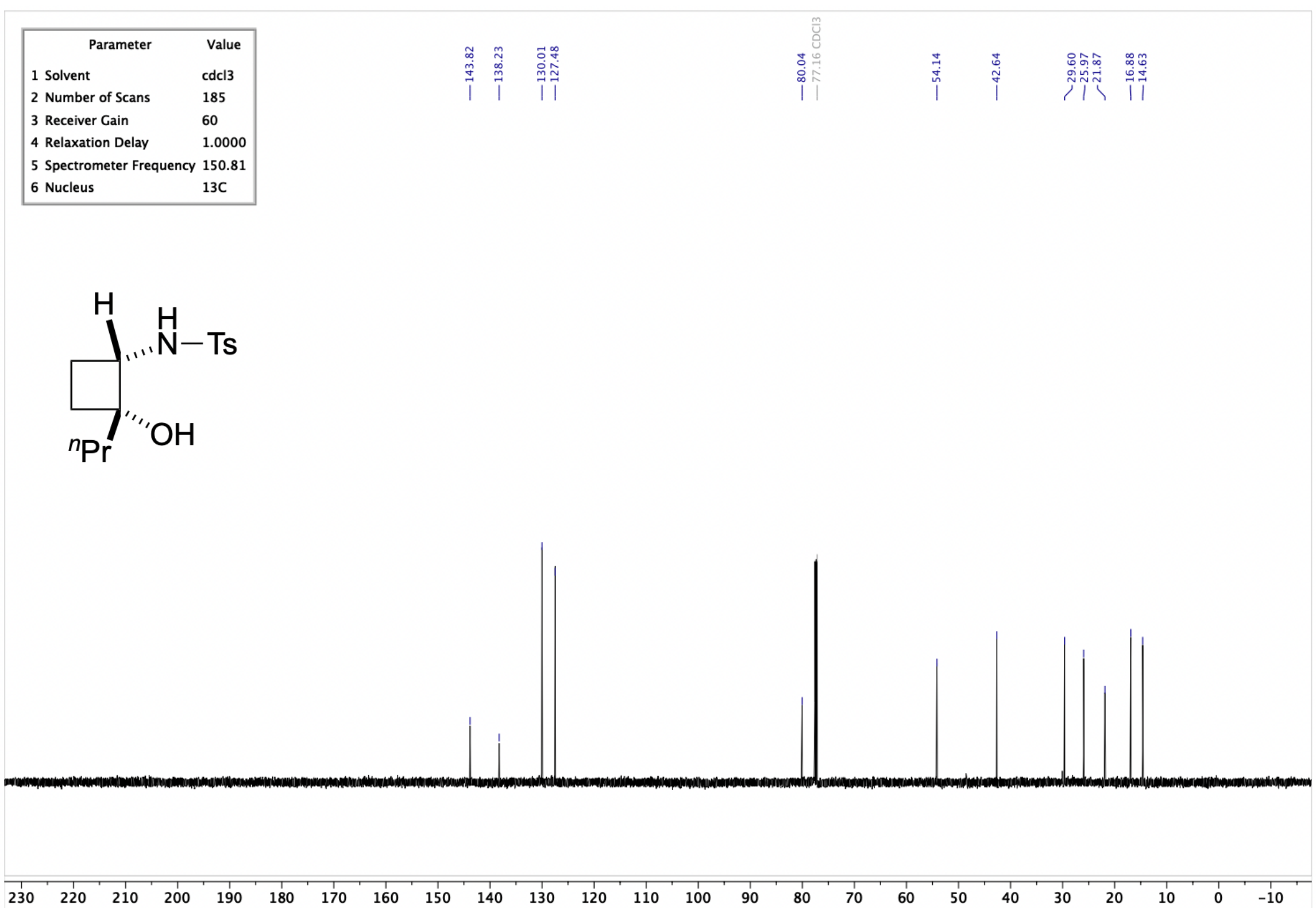

Parameter Value
1 Solvent cdcl3
2 Number of Scans 185
3 Receiver Gain 60
4 Relaxation Delay 1.0000
5 Spectrometer Frequency 150.81
6 Nucleus 13C
143.82
138.23
130.01
127.48
80.04
77.16 CDCl3
54.14
42.64
29.60
25.97
21.87
16.88
14.63
f1 (ppm)

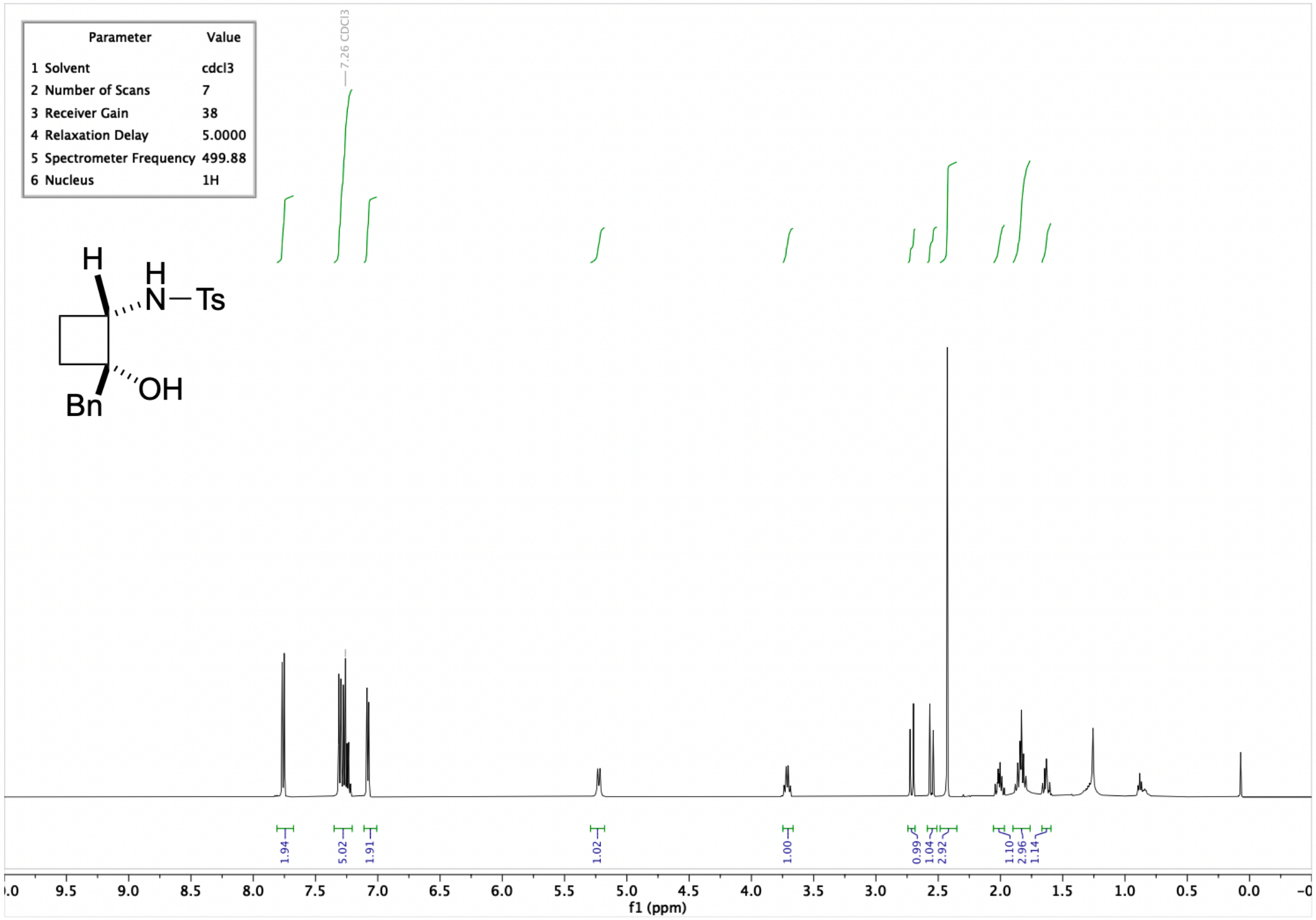

Parameter Value
1 Solvent cdcl3
2 Number of Scans 7
3 Receiver Gain 38
4 Relaxation Delay 5.0000
5 Spectrometer Frequency 499.88
6 Nucleus 1H
7.26 CDCl3
H
H
N—Ts
OH
Bn
1.94
5.02
1.91
1.02
1.00
0.99
1.04
2.92
1.10
2.96
1.14
f1 (ppm)

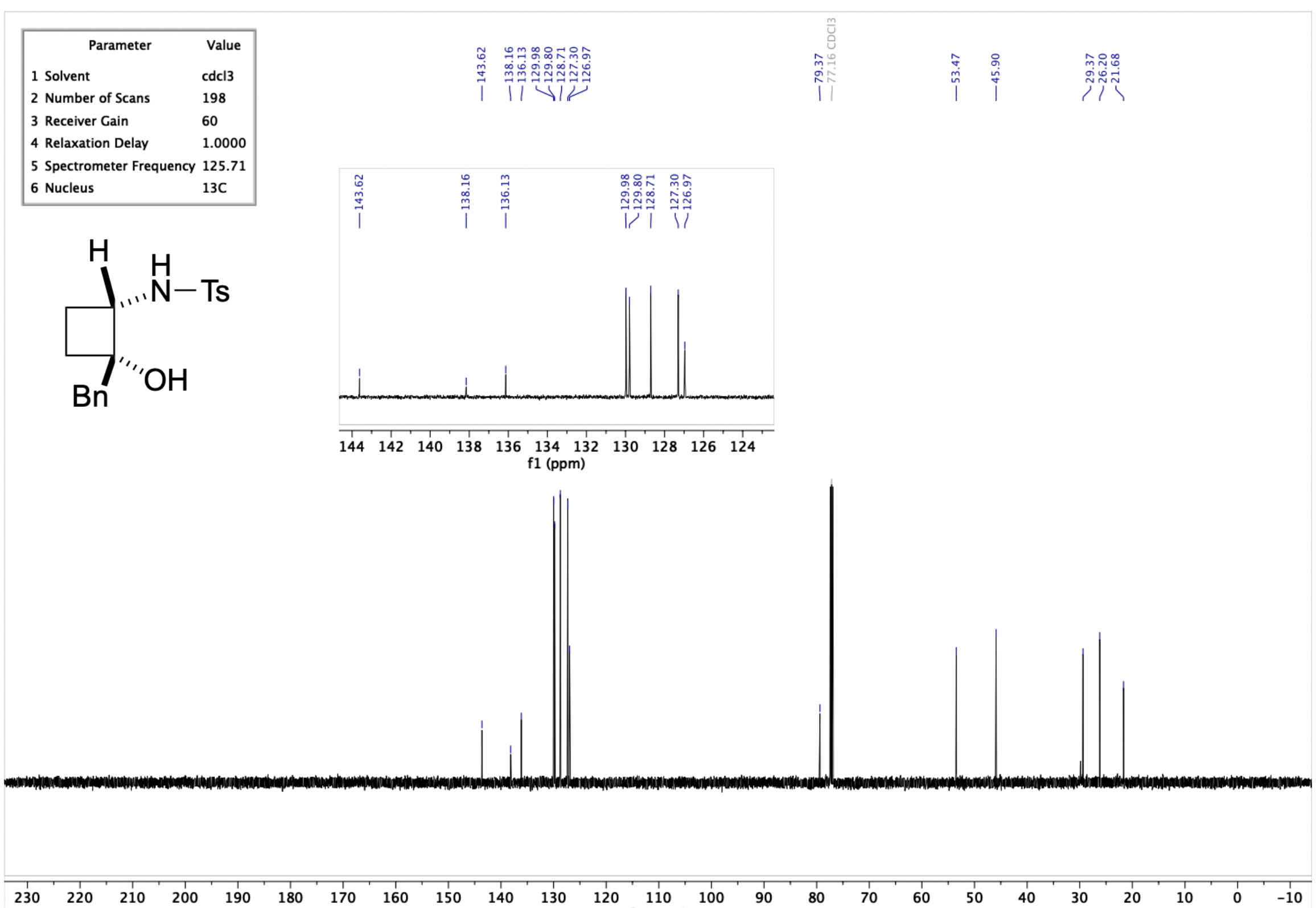

Parameter Value
1 Solvent cdcl3
2 Number of Scans 198
3 Receiver Gain 60
4 Relaxation Delay 1.0000
5 Spectrometer Frequency 125.71
6 Nucleus 13C
143.62
138.16
136.13
129.98
129.80
128.71
127.30
126.97
79.37
77.16 CDCl3
53.47
45.90
29.37
26.20
21.68
H
H
N—Ts
OH
Bn
f1 (ppm)

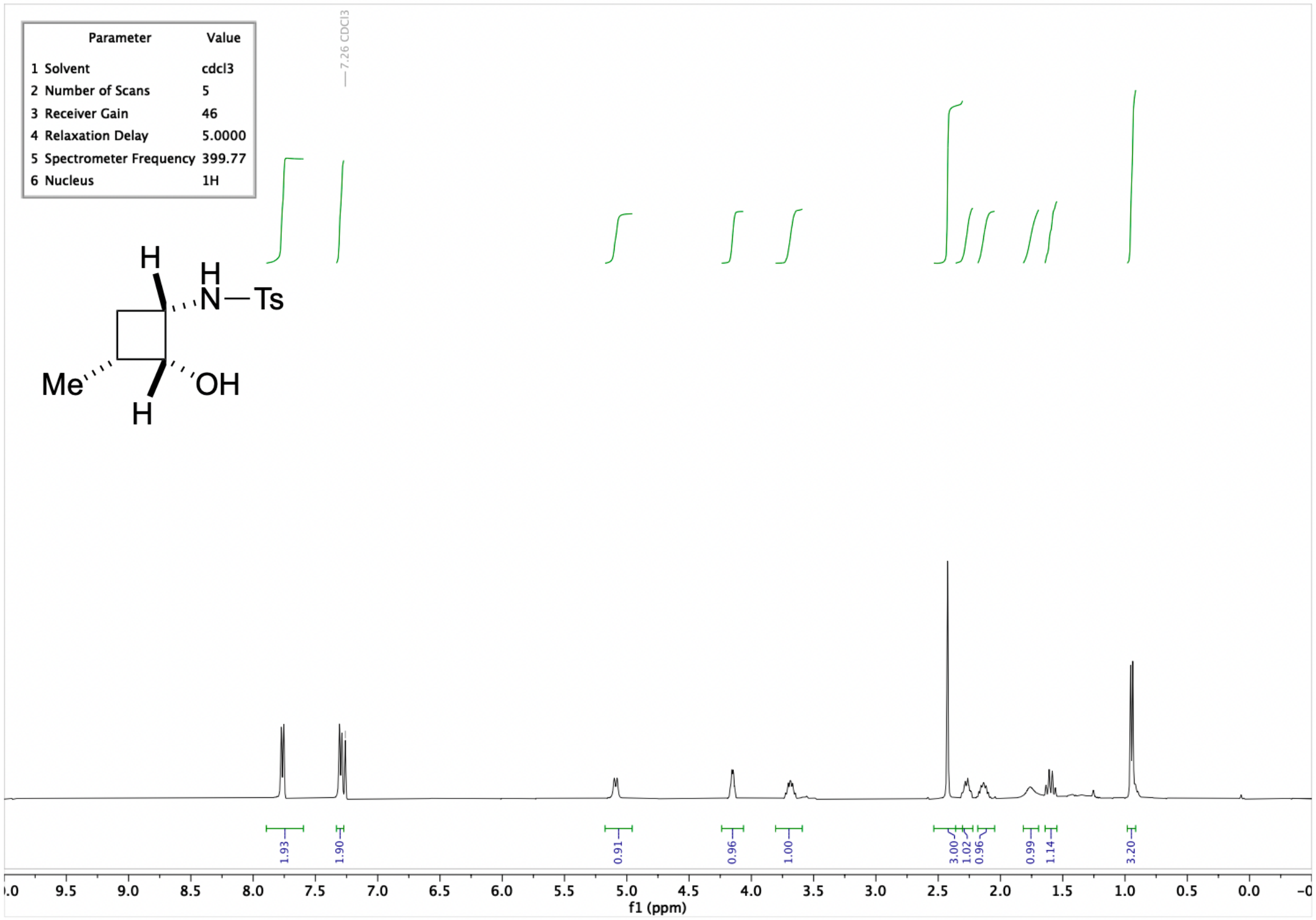

Parameter Value
1 Solvent cdcl3
2 Number of Scans 5
3 Receiver Gain 46
4 Relaxation Delay 5.0000
5 Spectrometer Frequency 399.77
6 Nucleus 1H
7.26 CDCl3
H
H
N—Ts
Me
OH
H
1.93
1.90
0.91
0.96
1.00
3.00
1.02
0.96
0.99
1.14
3.20
f1 (ppm)

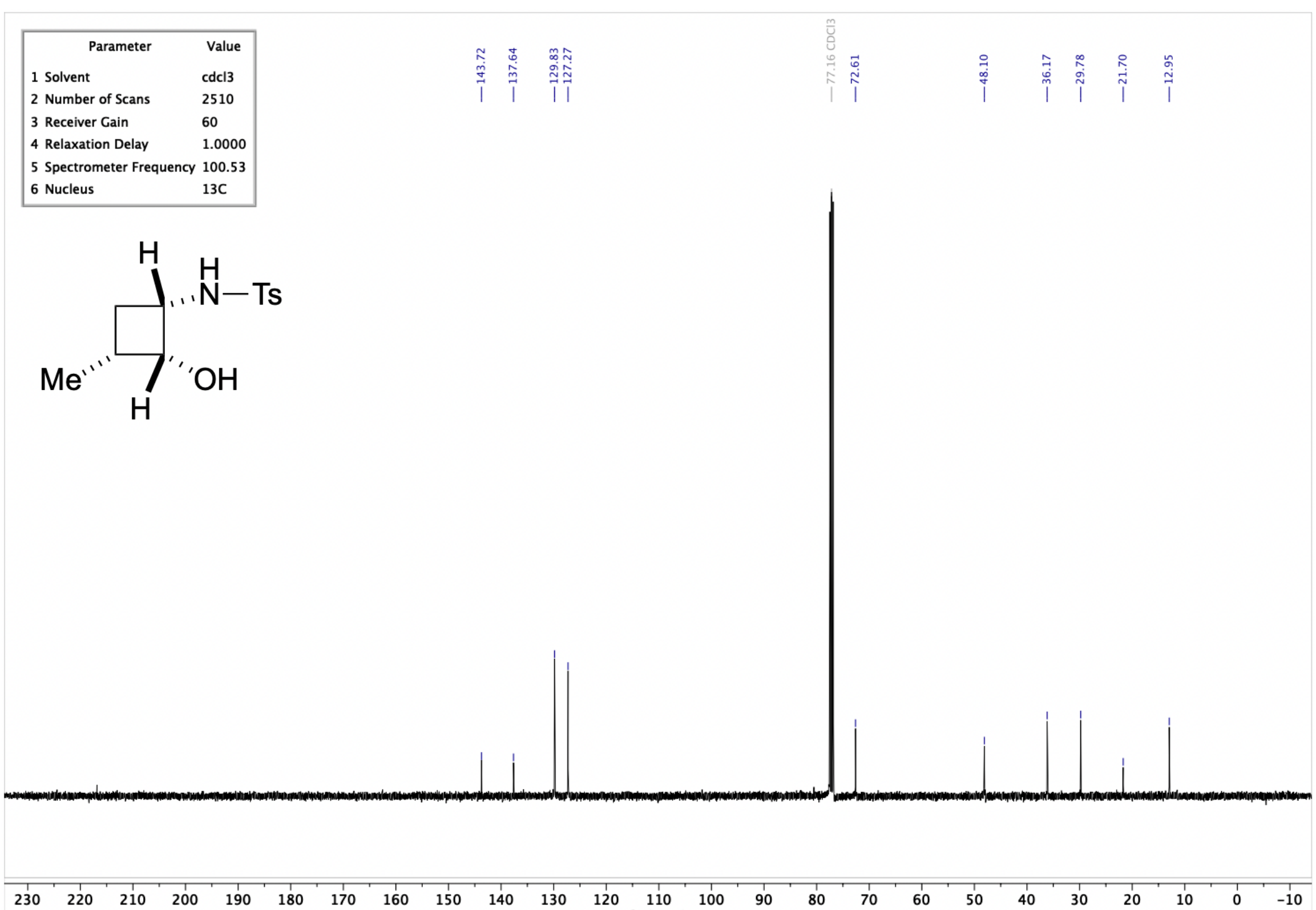

Parameter Value
1 Solvent cdcl3
2 Number of Scans 2510
3 Receiver Gain 60
4 Relaxation Delay 1.0000
5 Spectrometer Frequency 100.53
6 Nucleus 13C
143.72
137.64
129.83
127.27
77.16 CDCl3
72.61
48.10
36.17
29.78
21.70
12.95
H
H
N—Ts
Me
OH
H
f1 (ppm)

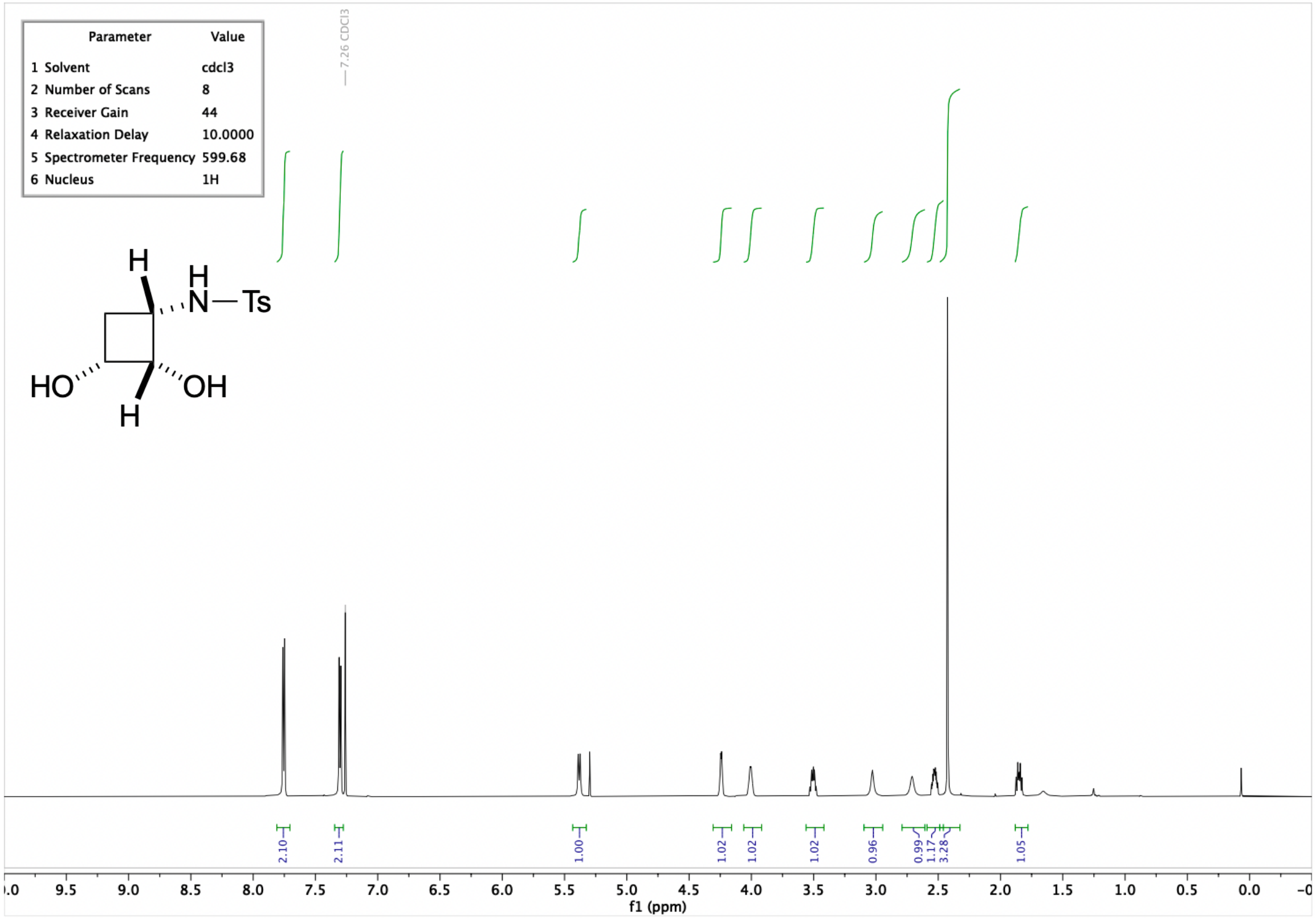
Parameter Value
1 Solvent cdcl3
2 Number of Scans 8
3 Receiver Gain 44
4 Relaxation Delay 10.0000
5 Spectrometer Frequency 599.68
6 Nucleus 1H
7.26 CDCl3
f1 (ppm)

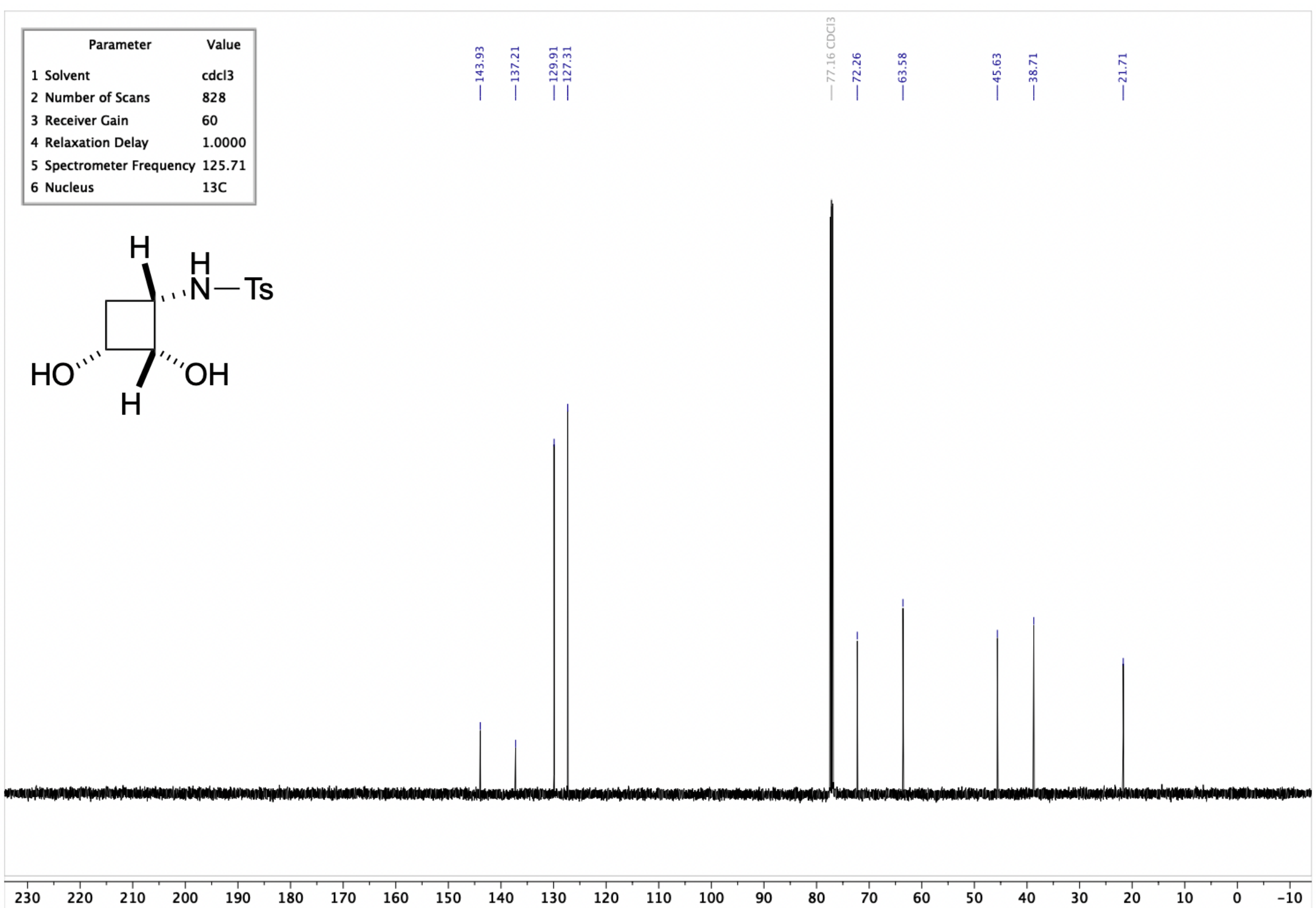
Parameter Value
1 Solvent cdcl3
2 Number of Scans 828
3 Receiver Gain 60
4 Relaxation Delay 1.0000
5 Spectrometer Frequency 125.71
6 Nucleus 13C
143.93
137.21
129.91
127.31
77.16 CDCl3
72.26
63.58
45.63
38.71
21.71
f1 (ppm)

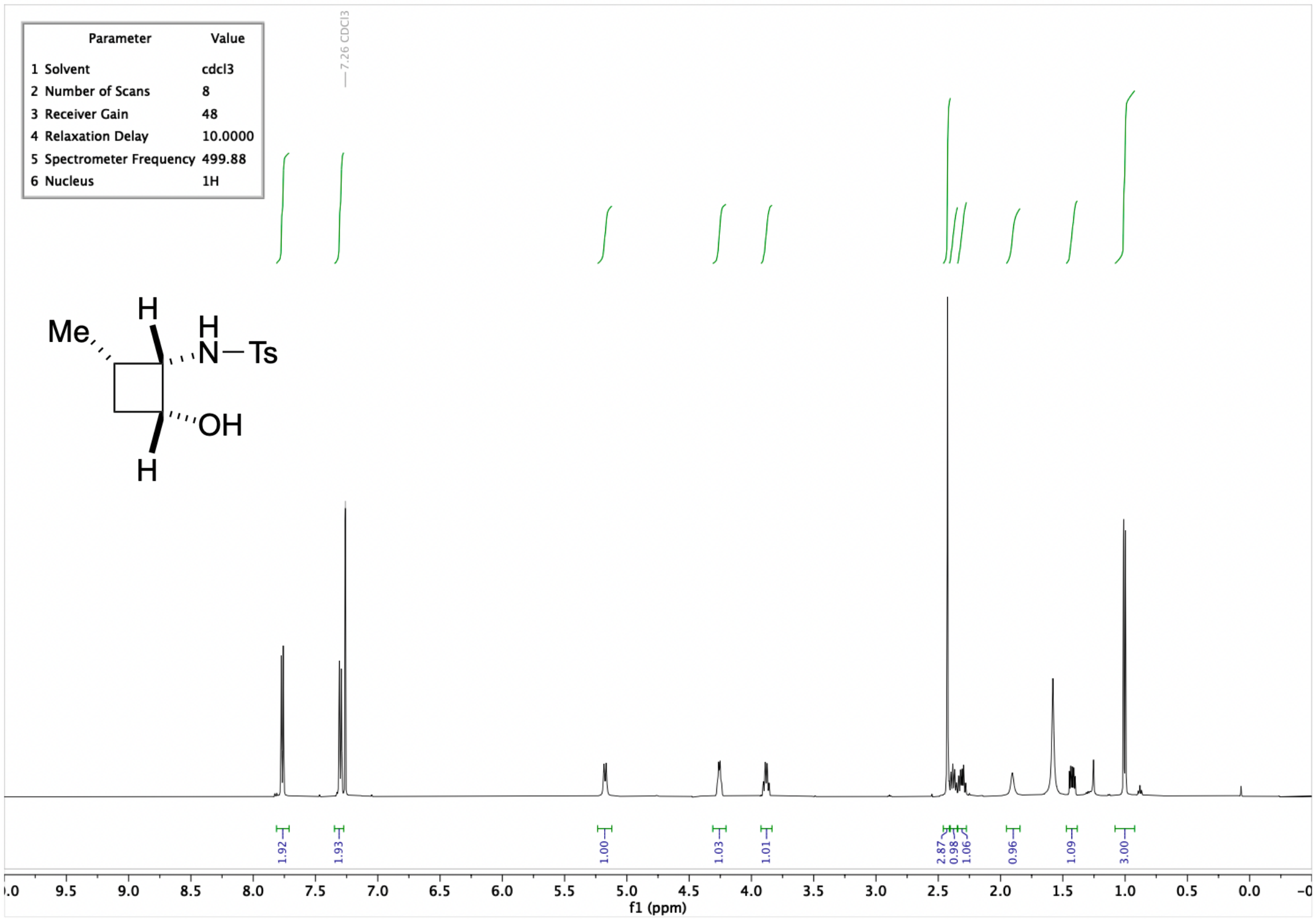
Parameter Value
1 Solvent cdcl3
2 Number of Scans 8
3 Receiver Gain 48
4 Relaxation Delay 10.0000
5 Spectrometer Frequency 499.88
6 Nucleus 1H
7.26 CDCl3
Me
H
H
N−Ts
OH
H
1.92
1.93
1.00
1.03
1.01
2.87
0.98
1.06
0.96
1.09
3.00
f1 (ppm)

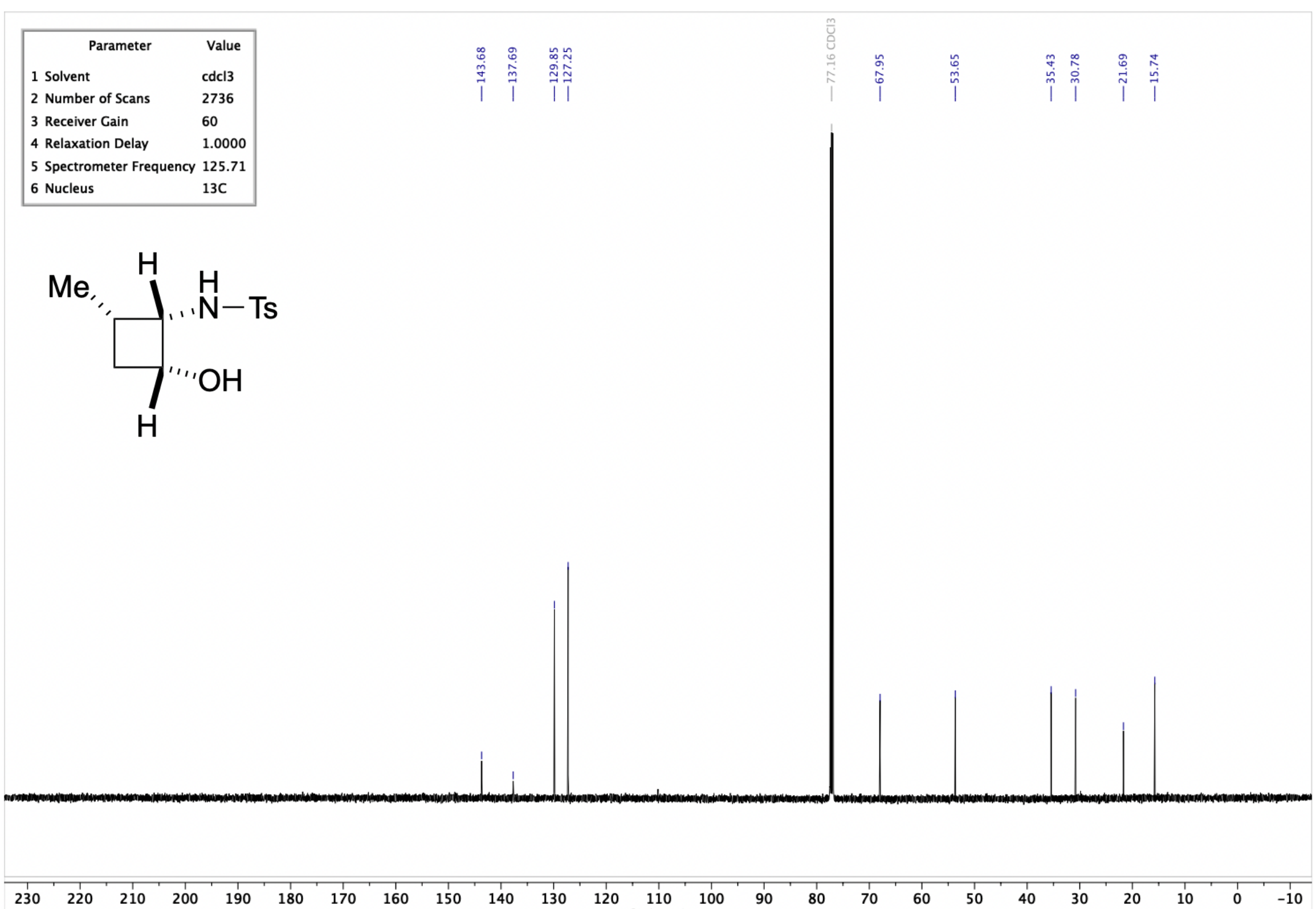
Parameter Value
1 Solvent cdcl3
2 Number of Scans 2736
3 Receiver Gain 60
4 Relaxation Delay 1.0000
5 Spectrometer Frequency 125.71
6 Nucleus 13C
143.68
137.69
129.85
127.25
77.16 CDCl3
67.95
53.65
35.43
30.78
21.69
15.74
Me
H
H
N−Ts
OH
H
f1 (ppm)

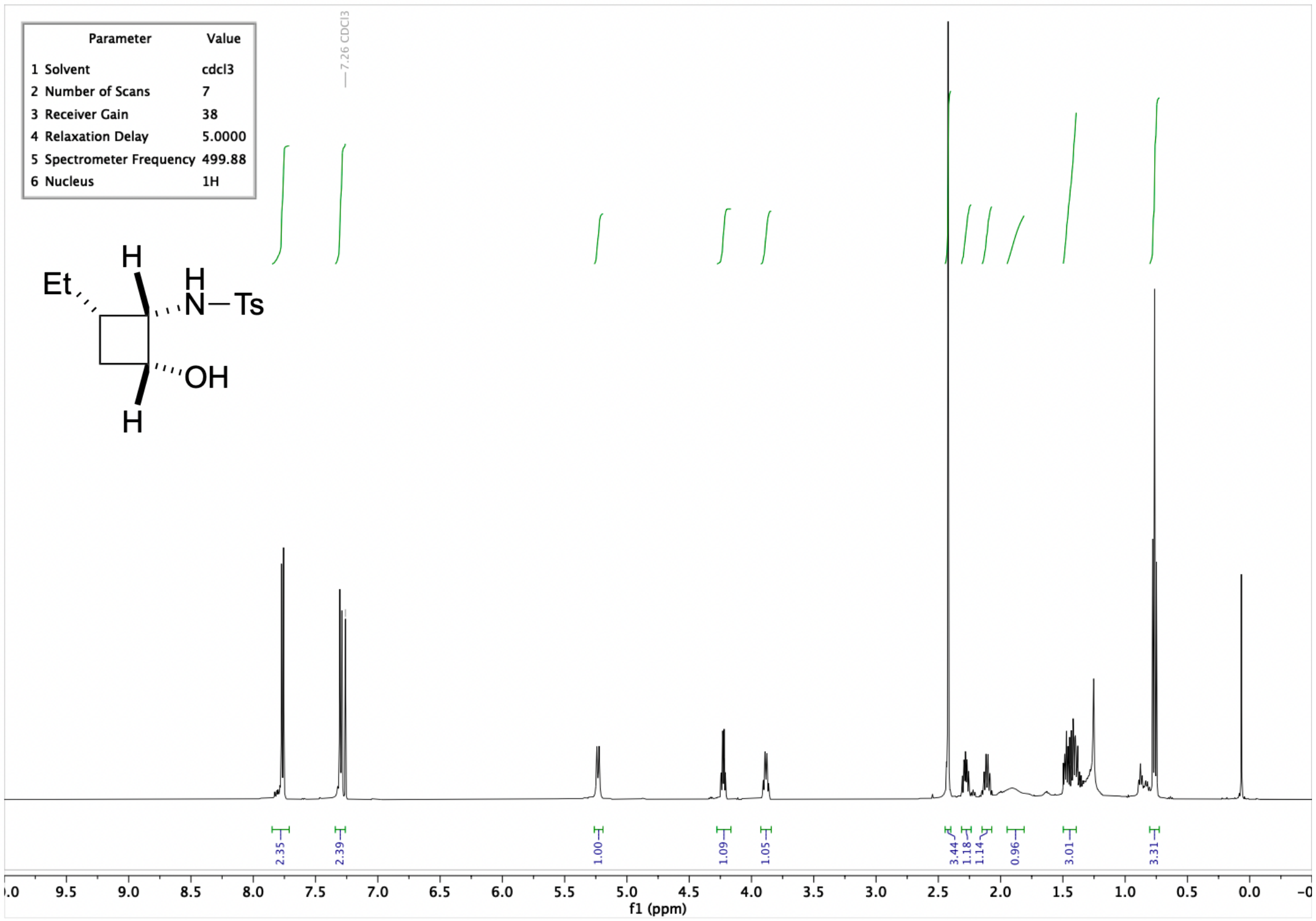
Parameter Value
1 Solvent cdcl3
2 Number of Scans 7
3 Receiver Gain 38
4 Relaxation Delay 5.0000
5 Spectrometer Frequency 499.88
6 Nucleus 1H
Et
H
N—Ts
OH
f1 (ppm)

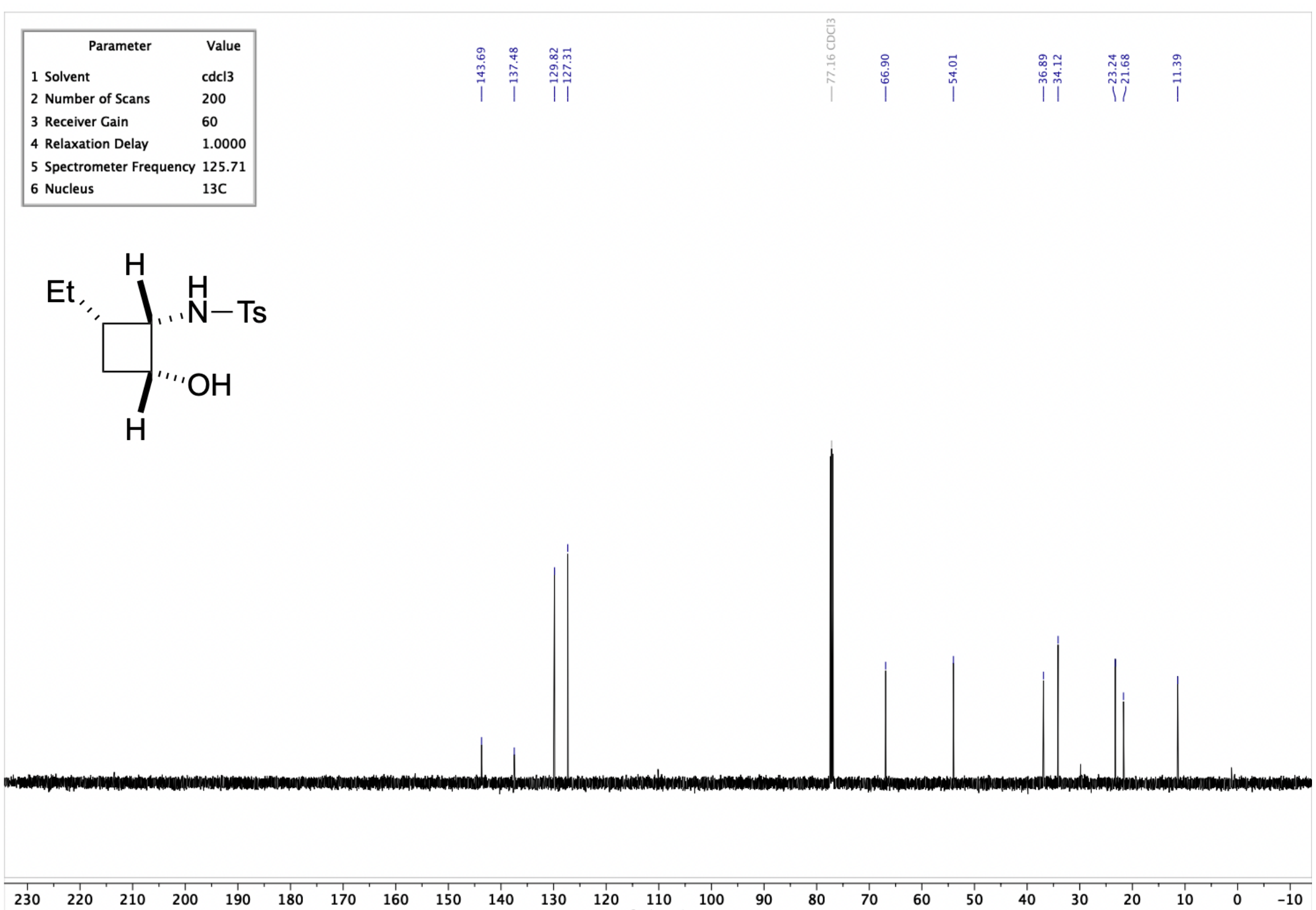
Parameter Value
1 Solvent cdcl3
2 Number of Scans 200
3 Receiver Gain 60
4 Relaxation Delay 1.0000
5 Spectrometer Frequency 125.71
6 Nucleus 13C
Et
H
N—Ts
OH
f1 (ppm)

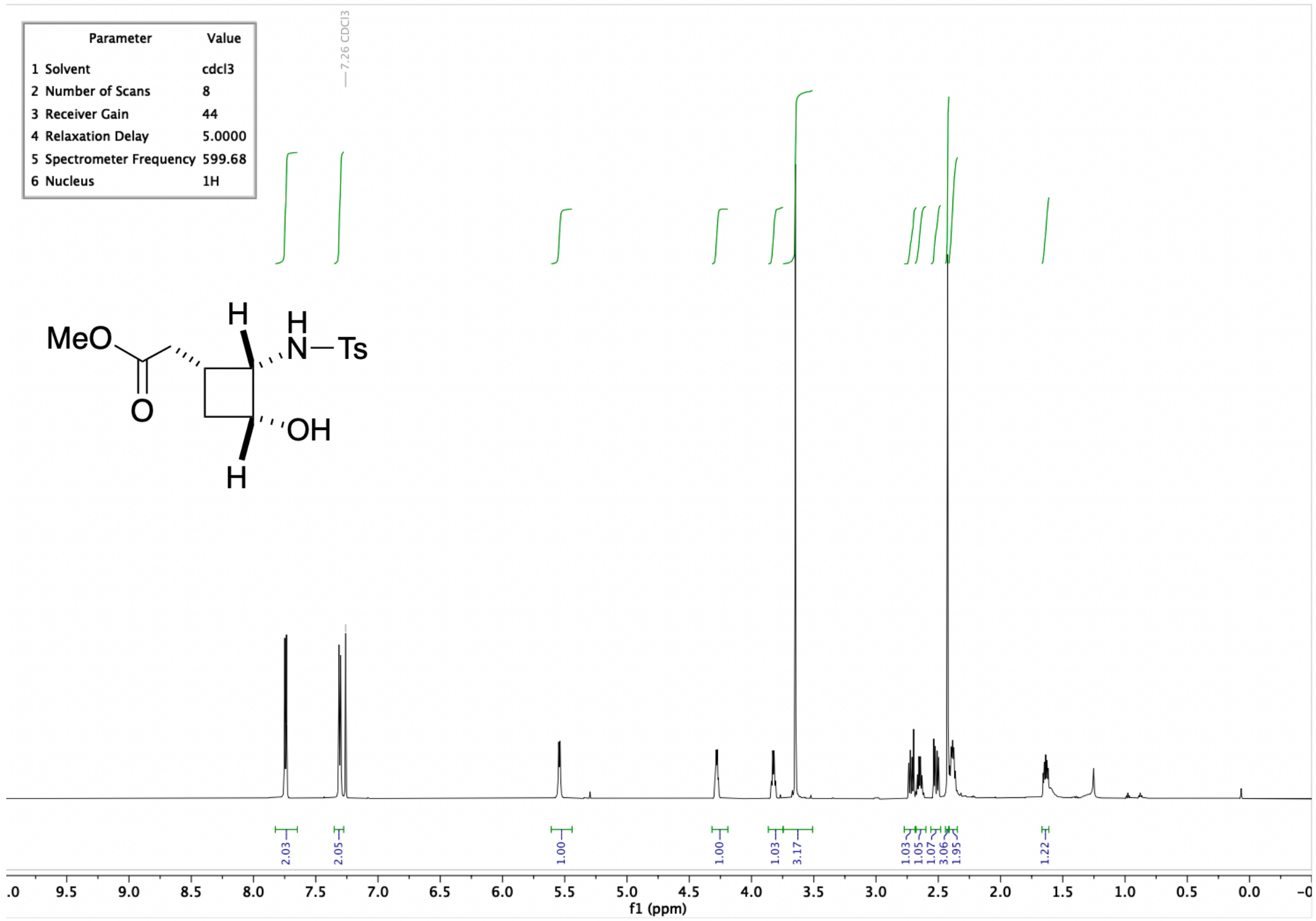

Parameter Value
1 Solvent cdcl3
2 Number of Scans 8
3 Receiver Gain 44
4 Relaxation Delay 5.0000
5 Spectrometer Frequency 599.68
6 Nucleus 1H
7.26 CDCl3
MeO
O
H
H
N—Ts
OH
H
2.03
2.05
1.00
1.00
1.03
3.17
1.03
1.05
1.07
3.06
1.95
1.22
f1 (ppm)

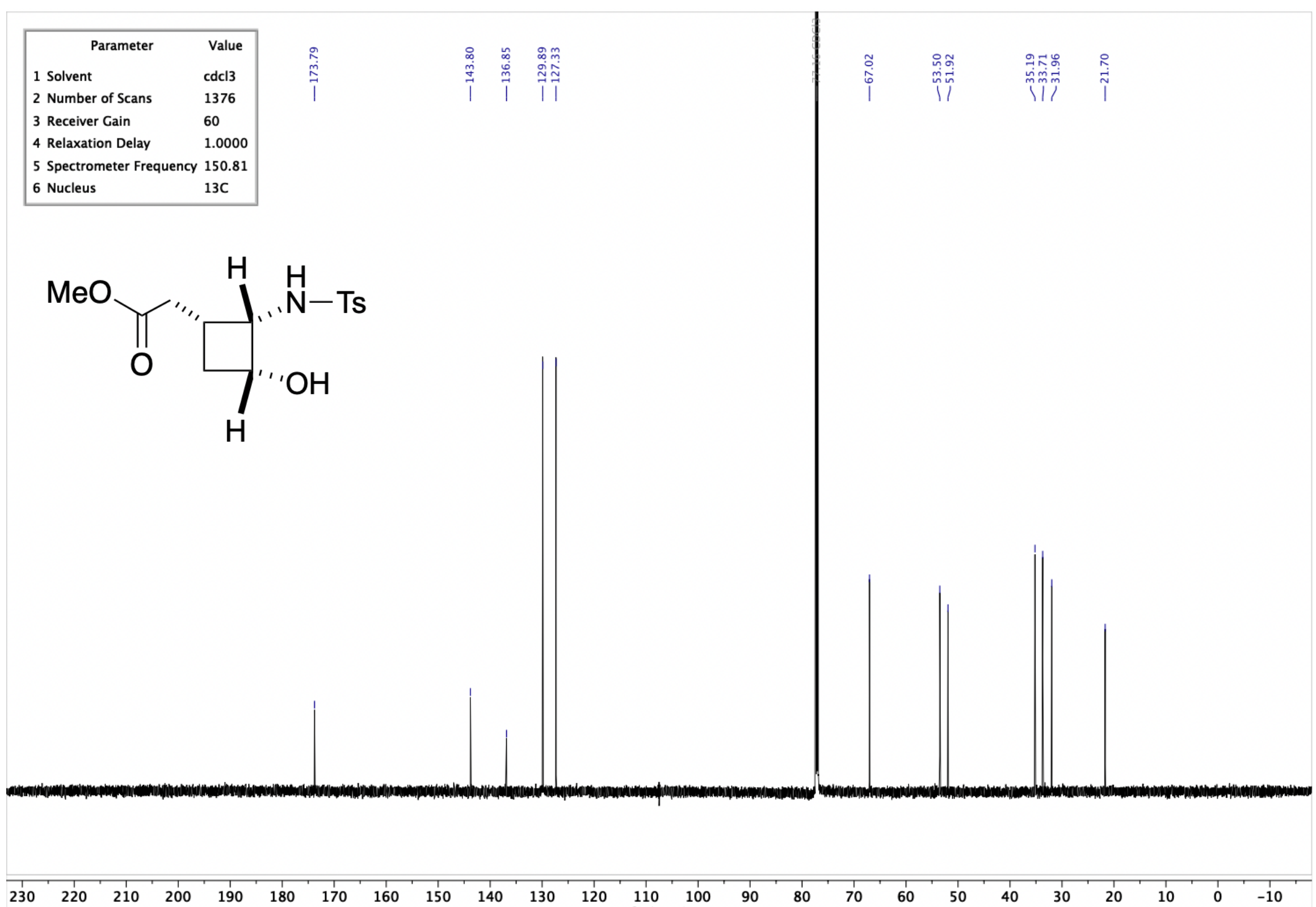

Parameter Value
1 Solvent cdcl3
2 Number of Scans 1376
3 Receiver Gain 60
4 Relaxation Delay 1.0000
5 Spectrometer Frequency 150.81
6 Nucleus 13C
173.79
143.80
136.85
129.89
127.33
67.02
53.50
51.92
35.19
33.71
31.96
21.70
MeO
O
H
H
N—Ts
OH
H
f1 (ppm)

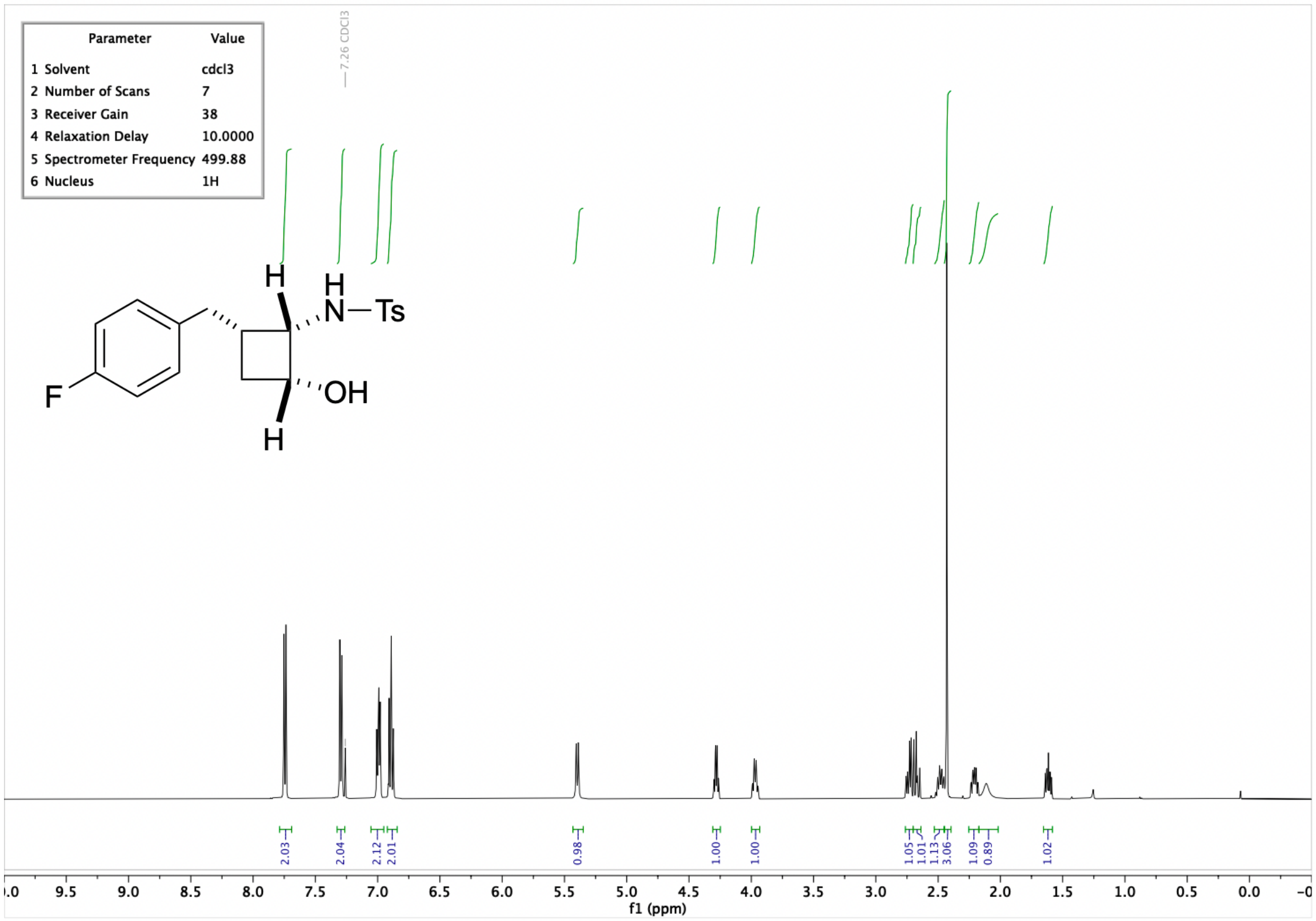

Parameter Value
1 Solvent cdcl3
2 Number of Scans 7
3 Receiver Gain 38
4 Relaxation Delay 10.0000
5 Spectrometer Frequency 499.88
6 Nucleus 1H
7.26 CDCl3
N—Ts
OH
F
f1 (ppm)

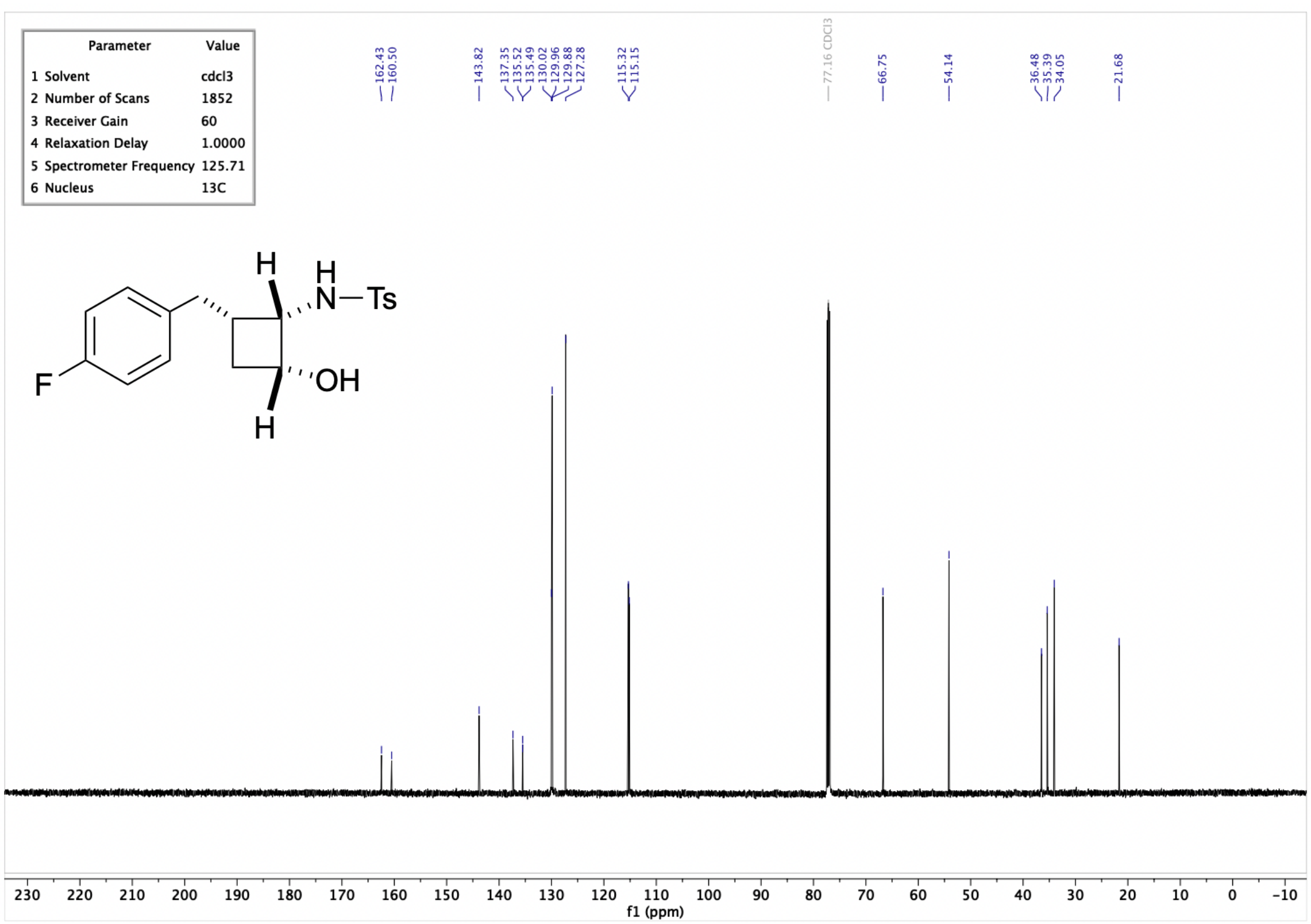

Parameter Value
1 Solvent cdcl3
2 Number of Scans 1852
3 Receiver Gain 60
4 Relaxation Delay 1.0000
5 Spectrometer Frequency 125.71
6 Nucleus 13C
162.43
160.50
143.82
137.35
135.52
135.49
130.02
129.96
129.88
127.28
115.32
115.15
77.16 CDCl3
66.75
54.14
36.48
35.39
34.05
21.68
N—Ts
OH
F
f1 (ppm)

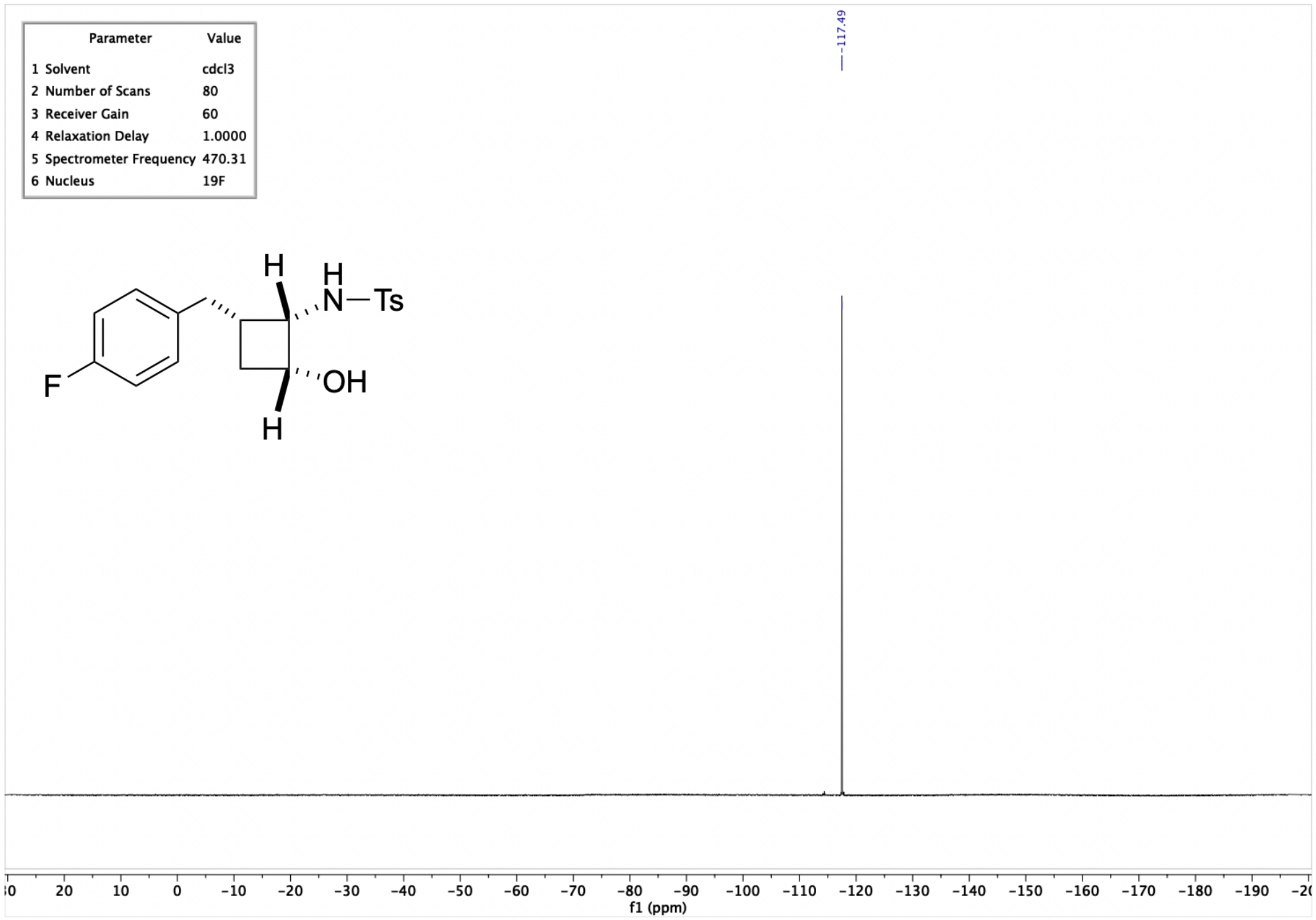

Parameter Value
1 Solvent cdcl3
2 Number of Scans 80
3 Receiver Gain 60
4 Relaxation Delay 1.0000
5 Spectrometer Frequency 470.31
6 Nucleus 19F
-117.49
F
H
H
N—Ts
OH
H
f1 (ppm)

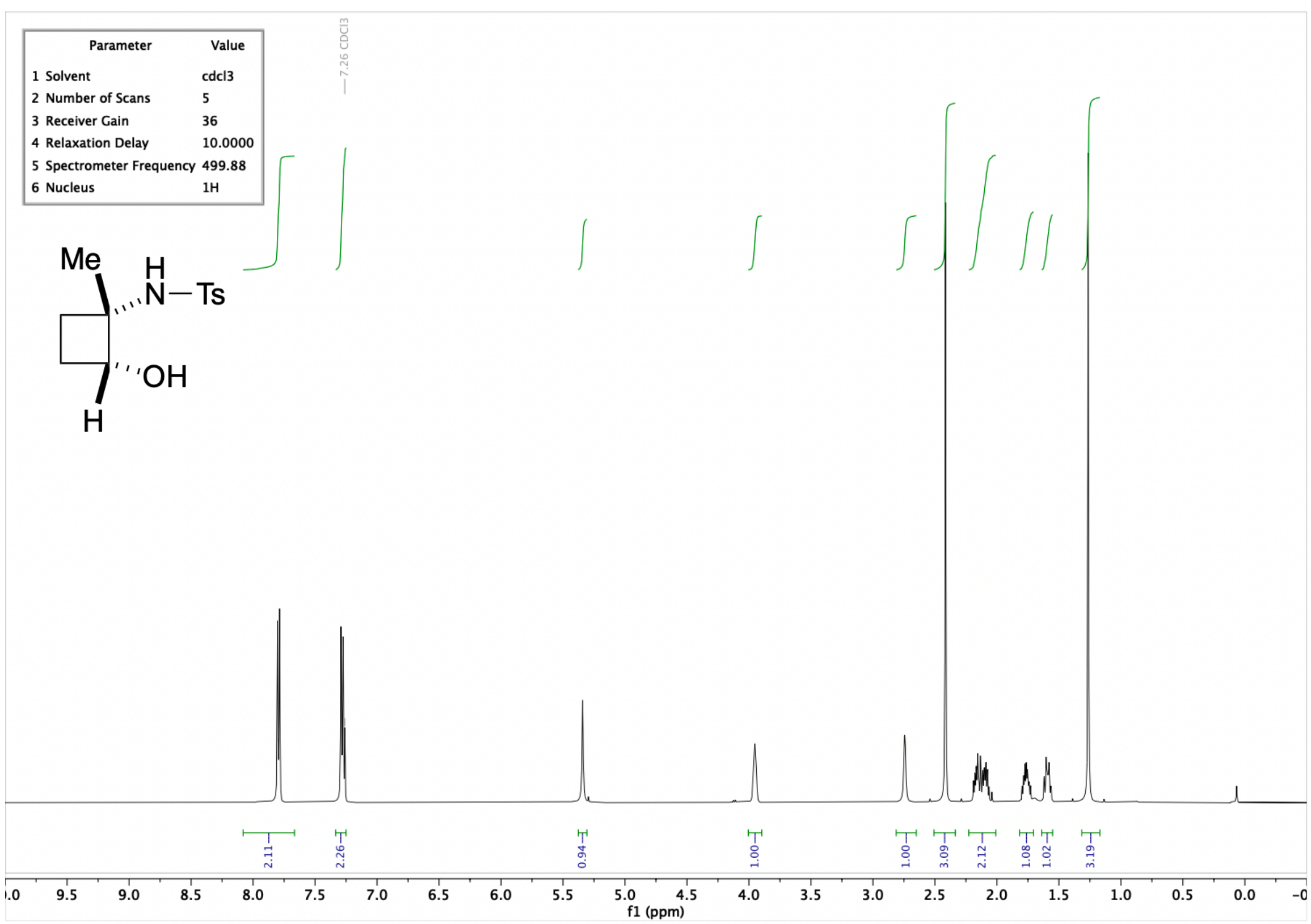

Parameter Value
1 Solvent cdcl3
2 Number of Scans 5
3 Receiver Gain 36
4 Relaxation Delay 10.0000
5 Spectrometer Frequency 499.88
6 Nucleus 1H
7.26 CDCl3
Me
H
N—Ts
OH
H
2.11
2.26
0.94
1.00
1.00
3.09
2.12
1.08
1.02
3.19
f1 (ppm)

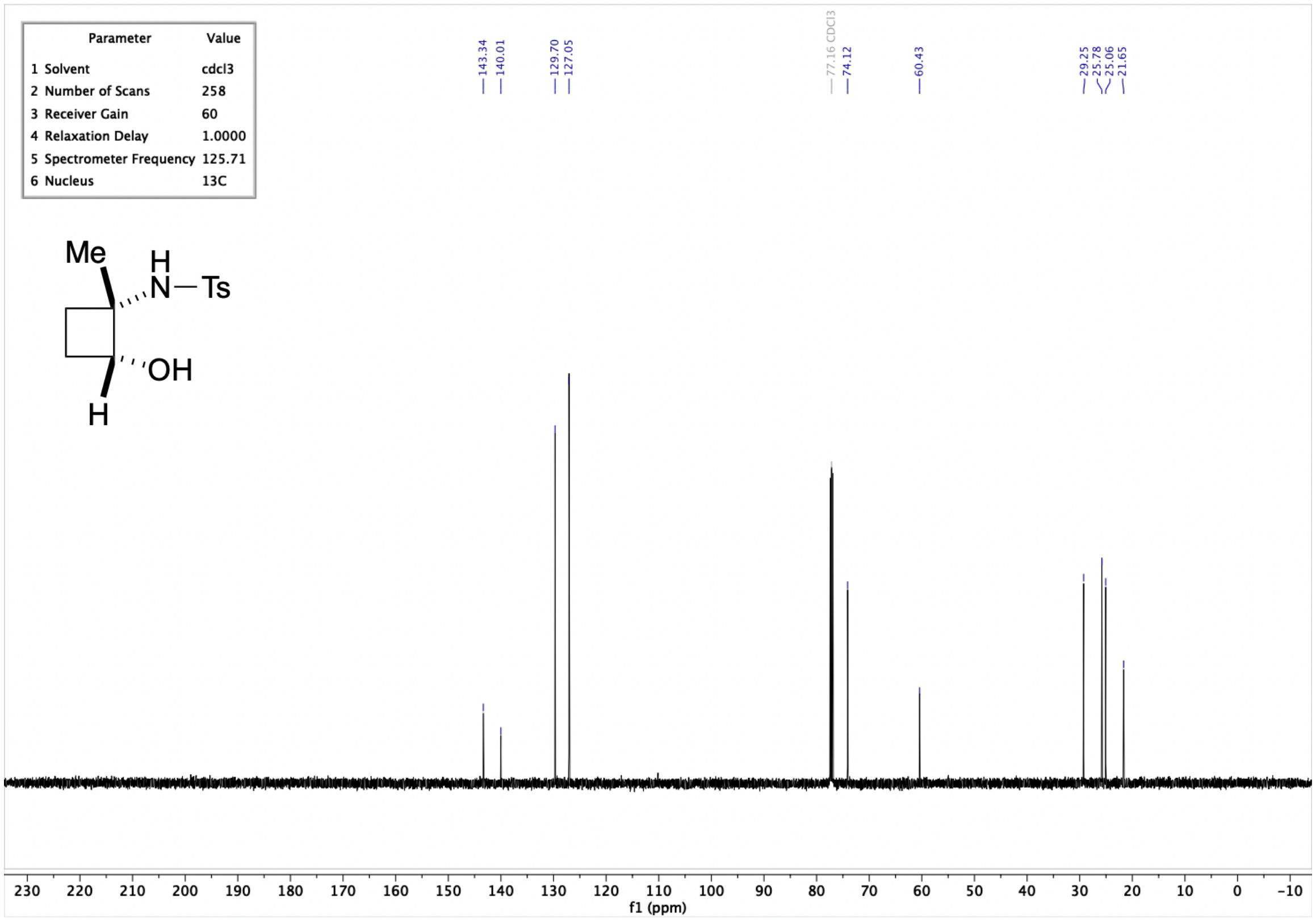
Parameter Value
1 Solvent cdcl3
2 Number of Scans 258
3 Receiver Gain 60
4 Relaxation Delay 1.0000
5 Spectrometer Frequency 125.71
6 Nucleus 13C
143.34
140.01
129.70
127.05
77.16 CDCl3
74.12
60.43
29.25
25.78
25.06
21.65
Me
H
N—Ts
OH
H
f1 (ppm)

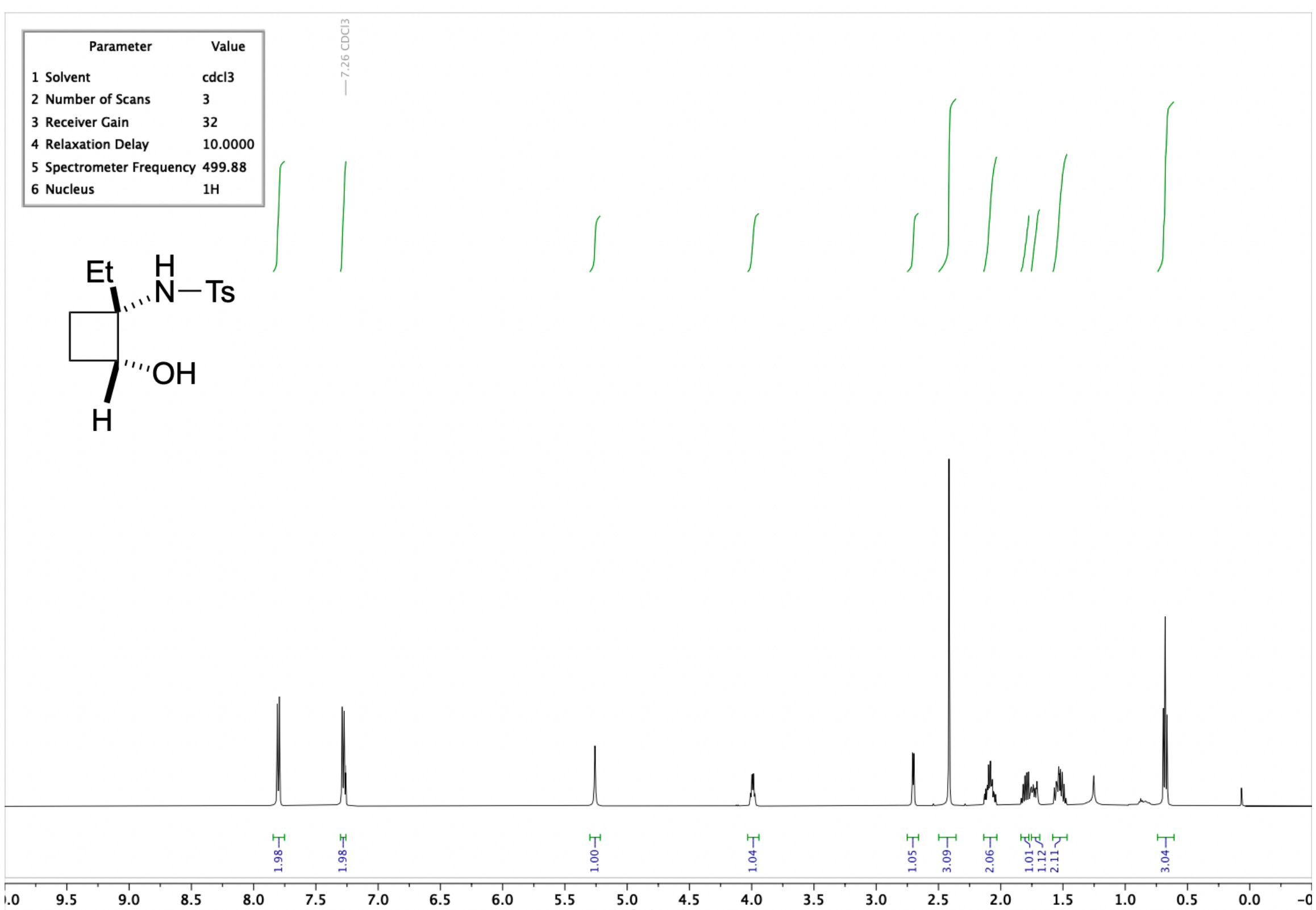
Parameter Value
1 Solvent cdcl3
2 Number of Scans 3
3 Receiver Gain 32
4 Relaxation Delay 10.0000
5 Spectrometer Frequency 499.88
6 Nucleus 1H
7.26 CDCl3
Et
H
N—Ts
OH
H
1.98
1.98
1.00
1.04
1.05
3.09
2.06
1.01
1.12
2.11
3.04
f1 (ppm)

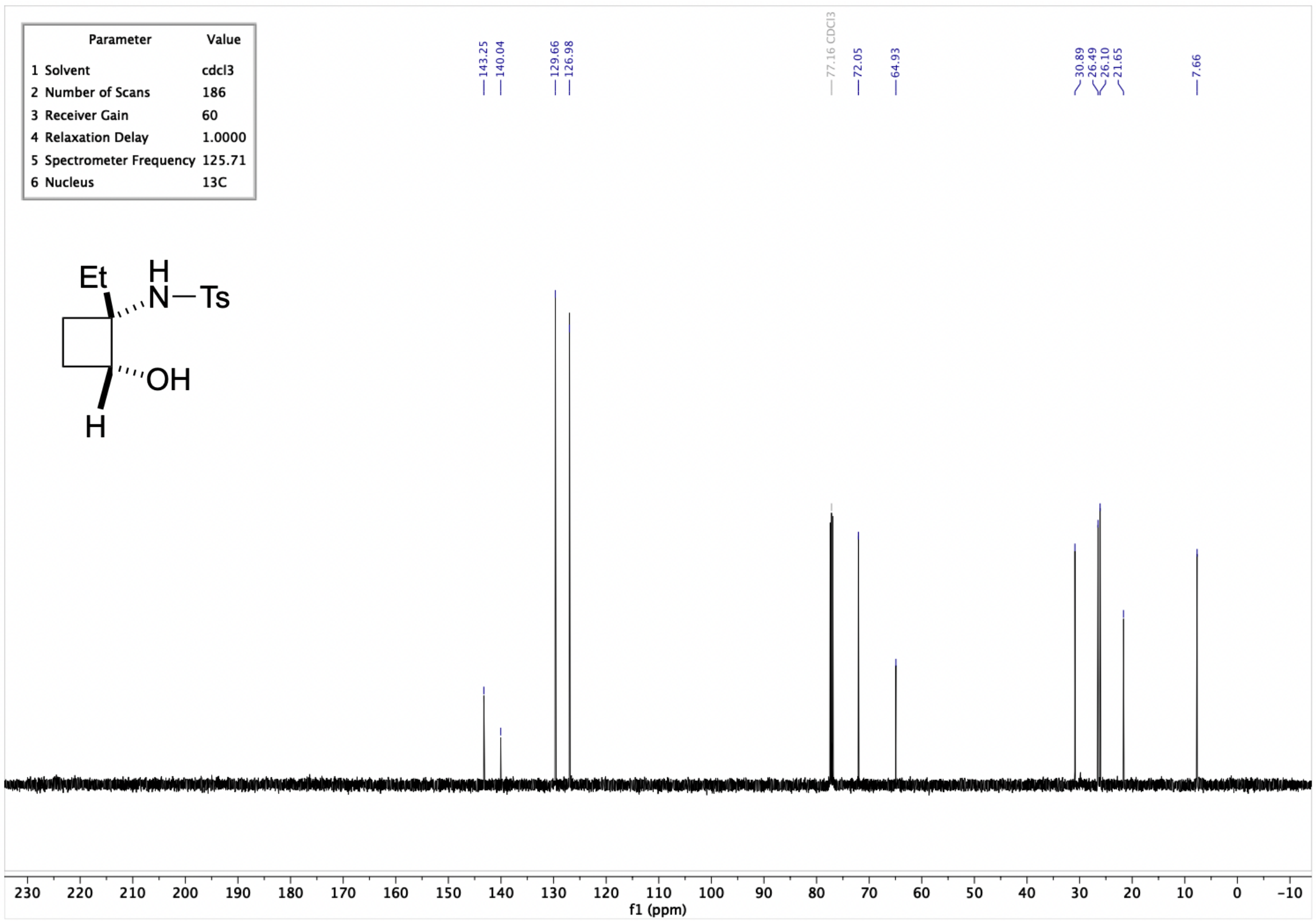

Parameter Value
1 Solvent cdcl3
2 Number of Scans 186
3 Receiver Gain 60
4 Relaxation Delay 1.0000
5 Spectrometer Frequency 125.71
6 Nucleus 13C
143.25
140.04
129.66
126.98
77.16 CDCl3
72.05
64.93
30.89
26.49
26.10
21.65
7.66
Et
H
N
Ts
OH
H
f1 (ppm)

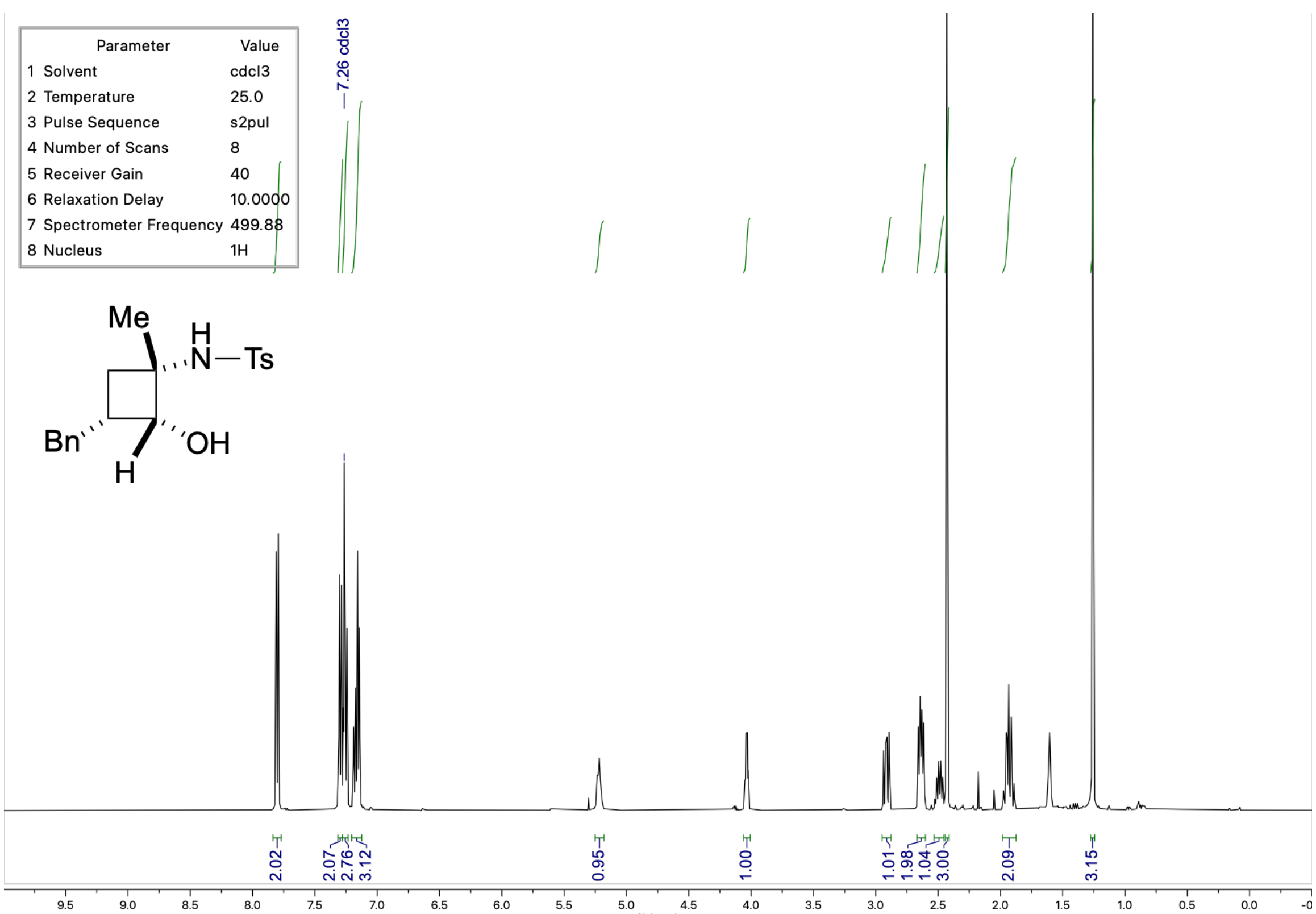

Parameter Value
1 Solvent cdcl3
2 Temperature 25.0
3 Pulse Sequence s2pul
4 Number of Scans 8
5 Receiver Gain 40
6 Relaxation Delay 10.0000
7 Spectrometer Frequency 499.88
8 Nucleus 1H
7.26 cdcl3
Me
H
N
Ts
Bn
OH
H
2.02
2.07
2.76
3.12
0.95
1.00
1.01
1.98
1.04
3.00
2.09
3.15
f1 (ppm)

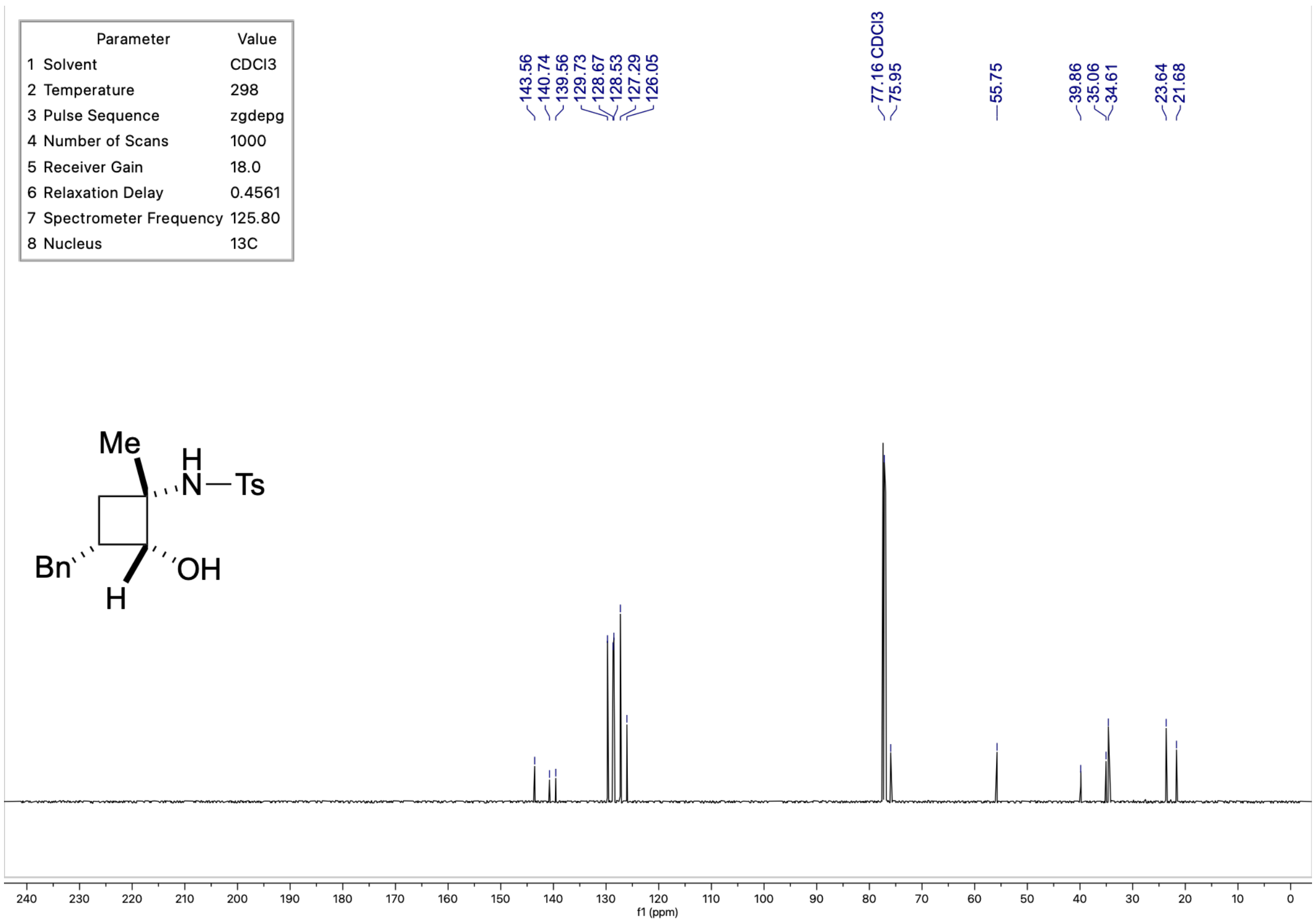
Parameter Value
1 Solvent CDCl3
2 Temperature 298
3 Pulse Sequence zgdepg
4 Number of Scans 1000
5 Receiver Gain 18.0
6 Relaxation Delay 0.4561
7 Spectrometer Frequency 125.80
8 Nucleus 13C
143.56
140.74
139.56
129.73
128.67
128.53
127.29
126.05
77.16 CDCl3
75.95
55.75
39.86
35.06
34.61
23.64
21.68
Me
H
N—Ts
Bn
OH
H
f1 (ppm)

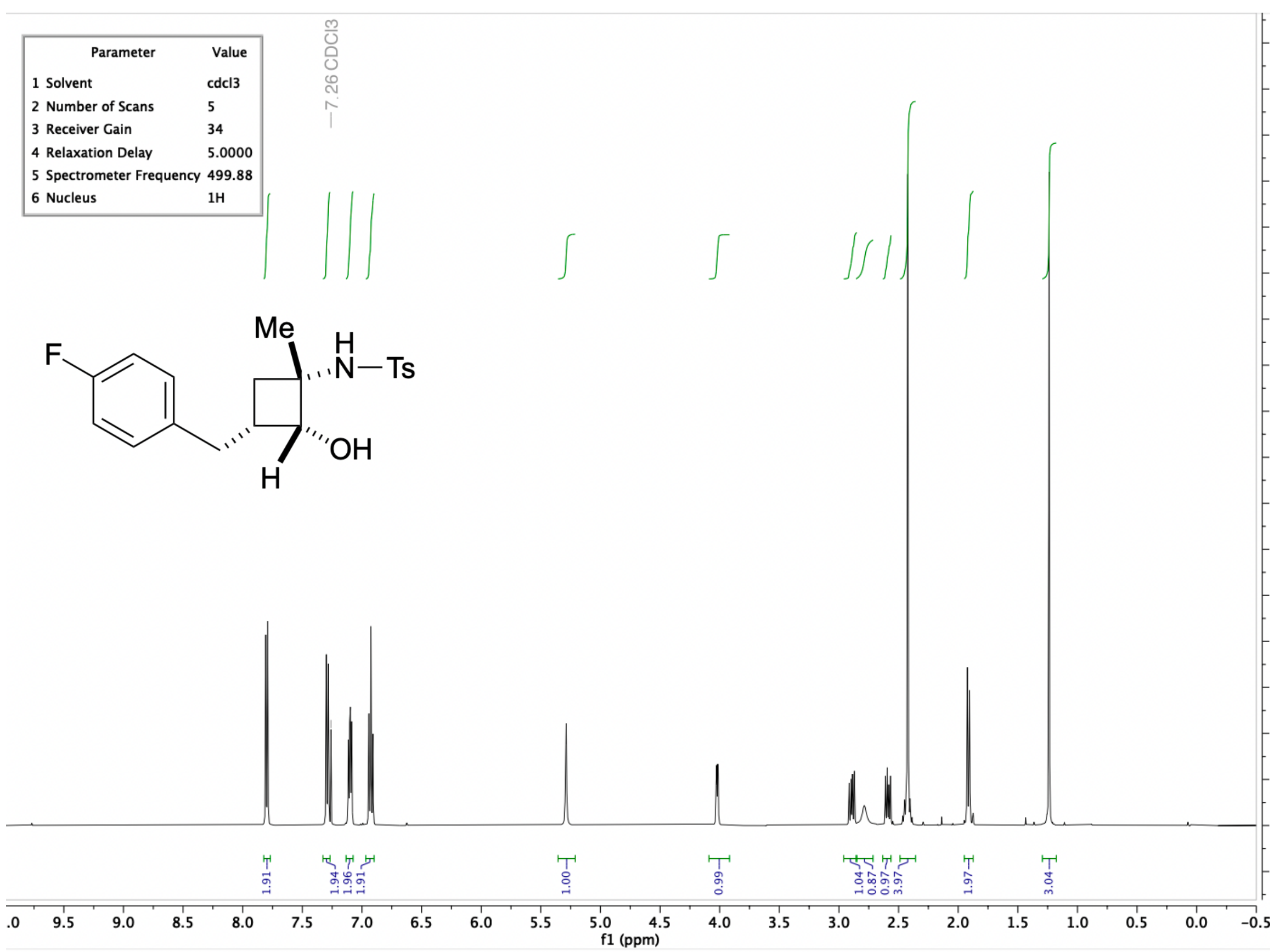
Parameter Value
1 Solvent cdcl3
2 Number of Scans 5
3 Receiver Gain 34
4 Relaxation Delay 5.0000
5 Spectrometer Frequency 499.88
6 Nucleus 1H
7.26 CDCl3
F
Me
H
N—Ts
OH
H
1.91
1.94
1.96
1.91
1.00
0.99
1.04
0.87
0.97
3.97
1.97
3.04
f1 (ppm)

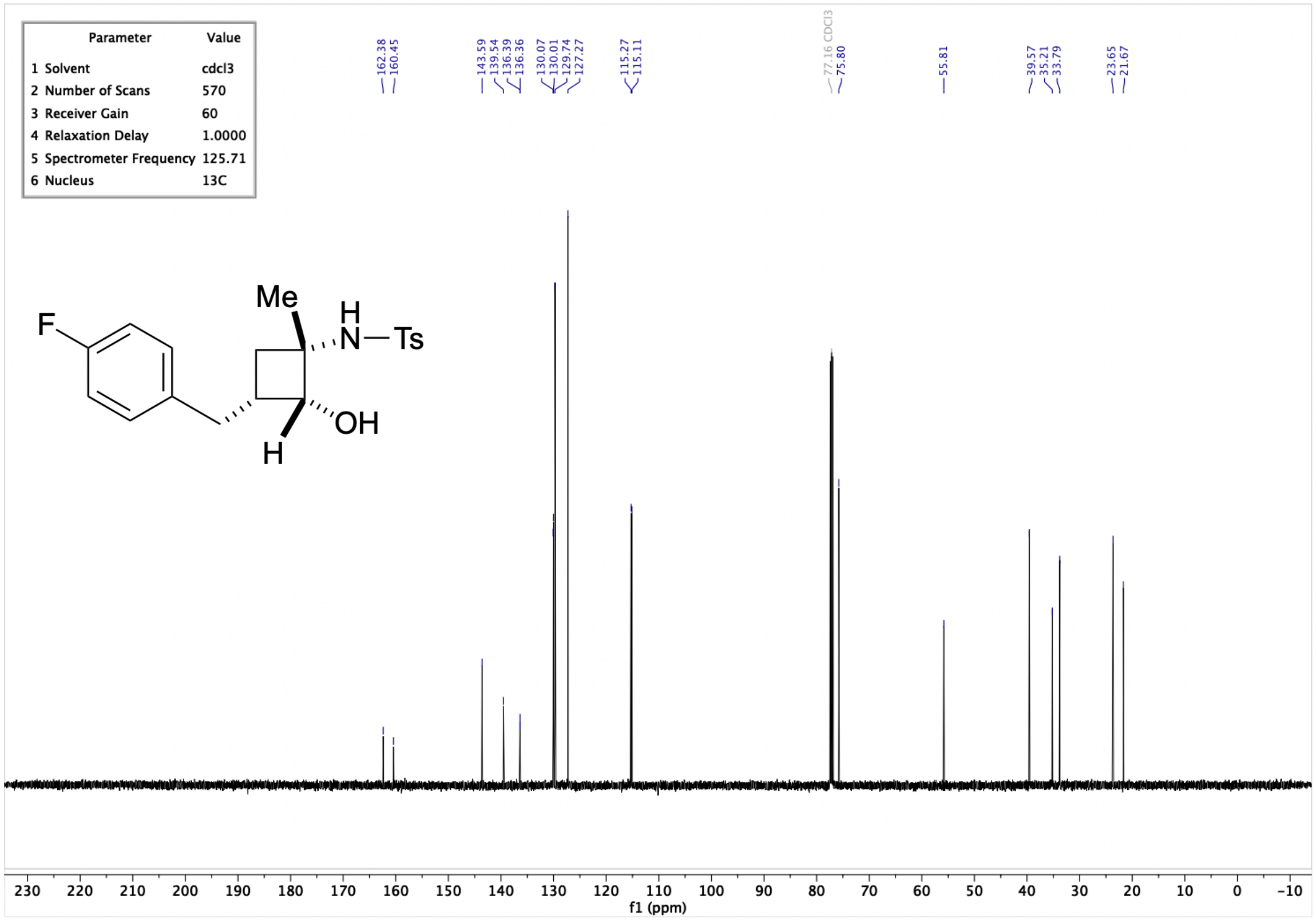
Parameter Value
1 Solvent cdcl3
2 Number of Scans 570
3 Receiver Gain 60
4 Relaxation Delay 1.0000
5 Spectrometer Frequency 125.71
6 Nucleus 13C
162.38
160.45
143.59
139.54
136.39
136.36
130.07
130.01
129.74
127.27
115.27
115.11
77.16 CDCl3
75.80
55.81
39.57
35.21
33.79
23.65
21.67
F
Me
H
N
Ts
OH
H
f1 (ppm)

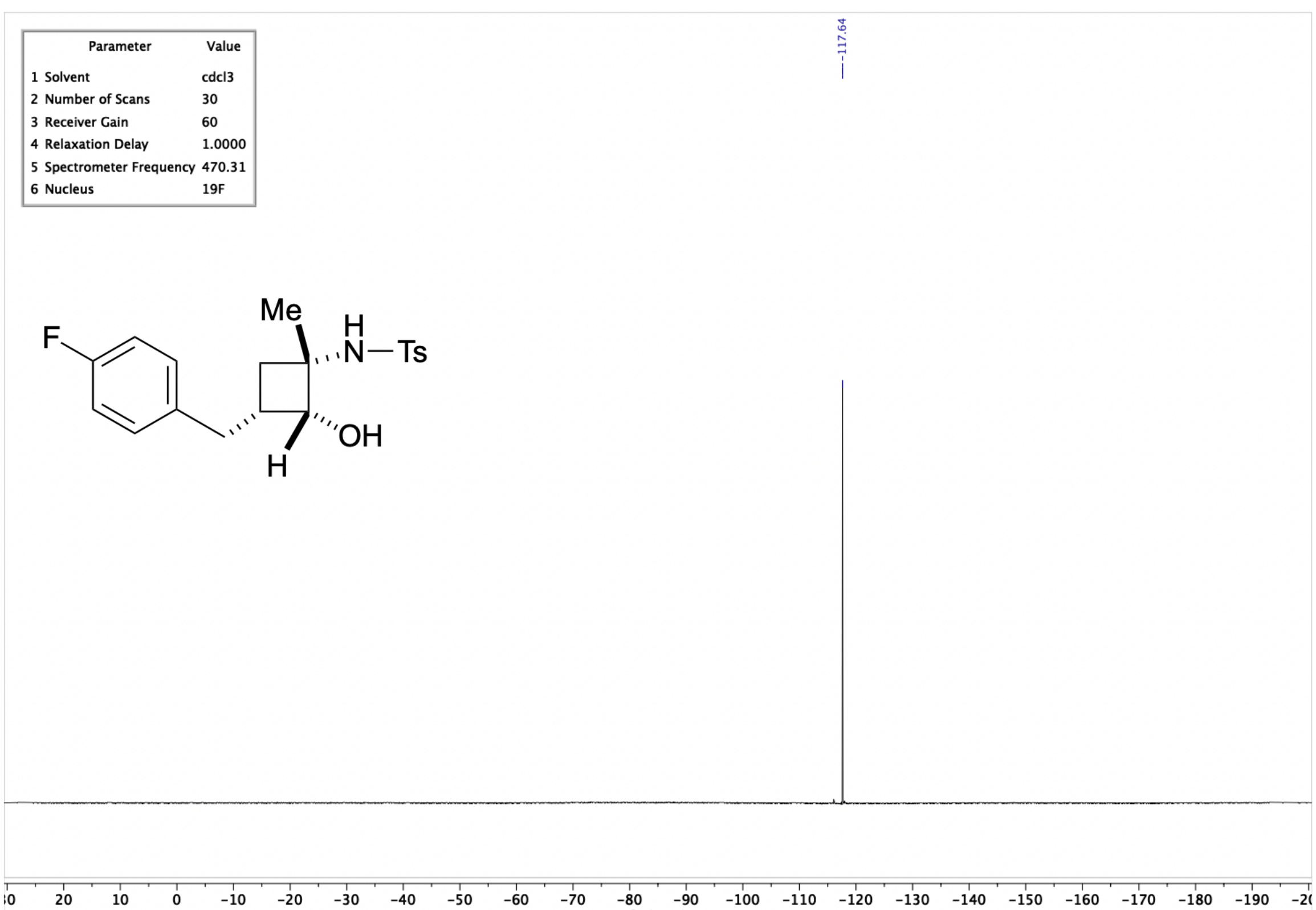
Parameter Value
1 Solvent cdcl3
2 Number of Scans 30
3 Receiver Gain 60
4 Relaxation Delay 1.0000
5 Spectrometer Frequency 470.31
6 Nucleus 19F
-117.64
F
Me
H
N
Ts
OH
H
f1 (ppm)

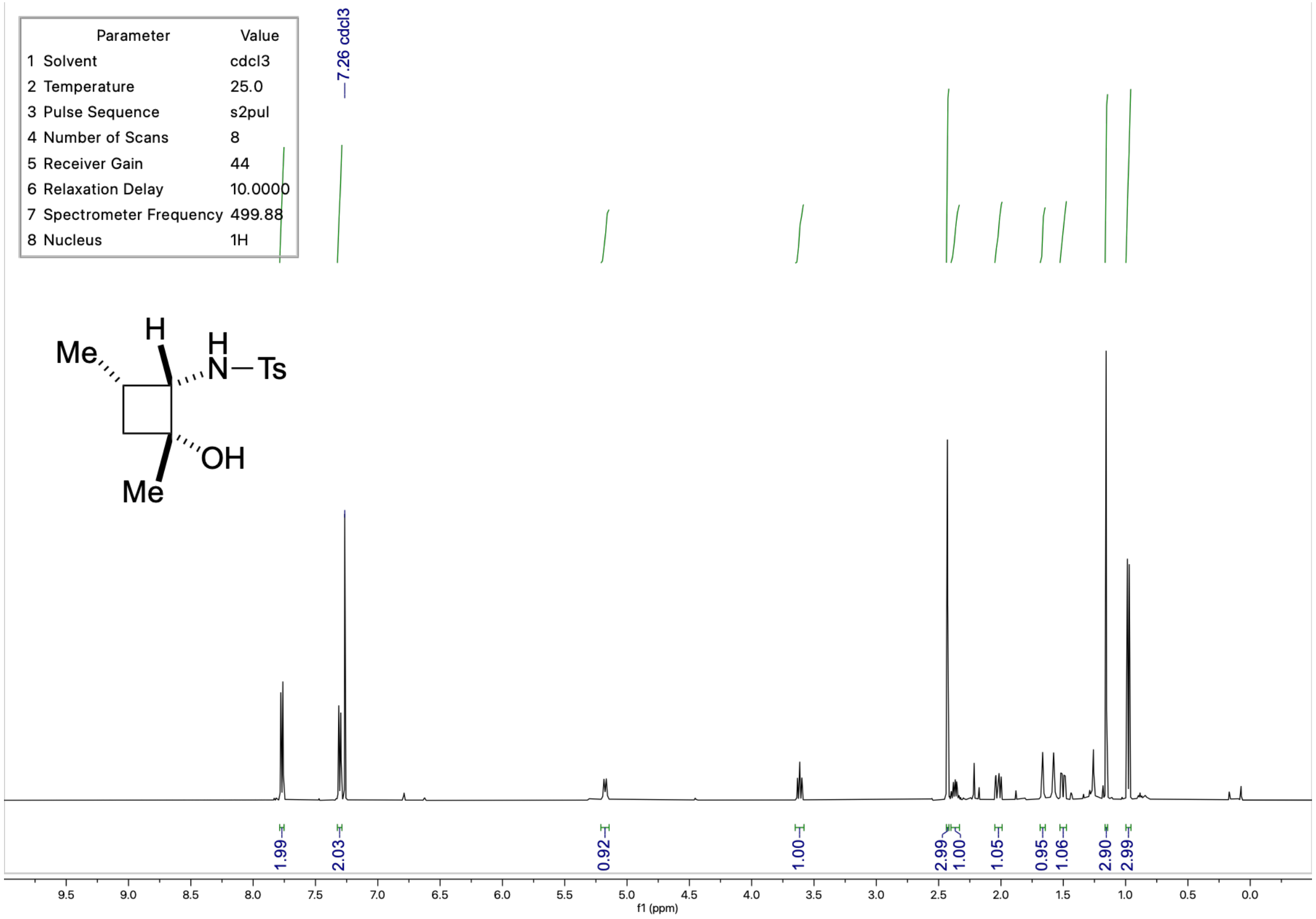

Parameter Value
1 Solvent cdcl3
2 Temperature 25.0
3 Pulse Sequence s2pul
4 Number of Scans 8
5 Receiver Gain 44
6 Relaxation Delay 10.0000
7 Spectrometer Frequency 499.88
8 Nucleus 1H
7.26 cdcl3
Me
H
H
N—Ts
OH
Me
1.99
2.03
0.92
1.00
2.99
1.00
1.05
0.95
1.06
2.90
2.99
f1 (ppm)

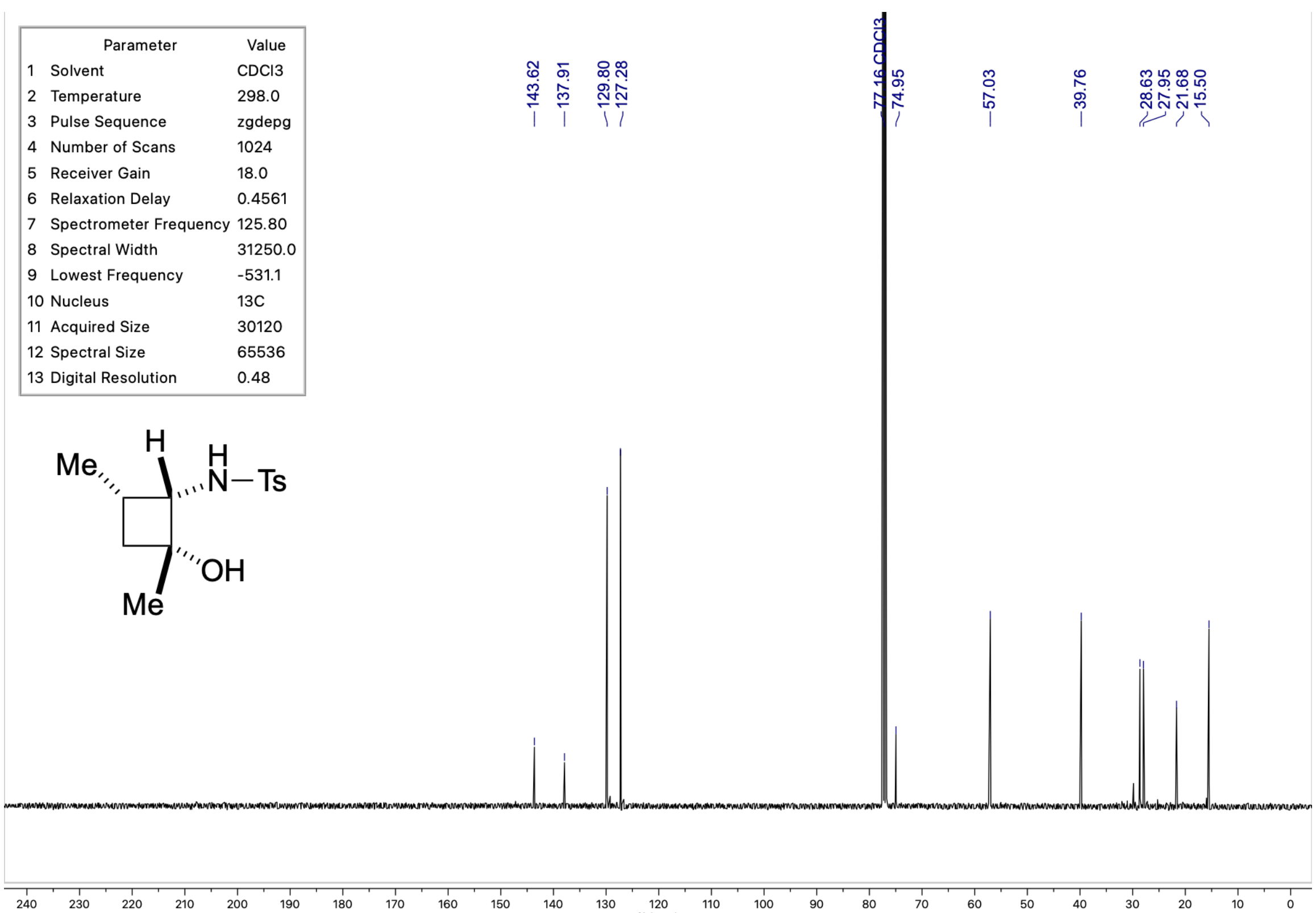

Parameter Value
1 Solvent CDCl3
2 Temperature 298.0
3 Pulse Sequence zgdepg
4 Number of Scans 1024
5 Receiver Gain 18.0
6 Relaxation Delay 0.4561
7 Spectrometer Frequency 125.80
8 Spectral Width 31250.0
9 Lowest Frequency -531.1
10 Nucleus 13C
11 Acquired Size 30120
12 Spectral Size 65536
13 Digital Resolution 0.48
143.62
137.91
129.80
127.28
77.16 CDCl3
74.95
57.03
39.76
28.63
27.95
21.68
15.50
Me
H
H
N—Ts
OH
Me
f1 (ppm)

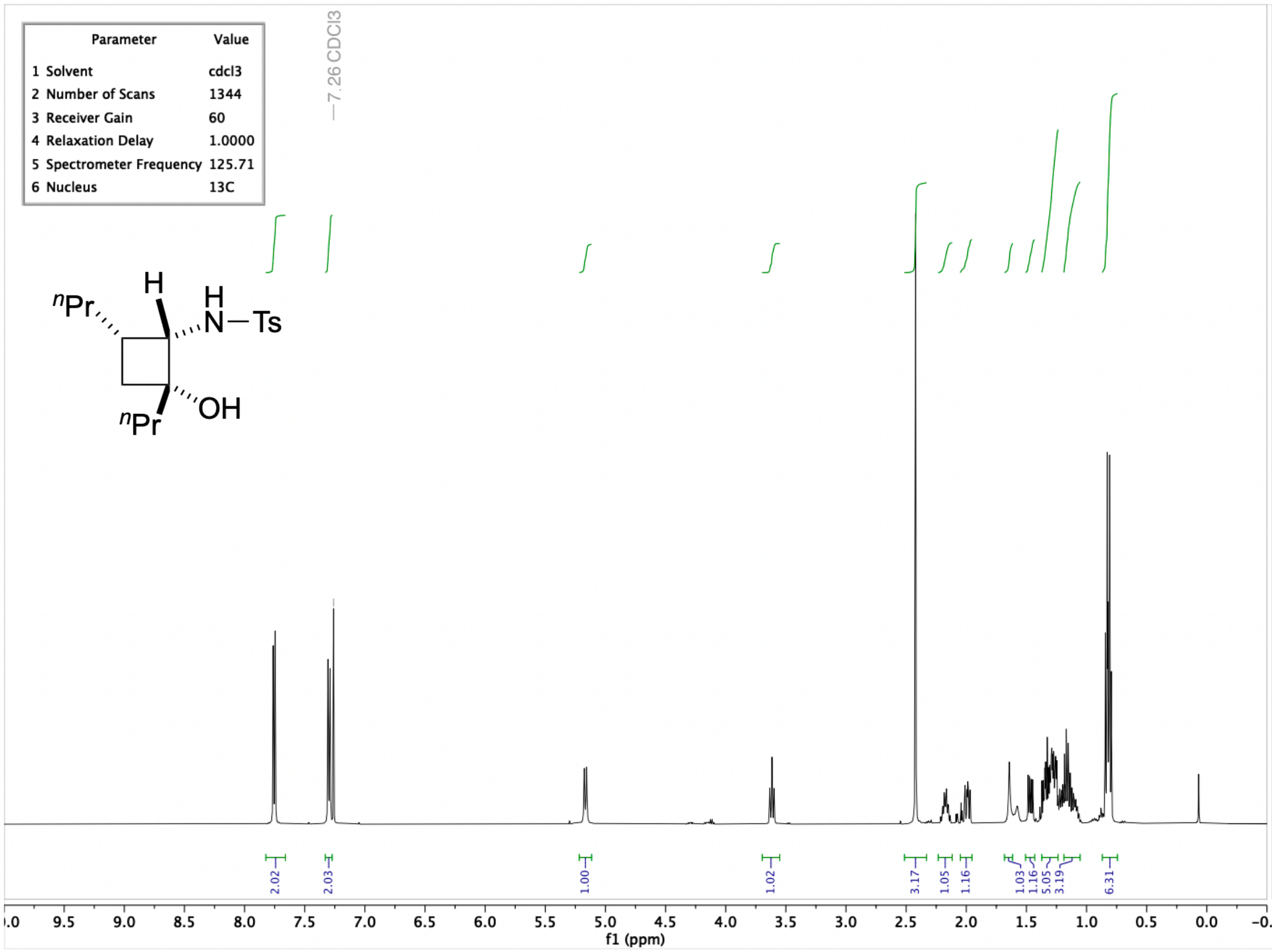
Parameter Value
1 Solvent cdcl3
2 Number of Scans 1344
3 Receiver Gain 60
4 Relaxation Delay 1.0000
5 Spectrometer Frequency 125.71
6 Nucleus 13C
7.26 CDCl3
nPr
H
H
N—Ts
OH
nPr
2.02
2.03
1.00
1.02
3.17
1.05
1.16
1.03
1.16
5.05
3.19
6.31
f1 (ppm)

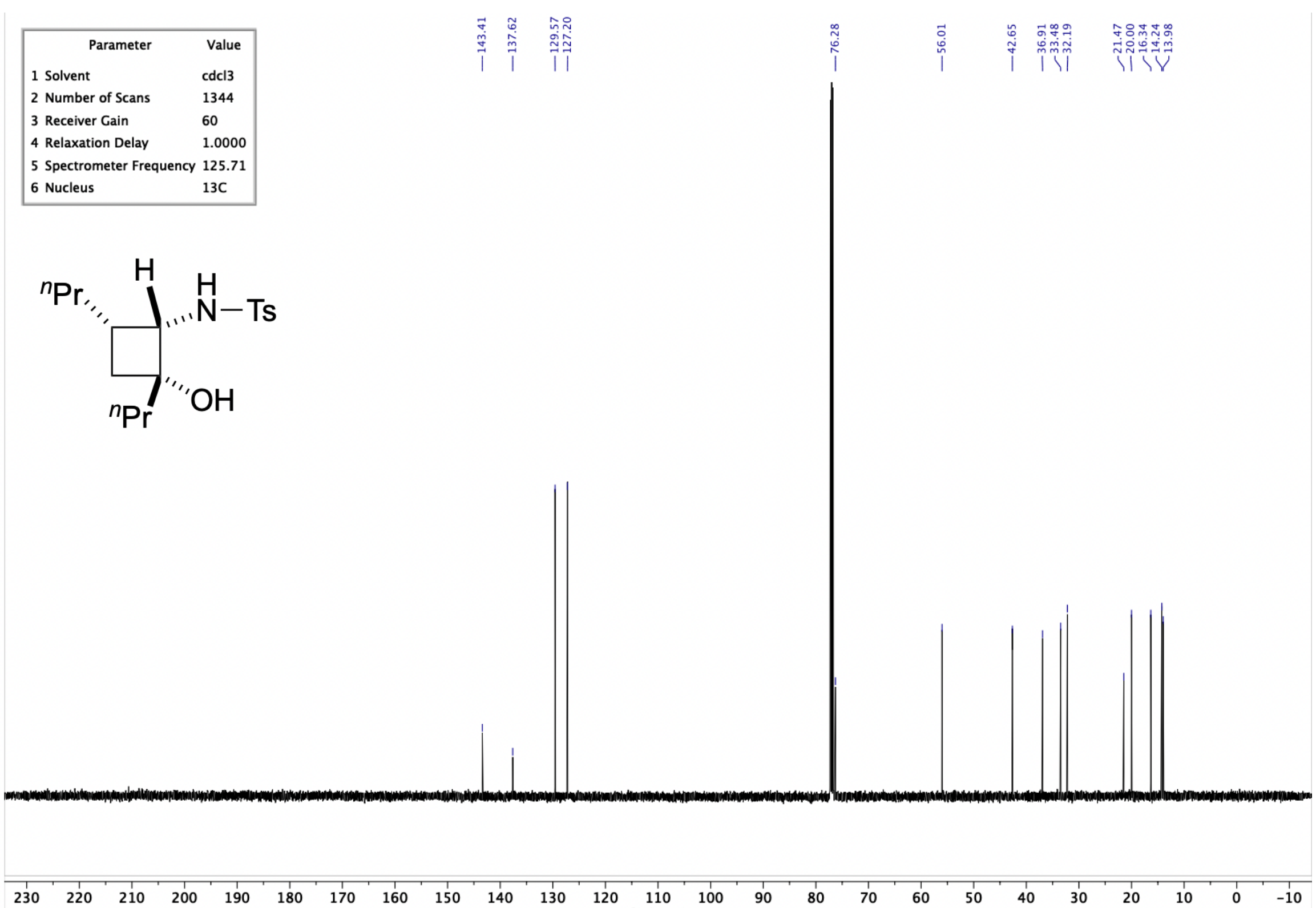
Parameter Value
1 Solvent cdcl3
2 Number of Scans 1344
3 Receiver Gain 60
4 Relaxation Delay 1.0000
5 Spectrometer Frequency 125.71
6 Nucleus 13C
143.41
137.62
129.57
127.20
76.28
56.01
42.65
36.91
33.48
32.19
21.47
20.00
16.34
14.24
13.98
nPr
H
H
N—Ts
OH
nPr
f1 (ppm)

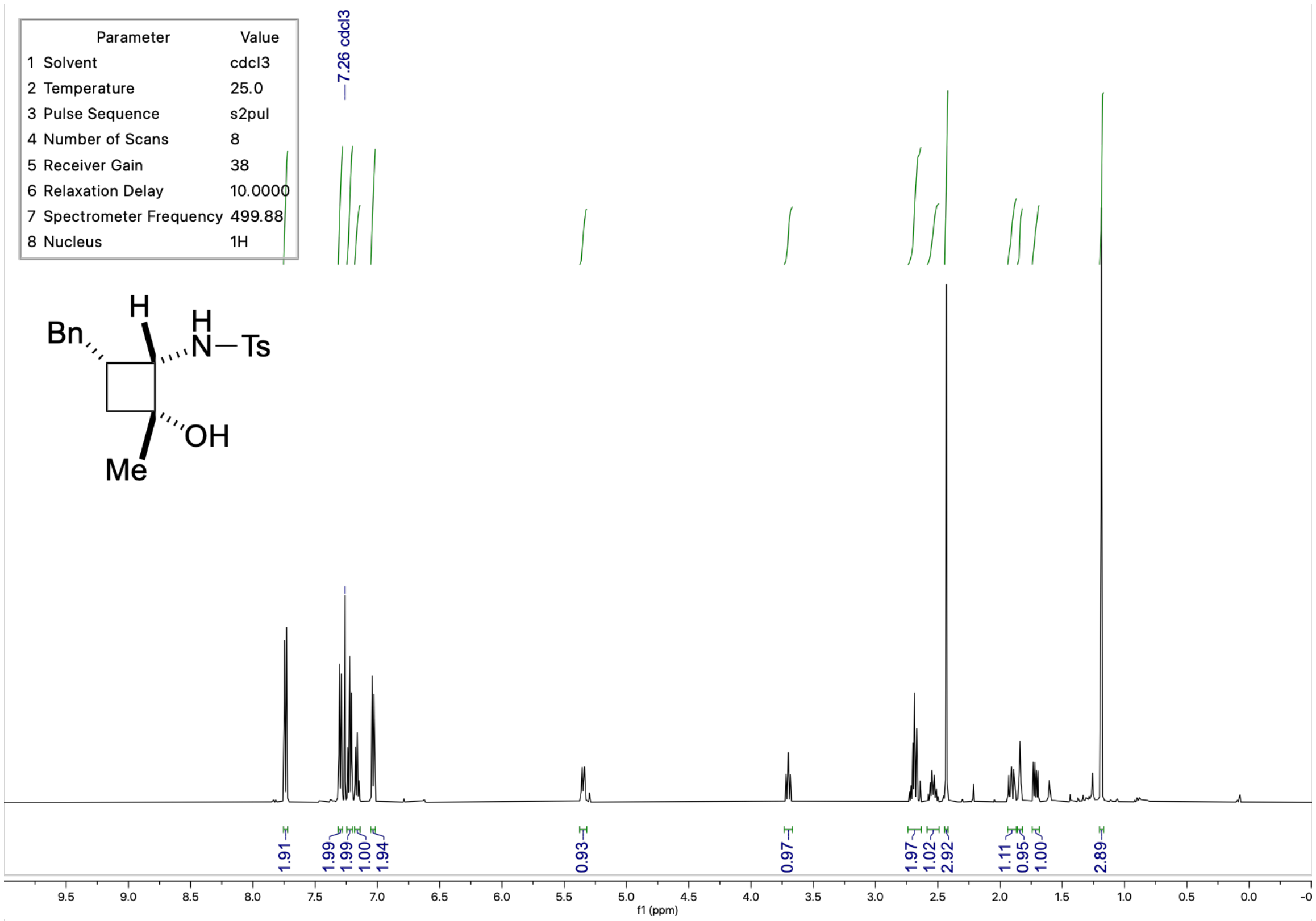
Parameter Value
1 Solvent cdcl3
2 Temperature 25.0
3 Pulse Sequence s2pul
4 Number of Scans 8
5 Receiver Gain 38
6 Relaxation Delay 10.0000
7 Spectrometer Frequency 499.88
8 Nucleus 1H
7.26 cdcl3
Bn
H
N—Ts
OH
Me
1.91
1.99
1.99
1.00
1.94
0.93
0.97
1.97
1.02
2.92
1.11
0.95
1.00
2.89
f1 (ppm)

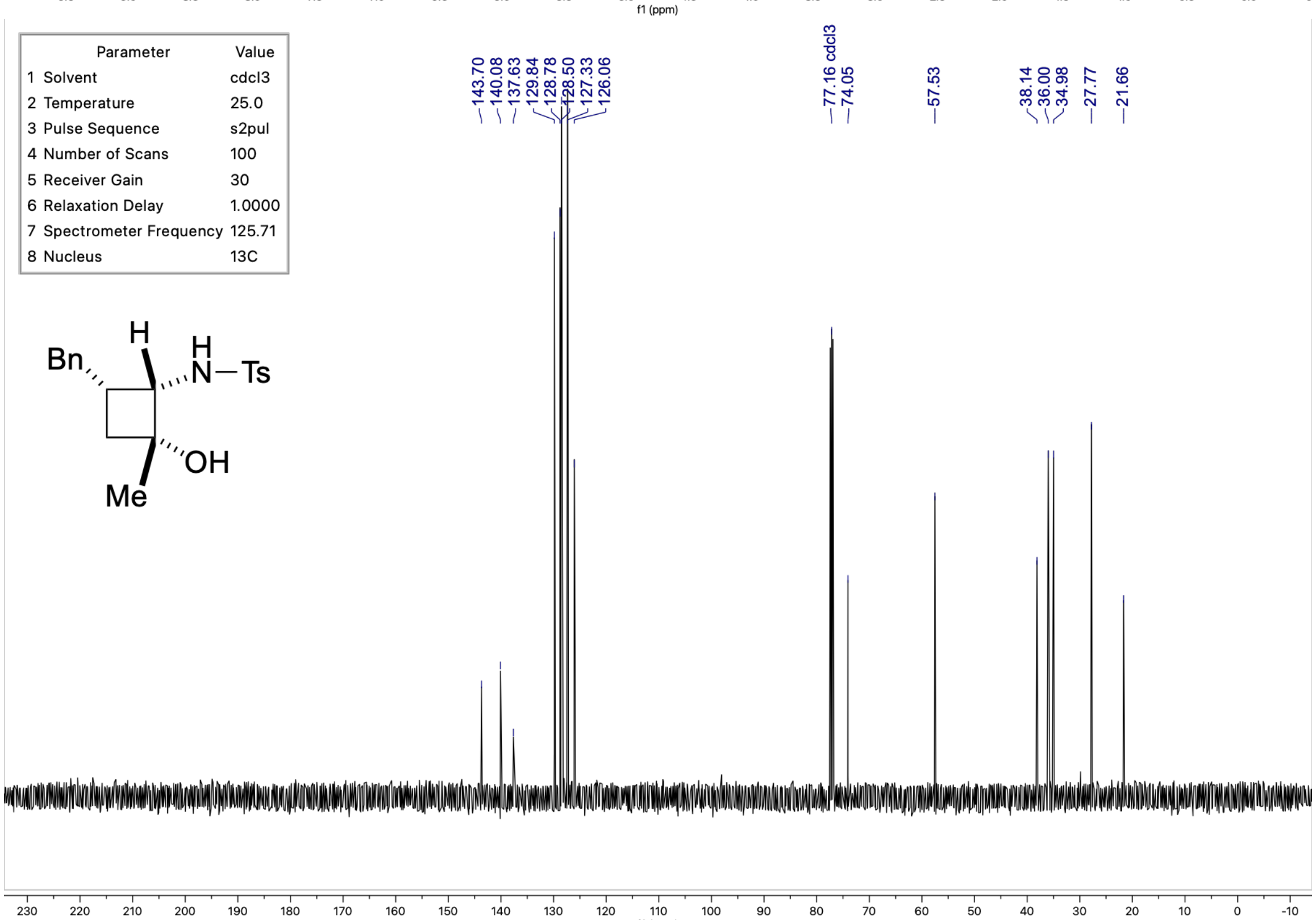
Parameter Value
1 Solvent cdcl3
2 Temperature 25.0
3 Pulse Sequence s2pul
4 Number of Scans 100
5 Receiver Gain 30
6 Relaxation Delay 1.0000
7 Spectrometer Frequency 125.71
8 Nucleus 13C
143.70
140.08
137.63
129.84
128.78
128.50
127.33
126.06
77.16 cdcl3
74.05
57.53
38.14
36.00
34.98
27.77
21.66
Bn
H
N—Ts
OH
Me
f1 (ppm)

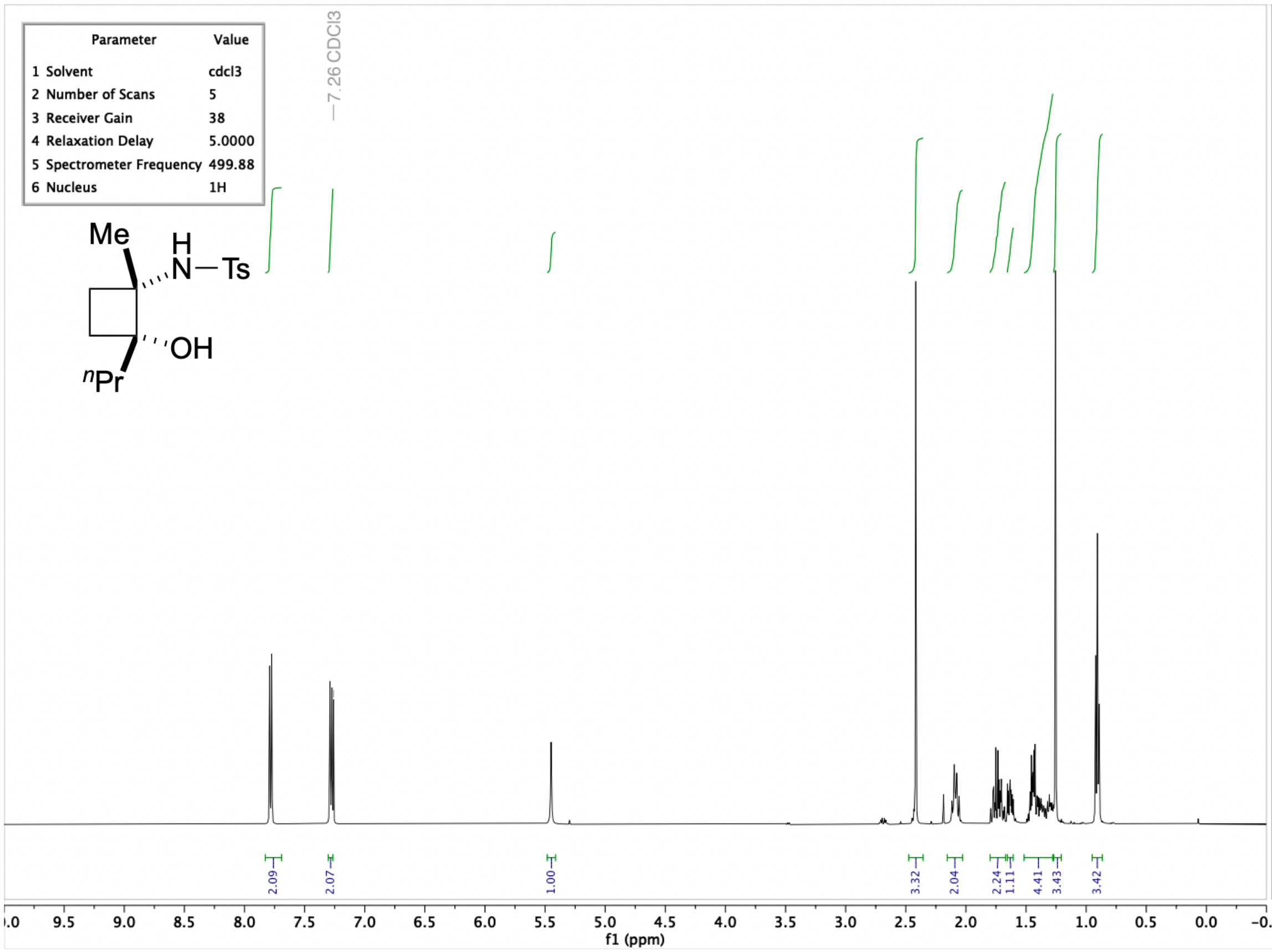
Parameter Value
1 Solvent cdcl3
2 Number of Scans 5
3 Receiver Gain 38
4 Relaxation Delay 5.0000
5 Spectrometer Frequency 499.88
6 Nucleus 1H
7.26 CDCl3
Me
H
N—Ts
OH
nPr
2.09
2.07
1.00
3.32
2.04
2.24
1.11
4.41
3.43
3.42
f1 (ppm)

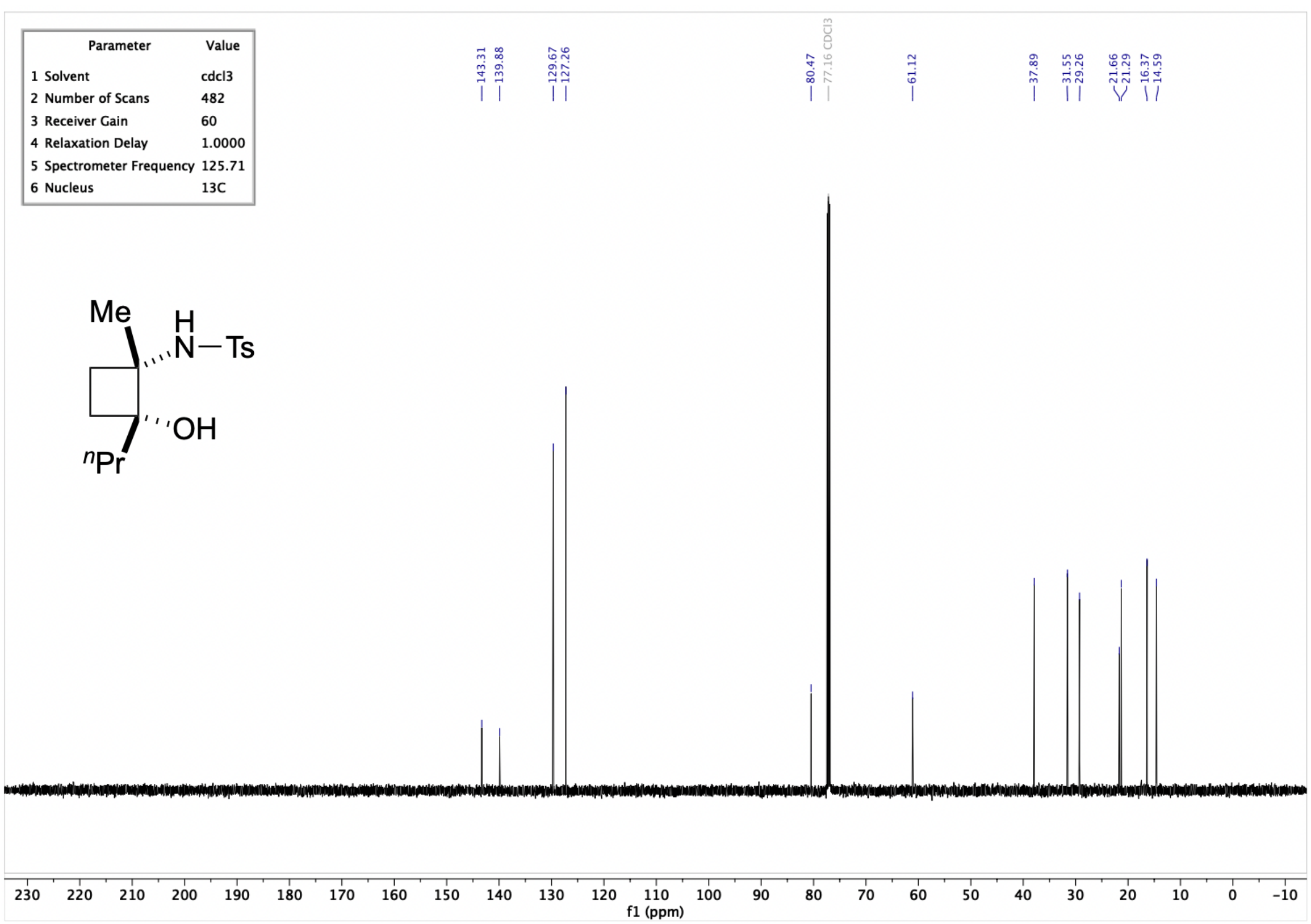
Parameter Value
1 Solvent cdcl3
2 Number of Scans 482
3 Receiver Gain 60
4 Relaxation Delay 1.0000
5 Spectrometer Frequency 125.71
6 Nucleus 13C
143.31
139.88
129.67
127.26
80.47
77.16 CDCl3
61.12
37.89
31.55
29.26
21.66
21.29
16.37
14.59
Me
H
N—Ts
OH
nPr
f1 (ppm)

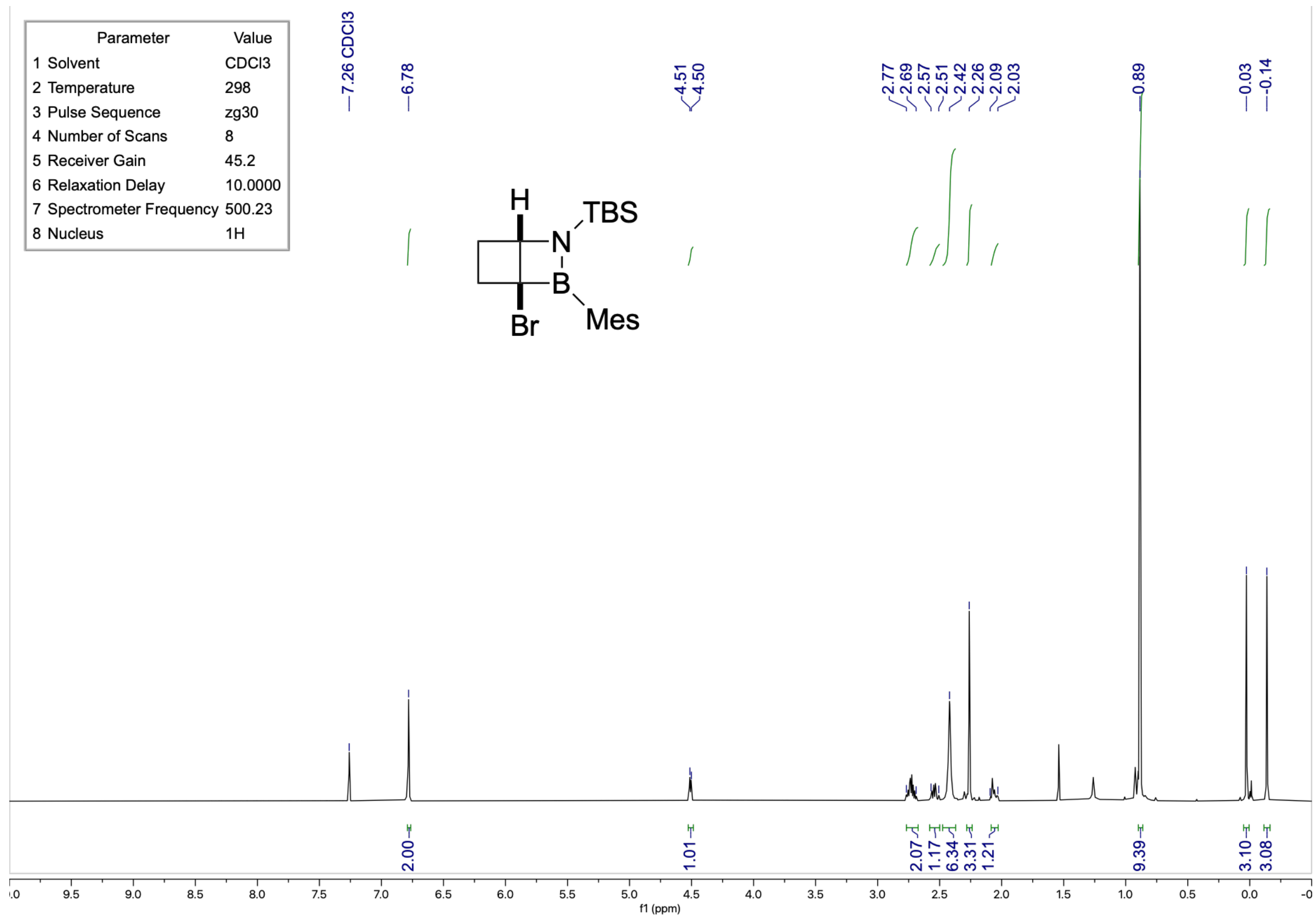
Parameter Value
1 Solvent CDCl3
2 Temperature 298
3 Pulse Sequence zg30
4 Number of Scans 8
5 Receiver Gain 45.2
6 Relaxation Delay 10.0000
7 Spectrometer Frequency 500.23
8 Nucleus 1H
7.26 CDCl3
6.78
4.51
4.50
2.77
2.69
2.57
2.51
2.42
2.26
2.09
2.03
0.89
0.03
-0.14
H
TBS
N
B
Br
Mes
2.00
1.01
2.07
1.17
6.34
3.31
1.21
9.39
3.10
3.08
f1 (ppm)

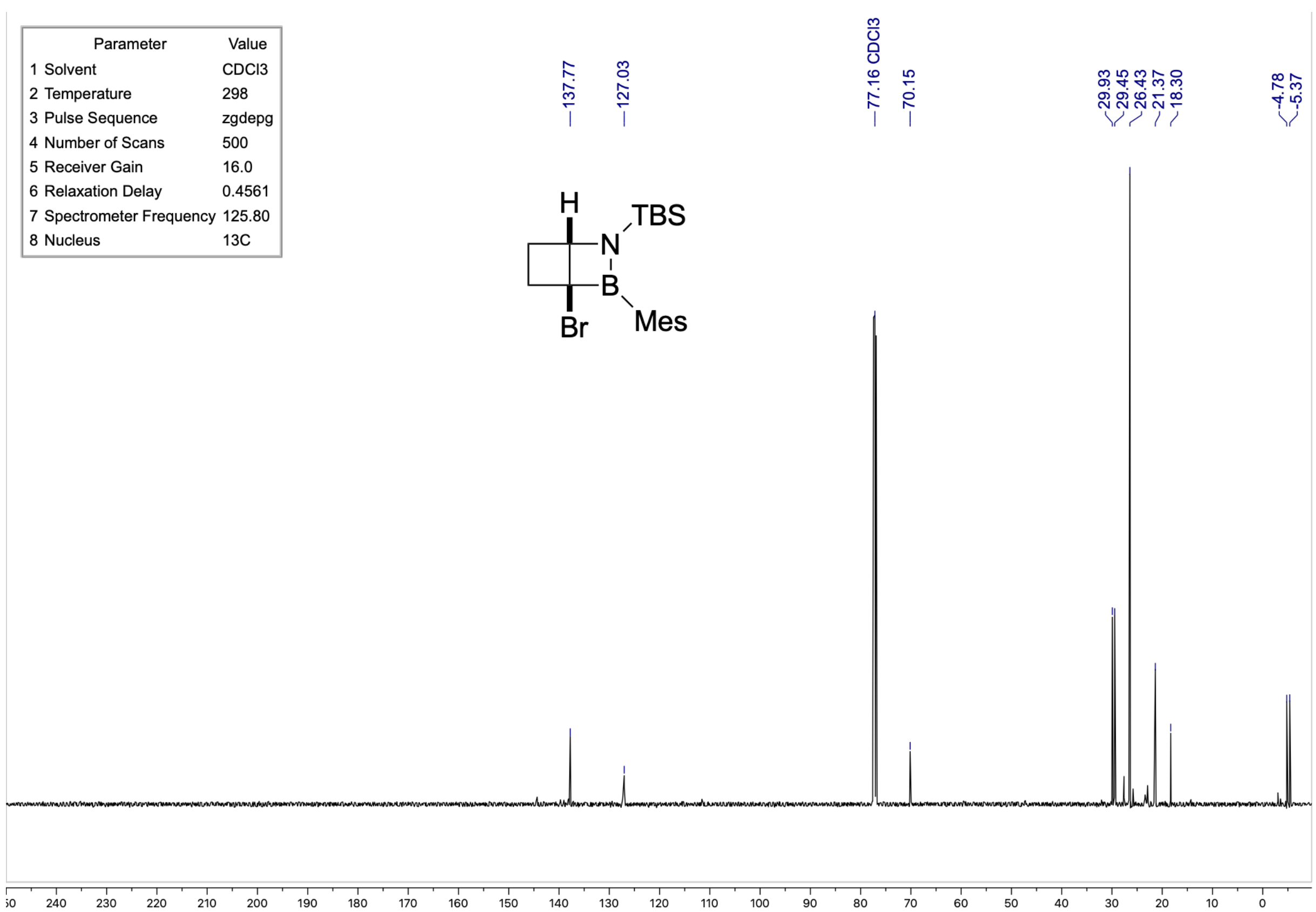
Parameter Value
1 Solvent CDCl3
2 Temperature 298
3 Pulse Sequence zgdepg
4 Number of Scans 500
5 Receiver Gain 16.0
6 Relaxation Delay 0.4561
7 Spectrometer Frequency 125.80
8 Nucleus 13C
137.77
127.03
77.16 CDCl3
70.15
29.93
29.45
26.43
21.37
18.30
-4.78
-5.37
H
TBS
N
B
Br
Mes
f1 (ppm)

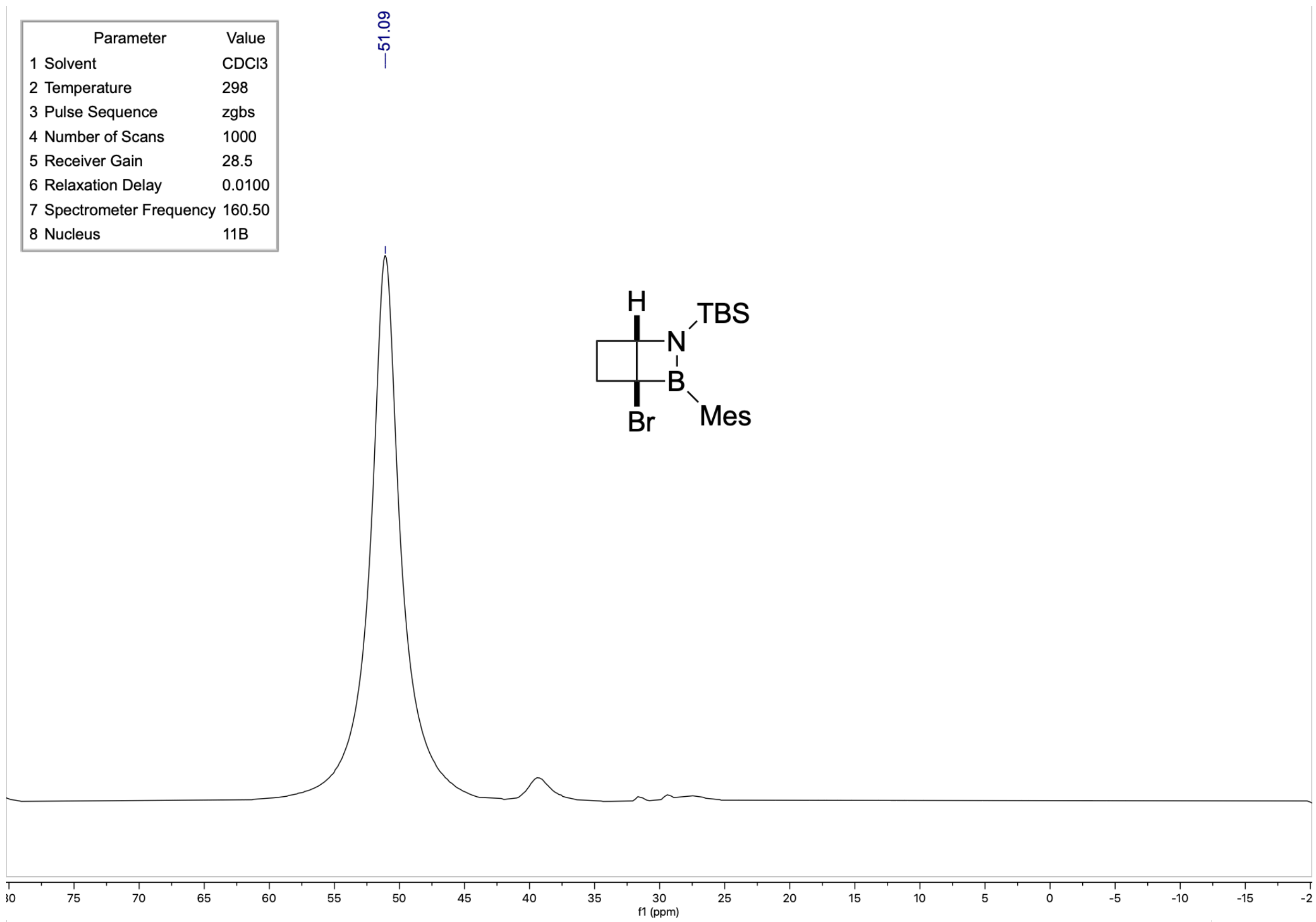
Parameter Value
1 Solvent CDCl3
2 Temperature 298
3 Pulse Sequence zgbs
4 Number of Scans 1000
5 Receiver Gain 28.5
6 Relaxation Delay 0.0100
7 Spectrometer Frequency 160.50
8 Nucleus 11B
51.09
H
TBS
N
B
Br
Mes
f1 (ppm)

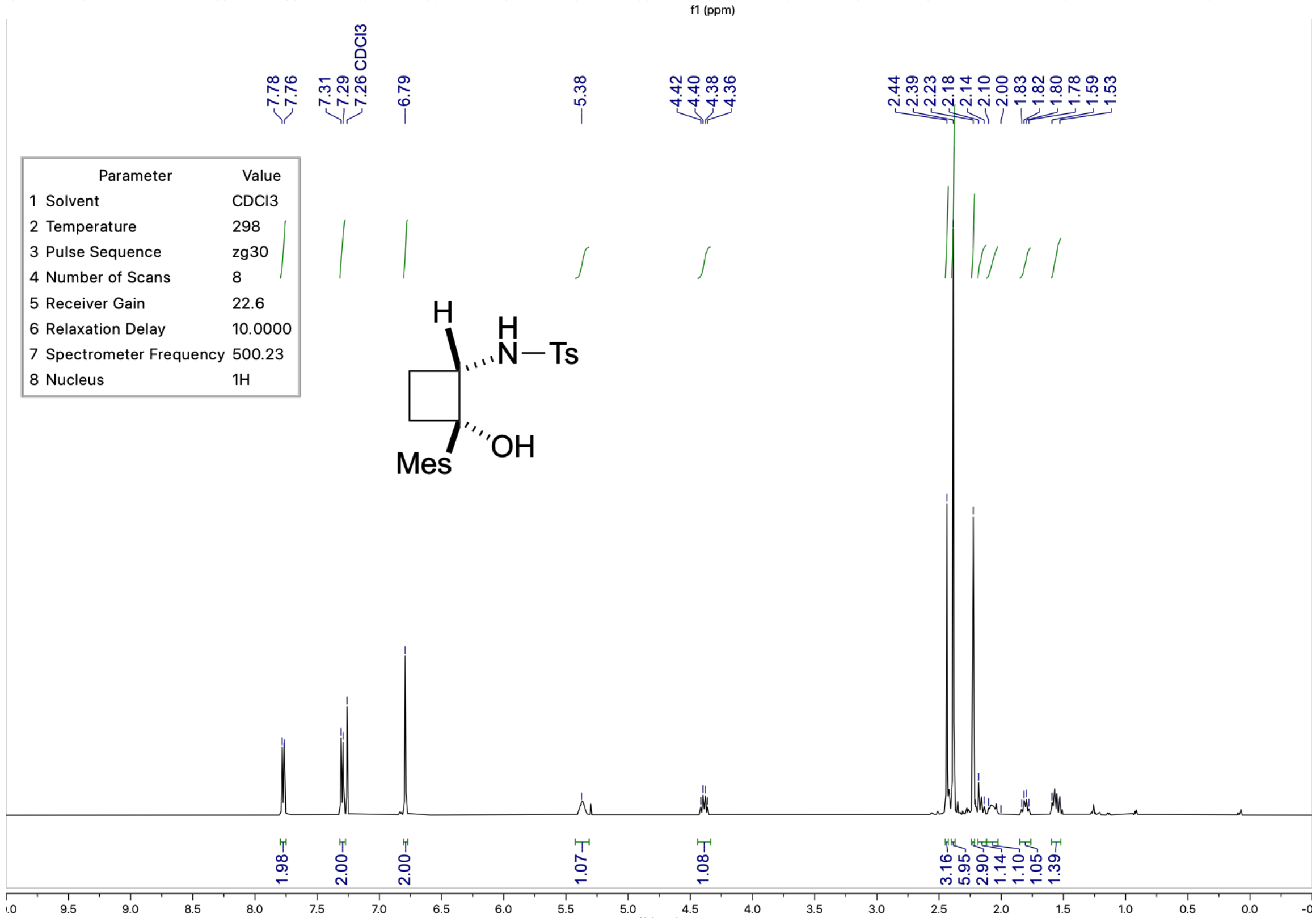
Parameter Value
1 Solvent CDCl3
2 Temperature 298
3 Pulse Sequence zg30
4 Number of Scans 8
5 Receiver Gain 22.6
6 Relaxation Delay 10.0000
7 Spectrometer Frequency 500.23
8 Nucleus 1H
7.78 7.76 7.31 7.29 7.26 CDCl3 6.79 5.38 4.42 4.40 4.38 4.36 2.44 2.39 2.23 2.18 2.14 2.10 2.00 1.83 1.82 1.80 1.78 1.59 1.53
H
H
N—Ts
OH
Mes
1.98 2.00 2.00 1.07 1.08 3.16 5.95 2.90 1.14 1.10 1.05 1.39
f1 (ppm)

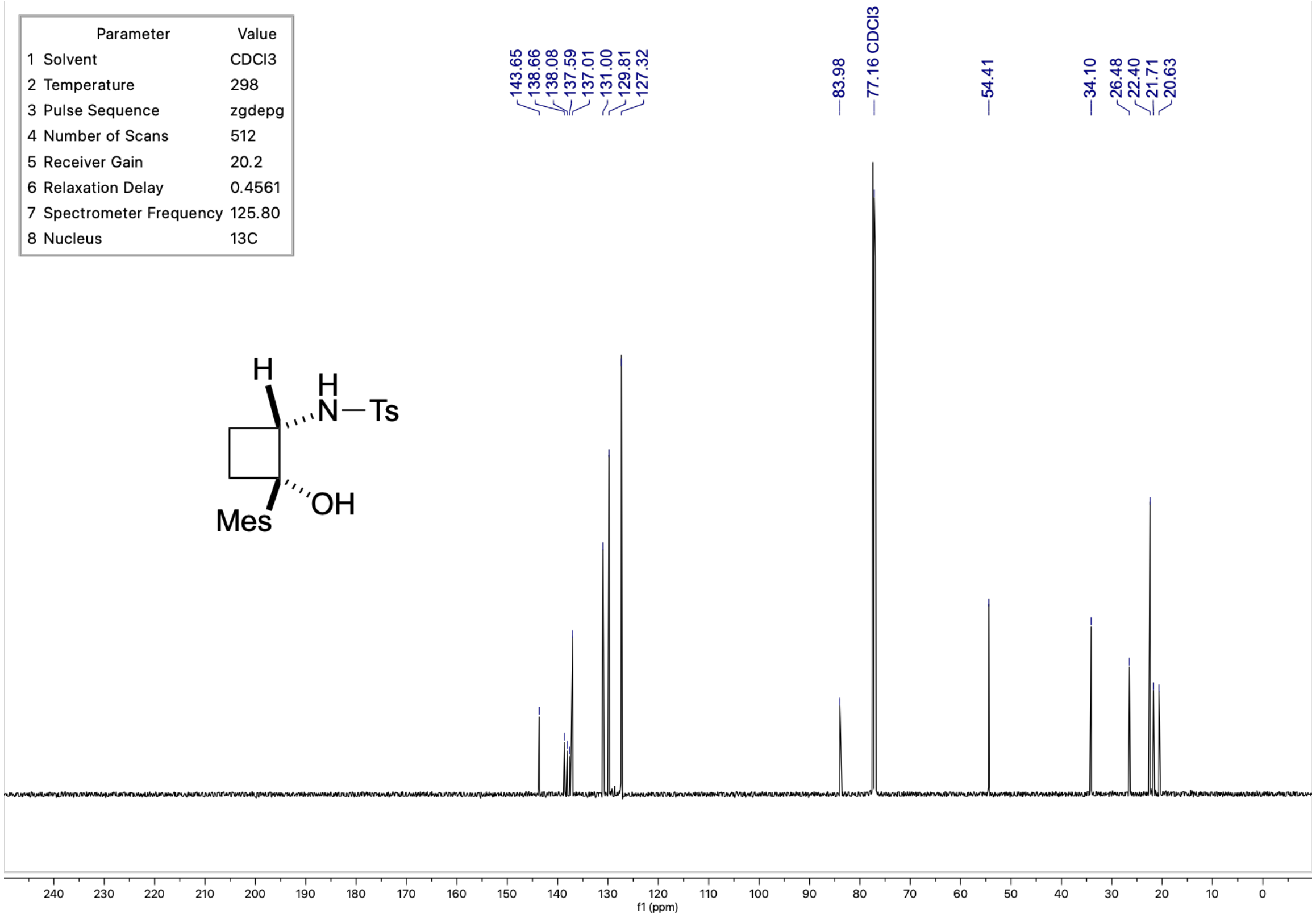
143.65
138.66
138.08
137.59
137.01
131.00
129.81
127.32
83.98
77.16 CDCl3
54.41
34.10
26.48
22.40
21.71
20.63
Parameter Value
1 Solvent CDCl3
2 Temperature 298
3 Pulse Sequence zgdepg
4 Number of Scans 512
5 Receiver Gain 20.2
6 Relaxation Delay 0.4561
7 Spectrometer Frequency 125.80
8 Nucleus 13C
H
H
N—Ts
OH
Mes
f1 (ppm)

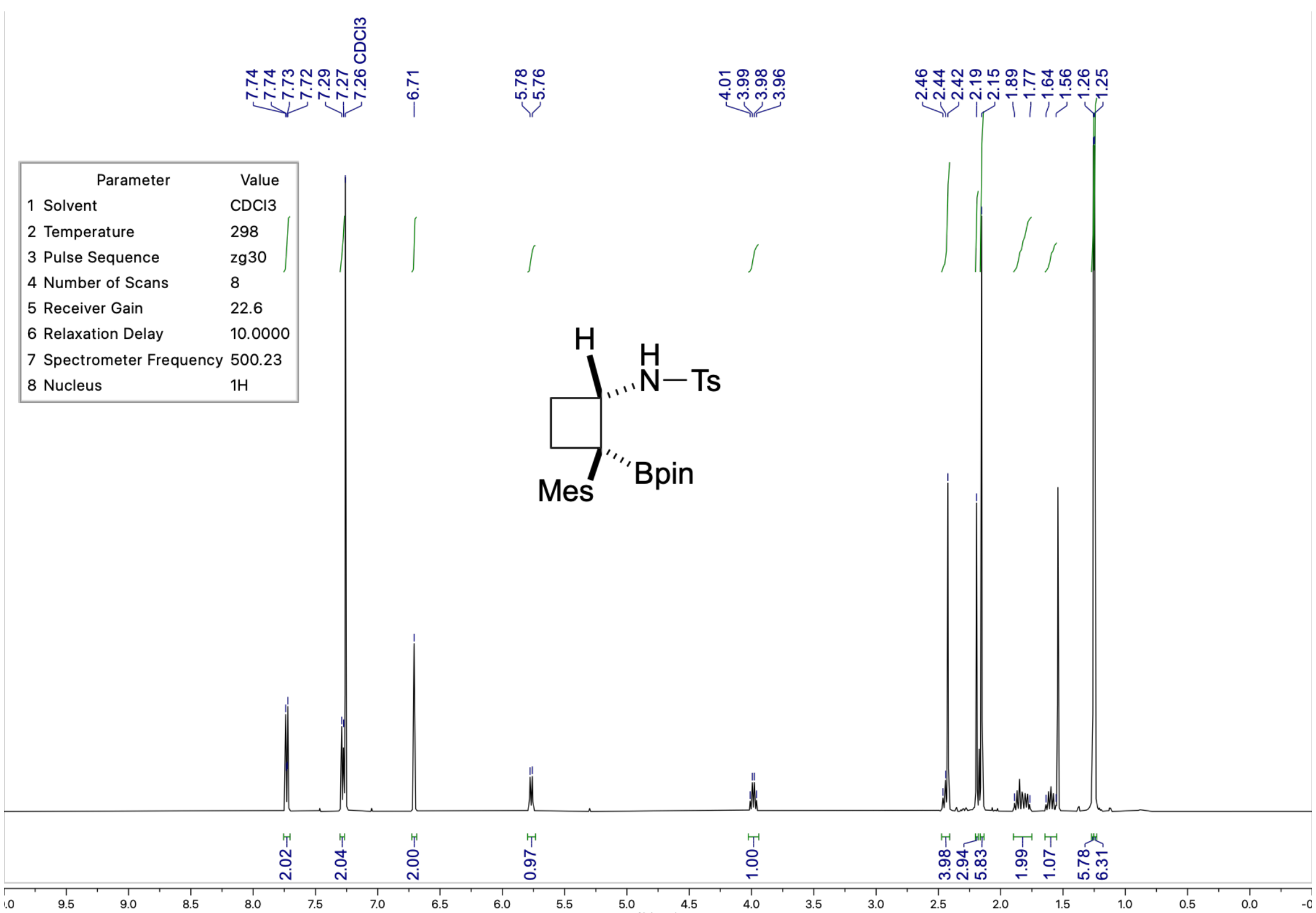
7.74
7.74
7.73
7.72
7.29
7.27
7.26 CDCl3
6.71
5.78
5.76
4.01
3.99
3.98
3.96
2.46
2.44
2.42
2.19
2.15
1.89
1.77
1.64
1.56
1.26
1.25
Parameter Value
1 Solvent CDCl3
2 Temperature 298
3 Pulse Sequence zg30
4 Number of Scans 8
5 Receiver Gain 22.6
6 Relaxation Delay 10.0000
7 Spectrometer Frequency 500.23
8 Nucleus 1H
H
H
N—Ts
Bpin
Mes
2.02
2.04
2.00
0.97
1.00
3.98
2.94
5.83
1.99
1.07
5.78
6.31
f1 (ppm)

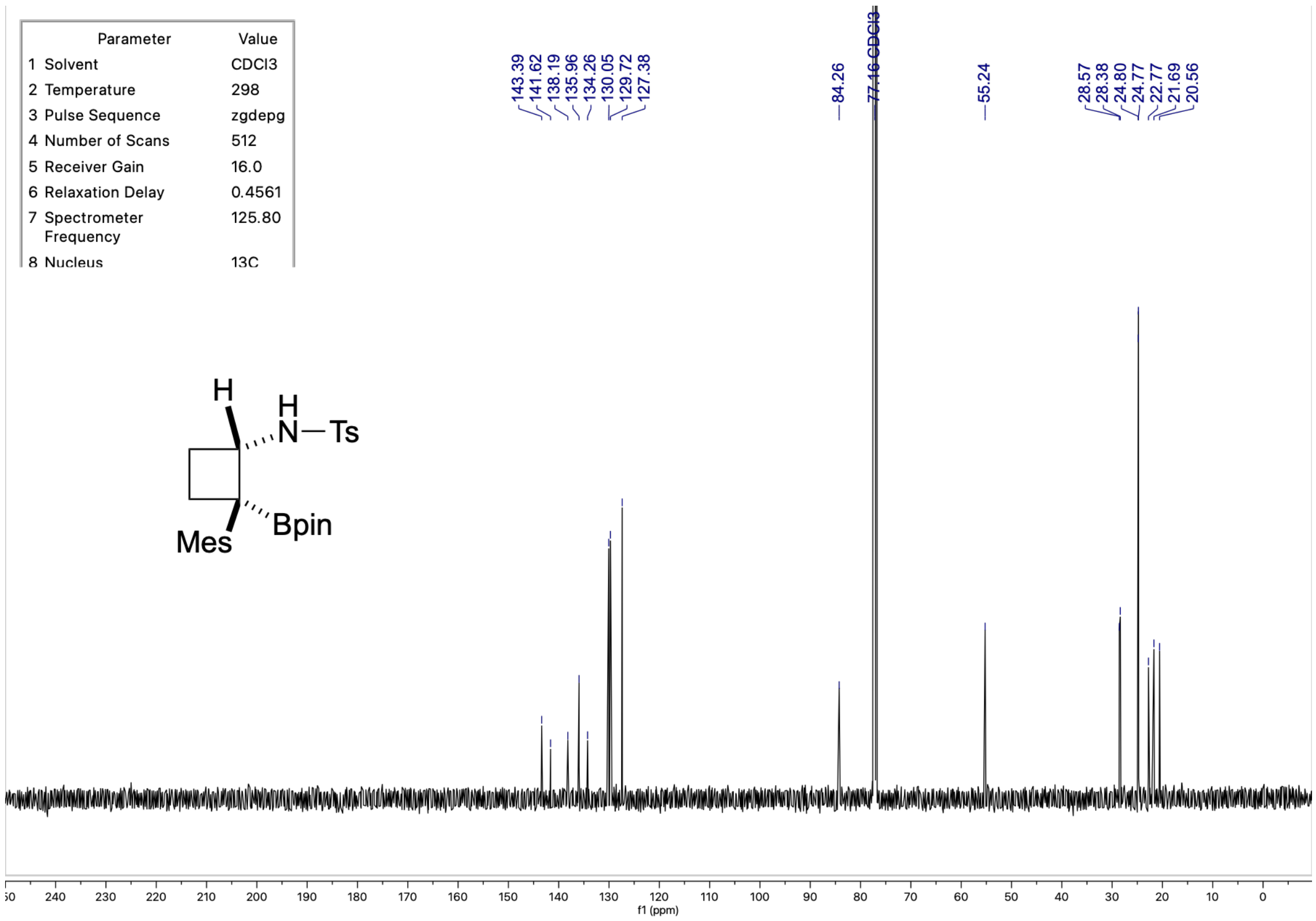
Parameter Value
1 Solvent CDCl3
2 Temperature 298
3 Pulse Sequence zgdepg
4 Number of Scans 512
5 Receiver Gain 16.0
6 Relaxation Delay 0.4561
7 Spectrometer Frequency 125.80
8 Nucleus 13C
143.39
141.62
138.19
135.96
134.26
130.05
129.72
127.38
84.26
77.16 CDCl3
55.24
28.57
28.38
24.80
24.77
22.77
21.69
20.56
H
H
N—Ts
Bpin
Mes
f1 (ppm)

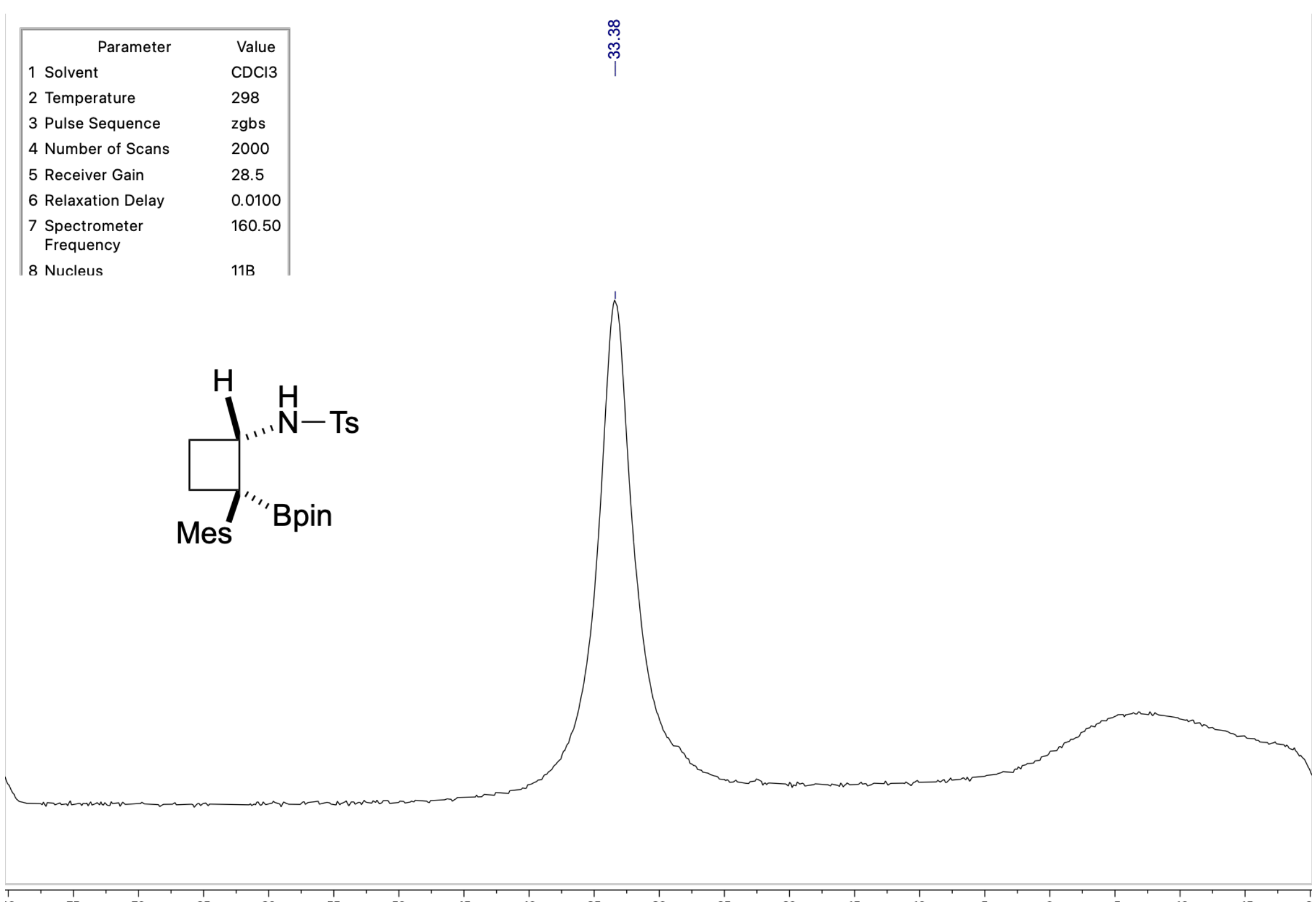
Parameter Value
1 Solvent CDCl3
2 Temperature 298
3 Pulse Sequence zgbs
4 Number of Scans 2000
5 Receiver Gain 28.5
6 Relaxation Delay 0.0100
7 Spectrometer Frequency 160.50
8 Nucleus 11B
33.38
H
H
N—Ts
Bpin
Mes
f1 (ppm)

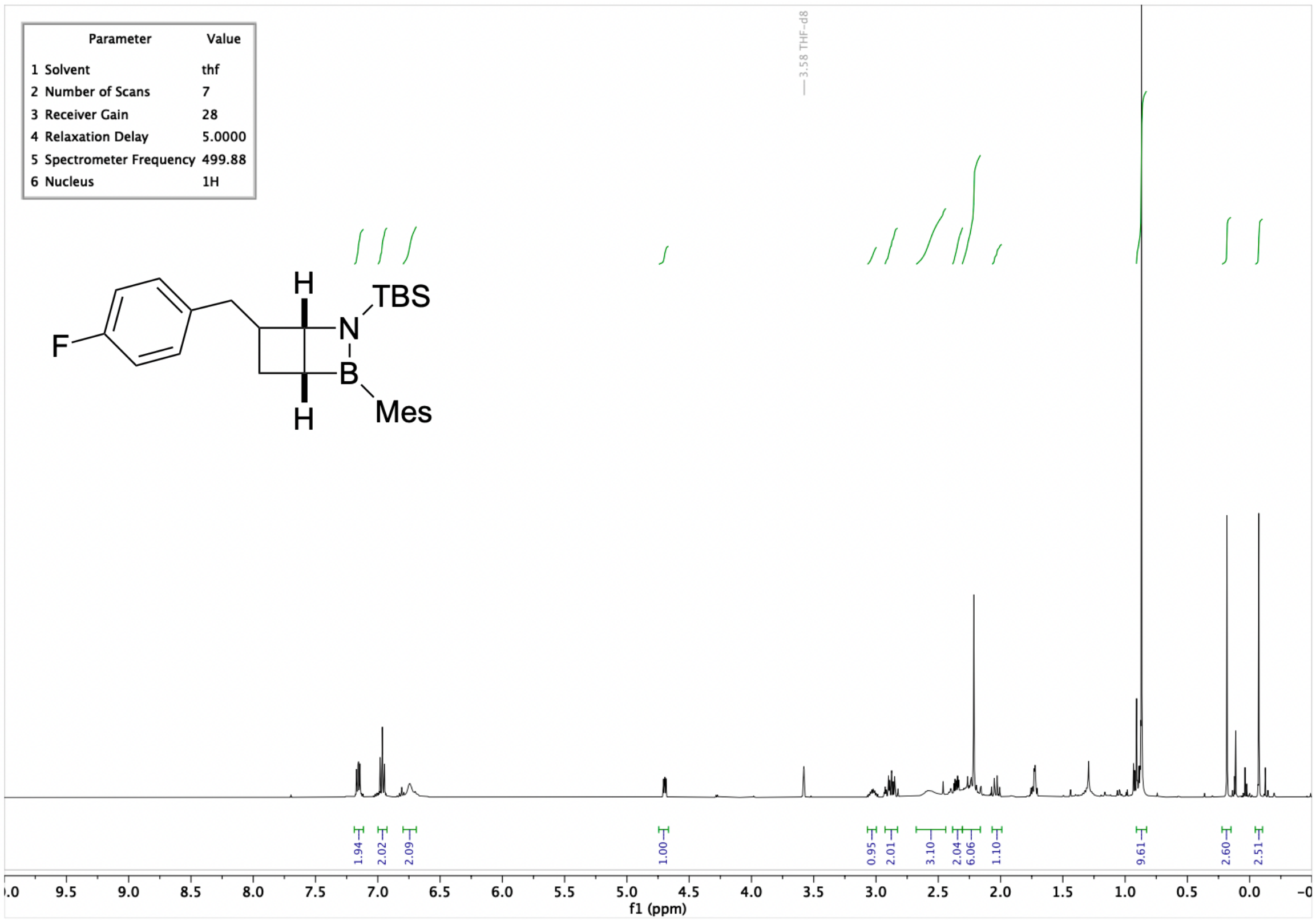
Parameter Value
1 Solvent thf
2 Number of Scans 7
3 Receiver Gain 28
4 Relaxation Delay 5.0000
5 Spectrometer Frequency 499.88
6 Nucleus 1H
3.58 THF-d8
TBS
N
B
H
H
Mes
F
1.94
2.02
2.09
1.00
0.95
2.01
3.10
2.04
6.06
1.10
9.61
2.60
2.51
f1 (ppm)

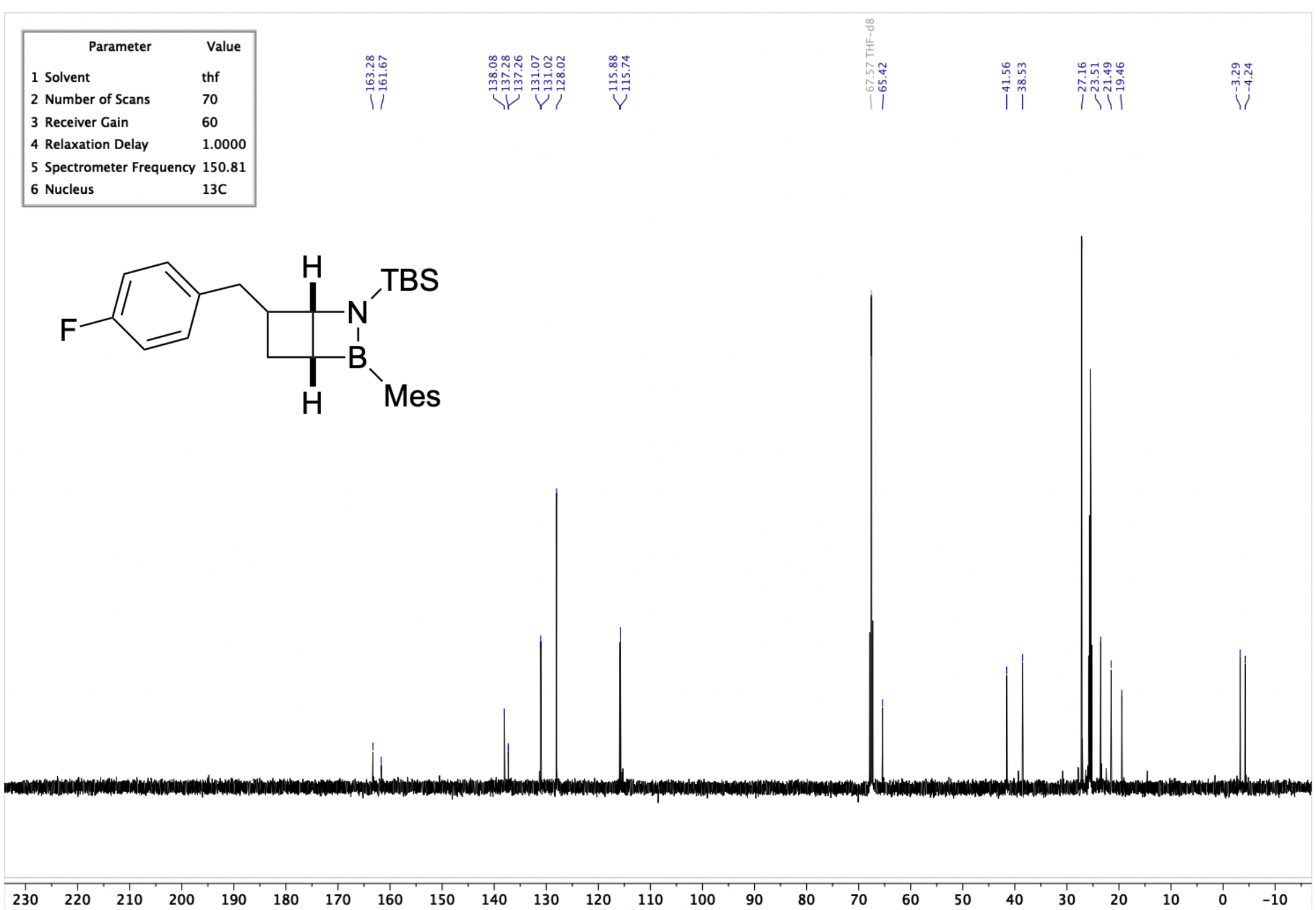
Parameter Value
1 Solvent thf
2 Number of Scans 70
3 Receiver Gain 60
4 Relaxation Delay 1.0000
5 Spectrometer Frequency 150.81
6 Nucleus 13C
163.28
161.67
138.08
137.28
137.26
131.07
131.02
128.02
115.88
115.74
67.57 THF-d8
65.42
41.56
38.53
27.16
23.51
21.49
19.46
-3.29
-4.24
TBS
N
B
H
H
Mes
F
f1 (ppm)

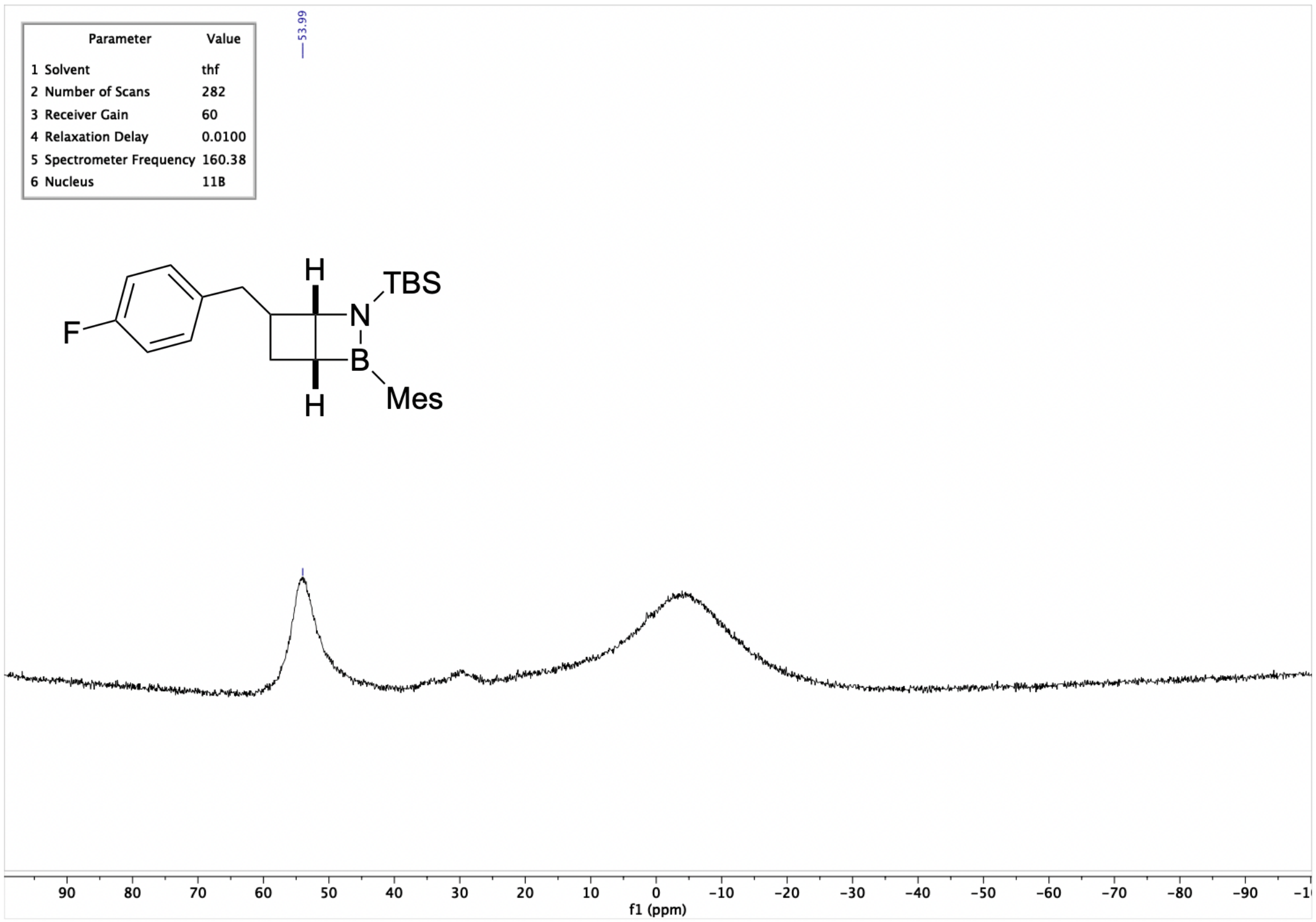
Parameter Value
1 Solvent thf
2 Number of Scans 282
3 Receiver Gain 60
4 Relaxation Delay 0.0100
5 Spectrometer Frequency 160.38
6 Nucleus 11B
53.99
H
TBS
N
F
B
H
Mes
f1 (ppm)

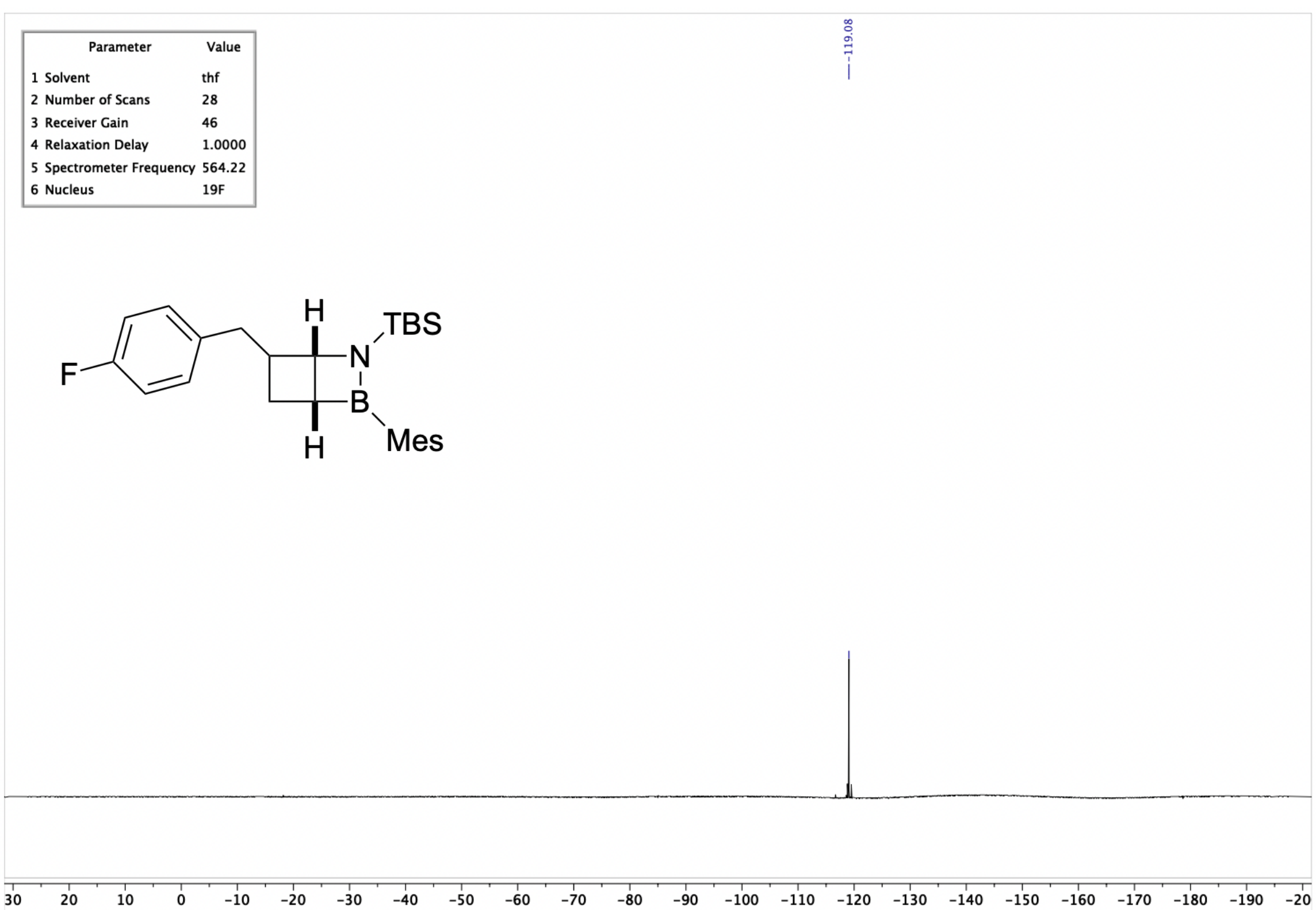
Parameter Value
1 Solvent thf
2 Number of Scans 28
3 Receiver Gain 46
4 Relaxation Delay 1.0000
5 Spectrometer Frequency 564.22
6 Nucleus 19F
-119.08
H
TBS
N
F
B
H
Mes
f1 (ppm)

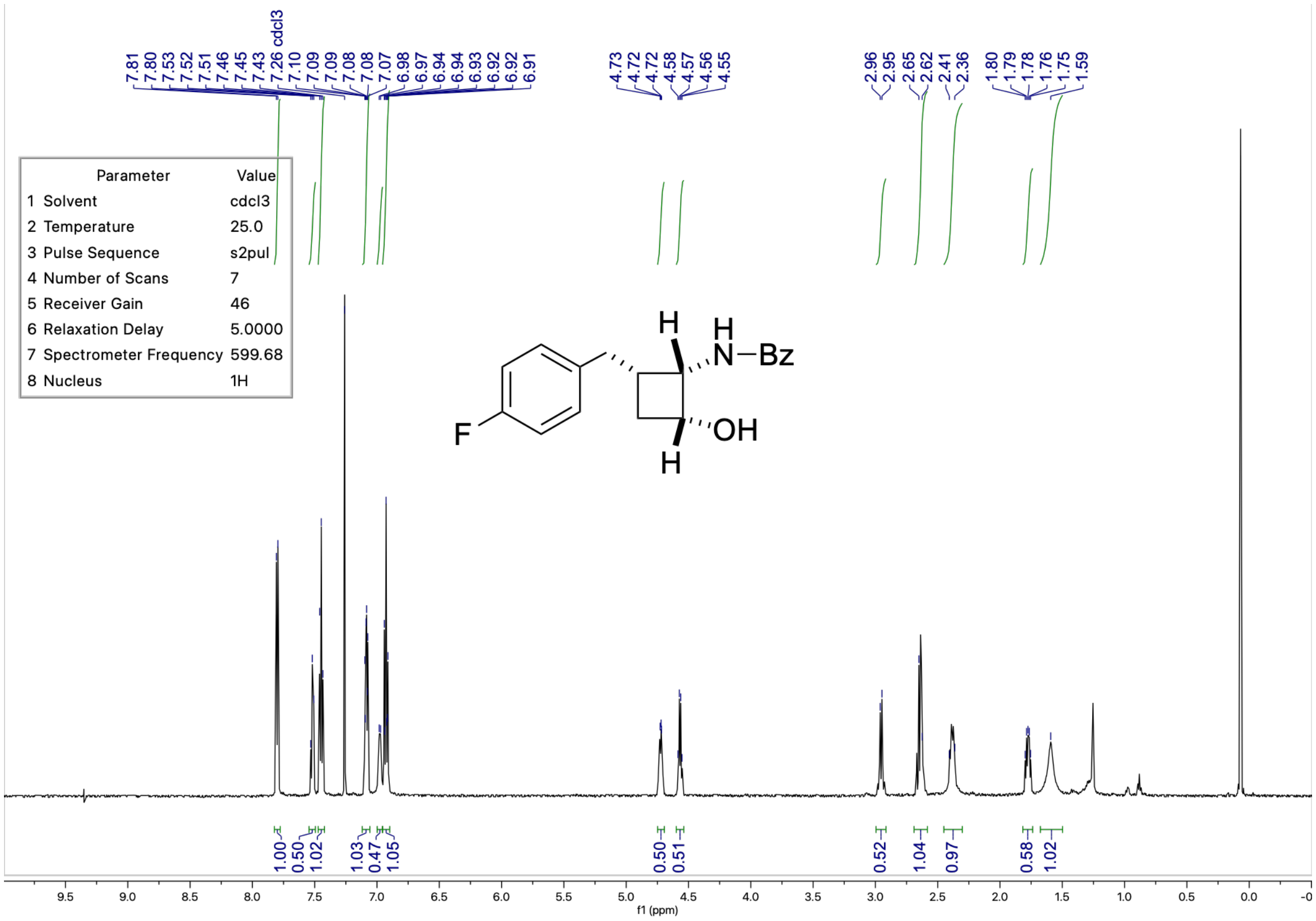

Parameter	Value
1 Solvent	cdcl3
2 Temperature	25.0
3 Pulse Sequence	s2pul
4 Number of Scans	7
5 Receiver Gain	46
6 Relaxation Delay	5.0000
7 Spectrometer Frequency	599.68
8 Nucleus	1H

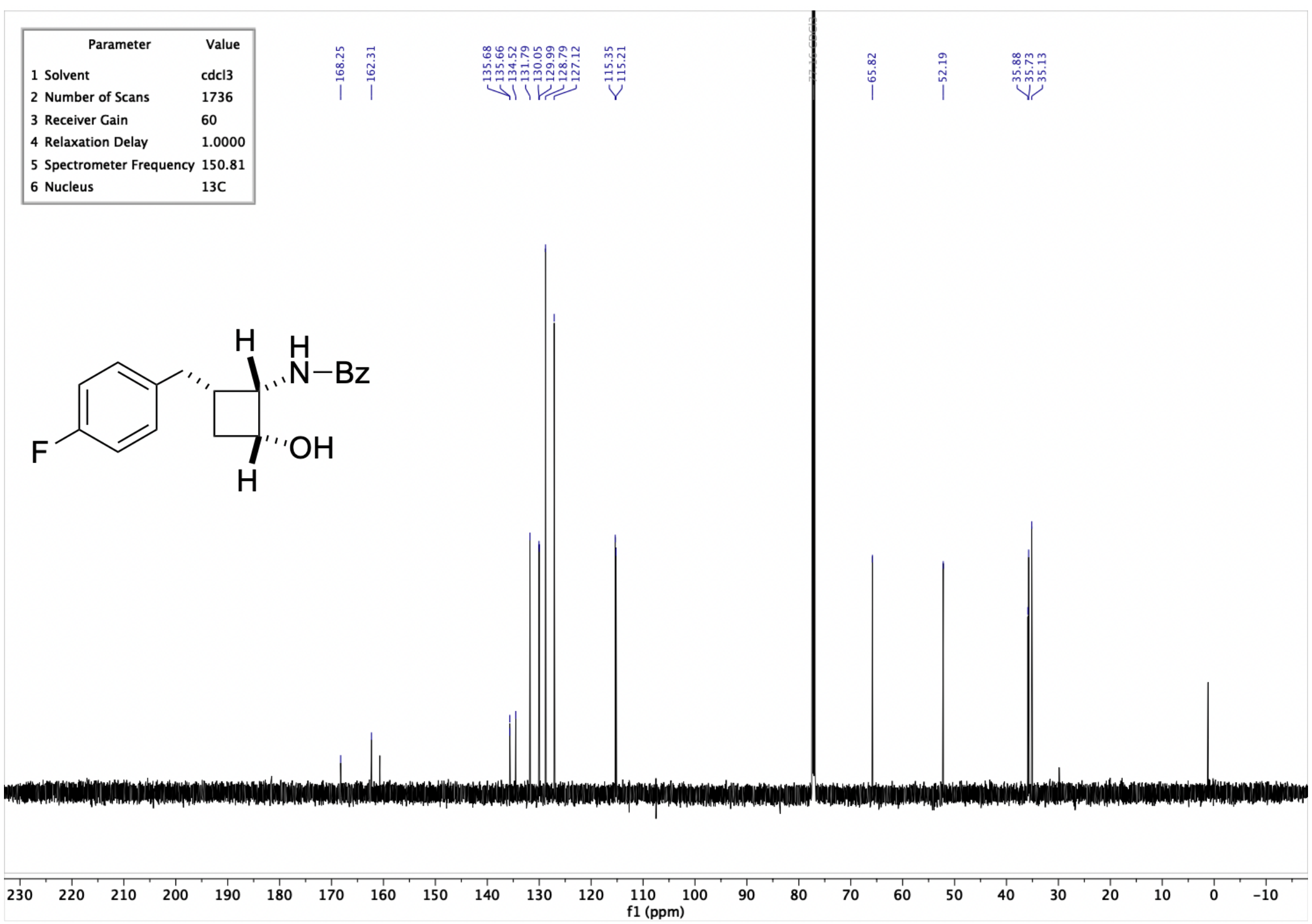

Parameter	Value
1 Solvent	cdcl3
2 Number of Scans	1736
3 Receiver Gain	60
4 Relaxation Delay	1.0000
5 Spectrometer Frequency	150.81
6 Nucleus	13C

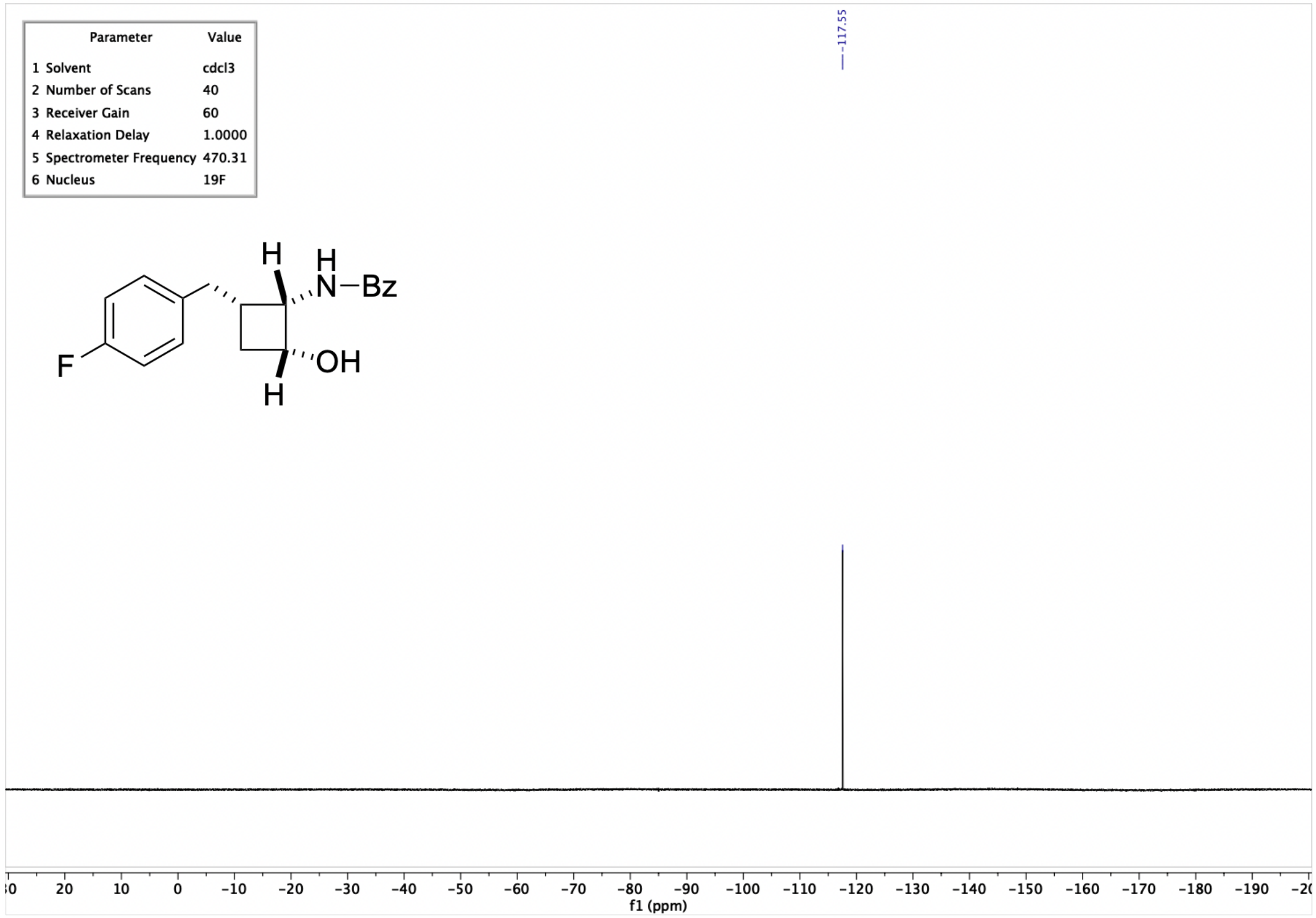
Parameter Value
1 Solvent cdcl3
2 Number of Scans 40
3 Receiver Gain 60
4 Relaxation Delay 1.0000
5 Spectrometer Frequency 470.31
6 Nucleus 19F
—117.55
H
H
N–Bz
F
OH
H
f1 (ppm)

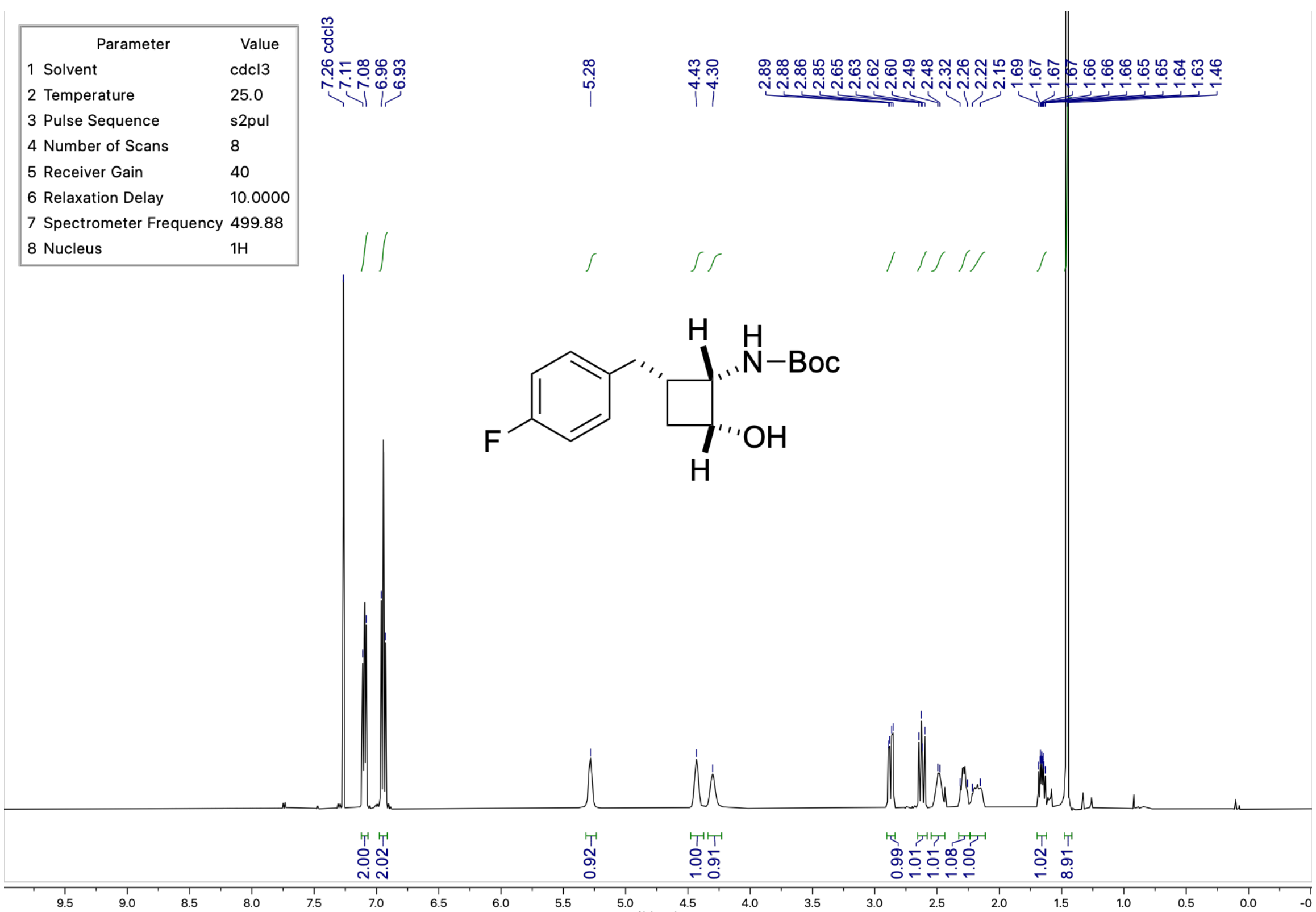
Parameter Value
1 Solvent cdcl3
2 Temperature 25.0
3 Pulse Sequence s2pul
4 Number of Scans 8
5 Receiver Gain 40
6 Relaxation Delay 10.0000
7 Spectrometer Frequency 499.88
8 Nucleus 1H
H
H
N–Boc
F
OH
H
f1 (ppm)

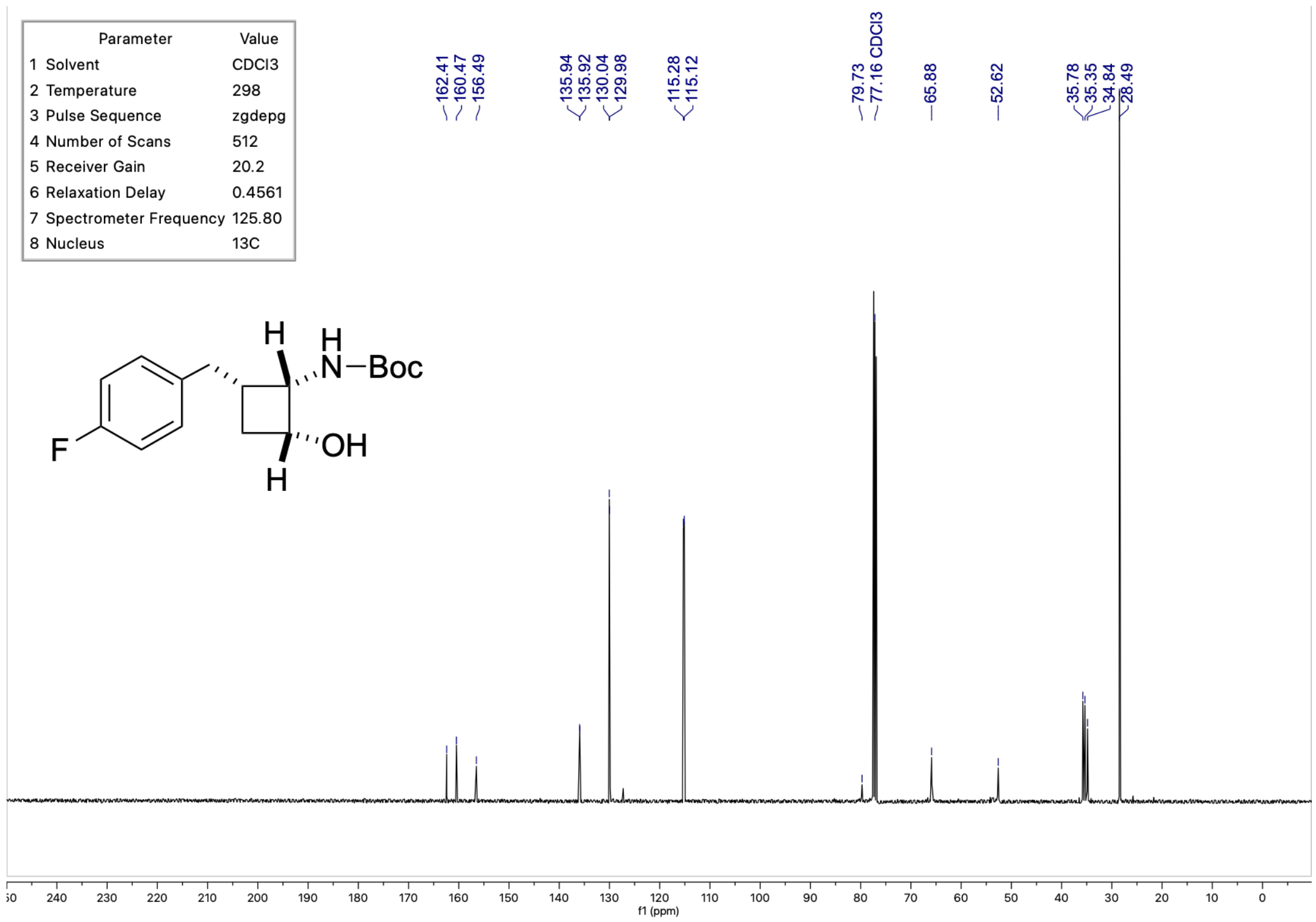

Parameter Value
1 Solvent CDCl3
2 Temperature 298
3 Pulse Sequence zgdepg
4 Number of Scans 512
5 Receiver Gain 20.2
6 Relaxation Delay 0.4561
7 Spectrometer Frequency 125.80
8 Nucleus 13C
162.41
160.47
156.49
135.94
135.92
130.04
129.98
115.28
115.12
79.73
77.16 CDCl3
65.88
52.62
35.78
35.35
34.84
28.49
F
H
H
N−Boc
OH
H
f1 (ppm)

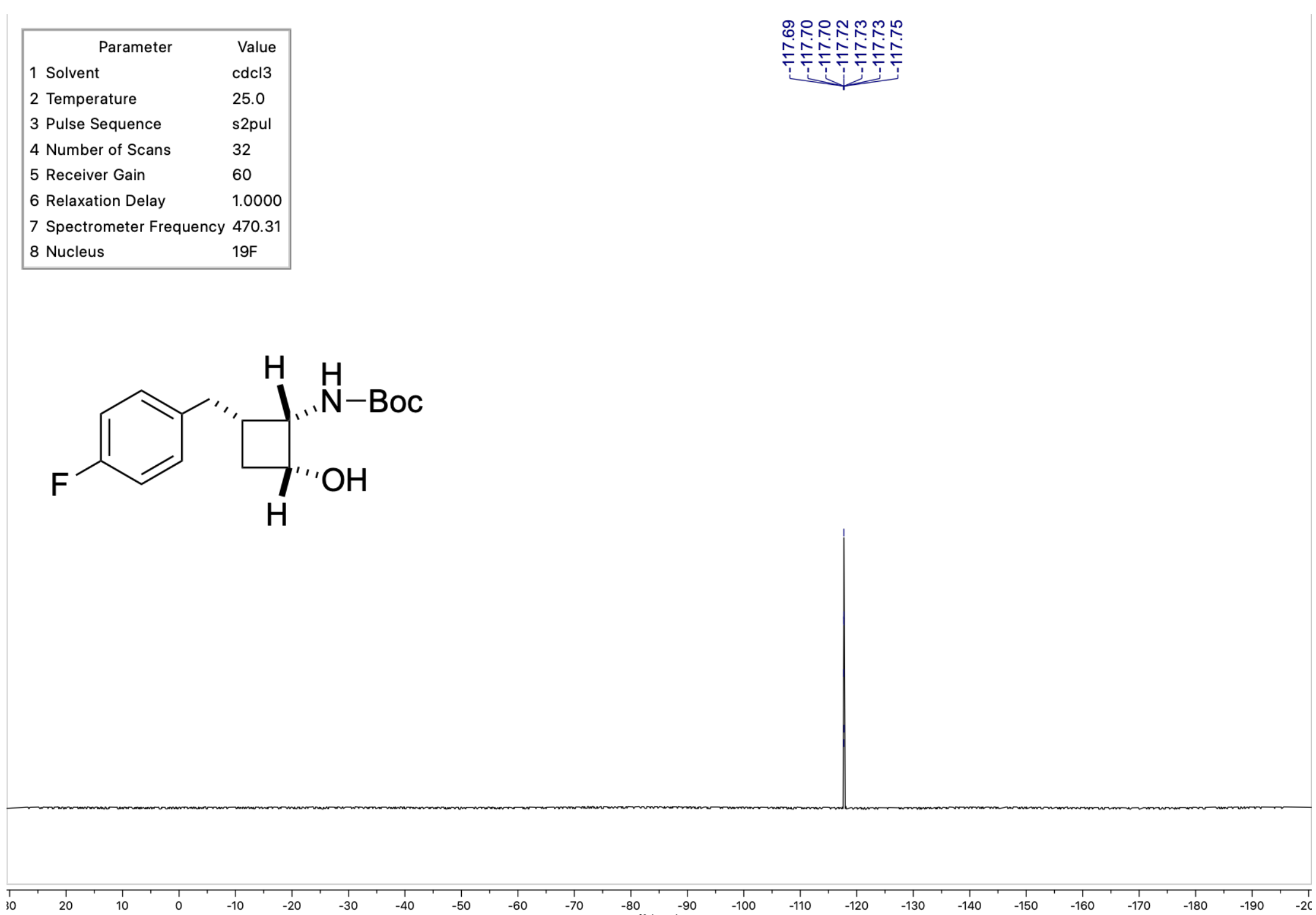

Parameter Value
1 Solvent cdcl3
2 Temperature 25.0
3 Pulse Sequence s2pul
4 Number of Scans 32
5 Receiver Gain 60
6 Relaxation Delay 1.0000
7 Spectrometer Frequency 470.31
8 Nucleus 19F
-117.69
-117.70
-117.70
-117.72
-117.73
-117.73
-117.75
F
H
H
N−Boc
OH
H
f1 (ppm)

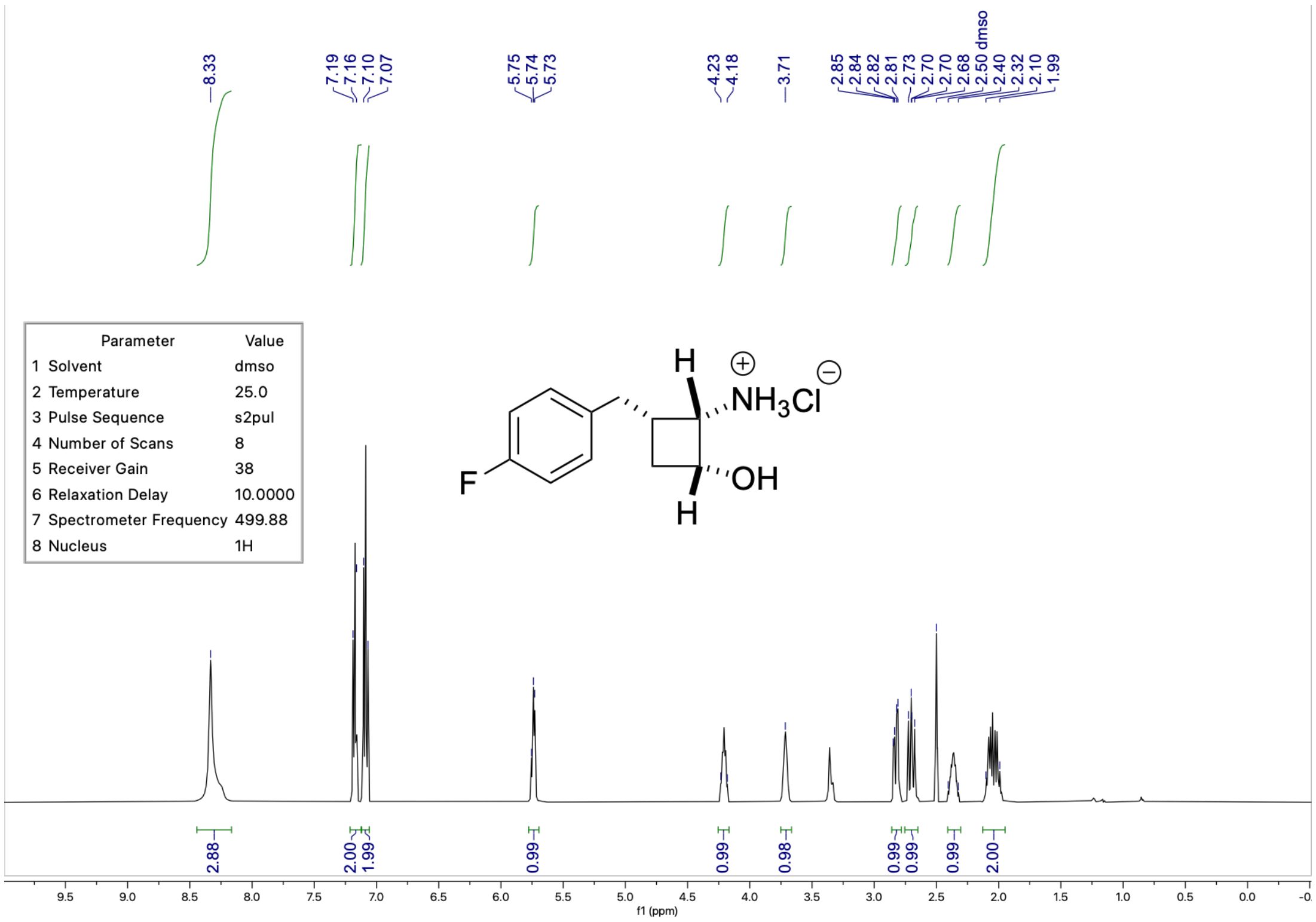

8.33
7.19
7.16
7.10
7.07
5.75
5.74
5.73
4.23
4.18
3.71
2.85
2.84
2.82
2.81
2.73
2.70
2.70
2.68
2.50 dmso
2.40
2.32
2.10
1.99
Parameter Value
1 Solvent dmso
2 Temperature 25.0
3 Pulse Sequence s2pul
4 Number of Scans 8
5 Receiver Gain 38
6 Relaxation Delay 10.0000
7 Spectrometer Frequency 499.88
8 Nucleus 1H
H
NH3Cl
OH
H
F
2.88
2.00
1.99
0.99
0.99
0.98
0.99
0.99
0.99
2.00
f1 (ppm)

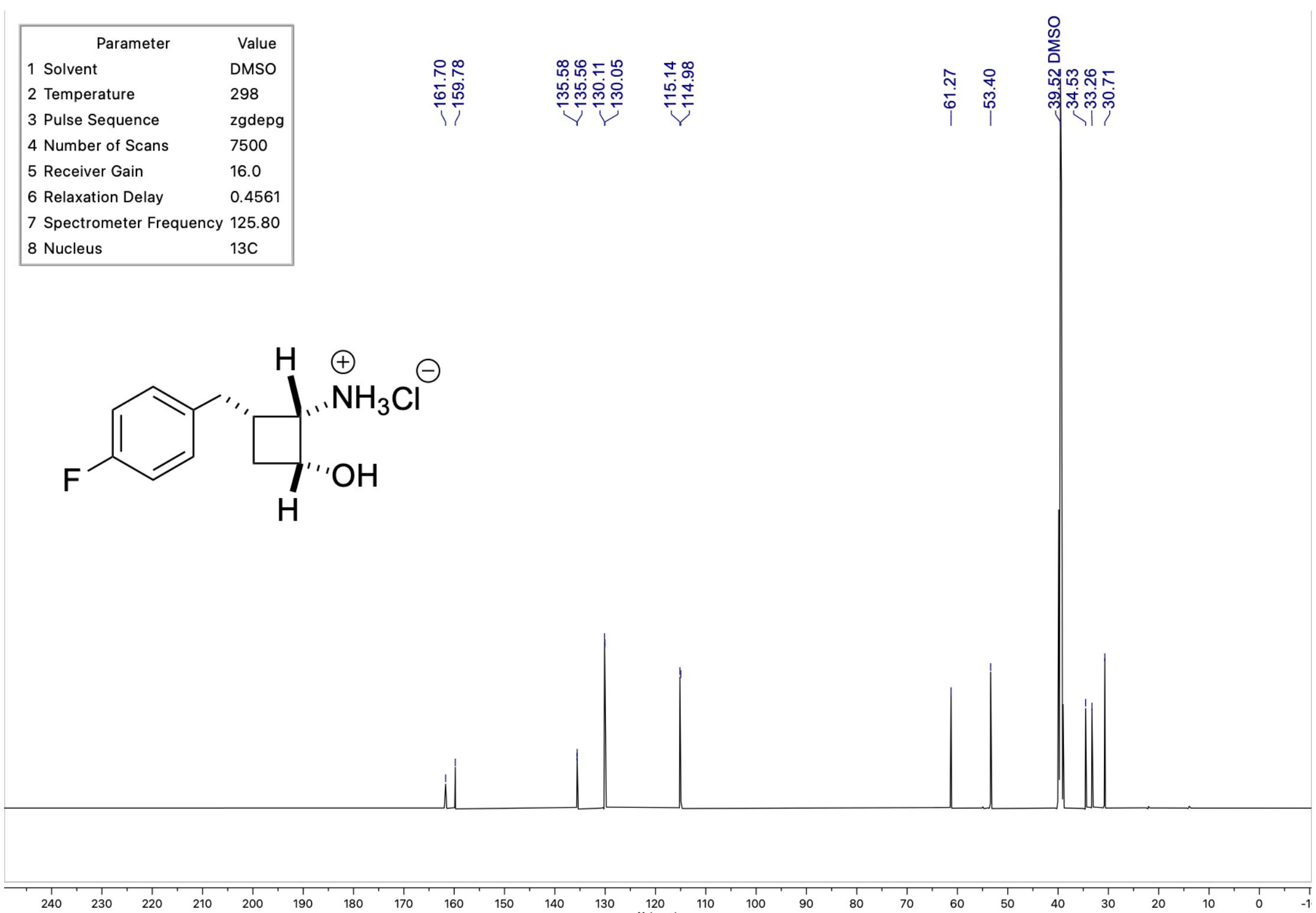

Parameter Value
1 Solvent DMSO
2 Temperature 298
3 Pulse Sequence zgdepg
4 Number of Scans 7500
5 Receiver Gain 16.0
6 Relaxation Delay 0.4561
7 Spectrometer Frequency 125.80
8 Nucleus 13C
161.70
159.78
135.58
135.56
130.11
130.05
115.14
114.98
61.27
53.40
39.52 DMSO
34.53
33.26
30.71
H
NH3Cl
OH
H
F
f1 (ppm)

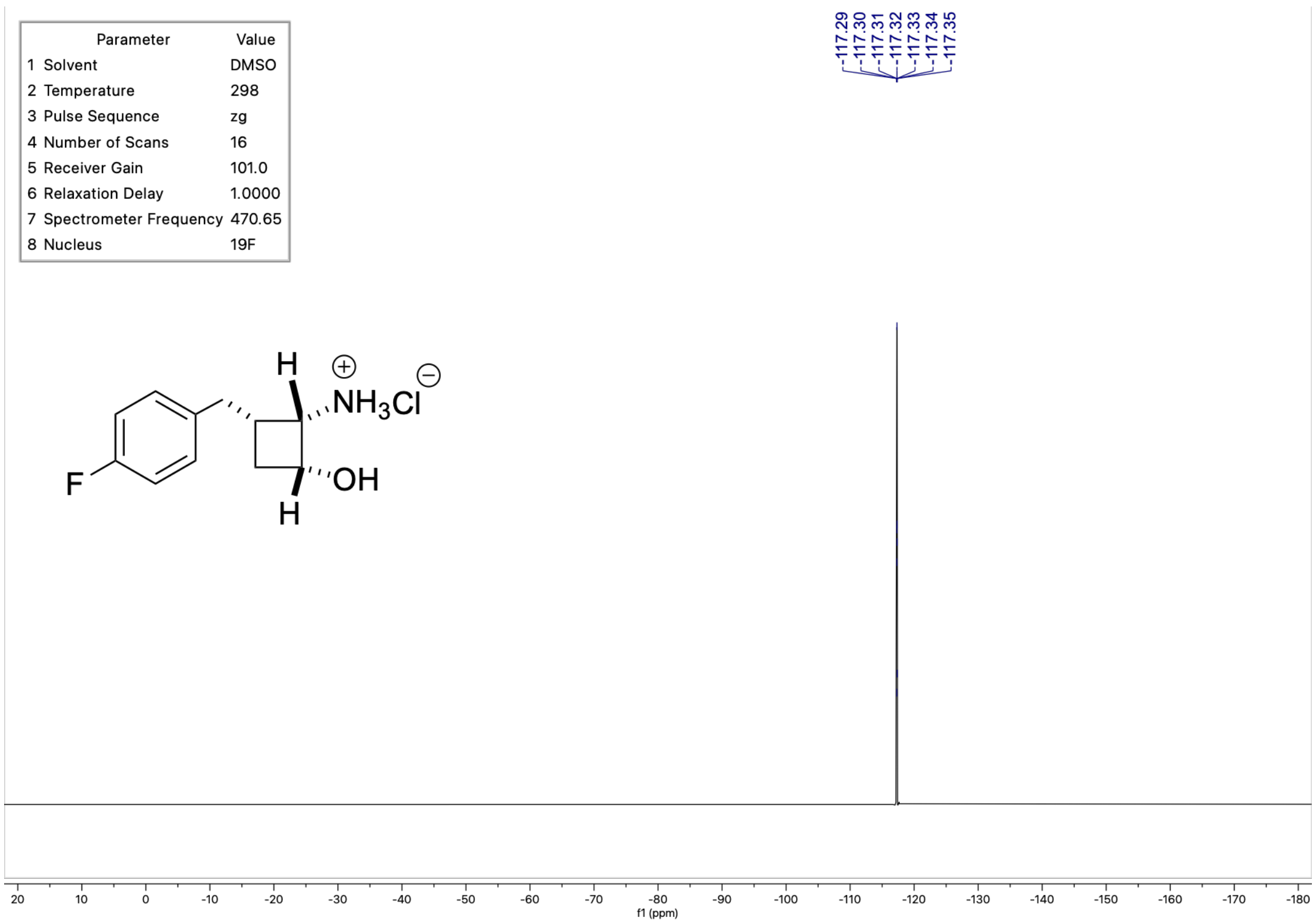

-117.29
-117.30
-117.31
-117.32
-117.33
-117.34
-117.35
Parameter Value
1 Solvent DMSO
2 Temperature 298
3 Pulse Sequence zg
4 Number of Scans 16
5 Receiver Gain 101.0
6 Relaxation Delay 1.0000
7 Spectrometer Frequency 470.65
8 Nucleus 19F
H
NH3Cl
F
OH
H
f1 (ppm)

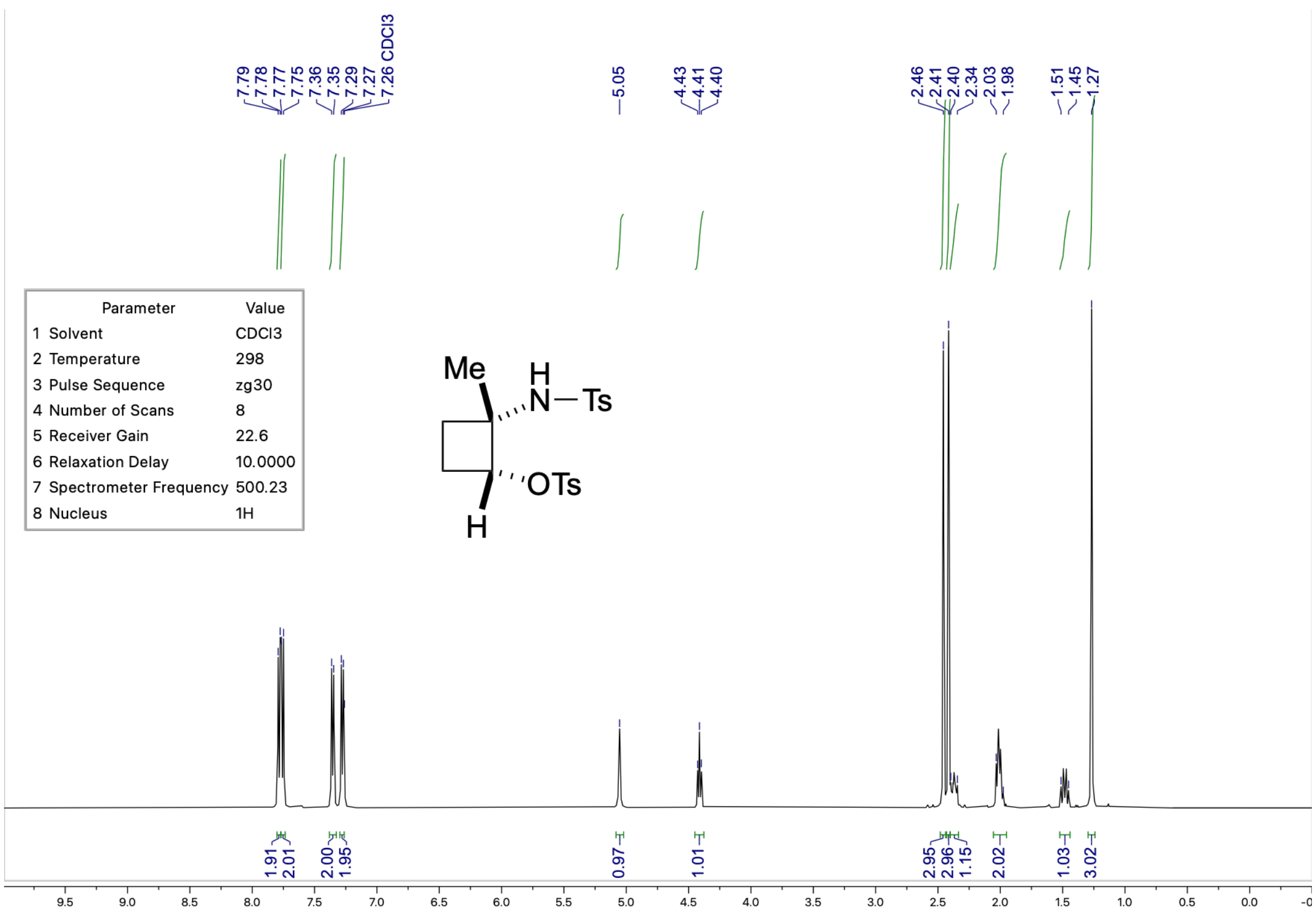

7.79
7.78
7.77
7.75
7.36
7.35
7.29
7.27
7.26 CDCl3
5.05
4.43
4.41
4.40
2.46
2.41
2.40
2.34
2.03
1.98
1.51
1.45
1.27
Parameter Value
1 Solvent CDCl3
2 Temperature 298
3 Pulse Sequence zg30
4 Number of Scans 8
5 Receiver Gain 22.6
6 Relaxation Delay 10.0000
7 Spectrometer Frequency 500.23
8 Nucleus 1H
Me
H
N—Ts
OTs
H
1.91
2.01
2.00
1.95
0.97
1.01
2.95
2.96
1.15
2.02
1.03
3.02
f1 (ppm)

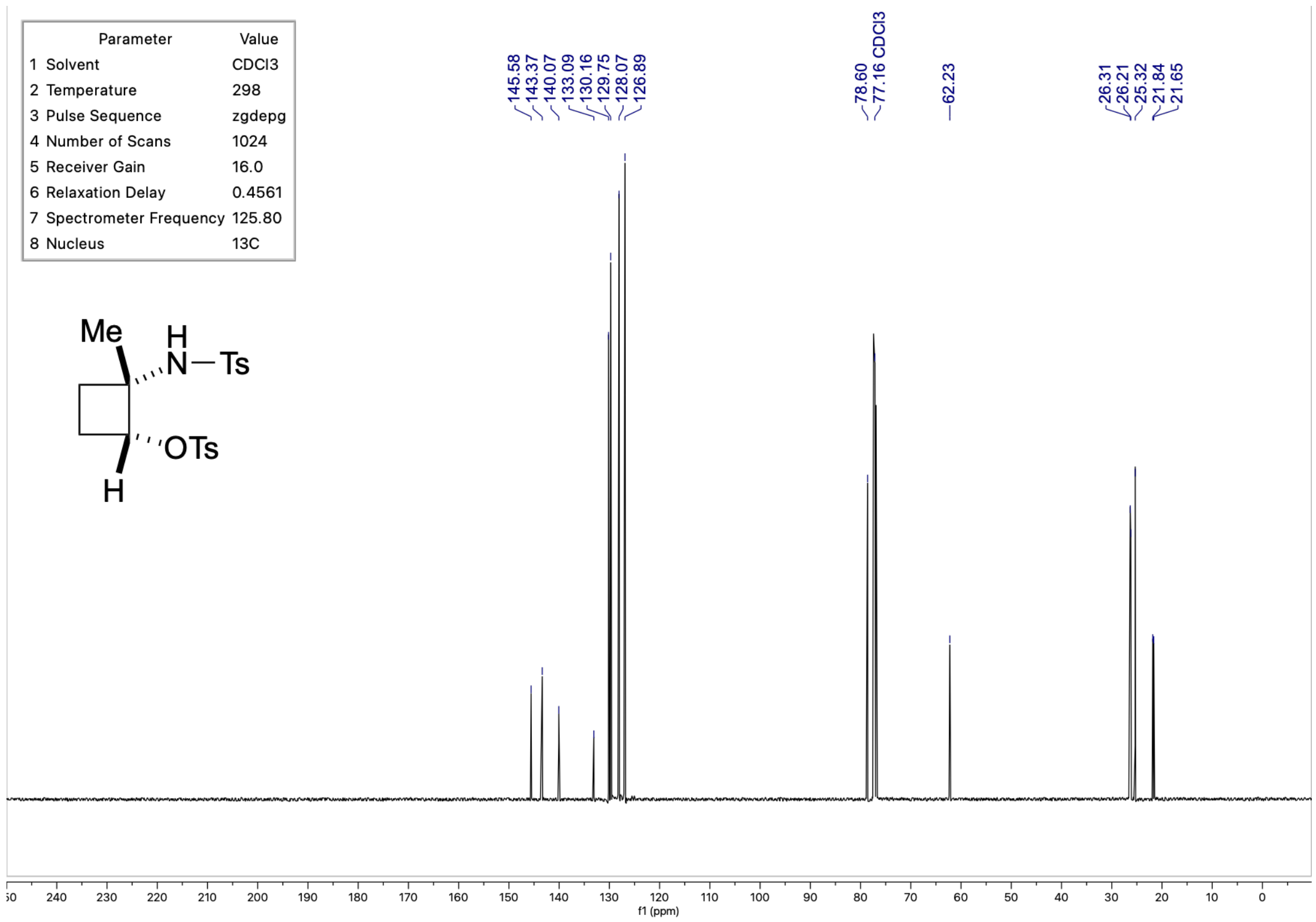
Parameter Value
1 Solvent CDCl3
2 Temperature 298
3 Pulse Sequence zgdepg
4 Number of Scans 1024
5 Receiver Gain 16.0
6 Relaxation Delay 0.4561
7 Spectrometer Frequency 125.80
8 Nucleus 13C
145.58 143.37 140.07 133.09 130.16 129.75 128.07 126.89
78.60 77.16 CDCl3
62.23
26.31 26.21 25.32 21.84 21.65
Me
H
N
Ts
OTs
H
f1 (ppm)

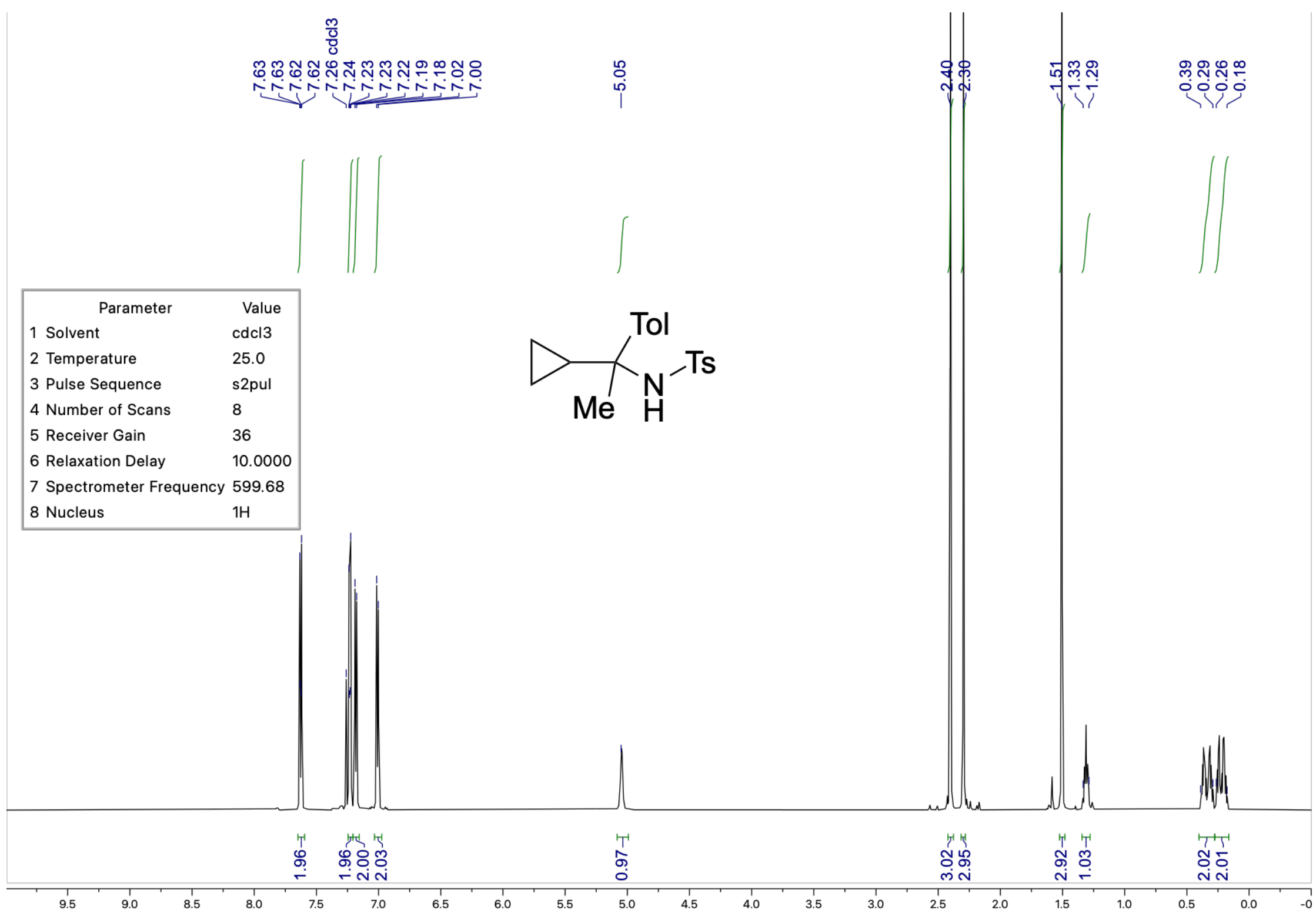
7.63 7.63 7.62 7.26 cdcl3 7.24 7.23 7.23 7.22 7.19 7.18 7.02 7.00
5.05
2.40 2.30
1.51 1.33 1.29
0.39 0.29 0.26 0.18
Parameter Value
1 Solvent cdcl3
2 Temperature 25.0
3 Pulse Sequence s2pul
4 Number of Scans 8
5 Receiver Gain 36
6 Relaxation Delay 10.0000
7 Spectrometer Frequency 599.68
8 Nucleus 1H
Tol
N
Ts
Me
H
1.96 1.96 2.00 2.03 0.97 3.02 2.95 2.92 1.03 2.02 2.01
f1 (ppm)

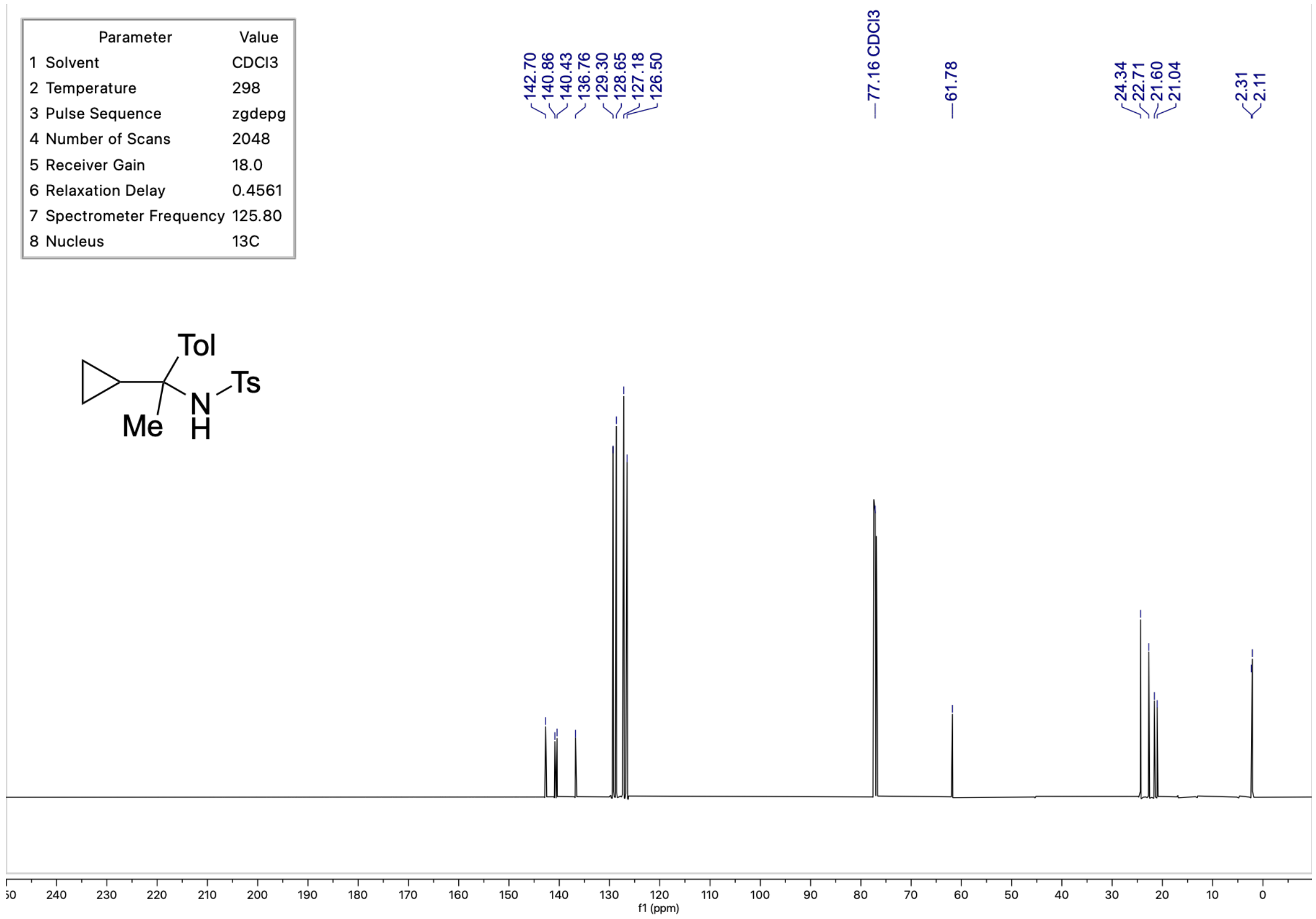
Parameter Value
1 Solvent CDCl3
2 Temperature 298
3 Pulse Sequence zgdepg
4 Number of Scans 2048
5 Receiver Gain 18.0
6 Relaxation Delay 0.4561
7 Spectrometer Frequency 125.80
8 Nucleus 13C
Tol
Ts
N
H
Me
142.70
140.86
140.43
136.76
129.30
128.65
127.18
126.50
77.16 CDCl3
61.78
24.34
22.71
21.60
21.04
2.31
2.11
f1 (ppm)

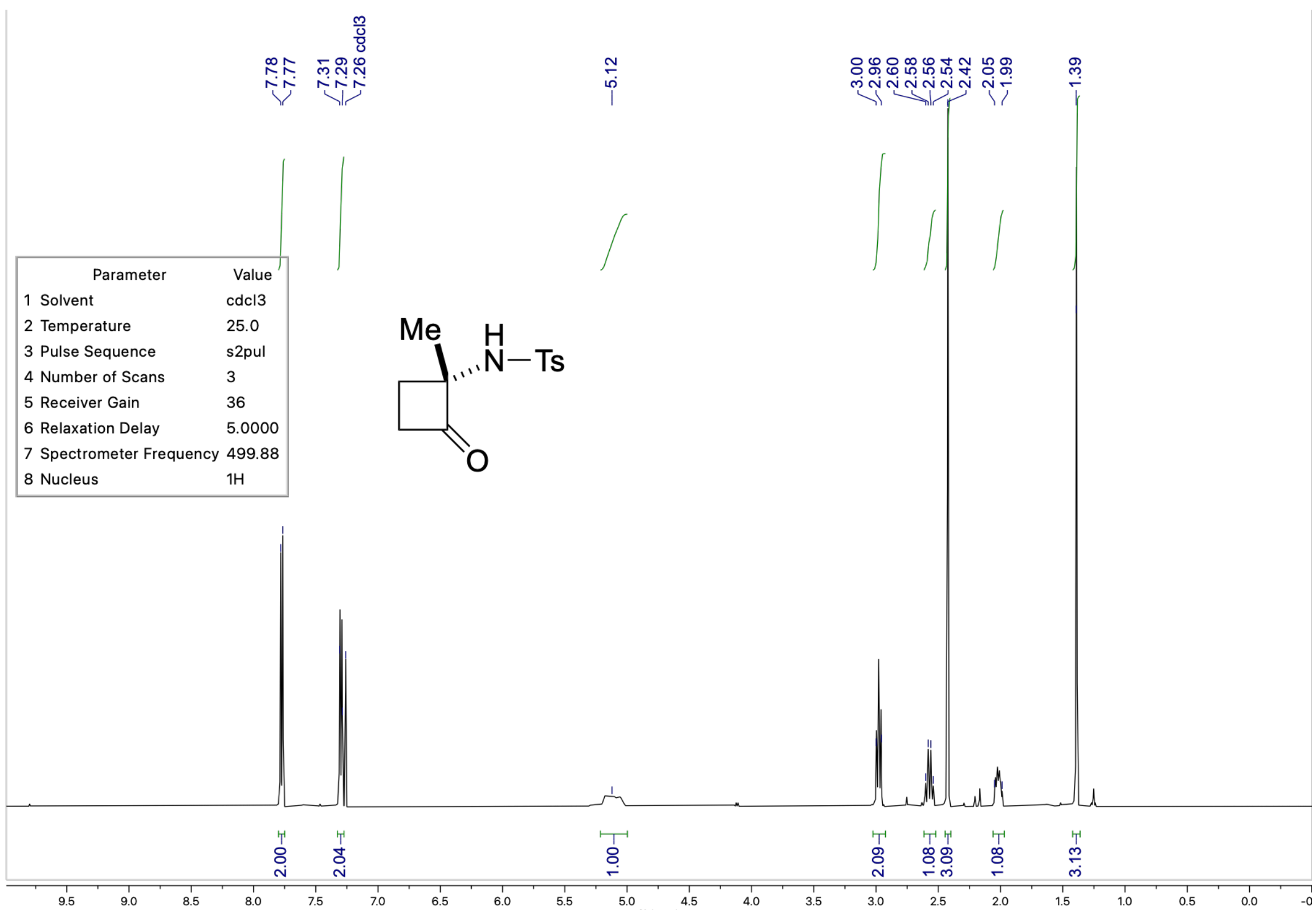
7.78
7.77
7.31
7.29
7.26 cdcl3
5.12
3.00
2.96
2.60
2.58
2.56
2.54
2.42
2.05
1.99
1.39
Parameter Value
1 Solvent cdcl3
2 Temperature 25.0
3 Pulse Sequence s2pul
4 Number of Scans 3
5 Receiver Gain 36
6 Relaxation Delay 5.0000
7 Spectrometer Frequency 499.88
8 Nucleus 1H
Me
H
N
Ts
O
2.00
2.04
1.00
2.09
1.08
3.09
1.08
3.13
f1 (ppm)

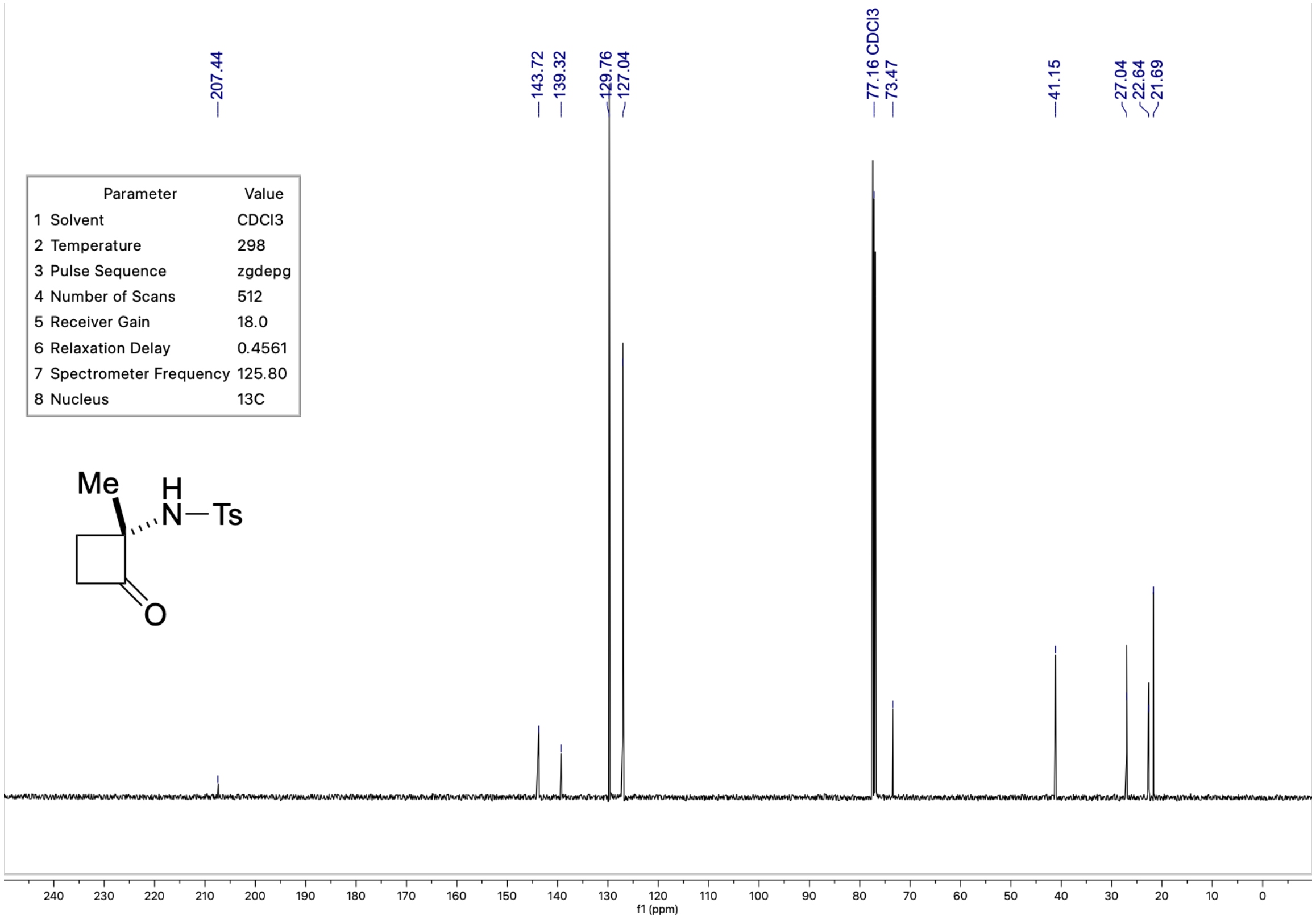

207.44
143.72
139.32
129.76
127.04
77.16 CDCl3
73.47
41.15
27.04
22.64
21.69
Parameter Value
1 Solvent CDCl3
2 Temperature 298
3 Pulse Sequence zgdepg
4 Number of Scans 512
5 Receiver Gain 18.0
6 Relaxation Delay 0.4561
7 Spectrometer Frequency 125.80
8 Nucleus 13C
Me
H
N—Ts
O
f1 (ppm)

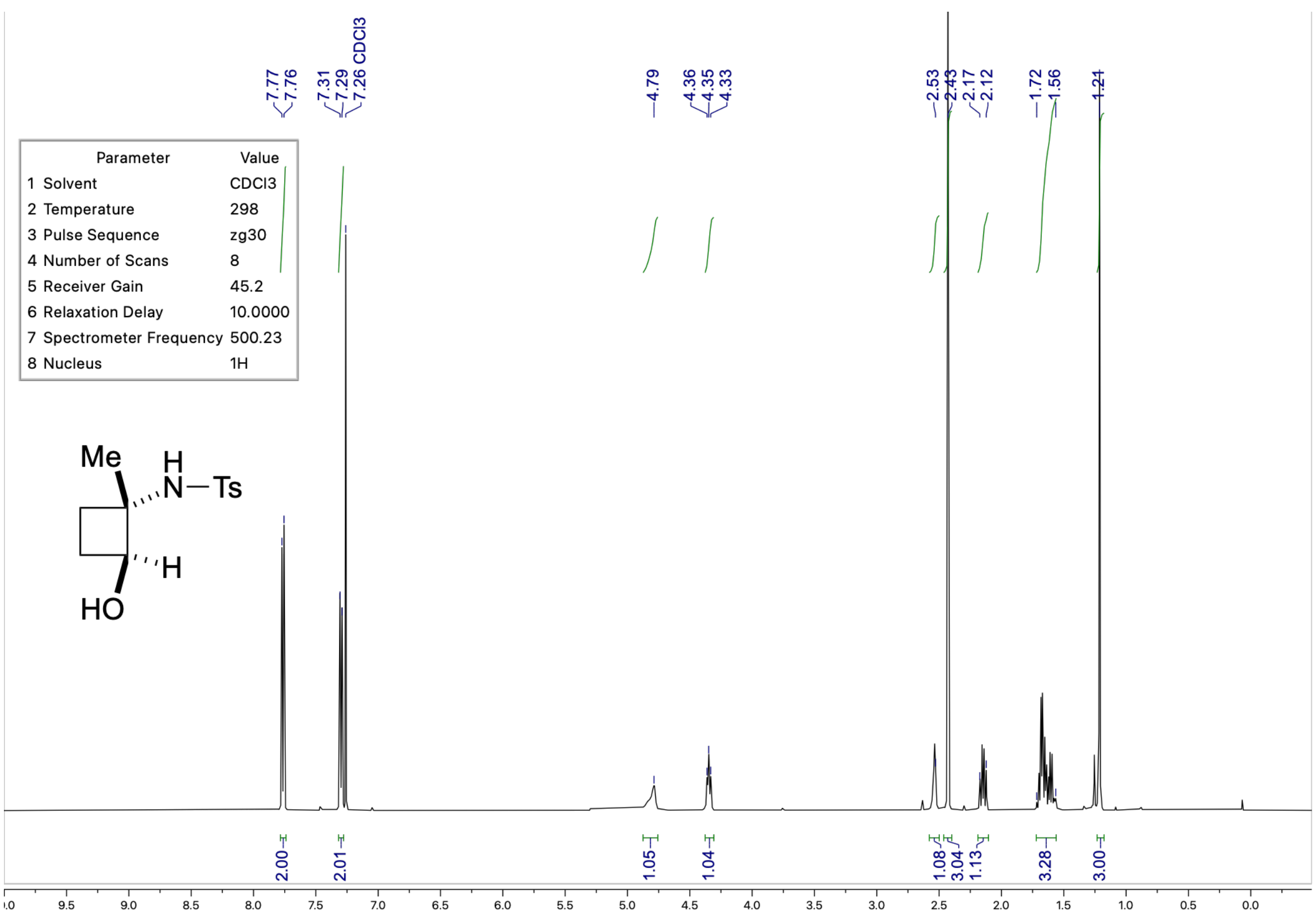

7.77
7.76
7.31
7.29
7.26 CDCl3
4.79
4.36
4.35
4.33
2.53
2.43
2.17
2.12
1.72
1.56
1.21
Parameter Value
1 Solvent CDCl3
2 Temperature 298
3 Pulse Sequence zg30
4 Number of Scans 8
5 Receiver Gain 45.2
6 Relaxation Delay 10.0000
7 Spectrometer Frequency 500.23
8 Nucleus 1H
Me
H
N—Ts
H
HO
2.00
2.01
1.05
1.04
1.08
3.04
1.13
3.28
3.00
f1 (ppm)

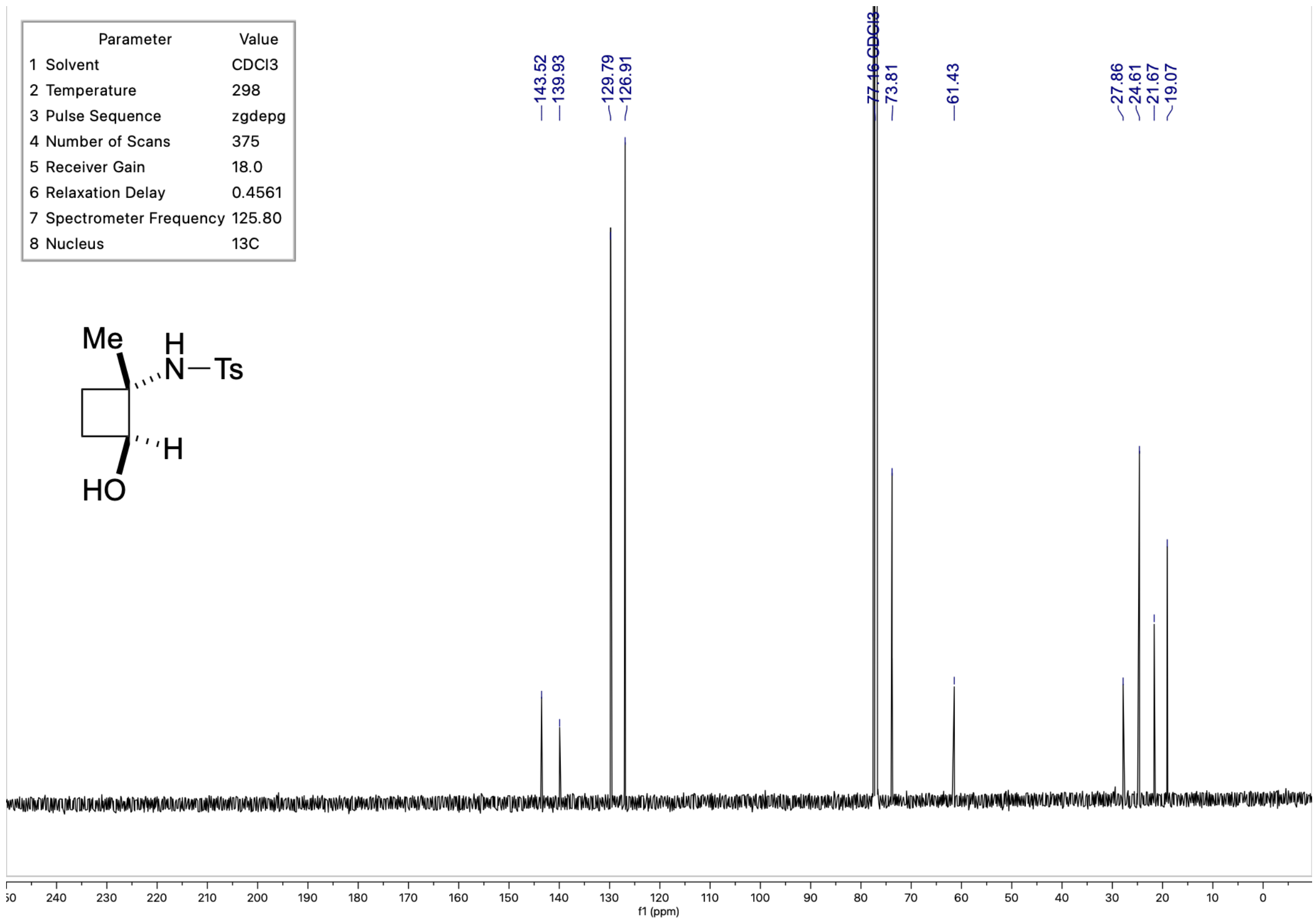
Parameter Value
1 Solvent CDCl3
2 Temperature 298
3 Pulse Sequence zgdepg
4 Number of Scans 375
5 Receiver Gain 18.0
6 Relaxation Delay 0.4561
7 Spectrometer Frequency 125.80
8 Nucleus 13C
143.52
139.93
129.79
126.91
77.16 CDCl3
73.81
61.43
27.86
24.61
21.67
19.07
Me
H
N—Ts
H
HO
f1 (ppm)

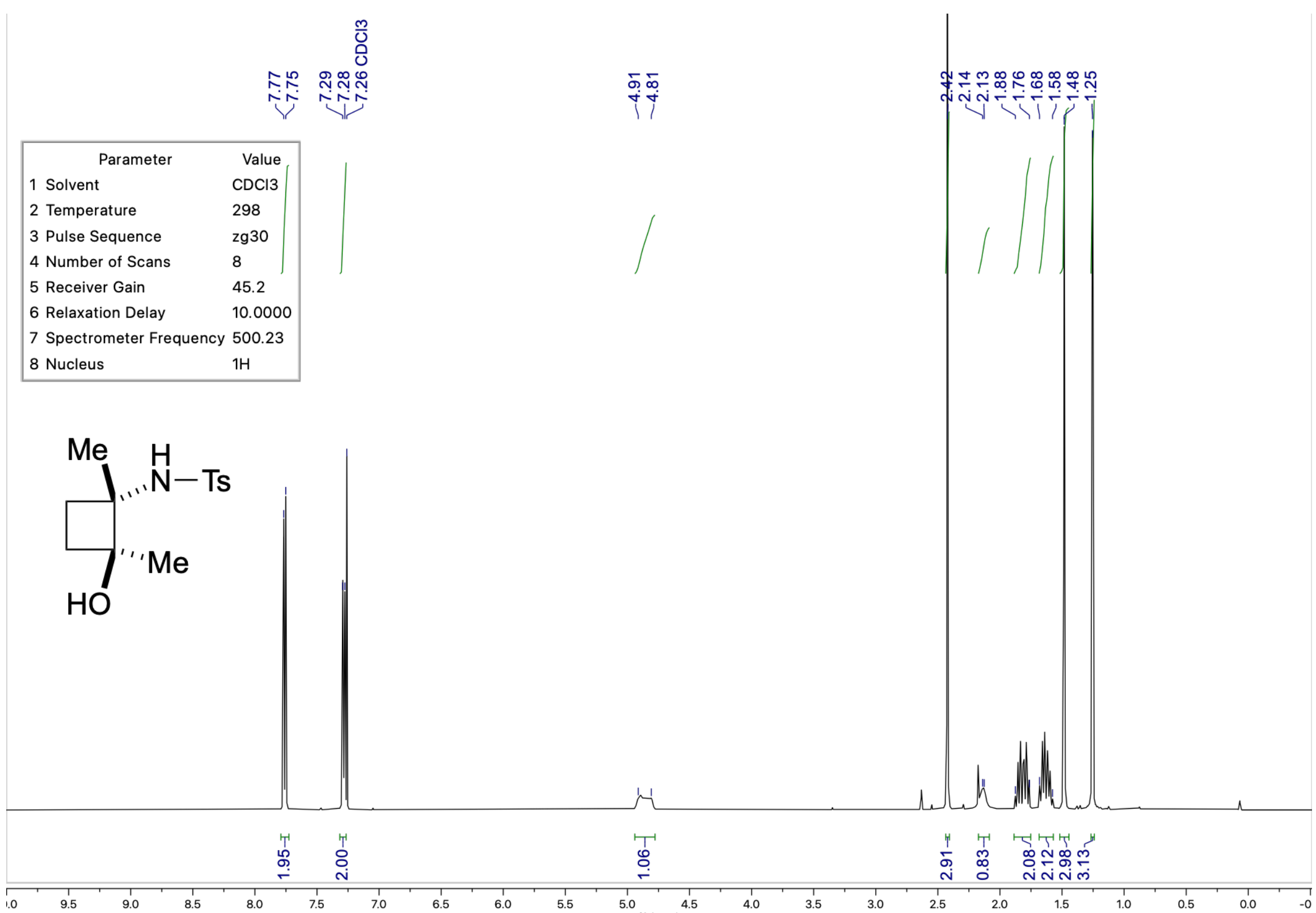
7.77
7.75
7.29
7.28
7.26 CDCl3
4.91
4.81
2.42
2.14
2.13
1.88
1.76
1.68
1.58
1.48
1.25
Parameter Value
1 Solvent CDCl3
2 Temperature 298
3 Pulse Sequence zg30
4 Number of Scans 8
5 Receiver Gain 45.2
6 Relaxation Delay 10.0000
7 Spectrometer Frequency 500.23
8 Nucleus 1H
Me
H
N—Ts
Me
HO
1.95
2.00
1.06
2.91
0.83
2.08
2.12
2.98
3.13
f1 (ppm)

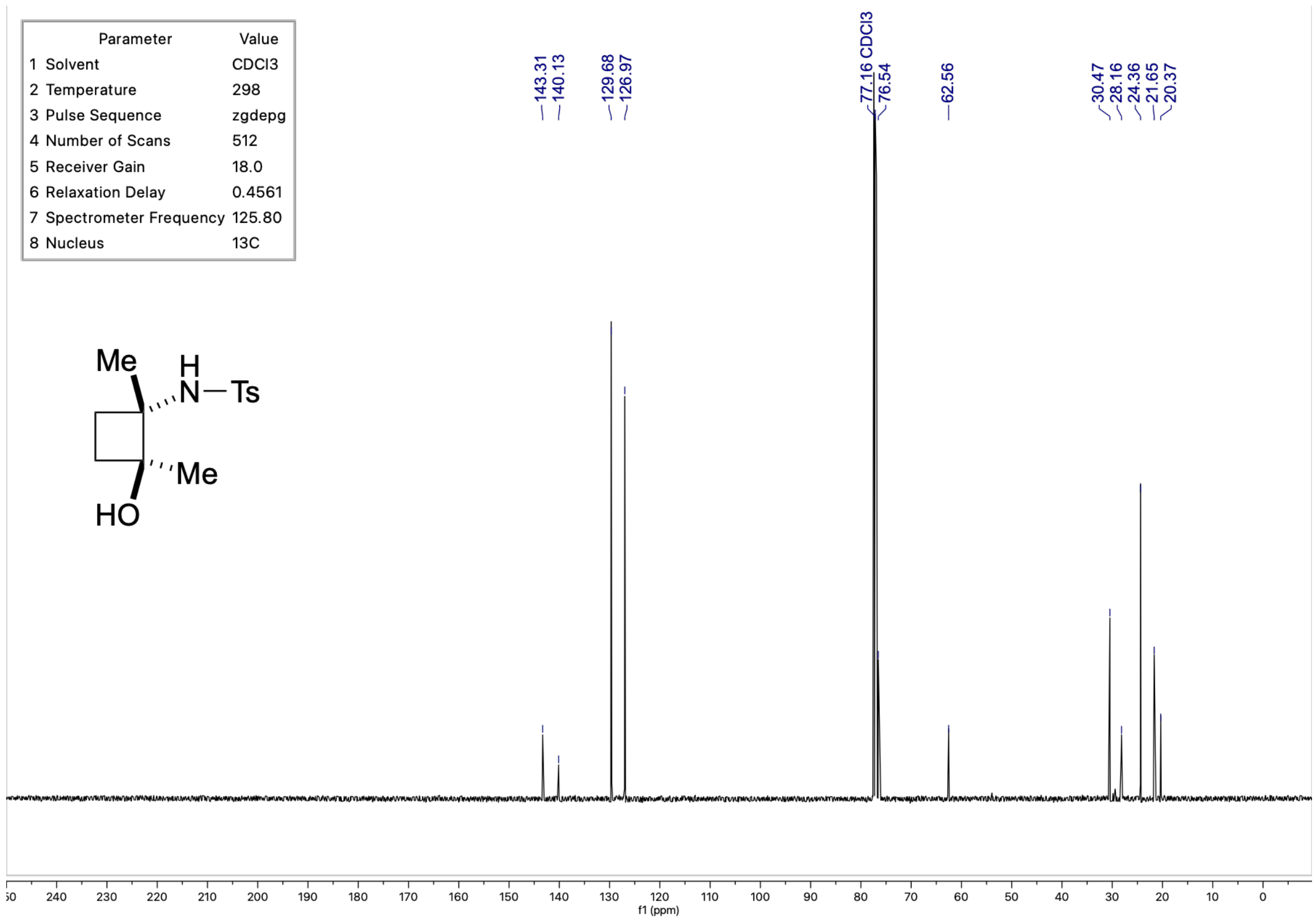
Parameter Value
1 Solvent CDCl3
2 Temperature 298
3 Pulse Sequence zgdepg
4 Number of Scans 512
5 Receiver Gain 18.0
6 Relaxation Delay 0.4561
7 Spectrometer Frequency 125.80
8 Nucleus 13C
143.31
140.13
129.68
126.97
77.16 CDCl3
76.54
62.56
30.47
28.16
24.36
21.65
20.37
Me
H
N—Ts
Me
HO
f1 (ppm)

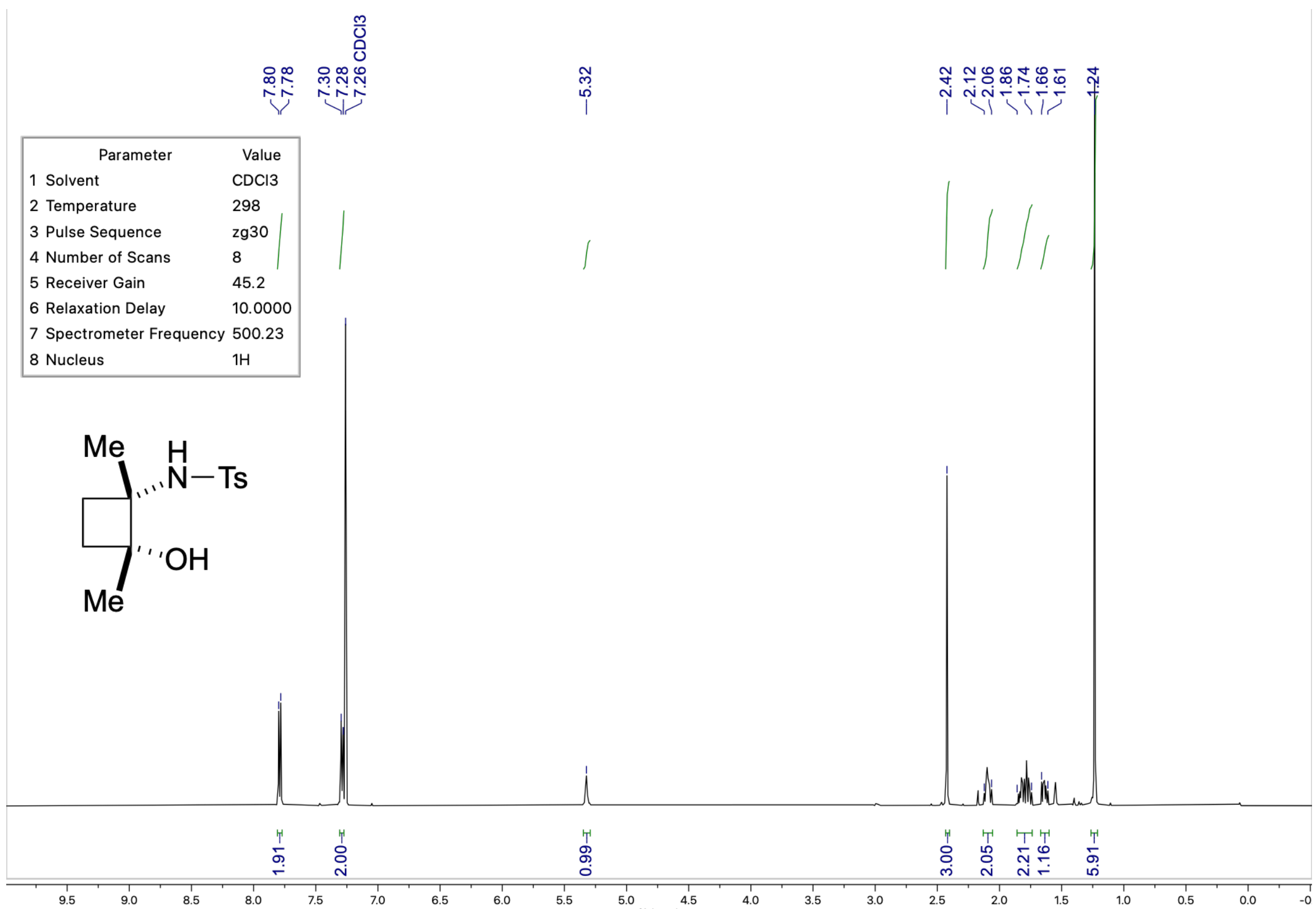
7.80
7.78
7.30
7.28
7.26 CDCl3
5.32
2.42
2.12
2.06
1.86
1.74
1.66
1.61
1.24
Parameter Value
1 Solvent CDCl3
2 Temperature 298
3 Pulse Sequence zg30
4 Number of Scans 8
5 Receiver Gain 45.2
6 Relaxation Delay 10.0000
7 Spectrometer Frequency 500.23
8 Nucleus 1H
Me
H
N—Ts
OH
Me
1.91
2.00
0.99
3.00
2.05
2.21
1.16
5.91
f1 (ppm)

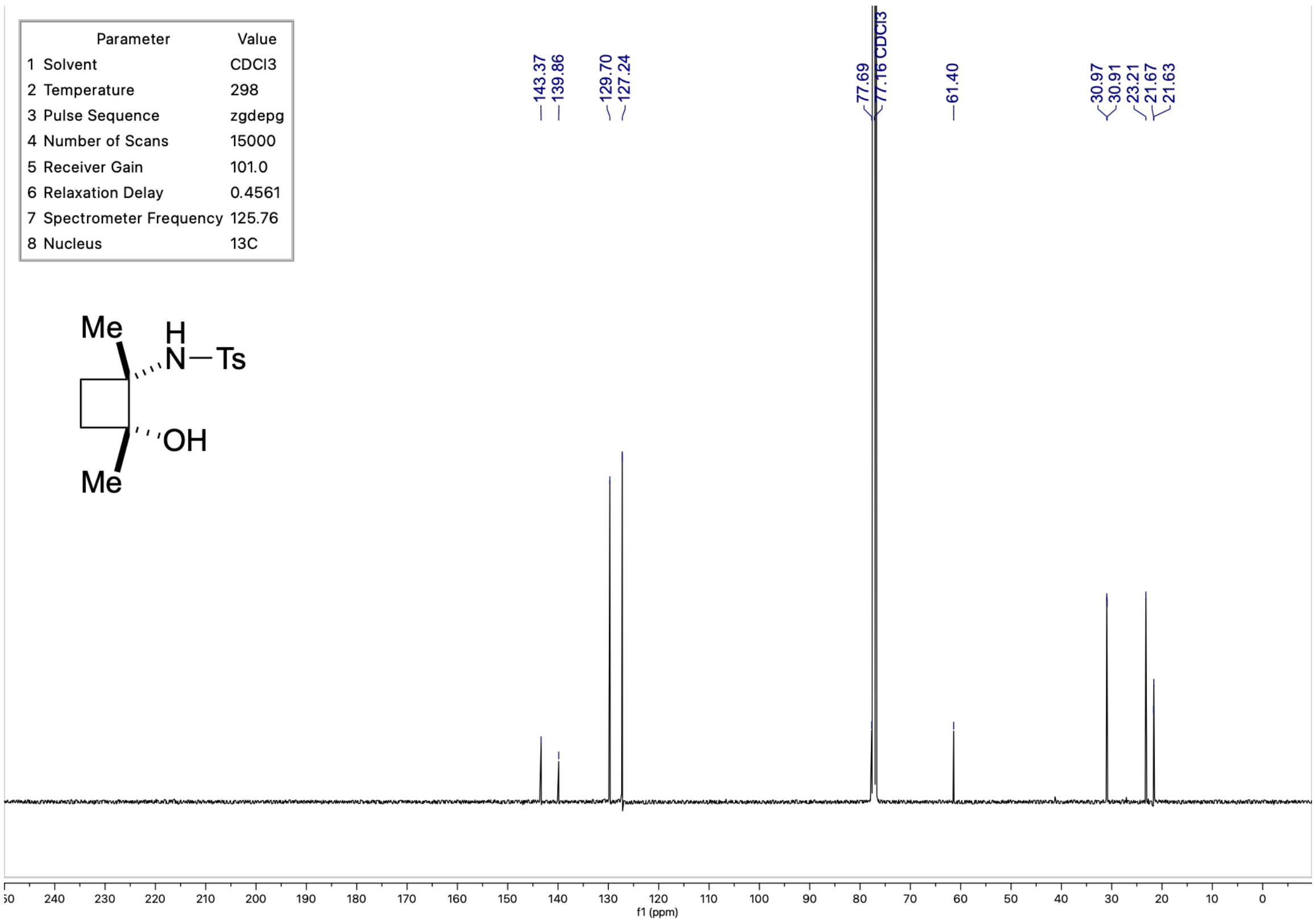

	Parameter	Value
1	Solvent	CDCl3
2	Temperature	298
3	Pulse Sequence	zgdepg
4	Number of Scans	15000
5	Receiver Gain	101.0
6	Relaxation Delay	0.4561
7	Spectrometer Frequency	125.76
8	Nucleus	13C

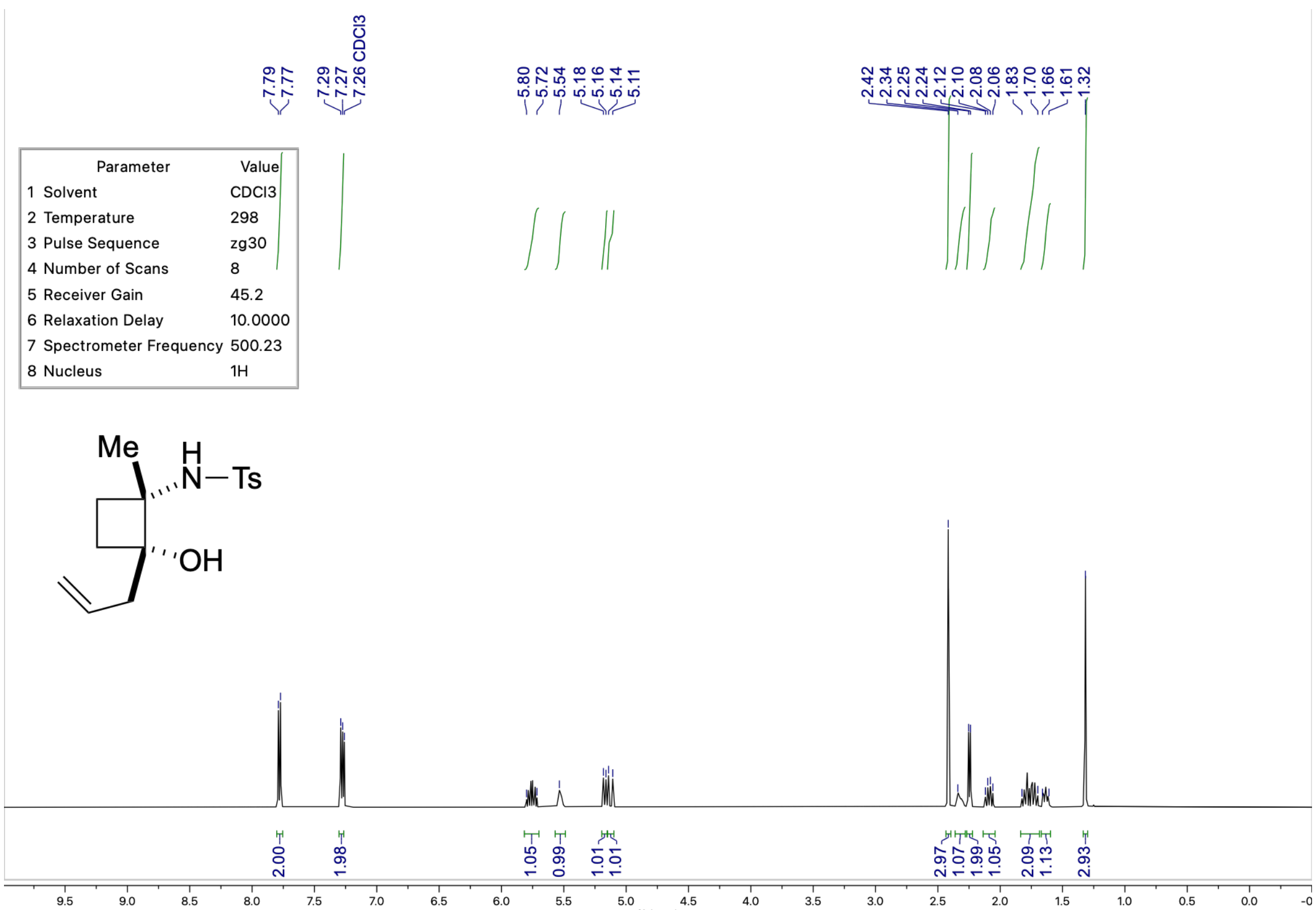

	Parameter	Value
1	Solvent	CDCl3
2	Temperature	298
3	Pulse Sequence	zg30
4	Number of Scans	8
5	Receiver Gain	45.2
6	Relaxation Delay	10.0000
7	Spectrometer Frequency	500.23
8	Nucleus	1H

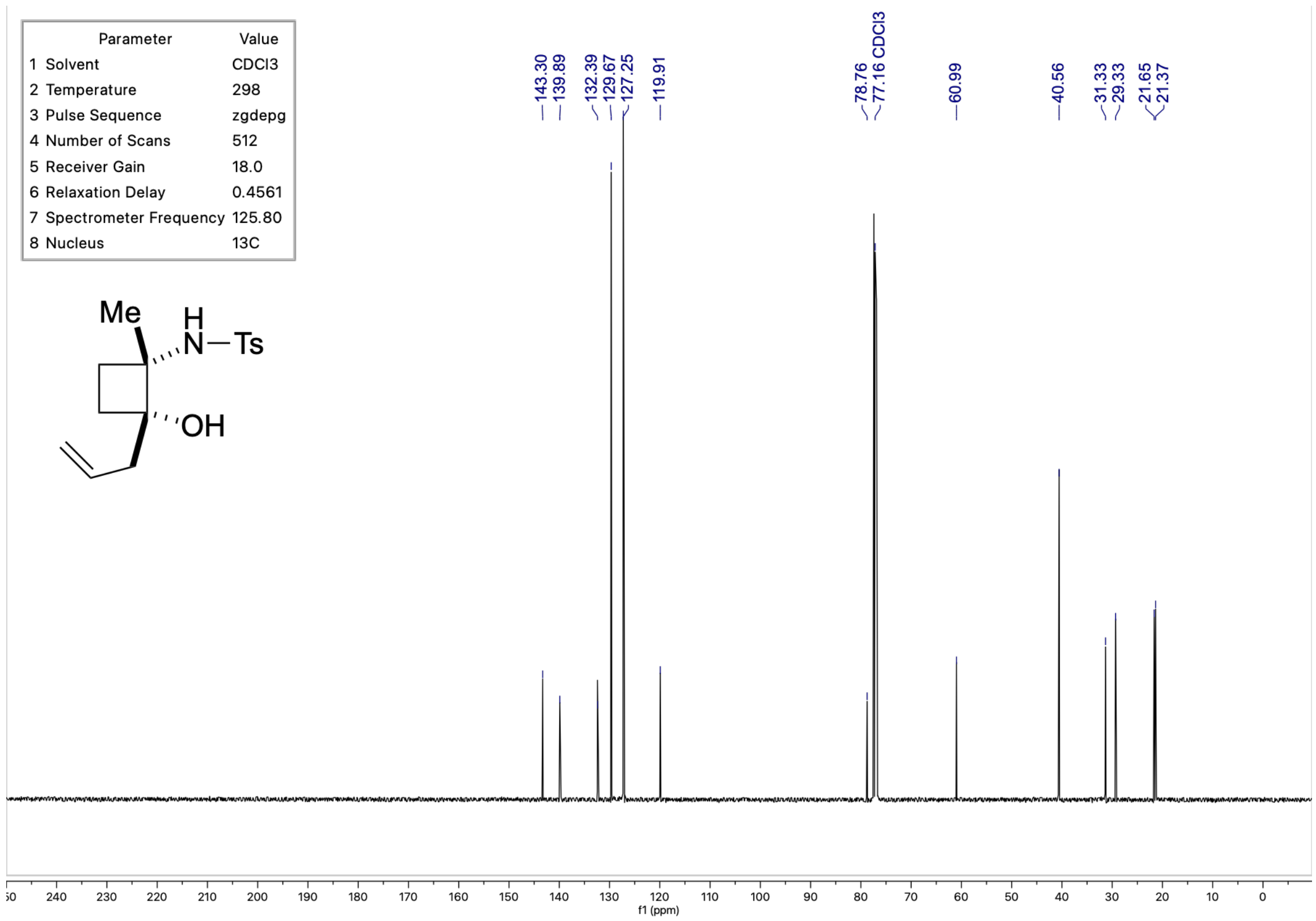
Parameter Value
1 Solvent CDCl3
2 Temperature 298
3 Pulse Sequence zgdepg
4 Number of Scans 512
5 Receiver Gain 18.0
6 Relaxation Delay 0.4561
7 Spectrometer Frequency 125.80
8 Nucleus 13C
Me
H
N—Ts
OH
143.30
139.89
132.39
129.67
127.25
119.91
78.76
77.16 CDCl3
60.99
40.56
31.33
29.33
21.65
21.37
f1 (ppm)

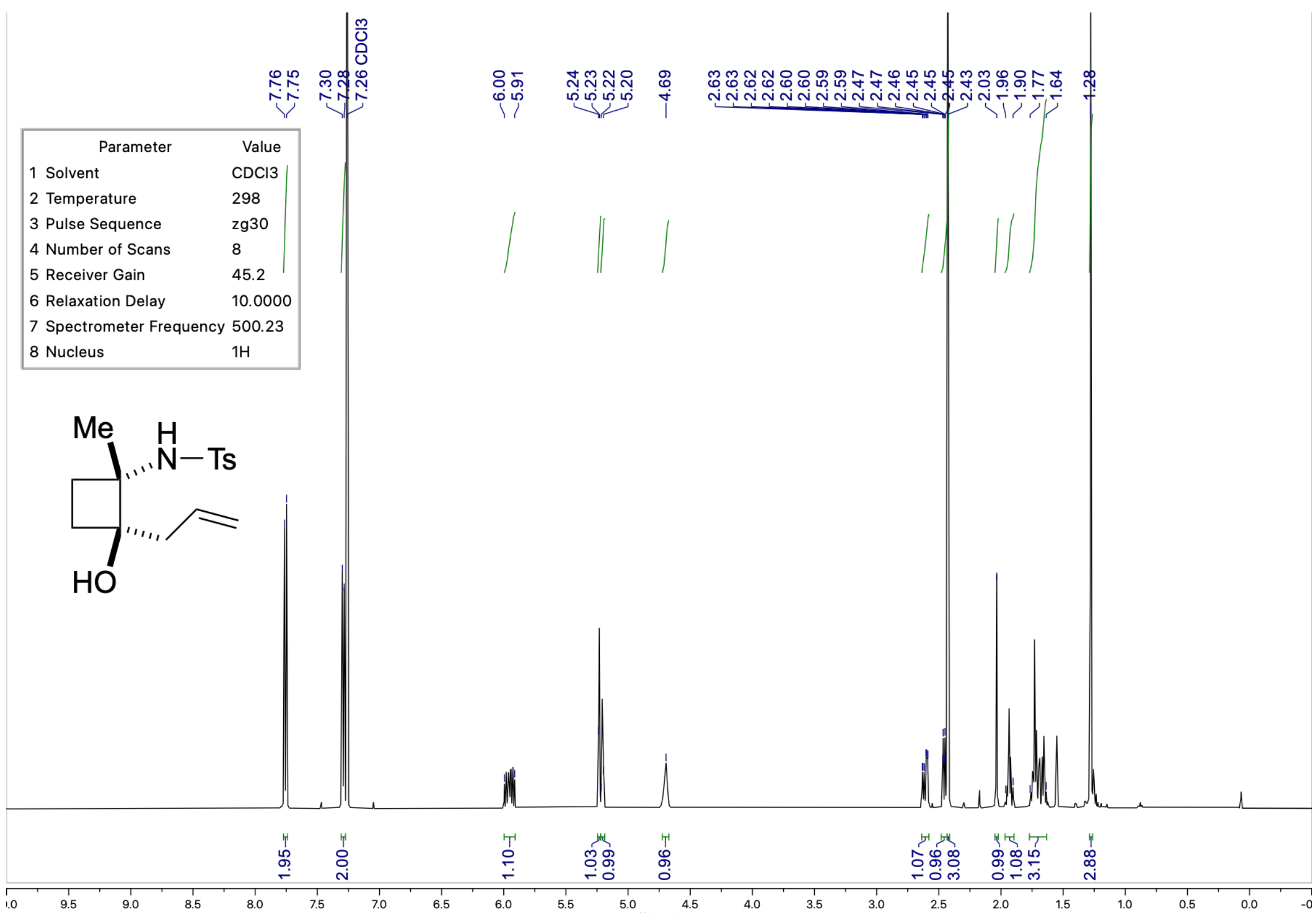
Parameter Value
1 Solvent CDCl3
2 Temperature 298
3 Pulse Sequence zg30
4 Number of Scans 8
5 Receiver Gain 45.2
6 Relaxation Delay 10.0000
7 Spectrometer Frequency 500.23
8 Nucleus 1H
Me
H
N—Ts
HO
7.76
7.75
7.30
7.28
7.26 CDCl3
6.00
5.91
5.24
5.23
5.22
5.20
4.69
2.63
2.63
2.62
2.62
2.60
2.60
2.59
2.59
2.47
2.46
2.45
2.45
2.43
2.43
2.03
1.96
1.90
1.77
1.64
1.28
1.95
2.00
1.10
1.03
0.99
0.96
1.07
0.96
3.08
0.99
1.08
3.15
2.88
f1 (ppm)

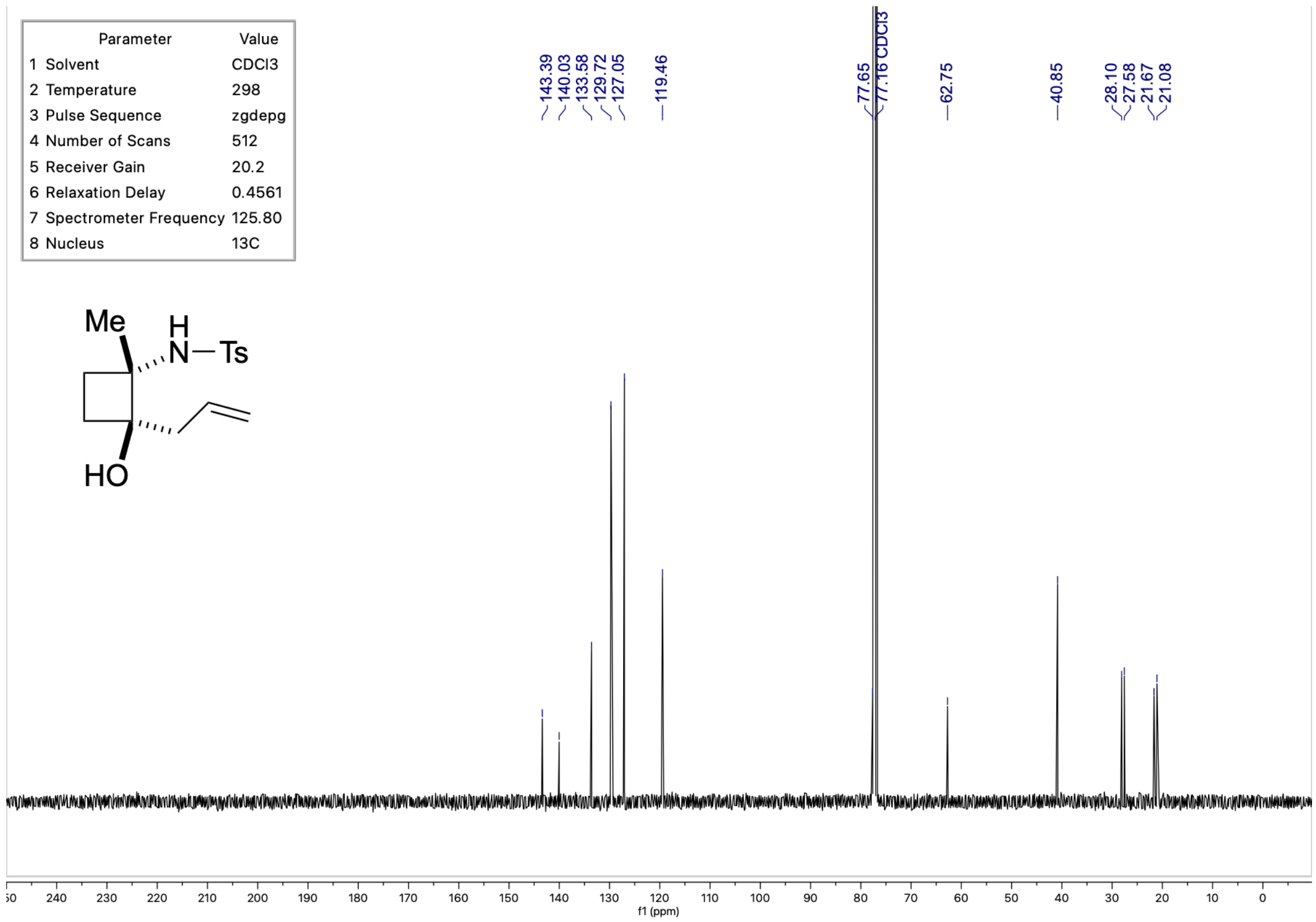
Parameter Value
1 Solvent CDCl3
2 Temperature 298
3 Pulse Sequence zgdepg
4 Number of Scans 512
5 Receiver Gain 20.2
6 Relaxation Delay 0.4561
7 Spectrometer Frequency 125.80
8 Nucleus 13C
Me
H
N—Ts
HO
143.39 140.03 133.58 129.72 127.05 119.46
77.65 77.16 CDCl3
62.75
40.85
28.10 27.58 21.67 21.08
f1 (ppm)

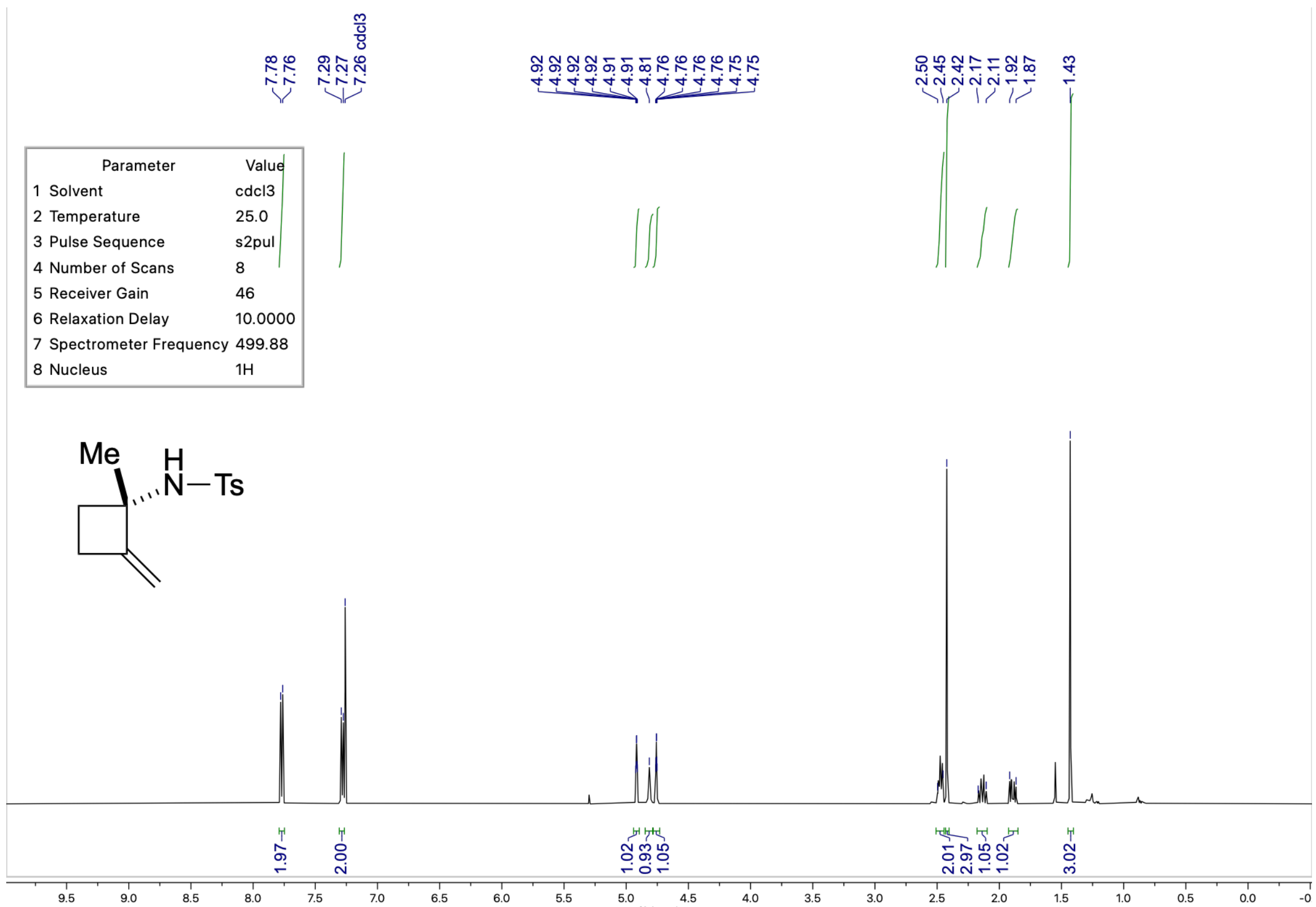
Parameter Value
1 Solvent cdcl3
2 Temperature 25.0
3 Pulse Sequence s2pul
4 Number of Scans 8
5 Receiver Gain 46
6 Relaxation Delay 10.0000
7 Spectrometer Frequency 499.88
8 Nucleus 1H
Me
H
N—Ts
7.78 7.76 7.29 7.27 7.26 cdcl3
4.92 4.92 4.92 4.92 4.91 4.91 4.81 4.76 4.76 4.76 4.76 4.75 4.75
2.50 2.45 2.42 2.17 2.11 1.92 1.87
1.43
1.97 2.00 1.02 0.93 1.05 2.01 2.97 1.05 1.02 3.02
f1 (ppm)

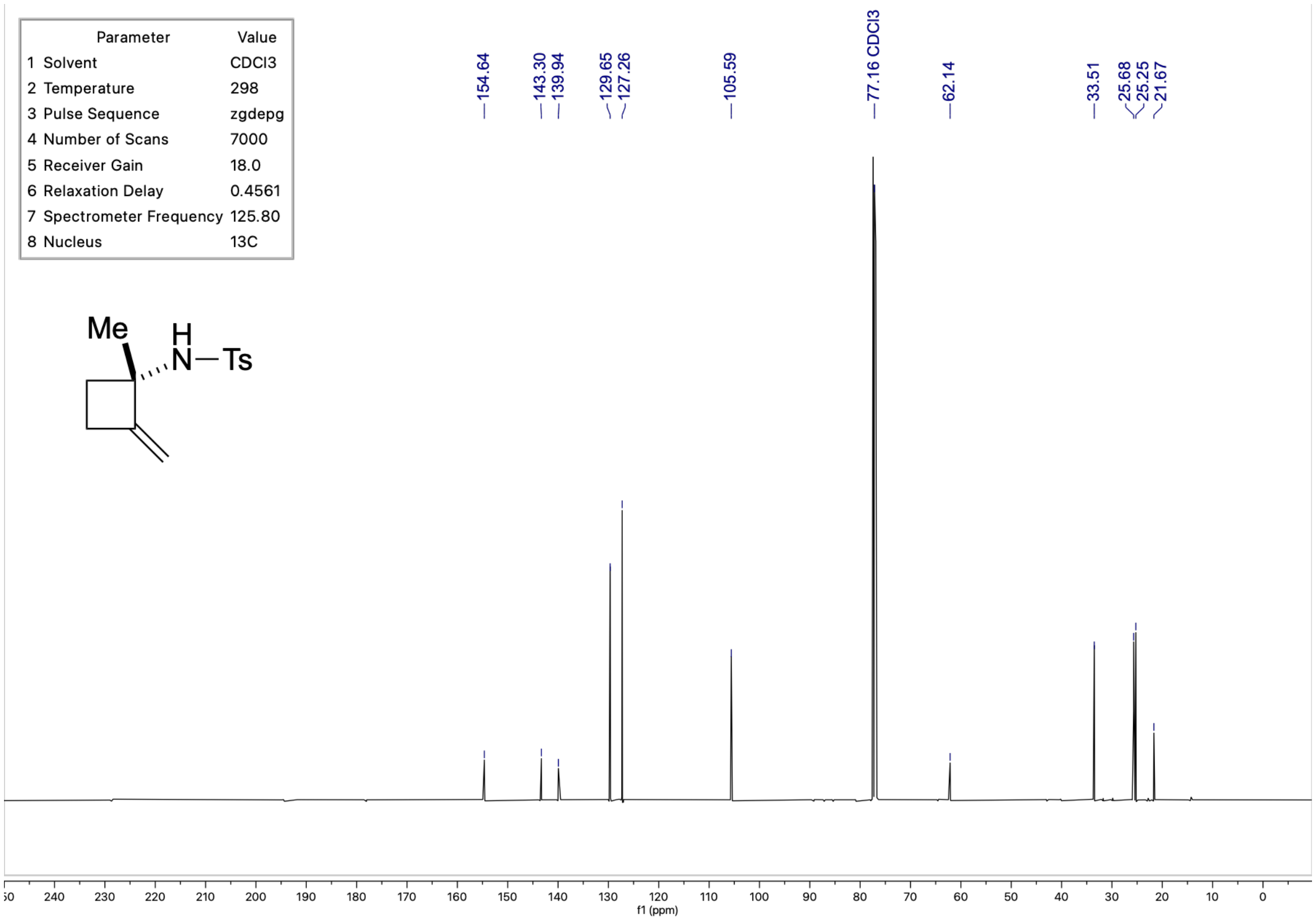

Parameter	Value
1 Solvent	CDCl3
2 Temperature	298
3 Pulse Sequence	zgdepg
4 Number of Scans	7000
5 Receiver Gain	18.0
6 Relaxation Delay	0.4561
7 Spectrometer Frequency	125.80
8 Nucleus	13C

2D NMR Spectra for Stereochemistry Determination

NMR Analysis of Compound **27b** (^{1}H NMR in acetone-d_6; 1D-COSY; 2D-NOESY)

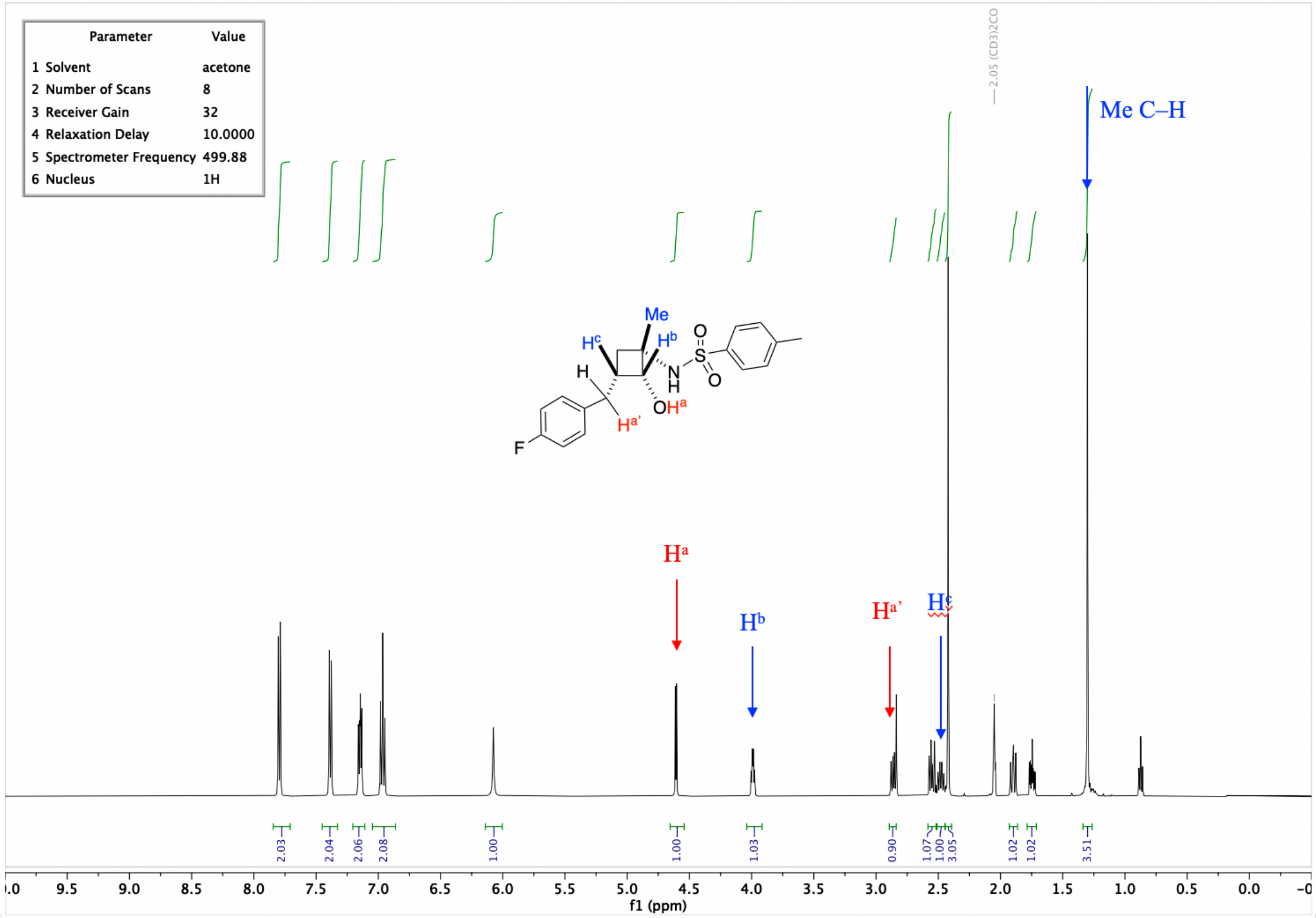

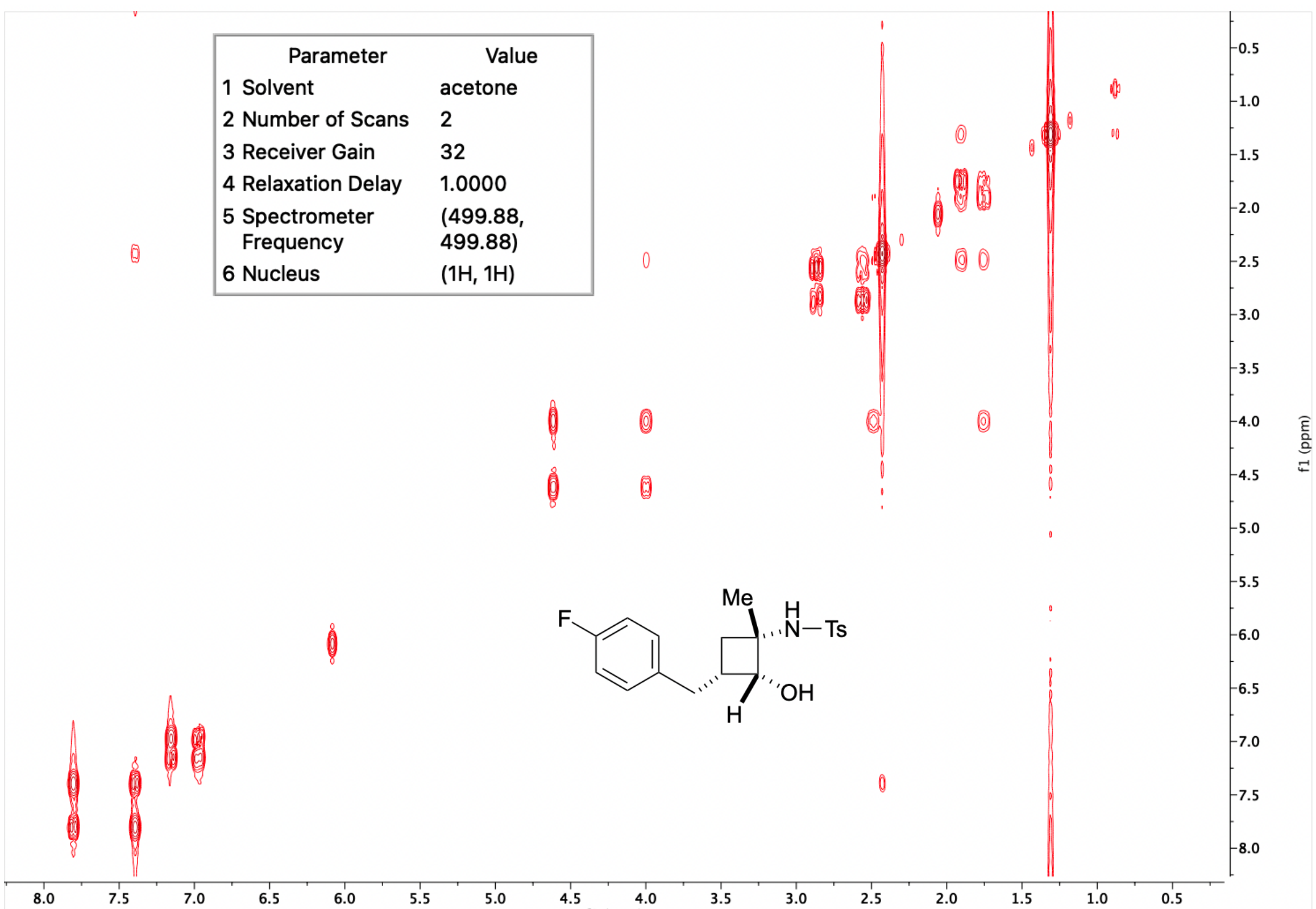

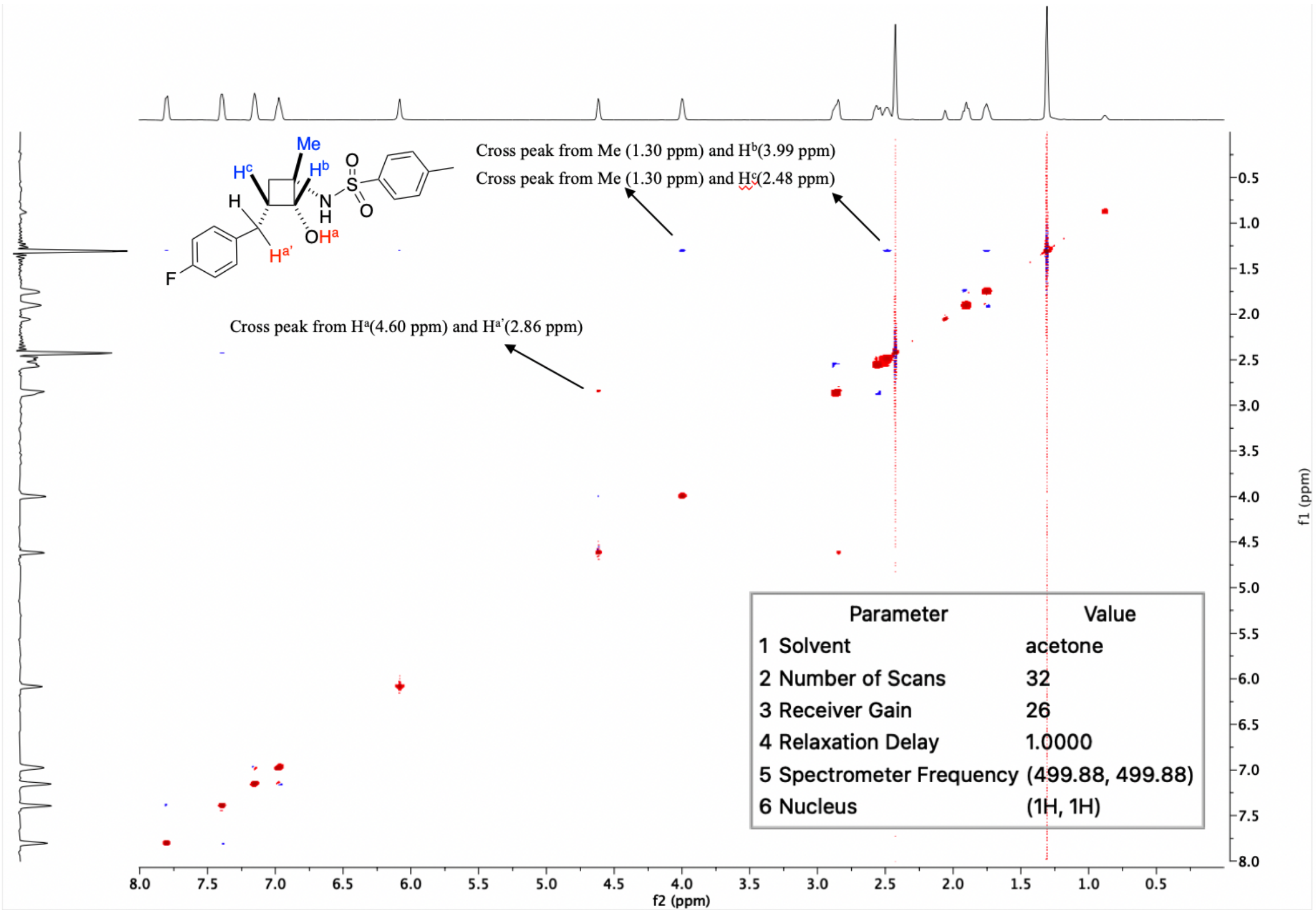

Cross peak from Me (1.30 ppm) and Hb(3.99 ppm)
Cross peak from Me (1.30 ppm) and Hc(2.48 ppm)
Cross peak from Ha(4.60 ppm) and Ha'(2.86 ppm)
Me
Hc
Hb
H
N
H
S
O
O
OHa
Ha'
F
Parameter Value
1 Solvent acetone
2 Number of Scans 32
3 Receiver Gain 26
4 Relaxation Delay 1.0000
5 Spectrometer Frequency (499.88, 499.88)
6 Nucleus (1H, 1H)
f2 (ppm)
f1 (ppm)

NMR Analysis of Compound **28b** (^{1}H NMR in $CDCl_3$; 1D-COSY; 2D-NOESY)

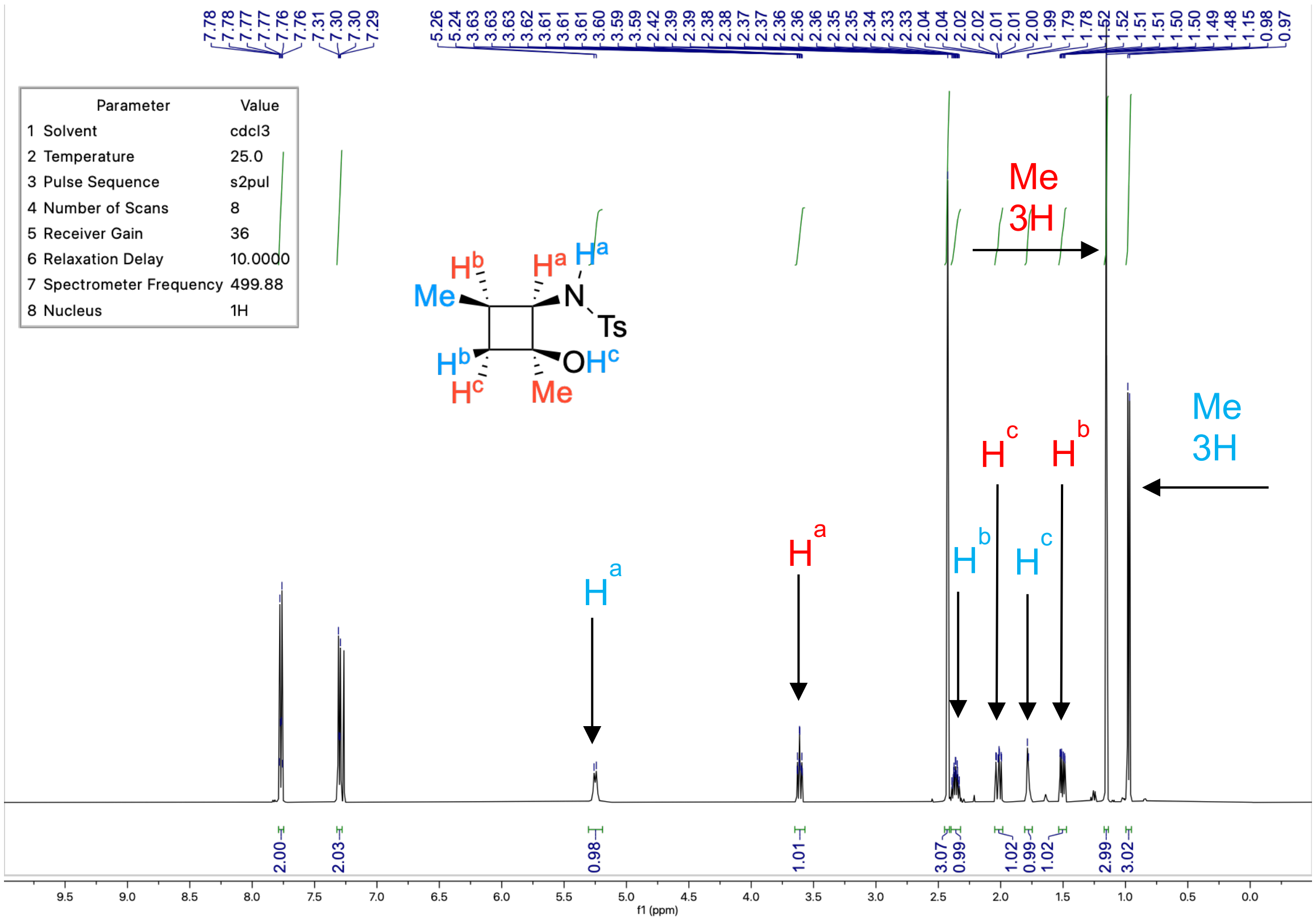

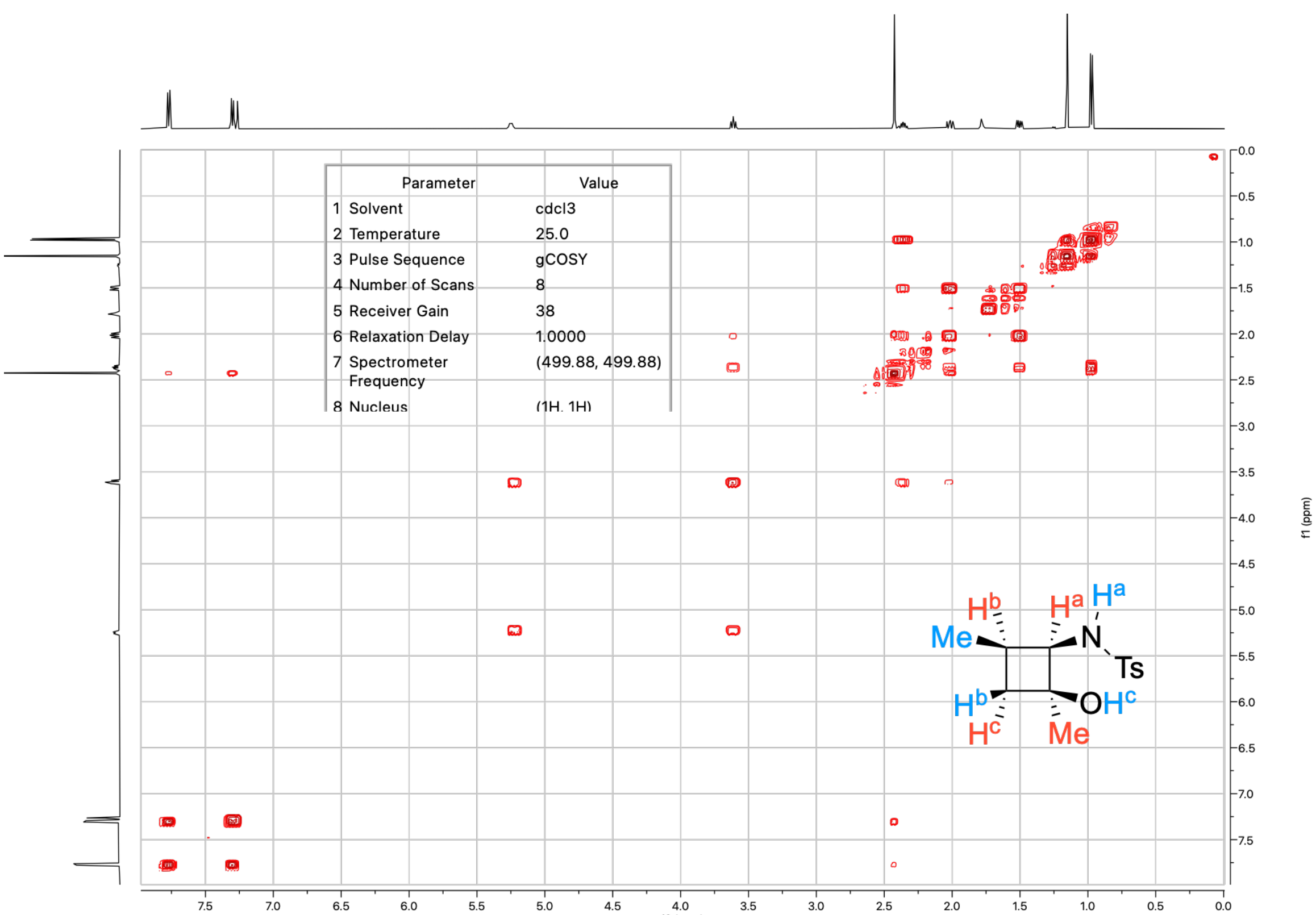

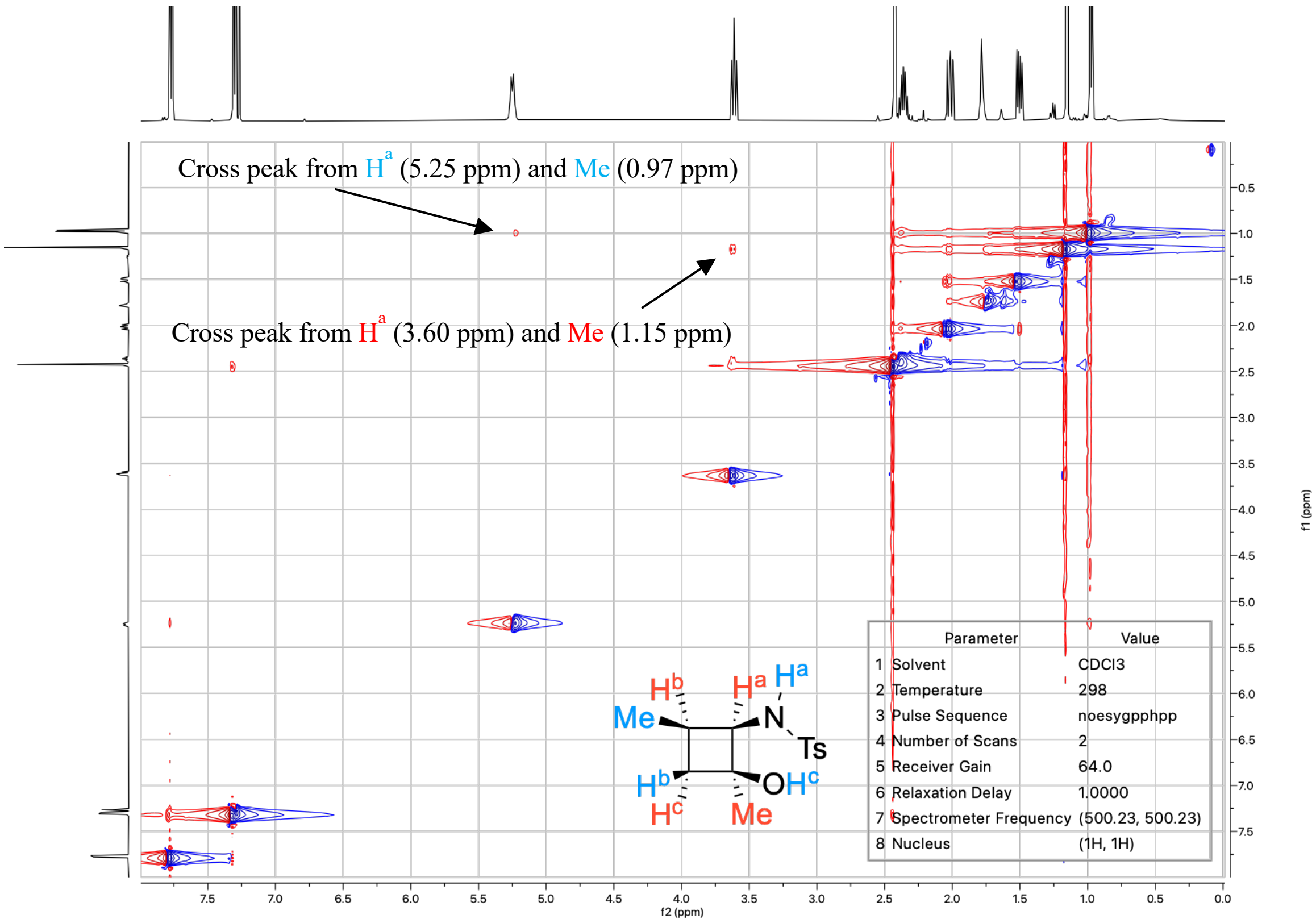
Cross peak from Ha (5.25 ppm) and Me (0.97 ppm)
Cross peak from Ha (3.60 ppm) and Me (1.15 ppm)
Hb
Ha
Ha
Me
N
Ts
Hb
OHc
Hc
Me
Parameter
Value
1 Solvent
CDCl3
2 Temperature
298
3 Pulse Sequence
noesygpphpp
4 Number of Scans
2
5 Receiver Gain
64.0
6 Relaxation Delay
1.0000
7 Spectrometer Frequency
(500.23, 500.23)
8 Nucleus
(1H, 1H)
f2 (ppm)
f1 (ppm)

NMR Analysis of Compound **49** (^{1}H NMR in $CDCl_3$; 1D-COSY; 2D-NOESY)

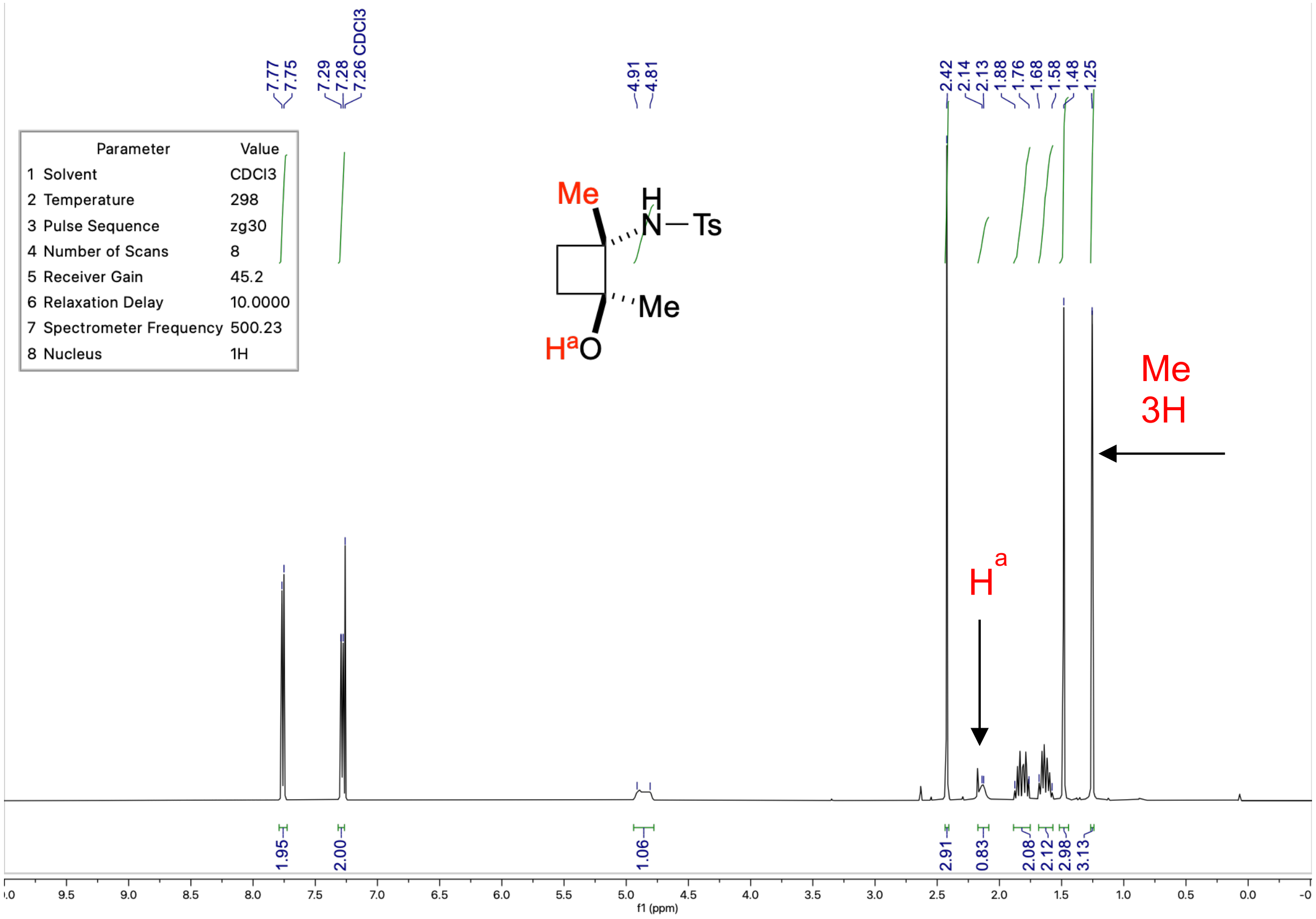

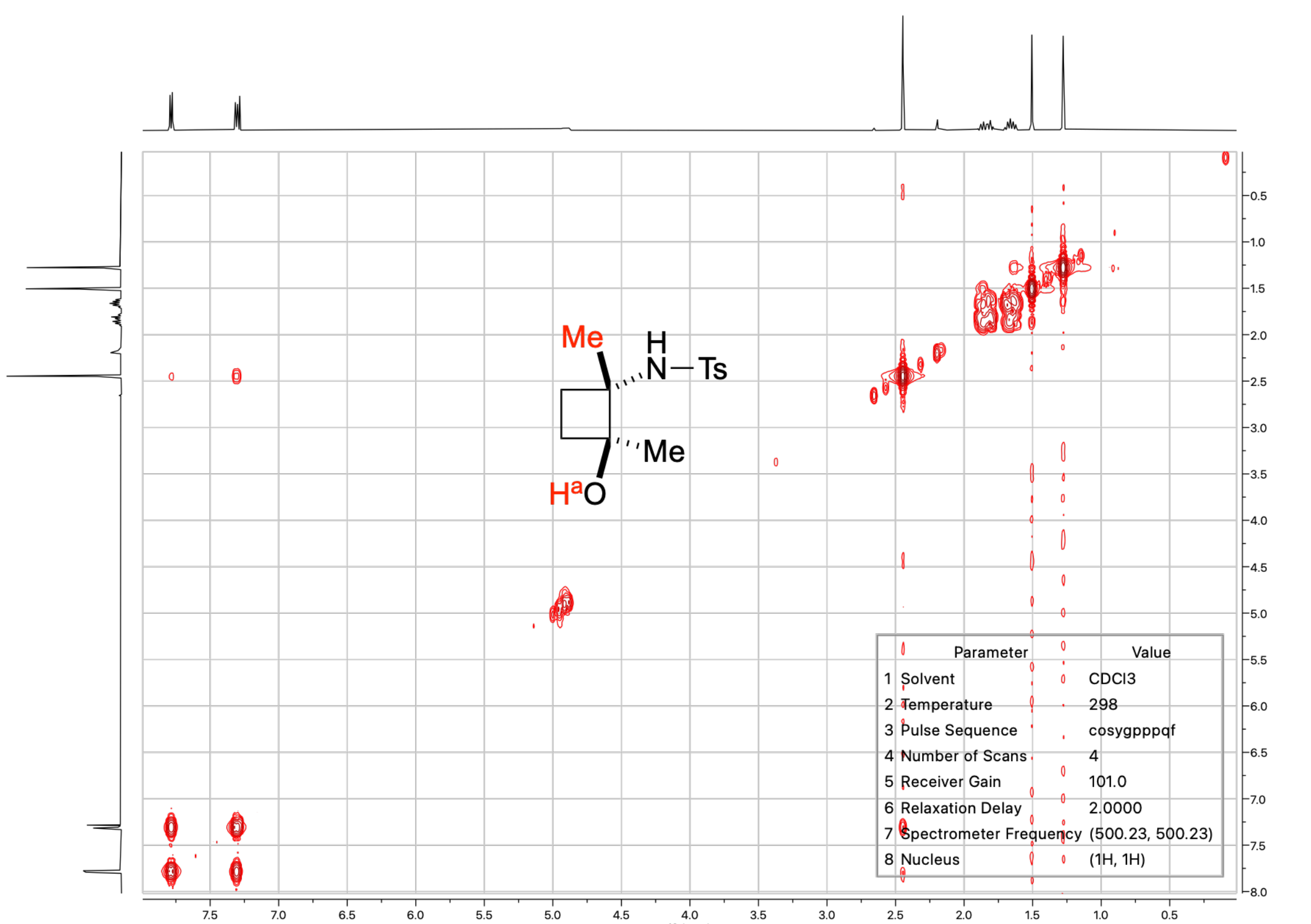

2D-NOESY

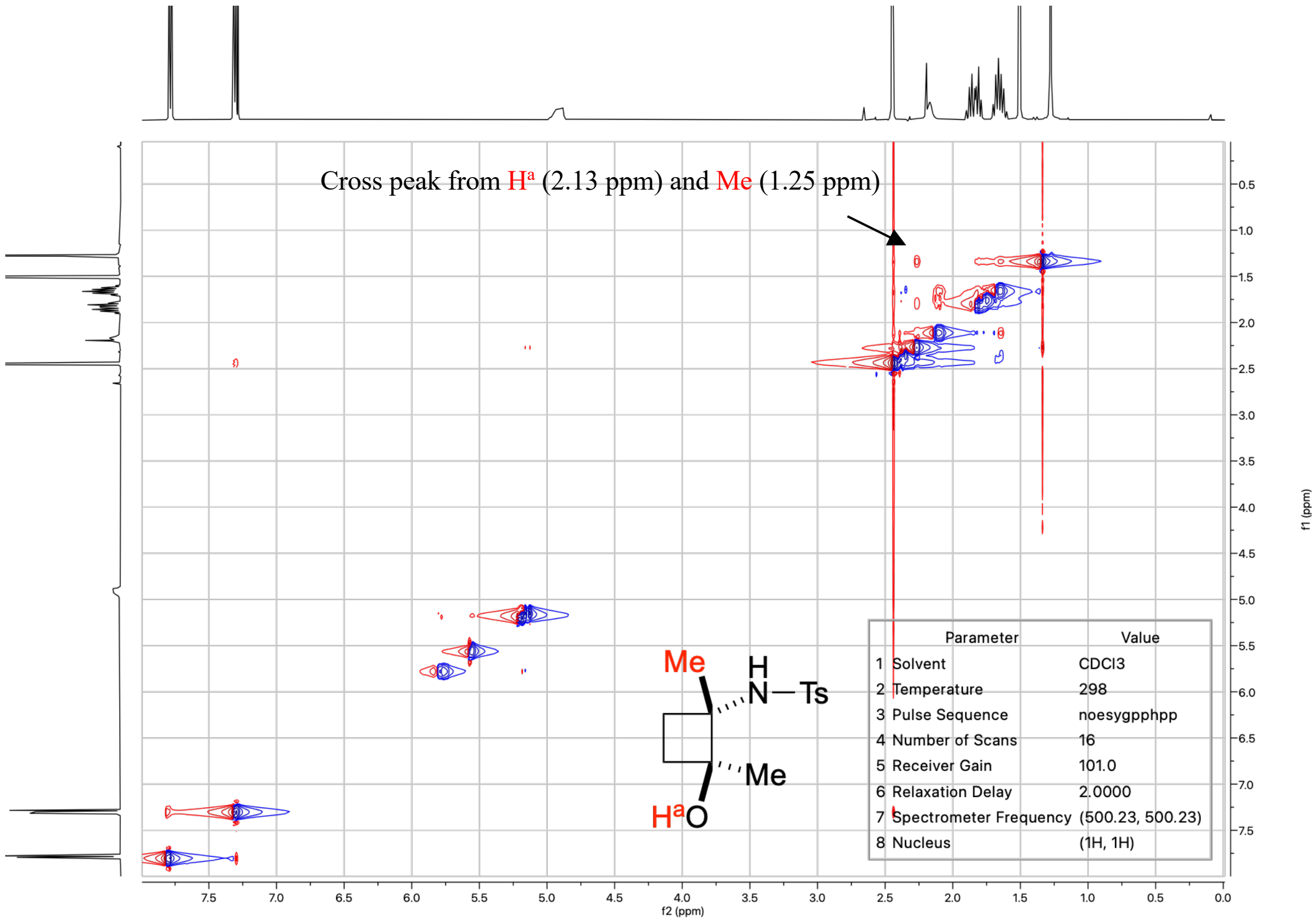

Cross peak from Hª (2.13 ppm) and Me (1.25 ppm)
Me
H
N—Ts
Me
HªO
Parameter
Value
1 Solvent
CDCl3
2 Temperature
298
3 Pulse Sequence
noesygpphpp
4 Number of Scans
16
5 Receiver Gain
101.0
6 Relaxation Delay
2.0000
7 Spectrometer Frequency
(500.23, 500.23)
8 Nucleus
(1H, 1H)
f2 (ppm)
f1 (ppm)

NMR Analysis of Compound **51** (^{1}H NMR in $CDCl_3$; 1D-COSY; 2D-NOESY)

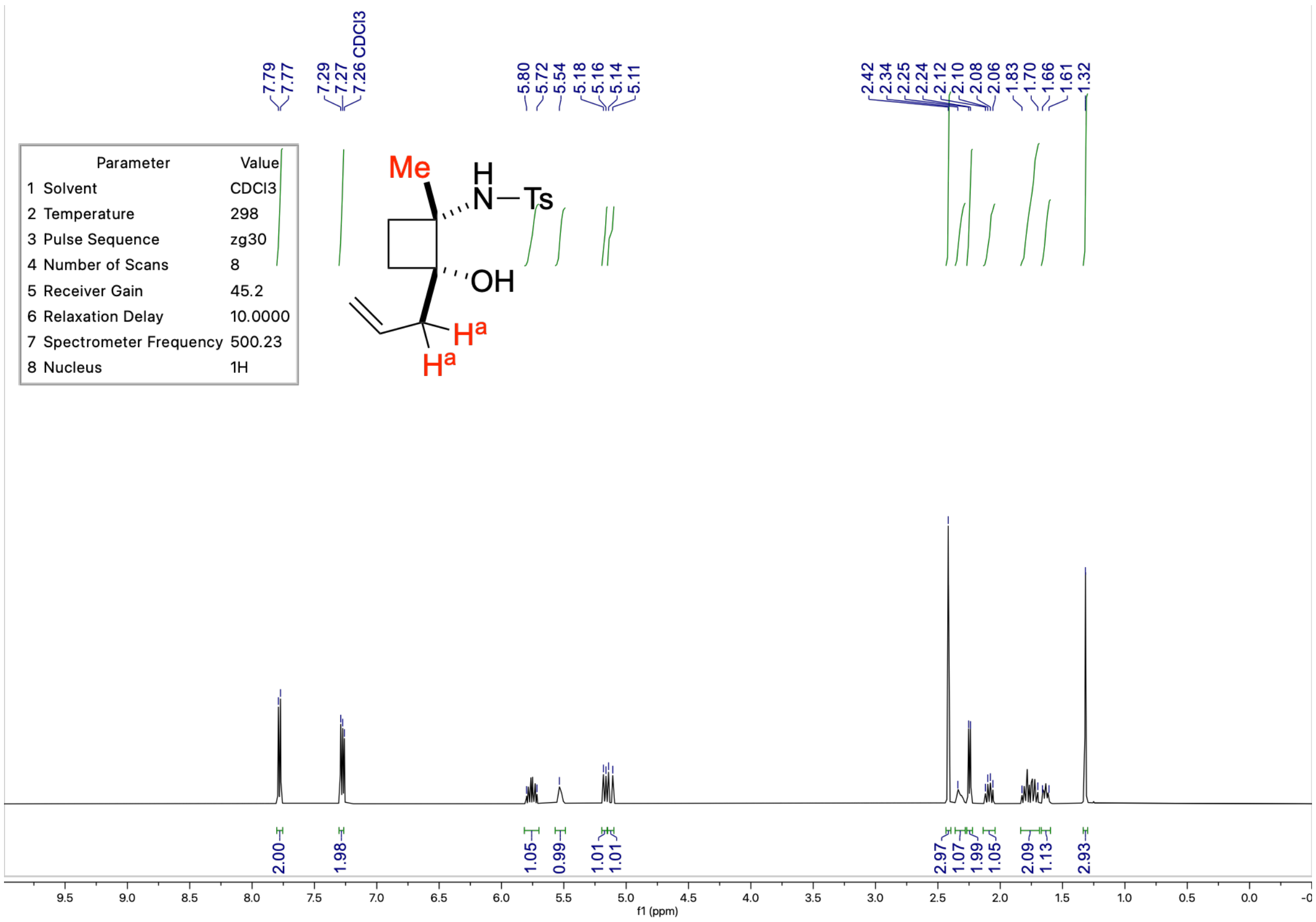

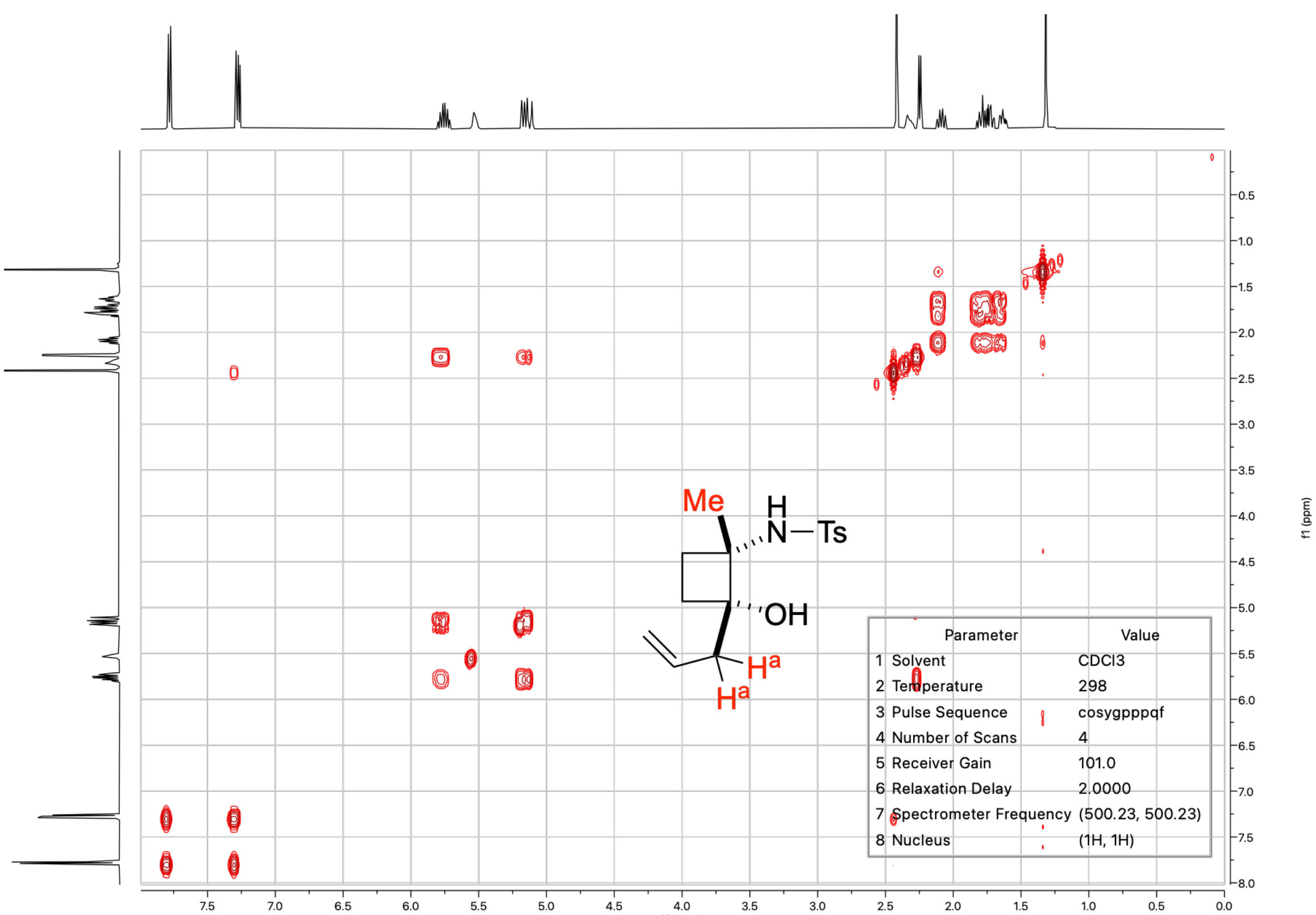

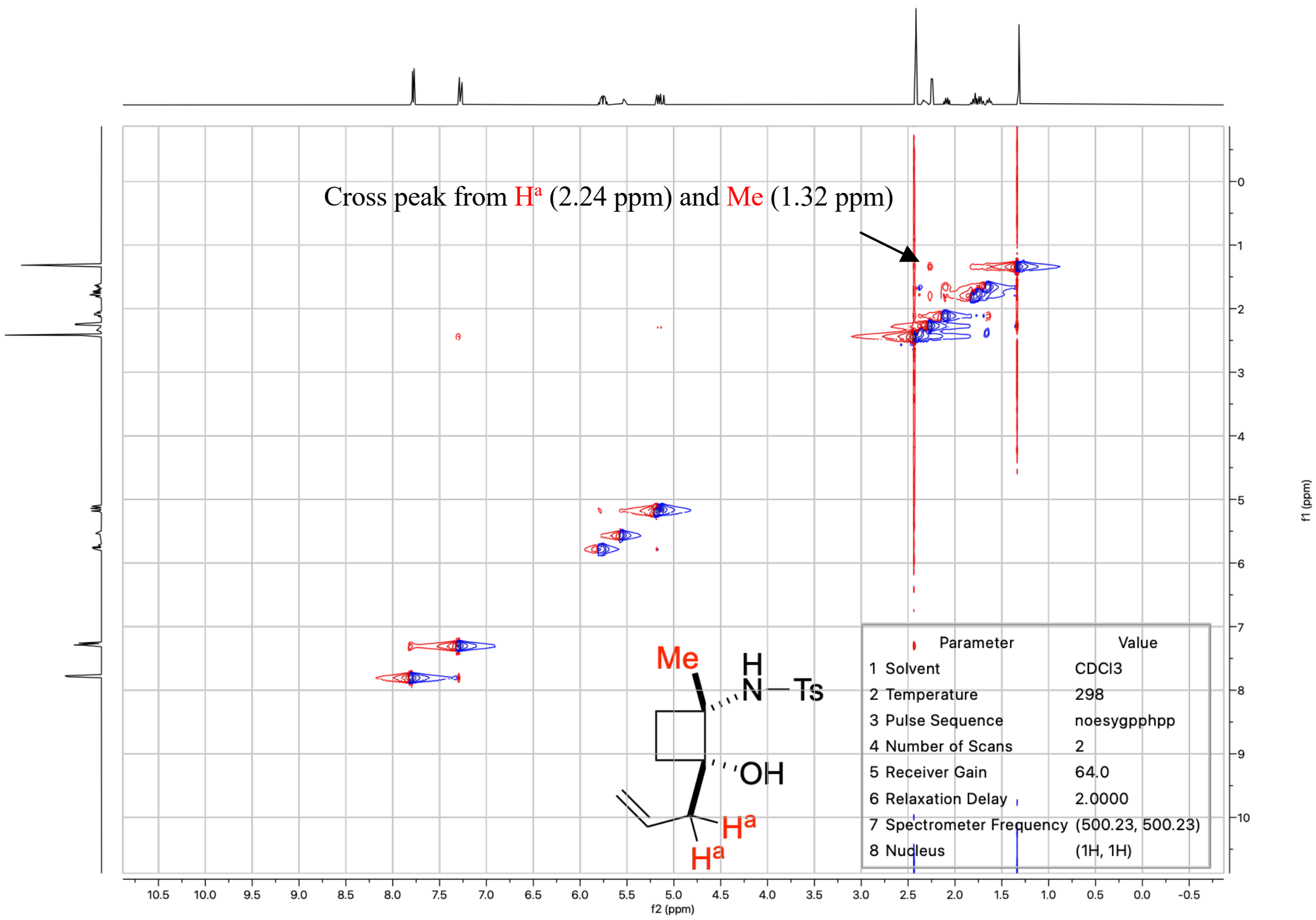
Cross peak from Ha (2.24 ppm) and Me (1.32 ppm)
Me
H
N—Ts
OH
Ha
Ha
Parameter
Value
1 Solvent
CDCl3
2 Temperature
298
3 Pulse Sequence
noesygpphpp
4 Number of Scans
2
5 Receiver Gain
64.0
6 Relaxation Delay
2.0000
7 Spectrometer Frequency
(500.23, 500.23)
8 Nucleus
(1H, 1H)
f2 (ppm)
f1 (ppm)
